# Atom-economic and stereoselective catalytic synthesis of fully substituted enol esters/carbonates of amides in acyclic systems enabled by boron Lewis acid catalysis†

**DOI:** 10.1039/d3sc01394d

**Published:** 2023-04-26

**Authors:** Yuanjiu Xiao, Lei Tang, Tong-Tong Xu, Jiang-Yi-Hui Sheng, Zhongyan Zhou, Lei Yue, Guoqiang Wang, Martin Oestreich, Jian-Jun Feng

**Affiliations:** a State Key Laboratory of Chemo/Biosensing and Chemometrics, Advanced Catalytic Engineering Research Center of the Ministry of Education, College of Chemistry and Chemical Engineering, Hunan University Changsha Hunan 410082 P. R. China jianjunfeng@hnu.edu.cn; b College of Biology, Mass Spectrometry Lab of Bio-Chemistry, Hunan University P. R. China; c Key Laboratory of Mesoscopic Chemistry of Ministry of Education, Institute of Theoretical and Computational Chemistry, School of Chemistry and Chemical Engineering, Nanjing University Nanjing 210093 P. R. China wangguoqiang710@nju.edu.cn; d Institut für Chemie, Technische Universität Berlin Strasse des 17. Juni 115 10623 Berlin Germany martin.oestreich@tu-berlin.de https://www.tu.berlin/en/organometallics

## Abstract

Carboacyloxylation of internal alkynes is emerging as a powerful and straightforward strategy for enol ester synthesis. However, the reported examples come with limitations, including the utilization of noble metal catalysts, the control of regio- and *Z*/*E* selectivity, and an application in the synthesis of enol carbonates. Herein, a boron Lewis acid-catalyzed intermolecular carboacyloxylation of ynamides with esters to access fully substituted acyclic enol esters in high yield with generally high *Z*/*E* selectivity (up to >96 : 4) is reported. Most importantly, readily available allylic carbonates are also compatible with this difunctionalization reaction, representing an atom-economic, catalytic and stereoselective protocol for the construction of acyclic β,β-disubstituted enol carbonates of amides for the first time. The application of the carboacyloxylation products to decarboxylative allylations provided a ready access to enantioenriched α-quaternary amides. Moreover, experimental studies and theoretical calculations were performed to illustrate the reaction mechanism and rationalize the stereochemistry.

## Abstract 2

A boron Lewis acid catalyzes trans-carboacyloxylations of ynamides with esters to afford fully substituted acyclic enol esters/carbonates in high yields and with excellent stereocontrol.

## Graphical abstract

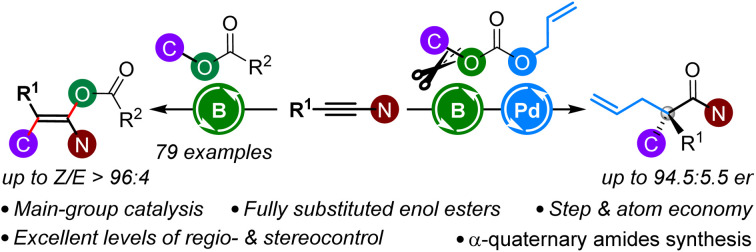

## Introduction

**Scheme 1:**
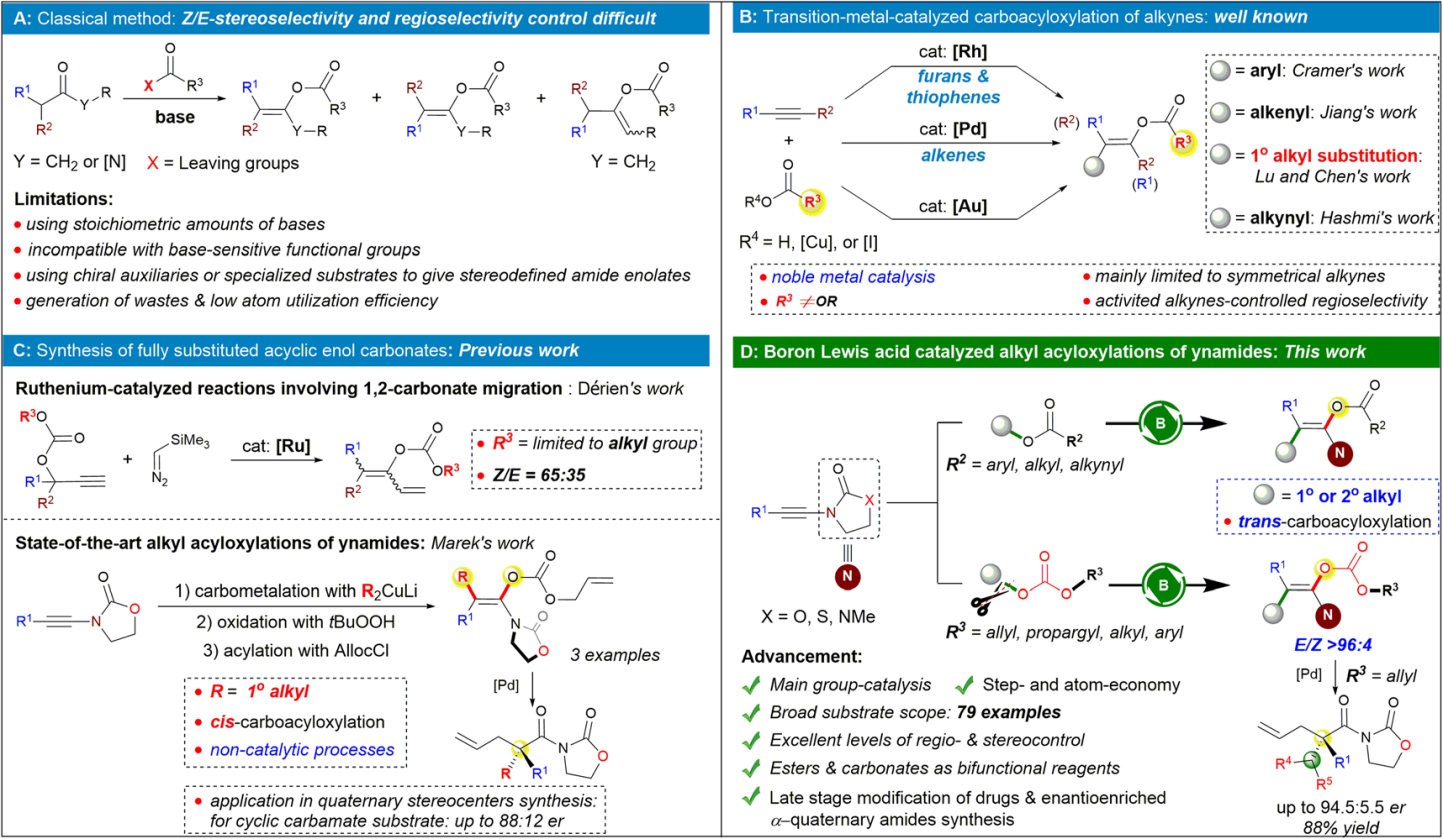
Our catalytic strategy to access fully substituted acyclic enol ester/carbonates and its scientific context.

The chemistry of enolates can be considered one of the cornerstone areas in organic chemistry, driven by this compound class's role as carbon nucleophiles.^1^ As a crucial subclass of enolates endowed with a delicate balance of reactivity and stability, enol ester/carbonate derivatives have proven to be fascinating building blocks due to their versatility for further synthetic transformations such as aldol-^2^ and Mannich-type reactions,^3^ cross-coupling reactions,^4^ asymmetric hydrogenations,^5^ cyclizations,^6^ and decarboxylative allylations.^7^ The enol ester skeleton is also found in an array of natural products and pharmaceuticals.^8^ Due to the importance of enol esters/carbonates, many efforts have been focused on their synthesis. Most of the conventional methods for their preparation rely on α-deprotonation of the corresponding carbonyl compounds and subsequent *O*-acylation of enolates, a route that is typically plagued with regio- or stereoselectivity issues and incompatible with base-sensitive functional groups (Scheme 1A).^9^ Recently, several attractive catalytic approaches for the preparation of enol esters have been reported including the hydroacyloxylation of alkynes,^10^ rearrangements of propargylic esters,^11^ Chan-Lam couplings,^12^ organocatalyzed Michael addition-rearrangement of ynals with carboxylic acids,^13^ and others.^14^ Despite significant progress, construction of fully substituted acyclic enol esters remains limited because of inevitable issues including significant steric hindrance and the difficulty to distribute the various substituents in a stereo- and regioselective way.

Attractive solutions to this problem have been described, and one of the most straightforward catalytic routes to rapidly build these valuable enol esters is the 1,2-difunctionalization of internal alkynes.^15^ For example, the Cramer group performed pioneering work on the heteroaryl acyloxylation of alkynes by cooperative rhodium/copper catalysis. However, the substrates were mainly symmetrical dialkyl alkynes, and the reaction afforded the desired products with poor regioselectivities when unsymmetrically substituted alkynes were used (Scheme 1B, top).^16^ On the other hand, pioneered by Lu, *trans*-acetoxypalladation of alkynes followed by olefin insertion and depalladation of the C–Pd bond was demonstrated by the groups of Lu,^17*a*–*d*^ Chen,^17*e*^ and Jiang^17*f*^ as an efficient method for accessing fully substituted acyclic enol esters (Scheme 1B, middle). It has been reported that unsymmetrical internal alkynes activated by an electron-withdrawing group showed improved regioselectivity for the reaction,^17*c*,*e*^ probably due to the polarization of the triple bonds. However, as for normal unactivated unsymmetrical alkynes, their regioselective carboacyloxylation is still a formidable challenge.

Ynamides are versatile nitrogen-containing alkyne synthons in organic synthesis. Owing to their unique reactivity and regioselectivity, ynamides have been regarded as versatile building blocks to react with diverse starting materials providing a concise and flexible approach to construct various useful nitrogen-containing molecules.^18,19^ Thus, ynamides are accordingly selected as the reaction substrates in the investigation of the 1,2-difunctionalization process.^20^ Along these lines, the Hashmi group reported an impressive example of a regio- and stereoselective intermolecular acyloxyalkynylation of ynamides based on an Au(i)/Au(iii) catalytic cycle using ethynylbenziodoxolones as bifunctional reagents (Scheme 1B, bottom).^21^

Despite the progress made, the carboacyloxylation of alkynes continues to face several challenges such as (a) the carboacyloxylation strategy could not be extended to benzyl acyloxylations and secondary alkyl acyloxylations of alkynes; (b) in contrast to transition-metal catalysis, the complementary main group-catalyzed carboacyloxylation of alkynes in a sustainable manner is still scarce. A landmark in this field was described by Melen and co-workers, yet this reaction proceeds in an intramolecular fashion.^22^ (c) So far, most of the transformations were focused on the carboacyloxylation of alkynes for synthesis of enol esters while less work was reported on the selective delivery of a carbon atom and a carbonate across an alkyne for synthesis of enol carbonates, probably due to the carbonate's ability to undergo decarboxylation.^23^ Marek's group developed a state-of-the-art strategy for preparation of stereodefined acyclic α,α-dialkylsubstituted amide enol carbonates through a regio- and stereoselective carbocupration reaction of ynamides followed by a stereoretentive oxidation with an oxenoid and subsequent enolate trapping with allyl chloroformate (AllocCl; Scheme 1C, bottom).^24^ Of note, other elegant methods for the generation of stereo-defined amide enolates mentioned above typically require highly specialized substrates, often incorporating chiral auxiliaries to impart selectivity in the enolate formation step (Scheme 1A).^25^ Thus, the development of a catalytic protocol for accessing this important structural motif would be highly desirable. As a rare example, Dérien and co-workers reported a ruthenium-catalyzed synthesis of dienyl carbonates from propargylic carbonates and silyl diazo compounds by 1,2-carbonate migration of the propargylic carbonates, affording the desired products with poor to moderate *Z*/*E* ratios (Scheme 1C, top).^26^

An ester is one of the most common functional groups in organic chemistry. Carboxylates, especially carbonate, were always utilized as ideal leaving groups for the electrophile.^27^ In contrast to the studies on the difunctionalization reactions by using carboxylic acids, the successful use of esters as the bifunctional reagents^28^ in difunctionalization of unsaturated hydrocarbons to build molecular complexity still lags behind and had been limited to the intramolecular reactiosn.^29^ In line with our interest in developing atom-economic reactions and main group catalysis,^30^ we report here a complementary and main-group-catalyzed intermolecular 1° and 2° alkyl acyloxylations of ynamides with benzyl carboxylates or carbonates, in which the RCO_2_-C(sp^3^) bond is formally cleaved and added across ynamides to generate the acyclic β,β-disubstituted enol esters/carbonates of amides. The synthetic utility is illustrated by the late-stage modification of natural products and drug derivatives and the construction of acyclic quaternary carbon centers by palladium-catalyzed decarboxylative allylic alkylation of fully substituted amide enolates.

## Results and discussion

### Catalytic synthesis of fully substituted enol esters

Given that geminal diaryl skeletons are prevalent in many natural products and pharmaceuticals,^31^ benzhydryl carboxylate 2a was selected as the model substrate, which can be easily prepared and also serves as a suitable cation precursor.^27*b*,*c*^ Initially, diaryl ester 2a and ynamide 1a were treated with commonly used metal-based Lewis acid catalysts such as Cu(OTf)_2_, Zn(OTf)_2_, and ZnCl_2_ in toluene at 80 °C for 12 h. These catalysts led to no conversion (Table 1, entries 1–3). Using Sc(OTf)_3_ or BF_3_·Et_2_O as the catalyst, a mixture of 3aa with a poor *Z*/*E* ratio and yield and some hydro-oxycarbonylation side product 4aa was obtained (entries 4 and 5). Brønsted acid catalyst TfOH failed to afford the desired product (entry 6). In light of Komeyama's and Takaki's^29*d*^ as well as Melen's^22^ elegant work on intramolecular carbo-oxycarbonylation of alkynyl esters, we tried to perform the current intermolecular 1,2-difunctionalization reaction by means of bismuth and boron Lewis acid catalysis, respectively.^32–34^ Gratifyingly, with the use of B(C_6_F_5_)_3_, the reaction provided the desired stereodefined fully substituted enol ester (*Z*)-3aa in 94% NMR yield (entry 8). The structure and configuration of (*Z*)-3aa were confirmed by X-ray diffraction.^35^ In contrast, Bi(OTf)_3_ failed to give the desired product (entry 7 *versus* 8). A decrease in the yield and stereoselectivity was observed when the reaction was conducted at 60 °C (entry 9) or in the presence of a hindered Lewis basic phosphine (entries 10 and 11).

**Table 1:** Selected examples of the optimization of the alkyl acyloxylations of ynamides^a^

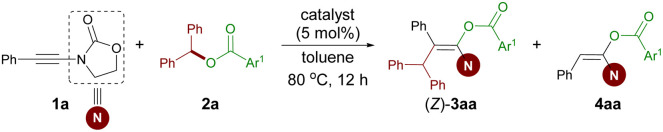					
Entry	Catalyst	Conv.^b^ [%]	(*Z*)-3aa^b^^,^^c^ [%]	(*Z*)-3aa : (*E*)-3aa^b^	4aa^b^^,^^c^ [%]
1	Cu(OTf)_2_	0	—	—	—
2	Zn(OTf)_2_	0	—	—	—
3	ZnCl_2_	0	—	—	—
4	Sc(OTf)_3_	25	<5	75 : 25	10
5	BF_3_·Et_2_O	20	12	79 : 21	8
6	TfOH	100	0	—	15
7	Bi(OTf)_3_	13	0	—	9
**8**	**B(C**_**6**_ **F**_**5**_ **)**_**3**_	**100**	**94**	**>96 : 4**	**5**
9^d^	B(C_6_F_5_)_3_	13	9	90 : 10	3
10^e^	B(C_6_F_5_)_3_	82	64	93 : 7	11
11^f^	B(C_6_F_5_)_3_	96	65	78 : 22	4

a Unless otherwise noted, the reactions were performed with 1a (0.24 mmol), 2a (Ar^1^ = 4-FC_6_H_4_; 0.2 mmol) and catalyst (5.0 mol%) in toluene (2 mL) at 80 °C for 12 h.

b Determined by ^1^H NMR spectroscopy of the crude reaction mixture with CH_2_Br_2_ as an internal standard.

c NMR yield.

d The reaction was run at 60 °C.

e 5 mol% B(C_6_F_5_)_3_ and 5 mol% Mes_3_P used.

f B(C_6_F_5_)_3_ (0.2 mmol) and Mes_3_P (0.2 mmol) used.

**Scheme 2:**
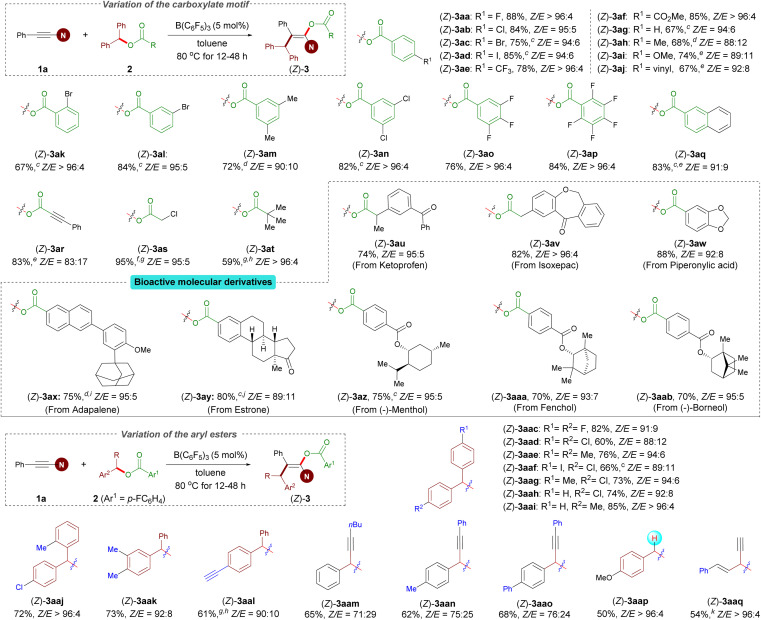
Variation of the esters.^*a*,*b a*^ Conditions A: 1a (0.24 mmol), 2 (0.20 mmol), B(C_6_F_5_)_3_ (5.0 mol%), toluene (2.0 mL), at 80 °C for 12–48 h. ^*b*^ Isolated yield of (*Z*)-3. ^*c*^ 10 mol% B(C_6_F_5_)_3_ used. ^*d*^ Run at 100 °C. ^*e*^ Combined isolated yield of *Z*/*E* mixtures which cannot be separated by chromatography. ^*f*^ Unstable compound. ^*g*^ The yield was estimated by ^1^H NMR spectroscopy. ^*h*^ The desired product containing a trace amount of the hydroacyloxylation side product. ^*i*^ 20 mol% B(C_6_F_5_)_3_ used. ^*j*^ 0.4 mmol 1a used. ^*k*^ Combined isolated yield of the regioisomers which cannot be separated by chromatography.

With optimized reaction conditions established, we examined the reactions of benzhydryl esters 2 containing various carboxylates with 1a. Esters 2a–q derived from aryl carboxylic acids were subjected to Conditions A described in Scheme 2. This protocol is amenable to a variety of esters bearing different R^1^ functional groups, including halogen (2a–d), trifluoromethyl (2e), carboxyl (2f), methoxy (2i), and vinyl (2j) groups in the *para* position of the aromatic ring, and led to the corresponding enol esters in good to excellent yields and diastereocontrol with *Z*/*E* up to >96 : 4. In general, substrates with an electron-withdrawing group showed higher yield and *Z*/*E* ratio than the other substrates bearing an electron-donating group (2e*versus*2h and 2f*versus*2i). As expected, functional groups at the *meta*- and *ortho*-positions gave satisfactory results for 10 mol% catalyst loading (2k and 2l). In addition, disubstituted (2m and 2n) and polysubstituted (2o and 2p) substrates were found to function exceptionally well in this reaction. Of note, benzhydryl propiolate 2r reacted chemoselectively (ynamides over electron-deficient alkynes) in good yield. The present protocol can also be efficiently applied to benzhydryl acetate derivative 2s and pivalic acid ester 2t to afford the corresponding products 3as and 3at in high yields and with excellent *Z*/*E* ratios. Importantly, our protocol allowed the incorporation of the enol ester fragment into bioactive molecules such as ketoprofen, isoxepac, adapalene, estrone, and borneol (2u–ab). Notably, when benzhydryl esters 2z–ab bearing an additional ester functional group were subjected to the standard reaction conditions, only the benzhydryl ester motif was successfully incorporated into the products, affording corresponding products in good yields and *Z*/*E* ratios. We next investigated the scope of the aryl ester. Symmetrical diaryl esters bearing electron-withdrawing (4-F as in 2ac and 4-Cl as in 2ad) and electron-donating (4-Me as in 2ae) groups all worked well for the reactions when coupled with 1a generating 3aac–3aae in good yields; The unsymmetrical diaryl esters containing halogen (2af–ah and 2aj), electron-neutral (H as in 2ai), alkyl in the *ortho*- (2aj) or *meta*-position (2ak), and even alkynyl (2al) groups on the aryl ring were all tolerated and gave the corresponding products (3aaf–aal) in good yields and *Z*/*E* ratios ranging from 89 : 11 to >96 : 4. Rather than diaryl esters, alkynyl(aryl) esters were also competent to afford products 3aam–aao, albeit with a lower yield and stereoselectivity. It is particularly noteworthy that the reaction of 4-methoxybenzyl 4-fluorobenzoate 2ap was successful under Conditions A, yielding 3aap in reasonable yield and with an excellent *Z*/*E* ratio. Besides benzyl ester derivatives, the reaction of alkynyl(alkenyl) ester 2aq with 1a at 80 °C for 12 h resulted in in a mixture of regioisomers in 54% total yield (see the ESI† for details).

**Scheme 3:**
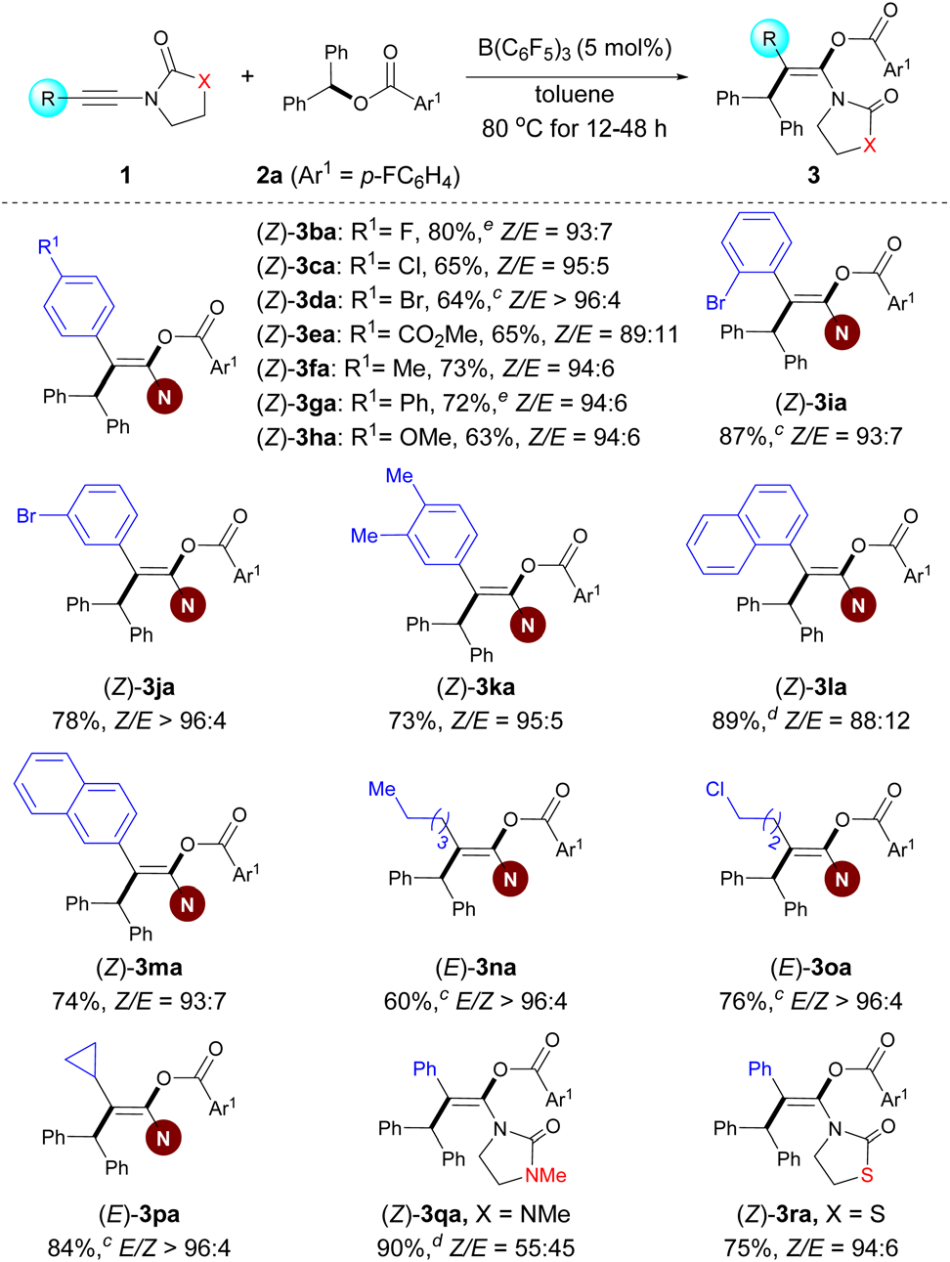
Variation of the ynamides.^*a–c*^ For footnotes *a–c*, see Scheme 2. ^*d*^ Combined isolated yield of *Z*/*E* mixtures which cannot be separated by chromatography. ^*e*^ The desired product containing a trace amount of the hydroacyloxylation side product.

After the investigation of the ester scope, we studied the scope with respect to the ynamides (Scheme 3). Several 2-aryl ynamides 1b–k were tested with the ester 2a. Different substitution patterns of the aromatic substituent were tolerated independent of their electronic nature, providing products 3ba–ka in yields between 63% and 87% and with *Z*/*E* ratios ranging from 89 : 11 to >96 : 4. The naphthyl-containing ynamides 1l and 1m also afforded the corresponding products in good yields. Moreover, ynamides bearing different primary or secondary alkyl groups afforded the desired products with exclusive *trans* selectivity with a higher catalyst loading (1n–p). The amide moiety was not limited to oxazolidin-2-one; ynamides featuring a urea functionality and a thiazolidine-2-one were also competent in this reaction, resulting in the desired products (3qa–ra) in excellent yields, albeit with a low stereoselectivity for product 3qa. Meanwhile, it should be noted that an ynamide containing a sulfonamide group instead of the oxazolidinone group afforded a complex reaction mixture, and attempts to isolate any pure compound failed. It is probably due to the ynamide containing a sulfonamide group, which readily undergoes hydrocarbation as reported by Mayr and co-workers.^36^

### Catalytic synthesis of fully substituted enol carbonates

**Scheme 4:**
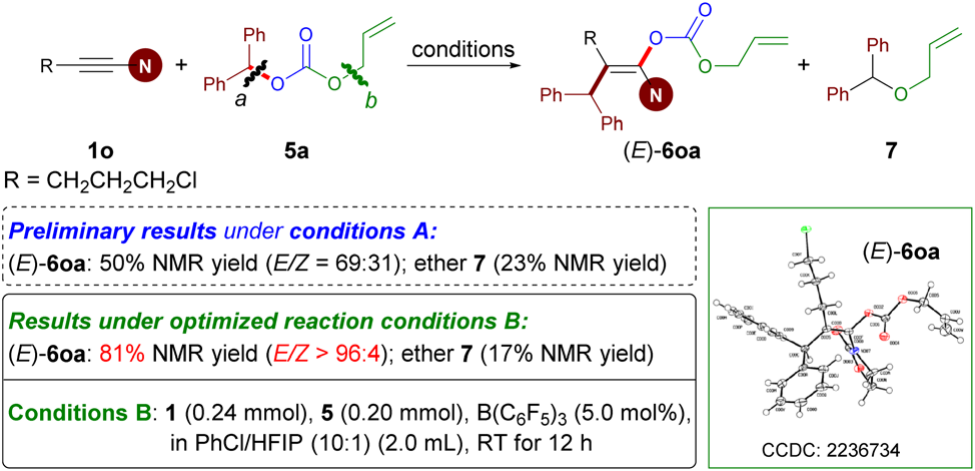
Preliminary trials and conditions B for the synthesis of stereodefined enol carbonates.

Enol carbonate derivatives have served as versatile synthons in organic transformations.^7^ After the implementation of the carboacyloxylation of ynamides for the synthesis of enol esters, we wondered whether we could expand this catalytic methodology to build enol carbonates. To examine this hypothesis, readily available allyl benzhydryl carbonate 5a and ynamide 1o were selected as the model substrates (Scheme 4). In contrast to esters 2, the employment of carbonates 5 as bifunctional reagents in the current reaction poses formidable challenges. On the one hand, the main challenge is the issue of site selectivity because of the presence of two C–O reactive sites (*e.g.* bond *a versus b*). On the other hand, a competitive side reaction encountered in this case is the formation of ethers *via* decarboxylative etherification.

Treatment of 1o and 5a with B(C_6_F_5_)_3_ under Conditions A did afford the desired product 6oa. However, a lower yield (50%) and *E*/*Z* ratio (69 : 31) of 6oa together with side product ether 7 were obtained. After further investigations leading to the reoptimized reaction conditions B, the carboacyloxylation reaction provided the desired (*E*)-6oa in 81% NMR yield with *E*/*Z* > 96 : 4 at room temperature.^35^ Of note, the solvent (PhCl/HFIP) and the reaction temperature played an important role in controlling the stereoselectivity and the inhibition of side reactions (see Table S2 in the ESI†).

**Scheme 5:**
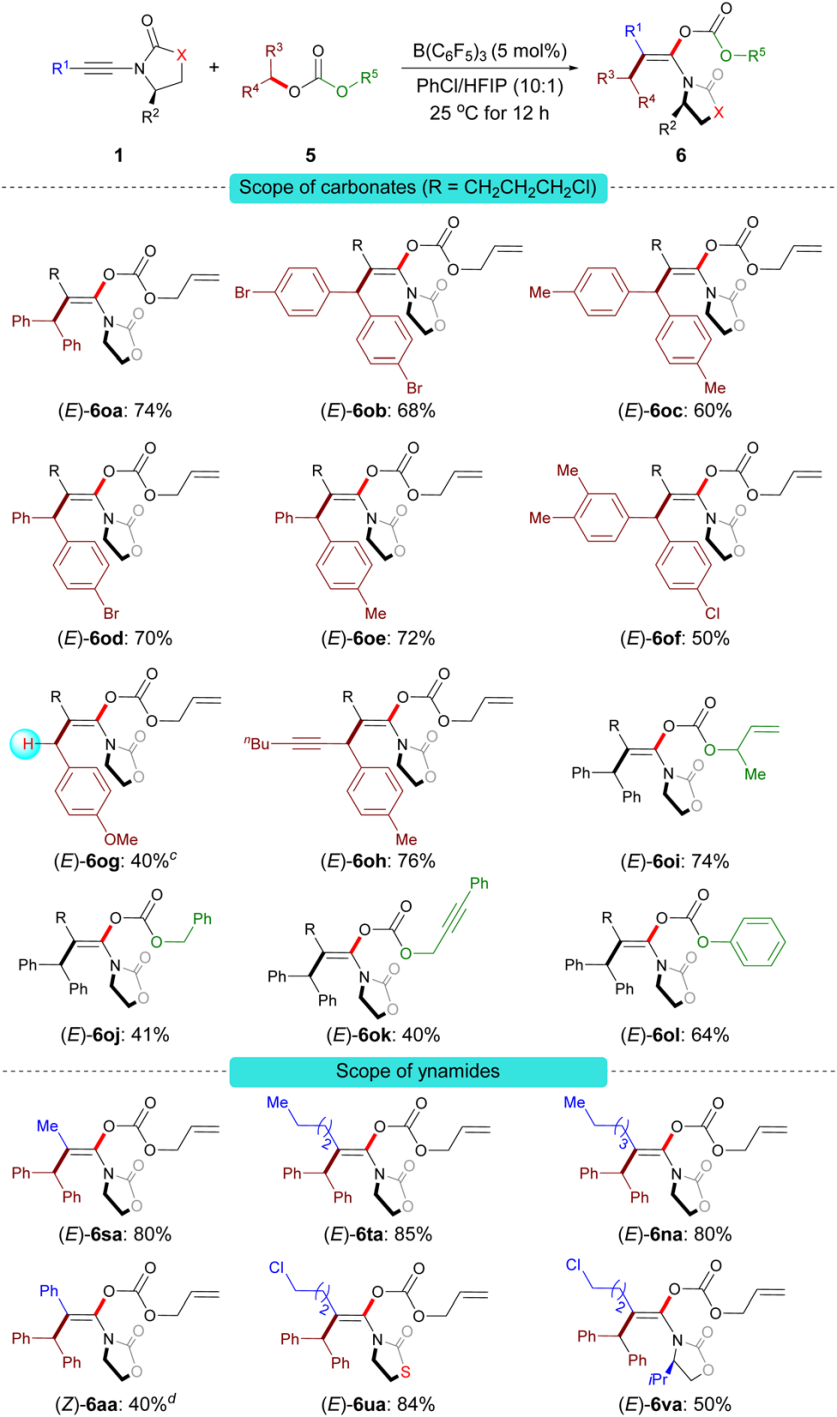
Scope of the carboacyloxylation of ynamides with carbonate derivatives.^*a*,*b a*^ Conditions B. ^*b*^ Isolated yield of 6, *E*/*Z* > 96 : 4. ^*c*^ The product was obtained along with a trace amount of unidentified mixtures; the yield was estimated by ^1^H NMR spectroscopy. ^*d*^*Z*/*E* > 96 : 4.

Under the optimal reaction conditions B, a series of carbonate derivatives 5 were prepared and examined. As shown in Scheme 5, allyl benzhydryl carbonate derivatives 5a–f bearing either electron-withdrawing or electron-donating groups on the aromatic ring were effectively converted into the stereodefined enol carbonates 6oa–of in moderate to good yields. The reaction was not limited to allyl benzhydryl carbonates; carbonates 5g derived from a primary benzylic alcohol and 5h derived from the alkynyl(aryl) alcohol were also tolerated. Furthermore, the substituent R^5^ can be 2-methylallyl (5i), alkyl (5j), propargyl (5k) or phenyl (5l). The reactions proceeded smoothly to give the desired stereodefined enol carbonates in reasonable yields.

Having demonstrated the scope of the carbonates, our attention moved towards exploring the versatility of the ynamides. Various alkyl-substituted ynamides were screened and the desired enol carbonates (6sa–ta and 6na) were obtained in excellent yields. Aryl-substituted ynamide 1a was also compatible, albeit in lower yield. Also, thiazolidine-2-one-derived ynamide 1u and chiral ynamide 1v reacted smoothly to afford the corresponding stereodefined enol carbonates in good yields.

### Synthetic transformations

**Scheme 6:**
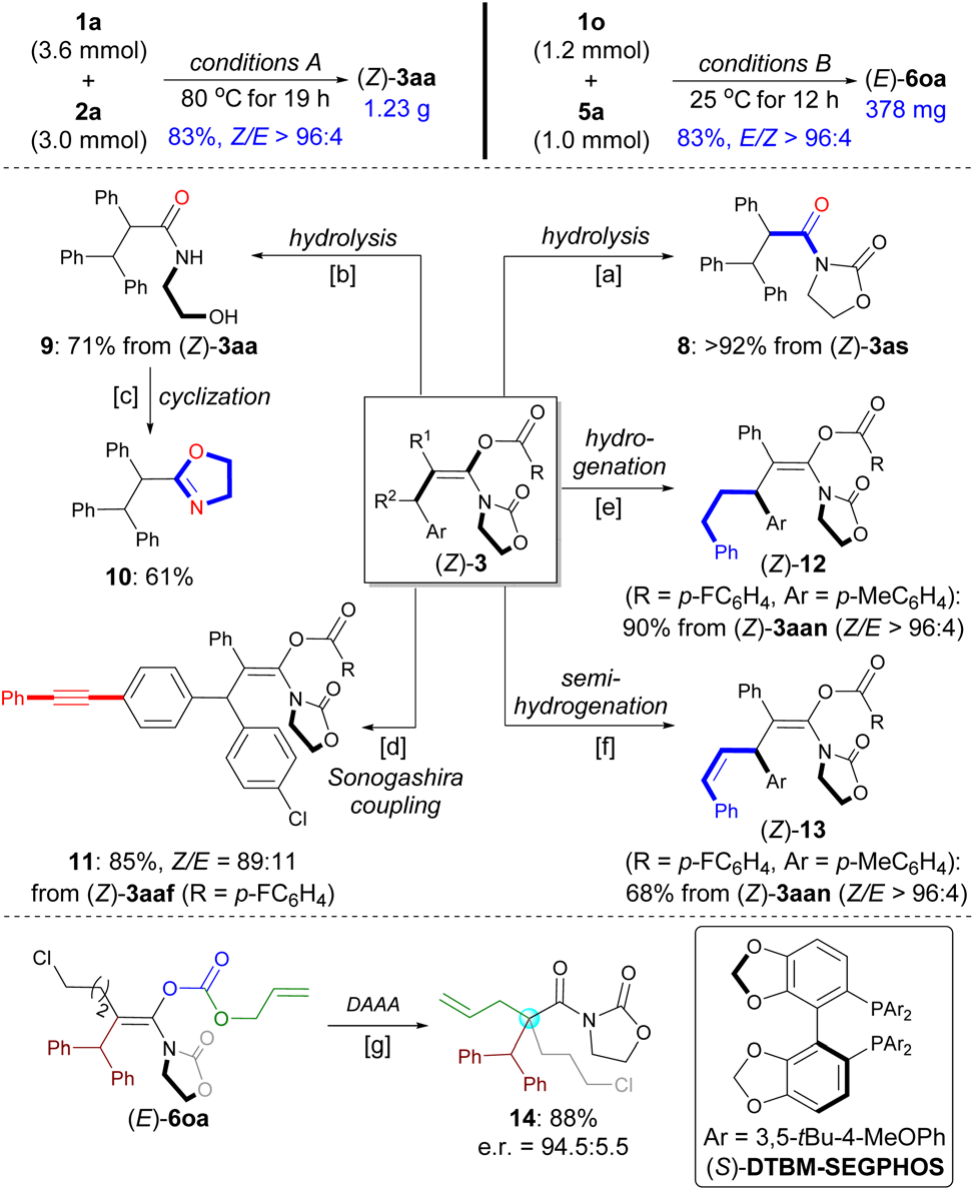
Scope of Gram-scale synthesis and synthetic transformations. ^*a*^ SiO_2_, Et_3_N (2.0 equiv.), toluene, RT. ^*b*^ LiOH (1.0 equiv.), MeOH/H_2_O (3 : 1), 80 °C. ^*c*^ TsCl (1.0 equiv.), CH_2_Cl_2_/Et_3_N (1 : 1), 80 °C. ^*d*^ Pd(PPh_3_)_2_Cl_2_ (5 mol%), CuI (5 mol%), Et_3_N, THF, RT. ^*e*^ Pd/C (10%), H_2_ (1 atm), MeOH, 50 °C. ^*f*^ Pd(OAc)_2_ (5 mol%), P(*o*-Tol)_3_ (10 mol%), B_2_(Pin)_2_ (1.2 equiv.), H_2_O (5 equiv.), toluene, 80 °C. ^*g*^ Pd_2_(dba)_3_ (4 mol%), (*S*)-DTBM-SEGPHOS (16 mol%), THF, −20 °C.

The practicality of this method was demonstrated by performing a gram-scale synthesis of (*Z*)-3aa (1.23 g) and a scale-up synthesis of (*E*)-6oa (1.0 mmol) with maintaining selectivity and yield (Scheme 6, top). 3as was found to be unstable and was expected to hydrolyze on silica gel during purification by flash column chromatography. Thus, amide 8 was isolated in 92% yield after subjecting crude 3as to the silica gel and Et_3_N (Scheme 6a). By contrast, both the enol ester group and oxazolidinone were hydrolyzed to give *N*-acyl ethanolamine 9 in 71% yield in the presence of LiOH, which can be readily converted into oxazole 10 (Schemes 6b and c). The functionalized enol ester 11 can be prepared from 3aaf by chemoselective Sonogashira coupling (Scheme 6d).

The C–C triple bond in (*Z*)-3aan was hydrogenated over Pd/C to produce 12 in 90% yield (Scheme 6e). Selective semi-reduction of the alkyne for obtaining *Z*-alkene in 13 was achieved by using Prabhu's protocol (Scheme 6f).^37^ These two transformations as alternative strategies have, to a certain extent, served the purposes of the synthesis of corresponding enol esters from alkyl aryl esters or alkenyl aryl esters with ynamides.

Palladium-catalyzed decarboxylative asymmetric allylic alkylation (DAAA) of fully substituted enol carbonates represents a practical strategy to set a quaternary carbon stereocenter in an acyclic system.^7^ In 2017, utilizing chemistry developed by the Marek group for the synthesis of acyclic enolates,^24*a*^ the Stoltz group disclosed the elegant DAAA reactions of acyclic amide enolates utilizing Trost's ligand (Scheme 1C).^24*f*^ The use of acyclic carbamate substrates proved to be crucial to afford products in high enantioselectivities. In contrast, the DAAA of oxazolidinone-based allyl enol carbonates only gave the desired product with up to e.r*.* = 88 : 12. With a library of stereodefined oxazolidinone-based allyl enol carbonates 6 in hand, we decided to evaluate the palladium-catalyzed DAAA of (*E*)-6oa to afford α-quaternary amide 14 that is not reported for Stoltz's system. Gratifyingly, with the use of Pd_2_(dba)_3_/(*S*)-DTBM-SEGPHOS as the catalyst, the reaction provided the desired product 14 in 88% yield with e.r. = 94.5 : 5.5 e.r. (Scheme 6g; see also Table S3 in the ESI†).

### Mechanistic studies

**Scheme 7:**
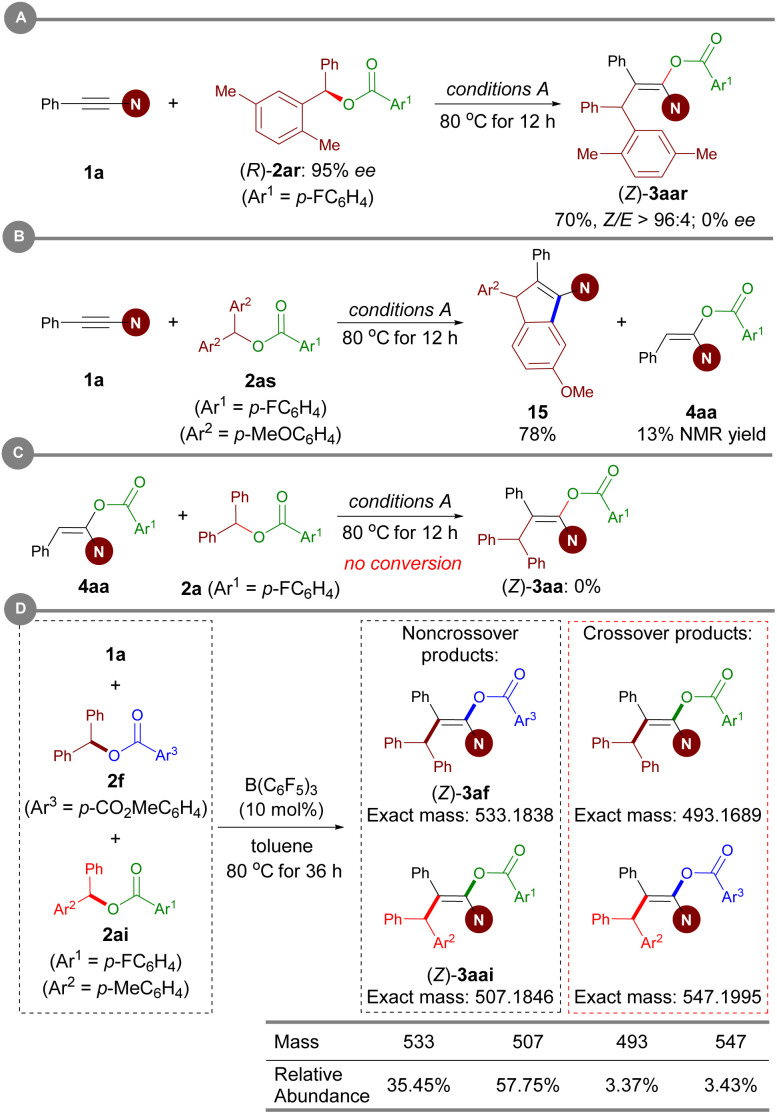
Mechanistic experiments.

To gain insight into the reaction mechanism, several control experiments were conducted. When an enantiomerically pure sample of ester (*R*)-2ar was subjected to Conditions A, the resulting enol ester was obtained in racemic form, thus revealing that carboacyloxylation proceeds through a carbocation intermediate (Scheme 7A). Subsequently, electrophilic addition of the *in situ*-generated carbocation to ynamide 1 could give a keteniminium ion as an intermediate. To verify this proposal, ester 2as bearing electron-rich aromatic moieties was prepared, and then treated with 1a under Conditions A. Interestingly, the reaction afforded 1-amidoindene 15 in 78% NMR yield along with 13% NMR yield of 4aa (Scheme 7B).^19*m*^ Given that 4aa was observed in current carboacyloxylations, we subjected 4aa and ester 2a to Conditions A to test whether the carboacyloxylation product arose from downstream benzylation of the hydroacyloxylation product, but 3aa was not formed (Scheme 7C). Furthermore, we performed crossover experiments with a mixture of equimolar amounts of ynamide 1a, and esters 2f and 2ai under conditions A (Scheme 7D). Analysis of the products by liquid chromatography-mass spectrometry revealed the presence of all four possible product masses, indicating that the addition of the carboxylate and the benzyl group to the ynamide is proceeding in a stepwise manner, and the resonance-stabilized anion may facilitate crossover by dissociation from the carbocation center.

To further elucidate the mechanistic details of this reaction and to explain the observed stereoselectivity, DFT calculations on a model reaction of ynamide 1a with diaryl ester 2a using the B(C_6_F_5_)_3_ catalyst were conducted at the M06-2X/cc-pVTZ//M06-2X/6-31G(d,p) level^38^ using the Gaussian 16 program.^39^ The solvent effect for toluene was taken into consideration using the polarizable continuum model.^40^ 3D structures were generated with CYLview.^41^ According to our calculations and the aforementioned control experiments (see Table 1, entries 10 and 11), the reaction proceeds through an ionic mechanism rather than a radical pathway due to the high energies required for the related single electron transfer (SET) process^27*a*,*d*^ (see Fig. S1 in the ESI†). Therefore, the following discussion will be focused on the possible two-electron processes involved in this reaction.

**Fig. 1:**
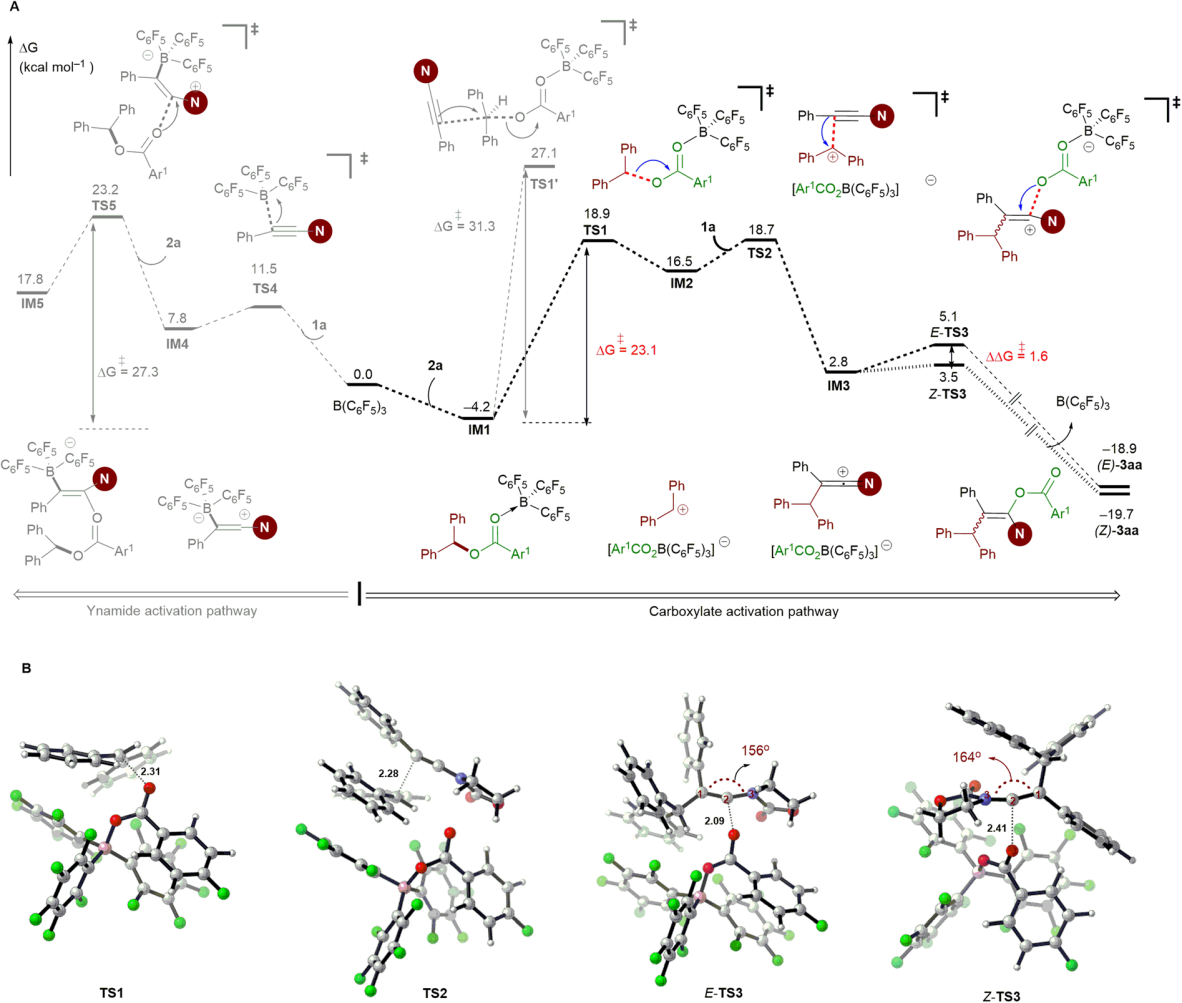
DFT calculations for the reaction mechanism. A: Reaction coordinate for the B(C_6_F_5_)_3_-catalyzed carboacyloxylation of ynamide 1a with diaryl ester 2a. Computed at the PCM (toluene)/M062X/cc-PVTZ//PCM (toluene)/M062X/6-31G(d,p) level (Gibbs free energies are in kcal mol^−1^, Ar^1^ = *p*-FC_6_H_4_). B: Optimized transition state structures involved in the carboxylate activation pathway (distances are shown in Å). Color code: H, white; B, pink; C, gray; N, blue; O, red; N, blue.

As shown in Fig. 1A, the carboacyloxylation reaction starts with the complexation of 2a with B(C_6_F_5_)_3_ to give the Lewis adduct IM1. The formation of IM1 is exergonic by 4.2 kcal mol^−1^. Although it is thermodynamically less favorable compared to 1a·B(C_6_F_5_)_3_ carbonyl adduct IM1′ (Δ*G* = −6.4 kcal mol^−1^), our computational results show that the ynamide activation pathway through the complexation of B(C_6_F_5_)_3_ with the oxygen atom of 1a (IM1′) can be excluded (see Fig. S2, Table S1† and related discussions for details). IM1 undergoes C–O cleavage to form ion pair IM2 with a barrier of 23.1 kcal mol^−1^ (*via*TS1). IM2 consists of an electrophilic carbenium ion and a borate anion [Ar^1^CO_2_B(C_6_F_5_)_3_]^–^ as the counteranion, which readily undergo an electrophilic addition reaction with ynamide 1a to afford the new ion pair IM3 (*via*TS2). This step has a barrier of 22.9 kcal mol^−1^, and the formation of IM3 is endergonic by 2.8 kcal mol^−1^ relative to 2a and B(C_6_F_5_)_3_. The borate anion [Ar^1^CO_2_B(C_6_F_5_)_3_]^–^ of IM3 can attack at the carbocationic center of IM3 from the same and opposite side of the 1,1-diarylmethyl group to give the *syn*- (*via E*-TS3) and *anti*-addition (*via Z*-TS3) products, respectively (Fig. 1B). The formation of *E*-3aa requires a higher barrier than that of *Z*-3aa (3.5 *versus* 5.1 kcal mol^−1^), and *Z*-3aa is thermodynamically more favorable than the *syn*-addition product by 0.8 kcal mol^−1^. In *E*-TS3, the ketene iminium fragment is in a more distorted conformation than it is in the favored transition state *Z*-TS3 (C1–C2–N3 bond angle: 156° *versus* 164°). This might be attributed to the steric hindrance imparted by the 1,1-diarylmethyl moiety. The calculated free energy difference ΔΔ*G*^‡^ = 1.6 kcal mol^−1^ is in good agreement with the experimentally observed stereoselectivity [(*Z*)-3aa/(*E*)-3aa > 96 : 4].

Alternatively, IM1 could also react with ynamide *via* a concerted S_N_2 transition state to afford the ion pair IM3 (*via*TS1′). Our computations exclude this pathway due to the involvement of a high-energy transition state. This result is supported by the control experiment shown in Scheme 7A. Besides, a pathway proceeding through borane activation of the alkyne for the intramolecular carboacyloxylation of alkynyl carboxylic esters was proposed by Melen and co-workers.^22^ Although the addition of B(C_6_F_5_)_3_ to ynamide is kinetically feasible (Δ*G*^‡^ = 15.7 kcal mol^−1^), the subsequent nucleophilic attack of diaryl ester 2a to the 1a·B(C_6_F_5_)_3_ adduct IM4 is kinetically less favored than the above pathway by 4.3 kcal mol^−1^. Therefore, a pathway involving the activation of the carboxylate of the diaryl ester by B(C_6_F_5_)_3_ by an S_N_1-type mechanism is likely responsible for this carboacyloxylation process.

## Conclusion

In summary, by taking advantage of esters as bifunctional reagents in the metal-free carboacyloxylation reaction of ynamides, an atom-economic and highly selective method for the synthesis of fully substituted acyclic enol esters was developed. To the best of our knowledge, this is the first B(C_6_F_5_)_3_-catalyzed intermolecular 1,2-difunctionalization reaction of internal alkynes for the synthesis of acyclic tetrasubstituted alkenes. The salient features of this transformation include readily available starting materials, broad substrate scope, and scalability. The applicability was further illustrated in the late-stage modification of natural products and drug-like molecules. Notably, the protocol is also amenable to the synthesis of stereodefined acyclic β,β-disubstituted enol carbonates of amides, especially amide enol allyl carbonates, in one step. They are difficult to synthesize using transition-metal-catalyzed methods and can only be prepared by non-catalytic processes.^24*a*,*f*^ Furthermore, we applied a palladium-catalyzed decarboxylative asymmetric allylic alkylation to the amide enol allyl carbonate to generate an *α*-quaternary amide in high yield and enantioselectivity (up to 88% yield and e.r. = 94.5 : 5.5). Control experiments combined with DFT studies support an S_N_1 pathway and rule out a concerted S_N_2 mechanism as well as a pathway involving the activation of the alkyne by B(C_6_F_5_)_3_.

## Data availability

The datasets supporting this article have been uploaded as part of the ESI.†

## Author contributions

Y. X., L. T., T.-T. X., and J.-Y.-H. S. performed the experiments and conducted the analytical characterization. Z. Z. and L. Y. conducted the crossover experiments described in Scheme 7D. G. W. executed the theoretical calculations. G. W., M. O. and J.-J. F. wrote the manuscript. J.-J. F. conceived the catalytic system.

## Conflicts of interest

There are no conflicts to declare.

## Supplementary Material

SC-014-D3SC01394D-s001

SC-014-D3SC01394D-s002
