# Supplementary material for: Atom-economic and stereoselective catalytic synthesis of fully substituted enol esters/carbonates of amides in acyclic systems enabled by boron Lewis acid catalysis
Source: Chem Sci. 2023 Apr 26;14(21):5608–18. doi: 10.1039/d3sc01394d (PMC10231430; doi:10.1039/d3sc01394d)
Supplement: SC-014-D3SC01394D-s001 [file SC-014-D3SC01394D-s001.pdf]

Electronic Supplementary Material (ESI) for Chemical Science.
This journal is © The Royal Society of Chemistry 2023



# Atom-economic and stereoselective catalytic synthesis of fully substituted enol esters/carbonates of amides in acyclic systems enabled by boron Lewis acid catalysis

Yuanjiu Xiao, Lei Tang, Tong-Tong Xu, Jiang-Yi-Hui Sheng, Zhongyan Zhou, Lei Yue, Guoqiang Wang,* Martin Oestreich* and Jian-Jun Feng*

## Electronic Supplementary Information

### Table of Contents

1 General Information ........................................................................ S3

2 Table S1. Optimization of the Alkyl Acyloxylations of Ynamides.............. S4

3 Table S2. Optimization of the Alkyl Carboacyloxylation of Ynamides ....... S5

4 Table S3. Optimization of the Decarboxylative Allylation Reaction ........... S6

5 Unsuccessful Substrates & More Details About the Scope the Reactions S6

6 General Procedure for the Alkyl Acyloxylations of Ynamides (GP1)......... S8

7 General Procedure for the Alkyl Carboacyloxylation of Ynamides (GP2).. S8

8 Procedure for the Decarboxylative Allylic Alkylation ................................ S8

9 General Procedure for the Synthesis of Esters **2u-2ab** (GP3)................ S10

10 Scale-Up Experiment ............................................................................ S16

11 Synthetic Transformations.................................................................... S17

12 Mechanistic Control Experiments ......................................................... S20

13 Characterization Data of the Products.................................................. S22

14 Crystal Structure of (*Z*)-**3aa** and (*E*)-**6oa** ............................................. S89

15 NMR Spectra ......................................................................................... S91

16 Computational Data ............................................................................. S210

16.1 Results of the DFT Calculations ......................................................... S210

16.2 Cartesian Coordinates of the Optimized Stationary Point ..................... S213

17 References........................................................................................... S237

# 1 General Information

All reactions were performed in flame-dried glassware using conventional Schlenk techniques under a static pressure of nitrogen unless otherwise stated. Liquids and solutions were transferred with syringes. Ynamides[1], esters[2] and carbonates[3] were prepared according to reported procedures. Tris(pentafluorophenyl)borane ($B(C_5F_5)_3$, 98%, *Energy Chemical*) was purchased from commercial suppliers and used as received. Other commercially available reagents were purchased from *Sigma-Adrich*, *Leyan* and *Bide* Chemical Company. Chlorobenzene (PhCl) and Benzotrifluoride ($PhCF_3$) were purchased from *Energy Chemical* (99%, Extra Dry) and used as received. All other solvents ($CH_2Cl_2$, toluene, and 1,2-dichloroethane *etc*.) were dried and purified following standard procedures. Technical grade solvents for extraction or chromatography (Petroleum ether, $CH_2Cl_2$, and ethyl acetate) were distilled prior to use. Analytical thin layer chromatography (TLC) was performed on silica gel 60 F254 glass plates by *Merck*. Flash column chromatography was performed on silica gel 60 (40–63 µm, 230–400 mesh, ASTM) by *Grace* using the indicated solvents. $^1$H, $^{13}$C NMR spectra were recorded in $CDCl_3$ on Bruker AV400 instruments. Chemical shifts are reported in parts per million (ppm) and are referenced to the residual solvent resonance as the internal standard ($CHCl_3$: δ = 7.26 ppm for $^1$H NMR and $CDCl_3$: δ = 77.0 ppm for $^{13}$C NMR). Data are reported as follows: chemical shift, multiplicity (s = singlet, d = doublet, t = triplett, q = quartet, m = multiplet), coupling constants (Hz), and integration. The full-scan mass spectra were taken on a hybrid quadrupole-orbitrap mass spectrometer equipped with a heated electrospray ionization source (ThermoFischer Scientific, Bremen, Germany). X-ray data were taken on a Bruker SMART APEX II X-Ray diffractometer equipped with a large area CCD detector.

## 2 Table S1. Optimization of the alkyl acyloxylations of ynamides

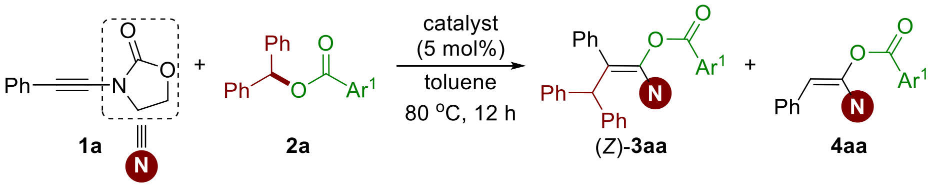


| entry | catalyst | solvent | conv. (%)[b] | yield of (*Z*)-**3aa** (%)[b] | yield of **4aa** (%)[b] | *Z/E* [b] |
|---|---|---|---|---|---|---|
| **1** | **$B(C_6F_5)_3$** | **toluene** | **100** | **95(88)** | **5** | **>96:4** |
| 2[c] | $B(C_6F_5)_3$ | toluene | 100 | 95 | 5 | >96:4 |
| 3[d] | $B(C_6F_5)_3$ | toluene | 94 | 88 | 5 | 95:5 |
| 4 | $Cu(OTf)_2$ | toluene | 0 | 0 | 0 | – |
| 5 | $Sc(OTf)_3$ | toluene | 25 | 3 | 10 | 75:25 |
| 6 | $Zn(OTf)_2$ | toluene | 0 | 0 | 0 | – |
| 7 | $ZnCl_2$ | toluene | 0 | 0 | 0 | – |
| 8 | TfOH | toluene | 100 | 0 | 15 | – |
| 9 | $BF_3{\cdot}Et_2O$ | toluene | 20 | 12 | 8 | 79:21 |
| 10 | $BCl_3$ | toluene | 0 | 0 | 0 | – |
| 11[e] | $B(C_6F_5)_3$ | toluene | 100 | 75 | 17 | 92:8 |
| 12[f] | $B(C_6F_5)_3$ | toluene | 100 | 69 | 20 | 92:8 |
| 13[g] | $B(C_6F_5)_3$ | toluene | 13 | 9 | 3 | 90:10 |
| 14 | $B(C_6F_5)_3$ | $PhCF_3$ | 100 | 66 | 25 | >96:4 |
| 15 | $B(C_6F_5)_3$ | PhCl | 100 | 73 | 23 | >96:4 |
| 16 | $B(C_6F_5)_3$ | MeCN | 0 | 0 | 0 | – |
| 17 | $B(C_6F_5)_3$ | DMF | 0 | 0 | 0 | – |
| 18 | $B(C_6F_5)_3$ | DCE | 75 | 26 | 38 | 90:10 |
| 19 | $B(C_6F_5)_3$ | Hexane | 40 | 8 | 21 | 80:20 |
| 20 | $B(C_6F_5)_3$ | $MeNO_2$ | 5 | 0 | 5 | – |
| 21 | $B(C_6F_5)_3$ | HFIP | 100 | 4 | 27 | >96:4 |
| 22[h] | $B(C_6F_5)_3$ | toluene | 80 | 64 | 8 | 93:7 |

[a]Reaction conditions: **1a** (0.24 mmol), **2a** (0.20 mmol), catalyst (5.0 mol %), solvent (2 mL), 80 °C, under $N_2$ for 12 h. Abbreviations: Tf = trifluoromethanesulfonyl; DMF = *N*, *N*-Dimethylformamide; DCE = 1,2-dichloroethane. [b]The yields of (*Z*)-**3aa** and *Z/E* ratio were determined by $^1H$ NMR spectroscopy using $CH_2Br_2$ as the internal standard. The number in the parentheses is the isolated yield of (*Z*)-**3aa**. [c]**1a** (0.30 mmol), **2a** (0.20 mmol). [d]**1a** (0.20 mmol), **2a** (0.24 mmol). [e]The reaction was run at 90 °C. [f]The reaction was run at 100 °C. [g]The reaction was run at 60 °C. [h]2.5 mol % catalyst.

# 3 Table S2. Optimization of the Alkyl Carboacyloxylation of Ynamides

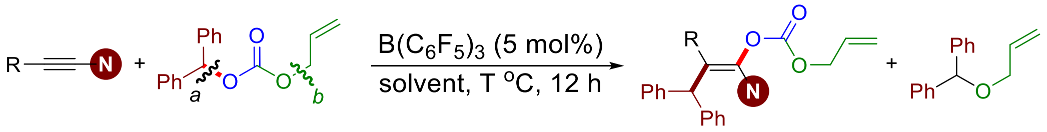


| entry | solvent | T (℃) | conv. (%)[b] | yield of (*E*)-**6oa** (%)[b] | yield of **7** (%)[b] | *E/Z*[b] |
|---|---|---|---|---|---|---|
| 1 | toluene | 100 | 100 | 49 | 15 | 60:40 |
| 2 | toluene | 80 | 100 | 50 | 23 | 69:31 |
| 3[c] | toluene | 80 | 100 | 50 | 25 | 67:33 |
| 4 | toluene | 60 | 100 | 80 | 5 | 84:16 |
| 5 | toluene | 40 | 97 | 80 | 3 | 84:16 |
| 6 | toluene | 25 | 97 | 76 | 5 | 83:17 |
| 7 | PhCl | 80 | 100 | 72 | 1 | 73:27 |
| 8 | PhCl | 60 | 100 | 82 | 3 | 85:15 |
| 9 | PhCl | 40 | 100 | 84 | 2 | 86:14 |
| 10 | PhCl | 25 | 100 | 90 | 0 | 90:10 |
| 11 | PhCl | 10 | 98 | 80 | 2 | 83:7 |
| 12 | DCE | 25 | 100 | 60 | 35 | 92:8 |
| 13 | DCM | 25 | 100 | 76 | 17 | 92:8 |
| 14 | HFIP | 25 | 100 | 45 | 42 | >96:4 |
| 15 | $^i$PrOH | 25 | 93 | 0 | 18 | - |
| 16 | $^t$BuOH | 25 | 74 | 10 | 24 | 78:22 |
| 17 | PhCl/HFIP(1:1) | 25 | 100 | 48 | 13 | >96:4 |
| 18 | PhCl/HFIP(1:4) | 25 | 100 | 55 | 10 | >96:4 |
| 19 | PhCl/HFIP(4:1) | 25 | 100 | 70 | 19 | >96:4 |
| 20 | PhCl/HFIP(6:1) | 25 | 100 | 78 | 21 | >96:4 |
| **21** | **PhCl/HFIP(10:1)** | **25** | **100** | **80(74)** | **17** | **>96:4** |
| 22 | PhCl/HFIP(15:1) | 25 | 100 | 77 | 17 | 93:7 |
| 23 | PhCl/HFIP(20:1) | 25 | 100 | 80 | 13 | 92:8 |
| 24[d] | PhCl/HFIP(10:1) | 25 | 100 | 62 | 59 | >96:4 |
| 25[e] | PhCl/HFIP(10:1) | 25 | 100 | 70 | 60 | >96:4 |
| 26[f] | PhCl/HFIP(10:1) | 25 | 100 | 75 | 23 | >96:4 |
| 27[g] | PhCl/HFIP(10:1) | 25 | 100 | 81 | 16 | >96:4 |

[a]Reaction conditions: **1o** (0.24 mmol), **5a** (0.20 mmol), catalyst (5.0 mol %), solvent (2 mL), 80 ℃, under $N_2$ for 12 h. Abbreviations: DCE = 1,2-dichloroethane. [b]The yields of (*E*)-**6oa** and *E/Z* ratio were determined by $^1$H NMR spectroscopy using $CH_2Br_2$ as the internal standard. The number in the parentheses is the isolated yield of (*E*)-**6oa**. [c]10 mol% catalyst. [d]**1o** (0.20 mmol), **5a** (0.30 mmol). [e]**1o** (0.20 mmol), **5a** (0.40 mmol). [f]**1o** (0.3 mmol), **5a** (0.2 mmol). [g]**1o** (0.4 mmol), **5a** (0.2 mmol).

## 4 Table S3. Optimization of the Decarboxylative Allylation Reaction

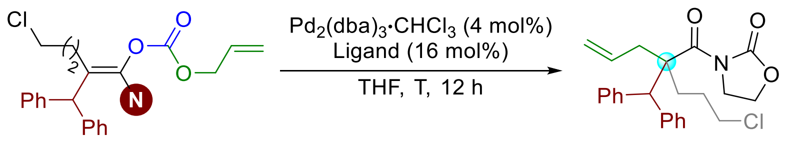


(*E*)-**6oa** **14**

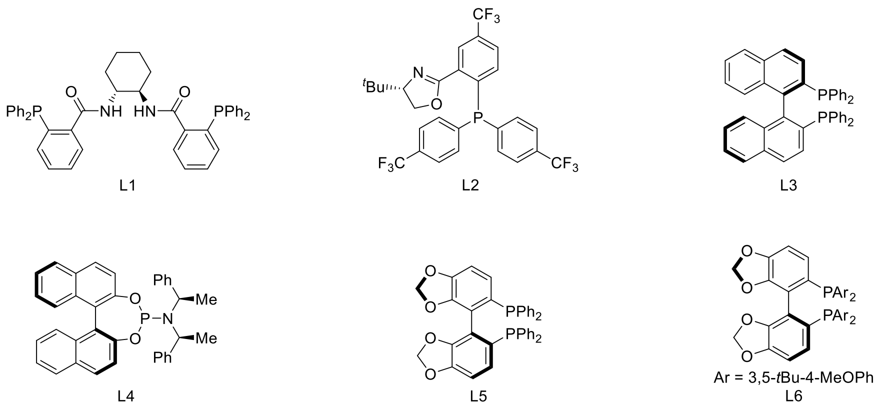


| entry | Ligand | T (℃) | conv. (%)$^b$ | yield of 14 (%)$^b$ | *er*$^c$ |
|---|---|---|---|---|---|
| 1 | L1 | rt | 100 | 55 | 59.5:40.5 |
| 2 | L2 | rt | 100 | 93 | 55:45 |
| 3$^c$ | L3 | rt | 100 | 100 | 78:22 |
| 4 | L4 | rt | 100 | 100 | 75.5:24.5 |
| 5 | L5 | rt | 75 | 74 | 81:19 |
| 6 | L6 | rt | 100 | 100 | 87:13 |
| 7 | L6 | 0 | 100 | 100 | 92:8 |
| **8** | **L6** | **-20** | **97** | **95** | **94.5:5.5** |

$^a$Reaction conditions: (*E*)-6**oa** (0.24 mmol), $Pa_2(dba)_3 \cdot CHCl_3$ (4.0 mol %), Ligand (16 mol%), THF (2 mL),under $N_2$ for 12 h. $^b$The yields of **14** was determined by $^1$H NMR spectroscopy using Mesitylene as the internal standard. $^c$Enantiomeric excess was determined by HPLC.

## 5 Unsuccessful Substrates & More Details About the Scope the Reactions

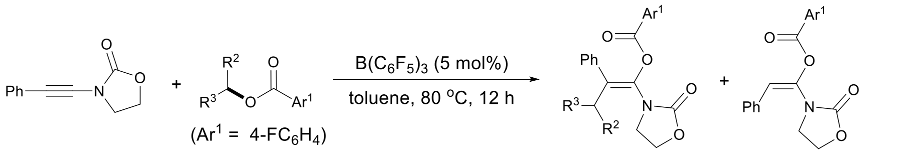


**1a** 0.24 mmol; **2** 0.2 mmol; **3**; **4aa**

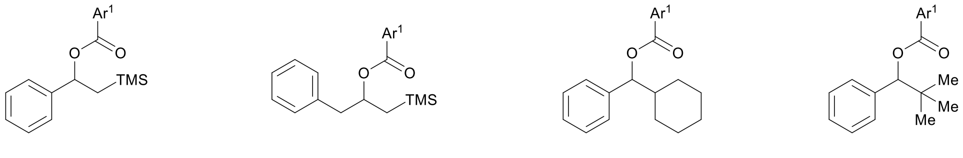

**2at**
100% conversion of **2at**
**3aat**, 0% NMR yield
**4aa,** 27% NMR yield

**2au**
100% conversion of **2au**
**3aau**, 0% NMR yield
**4aa,** 25% NMR yield

**2av**
10% conversion of **2av**
**3aav**, 0% NMR yield
**4aa,** 10% NMR yield

**2aw**
0% conversion of **2aw**

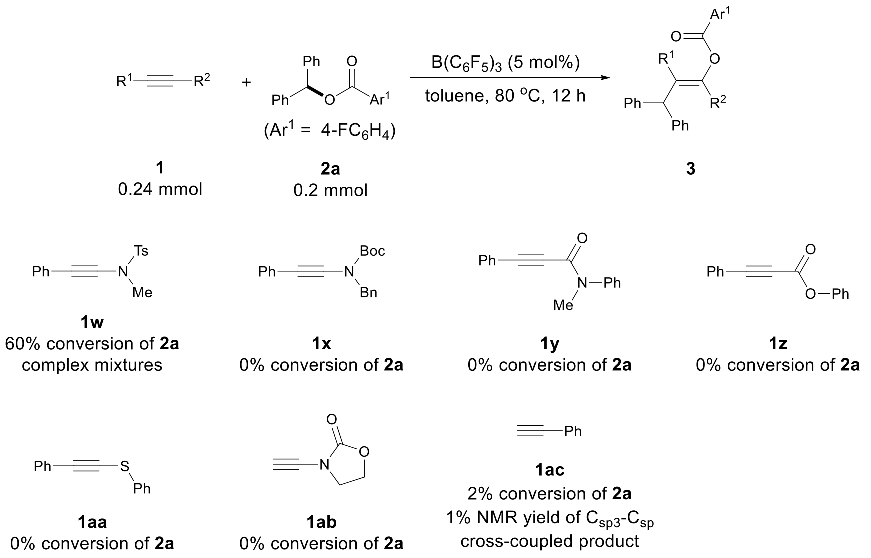


The reactions of other alkynes (**1w-1ac**) with esters **2a** have been examined. When these substrates were tested under the standard alkyl acyloxylations reaction condition (GP1), the ynamide **1w** with **2a** gave complex mixtures. Other alkynes (**1x-1ab**) were all gave results with 0% conversion of **2a**. Subjecting ethynylbenzene (**1ac**) and **2a** to the standard conditions yield a $Csp^3$-Csp cross-coupled product as reported by Prof. Melen (*J. Am. Chem. Soc.* 2021, **143**, 4451-4464). However, the carboacyloxylation of ynol ether **1ad** with ester **2a** gave the desired product (*Z/E*)-**16** in 42% NMR yield.

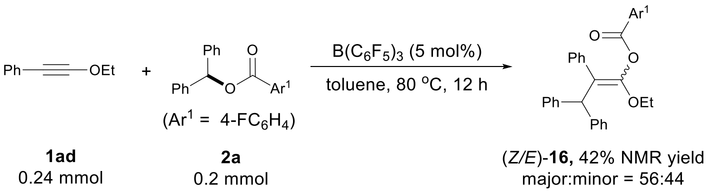


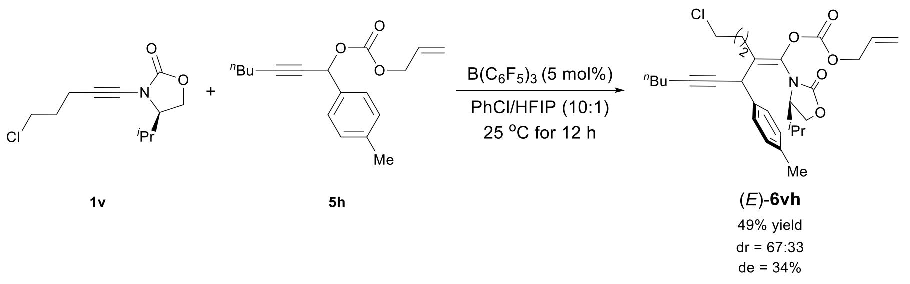

# 6 General Procedure for the Alkyl Acyloxylations of Ynamides (GP1)

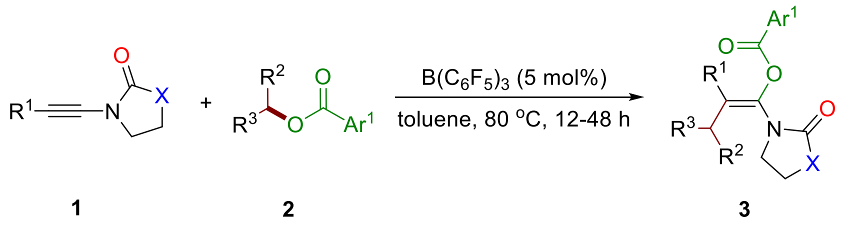

In glove box, to a flame-dried Schlenk tube is charged with the ynamides **1** (0.24 mmol, 1.2 equiv), esters **2** (0.2 mmol), and $B(C_6F_5)_3$ (5.1 mg, 10 μmol, 5 mol%). The tube is evacuated and backfilled with $N_2$ (3 times) followed by the addition of the toluene (2 mL). The reaction mixture is stirred at 80 ℃ for 12-48 h. Evaporation of the solvents under reduced pressure afforded the crude title compound. Purification by flash column chromatography using the indicated mixtures of solvents as eluent yields the analytical pure fully substituted acyclic enol esters **3**.

# 7 General Procedure for the Alkyl Carboacyloxylation of Ynamides (GP2)

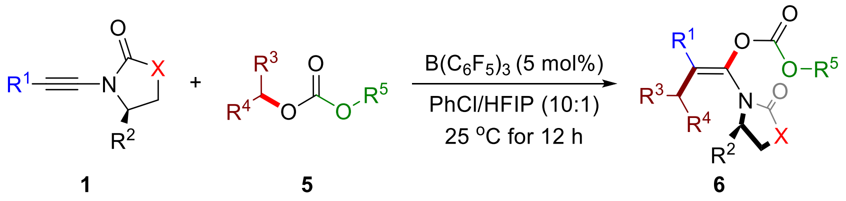

In glove box, to a flame-dried Schlenk tube is charged with the ynamides **1** (0.24 mmol, 1.2 equiv), $B(C_6F_5)_3$ (5.1 mg, 10 μmol, 5 mol%). The tube is evacuated and backfilled with $N_2$ (3 times) followed by the addition of the PhCl/HFIP (1 mL), then the carbonates were dissolved in PhCl/HFIP (1 mL) and added to the reaction tube at ambient temperature. The reaction mixture is stirred at 25 ℃ for 12 h. Evaporation of the solvents under reduced pressure afforded the crude title compound. Purification by flash column chromatography using the indicated mixtures of solvents as eluent yields the analytical pure carboacyloxylation products **6**.

## 8 Procedure for the Decarboxylative Allylic Alkylation

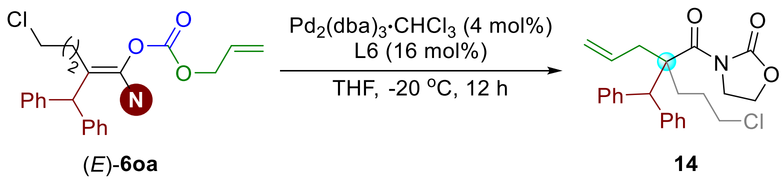


To a solution of $Pd_2(dba)_3{\cdot}CHCl_3$ (8.2 mg, 8 μmol, 4 mol%) in THF (1 mL) at room temperature was added **L6** ligand (36.6 mg, 32 μmol, 16 mol%) and the mixture was stirred for 1 h. This solution was then cooled to -20 ℃ and transferred via cannula to a flask containing of (*E*)-**6oa** (91.2 mg, 0.2 mmol, 1.0 equiv) in THF (1 mL). The reaction mixture was then stirred at the same temperature for 12 h. A saturated aqueous solution of brine was then added and the aqueous phase was extracted with EtOAc (3 x 2 mL). The combined organic phases were washed with brine, dried over anhydrous $MgSO_4$, filtered and evaporated under reduced pressure to afford a crude residue, which was purified by flash chromatography on silica gel using petroleum ether/EtOAc (10/1) afforded **14** as a white solid (72.5 mg, 88% yield).

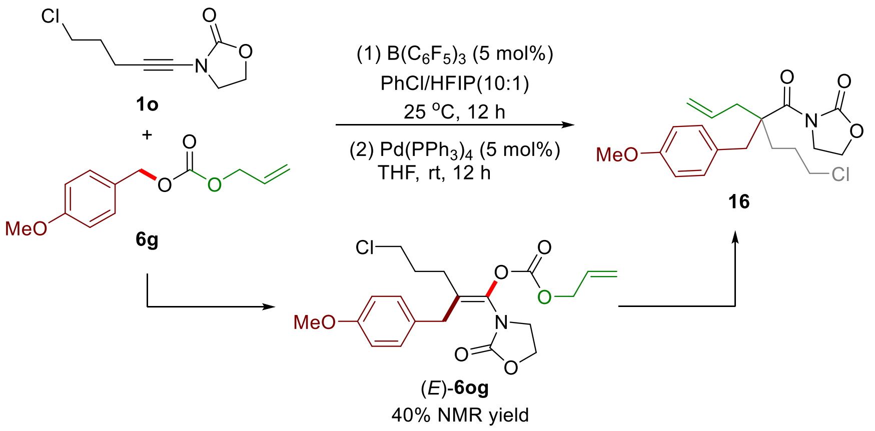


**Synthesis of (16):** In glove box, to a flame-dried Schlenk tube is charged with the 3-(5-chloropent-1-yn-1-yl)oxazolidin-2-one **1o** (45.0 mg, 0.24 mmol, 1.2 equiv), $B(C_6F_5)_3$ (5.1 mg, 5 μmol, 5 mol%). The tube is evacuated and backfilled with $N_2$ (3 times) followed by the addition of the PhCl/HFIP (1 mL), then, the allyl (4-methoxybenzyl) carbonate (44.4 mg, 0.2 mmol, 1.0 equiv) was dissolved in PhCl/HFIP (1 mL) and added to the reaction tube at ambient temperature The reaction mixture is stirred at 25 ℃ for 12 h. Evaporation of the solvents under

reduced pressure and filtered through a short silica gel column using petroleum ether/ EtOAc (5/1) afforded crude mixture (*E*)-**6og** which was used in decarboxylative allylic alkylation (**Note:** Product (*E*)-**6og** was obtained along with a trace amount of unidentified mixtures; 40% NMR yield of (*E*)-**6og** was estimated by $^1$HNMR spectroscopy).

To a flame-dried Schlenk tube is charged with the crude mixture (*E*)-**6og**, $Pd(PPh_3)_4$ (11.5 mg, 10 μmol, 5 mol%). The tube is evacuated and backfilled with $N_2$ (3 times) followed by the addition of the THF (2 mL), then the reaction mixture is stirred at 25 ℃ for 12 h. After removal of the solvent, the residue was purification by flash chromatography on silica gel using petroleum ether/ EtOAc (10/1) afforded **16** as a colorless oil (23.4 mg, 32% yield)

## 9 General Procedure for the Synthesis of Esters 2u-2ab (GP3)

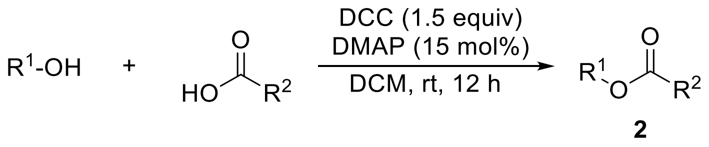


Acid (1.2 equiv), 4-dimethylaminopyridine (DMAP, 15 mol%) and dicyclohexylcarbodiimide (DCC, 1.5 equiv) were dissolved in dichloromethane and stirred at rt for 10 min. Alcohol (1.0 equiv) was slowly added to the reaction mixture that was then stirred at rt for 12 h. The resulting precipitate was removed by filtration. The filtrate was evaporated under reduced pressure. The crude residue was purified by flash column chromatography to give esters **2**.

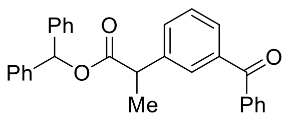


**2u**
$C_{29}H_{24}O_3$
M = 420.51 g/mol

**Benzhydryl 2-(3-benzoylphenyl)propanoate**: (**2u**) Prepared from diphenylmethanol (368.5 mg, 2 mmol, 1.0 equiv) and 2-(3-benzoylphenyl)propanoic acid (610.3 mg, 2.4 mmol, 1.2 equiv) according to the **GP3**. Purification by flash chromatography on silica gel

using petroleum ether/EtOAc (20/1) afforded **2u** as a white solid (832.5 mg, 99% yield).

**R*f*** = 0.60 (petroleum ether/EtOAc = 5/1). **$^1$H NMR** (400 MHz, $CDCl_3$): δ 7.73-7.67 (m, 4H), 7.56 (t, *J* = 7.4 Hz, 1H), 7.50 (d, *J* = 7.6 Hz, 1H), 7.43 (t, *J* = 7.0 Hz, 3H), 7.32-7.17 (m, 8H), 7.13-7.07 (m, 2H), 6.84 (s, 1H), 3.91 (q, *J* = 7.2 Hz, 1H), 1.55 (d, *J* = 7.2 Hz, 3H) ppm. **$^{13}$C NMR** (100 MHz, $CDCl_3$): δ 196.3, 172.7, 140.5, 140.0, 139.9, 138.0, 137.5, 132.4, 131.6, 130.0, 129.3, 128.9, 128.53, 128.47, 128.34, 128.26, 127.9, 127.8, 127.1, 126.7, 77.3, 45.6, 18.0 ppm. **HRMS** (ESI) *m/z*: $[M+Na]^+$ calcd. for $C_{29}H_{24}O_3Na$: 443.1623; found: 443.1622.

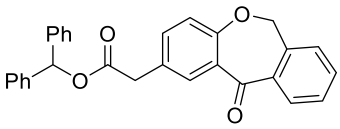


**2v**

$C_{29}H_{22}O_4$

M = 434.49 g/mol

**Benzhydryl 2-(11-oxo-6,11-dihydrodibenzo[*b*,*e*]oxepin-2-yl)acetate**: (**2v**) Prepared from diphenylmethanol (368.5 mg, 2 mmol, 1.0 equiv) and 2-(11-oxo-6,11-dihydrodibenzo[*b*,*e*]oxepin-2-yl)acetic acid (643.8 mg, 2.4 mmol, 1.2 equiv) according to the **GP3**. Purification by flash chromatography on silica gel using petroleum ether/EtOAc (30/1) afforded **2v** as a white solid (832.5 mg, 99% yield).

**R*f*** = 0.60 (petroleum ether/EtOAc = 10/1). **$^1$H NMR** (400 MHz, $CDCl_3$): δ 8.15 (d, J = 1.2 Hz, 1H), 7.89 (d, *J* = 7.6 Hz, 1H), 7.56 (t, *J* = 7.2 Hz, 1H), 7.47 (t, *J* = 7.6 Hz, 1H), 7.42-7.35 (m, 2H), 7.33-7.23 (m, 10H), 7.01 (d, *J* = 8.4 Hz, 1H), 6.88 (s, 1H), 5.18 (s, 2H), 3.75 (s, 2H) ppm. **$^{13}$C NMR** (100 MHz, $CDCl_3$): δ 190.8, 170.3, 160.5, 140.4, 139.9, 136.4, 135.5, 132.7, 132.6, 129.5, 129.3, 128.5, 127.9, 127.8, 127.5, 127.0, 125.1, 121.0, 77.4, 73.6, 40.4 ppm. **HRMS** (ESI) *m/z*: $[M+Na]^+$ calcd. For $C_{29}H_{22}O_4Na$: 457.1416; found: 457.1417.

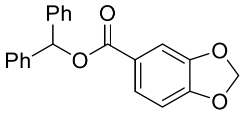

**2w**
$C_{21}H_{16}O_4$
M = 332.36 g/mol

**Benzhydryl benzo[*d*][1,3]dioxole-5-carboxylate**: (**2w**) Prepared from diphenylmethanol (368.5 mg, 2 mmol, 1.0 equiv) and benzo[*d*][1,3]dioxole-5-carboxylic acid (398.7 mg, 2.4 mmol, 1.2 equiv) according to the **GP3**. Purification by flash chromatography on silica gel using petroleum ether/EtOAc (30/1) afforded **2w** as a white solid (651.4 mg, 98% yield).

**$R_f$** = 0.60 (petroleum ether/EtOAc = 5/1). **$^1$H NMR** (400 MHz, $CDCl_3$): δ 7.75 (d, *J* = 8.0 Hz, 1H), 7.55 (s, 1H), 7.41 (d, *J* = 7.6 Hz, 4H), 7.34 (t, *J* = 7.4 Hz, 4H), 7.29-7.23 (m, 2H), 7.08 (s, 1H), 6.84 (d, *J* = 8.4 Hz, 1H), 6.00 (s, 2H) ppm. **$^{13}$C NMR** (100 MHz, $CDCl_3$): δ 164.8, 151.8, 147.8, 140.3, 128.5, 127.9, 127.1, 125.6, 124.2, 109.6, 108.0, 101.8, 77.3 ppm. **HRMS** (ESI) *m/z*: $[M+Na]^+$ calcd. for $C_{21}H_{16}O_4Na$: 355.0947; found: 355.0946.

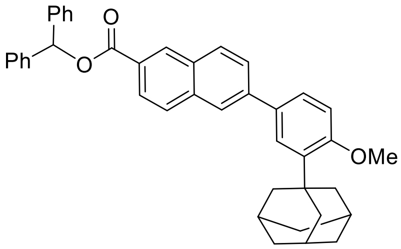

**2x**
$C_{41}H_{38}O_3$
M = 578.75 g/mol

**Benzhydryl 6-(3-(adamantan-1-yl)-4-methoxyphenyl)-2-naphthoate**: (**2x**) Prepared from diphenylmethanol (368.5 mg, 2 mmol, 1.0 equiv) and 6-(3-(adamantan-1-yl)-4-methoxyphenyl)-2-naphthoic acid (990.1 mg, 2.4 mmol, 1.2 equiv) according to the **GP3**. Purification by flash chromatography on silica gel using petroleum ether/EtOAc (20/1) afforded **2x** as a white solid (651.4 mg, 98% yield).

**$R_f$** = 0.55 (petroleum ether/EtOAc = 10/1). **$^1$H NMR** (400 MHz, $CDCl_3$): δ 8.70 (s, 1H), 8.15 (d, *J* = 8.8 Hz, 1H), 8.00 (d, *J* = 9.2 Hz, 2H), 7.92 (d, *J* = 8.4 Hz, 1H), 7.80 (d, *J*

= 8.4 Hz, 1H), 7.60 (s, 1H), 7.56-7.46 (m, 5H), 7.38 (t, *J* = 7.4 Hz, 4H), 7.30 (t, *J* = 7.4 Hz, 2H), 7.20 (s, 1H), 6.99 (d, *J* = 8.4 Hz, 1H), 3.90 (s, 3H), 2.19 (s, 6H), 2.10 (s, 3H), 1.81 (s, 6H) ppm. **$^{13}$C NMR** (100 MHz, $CDCl_3$): δ 165.8, 159.0, 141.5, 140.4, 139.0, 136.1, 132.5, 131.3, 131.1, 129.7, 128.6, 128.3, 128.0, 127.2, 127.0, 126.5, 126.0, 125.7, 125.69, 124.7, 112.2, 77.5, 55.2, 40.7, 37.3, 37.2, 29.1 ppm. **HRMS** (ESI) *m/z*: $[M+Na]^+$ calcd. for $C_{41}H_{38}O_3Na$: 601.2719; found: 601.2708.

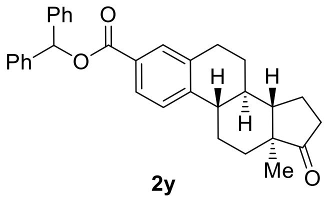


**2y**
$C_{32}H_{32}O_3$
M = 464.61 g/mol

**Benzhydryl (8*R*,9*S*,13*S*,14*S*)-13-methyl-17-oxo-7,8,9,11,12,13,14,15,16,17-decahydro-6*H*-cyclopenta[*a*]phenanthrene-3-carboxylate**: (**2y**) Prepared from diphenylmethanol (368.5 mg, 2 mmol, 1.0 equiv) and (8*R*,9*S*,13*S*,14*S*)-13-methyl-17-oxo-7,8,9,11,12,13,14,15,16,17-decahydro-6*H*-cyclopenta[*a*]phenanthrene-3-carboxylic acid (716.1 mg, 2.4 mmol, 1.2 equiv) according to the **GP3**. Purification by flash chromatography on silica gel using petroleum ether/EtOAc (20/1) afforded **2y** as a white solid (557.5 mg, 60% yield).

**$R_f$** = 0.50 (petroleum ether/EtOAc = 5/1). **$^1$H NMR** (400 MHz, $CDCl_3$): δ 7.91 (d, *J* = 8.4 Hz, 1H), 7.85 (s, 1H), 7.42 (d, *J* = 7.2 Hz, 4H), 7.34(t, *J* = 7.8 Hz, 5H), 7.29-7.24 (m, 2H), 7.11 (s, 1H), 3.00-2.89 (m, 2H), 2.56-2.40 (m, 2H), 2.33 (t, *J* = 10.6 Hz, 1H), 2.19-1.96 (m, 4H), 1.76-1.36 (m, 6H), 0.91 (s, 3H) ppm. **$^{13}$C NMR** (100 MHz, $CDCl_3$): δ 165.6, 145.4, 140.4, 136.8, 130.4, 128.5, 127.9, 127.13, 127.11, 127.07, 125.5, 77.2, 50.5, 47.8, 44.7, 37.8, 35.8, 31.6, 29.2, 26.2, 25.6, 21.7, 13.8 ppm. **HRMS** (ESI) *m/z*: $[M+Na]^+$ calcd. for $C_{32}H_{32}O_3Na$: 487.2249; found: 487.2251.

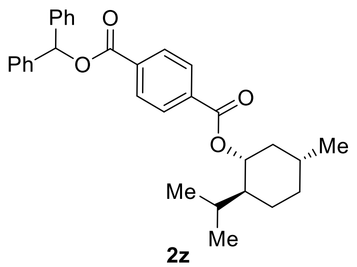

**2z**
$C_{31}H_{34}O_4$
M = 470.61 g/mol

**Benzhydryl ((1*R*,2*S*,5*R*)-2-isopropyl-5-methylcyclohexyl) terephthalate**: (**2z**) Prepared from (1*R*,2*S*,5*R*)-2-isopropyl-5-methylcyclohexan-1-ol (312.5 mg, 2 mmol, 1.0 equiv) and (4-((benzhydryloxy)carbonyl)benzoic acid (797.7 mg, 2.4 mmol, 1.2 equiv) according to the **GP3**. Purification by flash chromatography on silica gel using petroleum ether/EtOAc (30/1) afforded **2z** as a colorless oil (665.4 mg, 70% yield).

$\mathbf{R}_f$ = 0.65 (petroleum ether/EtOAc = 10/1). **$^1$H NMR** (400 MHz, $CDCl_3$): δ 8.19 (d, *J* = 8.4 Hz, 2H), 8.12 (d, *J* = 8.4 Hz, 2H), 7.43 (d, *J* = 7.2 Hz, 4H), 7.35 (t, *J* = 7.2 Hz, 4H), 7.32-7.26 (m, 2H), 7.13 (s, 1H), 4.99-4.92 (m, 1H), 2.14 (d, *J* = 12.0 Hz, 1H), 1.98-1.88 (m, 1H), 1.73 (d, *J* = 12.0 Hz, 2H), 1.59-1.53 (m, 2H), 1.18-1.07 (m, 2H), 0.98-0.90 (m, 7H), 0.80 (d, *J* = 6.8 Hz, 3H) ppm. **$^{13}$C NMR** (100 MHz, $CDCl_3$): δ 165.2, 164.8, 140.0, 134.9, 133.8, 129.7, 129.6, 128.6, 128.0, 127.1, 77.9, 75.5, 47.3, 40.9, 34.3, 31.4, 26.6, 23.7, 22.0, 20.7, 16.6 ppm. **HRMS** (ESI) *m/z*: $[M+Na]^+$ calcd. for $C_{31}H_{34}O_4Na$: 493.2355; found: 493.2358.

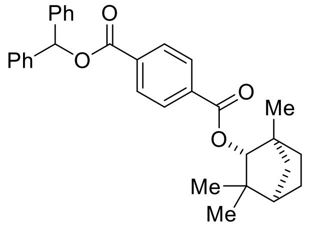

**2aa**
$C_{31}H_{32}O_4$
M = 468.59 g/mol

**Benzhydryl (1,3,3-trimethylbicyclo[2.2.1]heptan-2-yl) terephthalate**: (**2aa**) Prepared from 1,3,3-trimethylbicyclo[2.2.1]heptan-2-ol (308.5 mg, 2 mmol, 1.0 equiv) and (4-((benzhydryloxy)carbonyl)benzoic acid (797.7 mg, 2.4 mmol, 1.2 equiv) according to

the **GP3**. Purification by flash chromatography on silica gel using petroleum ether/EtOAc (30/1) afforded **2aa** as a colorless oil (871.6 mg, 93% yield).

**R*f*** = 0.60 (petroleum ether/EtOAc = 10/1). **$^1$H NMR** (400 MHz, $CDCl_3$): δ 8.22 (d, *J* = 8.4 Hz, 2H), 8.14 (d, *J* = 8.0 Hz, 2H), 7.44 (d, *J* = 8.0 Hz, 4H), 7.37 (t, *J* = 7.4 Hz, 4H), 7.31 (d, *J* = 6.8 Hz, 2H), 7.13 (s, 1H), 4.64 (s, 1H), 1.95-1.88 (m, 1H), 1.79-1.75 (m, 2H), 1.67 (d, *J* = 10.4 Hz, 1H), 1.60-1.47 (m, 2H), 1.27 (d, *J* = 10.4 Hz, 1H), 1.19 (s, 3H), 1.11 (s, 3H), 0.84 (s, 3H) ppm. **$^{13}$C NMR** (100 MHz, $CDCl_3$): δ 166.0, 164.8, 139.9, 134.6, 133.8, 129.8, 129.5, 128.6, 128.1, 127.1, 87.2, 77.9, 48.6, 48.4, 41.4, 39.9, 29.7, 26.8, 25.9, 20.3, 19.5 ppm. **HRMS** (ESI) *m/z*: $[M+Na]^+$ calcd. for $C_{31}H_{32}O_4Na$: 491.2199; found: 491.2199.

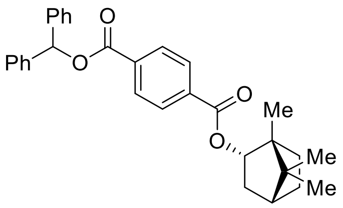


**2ab**
$C_{31}H_{32}O_4$
M = 468.59 g/mol

**Benzhydryl ((1*R*,2*S*,4*R*)-1,7,7-trimethylbicyclo[2.2.1]heptan-2-yl) terephthalate**: (**2ab**) Prepared from (1*R*,2*S*,4*R*)-1,7,7-trimethylbicyclo[2.2.1]heptan-2-ol (308.5 mg, 2 mmol, 1.0 equiv) and (4-((benzhydryloxy)carbonyl)benzoic acid (797.7 mg, 2.4 mmol, 1.2 equiv) according to the **GP3**. Purification by flash chromatography on silica gel using petroleum ether/EtOAc (30/1) afforded **2ab** as a colorless oil (851.1 mg, 91% yield).

**R*f*** = 0.60 (petroleum ether/EtOAc = 10/1). **$^1$H NMR** (400 MHz, $CDCl_3$): δ 8.21 (d, *J* = 8.0 Hz, 2H), 8.14 (d, *J* = 8.4 Hz, 2H), 7.44 (d, *J* = 7.2 Hz, 4H), 7.36 (t, *J* = 7.4 Hz, 4H), 7.30 (t, *J* = 7.0 Hz, 2H), 7.13 (s, 1H), 5.14 (d, *J* = 9.6 Hz, 1H), 2.53-2.45 (m, 1H), 2.15-2.07 (m, 1H), 1.85-1.73 (m, 2H), 1.46-1.38 (m, 1H), 1.34-1.28 (m, 1H), 1.13 (dd, *J* = 13.6, 3.2 Hz, 1H), 0.97 (s, 3H), 0.92 (s, 6H) ppm. **$^{13}$C NMR** (100 MHz, $CDCl_3$): δ 165.9, 164.8, 139.9, 134.8, 133.8, 129.7, 129.5, 128.6, 128.1, 127.1, 81.1, 77.9, 49.1, 47.9, 44.9, 36.8, 28.0, 27.3, 19.7, 18.9, 13.6 ppm. **HRMS** (ESI) *m/z*: $[M+Na]^+$ calcd. for $C_{31}H_{32}O_4Na$: 491.2199; found: 491.2200.

## 10 Scale-Up Experiment

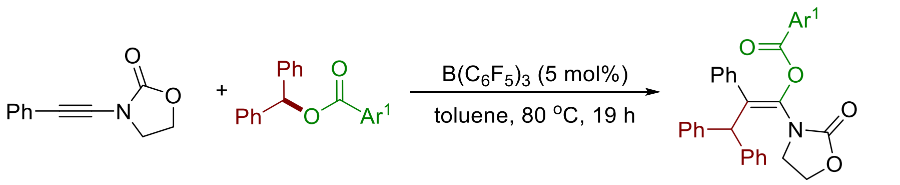


**1a** 3.6 mmol; **2a** (Ar[1] = *p*-$FC_6H_4$) 3.0 mmol; (Z)-**3aa** **1.23 g, 83%, *Z/E* > 96:4**

In glove box, to a flame-dried Schlenk tube is charged with the 3-(phenylethynyl)oxazolidin-2-one **1a** (673.9 mg, 3.6 mmol, 1.2 equiv), benzhydryl 4-fluorobenzoate **2a** (919.0 mg, 3.0 mmol, 1.0 equiv) and $B(C_6F_5)_3$ (76.8 mg, 0.15 mmol, 5 mol%). The tube is evacuated and backfilled with $N_2$ (3 times) followed by the addition of the toluene (30 mL). The reaction mixture is stirred at 80 ℃ for 19 h. Evaporation of the solvents under reduced pressure afforded the crude title compound. Purification by flash chromatography on silica gel using petroleum ether/EtOAc (10/1 to 5/1) afforded (*Z*)-**3aa** as a white solid (1.23 g, 83% yield, *Z*:*E* > 96:4).

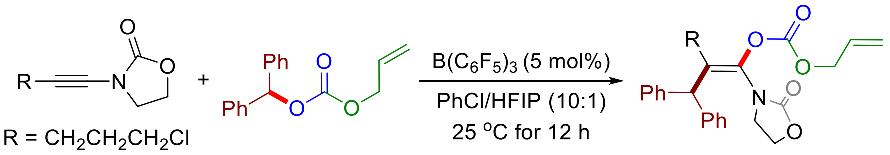


**1o** 1.2 mmol; **5a** 1.0 mmol; (*E*)-**6oa** 378.0 mg, 83%, *E/Z* >96:4

In glove box, to a flame-dried Schlenk tube is charged with the 3-(5-chloropent-1-yn-1-yl)oxazolidin-2-one **1o** (225.1 mg, 1.2 mmol, 1.2 equiv), $B(C_6F_5)_3$ (25.6 mg, 50 μmol, 5 mol%). The tube is evacuated and backfilled with $N_2$ (3 times) followed by the addition of the PhCl/HFIP (5 mL), then, the carbonates were dissolved in PhCl/HFIP (5 mL) and added to the reaction tube at ambient temperature The reaction mixture is stirred at 25 ℃ for 12 h. Evaporation of the solvents under reduced pressure afforded the crude title compound. Purification by flash chromatography on silica gel using petroleum ether/EtOAc (10/1 to 5/1) afforded (*E*)-**6oa** as a white solid (378.0 mg, 83% yield, *E*:*Z* > 96:4).

## 11 Synthetic Transformations

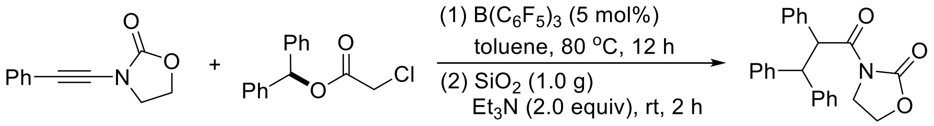


**1a** **2a** **8**

**Synthesis of (8):** In glove box, to a flame-dried Schlenk tube is charged with the 3-(phenylethynyl)oxazolidin-2-one **1a** (44.9 mg, 0.2 mmol, 1.2 equiv), benzhydryl 2-chloroacetate **2a** (52.1 mg, 0.2 mmol, 1.0 equiv) and $B(C_6F_5)_3$ (5.1 mg, 10 μmol, 5 mol%). The tube is evacuated and backfilled with $N_2$ (3 times) followed by the addition of the toluene (2 mL). The reaction mixture is stirred at 80 ℃ for 12 h. After cooling down to room temperature, silica power (1.0 g) and $Et_3N$ (40.5 mg, 0.4 mmol, 2.0 equiv) was added sequentially. The resulting mixture was stirred at room temperature for 2 hours. Then the resulting solution was directly filtered through a pad of silica by EtOAc. The solvent was evaporated in vacuum to give the crude product. Purification by flash chromatography on silica gel using petroleum ether/ EtOAc (10/1 to 5/1) afforded **8** as a colorless oil (68.3 g, 92% yield).

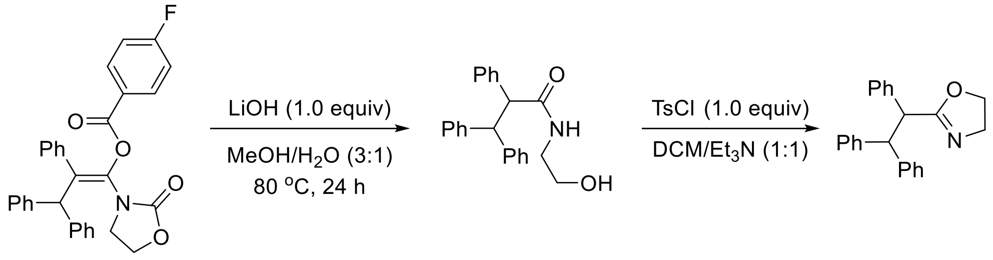


(*Z*)-**3aa** **9** **10**

**Synthesis of (9):** Compound (*Z*)-**3aa** (49.4 mg, 0.1 mmol, 1.0 equiv), LiOH (2.4 mg, 0.1 mmol, 1.0 equiv) and $MeOH/H_2O$ (300 μL/100 μL) were added into a 10 mL glass vial, and the vial was sealed with a PTFE cap. The reaction mixture was stirred at room temperature for 10 min, then heated at 80 ℃ for 24 hours. After being cooled to room temperature, the mixture was filtered through a pad of celite and concentrated in vacuo. The residue was diluted with EtOAc (5 mL) and washed with $NaHCO_3$ (5 mL×2) and brine (5 mL), dried over $Na_2SO_4$ and concentrated in vacuo. The resulting

residue was purification by flash chromatography on silica gel using petroleum ether/ EtOAc (3/1) afforded **9** as a colorless oil (24.5 mg, 71% yield).

**Synthesis of (10):** To a solution of **9** (34.5 mg, 0.1 mmol, 1.0 equiv) in anhydrous dichloromethane (1 mL) and $Et_3N$ (1 mL) was added *p*-toluenesulfonylchloride (19.1 mg, 0.1 mmol, 1.0 equiv). After the reaction mixture was refluxed for 24 h, the solvent was evaporated. To the residue was added water (2 mL), and the aqueous layer was extracted with dichloromethane (3 × 3 mL). The combined organic layer was washed with brine and dried over anhydrous $Na_2SO_4$. After removal of the solvent, the residue was purification by flash chromatography on silica gel using petroleum ether/ EtOAc (5/1) afforded **10** as a colorless oil (20.2 mg, 61% yield).

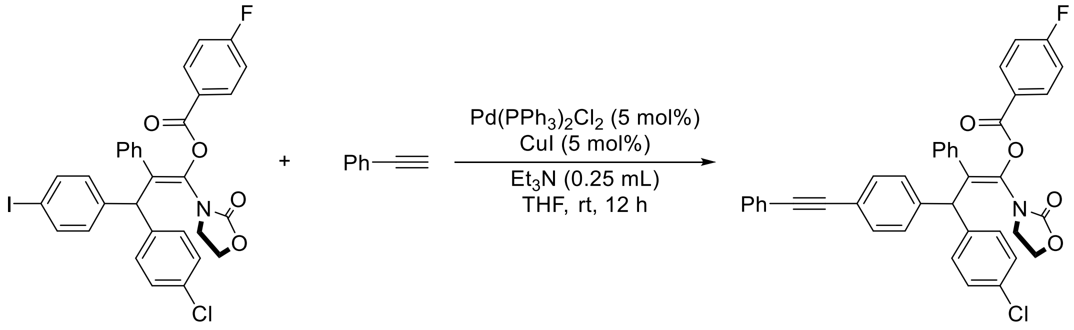


(*Z*)-**3aaf** **11**

**Synthesis of (11):** To an oven-dried sealed tube were added (*Z*)-**3aaf** (57.2 mg, 0.1 mmol, 1.0 equiv), $PdCl_2(PPh_3)_2$ (3.5 mg, 5 μmol, 5 mol%), CuI (1.0 mg, 5 μmol, 5 mol%), DMF (1.5 mL), phenylacetylene (12.3 mg, 0.12 mmol, 1.2 equiv) and $NEt_3$ (0.25 mL) in sequence. The reaction mixture was stirred at room temperature for 12 h, After consumption of the starting material, the mixture was filtered through a pad of celite and concentrated in vacuo. The residue was diluted with EtOAc (5 mL) and washed with $NaHCO_3$ and brine, dried over $Na_2SO_4$ and concentrated in vacuo. Purification by flash chromatography on silica gel using petroleum ether/ EtOAc (10/1) afforded **11** as a colorless oil (53.3 mg, 85% yield, *Z/E* = 89:11).

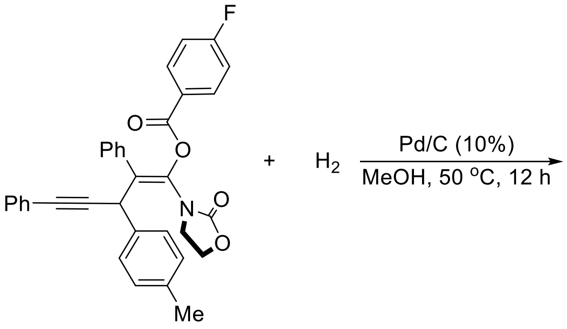


(*Z*)-**3aan** (*Z*)-**12**

**Synthesis of ((*Z*)-12):** (Z)-**3aan** (53.2 mg, 0.1 mmol) was reduced to (*Z*)-**12** in the presence of Pd/C (10.6 mg) in MeOH (2 mL) under $H_2$ (1 atm) at 50 ℃ and stirred for 12 h. The resulting mixture was filtered through a Celite pad and concentrated in vacuo. The residue was purified by flash chromatography on silica gel using petroleum ether/ EtOAc (10/1) afforded (*Z*)-**12** as a colorless oil (48.3 mg, 90% yield, *Z/E* > 96:4).

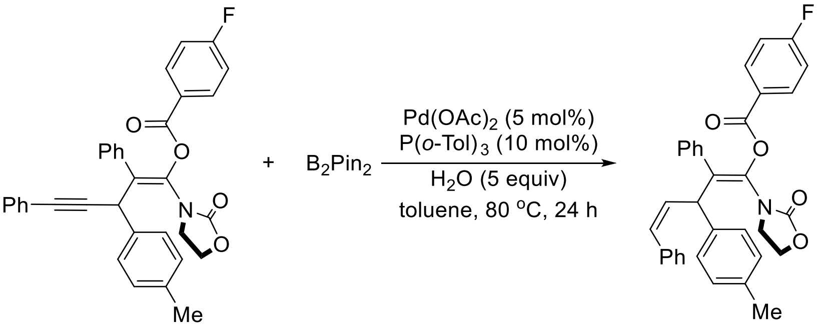


(*Z*)-**3aan** (*Z*)-**13**

**Synthesis of ((*Z*)-13):** An oven dried, screw cap equipped reaction vial with a magnetic stir bar was charged with (*Z*)-**3aan** (53.2 mg, 0.1 mmol, 1.0 equiv), $B_2pin_2$ (30.5 mg, 0.12 mmol. 1.2 equiv), P(*o*-Tol)$_3$ (3.0 mg, 0.01 mmol, 10 mol%), $H_2O$ (9.0 mg, 0.5 mmol, 5 equiv) and $Pd(OAc)_2$ (1.1 mg, 5 μmol, 5 mol%). The reaction mixture was then dissolved in toluene solvent (1.0 mL) and stirred at 80 ℃ for 12 h. The crude mixture was then filtered through a pad of celite and then concentrated. The residue was purified by flash chromatography on silica gel using petroleum ether/ EtOAc (10/1) afforded (*Z*)-**13** as a colorless oil (36.2 mg, 68% yield, *Z/E* > 96:4).

## 12 Mechanistic Control Experiments

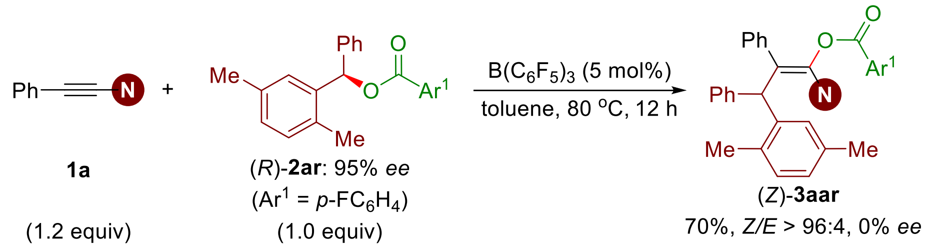


In glove box, to a flame-dried Schlenk tube is charged with the 3-(phenylethynyl)oxazolidin-2-one **1a** (44.9 mg, 0.24 mmol, 1.2 equiv), (*R*)-(2,5-dimethylphenyl)(phenyl)methyl 4-fluorobenzoate (*R*)-**2ar** (66.9 mg, 0.2 mmol, 1.0 equiv, 95% *ee*) and $B(C_6F_5)_3$ (5.1 mg, 0.01 mmol, 5 mol%). The tube is evacuated and backfilled with $N_2$ (3 times) followed by the addition of the toluene (2 mL). The reaction mixture is stirred at 80 ℃ for 12 h. Evaporation of the solvents under reduced pressure afforded the crude title compound. Purification by flash chromatography on silica gel using petroleum ether/EtOAc (10/1 to 5/1) afforded (*Z*)-**3aar** as a white solid (73.0 mg, 70% yield, *Z*:*E* > 96:4, 0% *ee*).

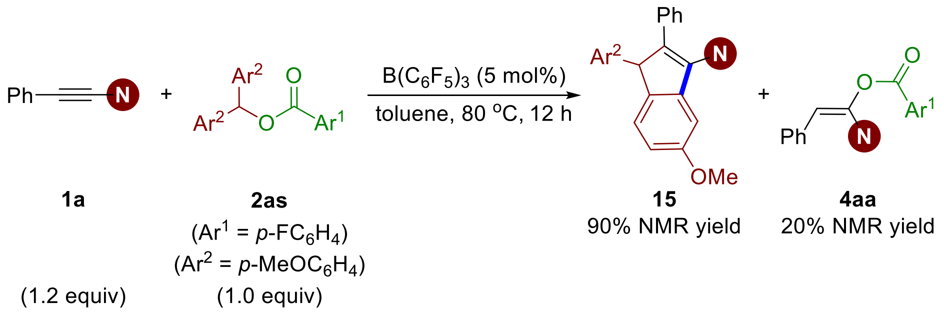


In glove box, to a flame-dried Schlenk tube is charged with the 3-(phenylethynyl)oxazolidin-2-one **1a** (44.9 mg, 0.24 mmol, 1.2 equiv), bis(4-methoxyphenyl)methyl 4-fluorobenzoate **2as** (73.3 mg, 0.2 mmol, 1.0 equiv) and $B(C_6F_5)_3$ (5.1 mg, 0.01 mmol, 5 mol%). The tube is evacuated and backfilled with $N_2$ (3 times) followed by the addition of the toluene (2 mL). The reaction mixture is stirred at 80 ℃ for 12 h. Evaporation of the solvents under reduced pressure afforded the crude title compound (the NMR yield was determined by $^1H$ NMR spectroscopy of the crude reaction mixture with $CH_2Br_2$ as an internal standard). Purification by flash chromatography on silica gel using petroleum ether/EtOAc (10/1 to 5/1) afforded **15** as a white solid (64.5 mg, 78%).

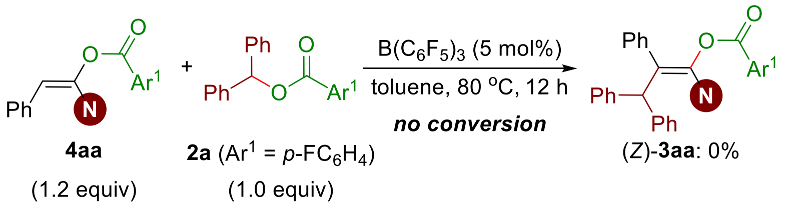


In glove box, to a flame-dried Schlenk tube is charged with the (*E*)-1-(2-oxooxazolidin-3-yl)-2-phenylvinyl 4-fluorobenzoate **4aa** (78.6 mg, 0.24 mmol, 1.2 equiv), benzhydryl 4-fluorobenzoate **2a** (61.3 mg, 0.2 mmol, 1.0 equiv) and $B(C_6F_5)_3$ (5.1 mg, 0.01 mmol, 5 mol%). The tube is evacuated and backfilled with $N_2$ (3 times) followed by the addition of the toluene (2 mL). The reaction mixture is stirred at 80 ℃ for 12 h. Evaporation of the solvents under reduced pressure afforded the crude title compound. The yield of products are determined by $^1$H NMR analysis with $CH_2Br_2$ as the internal standard.

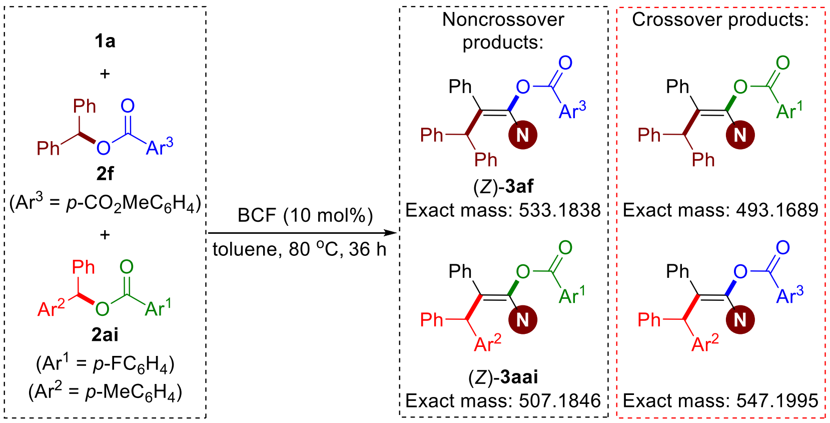


| Mass | 533 | 507 | 493 | 547 |
|---|---|---|---|---|
| Relative Abundance | 35.45% | 57.75% | 3.37% | 3.43% |

In glove box, to a flame-dried Schlenk tube is charged with the 3-(phenylethynyl)oxazolidin-2-one **1a** (74.9 mg, 0.4 mmol, 2.0 equiv), benzhydryl methyl terephthalate **2f** (69.3 mg, 0.2 mmol, 1.0 equiv), phenyl(p-tolyl)methyl 4-fluorobenzoate **2ai** (64.1 mg, 0.2 mmol, 1.0 equiv) and $B(C_6F_5)_3$ (10.2 mg, 0.02 mmol, 10 mol%). The tube is evacuated and backfilled with $N_2$ (3 times) followed by the addition of the toluene (4 mL). The reaction mixture is stirred at 80 ℃ for 36 h. Evaporation of the solvents under reduced pressure afforded the crude title compound. The crude mixture solution was then measured by ESI-MS.

## 13 Characterization Data of the Products

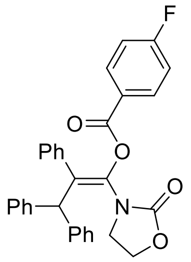


(*Z*)-**3aa**
$C_{31}H_{24}FNO_4$
M = 493.53 g/mol

**(*Z*)-1-(2-oxooxazolidin-3-yl)-2,3,3-triphenylprop-1-en-1-yl 4-fluorobenzoate** ((*Z*)-**3aa**): Prepared from 3-(phenylethynyl)oxazolidin-2-one (**1a**, 44.9 mg, 0.24 mmol) and benzhydryl 4-fluorobenzoate (**2a**, 61.3 mg, 0.2 mmol) according to the **GP1** with 5 mol% $B(C_6F_5)_3$ at 80 ℃ for 12 h. Purification by flash chromatography on silica gel using petroleum ether/EtOAc (10/1 to 5/1) afforded (*Z*)-**3aa** as a white solid (86.9 mg, 88% yield, *Z*:*E* > 96:4).

(*Z*)-**3aa**: $\mathbf{R}_f$ = 0.30 (petroleum ether/EtOAc = 5/1). **$^1$H NMR** (400 MHz, $CDCl_3$): δ 7.75 (dd, *J* = 8.8, 5.6 Hz, 2H), 7.33 (d, *J* = 7.2 Hz, 4H), 7.26 (t, *J* = 7.6 Hz, 4H), 7.20 (d, *J* = 7.2 Hz, 2H), 7.04-6.94 (m, 7H), 5.58 (s, 1H), 4.02 (t, *J* = 8.0 Hz, 2H), 3.58 (t, *J* = 8.0 Hz, 2H) ppm. **$^{13}$C NMR** (100 MHz, $CDCl_3$): δ 166.1 (d, *J* = 254.0 Hz), 164.6, 155.8, 140.6, 136.1, 135.1, 134.0, 132.6 (d, *J* = 9.5 Hz), 129.8, 128.9, 128.2, 127.5, 127.3, 126.7, 124.4 (d, *J* = 2.8 Hz), 115.6 (d, *J* = 22.0 Hz), 62.8, 54.0, 45.3 ppm. $^{19}$F NMR (376 MHz, $CDCl_3$) δ -103.86. **HRMS** (ESI) *m/z*: $[M+Na]^+$ calcd. for $C_{31}H_{24}FNO_4Na$: 516.1587; found: 516.1592.

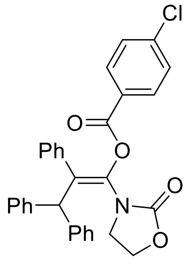


(*Z*)-**3ab**
$C_{31}H_{24}ClNO_4$
M = 509.99 g/mol

**(*Z*)-1-(2-oxooxazolidin-3-yl)-2,3,3-triphenylprop-1-en-1-yl 4-chlorobenzoate** ((*Z*)-**3ab**): Prepared from 3-(phenylethynyl)oxazolidin-2-one (**1a**, 44.9 mg, 0.24 mmol) and benzhydryl 4-chlorobenzoate (**2b**, 64.6 mg, 0.2 mmol) according to the **GP1** with 5 mol% $B(C_6F_5)_3$ at 80 ℃ for 36 h. Purification by flash chromatography on silica gel using petroleum ether/EtOAc (10/1 to 5/1) afforded (*Z*)-**3ab** as a white solid (85.7 mg, 84% yield, *Z*:*E* = 95:5).

(*Z*)-**3ab**: $\mathbf{R_f}$ = 0.30 (petroleum ether/EtOAc = 5/1). $^1$**H NMR** (400 MHz, $CDCl_3$): δ 7.67 (d, *J* = 8.8 Hz, 2H), 7.33 (d, *J* = 7.6 Hz, 4H), 7.30-7.24 (m, 6H), 7.22-7.18 (m, 2H), 7.03 -6.98(m, 5H), 5.58 (s, 1H), 4.03 (t, *J* = 8.0 Hz, 2H), 3.59 (t, *J* = 8.0 Hz, 2H) ppm. $^{13}$**C NMR** (100 MHz, $CDCl_3$): δ 164.8, 155.8, 140.5, 140.2, 136.0, 135.0, 134.1, 131.3, 129.6, 128.9, 128.7, 128.2, 127.5, 127.3, 126.7, 126.6, 62.8, 54.0, 45.3 ppm. **HRMS** (ESI) *m/z*: $[M+Na]^+$ calcd. for $C_{31}H_{24}ClNO_4Na$: 532.1292; found: 532.1303.

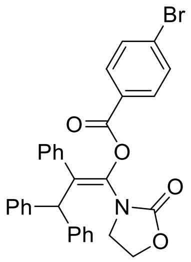


(*Z*)-**3ac**
$C_{31}H_{24}BrNO_4$
M = 554.44 g/mol

**(*Z*)-1-(2-oxooxazolidin-3-yl)-2,3,3-triphenylprop-1-en-1-yl 4-bromobenzoate**: ((*Z*)-**3ac**) Prepared from 3-(phenylethynyl)oxazolidin-2-one (**1a**, 44.9 mg, 0.24 mmol) and benzhydryl 4-bromobenzoate (**2c**, 73.4 mg, 0.2 mmol) according to the **GP1** with 10 mol% $B(C_6F_5)_3$ at 80 ℃ for 24 h. Purification by flash chromatography on silica gel using petroleum ether/EtOAc (10/1 to 5/1) afforded (*Z*)-**3ac** as a white solid (82.7 mg, 75% yield,*Z*:*E* = 94:6).

(*Z*)-**3ac**: $\mathbf{R_f}$ = 0.35 (petroleum ether/EtOAc = 5/1). $^1$**H NMR** (400 MHz, $CDCl_3$): δ 7.59 (d, *J* = 8.4 Hz, 2H), 7.45 (d, *J* = 8.4 Hz, 2H), 7.33 (d, *J* = 7.6 Hz, 4H), 7.27 (t, *J* = 7.6 Hz, 4H), 7.21 (t, *J* = 7.2 Hz, 2H), 7.03-6.99 (m, 5H), 5.57 (s, 1H), 4.03 (t, *J* = 8.0 Hz, 2H), 3.58 (t, *J* = 8.0 Hz, 2H) ppm. $^{13}$**C NMR** (100 MHz, $CDCl_3$): δ 164.4, 155.8, 140.5,

136.0, 135.0, 134.4, 131.5, 130.3, 129.6, 128.8, 128.2, 127.6, 127.4, 126.7, 125.40, 125.36, 62.8, 54.1, 45.3 ppm. **HRMS** (ESI) *m/z*: $[M+Na]^+$ calcd. for $C_{31}H_{24}BrNO_4Na$: 576.0790; found: 576.0792.

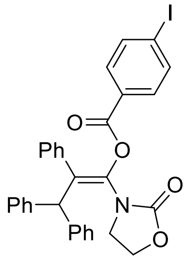


(*Z*)-**3ad**
$C_{31}H_{24}INO_4$
M = 601.44 g/mol

**(*Z*)-1-(2-oxooxazolidin-3-yl)-2,3,3-triphenylprop-1-en-1-yl 4-iodobenzoate**: ((*Z*)-**3ad**) Prepared from 3-(phenylethynyl)oxazolidin-2-one (**1a**, 44.9 mg, 0.24 mmol) and benzhydryl 4-iodobenzoate (**2d**, 82.8 mg, 0.2 mmol) according to the **GP1** with 10 mol% $B(C_6F_5)_3$ at 80 ℃ for 36 h. Purification by flash chromatography on silica gel using petroleum ether/EtOAc (10/1 to 5/1) afforded (*Z*)-**3ad** as a white solid (101.7 mg, 85% yield, *Z*:*E* = 94:6).

(*Z*)-**3ad**: $\mathbf{R}_f$ = 0.30 (petroleum ether/EtOAc = 5/1). **$^1$H NMR** (400 MHz, $CDCl_3$): δ 7.66 (d, *J* = 8.4 Hz, 2H), 7.43 (d, *J* = 8.0 Hz, 2H), 7.32 (d, *J* = 7.6 Hz, 4H), 7.26 (t, *J* = 7.4 Hz, 4H), 7.20 (t, *J* = 7.2 Hz, 2H), 7.02-6.96 (m, 5H), 5.57 (s, 1H), 4.02 (t, *J* = 8.0 Hz, 2H), 3.57 (t, *J* = 8.0 Hz, 2H) ppm. **$^{13}$C NMR** (100 MHz, $CDCl_3$): δ 165.1, 155.7, 140.5, 137.7, 136.0, 135.0, 134.1, 131.2, 129.6, 128.9, 128.2, 127.6, 127.5, 127.3, 126.7, 101.8, 62.8, 54.0, 45.2 ppm. **HRMS** (ESI) *m/z*: $[M+Na]^+$ calcd. for $C_{31}H_{24}INO_4Na$: 624.0648; found: 624.0655.

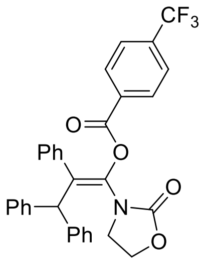


(*Z*)-**3ae**
$C_{32}H_{24}F_3NO_4$
M = 543.54 g/mol

**(*Z*)-1-(2-oxooxazolidin-3-yl)-2,3,3-triphenylprop-1-en-1-yl 4-(trifluoromethyl)benzoate**: ((*Z*)-**3ae**) Prepared from 3-(phenylethynyl)oxazolidin-2-one (**1a**, 44.9 mg, 0.24 mmol) and benzhydryl 4-(trifluoromethyl)benzoate (**2e**, 71.3 mg, 0.2 mmol) according to the **GP1** with 10 mol% $B(C_6F_5)_3$ at 80 ℃ for 24 h. Purification by flash chromatography on silica gel using petroleum ether/EtOAc (10/1 to 5/1) afforded (*Z*)-**3ae** as a white solid (84.3 mg, 78% yield, *Z*:*E* > 96:4).

(*Z*)-**3ae**: **R*f*** = 0.40 (petroleum ether/EtOAc = 5/1). **$^1$H NMR** (400 MHz, $CDCl_3$): δ 7.66 (d, *J* = 8.4 Hz, 2H), 7.43 (d, *J* = 8.0 Hz, 2H), 7.32 (d, *J* = 7.6 Hz, 4H), 7.26 (t, *J* = 7.4 Hz, 4H), 7.20 (t, *J* = 7.2 Hz, 2H), 7.02-6.96 (m, 5H), 5.58 (s, 1H), 4.04 (t, *J* = 8.0 Hz, 2H), 3.60 (t, *J* = 8.0 Hz, 2H) ppm. **$^{13}$C NMR** (100 MHz, $CDCl_3$): δ 164.4, 155.7, 140.5, 136.0, 135.1, 135.0 (q, *J* = 32.5 Hz), 131.5, 130.3, 129.7, 128.9, 128.2, 127.6, 127.4, 126.8, 125.4 (q, *J* = 3.7 Hz), 123.4 (q, *J* = 271.2 Hz), 62.8, 54.1, 45.3 ppm. $^{19}$F NMR (376 MHz, $CDCl_3$) δ -63.29. **HRMS** (ESI) *m/z*: $[M+Na]^+$ calcd. for $C_{32}H_{24}F_3NO_4Na$: 566.1555; found: 566.1559.

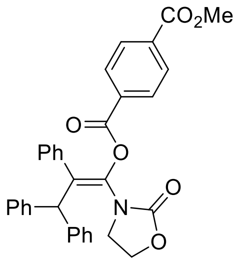


(*Z*)-**3af**
$C_{33}H_{27}NO_6$
M = 533.58 g/mol

**(*Z*)-methyl (1-(2-oxooxazolidin-3-yl)-2,3,3-triphenylprop-1-en-1-yl) terephthalate**: ((*Z*)-**3af**) Prepared from 3-(phenylethynyl)oxazolidin-2-one (**1a**, 44.9 mg, 0.24 mmol) and benzhydryl methyl terephthalate (**2f**, 69.3 mg, 0.2 mmol) according to the **GP1** with 5 mol% $B(C_6F_5)_3$ at 80 ℃ for 36 h. Purification by flash chromatography on silica gel using petroleum ether/EtOAc (10/1 to 5/1) afforded (*Z*)-**3af** as a white solid (90.7 mg, 85% yield, *Z*:*E* > 96:4).

(*Z*)-**3af**: **R*f*** = 0.35 (petroleum ether/EtOAc = 5/1). **$^1$H NMR** (400 MHz, $CDCl_3$): δ 7.96 (d, *J* = 8.4 Hz, 2H), 7.78 (d, *J* = 8.4 Hz, 2H), 7.33 (d, *J* = 7.2 Hz, 4H), 7.27 (t, *J* = 7.4

Hz, 4H), 7.21 (t, *J* = 7.2 Hz, 2H), 7.05-6.95 (m, 5H), 5.58 (s, 1H), 4.04 (t, *J* = 8.0 Hz, 2H), 3.90 (s, 3H), 3.60 (t, *J* = 8.0 Hz, 2H) ppm. **$^{13}$C NMR** (100 MHz, $CDCl_3$): δ 165.9, 164.8, 155.8, 140.5, 136.0, 135.1, 134.5, 134.2, 131.9, 129.8, 129.6, 129.4, 128.9, 128.2, 127.5, 127.3, 126.7, 62.8, 54.0, 52.4, 45.3 ppm. **HRMS** (ESI) *m/z*: $[M+Na]^+$ calcd. for $C_{33}H_{27}NO_6Na$: 556.1736; found: 556.1739.

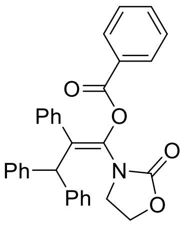


(*Z*)-**3ag**
$C_{31}H_{25}NO_4$
M = 475.54 g/mol

**(*Z*)-1-(2-oxooxazolidin-3-yl)-2,3,3-triphenylprop-1-en-1-yl benzoate** ((*Z*)-**3ag**): Prepared from 3-(phenylethynyl)oxazolidin-2-one (**1a**, 44.9 mg, 0.24 mmol) and benzhydryl benzoate (**2g**, 57.7 mg, 0.2 mmol) according to the **GP1** with 10 mol% $B(C_6F_5)_3$ at 80 ℃ for 36 h. Purification by flash chromatography on silica gel using petroleum ether/EtOAc (10/1 to 5/1) afforded (*Z*)-**3ag** as a white solid (63.7 mg, 67% yield, *Z*:*E* = 94:6).

(*Z*)-**3ag**: **$R_f$** = 0.40 (petroleum ether/EtOAc = 5/1). **$^1$H NMR** (400 MHz, $CDCl_3$): δ 7.75 (d, *J* = 7.6 Hz, 2H), 7.48 (t, *J* = 7.4 Hz, 1H), 7.35-7.24 (m, 10H), 7.20 (t, *J* = 7.0 Hz, 2H), 7.06-6.95 (m, 5H), 5.60 (s, 1H), 4.02 (t, *J* = 8.0 Hz, 2H), 3.59 (t, *J* = 8.0 Hz, 2H). ppm. **$^{13}$C NMR** (100 MHz, $CDCl_3$): δ 165.6, 155.8, 140.7, 136.1, 135.1, 133.9, 133.6, 129.9, 129.7, 129.0, 128.3, 128.2, 127.5, 127.2, 126.6, 62.8, 54.0, 45.2 ppm. **HRMS** (ESI) *m/z*: $[M+Na]^+$ calcd. for $C_{31}H_{25}NO_4Na$: 498.1682; found: 498.1684.

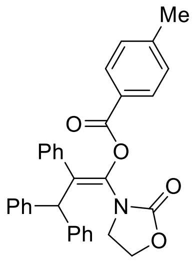


($Z$)-**3ah**
$C_{32}H_{27}NO_4$
M = 489.57 g/mol

**(*Z*)-1-(2-oxooxazolidin-3-yl)-2,3,3-triphenylprop-1-en-1-yl 4-methylbenzoate** ((*Z*)-**3ah**): Prepared from 3-(phenylethynyl)oxazolidin-2-one (**1a**, 44.9 mg, 0.24 mmol) and benzhydryl 4-methylbenzoate (**2h**, 60.5 mg, 0.2 mmol) according to the **GP1** with 5 mol% $B(C_6F_5)_3$ at 100 ℃ for 36 h. Purification by flash chromatography on silica gel using petroleum ether/EtOAc (10/1 to 5/1) afforded (*Z*/*E*)-**3ah** as a white solid (66.6 mg, 68% yield, *Z*:*E* = 88:12).

(*Z*/*E*)-**3ah**: $\mathbf{R_f}$ = 0.35 (petroleum ether/EtOAc = 5/1). (*Z*)-**3ah: $^1$H NMR** (400 MHz, $CDCl_3$): δ 7.64 (d, *J* = 8.0 Hz, 2H), 7.34 (d, *J* = 7.2 Hz, 4H), 7.26 (t, *J* = 7.4 Hz, 4H), 7.19 (d, *J* = 7.2 Hz, 2H), 7.09 (d, *J* = 8.0 Hz, 2H), 7.05 (d, *J* = 6.4 Hz, 2H), 7.01-6.93 (m, 3H), 5.60 (s, 1H), 4.02-3.95 (m, 2H), 3.57 (t, *J* = 8.0 Hz, 2H), 2.31 (s, 3H) ppm. (*E*)-**3ah**: **$^1$H NMR** (400 MHz, $CDCl_3$): δ 7.76 (d, *J* = 8.0 Hz, 2H), 7.26 (t, *J* = 7.4 Hz, 4H), 7.19 (d, *J* = 7.2 Hz, 2H), 7.17-7.12 (m, 9H), 7.09 (d, *J* = 8.0 Hz, 2H), 5.42 (s, 1H), 4.02-3.95 (m, 2H), 3.47 (t, *J* = 8.0 Hz, 2H), 2.38 (s, 3H) ppm. (*Z*/*E*)-**3ah: $^{13}$C NMR** (100 MHz, $CDCl_3$): δ 165.6, 164.6, 156.2, 155.9, 144.7, 144.6, 140.8, 140.2, 136.8, 136.6, 136.2, 135.2, 133.8, 131.5, 130.4, 130.1, 129.8, 129.5, 129.12, 129.09, 129.05, 129.0, 128.4, 128.3, 128.2, 127.9, 127.5, 127.2, 126.7, 126.6, 125.45, 125.39, 63.0, 62.8, 54.3, 54.0, 45.7, 45.3, 21.8, 21.7 ppm. **HRMS** (ESI) *m/z*: $[M+Na]^+$ calcd. for $C_{31}H_{25}NO_4Na$: 512.1838; found: 512.1839.

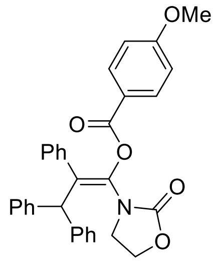


($Z$)-**3ai**
$C_{32}H_{27}NO_5$
M = 505.57 g/mol

**(*Z*)-1-(2-oxooxazolidin-3-yl)-2,3,3-triphenylprop-1-en-1-yl 4-methoxybenzoate** ((*Z*)-**3ai**): Prepared from 3-(phenylethynyl)oxazolidin-2-one (**1a**, 44.9 mg, 0.24 mmol) and benzhydryl 4-methoxybenzoate (**2i**, 63.7 mg, 0.2 mmol) according to the **GP1** with 5 mol% $B(C_6F_5)_3$ at 80 ℃ for 12 h. Purification by flash chromatography on silica gel using petroleum ether/EtOAc (10/1 to 5/1) afforded (*Z*/*E*)-**3ai** as a white solid (75.0 mg, 70% yield, *Z*:*E* = 89:11).

(*Z*/*E*)-**3ai**: **R*f*** = 0.35 (petroleum ether/EtOAc = 5/1). (*Z*)-**3ai:** $^{1}$**H NMR** (400 MHz, $CDCl_3$): δ 7.71 (d, *J* = 8.8 Hz, 2H), 7.34 (d, *J* = 7.6 Hz, 4H), 7.28-7.22 (m, 4H), 7.21-7.16 (m, 3H), 7.10-6.94 (m, 4H), 6.77 (d, *J* = 9.2 Hz, 2H), 5.59 (s, 1H), 4.03-3.95 (m, 2H), 3.78 (s, 3H), 3.58 (t, *J* = 8.0 Hz, 2H) ppm. (*E*)-**3ai**: $^{1}$**H NMR** (400 MHz, $CDCl_3$): δ 7.82 (d, *J* = 8.8 Hz, 2H), 7.28-7.22 (m, 4H), 7.21-7.16 (m, 3H), 7.14-7.09 (m, 4H), 7.05-6.94 (m, 4H), 6.84 (d, *J* = 8.8 Hz, 2H), 5.41 (s, 1H), 4.03-3.95 (m, 2H), 3.83 (s, 3H), 3.47 (t, *J* = 8.0 Hz, 2H) ppm. (*Z*/*E*)-**3ai:** $^{13}$**C NMR** (100 MHz, $CDCl_3$): δ 165.2, 164.2, 164.0, 163.9, 156.1, 155.8, 140.8, 140.2, 136.8, 136.6, 136.2, 135.2, 133.6, 132.4, 132.1, 131.3, 129.7, 129.4, 129.0, 128.9, 128.3, 128.2, 128.1, 127.4, 127.1, 126.6, 126.5, 120.5, 120.4, 113.61, 113.58, 63.0, 62.7, 55.43, 55.36, 54.2, 53.9, 45.6, 45.2 ppm. **HRMS** (ESI) *m/z*: $[M+Na]^+$ calcd. for $C_{32}H_{27}NO_5Na$: 528.1787; found: 528.1788.

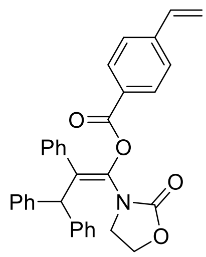

(*Z*)-**3aj**
$C_{33}H_{27}NO_4$
M = 501.58 g/mol

**(*Z*)-1-(2-oxooxazolidin-3-yl)-2,3,3-triphenylprop-1-en-1-yl 4-vinylbenzoate** ((*Z*)-**3aj**): Prepared from 3-(phenylethynyl)oxazolidin-2-one (**1a**, 44.9 mg, 0.24 mmol) and benzhydryl 4-vinylbenzoate (**2j**, 62.9 mg, 0.2 mmol) according to the **GP1** with 5 mol% $B(C_6F_5)_3$ at 80 ℃ for 36 h. Purification by flash chromatography on silica gel using petroleum ether/EtOAc (10/1 to 5/1) afforded (*Z/E*)-**3aj** as a white solid (67.1 mg, 67% yield, *Z*:*E* = 92:8).

(*Z/E*)-**3aj**: **R*f*** = 0.30 (petroleum ether/EtOAc = 5/1). (*Z*)-**3aj: $^{1}$H NMR** (400 MHz, $CDCl_3$): δ 7.70 (d, *J* = 8.4 Hz, 2H), 7.40-7.16 (m, 12H), 7.14-6.93 (m, 5H), 6.66 (dd, *J* = 17.6, 11.2 Hz, 1H), 5.80 (d, *J* = 17.6 Hz, 1H), 5.59 (s, 1H), 5.35 (d, *J* = 10.8 Hz, 1H), 4.03-3.97 (m, 2H), 3.58 (t, *J* = 8.0 Hz, 2H) ppm. (*E*)-**3aj**: **$^{1}$H NMR** (400 MHz, $CDCl_3$): δ 7.81 (d, *J* = 8.4 Hz, 2H), 7.40-7.16 (m, 12H), 7.14-6.93 (m, 5H), 6.72 (dd, *J* = 17.6, 11.2 Hz, 1H), 5.86 (d, *J* = 17.6 Hz, 1H), 5.41 (s, 1H), 5.39 (d, *J* = 10.8 Hz, 1H), 4.03-3.97 (m, 2H), 3.47 (t, *J* = 8.0 Hz, 2H) ppm. (*Z*/*E*)-**3aj: $^{13}$C NMR** (100 MHz, $CDCl_3$): δ 165.3, 155.8, 142.6, 140.7, 140.1, 136.1, 135.8, 135.7, 135.1, 133.8, 130.6, 130.3, 129.7, 129.4, 128.93, 128.85, 128.4, 128.2, 128.1, 127.5, 127.17, 127.15, 126.6, 126.6, 126.0, 125.96 117.0, 63.0, 62.7, 54.4, 54.0, 45.6, 45.2 ppm. **HRMS** (ESI) *m/z*: $[M+Na]^+$ calcd. for $C_{33}H_{27}NO_4Na$: 524.1838; found: 524.1845.

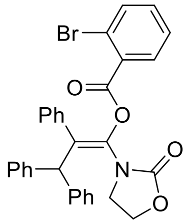

(*Z*)-**3ak**
$C_{31}H_{24}BrNO_4$
M = 554.44 g/mol

**(*Z*)-1-(2-oxooxazolidin-3-yl)-2,3,3-triphenylprop-1-en-1-yl 2-bromobenzoate** ((*Z*)-**3ak**): Prepared from 3-(phenylethynyl)oxazolidin-2-one (**1a**, 44.9 mg, 0.24 mmol) and benzhydryl 2-bromobenzoate (**2k**, 73.4 mg, 0.2 mmol) according to the **GP1** with 10 mol% $B(C_6F_5)_3$ at 80 ℃ for 12 h. Purification by flash chromatography on silica gel using petroleum ether/EtOAc (10/1 to 5/1) afforded (*Z*)-**3ak** as a white solid (73.9mg, 67% yield, *Z*:*E* > 96:4).

(*Z*)-**3ak**: **R*f*** = 0.35 (petroleum ether/EtOAc = 5/1). **$^1$H NMR** (400 MHz, $CDCl_3$): δ 7.55 (d, *J* = 8.0 Hz, 1H), 7.34 (d, *J* = 7.2 Hz, 4H), 7.27 (t, *J* = 7.2 Hz, 4H), 7.22-7.18 (m, 3H), 7.14-7.08 (m, 6H), 6.96 (d, *J* = 8.0 Hz, 1H), 5.57 (s, 1H), 4.04 (t, *J* = 7.6 Hz, 2H), 3.65 (t, *J* = 7.6 Hz, 2H) ppm. **$^{13}$C NMR** (100 MHz, $CDCl_3$): δ 164.8, 155.8, 140.4, 136.2, 134.8, 134.4, 134.0, 133.0, 131.2, 130.3, 129.7, 129.0, 128.2, 127.7, 127.4, 127.0, 126.7, 121.6, 62.9, 54.2, 45.4 ppm. **HRMS** (ESI) *m/z*: $[M+Na]^+$ calcd. for $C_{31}H_{24}BrNO_4Na$: 576.0787; found: 576.0790.

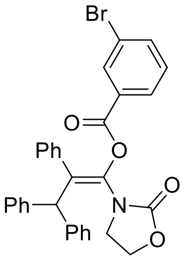


(*Z*)-**3al**
$C_{31}H_{24}BrNO_4$
M = 554.44 g/mol

**(*Z*)-1-(2-oxooxazolidin-3-yl)-2,3,3-triphenylprop-1-en-1-yl 3-bromobenzoate** ((*Z*)-**3al**): Prepared from 3-(phenylethynyl)oxazolidin-2-one (**1a**, 44.9 mg, 0.24 mmol) and benzhydryl 3-bromobenzoate (**2l**, 73.4 mg, 0.2 mmol) according to the **GP1** with 10 mol% $B(C_6F_5)_3$ at 80 ℃ for 12 h. Purification by flash chromatography on silica gel using petroleum ether/EtOAc (10/1 to 5/1) afforded (*Z*)-**3al** as a white solid (92.2 mg, 84% yield, *Z*:*E* = 95:5).

(*Z*)-**3al**: **R*f*** = 0.35 (petroleum ether/EtOAc = 5/1). **$^1$H NMR** (400 MHz, $CDCl_3$): δ 7.84 (s, 1H), 7.65 (d, *J* = 7.6 Hz, 1H), 7.59 (d, *J* = 8.4 Hz, 1H), 7.33 (d, *J* = 7.6 Hz, 4H), 7.26 (t, *J* = 7.4 Hz, 4H), 7.22-7.14 (m, 3H), 7.08-6.95 (m, 5H), 5.58 (s, 1H), 4.02 (t, *J*

= 8.0 Hz, 2H), 3.57 (t, *J* = 8.0 Hz, 2H) ppm. **$^{13}$C NMR** (100 MHz, $CDCl_3$): δ 164.3, 155.7, 140.5, 136.5, 136.0, 135.0, 134.2, 132.8, 130.1, 129.9, 129.6, 128.9, 128.4, 128.2, 127.6, 127.3, 126.7, 122.3, 62.8, 54.0, 45.2 ppm. **HRMS** (ESI) *m/z*: $[M+Na]^+$ calcd. for $C_{31}H_{24}BrNO_4Na$: 576.0787; found: 576.0795.

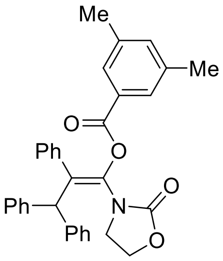


(*Z*)-**3am**
$C_{33}H_{29}NO_4$
M = 503.60 g/mol

**(*Z*)-1-(2-oxooxazolidin-3-yl)-2,3,3-triphenylprop-1-en-1-yl 3,5-dimethylbenzoate** ((*Z*)-**3am**): Prepared from 3-(phenylethynyl)oxazolidin-2-one (**1a**, 44.9 mg, 0.24 mmol) and benzhydryl 3,5-dimethylbenzoate (**2m**, 63.3 mg, 0.2 mmol) according to the **GP1** with 5 mol% $B(C_6F_5)_3$ at 100 ℃ for 40 h. Purification by flash chromatography on silica gel using petroleum ether/EtOAc (10/1 to 5/1) afforded (*Z/E*)-**3am** as a white solid (72.5 mg, 72% yield, *Z*:*E* = 90:10).

(*Z/E*)-**3am**: **$R_f$** = 0.30 (petroleum ether/EtOAc = 5/1). (*Z*)-**3am: $^1$H NMR** (400 MHz, $CDCl_3$): δ 7.34 (d, *J* = 8.0 Hz, 5H), 7.29-7.16 (m, 7H), 7.14-6.94 (m, 6H), 5.59 (s, 1H), 4.00 (t, *J* = 8.0 Hz, 2H), 3.57 (t, *J* = 8.0 Hz, 2H), 2.23 (s, 6H) ppm. (*E*)-**3am: $^1$H NMR** (400 MHz, $CDCl_3$): δ 7.48 (s, 2H), δ 7.34 (d, *J* = 8.0 Hz, 3H), 7.29-7.16 (m, 7H), 7.14-6.94 (m, 6H), 5.43 (s, 1H), 4.00 (t, *J* = 8.0 Hz, 2H), 3.48 (t, *J* = 8.0 Hz, 2H), 2.31 (s, 6H) ppm.**$^{13}$C NMR** (100 MHz, $CDCl_3$): δ 165.9, 165.0, 155.8, 155.5, 140.7, 140.2, 138.0, 137.9, 136.1, 135.3, 135.1, 133.7, 129.7, 129.4, 129.01, 128.96, 128.3, 128.2, 128.1, 127.99, 127.97, 127.7, 127.4, 127.1, 126.6, 126.4, 62.9, 62.7, 54.2, 54.0, 45.7, 45.2, 21.0, 20.9 ppm. **HRMS** (ESI) *m/z*: $[M+Na]^+$ calcd. for $C_{33}H_{29}NO_4Na$: 526.1995; found: 526.1996.

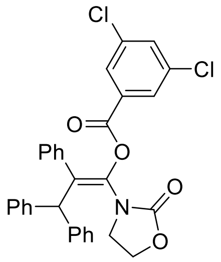

(*Z*)-**3an**
$C_{31}H_{23}Cl_2NO_4$
M = 544.43 g/mol

**(*Z*)-1-(2-oxooxazolidin-3-yl)-2,3,3-triphenylprop-1-en-1-yl 3,5-dichlorobenzoate** ((*Z*)-**3an**): Prepared from 3-(phenylethynyl)oxazolidin-2-one (**1a**, 44.9 mg, 0.24 mmol) and benzhydryl 3,5-dichlorobenzoate (**2n**, 71.4 mg, 0.2 mmol) according to the **GP1** with 10 mol% $B(C_6F_5)_3$ at 80 ℃ for 24 h. Purification by flash chromatography on silica gel using petroleum ether/EtOAc (10/1 to 5/1) afforded (*Z/E*)-**3an** as a white solid (89.3 mg, 82% yield, *Z*:*E* > 96:4).

(*Z*)-**3an**: **R*f*** = 0.35 (petroleum ether/EtOAc = 5/1). **$^1$H NMR** (400 MHz, $CDCl_3$): δ 7.59-7.56 (m, 2H), 7.45 (t, *J* = 2.0 Hz, 1H), 7.32 (d, *J* = 7.6 Hz, 4H), 7.27 (t, *J* = 7.6 Hz, 4H), 7.23-7.18 (m, 2H), 7.08-7.00 (m, 5H), 5.56 (s, 1H), 4.03 (t, *J* = 8.0 Hz, 2H), 3.56 (t, *J* = 8.0 Hz, 2H) ppm. **$^{13}$C NMR** (100 MHz, $CDCl_3$): δ 163.4, 155.7, 140.4, 135.9, 135.2, 135.0, 134.6, 133.4, 131.0, 129.6, 128.8, 128.23, 128.19, 127.7, 127.5, 126.8, 62.8, 54.1, 45.3 ppm. **HRMS** (ESI) *m/z*: $[M+Na]^+$ calcd. for $C_{31}H_{23}Cl_2NO_4Na$: 566.0902; found: 566.0902.

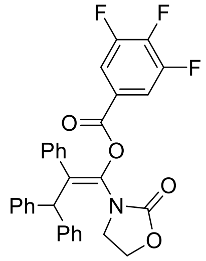

(*Z*)-**3ao**
$C_{31}H_{22}F_3NO_4$
M = 529.52 g/mol

**(*Z*)-1-(2-oxooxazolidin-3-yl)-2,3,3-triphenylprop-1-en-1-yl 3,4,5-trifluorobenzoate** ((*Z*)-**3ao**): Prepared from 3-(phenylethynyl)oxazolidin-2-one (**1a**, 44.9 mg, 0.24 mmol) and benzhydryl 3,4,5-trifluorobenzoate (**2o**, 68.5 mg, 0.2 mmol) according to the **GP1**

with 5 mol% $B(C_6F_5)_3$ at 80 ℃ for 12 h. Purification by flash chromatography on silica gel using petroleum ether/EtOAc (10/1 to 5/1) afforded (*Z*)-**3ao** as a white solid (80.0 mg, 76% yield, *Z*:*E* > 96:4).

(*Z*)-**3ao**: **$R_f$** = 0.35 (petroleum ether/EtOAc = 5/1). **$^1$H NMR** (400 MHz, $CDCl_3$): δ 7.37 (t, *J* = 7.0 Hz, 2H), 7.33-7.25 (m, 8H), 7.23-7.19 (m, 2H), 7.07-6.99 (m, 5H), 5.55 (s, 1H), 4.04 (t, *J* = 8.0 Hz, 2H), 3.57 (t, *J* = 8.0 Hz, 2H) ppm. **$^{13}$C NMR** (100 MHz, $CDCl_3$): δ 162.9, 155.7, 152.1 (dd, *J* = 10.3, 3.3 Hz), 149.6 (dd, *J* = 10.4, 3.4 Hz), 140.3, 135.9, 135.0, 134.6, 129.6, 128.8, 128.3, 127.7, 127.5, 126.8, 114.5(dd, *J* = 16.4, 6.6 Hz), 62.8, 54.1, 45.3 ppm. $^{19}$F NMR (376 MHz, $CDCl_3$) δ -132.1– -132.2 (m, 2F), -150.8– -150.9 (m, 1F) ppm. **HRMS** (ESI) *m/z*: $[M+Na]^+$ calcd. for $C_{31}H_{22}F_3NO_4Na$: 552.1399; found: 552.1405.

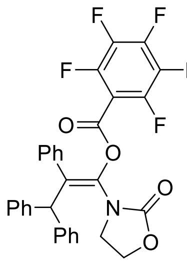


(*Z*)-**3ap**
$C_{31}H_{20}F_5NO_4$
M = 565.50 g/mol

**(*Z*)-1-(2-oxooxazolidin-3-yl)-2,3,3-triphenylprop-1-en-1-yl 2,3,4,5,6-pentafluorobenzoate** ((*Z*)-**3ap**): Prepared from 3-(phenylethynyl)oxazolidin-2-one (**1a**, 44.9 mg, 0.24 mmol) and benzhydryl 2,3,4,5,6-pentafluorobenzoate (**2p**, 75.7 mg, 0.2 mmol) according to the **GP1** with 5 mol% $B(C_6F_5)_3$ at 80 ℃ for 12 h. Purification by flash chromatography on silica gel using petroleum ether/EtOAc (10/1 to 5/1) afforded (*Z*)-**3ap** as a white solid (92.8 mg, 84% yield, *Z*:*E* > 96:4).

(*Z*)-**3ap**: **$R_f$** = 0.35 (petroleum ether/EtOAc = 5/1). **$^1$H NMR** (400 MHz, $CDCl_3$): δ 7.31 (d, *J* = 7.6 Hz, 4H), 7.26 (t, *J* = 7.4 Hz, 4H), 7.22-7.17 (m, 2H), 7.12-7.07 (m, 3H), 7.05-7.02 (m, 2H), 5.56 (s, 1H), 4.01 (t, *J* = 8.0 Hz, 2H), 3.51 (t, *J* = 8.0 Hz, 2H). ppm. **$^{13}$C NMR** (100 MHz, $CDCl_3$): δ 157.6, 155.4, 145.3 (dm, *J* = 266.0 Hz),143.6 (dm, *J* = 250.2 Hz), 140.3, 137.5 (dm, *J* = 249.7 Hz), 135.63, 135.61, 134.1, 129.6,

128.7, 128.2, 127.7, 126.8, 106.5 (m), 62.7, 54.3, 44.9 ppm. $^{19}$F NMR (376 MHz, $CDCl_3$): -136.9– -137.0 (m, 2F), -146.8 (tt, *J* = 21.1, 5.3 Hz, 1F), δ -159.8– -160.0 (m, 2F) ppm. **HRMS** (ESI) *m/z*: $[M+Na]^+$ calcd. for $C_{31}H_{20}F_5NO_4Na$: 588.1210; found: 588.1219.

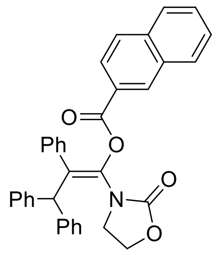

(*Z*)-**3aq**
$C_{35}H_{27}NO_4$
M = 525.60 g/mol

**(*Z*)-1-(2-oxooxazolidin-3-yl)-2,3,3-triphenylprop-1-en-1-yl 2-naphthoate** ((*Z*)-**3aq**): Prepared from 3-(phenylethynyl)oxazolidin-2-one (**1a**, 44.9 mg, 0.24 mmol) and benzhydryl 2-naphthoate (**2q**, 67.7 mg, 0.2 mmol) according to the **GP1** with 10 mol% $B(C_6F_5)_3$ at 80 ℃ for 36 h. Purification by flash chromatography on silica gel using petroleum ether/EtOAc (10/1 to 5/1) afforded (*Z/E*)-**3aq** as a white solid (87.0 mg, 83% yield, *Z*:*E* = 91:9).

(*Z/E*)-**3aq**: **R*f*** = 0.30 (petroleum ether/EtOAc = 5/1). (*Z*)-**3aq**: **$^1$H NMR** (400 MHz, $CDCl_3$): δ 8.29 (s, 1H), 7.90-7.72 (m, 4H), 7.54 (t, *J* = 7.4 Hz, 1H), 7.48 (t, *J* = 7.0 Hz, 1H), 7.37 (d, *J* = 7.6 Hz, 3H), 7.30-7.15 (m, 7H), 7.10 (d, *J* = 7.6 Hz, 2H), 6.99 (t, *J* = 7.4 Hz, 2H), 6.93 (t, *J* = 7.2 Hz, 1H), 5.63 (s, 1H), 4.03 (t, *J* = 8.0 Hz, 2H), 3.63 (t, *J* = 8.0 Hz, 2H) ppm. (*E*)-**3aq**: **$^1$H NMR** (400 MHz, $CDCl_3$): δ 8.39 (s, 1H), 7.90-7.72 (m, 4H), 7.59 (d, *J* = 7.2 Hz, 1H), 7.54 (t, *J* = 7.4 Hz, 1H), 7.48 (t, *J* = 7.0 Hz, 1H), 7.37 (d, *J* = 7.6 Hz, 4H), 7.30-7.15(m, 7H), 7.10 (d, *J* = 7.6 Hz, 2H), 6.99 (t, *J* = 7.4 Hz, 1H), 5.45 (s, 1H), 4.03 (t, *J* = 8.0 Hz, 2H), 3.52 (t, *J* = 8.0 Hz, 2H) ppm.

(*Z/E*)-**3aq**: **$^{13}$C NMR** (100 MHz, $CDCl_3$): δ 165.7, 156.2, 155.9, 140.7, 140.2, 136.6, 136.1, 135.8, 135.7, 135.2, 134.0, 132.3, 132.2, 132.0, 129.7, 129.5, 129.39, 129.37, 129.0, 128.9, 128.4, 128.6, 128.4, 128.23, 128.17, 128.1 127.6, 127.5, 127.2, 126.7,

126.6, 126.5, 125.3, 125.0, 63.0, 62.8, 54.5, 54.0, 45.7, 45.3 ppm. **HRMS** (ESI) *m/z*: $[M+Na]^+$ calcd. for $C_{35}H_{27}NO_4Na$: 548.1838; found: 548.1849.

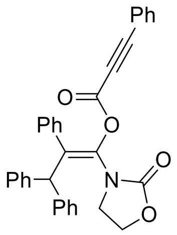

(*Z*)-**3ar**
$C_{33}H_{25}NO_4$
M = 499.57 g/mol

**(*Z*)-1-(2-oxooxazolidin-3-yl)-2,3,3-triphenylprop-1-en-1-yl 3-phenylpropiolate** ((*Z*)-**3ar**): Prepared from 3-(phenylethynyl)oxazolidin-2-one (**1a**, 44.9 mg, 0.24 mmol) and benzhydryl 3-phenylpropiolate (**2r**, 62.5 mg, 0.2 mmol) according to the GP1 with 5 mol% $B(C_6F_5)_3$ at 80 ℃ for 14 h. Purification by flash chromatography on silica gel using petroleum ether/EtOAc (10/1 to 5/1) afforded (*Z/E*)-**3ar** as a white solid (82.9 mg, 83% yield, *Z*:*E* = 83:17).

(*Z/E*)-**3ar**: $R_f$ = 0.40 (petroleum ether/EtOAc = 5/1). (*Z*)-**3ar**: **$^1$H NMR** (400 MHz, $CDCl_3$): δ 7.46 (d, *J* = 7.2 Hz, 2H), 7.44-7.36 (m, 2H), 7.35-7.15 (m, 11H), 7.15-7.09 (m, 3H), 7.08-7.02 (m, 2H), 5.56 (s, 1H), 3.99 (t, *J* = 8.0 Hz, 2H), 3.48 (t, *J* = 8.0 Hz, 2H) ppm. (*E*)-**3ar**: **$^1$H NMR** (400 MHz, $CDCl_3$): δ 7.55 (d, *J* = 7.2 Hz, 2H), 7.35-7.15 (m, 14H), 7.15-7.09 (m, 3H), 7.08-7.02 (m, 1H), 5.46 (s, 1H), 3.99 (t, *J* = 8.0 Hz, 2H), 3.41 (t, *J* = 8.0 Hz, 2H) ppm. (*Z/E*)-**3ar**: **$^{13}$C NMR** (100 MHz, $CDCl_3$): δ 156.0, 155.5, 152.1, 151.4, 140.5, 139.7, 136.2, 135.7, 135.6, 134.8, 134.0, 133.11, 133.07, 132.5, 131.0, 129.6, 129.5, 128.8, 128.7, 128.54, 128.49, 128.32, 128.28, 128.1, 127.6, 127.5, 126.7, 126.6, 119.0, 118.8, 89.2, 79.4, 79.2, 63.0, 62.7, 54.3, 54.1, 45.4, 44.9 ppm. **HRMS** (ESI) *m/z*: $[M+Na]^+$ calcd. for $C_{33}H_{25}NO_4Na$: 522.1682; found: 522.1685.

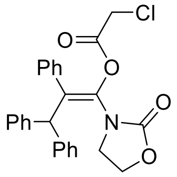

(*Z*)-**3as**
$C_{26}H_{22}ClNO_4$
M = 447.92 g/mol

**(*Z*)-1-(2-oxooxazolidin-3-yl)-2,3,3-triphenylprop-1-en-1-yl 2-chloroacetate** ((*Z*)-**3as**): Prepared from 3-(phenylethynyl)oxazolidin-2-one (**1a**, 44.9 mg, 0.24 mmol) and benzhydryl 2-chloroacetate (**2s**, 52.1 mg, 0.2 mmol) according to the GP1 with 5 mol% $B(C_6F_5)_3$ at 80 ℃ for 12 h. Purification by flash chromatography on silica gel using petroleum ether/EtOAc (10/1 to 5/1) afforded (*Z*)-**3as** as a colorless oil (*Z*:*E* = 95:5). Product **3as** was found to be unstable and was expected to hydrolysis on column during the purification. 95% NMR yield of (*Z*)-**3as** was estimated by $^1$HNMR spectroscopy.

(*Z*)-**3as**: **R*f*** = 0.45 (petroleum ether/EtOAc = 5/1). **$^1$H NMR** (400 MHz, $CDCl_3$): δ 7.30-7.18 (m, 10H), 7.13-7.08 (m, 3H), 7.02-6.96 (m, 2H), 5.50 (s, 1H), 4.03 (t, *J* = 8.0 Hz, 2H), 3.68 (s, 2H), 3.52 (t, *J* = 8.0 Hz, 2H) ppm. **$^{13}$C NMR** (100 MHz, $CDCl_3$): δ 166.6, 155.7, 140.2, 135.8, 134.8, 134.7, 129.6, 128.8, 128.2, 127.8, 127.6, 126.8, 62.8, 54.0, 45.1, 40.0 ppm. **HRMS** (ESI) *m/z*: $[M+Na]^+$ calcd. for $C_{26}H_{22}ClNO_4Na$: 470.1135; Found: 470.1140.

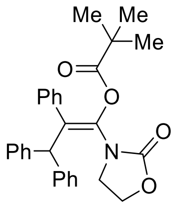


(*Z*)-**3at**
$C_{29}H_{29}NO_4$
M = 455.55 g/mol

**(*Z*)-1-(2-oxooxazolidin-3-yl)-2,3,3-triphenylprop-1-en-1-yl pivalate** ((*Z*)-**3at**): Prepared from 3-(phenylethynyl)oxazolidin-2-one (**1a**, 44.9 mg, 0.24 mmol) and benzhydryl pivalate (**2t**, 53.7 mg, 0.2 mmol) according to the GP1 with 5 mol% $B(C_6F_5)_3$ at 80 ℃ for 24 h. Purification by flash chromatography on silica gel using petroleum ether/EtOAc (10/1 to 5/1) afforded (*Z*)-**3at** as a colorless oil (*Z*:*E* > 96:4). The desired product containing a trace amount hydroacyloxylation side product. 59% NMR yield of (*Z*)-**3at** was estimated by $^1$HNMR spectroscopy.

(*Z*)-**3at**: **R*f*** = 0.40 (petroleum ether/EtOAc = 5/1). **$^1$H NMR** (400 MHz, $CDCl_3$): δ 7.29 (d, *J* = 7.2 Hz, 4H), 7.23 (t, *J* = 7.2 Hz, 4H), 7.17 (t, *J* = 7.0 Hz, 2H), 7.10-7.05 (m,

3H), 6.98-6.95 (m, 2H), 5.51 (s, 1H), 4.00 (t, *J* = 8.0 Hz, 2H), 3.49 (t, *J* = 8.0 Hz, 2H), 0.85 (s, 9H) ppm. **$^{13}$C NMR** (100 MHz, $CDCl_3$): δ 177.4, 155.6, 140.7, 136.2, 134.8, 133.2, 129.7, 129.2, 128.7, 128.1, 128.0, 127.4, 127.1, 126.5, 115.1, 62.6, 53.9, 45.1, 38.6, 26.4 ppm. **HRMS** (ESI) *m/z*: $[M+Na]^+$ calcd. for $C_{29}H_{29}NO_4Na$: 478.1995; found: 478.1995.

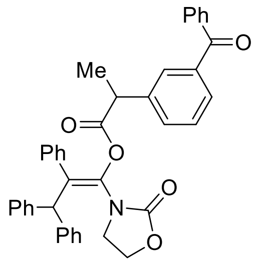


(*Z*)-**3au**
$C_{40}H_{33}NO_5$
M = 607.71 g/mol

**(*Z*)-1-(2-oxooxazolidin-3-yl)-2,3,3-triphenylprop-1-en-1-yl 2-(3-benzoylphenyl)propanoate** ((*Z*)-**3au**): Prepared from 3-(phenylethynyl)oxazolidin-2-one (**1a**, 44.9 mg, 0.24 mmol) and benzhydryl 2-(3-benzoylphenyl)propanoate (**2u**, 84.1 mg, 0.2 mmol) according to the GP1 with 5 mol% $B(C_6F_5)_3$ at 80 ℃ for 12 h. Purification by flash chromatography on silica gel using petroleum ether/EtOAc (10/1 to 5/1) afforded (*Z*)-**3au** as a white solid (89.9 mg, 74% yield, *Z*:*E* = 95:5).

(*Z*)-**3au**: **$R_f$** = 0.35 (petroleum ether/EtOAc = 5/1). **$^1$H NMR** (400 MHz, $CDCl_3$): δ 7.76 (d, *J* = 7.6 Hz, 2H), 7.65-7.57 (m, 2H), 7.52-7.46 (m, 2H), 7.35-7.13 (m, 13H), 7.05-6.96 (m, 3H), 6.91 (d, *J* = 6.8 Hz, 2H), 5.49 (s, 1H), 3.96 (t, *J* = 8.0 Hz, 2H), 3.53 (q, *J* = 7.2 Hz, 1H), 3.38 (t, *J* = 8.0 Hz, 2H), 1.16 (d, *J* = 7.2 Hz, 3H) ppm. **$^{13}$C NMR** (100 MHz, $CDCl_3$): δ 196.2, 172.9, 155.6, 140.49, 140.47, 139.2, 137.7, 137.3, 135.9, 134.6, 133.9, 132.5, 131.4, 123.0, 129.6, 129.57, 129.1, 129.0, 128.9, 128.6, 128.3, 128.1, 128.09, 127.5, 127.3, 126.59, 126.58, 62.7, 54.0, 44.9, 44.6, 17.8 ppm. **HRMS** (ESI) *m/z*: $[M+Na]^+$ calcd. for $C_{40}H_{33}NO_5Na$: 630.2257; Found: 630.2257.

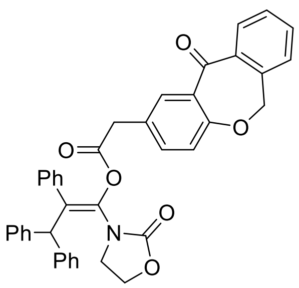

(*Z*)-**3av**
$C_{40}H_{31}NO_6$
M = 621.69 g/mol

**(*Z*)-1-(2-oxooxazolidin-3-yl)-2,3,3-triphenylprop-1-en-1-yl 2-(11-oxo-6,11-dihydrodibenzo[*b*,*e*]oxepin-2-yl)acetate** ((*Z*)-**3av**): Prepared from 3-(phenylethynyl)oxazolidin-2-one (**1a**, 44.9 mg, 0.24 mmol) and benzhydryl 2-(3-benzoylphenyl)propanoate (**2v**, 86.9 mg, 0.2 mmol) according to the GP1 with 5 mol% $B(C_6F_5)_3$ at 80 ℃ for 12 h. Purification by flash chromatography on silica gel using petroleum ether/EtOAc (10/1 to 5/1) afforded (*Z*)-**3av** as a white solid (101.9 mg, 82% yield, *Z*:*E* > 96:4).

(*Z*)-**3av**: $\mathbf{R}_f$ = 0.25 (petroleum ether/EtOAc = 5/1). **$^1$H NMR** (400 MHz, $CDCl_3$): δ 7.90-7.83 (m, 2H), 7.56 (t, *J* = 7.4 Hz, 1H), 7.47 (t, *J* = 7.6 Hz, 1H), 7.36 (d, *J* = 7.2 Hz, 1H), 7.28-7.16 (m, 10H), 7.09-7.04 (m, 3H), 6.97-6.92 (m, 3H), 6.89 (d, *J* = 8.4 Hz, 1H), 5.50 (s, 1H), 5.16 (s, 2H), 4.01 (t, *J* = 8.0 Hz, 2H), 3.48 (t, *J* = 8.0 Hz, 2H), 3.32 (s, 2H) ppm. **$^{13}$C NMR** (100 MHz, $CDCl_3$): δ 190.6, 170.6, 160.4, 155.7, 140.5, 140.3, 136.1, 136.0, 135.4, 134.8, 134.0, 132.8, 132.4, 129.6, 129.4, 129.2, 128.9, 128.1, 127.8, 127.6, 127.3, 126.6, 126.2, 125.0, 121.1, 73.5, 62.8, 53.9, 45.0, 39.0 ppm. **HRMS** (ESI) *m/z*: $[M+Na]^+$ calcd. for $C_{40}H_{31}NO_6Na$: 644.2049; Found: 644.2051.

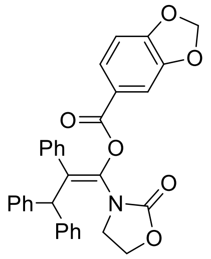

(*Z*)-**3aw**
$C_{32}H_{25}NO_6$
M = 519.55 g/mol

**(*Z*)-1-(2-oxooxazolidin-3-yl)-2,3,3-triphenylprop-1-en-1-yl benzo[*d*][1,3]dioxole-5-carboxylate** ((*Z*)-**3aw**): Prepared from 3-(phenylethynyl)oxazolidin-2-one (**1a**, 44.9 mg, 0.24 mmol) and

benzhydryl benzo[*d*][1,3]dioxole-5-carboxylate (**2w**, 66.5 mg, 0.2 mmol) according to the GP1 with 5 mol% $B(C_6F_5)_3$ at 80 ℃ for 24 h. Purification by flash chromatography on silica gel using petroleum ether/EtOAc (10/1 to 5/1) afforded (*Z*)-**3aw** as a white solid (91.0 mg, 88% yield, *Z*:*E* = 92:8).

(*Z*)-**3aw**: **R*f*** = 0.30 (petroleum ether/EtOAc = 5/1). **$^1$H NMR** (400 MHz, $CDCl_3$): δ 7.39-7.31 (m, 5H), 7.26 (t, *J* = 7.6 Hz, 4H), 7.23-7.15 (m, 3H), 7.04-6.09 (m, 5H), 6.70 (d, *J* = 8.0 Hz, 1H), 5.97 (s, 2H), 5.58 (s, 1H), 4.03 (t, *J* = 8.0 Hz, 2H), 3.58 (t, *J* = 8.0 Hz, 2H) ppm. **$^{13}$C NMR** (100 MHz, $CDCl_3$): δ 164.9, 155.8, 152.3, 147.6, 140.7, 136.1, 135.1, 133.7, 129.7, 129.0, 128.2, 127.5, 127.2, 126.6, 126.2, 121.9, 109.6, 108.0, 101.9, 62.8, 54.0, 45.3 ppm. **HRMS** (ESI) *m/z*: $[M+Na]^+$ calcd. for $C_{32}H_{25}NO_6Na$: 542.1580; Found: 542.1583.

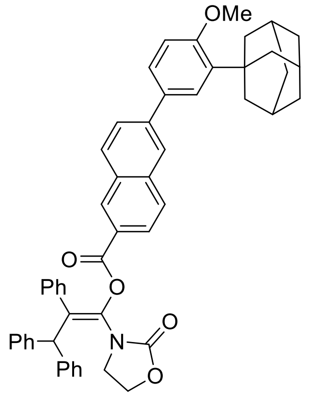


(*Z*)-**3ax**
$C_{52}H_{47}NO_5$
M = 765.95 g/mol

**(*Z*)-1-(2-oxooxazolidin-3-yl)-2,3,3-triphenylprop-1-en-1-yl 6-(3-(adamantan-1-yl)-4-methoxyphenyl)-2-naphthoate** ((*Z*)-**3ax**): Prepared from 3-(phenylethynyl)oxazolidin-2-one (**1a**, 44.9 mg, 0.24 mmol) and benzhydryl 6-(3-(adamantan-1-yl)-4-methoxyphenyl)-2-naphthoate (**2x**, 115.8 mg, 0.2 mmol) according to the GP1 with 20 mol% $B(C_6F_5)_3$ at 100 ℃ for 24 h. Purification by flash chromatography on silica gel using petroleum ether/EtOAc (10/1 to 5/1) afforded (*Z*)-**3ax** as a white solid (114.9 mg, 75% yield, *Z*:*E* = 95:5).

(*Z*)-**3ax**: **R*f*** = 0.25 (petroleum ether/EtOAc = 5/1). **$^1$H NMR** (400 MHz, $CDCl_3$): δ 8.29 (s, 1H), 7.93 (s, 1H), 7.85 (d, *J* = 8.4 Hz, 1H), 7.80-7.73 (m, 3H), 7.56 (d, *J* = 2.0 Hz,

1H), 7.50 (dd, *J* = 8.4, 2.4 Hz, 1H), 7.37 (d, *J* = 7.6 Hz, 4H), 7.30-7.19 (m, 6H), 7.10 (d, *J* = 7.2 Hz, 2H), 7.02-6.91 (m, 4H), 5.63 (s, 1H), 4.06 (t, *J* = 8.0 Hz, 2H), 3.89 (s, 3H), 3.65 (t, *J* = 8.0 Hz, 2H), 2.16 (s, 6H), 2.09 (s, 3H), 1.79 (s, 6H) ppm. **$^{13}$C NMR** (100 MHz, $CDCl_3$): δ 165.8, 159.0, 156.0, 141.8, 140.7, 139.0, 136.2, 135.2, 134.0, 132.3, 131.8, 130.9, 129.8, 129.7, 129.0, 128.2, 127.5, 127.2, 126.6, 126.56, 125.9, 125.7, 125.4, 124.8, 124.6, 112.1, 62.8, 55.1, 54.0, 45.3, 40.6, 37.2, 37.1, 29.1 ppm. **HRMS** (ESI) *m/z*: $[M+Na]^+$ calcd. for $C_{52}H_{47}NO_5Na$: 788.3352; Found: 788.3363.

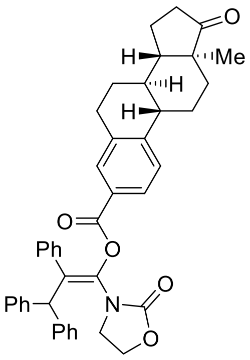


(*Z*)-**3ay**
$C_{43}H_{41}NO_5$
M = 651.80 g/mol

**(*Z*)-1-(2-oxooxazolidin-3-yl)-2,3,3-triphenylprop-1-en-1-yl (8*R*,9*S*,13*S*,14*S*)-13-methyl-17-oxo-7,8,9,11,12,13,14,15,16,17-decahydro-6*H*-cyclopenta[*a*]phenanthrene-3-carboxylate** ((*Z*)-**3ay**): Prepared from 3-(phenylethynyl)oxazolidin-2-one (**1a**, 74.9 mg, 0.4 mmol) and benzhydryl (8*R*,9*S*,13*S*,14*S*)-13-methyl-17-oxo-7,8,9,11,12,13,14,15,16,17-decahydro-6*H*-cyclopenta[a]phenanthrene-3-carboxylate (**2y**, 92.8 mg, 0.2 mmol) according to the GP1 with 10 mol% $B(C_6F_5)_3$ at 80 ℃ for 48 h. Purification by flash chromatography on silica gel using petroleum ether/EtOAc (10/1 to 5/1) afforded (*Z*)-**3ay** as a white solid (104.3 mg, 80% yield, *Z*:*E* = 89:11).

(*Z*)-**3ay**: **$R_f$** = 0.25 (petroleum ether/EtOAc = 5/1). **$^1$H NMR** (400 MHz, $CDCl_3$): δ 7.53 (d, *J* = 8.0 Hz, 1H), 7.48 (s, 1H), 7.34 (d, *J* = 7.6 Hz, 4H), 7.29-7.17 (m, 7H), 7.07-6.97 (m, 5H), 5.59 (s, 1H), 4.02 (t, *J* = 8.0 Hz, 2H), 3.58 (t, *J* = 8.0 Hz, 2H), 2.86-2.81 (m, 2H), 2.50 (dd, *J* = 18.8, 8.6 Hz, 1H), 2.41-2.34 (m, 1H), 2.29-2.25 (m, 1H), 2.15-1.93 (m, 4H), 1.63-1.37 (m, 6H), 0.88 (s, 3H) ppm. **$^{13}$C NMR** (100 MHz, $CDCl_3$): δ 165.6, 155.9, 146.1, 140.7, 136.8, 136.1, 135.0, 133.8, 130.6, 129.7, 129.0, 128.1,

127.4, 127.2, 127.1, 126.6, 125.5, 125.4, 62.8, 54.0, 50.4, 47.8, 45.3, 44.6, 37.6, 35.7, 31.4, 23.0, 26.1, 25.4, 21.5, 13.7 ppm. **HRMS** (ESI) *m/z*: $[M+Na]^+$ calcd. for $C_{43}H_{41}NO_5Na$: 594.0692; Found: 594.0697.

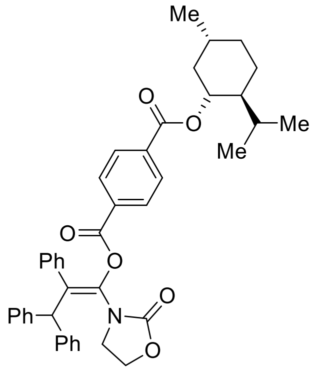


(*Z*)-**3az**
$C_{42}H_{43}NO_6$
M = 657.81 g/mol

**(1*R*,2*S*,5*R*)-2-isopropyl-5-methylcyclohexyl ((*Z*)-1-(2-oxooxazolidin-3-yl)-2,3,3-triphenylprop-1-en-1-yl) terephthalate** ((*Z*)-**3az**): Prepared from 3-(phenylethynyl)oxazolidin-2-one (**1a**, 44.9 mg, 0.24 mmol) and benzhydryl ((1*R*,2*S*,5*R*)-2-isopropyl-5-methylcyclohexyl) terephthalate (**2z**, 94.1 mg, 0.2 mmol) according to the GP1 with 10 mol% $B(C_6F_5)_3$ at 80 °C for 24 h. Purification by flash chromatography on silica gel using petroleum ether/EtOAc (10/1 to 5/1) afforded (*Z*)-**3az** as a white solid (98.6 mg, 75% yield, *Z*:*E* = 95:5).

(*Z*)-**3az**: $\mathbf{R_f}$ = 0.35 (petroleum ether/EtOAc = 5/1). **$^1$H NMR** (400 MHz, $CDCl_3$): δ 7.96 (d, *J* = 8.0 Hz, 2H), 7.79 (d, *J* = 8.4 Hz, 2H), 7.34 (d, *J* = 8.0 Hz, 4H), 7.28 (t, *J* = 7.0 Hz, 4H), 7.21 (d, *J* = 6.8 Hz, 2H), 7.06-6.96 (m, 5H), 5.59 (s, 1H), 4.95-4.88 (m, 1H), 4.04 (t, *J* = 8.0 Hz, 2H), 3.59 (t, *J* = 8.0 Hz, 2H), 2.08 (d, *J* = 12.0 Hz, 1H), 1.92-1.85 (m, 1H), 1.72 (d, *J* = 11.6 Hz, 2H), 1.53 (t, *J* = 11.4 Hz, 2H), 1.17-1.03 (m, 2H), 0.91 (t, *J* = 7.0 Hz, 7H), 0.76 (d, *J* = 6.8 Hz, 3H) ppm. **$^{13}$C NMR** (100 MHz, $CDCl_3$): δ 165.0, 164.9, 155.8, 140.5, 136.0, 135.2, 135.0, 134.2, 131.7, 129.8, 129.6, 129.4, 128.9, 128.2, 127.6, 127.3, 126.7, 75.5, 62.8, 54.0, 47.1, 45.2, 40.8, 34.2, 31.4, 26.4, 23.5, 22.0, 20.7, 16.4 ppm. **HRMS** (ESI) *m/z*: $[M+Na]^+$ calcd. for $C_{42}H_{43}NO_6Na$: 680.2988; Found: 680.2996.

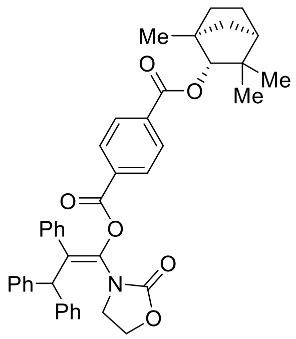


($Z$)-**3aaa**
$C_{42}H_{41}NO_6$
M = 655.79 g/mol

**(*Z*)-1-(2-oxooxazolidin-3-yl)-2,3,3-triphenylprop-1-en-1-yl((1*S*,2*R*,4*R*)-1,3,3-trimethylbicyclo [2.2.1]heptan-2-yl) terephthalate** (($Z$)-**3aaa**): Prepared from 3-(phenylethynyl)oxazolidin-2-one (**1a**, 44.9 mg, 0.24 mmol) and benzhydryl ((1*S*,2*R*,4*R*)-1,3,3-trimethylbicyclo[2.2.1]heptan-2-yl) terephthalate (**2aa**, 93.7 mg, 0.2 mmol) according to the GP1 with 5 mol% $B(C_6F_5)_3$ at 80 ℃ for 36 h. Purification by flash chromatography on silica gel using petroleum ether/EtOAc (10/1 to 5/1) afforded ($Z$)-**3aaa** as a white solid (91.8 mg, 70% yield, $Z$:$E$ = 93:7).

($Z$)-**3aaa**: $\mathbf{R_f}$ = 0.35 (petroleum ether/EtOAc = 5/1). **$^1$H NMR** (400 MHz, $CDCl_3$): δ 7.98 (d, $J$ = 8.4 Hz, 2H), 7.81 (d, $J$ = 8.4 Hz, 2H), 7.34 (d, $J$ = 7.6 Hz, 4H), 7.30-7.25 (m, 4H), 7.22 (d, $J$ = 7.2 Hz, 2H), 7.06-6.97 (m, 5H), 5.59 (s, 1H), 4.59 (s, 1H), 4.04 (t, $J$ = 8.0 Hz, 2H), 3.59 (t, $J$ = 8.0 Hz, 2H), 1.91-1.83 (m, 1H), 1.78-1.73 (dm, 2H), 1.64 (s, 1H), 1.56-1.47 (m, 1H), 1.27-1.18 (m, 2H), 1.16 (s, 3H), 1.08 (s, 3H), 0.80 (s, 3H) ppm. **$^{13}$C NMR** (100 MHz, $CDCl_3$): δ 165.8, 164.8, 155.7, 140.5, 136.0, 135.0, 134.97, 134.2, 131.7, 129.9, 129.6, 129.4, 128.8, 128.2, 127.6, 127.3, 126.7, 87.3, 62.8, 54.0, 48.6, 48.3, 45.2, 41.4, 39.8, 29.7, 26.8, 25.8, 20.2, 19.4 ppm. **HRMS** (ESI) *m/z*: $[M+Na]^+$ calcd. for $C_{42}H_{41}NO_6Na$: 678.2832; Found: 678.2836.

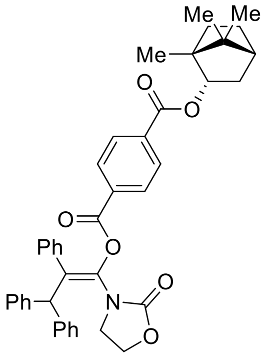


(*Z*)-**3aab**
$C_{42}H_{41}NO_6$
M = 655.79 g/mol

**(*Z*)-1-(2-oxooxazolidin-3-yl)-2,3,3-triphenylprop-1-en-1-yl-((1*R*,2*S*,4*R*)-1,7,7-trimethylbicyclo [2.2.1]heptan-2-yl) terephthalate** ((*Z*)-**3aab**): Prepared from 3-(phenylethynyl)oxazolidin-2-one (**1a**, 44.9 mg, 0.24 mmol) and benzhydryl ((1*R*,2*S*,4*R*)-1,7,7-trimethylbicyclo[2.2.1]heptan-2-yl) terephthalate (**2ab**, 93.7 mg, 0.2 mmol) according to the GP1 with 5 mol% $B(C_6F_5)_3$ at 80 ℃ for 48 h. Purification by flash chromatography on silica gel using petroleum ether/EtOAc (10/1 to 5/1) afforded (*Z*)-**3aab** as a white solid (91.8 mg, 70% yield, *Z*:*E* = 95:5).

(*Z*)-**3aab**: **$R_f$** = 0.35 (petroleum ether/EtOAc = 10/1). **$^1$H NMR** (400 MHz, $CDCl_3$): δ 7.97 (d, *J* = 8.4 Hz, 2H), 7.80 (d, *J* = 8.4 Hz, 2H), 7.34 (d, *J* = 7.6 Hz, 4H), 7.27 (t, *J* = 7.4 Hz, 4H), 7.21 (d, *J* = 7.2 Hz, 2H), 7.06-6.96 (m, 5H), 5.59 (s, 1H), 5.09 (d, *J* = 9.2 Hz, 1H), 4.04 (t, *J* = 8.0 Hz, 2H), 3.60 (t, *J* = 8.0 Hz, 2H), 2.50-2.42 (m, 1H), 2.09-2.02 (m, 1H), 1.87-1.70 (m, 2H), 1.43-1.36 (m, 1H), 1.32-1.25 (m, 1H), 1.08 (dd, *J* = 13.8, 3.4 Hz, 1H), 0.95 (s, 3H), 0.91 (s, 3H), 0.89 (s, 3H) ppm. **$^{13}$C NMR** (100 MHz, $CDCl_3$): δ 165.7, 164.8, 155.8, 140.5, 136.0, 135.2, 135.0, 134.2, 131.7, 129.8, 129.6, 129.3, 128.8, 128.2, 127.5, 127.3, 126.7, 81.2, 62.8, 54.0, 49.0, 47.9, 45.2, 44.9, 36.8, 28.0, 27.3, 19.6, 18.8, 13.6. ppm. **HRMS** (ESI) *m/z*: $[M+Na]^+$ calcd. for $C_{42}H_{41}NO_6Na$: 678.2832; Found: 678.2857.

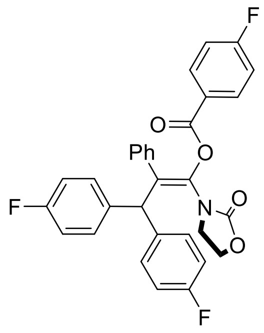

(*Z*)-**3aac**
$C_{31}H_{22}F_3NO_4$
M = 529.52 g/mol

**(*Z*)-3,3-bis(4-fluorophenyl)-1-(2-oxooxazolidin-3-yl)-2-phenylprop-1-en-1-yl 4-fluorobenzoate** ((*Z*)-**3aac**): Prepared from 3-(phenylethynyl)oxazolidin-2-one (**1a**, 44.9 mg, 0.24 mmol) and bis(4-fluorophenyl)methyl 4-fluorobenzoate (**2ac**, 68.5 mg, 0.2 mmol) according to the GP1 with 5 mol% $B(C_6F_5)_3$ at 80 ℃ for 24 h. Purification by flash chromatography on silica gel using petroleum ether/EtOAc (10/1 to 5/1) afforded (*Z*)-**3aac** as a white solid (86.8 mg, 82% yield, *Z*:*E* = 91:9).

(*Z*)-**3aac**: $\mathbf{R_f}$ = 0.35 (petroleum ether/EtOAc = 10/1). **$^1$H NMR** (400 MHz, $CDCl_3$): δ 7.75 (dd, *J* = 8.8, 5.4 Hz, 2H), 7.31-7.25 (m, 4H), 7.06-6.91 (m, 11H), 5.56 (s, 1H), 4.16 (t, *J* = 8.0 Hz, 2H), 3.64 (t, *J* = 8.0 Hz, 2H) ppm. **$^{13}$C NMR** (100 MHz, $CDCl_3$): δ 166.2 (d, *J* = 254.3 Hz), 164.6, 161.6 (d, *J* = 244.5 Hz), 155.9, 136.2 (d, *J* = 3.2 Hz), 135.4, 135.1, 133.5, 132.6 (d, *J* = 9.5 Hz), 131.1 (d, *J* = 7.8 Hz), 128.9, 127.7, 127.5, 124.2 (d, *J* = 3.0 Hz), 115.7 (d, *J* = 22.0 Hz) 115.1 (d, *J* = 21.1 Hz), 62.8, 52.1, 45.0 ppm. **$^{19}$F NMR** (376 MHz, $CDCl_3$): δ -103.53, -115.82 ppm. **HRMS** (ESI) *m/z*: $[M+Na]^+$ calcd. for $C_{31}H_{22}F_3NO_4Na$: 552.1399; Found: 552.1404.

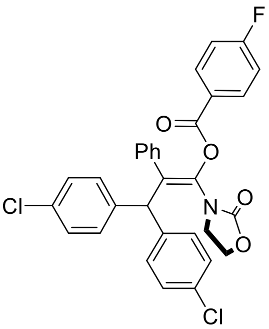

(*Z*)-**3aad**
$C_{31}H_{22}Cl_2FNO_4$
M = 562.42 g/mol

**(*Z*)-3,3-bis(4-chlorophenyl)-1-(2-oxooxazolidin-3-yl)-2-phenylprop-1-en-1-yl 4-fluorobenzoate** ((*Z*)-**3aad**): Prepared from 3-(phenylethynyl)oxazolidin-2-one (**1a**, 44.9 mg, 0.24 mmol) and bis(4-chlorophenyl)methyl 4-fluorobenzoate (**2ad**, 75.0 mg, 0.2 mmol) according to the GP1 with 5 mol% $B(C_6F_5)_3$ at 80 ℃ for 40 h. Purification by flash chromatography on silica gel using petroleum ether/EtOAc (10/1 to 5/1) afforded (*Z*)-**3aad** as a white solid (67.5 mg, 60% yield, *Z*:*E* = 88:12).

(*Z*)-**3aad**: **R*f*** = 0.35 (petroleum ether/EtOAc = 5/1). **$^1$H NMR** (400 MHz, $CDCl_3$): δ 7.75 (dd, *J* = 8.8, 5.4 Hz, 2H), 7.26-7.22 (m, 8H), 7.05-6.96 (m, 7H), 5.54 (s, 1H), 4.14 (t, *J* = 8.0 Hz, 2H), 3.62 (t, *J* = 8.0 Hz, 2H) ppm. **$^{13}$C NMR** (100 MHz, $CDCl_3$): δ 166.2 (d, *J* = 254.4 Hz), 164.5, 155.8, 138.8, 135.3, 135.26, 132.9, 132.7, 132.6 (d, *J* = 9.6 Hz), 130.9, 128.9, 128.4, 127.7, 127.6, 124.1 (d, *J* = 2.9 Hz), 115.7 (d, *J* = 22.0 Hz), 62.8, 52.4, 44.9 ppm. **$^{19}$F NMR** (376 MHz, $CDCl_3$): δ -103.46 ppm. **HRMS** (ESI) *m/z*: $[M+Na]^+$ calcd. for $C_{31}H_{22}Cl_2FNO_4Na$: 584.0808; Found: 584.0813.

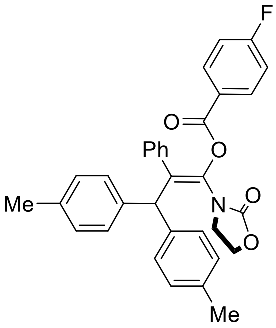


(*Z*)-**3aae**
$C_{33}H_{28}FNO_4$
M = 521.59 g/mol

**(*Z*)-1-(2-oxooxazolidin-3-yl)-2-phenyl-3,3-di-p-tolylprop-1-en-1-yl 4-fluorobenzoate** ((*Z*)-**3aae**): Prepared from 3-(phenylethynyl)oxazolidin-2-one (**1a**, 44.9 mg, 0.24 mmol) and di-*p*-tolylmethyl 4-fluorobenzoate (**2ae**, 66.9 mg, 0.2 mmol) according to the GP1 with 5 mol% $B(C_6F_5)_3$ at 80 ℃ for 12 h. Purification by flash chromatography on silica gel using petroleum ether/EtOAc (10/1 to 5/1) afforded (*Z*)-**3aae** as a white solid (79.4 mg, 76% yield, *Z*:*E* = 94:6).

(*Z*)-**3aae**: **R*f*** = 0.30 (petroleum ether/EtOAc = 5/1). **$^1$H NMR** (400 MHz, $CDCl_3$): δ 7.75 (dd, *J* = 8.4, 5.6 Hz, 2H), 7.19 (d, *J* = 8.0 Hz, 4H), 7.07 (d, *J* = 7.6 Hz, 4H), 7.03-

6.94 (m, 7H), 5.48 (s, 1H), 4.06 (t, *J* = 8.0 Hz, 2H), 3.65 (t, *J* = 8.0 Hz, 2H), 2.30 (s, 6H) ppm. **$^{13}$C NMR** (100 MHz, $CDCl_3$): δ 166.1 (d, *J* = 253.9 Hz), 164.6, 155.9, 137.6, 136.2, 136.1, 134.9, 134.2, 132.6 (d, *J* = 9.4 Hz), 129.5, 129.0, 128.8, 127.5, 127.2, 124.5 (d, *J* = 2.9 Hz), 115.5 (d, *J* = 22.0 Hz), 62.8, 53.3, 45.6, 21.0 ppm. **$^{19}$F NMR** (376 MHz, $CDCl_3$): δ -103.99 ppm. **HRMS** (ESI) *m/z*: $[M+Na]^+$ calcd. for $C_{33}H_{28}FNO_4Na$: 544.1902; Found: 544.1902.

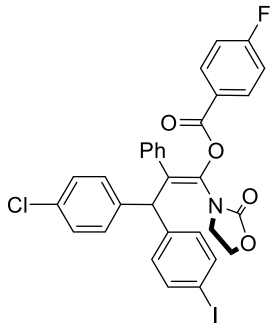


(*Z*)-**3aaf**
$C_{31}H_{22}ClFINO_4$
M = 653.87 g/mol

**(*Z*)-3-(4-chlorophenyl)-3-(4-iodophenyl)-1-(2-oxooxazolidin-3-yl)-2-phenylprop-1-en-1-yl 4-fluorobenzoate** ((*Z*)-**3aaf**): Prepared from 3-(phenylethynyl)oxazolidin-2-one (**1a**, 44.9 mg, 0.24 mmol) and (4-chlorophenyl)(4-iodophenyl)methyl 4-fluorobenzoate (**2af**, 93.3 mg, 0.2 mmol) according to the GP1 with 10 mol% $B(C_6F_5)_3$ at 80 ℃ for 12 h. Purification by flash chromatography on silica gel using petroleum ether/EtOAc (10/1 to 5/1) afforded (*Z*)-**3aaf** as a white solid (85.8 mg, 66% yield, *Z*:*E* = 89:11).

(*Z*)-**3aaf**: **$R_f$** = 0.30 (petroleum ether/EtOAc = 5/1). **$^{1}$H NMR** (400 MHz, $CDCl_3$): δ 7.75 (dd, *J* = 8.8, 5.2 Hz, 2H), 7.60 (d, *J* = 8.0 Hz, 2H), 7.23 (s, 4H), 7.07 (d, *J* = 8.4 Hz, 2H), 7.05-6.95 (m, 7H), 5.51 (s, 1H), 4.13 (t, *J* = 8.0 Hz, 2H), 3.61 (t, *J* = 8.0 Hz, 2H) ppm. **$^{13}$C NMR** (100 MHz, $CDCl_3$): δ 166.2 (d, *J* = 254.4 Hz), 164.4, 155.8, 140.0, 138.6, 137.3, 135.3, 135.2, 132.8, 132.7, 132.6 (d, *J* = 9.5 Hz), 131.6, 131.0, 128.9, 128.4, 127.7, 127.6, 124.1 (d, *J* = 2.9 Hz), 115.7 (d, *J* = 22.0 Hz), 92.4, 62.8, 52.6, 44.9 ppm. **$^{19}$F NMR** (376 MHz, $CDCl_3$): δ -103.43 ppm. **HRMS** (ESI) *m/z*: $[M+Na]^+$ calcd. for $C_{31}H_{21}ClFINO_4Na$: 676.0164; Found: 676.0171.

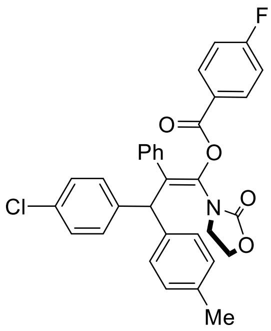

(*Z*)-**3aag**
$C_{32}H_{25}ClFNO_4$
M = 542.00 g/mol

**(Z)-3-(4-chlorophenyl)-1-(2-oxooxazolidin-3-yl)-2-phenyl-3-(p-tolyl)prop-1-en-1-yl 4-fluorobenzoate** ((*Z*)-**3aag**): Prepared from 3-(phenylethynyl)oxazolidin-2-one (**1a**, 44.9 mg, 0.24 mmol) and (4-chlorophenyl)(p-tolyl)methyl 4-fluorobenzoate (**2ag**, 71.0 mg, 0.2 mmol) according to the GP1 with 5 mol% $B(C_6F_5)_3$ at 80 ℃ for 48 h. Purification by flash chromatography on silica gel using petroleum ether/EtOAc (10/1 to 5/1) afforded (*Z*)-**3aag** as a white solid (80.0 mg, 73% yield, *Z*:*E* = 94:6).

(*Z*)-**3aag**: **R*f*** = 0.30 (petroleum ether/EtOAc = 5/1). **$^{1}$H NMR** (400 MHz, $CDCl_3$): δ 7.75 (dd, *J* = 9.0, 5.4 Hz, 2H), 7.24-7.18 (m, 6H), 7.10 (d, *J* = 8.0 Hz, 2H), 7.04-6.94 (m, 7H), 5.51 (s, 1H), 4.09 (t, *J* = 7.6 Hz, 2H), 3.62 (t, *J* = 7.6 Hz, 2H), 2.32 (s, 3H). ppm. **$^{13}$C NMR** (100 MHz, $CDCl_3$): δ 166.1 (d, *J* = 254.2 Hz), 164.5, 155.9, 139.2, 137.2, 136.5, 135.7, 135.1, 133.6, 132.6 (d, *J* = 9.5 Hz), 132.4, 131.1, 129.3, 129.0, 128.9, 128.2, 127.6, 127.4, 124.3 (d, *J* = 2.9 Hz), 115.6 (d, *J* = 22.0 Hz), 62.8, 52.9, 45.2, 21.0 ppm. **$^{19}$F NMR** (376 MHz, $CDCl_3$): δ -103.71 ppm. **HRMS** (ESI) *m/z*: $[M+Na]^+$ calcd. for $C_{32}H_{25}ClFNO_4Na$: 564.1354; Found: 564.1358.

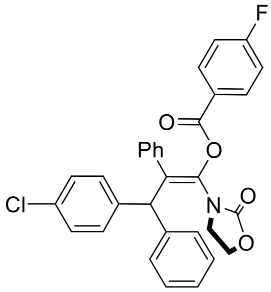

(*Z*)-**3aah**
$C_{31}H_{23}ClFNO_4$
M = 527.98 g/mol

**(*Z*)-3-(4-chlorophenyl)-1-(2-oxooxazolidin-3-yl)-2,3-diphenylprop-1-en-1-yl 4-fluorobenzoate** ((*Z*)-**3aah**): Prepared from 3-(phenylethynyl)oxazolidin-2-one (**1a**, 44.9 mg, 0.24 mmol) and (4-chlorophenyl)(phenyl)methyl 4-fluorobenzoate (**2ah**, 68.2 mg, 0.2 mmol) according to the GP1 with 5 mol% $B(C_6F_5)_3$ at 80 ℃ for 12 h. Purification by flash chromatography on silica gel using petroleum ether/EtOAc (10/1 to 5/1) afforded (*Z*)-**3aah** as a white solid (80.0 mg, 73% yield, *Z*:*E* = 94:6).

(*Z*)-**3aah**: $\mathbf{R_f}$ = 0.35 (petroleum ether/EtOAc = 5/1). $^1$**H NMR** (400 MHz, $CDCl_3$): δ 7.75 (dd, *J* = 8.6, 5.4 Hz, 2H), 7.34-7.20 (m, 9H), 7.05-6.96 (m, 7H), 5.56 (s, 1H), 4.08 (t, *J* = 8.0 Hz, 2H), 3.59 (t, *J* = 8.0 Hz, 2H) ppm. $^{13}$**C NMR** (100 MHz, $CDCl_3$): δ 166.1 (d, *J* = 254.1 Hz), 164.5, 155.8, 140.4, 139.0, 135.7,135.2, 133.5, 132.6 (d, *J* = 9.5 Hz), 132.5, 131.2, 129.4, 128.9, 128.3, 128.27, 127.6, 127.4, 126.9, 124.3 (d, *J* = 2.9 Hz), 115.6 (d, *J* = 22.0 Hz), 62.8, 53.2, 45.1 ppm. $^{19}$**F NMR** (376 MHz, $CDCl_3$): δ -103.66 ppm. **HRMS** (ESI) *m/z*: $[M+Na]^+$ calcd. for $C_{31}H_{23}ClFNO_4Na$: 550.1198; Found: 550.1204.

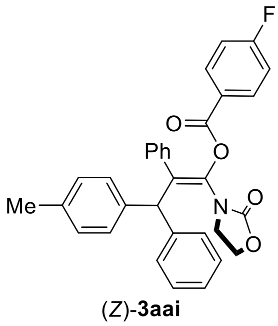


(*Z*)-**3aai**
$C_{32}H_{26}FNO_4$
M = 507.56 g/mol

**(*Z*)-1-(2-oxooxazolidin-3-yl)-2,3-diphenyl-3-(p-tolyl)prop-1-en-1-yl 4-fluorobenzoate** ((*Z*)-**3aai**): Prepared from 3-(phenylethynyl)oxazolidin-2-one (**1a**, 44.9 mg, 0.24 mmol) and phenyl(*p*-tolyl)methyl 4-fluorobenzoate (**2ai**, 64.1 mg, 0.2 mmol) according to the GP1 with 5 mol% $B(C_6F_5)_3$ at 80 ℃ for 48 h. Purification by flash chromatography on silica gel using petroleum ether/EtOAc (10/1 to 5/1) afforded (*Z*)-**3aai** as a white solid (86.3 mg, 85% yield, *Z*:*E* > 96:4).

(*Z*)-**3aai**: $\mathbf{R_f}$ = 0.30 (petroleum ether/EtOAc = 5/1). $^1$**H NMR** (400 MHz, $CDCl_3$): δ 7.75 (dd, *J* = 8.4, 5.6 Hz, 2H), 7.31 (d, *J* = 7.6 Hz, 2H), 7.27-7.18 (m, 5H), 7.08 (d, *J* = 8.0

Hz, 2H), 7.03-6.93 (m, 7H), 5.53 (s, 1H), 4.04 (t, *J* = 8.0 Hz, 2H), 3.61 (t, *J* = 8.0 Hz, 2H), 2.30 (s, 3H) ppm. **$^{13}$C NMR** (100 MHz, $CDCl_3$): δ 166.0 (d, *J* = 254.0 Hz), 164.6, 155.8, 140.8, 137.3, 136.2, 136.1, 135.0, 134.1, 132.6 (d, *J* = 8.9 Hz), 129.6, 129.5, 128.9, 128.86, 128.1, 127.5, 127.2, 126.6, 124.4 (d, *J* = 2.9 Hz), 115.5 (d, *J* = 22.0 Hz), 62.8, 53.6, 45.4, 21.0 ppm. **$^{19}$F NMR** (376 MHz, $CDCl_3$): δ -103.91 ppm. **HRMS** (ESI) *m/z*: $[M+Na]^+$ calcd. for $C_{32}H_{26}FNO_4Na$: 530.1744; Found: 530.1754.

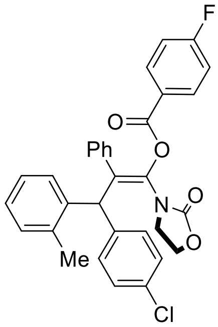


(*Z*)-**3aaj**
$C_{32}H_{25}ClFNO_4$
M = 542.00 g/mol

**(*Z*)-3-(4-chlorophenyl)-1-(2-oxooxazolidin-3-yl)-2-phenyl-3-(*o*-tolyl)prop-1-en-1-yl 4-fluorobenzoate** ((*Z*)-**3aaj**): Prepared from 3-(phenylethynyl)oxazolidin-2-one (**1a**, 44.9 mg, 0.24 mmol) and (4-chlorophenyl)(*o*-tolyl)methyl 4-fluorobenzoate (**2aj**, 71.0 mg, 0.2 mmol) according to the GP1 with 5 mol% $B(C_6F_5)_3$ at 80 ℃ for 36 h. Purification by flash chromatography on silica gel using petroleum ether/EtOAc (10/1 to 5/1) afforded (*Z*)-**3aaj** as a white solid (78.0 mg, 72% yield, *Z*:*E* > 96:4).

(*Z*)-**3aaj**: **$R_f$** = 0.35 (petroleum ether/EtOAc = 5/1). **$^1$H NMR** (400 MHz, $CDCl_3$): δ 7.76 (dd, *J* = 8.6, 5.4 Hz, 2H), 7.36 (d, *J* = 8.0 Hz, 2H), 7.26-7.19 (m, 5H), 7.17-7.11 (m, 3H), 7.05 (t, *J* = 7.4Hz, 2H), 7.00-6.95 (m, 3H), 5.59 (s, 1H), 3.84 (q, *J* = 8.8 Hz, 1H), 3.70 (q, *J* = 8.4 Hz, 1H), 3.32 (q, *J* = 8.4 Hz, 1H), 3.18 (q, *J* = 8.8 Hz, 1H), 2.45 (s, 3H) ppm. **$^{13}$C NMR** (100 MHz, $CDCl_3$): δ 166.1 (d, *J* = 254.2 Hz), 164.3, 155.7, 139.6, 139.0, 136.8, 136.7, 135.2, 133.5, 132.6 (d, *J* = 9.5 Hz), 132.4, 131.3, 130.3, 129.5, 128.6, 128.4, 127.7, 127.4, 126.8, 125.9, 124.2 (d, *J* = 2.9 Hz), 115.6 (d, *J* = 22.0 Hz), 62.7, 51.4, 44.4, 19.9 ppm. **$^{19}$F NMR** (376 MHz, $CDCl_3$): δ -103.69 ppm. **HRMS** (ESI) *m/z*: $[M+Na]^+$ calcd. for $C_{32}H_{25}ClFNO_4Na$: 564.1354; Found: 564.1360.

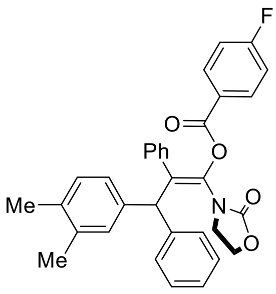

(*Z*)-**3aak**
$C_{33}H_{28}FNO_4$
M = 521.59 g/mol

**(*Z*)-3-(3,4-dimethylphenyl)-1-(2-oxooxazolidin-3-yl)-2,3-diphenylprop-1-en-1-yl 4-fluorobenzoate** ((*Z*)-**3aak**): Prepared from 3-(phenylethynyl)oxazolidin-2-one (**1a**, 44.9 mg, 0.24 mmol) and (3,4-dimethylphenyl)(phenyl)methyl 4-fluorobenzoate (**2ak**, 66.9 mg, 0.2 mmol) according to the GP1 with 5 mol% $B(C_6F_5)_3$ at 80 ℃ for 12 h. Purification by flash chromatography on silica gel using petroleum ether/EtOAc (10/1 to 5/1) afforded (*Z*)-**3aak** as a white solid (76.1 mg, 73% yield, *Z*:*E* = 92:8).

(*Z*)-**3aak**: **R*f*** = 0.35 (petroleum ether/EtOAc = 5/1). **$^1$H NMR** (400 MHz, $CDCl_3$): δ 7.79-7.72 (m, 2H), 7.30 (d, *J* = 7.6 Hz, 2H), 7.25 (t, *J* = 7.4 Hz, 2H), 7.19 (d, *J* = 7.2 Hz, 1H), 7.10-6.94 (m, 10H), 5.49 (s, 1H), 4.03 (t, *J* = 8.0 Hz, 2H), 3.62 (t, *J* = 8.0 Hz, 2H), 2.22 (s, 3H), 2.20 (s, 3H) ppm. **$^{13}$C NMR** (100 MHz, $CDCl_3$): δ 166.0 (d, *J* = 253.9 Hz), 164.7, 155.9, 140.8, 137.7, 136.3, 136,26, 134,9, 134.85, 134.2, 132.6 (d, *J* = 9.5 Hz), 130.9, 129.7, 129.4, 128.9, 128.1, 127.4, 127.2, 126.9, 126.5, 124.5 (d, *J* = 2.9 Hz), 115.5 (d, *J* = 22.0 Hz), 62.8, 53.7, 45.5, 19.7, 19.3 ppm. **$^{19}$F NMR** (376 MHz, $CDCl_3$): δ -103.97 ppm. **HRMS** (ESI) *m/z*: $[M+Na]^+$ calcd. for $C_{32}H_{28}FNO_4Na$: 544.1900; Found: 544.1902.

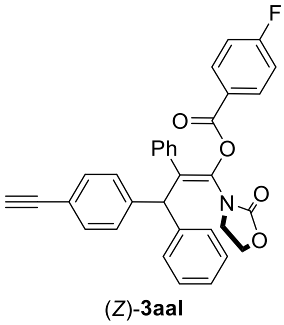

(*Z*)-**3aal**
$C_{33}H_{24}FNO_4$
M = 517.56 g/mol

**(*Z*)-3-(4-ethynylphenyl)-1-(2-oxooxazolidin-3-yl)-2,3-diphenylprop-1-en-1-yl 4-fluorobenzoate** ((*Z*)-**3aal**): Prepared from 3-(phenylethynyl)oxazolidin-2-one (**1a**, 44.9 mg, 0.24 mmol) and (4-ethynylphenyl)(phenyl)methyl 4-fluorobenzoate (**2al**, 66.1 mg, 0.2 mmol) according to the GP1 with 5 mol% $B(C_6F_5)_3$ at 80 ℃ for 48 h. Purification by flash chromatography on silica gel using petroleum ether/EtOAc (10/1 to 5/1) afforded (*Z*)-**3aal** as a white solid (63.1 mg, 61% yield, *Z*:*E* = 90:10).

(*Z*)-**3aal**: **R*f*** = 0.30 (petroleum ether/EtOAc = 5/1). **$^1$H NMR** (400 MHz, $CDCl_3$): δ 7.78 -7.73 (m, 2H), 7.38 (d, *J* = 8.4 Hz, 2H), 7.33-7.26 (m, 5H), 7.24-7.13 (m, 2H), 7.03-6.95 (m, 7H), 5.58 (s, 1H), 4.06 (t, *J* = 8.0 Hz, 2H), 3.58 (t, *J* = 8.0 Hz, 2H), 3.04 (s, 1H) ppm. **$^{13}$C NMR** (100 MHz, $CDCl_3$): δ 166.1 (d, *J* = 254.3 Hz), 164.5, 155.8, 141.5, 140.2, 135.7, 135.2, 133.5, 132.6 (d, *J* = 9.5 Hz), 131.9, 129.7, 129.5, 128.9, 128.3, 127.6, 127.4, 126.8, 124.3 (d, *J* = 3.0 Hz), 120.4, 115.6 (d, *J* = 22.0 Hz), 83.5, 77.2, 62.8, 53.7, 45.1 ppm. **$^{19}$F NMR** (376 MHz, $CDCl_3$): δ -103.71 ppm (The desired product containing a trace amount hydroacyloxylation side product **4aa**). **HRMS** (ESI) *m/z*: $[M+Na]^+$ calcd. for $C_{33}H_{24}FNO_4Na$: 540.1587; Found: 540.1589.

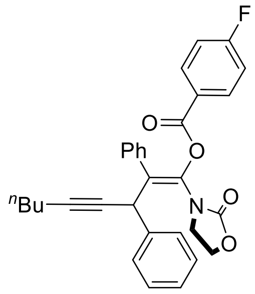


(*Z*)-**3aam**
$C_{31}H_{28}FNO_4$
M = 497.57 g/mol

**(*Z*)-1-(2-oxooxazolidin-3-yl)-2,3-diphenylnon-1-en-4-yn-1-yl 4-fluorobenzoate** ((*Z*)-**3aam**): Prepared from 3-(phenylethynyl)oxazolidin-2-one (**1a**, 44.9 mg, 0.24 mmol) and 1-phenylhept-2-yn-1-yl 4-fluorobenzoate (**2am**, 62.1 mg, 0.2 mmol) according to the GP1 with 5 mol% $B(C_6F_5)_3$ at 80 ℃ for 24 h. Purification by flash chromatography on silica gel using petroleum ether/EtOAc (10/1 to 5/1) afforded (*Z*)-**3aam** as a colorless oil (64.7 mg, 65% yield, *Z*:*E* = 71:29).

(*Z*)-**3aam**: **R*f*** = 0.40 (petroleum ether/EtOAc = 5/1). **$^{1}$H NMR** (400 MHz, $CDCl_3$): δ 8.18 (dd, *J* = 8.4, 5.6 Hz, 2H), 7.38 (d, *J* = 7.2 Hz, 2H), 7.29-7.14 (m, 8H), 7.13-7.07 (m, 2H), 5.18 (s, 1H), 4.08-3.98 (m, 2H), 3.54 (q, *J* = 8.4 Hz, 1H), 3.40 (q, *J* = 8.4 Hz, 1H), 2.15 (td, *J* = 7.2, 2.4 Hz, 2H), 1.44-1.37 (m, 2H), 1.34-1.26 (m, 2H), 0.86 (t, *J* = 7.2 Hz, 3H) ppm. **$^{13}$C NMR** (100 MHz, $CDCl_3$): δ 166.5 (d, *J* = 254.5 Hz), 164.0, 155.9, 138.0, 135.3, 134.4, 133.2 (d, *J* = 9.5 Hz), 129.8, 129.2, 128.3, 128.1, 128.0, 127.9, 126.9, 124.7 (d, *J* = 2.9 Hz), 115.9 (d, *J* = 22.0 Hz), 86.3, 77.2, 63.0, 45.9, 39.0, 30.7, 21.9, 18.5, 13.6 ppm. **$^{19}$F NMR** (376 MHz, $CDCl_3$): δ -103.38 ppm. **HRMS** (ESI) *m/z*: $[M+Na]^+$ calcd. for $C_{31}H_{28}FNO_4Na$: 520.1900; Found: 520.1912.

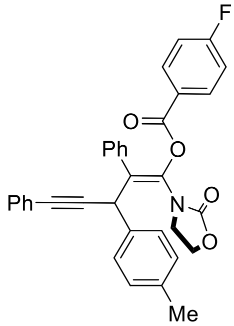


(*Z*)-**3aan**
$C_{34}H_{26}FNO_4$
M = 531.58 g/mol

**(*Z*)-1-(2-oxooxazolidin-3-yl)-2,5-diphenyl-3-(*p*-tolyl)pent-1-en-4-yn-1-yl 4-fluorobenzoate** ((*Z*)-**3aan**): Prepared from 3-(phenylethynyl)oxazolidin-2-one (**1a**, 44.9 mg, 0.24 mmol) and 3-phenyl-1-(*p*-tolyl)prop-2-yn-1-yl 4-fluorobenzoate (**2an**, 68.9 mg, 0.2 mmol) according to the GP1 with 5 mol% $B(C_6F_5)_3$ at 80 ℃ for 12 h. Purification by flash chromatography on silica gel using petroleum ether/EtOAc (10/1 to 5/1) afforded (*Z*)-**3aan** as a white solid (65.9 mg, 62% yield, *Z*:*E* = 75:25).

(*Z*)-**3aan**: **R*f*** = 0.40 (petroleum ether/EtOAc = 5/1). **$^{1}$H NMR** (400 MHz, $CDCl_3$): δ 7.77 (dd, *J* = 8.8, 5.6 Hz, 2H), 7.44-7.41 (m, 2H), 7.37 (d, *J* = 8.0 Hz, 2H), 7.32-7.28 (m, 3H), 7.11-7.07 (m, 7H), 6.97 (t, *J* = 8.6 Hz, 2H), 5.34 (s, 1H), 4.50-4.37 (m, 2H), 4.18-4.08 (m, 2H), 2.31 (s, 3H) ppm. **$^{13}$C NMR** (100 MHz, $CDCl_3$): δ 166.1 (d, *J* = 254.0 Hz), 164.3, 156.1, 136.9, 134.5, 134.0, 133.8, 132.6 (d, *J* = 9.5 Hz), 131.6, 131.59, 129.1, 129.0, 128.2, 128.1, 128.09, 127.6, 127.4, 124.4 (d, *J* = 2.9 Hz), 123.1,

115.6 (d, *J* = 22.0 Hz), 87.2, 85.8, 63.1, 46.3, 39.7, 21.1 ppm. **$^{19}$F NMR** (376 MHz, $CDCl_3$): δ -103.90 ppm. **HRMS** (ESI) *m/z*: $[M+Na]^+$ calcd. for $C_{34}H_{26}FNO_4Na$: 554.1744; Found: 554.1760.

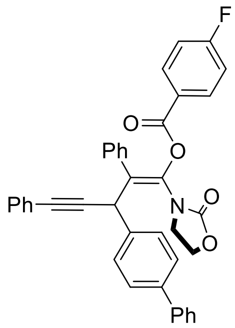


(*Z*)-**3aao**
$C_{39}H_{28}FNO_4$
M = 593.65 g/mol

**(*Z*)-3-([1,1'-biphenyl]-4-yl)-1-(2-oxooxazolidin-3-yl)-2,5-diphenylpent-1-en-4-yn-1-yl-4-fluorobenzoate** ((*Z*)-**3aao**): Prepared from 3-(phenylethynyl)oxazolidin-2-one (**1a**, 44.9 mg, 0.24 mmol) and 1-([1,1'-biphenyl]-4-yl)-3-phenylprop-2-yn-1-yl 4-fluorobenzoate (**2ao**, 81.3 mg, 0.2 mmol) according to the GP1 with 5 mol% $B(C_6F_5)_3$ at 80 ℃ for 24 h. Purification by flash chromatography on silica gel using petroleum ether/EtOAc (10/1 to 5/1) afforded (*Z*)-**3aao** as a white solid (80.7 mg, 68% yield, *Z*:*E* = 76:24).

(*Z*)-**3aao**: **$R_f$** = 0.40 (petroleum ether/EtOAc = 5/1). **$^1$H NMR** (400 MHz, $CDCl_3$): δ 7.78 (dd, *J* = 8.8, 5.4 Hz, 2H), 7.60-7.53 (m, 6H), 7.47-7.40 (m, 4H), 7.35-7.30 (m, 4H), 7.15-7.08 (m, 5H), 6.98 (t, *J* = 8.6 Hz, 2H), 5.42 (s, 1H), 4.52-4.38 (m, 2H), 4.19-4.09 (m, 2H) ppm. **$^{13}$C NMR** (100 MHz, $CDCl_3$): δ 166.1 (d, *J* = 254.0 Hz), 164.3, 156.0, 140.5, 140.1, 136.1, 134.7, 133.9, 132.6 (d, *J* = 9.4 Hz), 131.6, 131.4, 129.1, 128.7, 128.3, 128.2, 127.7, 127.4, 127.2, 127.0, 126.9, 124.4 (d, *J* = 2.9 Hz), 123.0, 115.6 (d, *J* = 22.0 Hz), 87.0, 86.1, 63.1, 46.2, 39.9 ppm. **$^{19}$F NMR** (376 MHz, $CDCl_3$): δ -103.71 ppm. **HRMS** (ESI) *m/z*: $[M+Na]^+$ calcd. for $C_{39}H_{28}FNO_4Na$: 616.1895; Found: 616.1899.

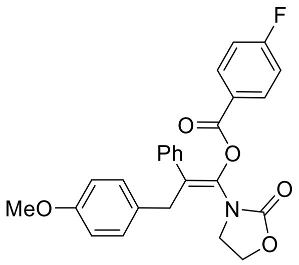

(*Z*)-**3aap**
$C_{26}H_{22}FNO_5$
M = 447.46 g/mol

**(*Z*)-3-(4-methoxyphenyl)-1-(2-oxooxazolidin-3-yl)-2-phenylprop-1-en-1-yl 4-fluorobenzoate** ((*Z*)-**3aap**): Prepared from 3-(phenylethynyl)oxazolidin-2-one (**1a**, 44.9 mg, 0.24 mmol) and 4-methoxybenzyl 4-fluorobenzoate (**2ap**, 52.1 mg, 0.2 mmol) according to the GP1 with 5 mol% $B(C_6F_5)_3$ at 80 ℃ for 12 h. Purification by flash chromatography on silica gel using petroleum ether/EtOAc (10/1 to 5/1) afforded (*Z*)-**3aap** as a colorless oil (44.7 mg, 50% yield, *Z*:*E* > 96:4).

(*Z*)-**3aap**: $\mathbf{R_f}$ = 0.45 (petroleum ether/EtOAc = 5/1). $^1$**H NMR** (400 MHz, $CDCl_3$): δ 8.20-8.15 (m, 2H), 7.32-7.26 (m, 3H), 7.23-7.14 (m, 4H), 7.07 (d, *J* = 8.4 Hz, 2H), 6.75 (d, *J* = 8.8 Hz, 2H), 4.09 (t, *J* = 8.0 Hz 2H), 3.74 (s, 3H), 3.71 (s, 2H), 3.48 (t, *J* = 8.0 Hz 2H) ppm. $^{13}$**C NMR** (100 MHz, $CDCl_3$): δ 166.4 (d, *J* = 254.4 Hz), 164.3, 158.1, 156.2, 136.4, 134.9, 133.1 (d, *J* = 9.5 Hz), 129.7, 129.2, 129.19, 128.6, 128.0, 124.6 (d, *J* = 2.7 Hz), 116.0 (d, *J* = 22.0 Hz), 113.8, 63.0, 55.1, 45.6, 37.1 ppm. $^{19}$**F NMR** (376 MHz, $CDCl_3$): δ -103.35 ppm. **HRMS** (ESI) *m/z*: $[M+Na]^+$ calcd. for $C_{26}H_{22}FNO_5Na$: 470.1380; Found: 470.1381.

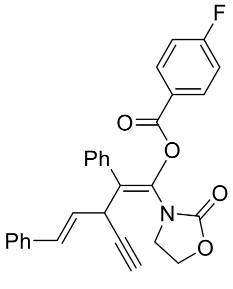

(*Z*)-**3aaq**
$C_{29}H_{22}FNO_4$
M = 467.50 g/mol

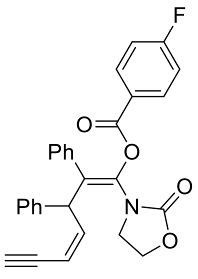

(*Z*)-**3aaq'**
$C_{29}H_{22}FNO_4$
M = 467.50 g/mol

**(1*Z*,4*E*)-3-ethynyl-1-(2-oxooxazolidin-3-yl)-2,5-diphenylpenta-1,4-dien-1-yl 4-fluorobenzoate** ((*Z*)-**3aaq**): Prepared from 3-(phenylethynyl)oxazolidin-2-one (**1a**, 44.9 mg, 0.24

mmol) and (*E*)-1-phenylpent-1-en-4-yn-3-yl 4-fluorobenzoate (**2aq**, 56.1 mg, 0.2 mmol) according to the GP1 with 5 mol% $B(C_6F_5)_3$ at 80 ℃ for 12 h. Purification by flash chromatography on silica gel using petroleum ether/EtOAc (10/1 to 5/1) afforded (*Z*)-**3aaq** as a colorless oil (50.5 mg, 54% yield, *Z*:*E* > 96:4, the desired product containing regioisomer (*Z*-**3aaq'**) which cannot be separated by chromatography).

(*Z*)-**3aaq**: **R*f*** = 0.35 (petroleum ether/EtOAc = 5/1). **$^1$H NMR** (400 MHz, $CDCl_3$): δ 7.75 (dd, *J* = 8.4, 5.6 Hz, 2H), 7.38-7.29 (m, 4H), 7.24-7.22 (m, 1H), 7.13- 7.07 (m, 3H), 7.06-6.94 (m, 4H), 6.45 (dd, *J* = 16.0, 8.0 Hz, 1H), 5.72 (d, *J* = 16.0 Hz, 1H), 4.85 (d, *J* = 8.0 Hz, 1H), 4.32-4.22 (m, 2H), 3.73 (t, *J* = 8.0 Hz, 2H), 2.86 (s, 1H) ppm.

(*Z*)-**3aaq': R*f*** = 0.35 (petroleum ether/EtOAc = 5/1). **$^1$H NMR** (400 MHz, $CDCl_3$): δ 7.75 (dd, *J* = 8.4, 5.6 Hz, 2H), 7.38-7.29 (m, 4H), 7.24-7.22 (m, 1H), 7.13- 7.07 (m, 3H), 7.06-6.94 (m, 4H), 6.26 (t, *J* = 10.4 Hz, 1H), 5.68 (d, *J* = 10.4 Hz, 1H), 5.50 (d, *J* = 10.4 Hz, 1H), 4.32-4.22 (m, 2H), 3.94-3.88 (m, 2H), 3.16 (s, 1H) ppm.

(*Z*)-**3aaq** and (*Z*)-**3aaq': $^{13}$C NMR** (100 MHz, $CDCl_3$): δ 166.11 (d, *J* = 254.1 Hz), δ 166.08 (d, *J* = 253.9 Hz) 164.5, 164.3, 156.1, 155.9, 144.1, 142.5, 139.7, 134.9, 134.7 (d, *J* = 8.5 Hz),133.1, 132.7, 132.6 (d, *J* = 9.5 Hz), 128.83, 128.80, 128.54, 128.50, 128.4, 128.1, 127.83, 127.76, 127.6, 127.0, 126.9, 129.8, 124.4 (d, *J* = 3.0 Hz), 124.3 (d, *J* = 2.9 Hz), 115.65 (d, *J* = 22.0 Hz), 115.60 (d, *J* = 22.0 Hz), 111.5, 110.1, 82.8, 81.8, 79.8, 77.8, 63.0, 62.8, 50.7, 47.4, 46.0, 45.3 ppm. **$^{19}$F NMR** (376 MHz, $CDCl_3$): δ -103.71, -103.92 ppm. **HRMS** (ESI) *m/z*: $[M+Na]^+$ calcd. for $C_{29}H_{22}FNO_4Na$: 490.1431; Found: 490.1429.

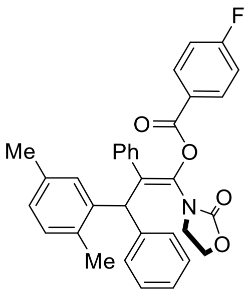


(*Z*)-**3aar**
$C_{33}H_{28}FNO_4$
M = 521.59 g/mol

**(*Z*)-3-(2,5-dimethylphenyl)-1-(2-oxooxazolidin-3-yl)-2,3-diphenylprop-1-en-1-yl-4-fluorobenzoate** ((*Z*)-**3aar**): Prepared from 3-(phenylethynyl)oxazolidin-2-one (**1a**, 44.9 mg, 0.24 mmol) and (*R*)-(2,5-dimethylphenyl)(phenyl)methyl 4-fluorobenzoate ((R)-**2ar**, 66.9 mg, 0.2 mmol) according to the GP1 with 5 mol% $B(C_6F_5)_3$ at 80 ℃ for 12 h. Purification by flash chromatography on silica gel using petroleum ether/EtOAc (10/1 to 5/1) afforded (*Z*)-**3aar** as a white solid (73.0 mg, 70% yield, *Z*:*E* > 96:4).

(*Z*)-**3aar**: **R*f*** = 0.35 (petroleum ether/EtOAc = 5/1). **$^1$H NMR** (400 MHz, $CDCl_3$): δ 7.77 (dd, *J* = 8.8, 5.6 Hz, 2H), 7.41 (d, *J* = 7.6 Hz, 2H), 7.27 (t, *J* = 7.4 Hz, 2H), 7.20 (t, *J* = 6.4 Hz, 3H), 7.10-7.00 (m, 4H), 6.99-6.92 (m, 4H), 5.57 (s, 1H), 3.84-3.78 (m, 1H), 3.73-3.66 (m, 1H), 3.36-3.29 (m, 1H), 3.25-3.17 (m, 1H), 2.35 (d, *J* = 10.4 Hz, 3H), 2.24 (d, *J* = 12.4 Hz, 3H) ppm. **$^{13}$C NMR** (100 MHz, $CDCl_3$): δ 166.0 (d, *J* = 253.9 Hz), 164.4, 155.8, 140.6, 139.3, 137.2, 135.1, 135.06, 134.2, 133.6, 132.6 (d, *J* = 9.4 Hz), 130.5, 130.1, 129.8, 128.6, 128.2, 127.5, 127.3, 127.2, 126.5, 124.4 (d, *J* = 2.9 Hz), 115.5 (d, *J* = 21.9 Hz), 62.7, 52.0, 44.7, 21.0, 19.4 ppm. **$^{19}$F NMR** (376 MHz, $CDCl_3$): δ -103.98 ppm. **HRMS** (ESI) *m/z*: $[M+Na]^+$ calcd. for $C_{33}H_{28}FNO_4Na$: 544.1900; Found: 544.1904.

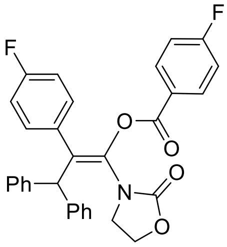


(*Z*)-**3ba**
$C_{31}H_{23}F_2NO_4$
M = 511.52 g/mol

**(*Z*)-2-(4-fluorophenyl)-1-(2-oxooxazolidin-3-yl)-3,3-diphenylprop-1-en-1-yl 4-fluorobenzoate** ((*Z*)-**3ba**): Prepared from 3-((4-fluorophenyl)ethynyl)oxazolidin-2-one (**1b**, 49.2 mg, 0.24 mmol) and benzhydryl 4-fluorobenzoate (**2a**, 61.3 mg, 0.2 mmol) according to the GP1 with 5 mol% $B(C_6F_5)_3$ at 80 ℃ for 24 h. Purification by flash chromatography on silica gel using petroleum ether/EtOAc (10/1 to 5/1) afforded (*Z*)-**3ba** as a white solid (81.8 mg, 80% yield, *Z*:*E* = 93:7).

(*Z*)-**3ba**: **R*f*** = 0.30 (petroleum ether/EtOAc = 5/1). **$^1$H NMR** (400 MHz, $CDCl_3$): δ 7.80 (dd, *J* = 8.8, 5.4 Hz, 2H), 7.32-7.25 (m, 8H), 7.22-7.18 (m, 2H), 7.03-6.97 (m, 4H), 6.70 (t, *J* = 8.6 Hz, 2H), 5.57 (s, 1H), 4.06 (t, *J* = 8.0 Hz, 2H), 3.60 (t, *J* = 8.0 Hz, 2H) ppm. **$^{13}$C NMR** (100 MHz, $CDCl_3$): δ 166.2 (d, *J* = 254.4 Hz), 164.4, 161.8 (d, *J* = 245.3 Hz), 155.8, 140.4, 135.3, 133.1, 132.6 (d, *J* = 9.5 Hz), 131.8 (d, *J* = 3.4 Hz), 130.8 (d, *J* = 8.0 Hz), 129.6, 128.2, 126.7, 124.2 (d, *J* = 2.9 Hz), 115.7 (d, *J* = 22.0 Hz), 114.5 (d, *J* = 21.3 Hz), 62.8, 53.8, 45.2 ppm. **$^{19}$F NMR** (376 MHz, $CDCl_3$): δ -103.44, -114.37 ppm. **HRMS** (ESI) *m/z*: $[M+Na]^+$ calcd. for $C_{31}H_{23}F_2NO_4Na$: 534.1493; Found: 534.1500.

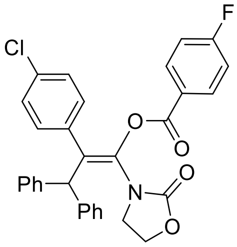


(*Z*)-**3ca**
$C_{31}H_{23}ClFNO_4$
M = 527.98 g/mol

**(*Z*)-2-(4-chlorophenyl)-1-(2-oxooxazolidin-3-yl)-3,3-diphenylprop-1-en-1-yl 4-fluorobenzoate** ((*Z*)-**3ca**): Prepared from 3-((4-chlorophenyl)ethynyl)oxazolidin-2-one (**1c**, 53.2 mg, 0.24 mmol) and benzhydryl 4-fluorobenzoate (**2a**, 61.3 mg, 0.2 mmol) according to the GP1 with 5 mol% $B(C_6F_5)_3$ at 80 ℃ for 36 h. Purification by flash chromatography on silica gel using petroleum ether/EtOAc (10/1 to 5/1) afforded (*Z*)-**3ca** as a white solid (68.6 mg, 65% yield, *Z*:*E* = 95:5).

(*Z*)-**3ca**: **R*f*** = 0.30 (petroleum ether/EtOAc = 5/1). **$^1$H NMR** (400 MHz, $CDCl_3$): δ 7.80 (dd, *J* = 8.6, 5.4 Hz, 2H), 7.33-7.24 (m, 8H), 7.21 (t, *J* = 6.8 Hz, 2H), 7.04-6.94 (m,

6H), 5.57 (s, 1H), 4.05 (t, *J* = 8.0 Hz, 2H), 3.59 (t, *J* = 8.0 Hz, 2H) ppm. **$^{13}$C NMR** (100 MHz, $CDCl_3$): δ 166.2 (d, *J* = 254.5 Hz), 164.4, 155.7, 140.3, 135.3, 134.5, 133.3, 132.9, 132.6 (d, *J* = 9.5 Hz), 130.4, 129.6, 128.3, 127.8, 126.8, 124.1 (d, *J* = 2.9 Hz), 115.8 (d, *J* = 22.1 Hz), 62.8, 53.8, 45.2 ppm. **$^{19}$F NMR** (376 MHz, $CDCl_3$): δ -105.89 ppm. **HRMS** (ESI) *m/z*: $[M+Na]^+$ calcd. for $C_{31}H_{23}ClFNO_4Na$: 550.1198; Found: 550.1208.

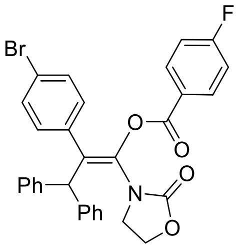


(*Z*)-**3da**
$C_{31}H_{23}BrFNO_4$
M = 572.43 g/mol

**(*Z*)-2-(4-bromophenyl)-1-(2-oxooxazolidin-3-yl)-3,3-diphenylprop-1-en-1-yl 4-fluorobenzoate** ((*Z*)-**3da**): Prepared from 3-((4-chlorophenyl)ethynyl)oxazolidin-2-one (**1d**, 63.9 mg, 0.24 mmol) and benzhydryl 4-fluorobenzoate (**2a**, 61.3 mg, 0.2 mmol) according to the GP1 with 10 mol% $B(C_6F_5)_3$ at 80 ℃ for 48 h. Purification by flash chromatography on silica gel using petroleum ether/EtOAc (10/1 to 5/1) afforded (*Z*)-**3da** as a white solid (73.3 mg, 64% yield, *Z*:*E* > 96:4).

(*Z*)-**3da**: **$R_f$** = 0.30 (petroleum ether/EtOAc = 5/1). **$^1$H NMR** (400 MHz, $CDCl_3$): δ 7.82-7.78 (m, 2H), 7.32-7.25 (m, 8H), 7.24-7.19 (m, 2H), 7.15 (d, *J* = 8.4 Hz, 2H), 7.02 (t, *J* = 8.6 Hz, 2H), 6.89 (d, *J* = 8.4 Hz, 2H), 5.56 (s, 1H), 4.05 (t, *J* = 8.0 Hz, 2H), 3.59 (t, *J* = 8.0 Hz, 2H) ppm. **$^{13}$C NMR** (100 MHz, $CDCl_3$): δ 166.3 (d, *J* = 254.5 Hz), 164.4, 155.7, 140.2, 135.2, 135.0, 133.0, 132.7 (d, *J* = 9.0 Hz), 130.7, 130.6, 129.6, 128.3, 126.8, 124.1 (d, *J* = 2.9 Hz), 121.5, 115.8 (d, *J* = 22.3 Hz), 62.8, 53.7, 45.3 ppm. **$^{19}$F NMR** (376 MHz, $CDCl_3$): δ -103.30 ppm. **HRMS** (ESI) *m/z*: $[M+Na]^+$ calcd. for $C_{31}H_{23}BrFNO_4Na$: 594.0692; Found: 594.0701.

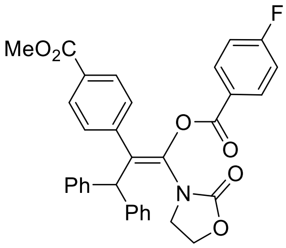


(*Z*)-**3ea**
$C_{32}H_{26}FNO_6$
M = 551.57 g/mol

**(*Z*)-2-(4-(methoxycarbonyl)phenyl)-1-(2-oxooxazolidin-3-yl)-3,3-diphenylprop-1-en-1-yl 4-fluorobenzoate** ((*Z*)-**3ea**): Prepared from methyl 4-((2-oxooxazolidin-3-yl)ethynyl)benzoate (**1e**, 58.9 mg, 0.24 mmol) and benzhydryl 4-fluorobenzoate (**2a**, 61.3 mg, 0.2 mmol) according to the GP1 with 5 mol% $B(C_6F_5)_3$ at 80 ℃ for 36 h. Purification by flash chromatography on silica gel using petroleum ether/EtOAc (10/1 to 5/1) afforded (*Z*)-**3ea** as a white solid (71.7 mg, 65% yield, *Z*:*E* = 89:11).

(*Z*)-**3ea**: **R*f*** = 0.30 (petroleum ether/EtOAc = 5/1). **$^1$H NMR** (400 MHz, $CDCl_3$): δ 7.78 (dd, *J* = 8.8, 5.2 Hz, 2H), 7.69 (d, *J* = 8.4 Hz, 2H), 7.32 (d, *J* = 7.6 Hz, 4H), 7.27 (t, *J* = 7.4 Hz, 4H), 7.21 (t, *J* = 7.0 Hz, 2H), 7.12 (d, *J* = 8.0 Hz, 2H), 6.99 (t, *J* = 8.6 Hz, 2H), 5.61 (s, 1H), 4.04 (t, *J* = 8.0 Hz, 2H), 3.79 (s, 3H), 3.58 (t, *J* = 8.0 Hz, 2H) ppm. **$^{13}$C NMR** (100 MHz, $CDCl_3$): δ 166.2 (d, *J* = 254.6 Hz), 166.6, 164.3, 155.6, 141.0, 140.2, 135.2, 133.2, 132.6 (d, *J* = 9.6 Hz), 129.5, 129.1, 128.9, 128.8, 128.3, 126.8, 124.0 (d, *J* = 2.8 Hz), 115.8 (d, *J* = 22.0 Hz), 62.8, 53.7, 52.0, 45.1 ppm. **$^{19}$F NMR** (376 MHz, $CDCl_3$): δ -103.32 ppm. **HRMS** (ESI) *m/z*: $[M+Na]^+$ calcd. for $C_{33}H_{26}FNO_6Na$: 574.1642; Found: 574.1647.

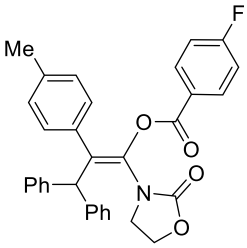


(*Z*)-**3fa**
$C_{32}H_{26}FNO_4$
M = 507.56 g/mol

**(*Z*)-1-(2-oxooxazolidin-3-yl)-3,3-diphenyl-2-(p-tolyl)prop-1-en-1-yl 4-fluorobenzoate** ((*Z*)-**3fa**): Prepared from methyl 3-(*p*-tolylethynyl)oxazolidin-2-one (**1f**, 48.3 mg, 0.24 mmol)

and benzhydryl 4-fluorobenzoate (**2a**, 61.3 mg, 0.2 mmol) according to the GP1 with 5 mol% $B(C_6F_5)_3$ at 80 ℃ for 24 h. Purification by flash chromatography on silica gel using petroleum ether/EtOAc (10/1 to 5/1) afforded (*Z*)-**3fa** as a white solid (74.5 mg, 73% yield, *Z*:*E* = 94:6).

(*Z*)-**3fa**: **R*f*** = 0.35 (petroleum ether/EtOAc = 5/1). **$^1$H NMR** (400 MHz, $CDCl_3$): δ 7.78 (dd, *J* = 8.0, 5.6 Hz, 2H), 7.33 (d, *J* = 7.6 Hz, 4H), 7.27 (t, *J* = 7.4 Hz, 4H), 7.20 (d, *J* = 7.2 Hz, 2H), 6.98 (t, *J* = 8.6 Hz, 2H), 6.92 (d, *J* = 7.6 Hz, 2H), 6.81 (d, *J* = 8.0 Hz, 2H), 5.56 (s, 1H), 4.02 (t, *J* = 8.0 Hz, 2H), 3.58 (t, *J* = 8.0 Hz, 2H), 2.10 (s, 3H) ppm. **$^{13}$C NMR** (100 MHz, $CDCl_3$): δ 166.1 (d, *J* = 253.7 Hz), 164.6, 155.9, 140.6, 136.9, 135.0, 134.0, 133.1, 132.6 (d, *J* = 9.5 Hz), 129.7, 128.7, 128.2, 128.15, 126.6, 124.5 (d, *J* = 2.9 Hz), 115.5 (d, *J* = 21.9 Hz), 62.8, 54.1, 45.4, 21.0 ppm. **$^{19}$F NMR** (376 MHz, $CDCl_3$): δ -103.99 ppm. **HRMS** (ESI) *m/z*: $[M+Na]^+$ calcd. for $C_{32}H_{26}FNO_4Na$: 530.1744; Found: 530.1747.

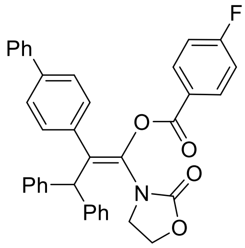


(*Z*)-**3ga**
$C_{37}H_{28}FNO_4$
M = 569.63 g/mol

**(*Z*)-2-([1,1'-biphenyl]-4-yl)-1-(2-oxooxazolidin-3-yl)-3,3-diphenylprop-1-en-1-yl 4-fluorobenzoate** ((*Z*)-**3ga**): Prepared from methyl 3-([1,1'-biphenyl]-4-ylethynyl)oxazolidin-2-one (**1g**, 63.2 mg, 0.24 mmol) and benzhydryl 4-fluorobenzoate (**2a**, 61.3 mg, 0.2 mmol) according to the GP1 with 5 mol% $B(C_6F_5)_3$ at 80 ℃ for 36 h. Purification by flash chromatography on silica gel using petroleum ether/EtOAc (10/1 to 5/1) afforded (*Z*)-**3ga** as a white solid (82.0 mg, 72% yield, *Z*:*E* = 94:6).

(*Z*)-**3ga**: **R*f*** = 0.30 (petroleum ether/EtOAc = 5/1). **$^1$H NMR** (400 MHz, $CDCl_3$): δ 7.79 (dd, *J* = 8.8, 5.2 Hz, 2H), 7.46-7.18 (m, 17H), 7.10 (d, *J* = 8.4 Hz, 2H), 6.96 (t, *J* = 8.4

Hz, 2H), 5.62 (s, 1H), 4.04 (t, *J* = 8.0 Hz, 2H), 3.61 (t, *J* = 8.0 Hz, 2H) ppm. **$^{13}$C NMR** (100 MHz, $CDCl_3$): δ 166.1 (d, *J* = 254.1 Hz), 164.6, 155.8, 140.5, 140.3, 139.8, 135.2, 135.1, 133.7, 132.6 (d, *J* = 9.5 Hz), 129.7, 129.4, 128.6, 128.2, 127.2, 126.7, 126.1, 124.4 (d, *J* = 2.9 Hz), 115.6 (d, *J* = 22.0 Hz), 62.8, 54.0, 45.4 ppm. **$^{19}$F NMR** (376 MHz, $CDCl_3$): δ -103.74 ppm (The desired product containing a trace amount hydroacyloxylation side product). **HRMS** (ESI) *m/z*: $[M+Na]^+$ calcd. for $C_{37}H_{28}FNO_4Na$: 592.1900; Found: 592.1911.

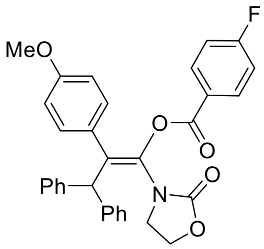


(*Z*)-**3ha**
$C_{32}H_{26}FNO_5$
M = 523.56 g/mol

**(*Z*)-2-(4-methoxyphenyl)-1-(2-oxooxazolidin-3-yl)-3,3-diphenylprop-1-en-1-yl 4-fluorobenzoate** ((*Z*)-**3ha**): Prepared from methyl 3-((4-methoxyphenyl)ethynyl)oxazolidin-2-one (**1h**, 52.1 mg, 0.24 mmol) and benzhydryl 4-fluorobenzoate (**2a**, 61.3 mg, 0.2 mmol) according to the GP1 with 5 mol% $B(C_6F_5)_3$ at 80 ℃ for 36 h. Purification by flash chromatography on silica gel using petroleum ether/EtOAc (10/1 to 5/1) afforded (*Z*)-**3ha** as a white solid (66.0 mg, 63% yield, *Z*:*E* = 94:6).

(*Z*)-**3ha**: **R*f*** = 0.35 (petroleum ether/EtOAc = 5/1). **$^{1}$H NMR** (400 MHz, $CDCl_3$): δ 7.81 (dd, *J* = 8.6, 5.4 Hz, 2H), 7.32-7.25 (m, 8H), 7.20 (t, *J* = 7.0 Hz, 2H), 7.00 (t, *J* = 8.6 Hz, 2H), 6.94 (d, *J* = 8.8 Hz, 2H), 6.54 (d, *J* = 8.4 Hz, 2H), 5.56 (s, 1H), 4.05 (t, *J* = 8.0 Hz, 2H), 3.60 (t, *J* = 8.0 Hz, 2H), 3.60 (s, 3H) ppm. **$^{13}$C NMR** (100 MHz, $CDCl_3$): δ 166.1 (d, *J* = 253.9 Hz), 164.6, 158.5, 155.9, 140.6, 134.9, 133.7, 132.6 (d, *J* = 9.5 Hz), 130.1, 129.6, 128.9, 128.2, 126.6, 124.4 (d, *J* = 2.9 Hz), 115.6 (d, *J* = 22.0 Hz), 112.9, 62.8, 54.9, 54.0, 45.4 ppm. **$^{19}$F NMR** (376 MHz, $CDCl_3$): δ -103.86 ppm. **HRMS** (ESI) *m/z*: $[M+Na]^+$ calcd. for $C_{32}H_{26}FNO_5Na$: 546.1693; Found: 546.1703.

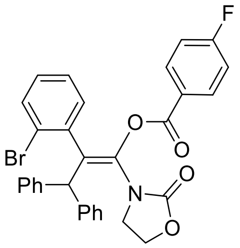

(*Z*)-**3ia**
$C_{31}H_{23}BrFNO_4$
M = 572.43 g/mol

**(*Z*)-2-(2-bromophenyl)-1-(2-oxooxazolidin-3-yl)-3,3-diphenylprop-1-en-1-yl 4-fluorobenzoate** ((*Z*)-**3ia**): Prepared from methyl 3-((2-bromophenyl)ethynyl)oxazolidin-2-one (**1i**, 63.9 mg, 0.24 mmol) and benzhydryl 4-fluorobenzoate (**2a**, 61.3 mg, 0.2 mmol) according to the GP1 with 10 mol% $B(C_6F_5)_3$ at 80 ℃ for 24 h. Purification by flash chromatography on silica gel using petroleum ether/EtOAc (10/1 to 5/1) afforded (*Z*)-**3ia** as a white solid (99.5 mg, 87% yield, *Z*:*E* = 93:7).

(*Z*)-**3ia**: **R*f*** = 0.35 (petroleum ether/EtOAc = 5/1). **$^1$H NMR** (400 MHz, $CDCl_3$): δ 7.76 (dd, *J* = 8.6, 5.4 Hz, 2H), 7.55 (d, *J* = 7.6 Hz, 2H), 7.37-7.29 (m, 5H), 7.25-7.16 (m, 3H), 7.12 (d, *J* = 7.2 Hz, 1H), 7.08-7.04 (m, 1H), 6.96 (t, *J* = 8.6 Hz, 2H), 6.91 (t, *J* = 7.6 Hz, 1H), 6.84-6.78 (m, 1H), 5.60 (s, 1H), 3.76 (t, *J* = 8.0 Hz, 2H), 3.44 (t, *J* = 8.0 Hz, 2H) ppm. **$^{13}$C NMR** (100 MHz, $CDCl_3$): δ 166.0 (d, *J* = 254.1 Hz), 163.6, 154.7, 141.6, 139.7, 137.2, 135.8, 132.5 (d, *J* = 9.5 Hz), 132.3, 131.3, 131.0, 130.0, 129.0, 128.7, 128.2, 128.0, 126.6, 126.5, 126.3, 124.2 (d, *J* = 2.9 Hz), 123.6, 115.6 (d, *J* = 22.0 Hz), 62.5, 53.6, 44.4 ppm. **$^{19}$F NMR** (376 MHz, $CDCl_3$): δ -103.71 ppm. **HRMS** (ESI) *m/z*: $[M+Na]^+$ calcd. for $C_{31}H_{23}BrFNO_4Na$: 594.0692; Found: 594.0706.

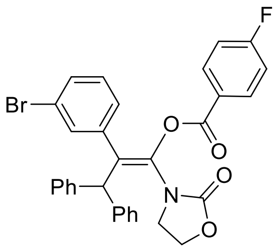

(*Z*)-**3ja**
$C_{31}H_{23}BrFNO_4$
M = 572.43 g/mol

**(*Z*)-2-(3-bromophenyl)-1-(2-oxooxazolidin-3-yl)-3,3-diphenylprop-1-en-1-yl 4-fluorobenzoate** ((*Z*)-**3ja**): Prepared from 3-((3-bromophenyl)ethynyl)oxazolidin-2-one (**1j**, 63.9 mg, 0.24 mmol) and benzhydryl 4-fluorobenzoate (**2a**, 61.3 mg, 0.2 mmol) according to the GP1 with 5 mol% $B(C_6F_5)_3$ at 80 ℃ for 12 h. Purification by flash chromatography on silica gel using petroleum ether/EtOAc (10/1 to 5/1) afforded (*Z*)-**3ja** as a white solid (89.3 mg, 78% yield, *Z*:*E* > 96:4).

(*Z*)-**3ja**: **$R_f$** = 0.35 (petroleum ether/EtOAc = 5/1). **$^1$H NMR** (400 MHz, $CDCl_3$): δ 7.83-7.79 (m, 2H), 7.33-7.26 (m, 8H), 7.23-7.20 (m, 3H), 7.11 (d, *J* = 8.0 Hz, 1H), 7.05-6.96 (m, 3H), 6.87 (t, *J* = 7.8 Hz, 1H), 5.57 (s, 1H), 4.03 (t, J = 8.0 Hz, 2H), 3.56 (t, *J* = 8.0 Hz, 2H) ppm. **$^{13}$C NMR** (100 MHz, $CDCl_3$): δ 166.2 (d, *J* = 254.4 Hz), 164.4, 155.6, 140.3, 138.1, 135.6, 132.7 (d, *J* = 9.8 Hz), 132.1, 130.3, 129.6, 129.0, 128.3, 127.5, 126.8, 124.1 (d, *J* = 2.9 Hz), 121.4, 115.8 (d, *J* = 22.0 Hz), 62.8, 53.7, 45.1 ppm. **$^{19}$F NMR** (376 MHz, $CDCl_3$): δ -103.37 ppm. **HRMS** (ESI) *m/z*: $[M+Na]^+$ calcd. for $C_{31}H_{23}BrFNO_4Na$: 594.0692; Found: 594.0697.

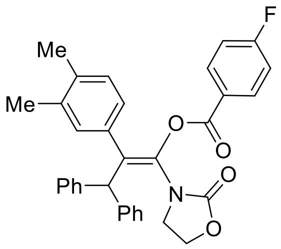


(*Z*)-**3ka**
$C_{33}H_{28}FNO_4$
M = 521.59 g/mol

**(Z)-2-(3,4-dimethylphenyl)-1-(2-oxooxazolidin-3-yl)-3,3-diphenylprop-1-en-1-yl 4-fluorobenzoate** ((*Z*)-**3ka**): Prepared from 3-((3,4-dimethylphenyl)ethynyl)oxazolidin-2-one (**1k**, 51.7 mg, 0.24 mmol) and benzhydryl 4-fluorobenzoate (**2a**, 61.3 mg, 0.2 mmol) according to the GP1 with 5 mol% $B(C_6F_5)_3$ at 80 ℃ for 24 h. Purification by flash chromatography on silica gel using petroleum ether/EtOAc (10/1 to 5/1) afforded (*Z*)-**3ka** as a white solid (76.1 mg, 73% yield, *Z*:*E* = 95:5).

(*Z*)-**3ka**: **$R_f$** = 0.30 (petroleum ether/EtOAc = 5/1). **$^1$H NMR** (400 MHz, $CDCl_3$): δ 7.78 (dd, *J* = 8.6, 5.4 Hz, 2H), 7.33 (d, *J* = 7.6 Hz, 4H), 7.27 (t, *J* = 7.4 Hz, 4H), 7.20 (t, *J* =

7.2 Hz, 2H), 6.98 (t, *J* = 8.6 Hz, 2H), 6.77 (s, 3H), 5.55 (s, 1H), 4.00 (t, *J* = 8.0 Hz, 2H), 3.56 (t, *J* = 8.0 Hz, 2H), 2.01 (s, 3H), 1.96 (s, 3H) ppm. **$^{13}$C NMR** (100 MHz, $CDCl_3$): δ 166.0 (d, *J* = 253.8 Hz), 164.7, 155.9, 140.7, 135.5, 134.9, 134.1, 133.5, 132.6 (d, *J* = 9.8 Hz), 130.2, 129.7, 128.7, 128.1, 126.6, 126.1, 124.6 (d, *J* = 2.9 Hz), 121.4, 115.5 (d, *J* = 22.0 Hz), 62.8, 54.2, 45.4, 19.4, 19.3 ppm. **$^{19}$F NMR** (376 MHz, $CDCl_3$): δ -104.09 ppm. **HRMS** (ESI) *m/z*: $[M+Na]^+$ calcd. for $C_{33}H_{28}FNO_4Na$: 544.1900; Found: 544.1901.

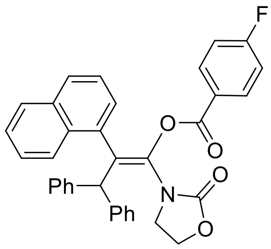


(*Z*)-**3la**
$C_{35}H_{26}FNO_4$
M = 543.59 g/mol

**(*Z*)-2-(naphthalen-1-yl)-1-(2-oxooxazolidin-3-yl)-3,3-diphenylprop-1-en-1-yl 4-fluorobenzoate** ((*Z*)-**3la**): Prepared from 3-(naphthalen-1-ylethynyl)oxazolidin-2-one (**1l**, 56.9 mg, 0.24 mmol) and benzhydryl 4-fluorobenzoate (**2a**, 61.3 mg, 0.2 mmol) according to the GP1 with 5 mol% $B(C_6F_5)_3$ at 80 ℃ for 24 h. Purification by flash chromatography on silica gel using petroleum ether/EtOAc (10/1 to 5/1) afforded (*Z/E*)-**3la** as a white solid (96.7 mg, 89% yield, *Z*:*E* = 88:12).

(*Z/E*)-**3la**: **R*f*** = 0.35 (petroleum ether/EtOAc = 5/1). (*Z*)-**3la: $^1$H NMR** (400 MHz, $CDCl_3$): δ 8.31 (d, *J* = 8.4 Hz, 1H), 7.69 (d, *J* = 7.6 Hz, 2H), 7.57 (d, *J* = 8.4 Hz, 1H), 7.47-7.01 (m, 12H), 6.94-6.85 (m, 3H), 6.73 (t, *J* = 8.6 Hz, 2H), 5.76 (s, 1H), 4.19-4.13 (m, 1H), 3.90 (q, *J* = 8.8 Hz, 1H), 3.74-3.66 (m, 1H), 3.53-3.46 (m, 1H) ppm. (*E*)-**3la: $^1$H NMR** (400 MHz, $CDCl_3$): δ 8.12 (d, *J* = 7.2 Hz, 1H), 7.88(dd, *J* = 8.8, 5.6 Hz, 2H), 7.80 (d, *J* = 7.2 Hz, 2H), 7.75 (d, *J* = 7.6 Hz, 2H), 7.47-7.01 (m, 14H), 5.36 (s, 1H), 4.12-4.07 (m, 1H), 3.74-3.66 (m, 1H), 3.34-3.27 (m, 1H), 3.25-3.18 (m, 1H) ppm. (*Z/E*)-**3la: $^{13}$C NMR** (100 MHz, $CDCl_3$): δ 165.8 (d, *J* = 253.9 Hz), 164.7, 155.7, 142.0, 140.9, 139.8, 136.8, 135.7, 133.5, 133.0, 132.2 (d, *J* = 9.5 Hz), 131.4, 130.7, 130.1,

129.8, 129.0, 128.4, 128.3, 128.1, 127.7, 127.6, 127.5, 126.9, 126.7, 126.4, 126.3, 126.2, 126.0, 125.9, 125.5, 125.2, 125.1, 124.2, 124.0 (d, *J* = 2.9 Hz), 115.6, 115.3 (d, *J* = 22.0 Hz), 62.9, 62.87, 55.3, 54.0, 45.4, 44.4 ppm. (*Z/E*)-**3la:** $^{19}$**F NMR** (376 MHz, $CDCl_3$): δ -103.80, -104.10 ppm. **HRMS** (ESI) *m/z*: $[M+Na]^+$ calcd. for $C_{35}H_{26}FNO_4Na$: 566.1744; Found: 566.1749.

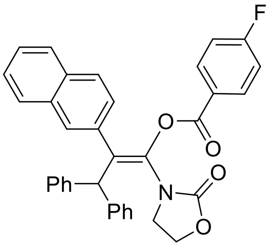

(*Z*)-**3ma**
$C_{35}H_{26}FNO_4$
M = 543.59 g/mol

**(Z)-2-(naphthalen-2-yl)-1-(2-oxooxazolidin-3-yl)-3,3-diphenylprop-1-en-1-yl 4-fluorobenzoate** ((*Z*)-**3ma**): Prepared from 3-(naphthalen-2-ylethynyl)oxazolidin-2-one (**1m**, 56.9 mg, 0.24 mmol) and benzhydryl 4-fluorobenzoate (**2a**, 61.3 mg, 0.2 mmol) according to the GP1 with 5 mol% $B(C_6F_5)_3$ at 80 °C for 24 h. Purification by flash chromatography on silica gel using petroleum ether/EtOAc (10/1 to 5/1) afforded (*Z*)-**3ma** as a white solid (80.4 mg, 74% yield, *Z*:*E* = 93:7).

(*Z*)-**3ma**: $\mathbf{R_f}$ = 0.35 (petroleum ether/EtOAc = 5/1). $^1$**H NMR** (400 MHz, $CDCl_3$): δ 7.72 (dd, *J* = 8.8, 5.6 Hz, 2H), 7.62-7.54 (m, 2H), 7.49 (d, *J* = 6.4 Hz, 2H), 7.37 (d, *J* = 7.6 Hz, 4H), 7.33-7.30 (m, 2H), 7.26 (t, *J* = 7.4 Hz, 4H), 7.19 (t, *J* = 7.4 Hz, 3H), 6.87 (t, *J* = 8.6 Hz, 2H), 5.68 (s, 1H), 4.04 (t, *J* = 8.0 Hz, 2H), 3.62 (t, *J* = 8.0 Hz, 2H) ppm. $^{13}$**C NMR** (100 MHz, $CDCl_3$): δ 166.0 (d, *J* = 254.1 Hz), 164.6, 155.9, 140.5, 135.4, 133.9, 133.6, 132.6 (d, *J* = 9.5 Hz), 132.5, 132.2, 128.2, 128.1, 127.9, 127.3, 127.1, 126.7, 125.9, 125.7, 124.2 (d, *J* = 2.8 Hz), 115.5 (d, *J* = 22.0 Hz), 62.8, 54.2, 45.4 ppm. $^{19}$**F NMR** (376 MHz, $CDCl_3$): δ -103.78 ppm. **HRMS** (ESI) *m/z*: $[M+Na]^+$ calcd. for $C_{35}H_{26}FNO_4Na$: 566.1744; Found: 566.1750.

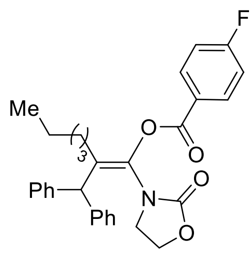

(*E*)-**3na**
$C_{30}H_{30}FNO_4$
M = 487.57 g/mol

**(*E*)-2-benzhydryl-1-(2-oxooxazolidin-3-yl)hept-1-en-1-yl 4-fluorobenzoate** ((*E*)-**3na**): Prepared from 3-(hept-1-yn-1-yl)oxazolidin-2-one (**1n**, 43.5 mg, 0.24 mmol) and benzhydryl 4-fluorobenzoate (**2a**, 61.3 mg, 0.2 mmol) according to the GP1 with 10 mol% $B(C_6F_5)_3$ at 80 ℃ for 36 h. Purification by flash chromatography on silica gel using petroleum ether/EtOAc (10/1 to 5/1) afforded (*E*)-**3na** as a white solid (58.6 mg, 60% yield, *E*:*Z* > 96:4).

(*E*)-**3na**: **R*f*** = 0.40 (petroleum ether/EtOAc = 5/1). **$^1$H NMR** (400 MHz, $CDCl_3$): δ 8.16 (dd, *J* = 8.8, 5.4 Hz, 2H), 7.35-7.23 (m, 10H), 7.16 (t, *J* = 8.6 Hz, 2H), 5.29 (s, 1H), 4.09 (t, *J* = 8.0 Hz, 2H), 3.56 (t, *J* = 8.0 Hz, 2H), 1.99-1.94(m, 2H), 0.98-0.90 (m, 6H), 0.63 (t, *J* = 6.8 Hz, 3H) ppm. **$^{13}$C NMR** (100 MHz, $CDCl_3$): δ 166.3 (d, *J* = 254.4 Hz), 164.5, 156.0, 141.0, 134.2, 133.0, 132.9 (d, *J* = 9.5 Hz), 129.4, 128.3, 126.7, 124.7 (d, *J* = 2.9 Hz), 115.9 (d, *J* = 22.0 Hz), 62.8, 51.9, 45.7, 31.9, 29.8, 27.9, 21.8, 13.7 ppm. **$^{19}$F NMR** (376 MHz, $CDCl_3$): δ -103.48 ppm. **HRMS** (ESI) *m/z*: $[M+Na]^+$ calcd. for $C_{30}H_{30}FNO_4Na$: 510.2057; Found: 510.2056.

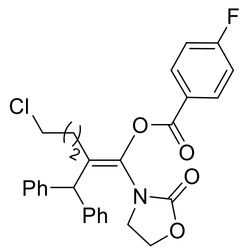

(*E*)-**3oa**
$C_{28}H_{25}ClFNO_4$
M = 493.96 g/mol

**(*E*)-2-benzhydryl-5-chloro-1-(2-oxooxazolidin-3-yl)pent-1-en-1-yl 4-fluorobenzoate** ((*E*)-**3oa**): Prepared from 3-(5-chloropent-1-yn-1-yl)oxazolidin-2-one (**1o**, 45.0 mg, 0.24 mmol)

and benzhydryl 4-fluorobenzoate (**2a**, 61.3 mg, 0.2 mmol) according to the GP1 with 10 mol% $B(C_6F_5)_3$ at 80 ℃ for 24 h. Purification by flash chromatography on silica gel using petroleum ether/EtOAc (10/1 to 5/1) afforded (*E*)-**3oa** as a white solid (75.1 mg, 76% yield, *E*:*Z* > 96:4).

(*E*)-**3oa**: **R*f*** = 0.40 (petroleum ether/EtOAc = 5/1). **$^1$H NMR** (400 MHz, $CDCl_3$): δ 8.16 (dd, *J* = 8.6, 5.4 Hz, 2H), 7.36-7.24 (m, 10H), 7.16 (t, *J* = 8.6 Hz, 2H), 5.32 (s, 1H), 4.13 (t, *J* = 8.0 Hz, 2H), 3.58 (t, *J* = 8.0 Hz, 2H), 3.15 (t, *J* = 6.0 Hz, 2H), 2.20-2.16 (m, 2H), 1.37-1.30 (m, 2H) ppm. **$^{13}$C NMR** (100 MHz, $CDCl_3$): δ 166.5 (d, *J* = 254.6 Hz), 164.5, 156.1, 140.8, 134.9, 133.1 (d, *J* = 9.5 Hz), 131.5, 129.4, 128.6, 127.0, 124.5 (d, *J* = 2.9 Hz), 115.6 (d, *J* = 22.1 Hz), 62.9, 51.8, 45.7, 44.8, 31.1, 27.0 ppm. **$^{19}$F NMR** (376 MHz, $CDCl_3$): δ -103.20 ppm. **HRMS** (ESI) *m/z*: $[M+Na]^+$ calcd. for $C_{28}H_{25}ClFNO_4Na$: 516.1354; Found: 516.1352.

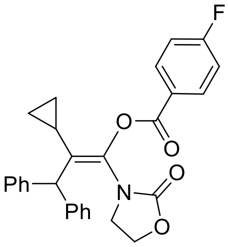


(*E*)-**3pa**
$C_{28}H_{24}FNO_4$
M = 457.50 g/mol

**(*E*)-2-cyclopropyl-1-(2-oxooxazolidin-3-yl)-3,3-diphenylprop-1-en-1-yl 4-fluorobenzoate** ((*E*)-**3pa**): Prepared from 3-(cyclopropylethynyl)oxazolidin-2-one (**1p**, 36.3 mg, 0.24 mmol) and benzhydryl 4-fluorobenzoate (**2a**, 61.3 mg, 0.2 mmol) according to the GP1 with 10 mol% $B(C_6F_5)_3$ at 80 ℃ for 24 h. Purification by flash chromatography on silica gel using petroleum ether/EtOAc (10/1 to 5/1) afforded (*E*)-**3pa** as a white solid (76.9 mg, 84% yield, *E*:*Z* > 96:4).

(*E*)-**3pa**: **R*f*** = 0.45 (petroleum ether/EtOAc = 5/1). **$^1$H NMR** (400 MHz, $CDCl_3$): δ 8.18 (dd, *J* = 8.6, 5.4 Hz, 2H), 7.39-7.28 (m, 8H), 7.23 (t, *J* = 7.0 Hz, 2H), 7.16 (t, *J* = 8.6 Hz, 2H), 5.11 (s, 1H), 3.87 (t, *J* = 7.8 Hz, 2H), 3.36 (t, *J* = 7.6 Hz, 2H), 1.34-1.27 (m, 1H), 0.52-0.39 (m, 4H) ppm. **$^{13}$C NMR** (100 MHz, $CDCl_3$): δ 166.3 (d, *J* = 254.2 Hz),

164.1, 155.7, 141.6, 135.2, 132.9 (d, $J$ = 9.5 Hz), 132.6, 129.3, 128.2, 125.5, 124.7 (d, $J$ = 3.0 Hz), 115.9 (d, $J$ = 21.9 Hz), 62.6, 52.3, 45.0, 12.7, 6.4 ppm. **$^{19}$F NMR** (376 MHz, $CDCl_3$): δ -103.61 ppm. **HRMS** (ESI) *m/z*: $[M+Na]^+$ calcd. for $C_{28}H_{24}FNO_4Na$: 480.1587; Found: 480.1588.

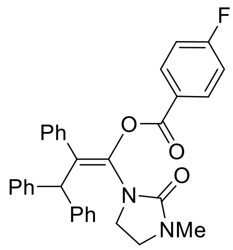


(*Z*)-**3qa**
$C_{32}H_{27}FN_2O_3$
M = 506.58 g/mol

**(*Z*)-1-(3-methyl-2-oxoimidazolidin-1-yl)-2,3,3-triphenylprop-1-en-1-yl 4-fluorobenzoate** ((*Z*)-**3qa**): Prepared from 1-methyl-3-(phenylethynyl)imidazolidin-2-one (**1q**, 48.1 mg, 0.24 mmol) and benzhydryl 4-fluorobenzoate (**2a**, 61.3 mg, 0.2 mmol) according to the GP1 with 5 mol% $B(C_6F_5)_3$ at 80 °C for 12 h. Purification by flash chromatography on silica gel using petroleum ether/EtOAc (10/1 to 5/1) afforded (*Z/E*)-**3qa** as a white solid (91.1 mg, 90% yield, *Z*:*E* = 55:45).

(*Z/E*)-**3qa**: **$R_f$** = 0.30 (petroleum ether/EtOAc = 5/1). (*Z*)-**3qa: $^1$H NMR** (400 MHz, $CDCl_3$): δ7.75 (dd, $J$ = 8.8, 5.6 Hz, 2H), 7.34 (d, $J$ = 7.2 Hz, 4H), 7.26-7.15 (m, 8H), 7.10 (d, $J$ = 7.2 Hz, 1H), 7.06-6.92 (m, 4H), 5.65 (s, 1H), 3.44 (t, $J$ = 8.0 Hz, 2H), 3.11-2.99 (m, 2H), 2.76 (s, 3H) ppm. (*E*)-**3qa: $^1$H NMR** (400 MHz, $CDCl_3$): δ 7.83 (dd, J = 8.4, 5.6 Hz, 2H), 7.26-7.15 (m, 12H), 7.10 (d, J = 7.2 Hz, 1H), 7.06-6.92 (m, 4H), 5.37 (s, 1H), 3.26 (t, $J$ = 8.0 Hz, 2H), 3.11-2.99 (m, 2H), 2.65 (s, 3H) ppm. (*Z/E*)-**3qa: $^{13}$C NMR** (100 MHz, $CDCl_3$): δ 166.0 (d, $J$ = 253.2 Hz), 165.9 (d, $J$ = 253.1 Hz), 164.7, 163.5, 158.5, 158.1, 141.2, 140.7, 138.8, 137.7, 137.0, 136.6, 132.8 (d, $J$ = 9.4 Hz), 132.5 (d, $J$ = 9.4 Hz), 132.4, 129.8, 129.4, 129.3, 129.2, 129.1, 128.2, 128.1, 127.9, 127.4, 127.3, 126.9, 126.3, 124.9 (d, $J$ = 3.1 Hz), 115.4 (d, $J$ = 21.9 Hz), 115.3 (d, $J$ = 21.9 Hz), 54.6, 53.7, 45.3, 44.9, 42.8, 42.6, 31.2, 30.9 ppm. (*Z/E*)-**3qa: $^{19}$F NMR** (376

MHz, $CDCl_3$): δ -104.65, -104.75 ppm. **HRMS** (ESI) *m/z*: $[M+Na]^+$ calcd. for $C_{32}H_{27}FN_2O_3Na$: 529.1904; Found: 529.1906.

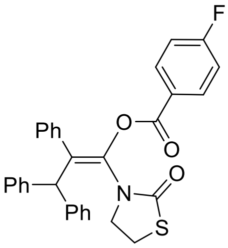

(*Z*)-**3ra**
$C_{31}H_{24}FNO_3S$
M = 509.60 g/mol

**(*Z*)-1-(2-oxothiazolidin-3-yl)-2,3,3-triphenylprop-1-en-1-yl 4-fluorobenzoate** ((*Z*)-**3ra**): Prepared from 3-(phenylethynyl)thiazolidin-2-one (**1r**, 48.8 mg, 0.24 mmol) and benzhydryl 4-fluorobenzoate (**2a**, 61.3 mg, 0.2 mmol) according to the GP1 with 5 mol% $B(C_6F_5)_3$ at 80 ℃ for 12 h. Purification by flash chromatography on silica gel using petroleum ether/EtOAc (10/1 to 5/1) afforded (*Z*)-**3ra** as a white solid (76.4 mg, 75% yield, *Z*:*E* = 94:6).

(*Z*)-**3ra**: $\mathbf{R_f}$ = 0.35 (petroleum ether/EtOAc = 5/1). **$^1$H NMR** (400 MHz, $CDCl_3$): δ 7.76 (dd, *J* = 8.8, 5.4 Hz, 2H), 7.36-7.17 (m, 10H), 7.01-6.94 (m, 7H), 5.54 (s, 1H), 3.72 (s, 2H), 2.99 (t, *J* = 7.2 Hz, 2H) ppm. **$^{13}$C NMR** (100 MHz, $CDCl_3$): δ 172.3, 166.0 (d, *J* = 253.8 Hz), 164.4, 140.7, 135.7, 135.5, 133.9, 132.6 (d, *J* = 9.4 Hz), 129.7, 129.1, 128.2, 127.4, 127.2, 126.6, 124.4 (d, *J* = 2.9 Hz), 115.6 (d, *J* = 21.9 Hz), 53.7, 48.9, 26.7 ppm. **$^{19}$F NMR** (376 MHz, $CDCl_3$): δ -103.92 ppm. **HRMS** (ESI) *m/z*: $[M+Na]^+$ calcd. for $C_{31}H_{24}FNO_3SNa$: 532.1359; Found: 532.1365.

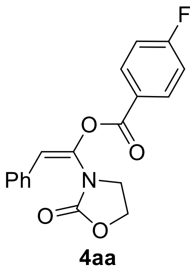

**4aa**
$C_{18}H_{14}FNO_4$
M = 327.31 g/mol

**1-(2-oxooxazolidin-3-yl)-2-phenylvinyl 4-fluorobenzoate** (**4aa**)**:** **R*f*** = 0.35 (petroleum ether/EtOAc = 5/1). **$^1$H NMR** (400 MHz, $CDCl_3$): δ 8.17 (dd, *J* = 8.8, 5.6 Hz, 2H), 7.41-7.35 (m, 4H), 7.33-7.28 (m, 1H), 7.17 (t, *J* = 8.6 Hz, 2H), 6.39 (s, 1H), 4.37 (t, *J* = 8.0 Hz, 2H), 3.78 (t, *J* = 8.0 Hz, 2H) ppm. **$^{13}$C NMR** (100 MHz, $CDCl_3$): δ 166.4 (d, *J* = 254.4 Hz), 164.2, 155.6, 137.6, 133.1 (d, *J* = 9.6 Hz), 131.9, 128.8, 128.2, 128.1, 124.7 (d, *J* = 2.9 Hz), 116.4, 115.9 (d, *J* = 22.0 Hz), 63.2, 44.6 ppm. **$^{19}$F NMR** (376 MHz, $CDCl_3$): δ -103.44 ppm. **HRMS** (ESI) *m/z*: $[M+Na]^+$ calcd. for $C_{18}H_{14}FNO_4Na$: 350.0805; Found: 350.0802.

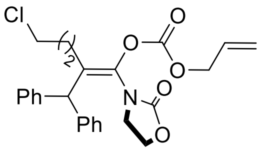


(*E*)-**6oa**
$C_{25}H_{26}ClNO_5$
M = 455.94 g/mol

**(*E*)-allyl (2-benzhydryl-5-chloro-1-(2-oxooxazolidin-3-yl)pent-1-en-1-yl) carbonate** ((*E*)-**6oa**): Prepared from 3-(5-chloropent-1-yn-1-yl)oxazolidin-2-one (**1o**, 45.0 mg, 0.24 mmol) and allyl benzhydryl carbonate (**5a**, 57.3 mg, 0.2 mmol) according to the GP2 with 5 mol% $B(C_6F_5)_3$ at 25 ℃ for 12 h. Purification by flash chromatography on silica gel using petroleum ether/EtOAc (10/1 to 5/1) afforded (*E*)-**6oa** as a white solid (67.5 mg, 74% yield, *E*:*Z* > 96:4).

(*E*)-**6oa**: **R*f*** = 0.30 (petroleum ether/EtOAc = 5/1). **$^1$H NMR** (400 MHz, $CDCl_3$): δ 7.33-7.29 (m, 4H), 7.25-7.23 (m, 6H), 6.01-5.91 (m, 1H), 5.41 (d, *J* = 17.2 Hz, 1H), 5.32 (d, *J* = 10.8 Hz, 1H), 5.24 (s, 1H), 4.72 (d, *J* = 5.6 Hz, 2H), 4.11 (t, *J* = 8.0 Hz, 2H), 3.49 (t, *J* = 8.0 Hz, 2H), 3.23 (t, *J* = 6.2 Hz, 2H), 2.22-2.18 (m, 2H), 1.38-1.31 (m, 2H) ppm. **$^{13}$C NMR** (100 MHz, $CDCl_3$): δ 155.6, 153.2, 140.7, 140.0, 131.4, 130.7, 129.2, 128.4, 126.9, 119.6, 69.4, 62.7, 51.5, 45.2, 44.9, 30.7, 26.8 ppm. **HRMS** (ESI) *m/z*: $[M+Na]^+$ calcd. for $C_{25}H_{26}ClNO_5Na$: 478.1398; Found: 478.1380.

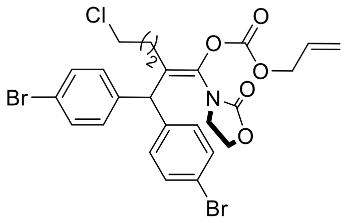

($E$)-**6ob**
$C_{25}H_{24}Br_2ClNO_5$
M = 613.73 g/mol

**(*E*)-allyl (2-(bis(4-bromophenyl)methyl)-5-chloro-1-(2-oxooxazolidin-3-yl)pent-1-en-1-yl) carbonate** (($E$)-**6ob**): Prepared from 3-(5-chloropent-1-yn-1-yl)oxazolidin-2-one (**1o**, 45.0 mg, 0.24 mmol) and allyl (bis(4-bromophenyl)methyl) carbonate (**5b**, 85.2 mg, 0.2 mmol) according to the GP2 with 5 mol% $B(C_6F_5)_3$ at 25 ℃ for 12 h. Purification by flash chromatography on silica gel using petroleum ether/EtOAc (10/1 to 5/1) afforded ($E$)-**6ob** as a colorless oil (83.4 mg, 68% yield, $E$:$Z$ > 96:4).

($E$)-**6ob**: $\mathbf{R_f}$ = 0.30 (petroleum ether/EtOAc = 5/1). **$^1$H NMR** (400 MHz, $CDCl_3$): δ 7.44 (d, $J$ = 8.4 Hz, 4H), 7.12 (d, $J$ = 8.4 Hz, 4H), 6.01-5.91 (m, 1H), 5.42 (d, $J$ = 17.2 Hz, 1H), 5.34 (d, $J$ = 10.4 Hz, 1H), 5.14 (s, 1H), 4.72 (d, $J$ = 6.0 Hz, 2H), 4.18 (t, $J$ = 8.0 Hz, 2H), 3.50 (t, $J$ = 8.0 Hz, 2H), 3.28 (t, $J$ = 6.0 Hz, 2H), 2.18-2.14 (m, 2H), 1.40-1.33 (m, 2H) ppm. **$^{13}$C NMR** (100 MHz, $CDCl_3$): δ 155.6, 153.1, 139.3, 135.3, 131.7, 130.9, 130.6, 130.3, 121.1, 112.0, 69.6, 62.6, 50.4, 44.9, 44.8, 30.7, 26.5 ppm. **HRMS** (ESI) $m/z$: $[M+Na]^+$ calcd. for $C_{25}H_{24}Br_2ClNO_5Na$: 633.9602; Found: 633.9609.

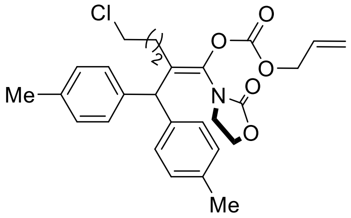

($E$)-**6oc**
$C_{27}H_{30}ClNO_5$
M =488.99 g/mol

**(*E*)-allyl (5-chloro-2-(di-p-tolylmethyl)-1-(2-oxooxazolidin-3-yl)pent-1-en-1-yl) carbonate** (($E$)-**6oc**): Prepared from 3-(5-chloropent-1-yn-1-yl)oxazolidin-2-one (**1o**, 45.0 mg, 0.24 mmol) and allyl (di-$p$-tolylmethyl) carbonate (**5c**, 59.3 mg, 0.2 mmol) according to the GP2 with 5 mol% $B(C_6F_5)_3$ at 25 ℃ for 12 h. Purification by flash chromatography on

silica gel using petroleum ether/EtOAc (10/1 to 5/1) afforded (*E*)-**6oc** as a white solid (58.7 mg, 60% yield, *E*:*Z* > 96:4).

(*E*)-**6oc**: **R*f*** = 0.35 (petroleum ether/EtOAc = 5/1). **$^1$H NMR** (400 MHz, $CDCl_3$): δ 7.14-7.09 (m, 8H), 6.01-5.91 (m, 1H), 5.41 (d, *J* = 17.2 Hz, 1H), 5.32 (d, *J* = 10.4 Hz, 1H), 5.14 (s, 1H), 4.71 (d, *J* = 5.6 Hz, 2H), 4.13 (t, *J* = 8.0 Hz, 2H), 3.52 (t, *J* = 8.0 Hz, 2H), 3.24 (t, *J* = 6.2 Hz, 2H), 2.32 (s, 6H), 2.20-2.16 (m, 2H), 1.39-1.32 (m, 2H) ppm. **$^{13}$C NMR** (100 MHz, $CDCl_3$): δ 155.7, 153.4, 137.8, 136.4, 134.7, 131.7, 130.8, 129.09, 129.08, 119.6, 69.4, 62.7, 50.8, 45.4, 45.0, 30.7, 26.8, 21.0 ppm. **HRMS** (ESI) *m/z*: $[M+Na]^+$ calcd. for $C_{27}H_{30}ClNO_5Na$: 506.1711; Found: 506.1692.

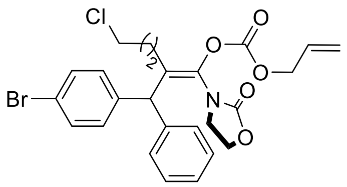

(*E*)-**6od**
$C_{25}H_{25}BrClNO_5$
M =534.83 g/mol

**(*E*)-allyl (2-((4-bromophenyl)(phenyl)methyl)-5-chloro-1-(2-oxooxazolidin-3-yl)pent-1-en-1-yl) carbonate** ((*E*)-**6od**): Prepared from 3-(5-chloropent-1-yn-1-yl)oxazolidin-2-one (**1o**, 45.0 mg, 0.24 mmol) and allyl ((4-bromophenyl)(phenyl)methyl) carbonate (**5d**, 69.4 mg, 0.2 mmol) according to the GP2 with 5 mol% $B(C_6F_5)_3$ at 25 ℃ for 12 h. Purification by flash chromatography on silica gel using petroleum ether/EtOAc (10/1 to 5/1) afforded (*E*)-**6od** as a colorless oil (74.9 mg, 70% yield, *E*:*Z* > 96:4).

(*E*)-**6od**: **R*f*** = 0.30 (petroleum ether/EtOAc = 5/1). **$^1$H NMR** (400 MHz, $CDCl_3$): δ 7.44 (d, *J* = 8.4 Hz, 2H), 7.34-7.25 (m, 3H), 7.21 (d, *J* = 7.6 Hz, 2H), 7.15 (d, *J* = 8.4 Hz, 2H), 6.01-5.91 (m, 1H), 5.42 (d, *J* = 17.2 Hz, 1H), 5.33 (d, *J* = 10.4 Hz, 1H), 5.19 (s, 1H), 4.72 (d, *J* = 6.0 Hz, 2H), 4.15 (t, *J* = 8.0 Hz, 2H), 3.50 (t, *J* = 8.0 Hz, 2H), 3.25 (t, *J* = 6.0 Hz, 2H), 2.18 (t, *J* = 8.0 Hz, 2H), 1.42-1.29 (m, 2H) ppm. **$^{13}$C NMR** (100 MHz, $CDCl_3$): δ 155.6, 153.2, 140.2, 139.8, 135.2, 131.5, 131.0, 130.9, 130.7, 129.1, 128.6, 127.1, 120.9, 119.8, 69.5, 62.7, 51.0, 45.0, 44.8, 30.7, 26.7 ppm. **HRMS** (ESI) *m/z*: $[M+Na]^+$ calcd. for $C_{25}H_{25}BrClNO_5Na$: 556.0497; Found: 556.0499.

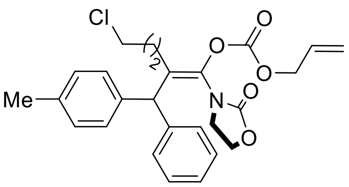

(*E*)-**6oe**
$C_{26}H_{28}ClNO_5$
M =469.96 g/mol

**(*E*)-allyl (5-chloro-1-(2-oxooxazolidin-3-yl)-2-(phenyl(p-tolyl)methyl)pent-1-en-1-yl) carbonate** ((*E*)-**6oe**): Prepared from 3-(5-chloropent-1-yn-1-yl)oxazolidin-2-one (**1o**, 45.0 mg, 0.24 mmol) and allyl (phenyl(p-tolyl)methyl) carbonate (**5e**, 56.5 mg, 0.2 mmol) according to the GP2 with 5 mol% $B(C_6F_5)_3$ at 25 ℃ for 12 h. Purification by flash chromatography on silica gel using petroleum ether/EtOAc (10/1 to 5/1) afforded (*E*)-**6oe** as a white solid (67.7 mg, 72% yield, *E*:*Z* > 96:4).

(*E*)-**6oe**: $\mathbf{R_f}$ = 0.30 (petroleum ether/EtOAc = 5/1). **$^1$H NMR** (400 MHz, $CDCl_3$): δ 7.32-7.28 (m, 2H), 7.26-7.21 (m, 3H), 7.12 (s, 4H), 6.01-5.91 (m, 1H), 5.41 (d, *J* = 17.2 Hz, 1H), 5.32 (d, *J* = 10.4 Hz, 1H), 5.19 (s, 1H), 4.72 (d, *J* = 5.6 Hz, 2H), 4.12 (t, *J* = 8.0 Hz, 2H), 3.51 (t, *J* = 8.0 Hz, 2H), 3.23 (t, *J* = 6.4 Hz, 2H), 2.33 (s, 3H), 2.19 (t, *J* = 8.0 Hz, 2H), 1.41-1.30 (m, 2H) ppm. **$^{13}$C NMR** (100 MHz, $CDCl_3$): δ 155.7, 153.3, 140.9, 137.5, 136.5, 134.8, 131.6, 130.8, 129.2, 129.12, 129.08, 128.4, 126.8, 119.6, 69.4, 62.7, 51.2, 45.3, 44.9, 30.7, 26.8, 21.0 ppm. **HRMS** (ESI) *m/z*: $[M+Na]^+$ calcd. for $C_{26}H_{28}ClNO_5Na$: 492.1554; Found: 492.1535.

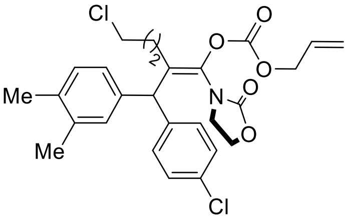

(*E*)-**6of**
$C_{27}H_{29}Cl_2NO_5$
M =518.43 g/mol

**(*E*)-allyl (5-chloro-2-((4-chlorophenyl)(3,4-dimethylphenyl)methyl)-1-(2-oxooxazolidin-3-yl)pent-1-en-1-yl) carbonate** ((*E*)-**6of**): Prepared from 3-(5-chloropent-1-yn-1-yl)oxazolidin-2-one (**1o**, 45.0 mg, 0.24 mmol) and allyl ((4-chlorophenyl)(3,4-dimethylphenyl)methyl) carbonate (**5f**, 66.2 mg, 0.2 mmol) according to the GP2 with

5 mol% $B(C_6F_5)_3$ at 25 ℃ for 12 h. Purification by flash chromatography on silica gel using petroleum ether/EtOAc (10/1 to 5/1) afforded (*E*)-**6of** as a colorless oil (51.8 mg, 50% yield, *E*:*Z* > 96:4).

(*E*)-**6of**: **R*f*** = 0.30 (petroleum ether/EtOAc = 5/1). **$^1$H NMR** (400 MHz, $CDCl_3$): δ 7.27 (d, *J* = 8.4 Hz, 2H), 7.20 (d, *J* = 8.4 Hz, 2H), 7.07 (d, *J* = 7.6 Hz, 1H), 6.95-6.91 (m, 2H), 6.01-5.91 (m, 1H), 5.42 (d, *J* = 17.2 Hz, 1H), 5.33 (d, *J* = 10.4 Hz, 1H), 5.12 (s, 1H), 4.72 (d, *J* = 6.0 Hz, 2H), 4.14 (t, *J* = 8.4 Hz, 2H), 3.50 (t, *J* = 8.0 Hz, 2H), 3.26 (t, *J* = 6.0 Hz, 2H), 2.24-2.13 (m, 8H), 1.40-1.32 (m, 2H) ppm. **$^{13}$C NMR** (100 MHz, $CDCl_3$): δ 155.7, 153.3, 139.6, 137.5, 136.8, 135.4, 135.0, 132.6, 131.2, 130.8, 130.7, 130.4, 129.8, 128.5, 126.4, 119.7, 69.5, 62.7, 50.6, 45.2, 45.0, 30.8, 26.7, 19.8, 19.4 ppm. **HRMS** (ESI) *m/z*: $[M+Na]^+$ calcd. for $C_{27}H_{29}Cl_2NO_5Na$: 540.1321; Found: 540.1304.

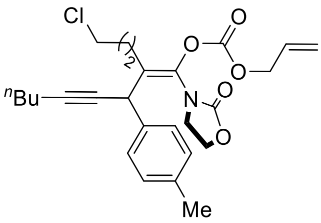


(*E*)-**6oh**
$C_{26}H_{32}ClNO_5$
M =473.99 g/mol

**(*E*)-allyl (2-(3-chloropropyl)-1-(2-oxooxazolidin-3-yl)-3-(p-tolyl)non-1-en-4-yn-1-yl) carbonate** ((*E*)-**6oh**): Prepared from 3-(5-chloropent-1-yn-1-yl)oxazolidin-2-one (**1o**, 45.0 mg, 0.24 mmol) and allyl (1-(*p*-tolyl)hept-2-yn-1-yl) carbonate (**5h**, 57.3 mg, 0.2 mmol) according to the GP2 with 5 mol% $B(C_6F_5)_3$ at 25 ℃ for 12 h. Purification by flash chromatography on silica gel using petroleum ether/EtOAc (10/1 to 5/1) afforded (*E*)-**6oh** as a colorless oil (72.0 mg, 76% yield, *E*:*Z* > 96:4).

(*E*)-**6oh**: **R*f*** = 0.35 (petroleum ether/EtOAc = 5/1). **$^1$H NMR** (400 MHz, $CDCl_3$): δ 7.36 (d, *J* = 7.6 Hz, 2H), 7.13 (d, *J* = 7.6 Hz, 2H), 6.00-5.90 (m, 1H), 5.40 (d, *J* = 17.2 Hz, 1H), 5.31 (d, *J* = 10.4 Hz, 1H), 4.73 (s, 1H), 4.69 (d, *J* = 6.0 Hz, 2H), 4.44-4.31 (m, 2H), 3.96-3.86 (m, 2H), 3.44-3.36 (m, 2H), 2.33 (s, 3H), 2.27 (t, *J* = 6.8 Hz, 2H), 2.05-1.98 (m, 1H), 1.93-1.86 (m, 1H), 1.69-1.62 (m, 2H), 1.57-1.50 (m, 2H), 1.48-1.39 (m,

2H), 0.93 (t, $J$ = 7.2 Hz, 3H) ppm. **$^{13}$C NMR** (100 MHz, $CDCl_3$): δ 155.7, 153.0, 136.9, 134.6, 134.4, 130.8, 129.8, 129.2, 127.7, 119.6, 85.6, 77.4, 69.4, 62.9, 46.0, 44.7, 37.8, 31.2, 30.8, 25.4, 22.0, 21.0, 18.5, 13.5 ppm. **HRMS** (ESI) *m/z*: $[M+Na]^+$ calcd. for $C_{26}H_{32}ClNO_5Na$: 496.1861; Found: 496.1868.

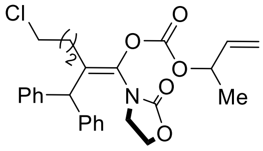


(*E*)-**6oi**
$C_{26}H_{28}ClNO_5$
M =469.96 g/mol

**(*E*)-2-benzhydryl-5-chloro-1-(2-oxooxazolidin-3-yl)pent-1-en-1-yl but-3-en-2-yl carbonate** ((*E*)-**6oi**): Prepared from 3-(5-chloropent-1-yn-1-yl)oxazolidin-2-one (**1o**, 45.0 mg, 0.24 mmol) and benzhydryl but-3-en-2-yl carbonate (**5i**, 56.5 mg, 0.2 mmol) according to the GP2 with 5 mol% $B(C_6F_5)_3$ at 25 ℃ for 12 h. Purification by flash chromatography on silica gel using petroleum ether/EtOAc (10/1 to 5/1) afforded (*E*)-**6oi** as a colorless oil (69.5 mg, 74% yield, *E*:*Z* > 96:4).

(*E*)-**6oi**: **$R_f$** = 0.35 (petroleum ether/EtOAc = 5/1). **$^1$H NMR** (400 MHz, $CDCl_3$): δ 7.33-7.29 (m, 4H), 7.25-7.22 (m, 6H), 5.94-5.85 (m, 1H), 5.35 (d, $J$ = 17.2 Hz, 1H), 5.28-5.21 (m, 3H), 4.10 (t, $J$ = 8.0 Hz, 2H), 3.47 (t, $J$ = 8.0 Hz, 2H), 3.22 (t, $J$ = 6.2 Hz, 2H), 2.21-2.17 (m, 2H), 1.43 (d, $J$ = 6.8 Hz, 3H), 1.39-1.32 (m, 2H) ppm. **$^{13}$C NMR** (100 MHz, $CDCl_3$): δ 155.6, 152.7, 140.8, 136.4, 135.0, 131.3, 129.3, 128.4, 126.9, 117.2, 76.7, 62.6, 51.6, 45.1, 44.9, 30.7, 26.9, 19.9 ppm. **HRMS** (ESI) *m/z*: $[M+Na]^+$ calcd. for $C_{26}H_{28}ClNO_5Na$: 492.1548; Found: 492.1557.

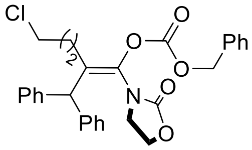


(*E*)-**6oj**
$C_{29}H_{28}ClNO_5$
M =506.00 g/mol

**(*E*)-2-benzhydryl-5-chloro-1-(2-oxooxazolidin-3-yl)pent-1-en-1-yl benzyl carbonate** ((*E*)-**6oj**): Prepared from 3-(5-chloropent-1-yn-1-yl)oxazolidin-2-one (**1o**, 45.0 mg, 0.24 mmol)

and benzhydryl benzyl carbonate (**5j**, 63.7 mg, 0.2 mmol) according to the GP2 with 5 mol% $B(C_6F_5)_3$ at 25 ℃ for 12 h. Purification by flash chromatography on silica gel using petroleum ether/EtOAc (10/1 to 5/1) afforded (*E*)-**6oj** as a colorless oil (41.5 mg, 41% yield, *E*:*Z* > 96:4).

(*E*)-**6oj**: **R*f*** = 0.40 (petroleum ether/EtOAc = 5/1). **$^1$H NMR** (400 MHz, $CDCl_3$): δ 7.42-7.35 (m, 5H), 7.33-7.28 (m, 4H), 7.25-7.22 (m, 6H), 5.25 (s, 2H), 5.23 (s, 1H), 4.07 (t, *J* = 8.0 Hz, 2H), 3.44 (t, *J* = 8.0 Hz, 2H), 3.16 (t, *J* = 6.2 Hz, 2H), 2.18-2.14 (m, 2H), 1.34-1.26 (m, 2H) ppm. **$^{13}$C NMR** (100 MHz, $CDCl_3$): δ 155.6, 153.4, 140.7, 135.0, 134.5, 131.4, 129.3, 128.8, 128.7, 128.4, 128.3, 126.9, 70.7, 62.6, 51.6, 45.1, 44.9, 30.7, 26.9 ppm. **HRMS** (ESI) *m/z*: $[M+Na]^+$ calcd. for $C_{29}H_{28}ClNO_5Na$: 528.1554; Found: 528.1539.

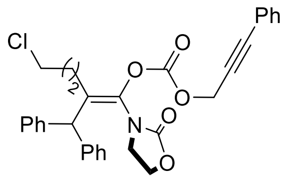


(*E*)-**6ok**
$C_{31}H_{28}ClNO_5$
M =530.02 g/mol

**(*E*)-2-benzhydryl-5-chloro-1-(2-oxooxazolidin-3-yl)pent-1-en-1-yl (3-phenylprop-2-yn-1-yl) carbonate** ((*E*)-**6ok**): Prepared from 3-(5-chloropent-1-yn-1-yl)oxazolidin-2-one (**1o**, 45.0 mg, 0.24 mmol) and benzhydryl (3-phenylprop-2-yn-1-yl) carbonate (**5k**, 68.5 mg, 0.2 mmol) according to the GP2 with 5 mol% $B(C_6F_5)_3$ at 25 ℃ for 12 h. Purification by flash chromatography on silica gel using petroleum ether/EtOAc (10/1 to 5/1) afforded (*E*)-**6ok** as a colorless oil (42.4 mg, 40% yield, *E*:*Z* > 96:4).

(*E*)-**6ok**: **R*f*** = 0.35 (petroleum ether/EtOAc = 5/1). **$^1$H NMR** (400 MHz, $CDCl_3$): δ 7.45 (d, *J* = 8.0 Hz, 1H), 7.35-7.29 (m, 7H), 7.26-7.21 (m, 7H), 5.24 (s, 1H), 5.05 (s, 2H), 4.09 (t, *J* = 8.0 Hz, 2H), 3.49 (t, *J* = 8.0 Hz, 2H), 3.22 (t, *J* = 6.0 Hz, 2H), 2.24-2.20 (m, 2H), 1.40-1.33 (m, 2H) ppm. **$^{13}$C NMR** (100 MHz, $CDCl_3$): δ 155.6, 153.1, 140.7, 135.1, 131.9, 131.8, 129.3, 129.1, 128.5, 128.4, 127.0, 121.7, 88.0, 81.5, 62.7, 57.3,

51.7, 45.2, 44.9, 30.8, 27.0 ppm. **HRMS** (ESI) *m/z*: $[M+Na]^+$ calcd. for $C_{31}H_{28}ClNO_5Na$: 552.1548; Found: 552.1557.

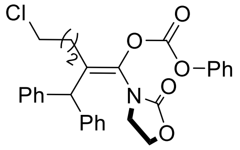


(*E*)-**6ol**
$C_{28}H_{26}ClNO_5$
M =491.97 g/mol

**(*E*)-2-benzhydryl-5-chloro-1-(2-oxooxazolidin-3-yl)pent-1-en-1-yl phenyl carbonate** ((*E*)-**6ol**): Prepared from 3-(5-chloropent-1-yn-1-yl)oxazolidin-2-one (**1o**, 45.0 mg, 0.24 mmol) and benzhydryl phenyl carbonate (**5l**, 60.9 mg, 0.2 mmol) according to the GP2 with 5 mol% $B(C_6F_5)_3$ at 25 ℃ for 12 h. Purification by flash chromatography on silica gel using petroleum ether/EtOAc (10/1 to 5/1) afforded (*E*)-**6ol** as a colorless oil (62.9 mg, 64% yield, *E*:*Z* > 96:4).

(*E*)-**6ol**: **R*f*** = 0.40 (petroleum ether/EtOAc = 5/1). **$^1$H NMR** (400 MHz, $CDCl_3$): δ 7.42 (t, *J* = 7.8 Hz, 2H), 7.33-7.24 (m, 13H), 5.26 (s, 1H), 4.15 (t, *J* = 8.0 Hz, 2H), 3.55 (t, *J* = 8.0 Hz, 2H), 3.27 (t, *J* = 6.0 Hz, 2H), 2.30-2.26 (m, 2H), 1.42-1.35 (m, 2H) ppm. **$^{13}$C NMR** (100 MHz, $CDCl_3$): δ 155.7, 152.4, 150.8, 140.5, 135.0, 132.1, 129.6, 129.2, 128.5, 127.0, 126.6, 120.8, 62.8, 51.6, 45.5, 45.0, 30.7, 27.0 ppm. **HRMS** (ESI) *m/z*: $[M+Na]^+$ calcd. for $C_{28}H_{26}ClNO_5Na$: 514.1398; Found: 514.1382.

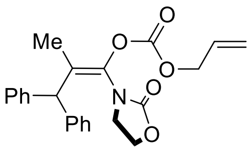


(*E*)-**6sa**
$C_{23}H_{23}NO_5$
M =393.44 g/mol

**(*E*)-allyl (2-methyl-1-(2-oxooxazolidin-3-yl)-3,3-diphenylprop-1-en-1-yl) carbonate** ((*E*)-**6sa**): Prepared from 3-(prop-1-yn-1-yl)oxazolidin-2-one (**1s**, 30.0 mg, 0.24 mmol) and allyl benzhydryl carbonate (**5a**, 53.7 mg, 0.2 mmol) according to the GP2 with 5 mol% $B(C_6F_5)_3$ at 25 ℃ for 12 h. Purification by flash chromatography on silica gel using

petroleum ether/EtOAc (10/1 to 5/1) afforded (*E*)-**6sa** as a white solid (63.0 mg, 80% yield, *E*:*Z* > 96:4).

(*E*)-**6sa**: $\mathbf{R}_f$ = 0.40 (petroleum ether/EtOAc = 5/1). **$^1$H NMR** (400 MHz, $CDCl_3$): δ 7.33-7.29 (m, 4H), 7.26-7.22 (m, 6H), 6.01-5.91 (m, 1H), 5.41 (dd, *J* = 17.2, 1.2 Hz, 1H), 5.31 (dd, *J* = 10.4, 1.2 Hz, 1H), 5.22 (s, 1H), 4.71 (dt, *J* = 5.6, 1.2 Hz, 2H), 4.15 (t, *J* = 8.0 Hz, 2H), 3.50 (t, *J* = 8.0 Hz, 2H), 1.60 (s, 3H) ppm. **$^{13}$C NMR** (100 MHz, $CDCl_3$): δ 155.8, 153.2, 141.0, 133.8, 130.8, 129.1, 128.9, 128.4, 126.7, 119.4, 69.3, 62.7, 51.6, 45.1, 14.7 ppm. **HRMS** (ESI) *m/z*: $[M+Na]^+$ calcd. for $C_{23}H_{23}NO_5Na$: 416.1474; Found: 416.1460.

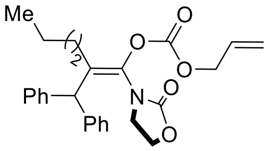


(*E*)-**6ta**
$C_{26}H_{29}NO_5$
M =435.52 g/mol

**(*E*)-allyl (2-benzhydryl-1-(2-oxooxazolidin-3-yl)hex-1-en-1-yl) carbonate** ((*E*)-**6ta**): Prepared from 3-(hex-1-yn-1-yl)oxazolidin-2-one (**1t**, 40.1 mg, 0.24 mmol) and allyl benzhydryl carbonate (**5a**, 53.7 mg, 0.2 mmol) according to the GP2 with 5 mol% $B(C_6F_5)_3$ at 25 ℃ for 12 h. Purification by flash chromatography on silica gel using petroleum ether/EtOAc (10/1 to 5/1) afforded (*E*)-**6ta** as a colorless oil (73.9 mg, 85% yield, *E*:*Z* > 96:4).

(*E*)-**6ta**: $\mathbf{R}_f$ = 0.40 (petroleum ether/EtOAc = 5/1). **$^1$H NMR** (400 MHz, $CDCl_3$): δ 7.32-7.28 (m, 4H), 7.25-7.21 (m, 6H), 6.01-5.91 (m, 1H), 5.41 (dd, *J* = 17.2, 1.2 Hz, 1H), 5.32 (dd, *J* = 10.4, 1.2 Hz, 1H), 5.21 (s, 1H), 4.71 (d, *J* = 5.6 Hz, 2H), 4.05 (t, *J* = 8.0 Hz, 2H), 3.45 (t, *J* = 8.0 Hz, 2H), 2.02-1.98 (m, 2H), 1.08-0.94 (m, 4H), 0.66 (t, *J* = 7.2 Hz, 3H) ppm. **$^{13}$C NMR** (100 MHz, $CDCl_3$): δ 155.6, 153.5, 141.0, 134.3, 133.0, 130.9, 129.3, 128.3, 126.7, 119.4, 69.2, 62.6, 51.7, 45.2, 30.0, 29.3, 22.7, 13.4 ppm. **HRMS** (ESI) *m/z*: $[M+Na]^+$ calcd. for $C_{26}H_{29}NO_5Na$: 458.1944; Found: 458.1927.

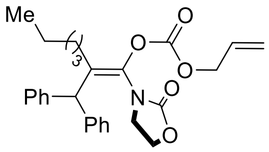

(*E*)-**6na**
$C_{27}H_{31}NO_5$
M =449.55 g/mol

**(*E*)-allyl (2-benzhydryl-1-(2-oxooxazolidin-3-yl)hept-1-en-1-yl) carbonate** ((*E*)-**6na**): Prepared from 3-(hept-1-yn-1-yl)oxazolidin-2-one (**1n**, 43.5 mg, 0.24 mmol) and allyl benzhydryl carbonate (**5a**, 53.7 mg, 0.2 mmol) according to the GP2 with 5 mol% $B(C_6F_5)_3$ at 25 ℃ for 12 h. Purification by flash chromatography on silica gel using petroleum ether/EtOAc (10/1 to 5/1) afforded (*E*)-**6na** as a colorless oil (71.9 mg, 80% yield, *E*:*Z* > 96:4).

(*E*)-**6na**: $\mathbf{R}_f$ = 0.40 (petroleum ether/EtOAc = 5/1). **$^1$H NMR** (400 MHz, $CDCl_3$): δ 7.31-7.21 (m, 10H), 6.01-5.91 (m, 1H), 5.41 (d, *J* = 17.2 Hz, 1H), 5.31 (d, *J* = 10.4 Hz, 1H), 5.21 (s, 1H), 4.71 (d, *J* = 5.6 Hz, 2H), 4.05 (t, *J* = 8.0 Hz, 2H), 3.45 (t, *J* = 8.0 Hz, 2H), 2.01-1.97 (m, 2H), 1.09-0.99 (m, 6H), 0.72 (t, J = 6.8 Hz, 3H) ppm. **$^{13}$C NMR** (100 MHz, $CDCl_3$): δ 155.6, 153.5, 141.1, 134.4, 133.1, 130.9, 129.4, 128.3, 126.7, 119.4, 69.3, 62.6, 51.8, 45.3, 31.9, 29.7, 27.6, 21.9, 13.8 ppm. **HRMS** (ESI) *m/z*: $[M+Na]^+$ calcd. for $C_{27}H_{31}NO_5Na$: 472.2100; Found: 472.2088.

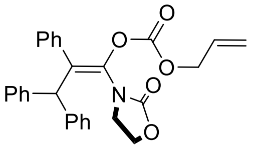

(*Z*)-**6aa**
$C_{28}H_{25}NO_5$
M =455.51 g/mol

**(*Z*)-allyl (1-(2-oxooxazolidin-3-yl)-2,3,3-triphenylprop-1-en-1-yl) carbonate** ((*Z*)-**6aa**): Prepared from 3-(phenylethynyl)oxazolidin-2-one (**1a**, 44.9 mg, 0.24 mmol) and allyl benzhydryl carbonate (**5a**, 53.7 mg, 0.2 mmol) according to the GP2 with 5 mol% $B(C_6F_5)_3$ at 25 ℃ for 12 h. Purification by flash chromatography on silica gel using petroleum ether/EtOAc (10/1 to 5/1) afforded (*Z*)-**6aa** as a colorless oil (36.4 mg, 40% yield, *Z*:*E* > 96:4).

(*Z*)-**6aa**: **R*f*** = 0.35 (petroleum ether/EtOAc = 5/1). **$^1$H NMR** (400 MHz, $CDCl_3$): δ 7.29-7.22 (m, 8H), 7.19 (d, *J* = 7.2 Hz, 2H), 7.13-7.11 (m, 3H), 7.07-7.03 (m, 2H), 5.71-5.61 (m, 1H), 5.53 (s, 1H), 5.15-5.07 (m, 2H), 4.44 (d, *J* = 5.6 Hz, 2H), 4.00 (t, *J* = 8.0 Hz, 2H), 3.48 (t, *J* = 8.0 Hz, 2H) ppm. **$^{13}$C NMR** (100 MHz, $CDCl_3$): δ 155.4, 153.1, 140.7, 135.7, 135.1, 134.0, 130.6, 129.6, 129.0, 128.2, 127.7, 127.4, 126.6, 118.9, 69.1, 62.6, 53.9, 44.6 ppm. **HRMS** (ESI) *m/z*: $[M+Na]^+$ calcd. for $C_{28}H_{25}NO_5Na$: 478.1631; Found: 478.1617.

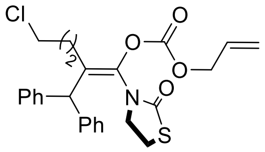


(*E*)-**6ua**
$C_{25}H_{26}ClNO_4S$
M =472.00 g/mol

**(*E*)-allyl (2-benzhydryl-5-chloro-1-(2-oxothiazolidin-3-yl)pent-1-en-1-yl) carbonate** ((*E*)-**6ua**): Prepared from 3-(5-chloropent-1-yn-1-yl)thiazolidin-2-one (**1u**, 48.9 mg, 0.24 mmol) and allyl benzhydryl carbonate (**5a**, 53.7 mg, 0.2 mmol) according to the GP2 with 5 mol% $B(C_6F_5)_3$ at 25 ℃ for 12 h. Purification by flash chromatography on silica gel using petroleum ether/EtOAc (10/1 to 5/1) afforded (*E*)-**6ua** as a colorless oil (79.3 mg, 84% yield, *E*:*Z* > 96:4).

(*E*)-**6ua**: **R*f*** = 0.35 (petroleum ether/EtOAc = 5/1). **$^1$H NMR** (400 MHz, $CDCl_3$): δ 7.32-7.28 (m, 4H), 7.26-7.21 (m, 6H), 6.01-5.91 (m, 1H), 5.41 (d, *J* = 17.2 Hz, 1H), 5.32 (d, *J* = 10.4 Hz, 1H), 5.18 (s, 1H), 4.71 (d, *J* = 5.6 Hz, 2H), 3.74-3.36 (m, 2H), 3.20 (t, *J* = 6.2 Hz, 2H), 3.04 (t, *J* = 6.8 Hz, 2H), 2.19 (s, 2H), 1.53-0.98 (m, 2H) ppm. **$^{13}$C NMR** (100 MHz, $CDCl_3$): δ 171.9, 153.2, 140.8, 135.4, 131.2, 130.8, 129.3, 128.5, 126.9, 119.5, 69.4, 51.5, 48.9, 44.9, 30.7, 26.8, 26.7 ppm. **HRMS** (ESI) *m/z*: $[M+Na]^+$ calcd. for $C_{25}H_{26}ClNO_4Na$: 494.1163; Found: 494.1168.

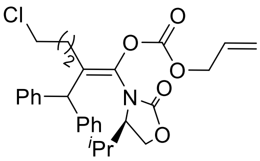

(*E*)-**6va**
$C_{28}H_{32}ClNO_5$
M =498.02 g/mol

**(*R*,*E*)-allyl (2-benzhydryl-5-chloro-1-(4-isopropyl-2-oxooxazolidin-3-yl)pent-1-en-1-yl) carbonate** ((*E*)-**6va**): Prepared from (*R*)-3-(5-chloropent-1-yn-1-yl)-4-isopropyloxazolidin-2-one (**1v**, 55.1 mg, 0.24 mmol) and allyl benzhydryl carbonate (**5a**, 53.7 mg, 0.2 mmol) according to the GP2 with 5 mol% $B(C_6F_5)_3$ at 25 ℃ for 12 h. Purification by flash chromatography on silica gel using petroleum ether/EtOAc (10/1 to 5/1) afforded (*E*)-**6va** as a colorless oil (49.8 mg, 50% yield, *E*:*Z* > 96:4).

(*E*)-**6va**: **$R_f$** = 0.30 (petroleum ether/EtOAc = 5/1). **$^1$H NMR** (400 MHz, $CDCl_3$): δ 7.36 (d, *J* = 7.6 Hz, 2H), 7.31-7.21 (m, 6H), 7.17 (d, *J* = 7.6 Hz, 2H), 6.02-5.91 (m, 1H), 5.43 (d, *J* = 16.4 Hz, 2H), 5.33 (d, *J* = 10.4 Hz, 1H), 4.72 (d, *J* = 6.0 Hz, 2H), 4.18 (d, *J* = 8.0 Hz, 1H), 4.05 (t, *J* = 7.4 Hz, 1H), 3.72 (s, 1H), 3.23-3.17 (m, 1H), 3.12-3.05 (m, 1H), 2.56-2.39 (m, 1H), 2.11 (s, 1H), 1.87-1.76 (m, 1H), 1.37-1.25 (m, 1H), 1.04-0.85 (m, 1H), 0.69 (d, *J* = 7.2 Hz, 3H), 0.41 (s, 3H) ppm. **$^{13}$C NMR** (100 MHz, $CDCl_3$): δ 156.0, 152.6, 141.5, 141.3, 134.5, 131.3, 130.8, 130.1, 129.0, 128.21, 128.19, 126.8, 126.6, 119.9, 69.5, 63.2, 59.8, 50.5, 45.0, 30.8, 28.6, 26.8, 18.3, 13.9 ppm. **HRMS** (ESI) *m/z*: $[M+Na]^+$ calcd. for $C_{28}H_{32}ClNO_5Na$: 520.1861; Found: 520.1866.

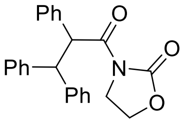

**8**
$C_{24}H_{21}NO_3$
M = 371.44 g/mol

**3-(2,3,3-triphenylpropanoyl)oxazolidin-2-one** (**8**): **$R_f$** = 0.50 (petroleum ether/EtOAc = 5/1). **$^1$H NMR** (400 MHz, $CDCl_3$): δ 7.44 (d, *J* = 7.2 Hz, 2H), 7.38 (d, *J* = 7.2 Hz, 2H), 7.28 (t, *J* = 7.6 Hz, 2H), 7.18-7.03 (m, 8H), 6.97 (t, *J* = 7.2 Hz, 1H), 6.19 (d, *J* = 12.0 Hz, 1H), 4.82 (d, *J* = 12.4 Hz, 1H), 4.14 (t, *J* = 8.0 Hz, 2H), 3.78-3.65 (m, 2H) ppm. **$^{13}$C**

**NMR** (100 MHz, $CDCl_3$): δ 173.0, 153.3, 143.1, 141.4, 136.5, 129.4, 128.7, 128.3, 128.3, 128.1, 127.6, 127.4, 126.6, 126.2, 61.6, 55.3, 52.3, 42.7 ppm. **HRMS** (ESI) *m/z*: $[M+Na]^+$ calcd. for $C_{24}H_{21}NO_3Na$: 394.1419; Found: 394.1416.

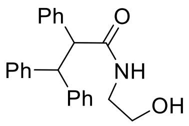

**9**

$C_{23}H_{23}NO_2$

M = 345.44 g/mol

***N*-(2-hydroxyethyl)-2,3,3-triphenylpropanamide** (**9**): **$R_f$** = 0.30 (petroleum ether/EtOAc = 1/1). **$^1$H NMR** (400 MHz, $CDCl_3$): δ 7.44 (d, *J* = 7.2 Hz, 2H), 7.35-7.26 (m, 4H), 7.22-7.07 (m, 8H), 7.01 (d, *J* = 5.6 Hz, 1H), 5.95 (s, 1H), 4.83 (d, *J* = 11.6 Hz, 1H), 4.13 (d, *J* = 12.4 Hz, 1H), 3.31-3.28 (m, 3H), 2.97-2.92 (m, 1H), 1.79 (s, 1H) ppm. **$^{13}$C NMR** (100 MHz, $CDCl_3$): δ 173.0, 143.3, 141.4, 137.7, 128.6, 128.4, 128.3, 128.2, 128.1, 127.2, 126.6, 126.2, 61.8, 58.8, 54.4, 42.3 ppm. **HRMS** (ESI) *m/z*: $[M+Na]^+$ calcd. for $C_{23}H_{23}NO_2Na$: 368.1627; Found: 368.1626.

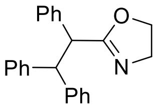

**10**

$C_{23}H_{21}NO$

M = 327.43 g/mol

**2-(1,2,2-triphenylethyl)-4,5-dihydrooxazole** (**10**): **$R_f$** = 0.30 (petroleum ether/EtOAc = 3/1). **$^1$H NMR** (400 MHz, $CDCl_3$): δ 7.42 (d, *J* = 7.6 Hz, 2H), 7.37-7.23 (m, 5H), 7.17 (t, *J* = 6.8 Hz, 3H), 7.12 (d, *J* = 7.2 Hz, 1H), 7.07 (d, *J* = 4.0 Hz, 3H), 7.01-6.96 (m, 1H), 4.71 (d, *J* = 12.4 Hz, 1H), 4.54 (d, *J* = 12.0 Hz, 1H), 4.09-4.03 (m, 1H), 3.97-3.90 (m, 1H), 3.58 (t, *J* = 9.0 Hz, 2H) ppm. **$^{13}$C NMR** (100 MHz, $CDCl_3$): δ 168.0, 142.9, 141.9, 138.1, 128.7, 128.4, 128.3, 128.24, 128.16, 127.9, 127.0, 126.4, 126.1, 67.3, 54.9, 54.0, 50.3 ppm. **HRMS** (ESI) *m/z*: $[M+Na]^+$ calcd. for $C_{23}H_{21}NONa$: 328.1701; Found: 328.1699.

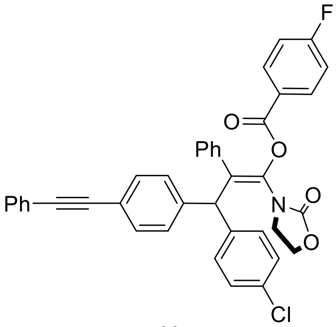


**11**
$C_{39}H_{27}ClFNO_4$
M =628.10 g/mol

**(*Z*)-3-(4-chlorophenyl)-1-(2-oxooxazolidin-3-yl)-2-phenyl-3-(4-(phenylethynyl)phenyl)prop-1-en-1-yl 4-fluorobenzoate** (**11**): **R*f*** = 0.40 (petroleum ether/EtOAc = 5/1). **$^{1}$H NMR** (400 MHz, $CDCl_3$): δ 7.76 (dd, *J* = 8.0, 5.6 Hz, 2H), 7.54-7.49 (m, 2H), 7.45 (d, *J* = 8.0 Hz, 2H), 7.36-7.30 (m, 5H), 7.28-7.22 (m, 4H), 7.02-6.96 (m, 7H), 5.58 (s, 1H), 4.11 (t, *J* = 8.0 Hz, 2H), 3.60 (t, *J* = 7.9 Hz, 2H) ppm. **$^{13}$C NMR** (100 MHz, $CDCl_3$): δ 166.1 (d, *J* = 254.2 Hz), 164.4, 155.8, 140.7, 138.6, 135.3, 135.25, 133.0, 132.6 (d, *J* = 9.5 Hz), 132.59, 131.5, 131.46, 131.0, 129.5, 128.8, 128.31, 128.28, 128.2, 127.7, 127.5, 124.1 (d, *J* = 2.9 Hz), 123.0, 121.7, 115.6 (d, *J* = 22.0 Hz), 89.6, 89.0, 62.8, 53.0, 44.9 ppm. **$^{19}$F NMR** (376 MHz, $CDCl_3$): -103.43 ppm. **HRMS** (ESI) *m/z*: $[M+Na]^+$ calcd. for $C_{39}H_{27}ClFNO_4Na$: 650.1511; Found: 650.1512.

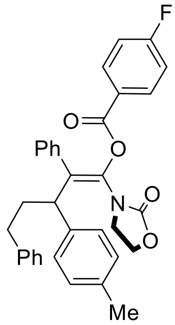


(*Z*)-**12**
$C_{34}H_{30}FNO_4$
M =535.62 g/mol

**(*Z*)-1-(2-oxooxazolidin-3-yl)-2,5-diphenyl-3-(p-tolyl)pent-1-en-1-yl 4-fluorobenzoate** ((*Z*)-**12**): **R*f*** = 0.40 (petroleum ether/EtOAc = 5/1). **$^{1}$H NMR** (400 MHz, $CDCl_3$): δ 7.65 (dd, *J* = 8.8, 5.4 Hz, 2H), 7.30-7.25 (m, 2H), 7.20 (d, *J* = 7.2 Hz, 1H), 7.16-7.05 (m, 7H), 7.02 (d, *J* = 8.0 Hz, 2H), 6.92 (t, *J* = 8.6 Hz, 2H), 6.80-6.78 (m, 2H), 4.26-4.13 (m, 2H),

4.01 (dd, *J* = 9.4, 5.8 Hz, 1H), 3.99-3.80 (m, 1H), 3.60-3.54 (m, 1H), 2.74 -2.59 (m, 2H), 2.33 (s, 3H), 2.25-2.18 (m, 1H), 2.12-2.03 (m, 1H) ppm. **$^{13}$C NMR** (100 MHz, $CDCl_3$): δ 166.0 (d, *J* = 253.7 Hz), 164.6, 158.2, 141.8, 136.4, 136.3, 135.9, 134.3, 133.3, 132.5 (d, *J* = 9.5 Hz), 128.93, 128.89, 128.71, 128.67, 128.3, 127.4, 127.3, 125.8, 124.5 (d, *J* = 3.0 Hz), 115.5 (d, *J* = 22.0 Hz), 62.8, 46.0, 44.8, 33.5, 33.3, 21.0 ppm. **$^{19}$F NMR** (376 MHz, $CDCl_3$): -104.14 ppm. **HRMS** (ESI) *m/z*: $[M+Na]^+$ calcd. for $C_{34}H_{30}FNO_4Na$: 558.2057; Found: 558.2063.

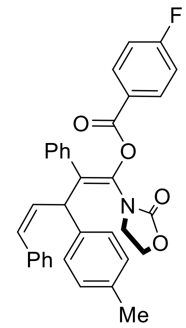


(*Z*)-**13**
$C_{34}H_{28}FNO_4$
M =533.60 g/mol

**(1*Z*,4*Z*)-1-(2-oxooxazolidin-3-yl)-2,5-diphenyl-3-(p-tolyl)penta-1,4-dien-1-yl 4-fluorobenzoate** ((*Z*)-**13**): **$R_f$** = 0.40 (petroleum ether/EtOAc = 5/1). **$^1$H NMR** (400 MHz, $CDCl_3$): δ 7.73 (s, 2H), 7.34-7.24 (m, 7H), 7.16 (d, *J* = 6.8 Hz, 2H), 7.10 (s, 3H), 6.97-6.89 (m, 4H), 6.74 (d, *J* = 11.2 Hz, 1H), 5.92 (t, *J* = 11.0 Hz, 1H), 5.29 (d, *J* = 10.8 Hz, 1H), 3.99-3.97 (m, 1H), 3.81-3.75 (m, 1H), 3.58-3.52 (m, 1H), 3.25-3.23 (m, 1H), 2.36 (s, 3H) ppm. **$^{13}$C NMR** (100 MHz, $CDCl_3$): δ 166.0 (d, *J* = 253.6 Hz), 164.7, 156.2, 137.0, 136.7, 136.6, 134.3, 134.2, 134.17, 132.6 (d, *J* = 9.5 Hz), 130.7, 129.7, 129.3, 128.9, 128.5, 128.48, 128.3, 127.6, 127.5, 127.2, 124.5 (d, *J* = 2.7 Hz), 115.5 (d, *J* = 22.0 Hz), 62.8, 46.5, 44.9, 21.1ppm. **$^{19}$F NMR** (376 MHz, $CDCl_3$): -104.10 ppm. **HRMS** (ESI) *m/z*: $[M+Na]^+$ calcd. for $C_{34}H_{28}FNO_4Na$: 556.1900; Found: 556.1900.

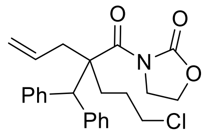


**14**
$C_{24}H_{26}ClNO_3$
M =411.93 g/mol

**3-(2-benzhydryl-2-(3-chloropropyl)pent-4-enoyl)oxazolidin-2-one** (**14**): **$R_f$** = 0.45 (petroleum ether/EtOAc = 5/1). **$^1$H NMR** (400 MHz, $CDCl_3$): δ 7.35-7.19 (m, 10H), 5.46-5.36 (m, 1H), 5.36 (s, 1H), 5.00 (d, *J* = 17.2 Hz, 1H), 4.91 (d, *J* = 10.0 Hz, 1H), 4.06-4.00 (m, 1H), 3.94-3.88 (m, 1H), 3.70-3.63 (m, 1H), 3.52-3.37 (m, 4H), 2.81-2.74 (m, 1H), 2.48 (dd, *J* = 14.6, 6.6 Hz, 1H), 1.81 (td, *J* = 12.8, 4.0 Hz, 1H), 1.65-1.47 (m, 2H) ppm. **$^{13}$C NMR** (100 MHz, $CDCl_3$): δ 175.1, 152.2, 141.4, 140.8, 135.1, 129.9, 129.5, 128.4, 128.3, 126.8, 126.7, 117.9, 62.1, 56.2, 55.3, 45.4, 45.0, 39.8, 32.9, 28.9 ppm. **HRMS** (ESI) *m/z*: $[M+Na]^+$ calcd. for $C_{24}H_{26}ClNO_3Na$: 434.1499; Found: 434.1486.

$[\alpha]_D^{20.0}$ = -35.86 (c = 0.30, $CHCl_3$); *er* was determined to be 94.5:5.5 by HPLC analysis with a Chiralcel AD-H column (hexane/isopropyl alcohol = 98/2, flow rate = 0.4 mL/min, λ = 220 nm), $t_r$ = 45.97 min (minor), $t_r$ = 48.96min (major).

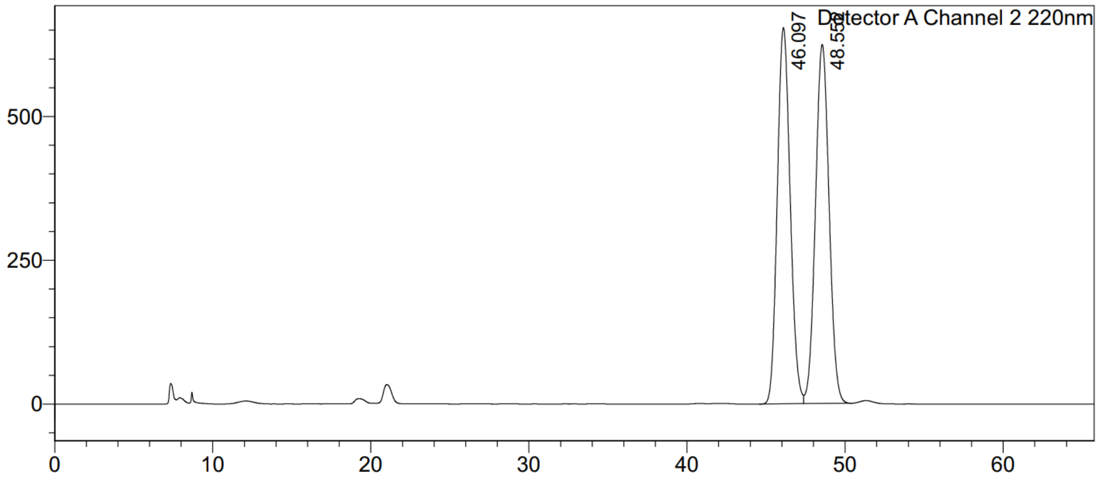


Detector A Channel 2 220nm

| Peak# | Ret. Time | Area | Height | Peak Start | Area% | Width at 50% Height |
|---|---|---|---|---|---|---|
| 1 | 46.097 | 37078585 | 653992 | 44.583 | 49.760 | 0.884 |
| 2 | 48.552 | 37435716 | 624233 | 47.383 | 50.240 | 0.934 |
| Total | | 74514301 | 1278225 | | 100.000 | |

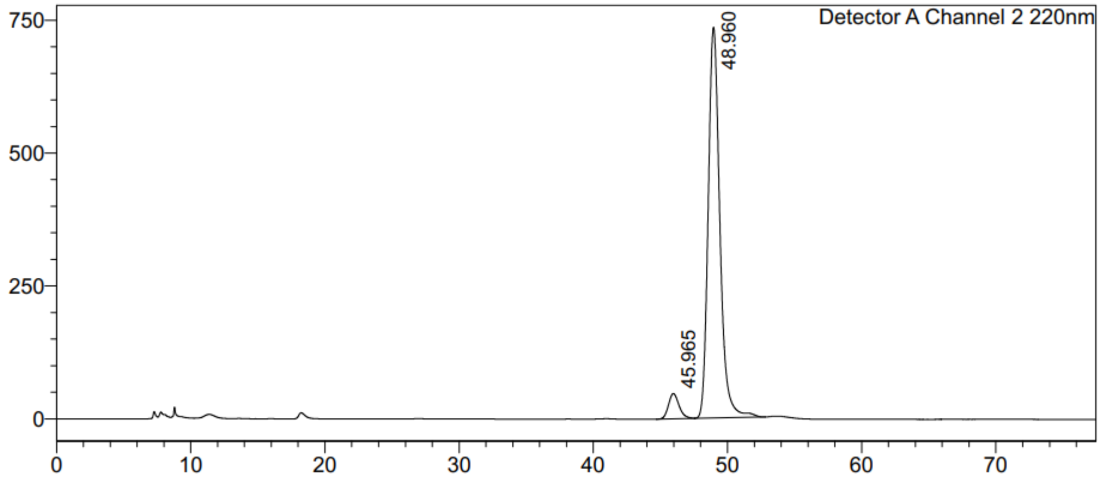


Detector A Channel 2 220nm

| Peak# | Ret. Time | Area | Height | Peak Start | Area% | Width at 50% Height |
|---|---|---|---|---|---|---|
| 1 | 45.965 | 2606171 | 47580 | 44.692 | 5.555 | 0.833 |
| 2 | 48.960 | 44307669 | 735100 | 47.558 | 94.445 | 0.894 |
| Total | | 46913840 | 782680 | | 100.000 | |

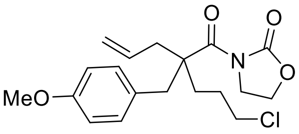


**16**
$C_{19}H_{24}ClNO_4$
M = 365.85 g/mol

**3-(2-(3-chloropropyl)-2-(4-methoxybenzyl)pent-4-enoyl)oxazolidin-2-one** (**16**): **$R_f$** = 0.35 (petroleum ether/EtOAc = 5/1). **$^1$H NMR** (400 MHz, $CDCl_3$): δ 7.04 (d, *J* = 8.4 Hz, 2H), 6.80 (d, *J* = 8.4 Hz, 2H), 5.85-5.74 (m, 1H), 5.17-5.13 (m, 2H), 4.40 (t, *J* = 8.0 Hz, 2H), 3.99 (t, *J* = 8.1 Hz, 2H), 3.78 (s, 3H), 3.50 (t, *J* = 6.6 Hz, 2H), 3.28 (d, *J* = 14.0 Hz, 1H), 3.14 (d, *J* = 14.4 Hz, 1H), 2.71-2.56 (m, 2H), 2.16-2.08 (m, 1H), 1.91-1.70 (m, 3H) ppm. **$^{13}$C NMR** (100 MHz, $CDCl_3$): δ 175.9, 158.3, 152.5, 133.9, 131.0, 129.3, 118.6, 113.7, 62.3, 55.2, 53.2, 45.5, 45.0, 37.2, 36.8, 30.2, 28.1 ppm. **HRMS** (ESI) *m/z*: $[M+Na]^+$ calcd. for $C_{19}H_{24}ClNO_4Na$: 388.1286; Found: 388.1293.

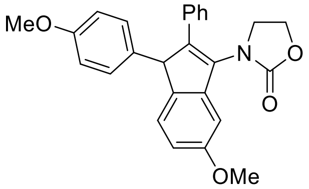


**15**
$C_{26}H_{23}NO_4$
M = 413.47 g/mol

**3-(5-methoxy-1-(4-methoxyphenyl)-2-phenyl-1H-inden-3-yl)oxazolidin-2-one** (**15**): **R*f*** = 0.40 (petroleum ether/EtOAc = 5/1). **$^1$H NMR** (400 MHz, $CDCl_3$): δ 7.34-7.25 (m, 5H), 7.18 (t, *J* = 7.2 Hz, 1H), 7.02 (d, *J* = 8.8 Hz, 2H), 6.85 (dd, *J* = 8.4, 2.2 Hz, 1H), 6.77 (s, 1H), 6.71 (d, *J* = 8.4 Hz, 2H), 4.89 (s, 1H), 4.50-4.39 (m, 2H), 3.76-3.62 (m, 8H) ppm. **$^{13}$C NMR** (100 MHz, $CDCl_3$): δ 159.1, 158.4, 156.7, 148.7, 141.6, 134.0, 133.5, 132.5, 130.4, 129.2, 128.5, 128.1, 127.6, 120.2, 114.1, 112.6, 110.6, 63.0, 55.5, 55.3, 55.0, 45.4 ppm. **HRMS** (ESI) *m/z*: $[M+Na]^+$ calcd. for $C_{26}H_{23}NO_4Na$: 414.1705; Found: 414.1705.

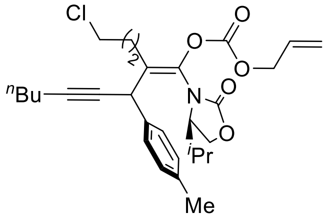


(*E*)-**6vh**
$C_{29}H_{38}ClNO_5$
M = 516.08 g/mol

**(*R*,*E*)-allyl (2-(3-chloropropyl)-1-(4-isopropyl-2-oxooxazolidin-3-yl)-3-(*p*-tolyl)non-1-en-4-yn-1-yl) carbonate** ((*E*)-**6vh**): Prepared from (*R*)-3-(5-chloropent-1-yn-1-yl)-4-isopropyloxazolidin-2-one (**1v**, 55.1 mg, 0.24 mmol) and allyl (1-(*p*-tolyl)hept-2-yn-1-yl) carbonate (**5h**, 57.3 mg, 0.2 mmol) according to the GP2 with 5 mol% $B(C_6F_5)_3$ at 25 ℃ for 12 h. Purification by flash chromatography on silica gel using petroleum ether/EtOAc (10/1 to 5/1) afforded (*E*)-**6vh** as a colorless oil (50.6 mg, 49% yield, *dr* = 67:33, the diastereoisomers cannot be separated by chromatography).

(*E*)-**6vh**: **R*f*** = 0.35 (petroleum ether/EtOAc = 5/1). The major isomer**:** **$^1$H NMR** (400 MHz, $CDCl_3$): δ 7.41-7.37 (m, 2H), 7.12 (d, *J* = 8.0 Hz, 2H), 5.98-5.84 (m, 1H), 5.37 (d, *J* = 17.2 Hz, 1H), 5.29 (d, *J* = 11.2 Hz, 1H), 4.94 (s, 1H), 4.64 (d, *J* = 6.0 Hz, 1H), 4.38-4.28 (m, 1H), 4.16-4.09 (m, 1H), 3.90-3.78 (m, 1H), 3.50-3.32 (m, 2H), 2.55-2.42 (m, 1H), 2.32 (s, 3H), 2.27 (td, *J* = 7.2, 2.0 Hz, 2H), 2.16-1.96 (m, 3H), 1.72-1.65 (m, 1H), 1.58-1.41 (m, 4H), 0.99-0.91 (m, 10H) ppm. The minor isomer**:** **$^1$H NMR** (400 MHz, $CDCl_3$): δ 7.41-7.37 (m, 2H), 7.12 (d, *J* = 8.0 Hz, 2H), 5.98-5.84 (m, 1H), 5.37 (d, *J* = 17.2 Hz, 1H), 5.29 (d, *J* = 11.2 Hz, 1H), 4.89 (s, 1H), 4.60 (d, *J* = 6.0 Hz, 1H),

4.38-4.28 (m, 1H), 4.16-4.09 (m, 1H), 3.90-3.78 (m, 1H), 3.50-3.32 (m, 2H), 2.55-2.42 (m, 1H), 2.32 (s, 3H), 2.27 (td, *J* = 7.2, 2.0 Hz, 2H), 2.16-1.96 (m, 3H), 1.72-1.65 (m, 1H), 1.58-1.41 (m, 4H), 0.99-0.91 (m, 10H) ppm.

**$^{13}$C NMR** (100 MHz, $CDCl_3$): δ 155.53, 155.49, 151.8, 151.7, 136.41, 136.39, 135.1, 133.81, 133.77, 130.74, 130.68, 129.1, 129.0, 128.9, 127.5, 127.4, 119.9, 119.8, 85.33, 85.26, 77.4, 77.2, 69.49, 69.46, 64.5, 64.1, 61.7, 59.6, 45.4, 43.6, 36.7, 34.7, 31.4, 30.9, 30.5, 30.1, 29.9, 29.0, 28.3, 26.8, 22.0, 21.6, 21.0, 18.5, 18.4, 18.2, 17.2, 16.1, 15.9, 15.0, 14.6, 13.6 ppm. **HRMS** (ESI) *m/z*: $[M+Na]^+$ calcd. for $C_{29}H_{38}ClNO_5Na$: 538.2337; Found: 538.2334.

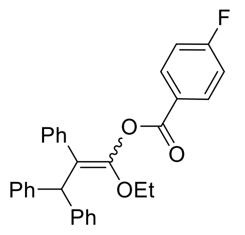


**16**
$C_{30}H_{25}FNO_3$
M = 452.53 g/mol

**1-ethoxy-2,3,3-triphenylprop-1-en-1-yl 4-fluorobenzoate** (**16**): Prepared from (ethoxyethynyl)benzene (**1ad**, 35.1 mg, 0.24 mmol) and benzhydryl 4-fluorobenzoate (**2a**, 61.3 mg, 0.2 mmol) according to the GP1 with 5 mol% $B(C_6F_5)_3$ at 80 ℃ for 12 h. Purification by flash chromatography on silica gel using petroleum ether/EtOAc (100/1 to 50/1) afforded the desired product **16.** The major stereoisomer was isolated as a colorless oil (19.4 mg, 21% yield).

For major stereoisomer: $\mathbf{R_f}$ = 0.35 (petroleum ether/EtOAc = 50/1). **$^1$H NMR** (400 MHz, $CDCl_3$): δ 7.82 (dd, *J* = 8.8, 5.2 Hz, 2H), 7.22-7.13 (m, 13H), 7.13-7.08 (m, 2H), 7.04 (t, *J* = 8.6 Hz, 2H), 5.26 (s, 1H), 3.78 (q, *J* = 7.2 Hz, 2H), 1.04 (t, *J* = 7.2 Hz, 3H) ppm. **$^{13}$C NMR** (100 MHz, $CDCl_3$): δ 166.0 (d, *J* = 253.5 Hz), 162.5, 150.2, 141.7, 137.8, 132.7 (d, *J* = 9.5 Hz), 129.8, 129.3, 128.0, 127.8, 126.7, 126.1, 124.7 (d, *J* = 2.9 Hz), 115.5 (d, *J* = 21.9 Hz), 113.4, 67.8, 53.6, 14.8. ppm. **$^{19}$F NMR** (376 MHz, $CDCl_3$): δ -104.28 ppm. **HRMS** (ESI) *m/z*: $[M+Na]^+$ calcd. for $C_{30}H_{25}FNO_3Na$: 475.1686; Found: 475.1683.

## 14 Crystal Structure of (*Z*)-3aa and (*E*)-6oa

Note: The thermal ellipsoids are 50% probability level. The crystals are grown by slow solvent (DCM/*n*-Hexane) evaporation at room temperature.

CCDC number of (*Z*)-**3aa** is 2236736.

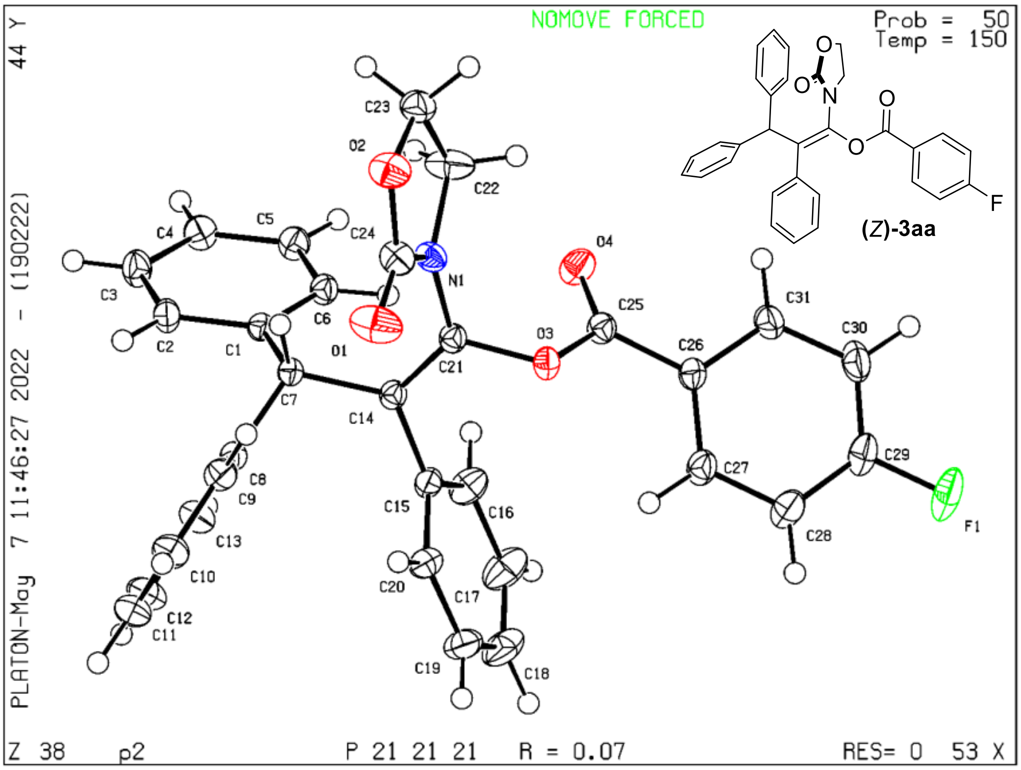


**Datablock: p2**

Bond precision: C-C = 0.0033 A Wavelength=0.71073

| | | | |
|---|---|---|---|
| Cell: | a=9.7830(18) | b=10.941(2) | c=22.744(4) |
| | alpha=90 | beta=90 | gamma=90 |
| Temperature: | 150 K | | |

| | Calculated | Reported |
|---|---|---|
| Volume | 2434.4(8) | 2434.4(8) |
| Space group | P 21 21 21 | P 21 21 21 |
| Hall group | P 2ac 2ab | P 2ac 2ab |
| Moiety formula | C31 H24 F N O4 | C31 H24 F N O4 |
| Sum formula | C31 H24 F N O4 | C31 H24 F N O4 |
| Mr | 493.51 | 493.51 |
| Dx,g cm-3 | 1.347 | 1.347 |
| Z | 4 | 4 |
| Mu (mm-1) | 0.094 | 0.094 |
| F000 | 1032.0 | 1032.0 |
| F000' | 1032.53 | |
| h,k,lmax | 15,17,36 | 15,17,36 |
| Nref | 10691[ 5917] | 10667 |
| Tmin,Tmax | 0.979,0.981 | |
| Tmin' | 0.979 | |

Correction method= Not given

Data completeness= 1.80/1.00 Theta(max)= 34.960

R(reflections)= 0.0714( 8426) wR2(reflections)= 0.1980( 10667)

S = 1.082 Npar= 335

CCDC number of (*E*)-**6oa** is 2236734.

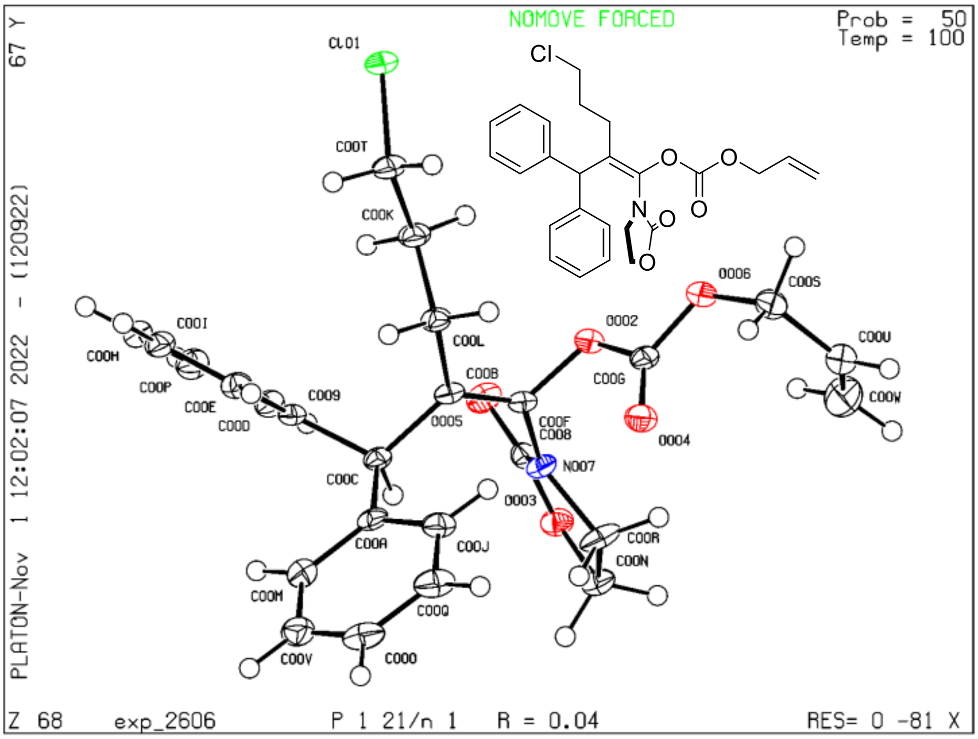


## Datablock: exp_2606

Bond precision: C-C = 0.0022 A Wavelength=1.54184

| | | | |
|---|---|---|---|
| Cell: | a=9.5099(2) | b=18.1304(3) | c=14.0225(2) |
| | alpha=90 | beta=105.611(2) | gamma=90 |
| Temperature: | 100 K | | |

| | Calculated | Reported |
|---|---|---|
| Volume | 2328.55(7) | 2328.55(7) |
| Space group | P 21/n | P 1 21/n 1 |
| Hall group | -P 2yn | -P 2yn |
| Moiety formula | C25 H26 Cl N O5 | C25 H26 Cl N O5 |
| Sum formula | C25 H26 Cl N O5 | C25 H26 Cl N O5 |
| Mr | 455.92 | 455.92 |
| Dx,g cm-3 | 1.301 | 1.300 |
| Z | 4 | 4 |
| Mu (mm-1) | 1.752 | 1.752 |
| F000 | 960.0 | 960.0 |
| F000' | 964.27 | |
| h,k,lmax | 11,21,16 | 11,21,16 |
| Nref | 4120 | 4118 |
| Tmin,Tmax | 0.521,0.496 | 0.345,1.000 |
| Tmin' | 0.473 | |

Correction method= # Reported T Limits: Tmin=0.345 Tmax=1.000
AbsCorr = MULTI-SCAN

Data completeness= 1.000 Theta(max)= 66.594

R(reflections)= 0.0363( 3851) wR2(reflections)= 0.0918( 4118)

S = 1.057 Npar= 297

# 15 NMR Spectra

## $^{1}$H and $^{13}$C NMR Spectra for Compound 2u:

### $^{1}$H NMR (400 MHz, $CDCl_3$)

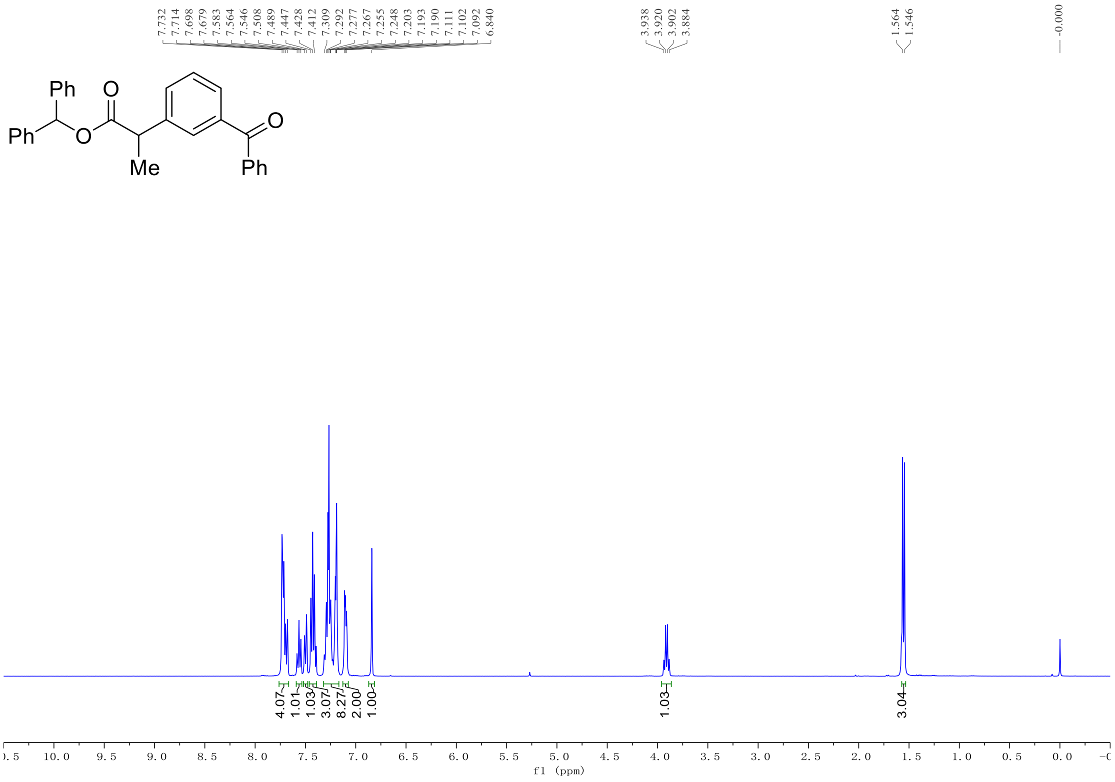


### $^{13}$C NMR (100 MHz, $CDCl_3$)

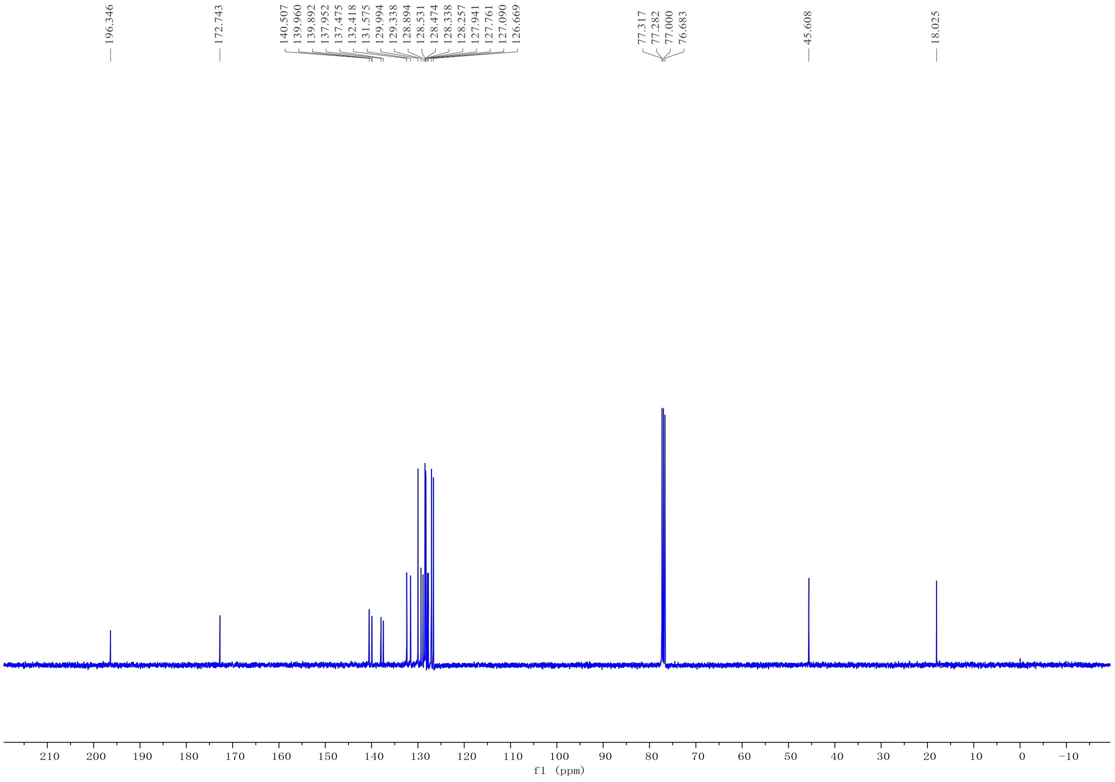

**$^{1}$H and $^{13}$C NMR Spectra for Compound 2v**:

$^{1}$H NMR (400 MHz, $CDCl_3$)

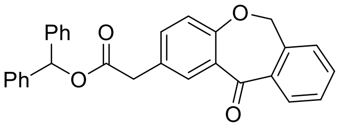


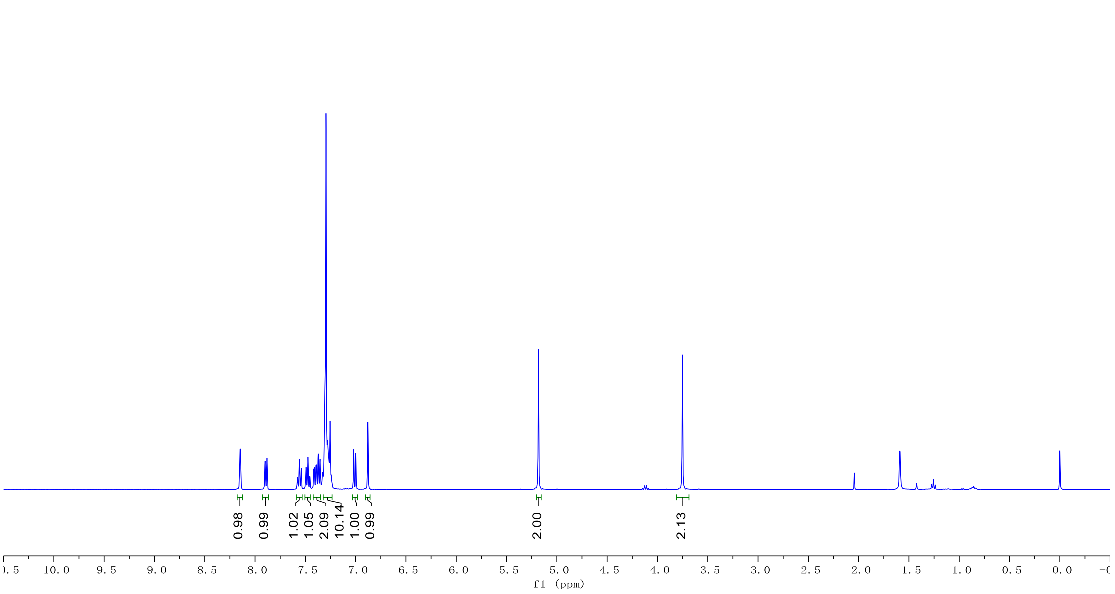


$^{13}$C NMR (100 MHz, $CDCl_3$)

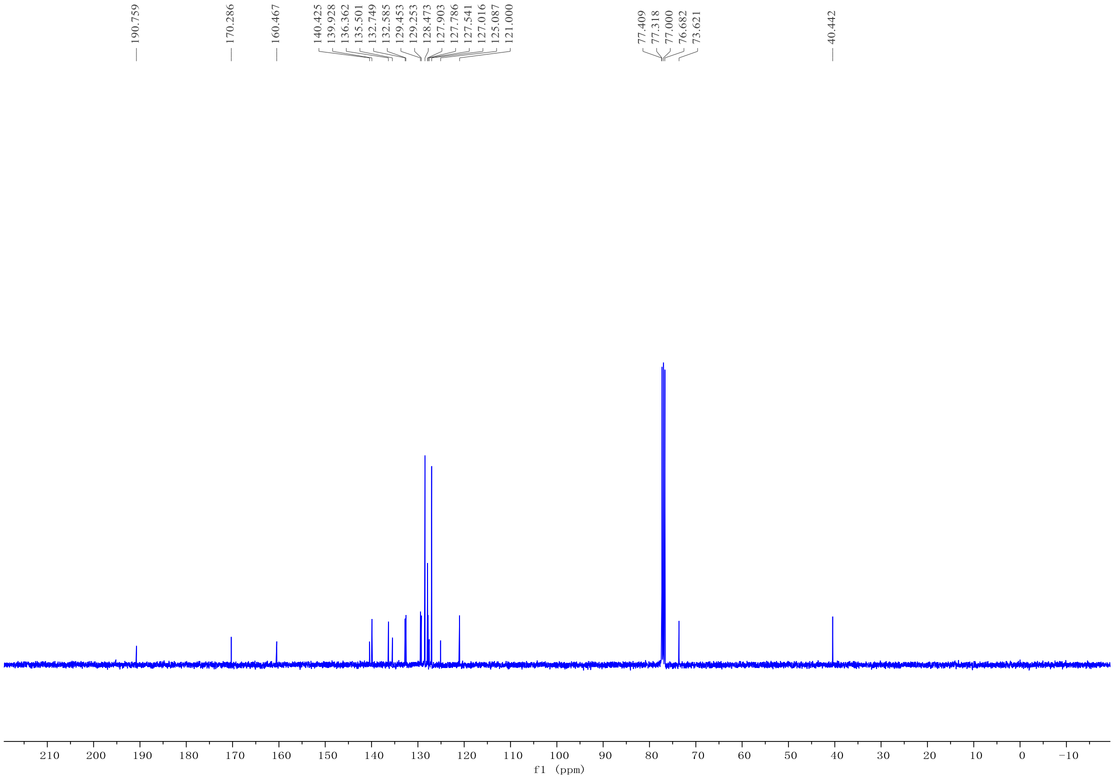

## $^{1}$H and $^{13}$C NMR Spectra for Compound 2w:

$^{1}$H NMR (400 MHz, $CDCl_3$)

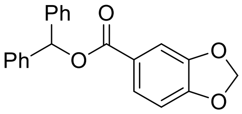


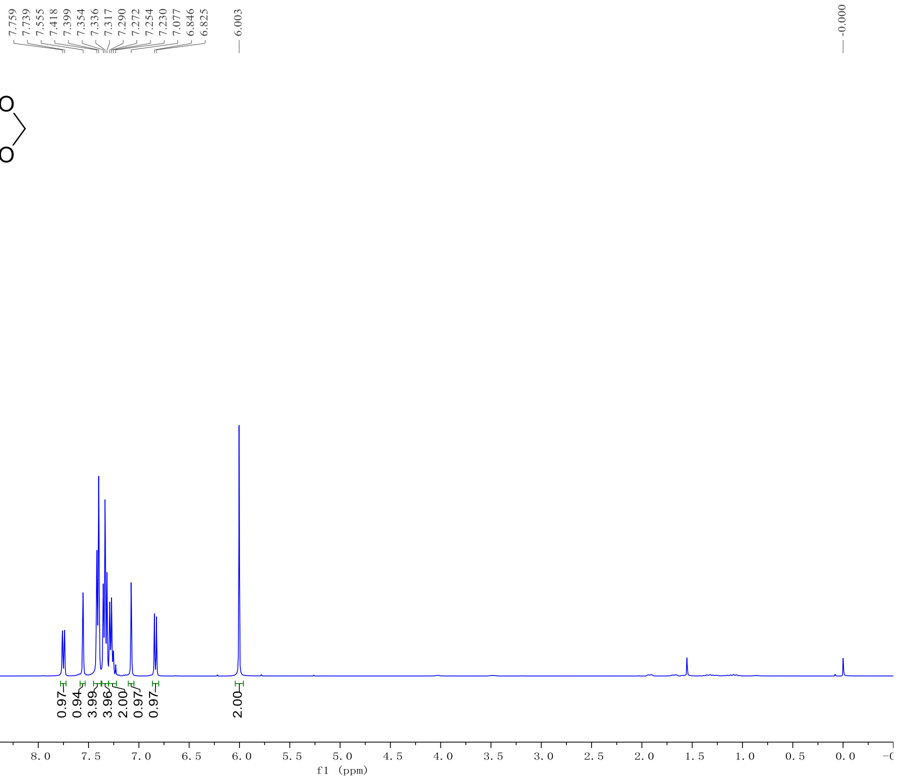


$^{13}$C NMR (100 MHz, $CDCl_3$)

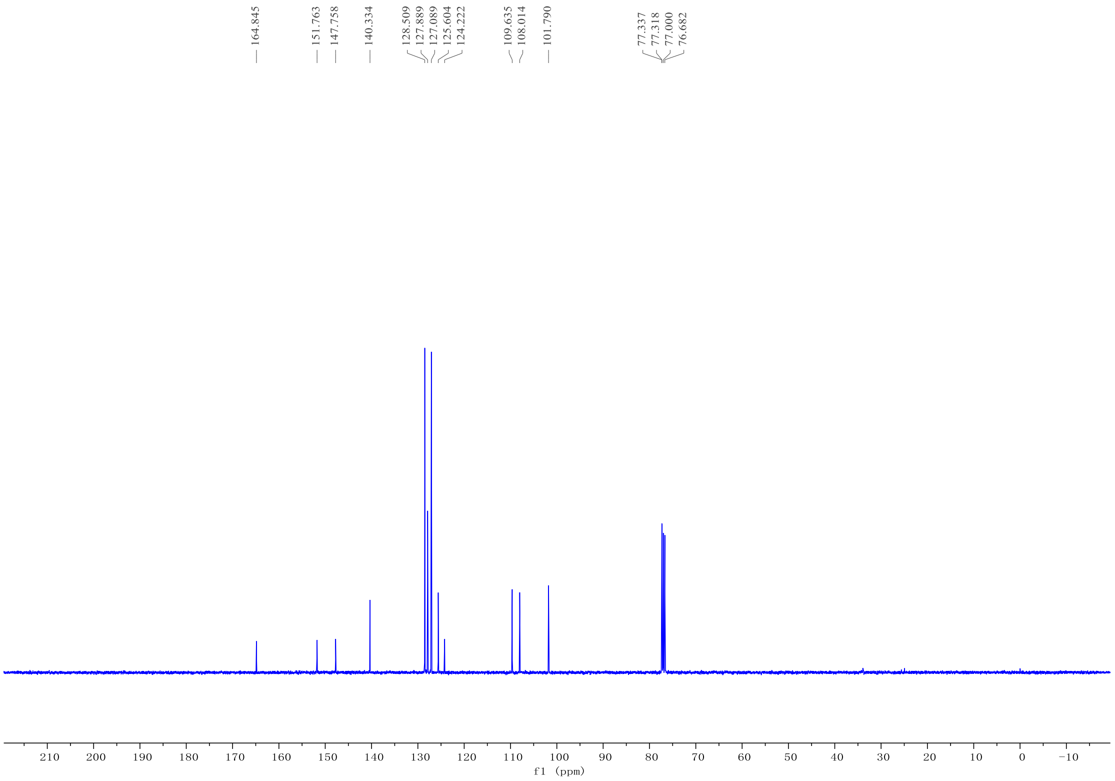

## $^{1}$H and $^{13}$C NMR Spectra for Compound 2x:

$^{1}$H NMR (400 MHz, $CDCl_3$)

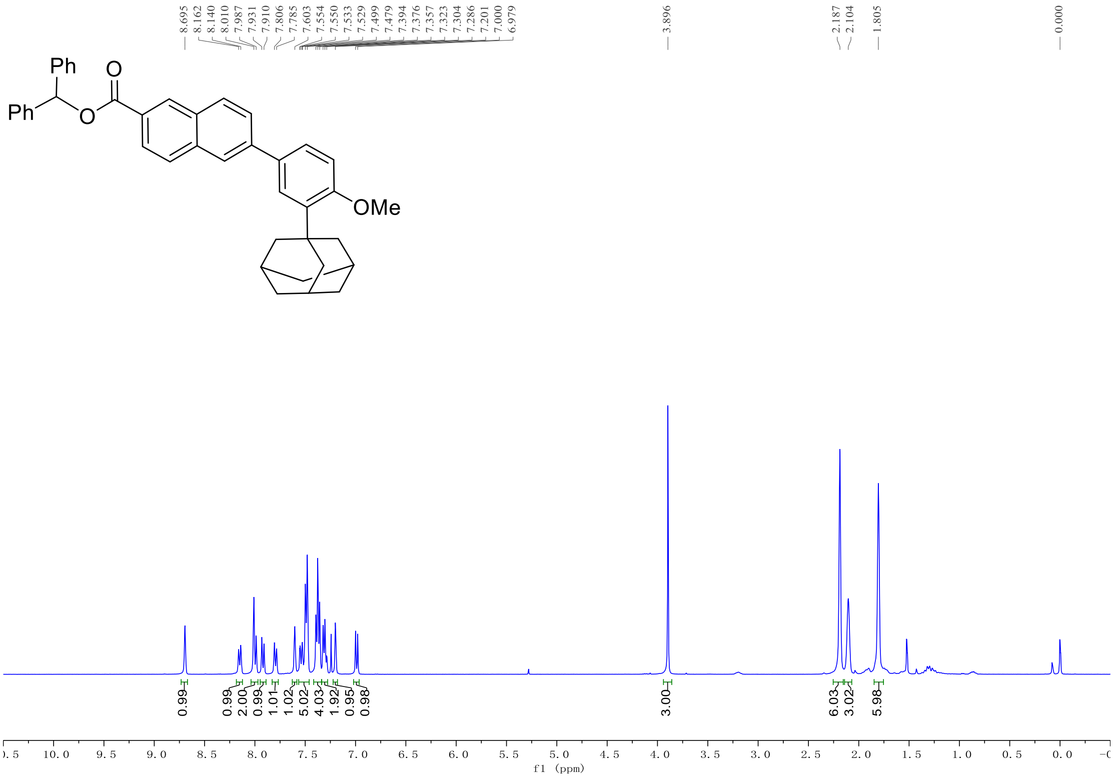


$^{13}$C NMR (100 MHz, $CDCl_3$)

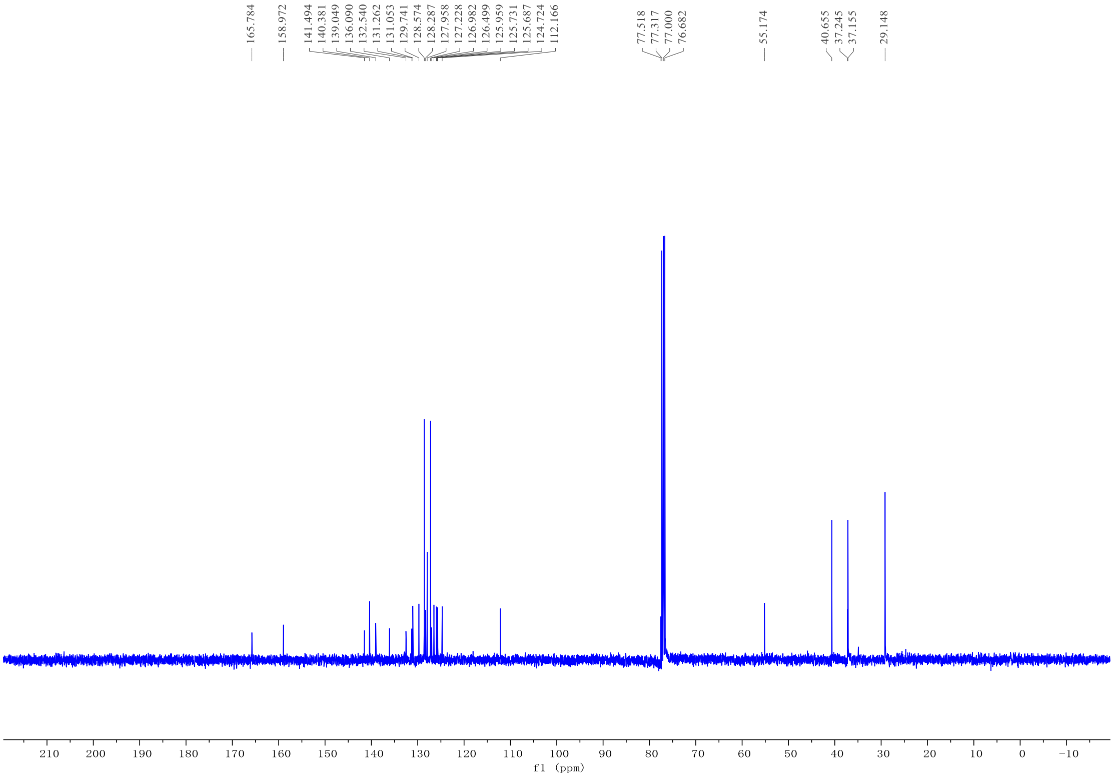

**$^{1}$H and $^{13}$C NMR Spectra for Compound 2y**:

$^{1}$H NMR (400 MHz, $CDCl_3$)

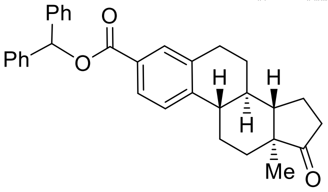

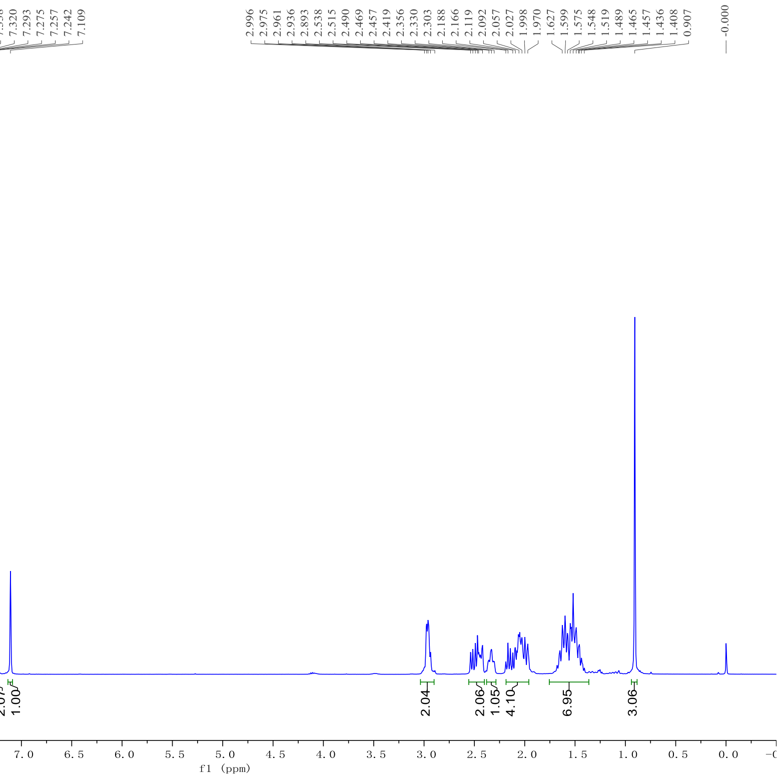

$^{13}$C NMR (100 MHz, $CDCl_3$)

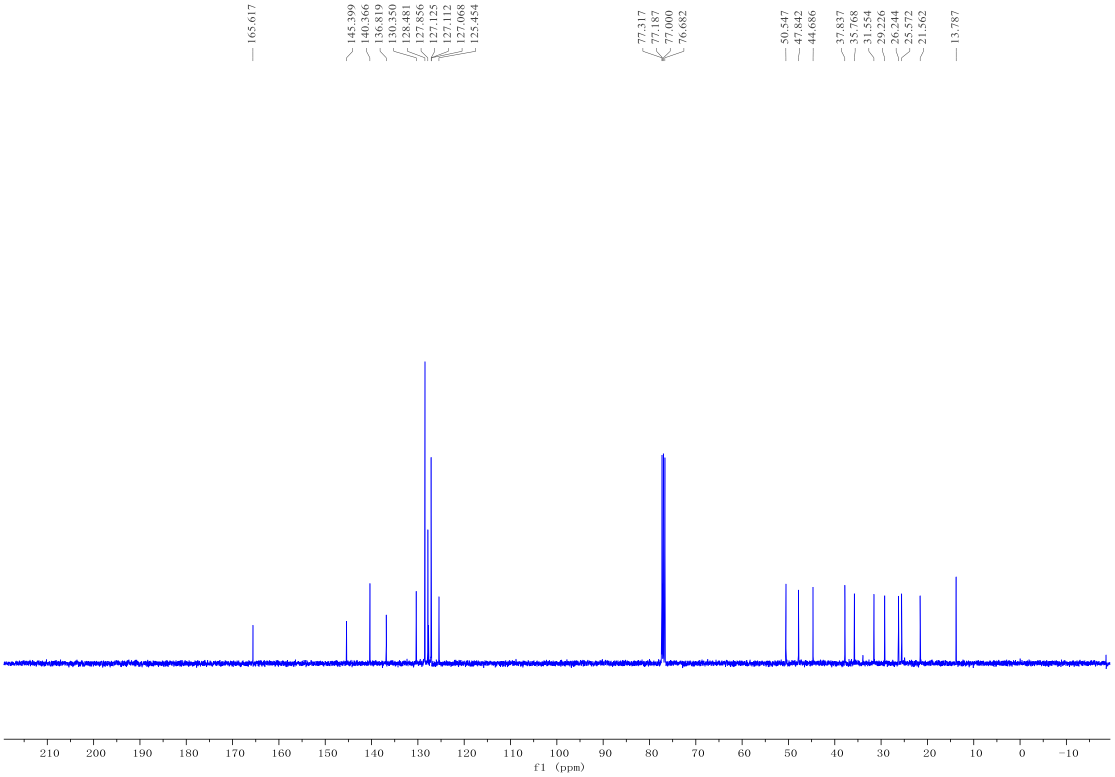

**$^{1}$H and $^{13}$C NMR Spectra for Compound 2z**:

$^{1}$H NMR (400 MHz, $CDCl_3$)

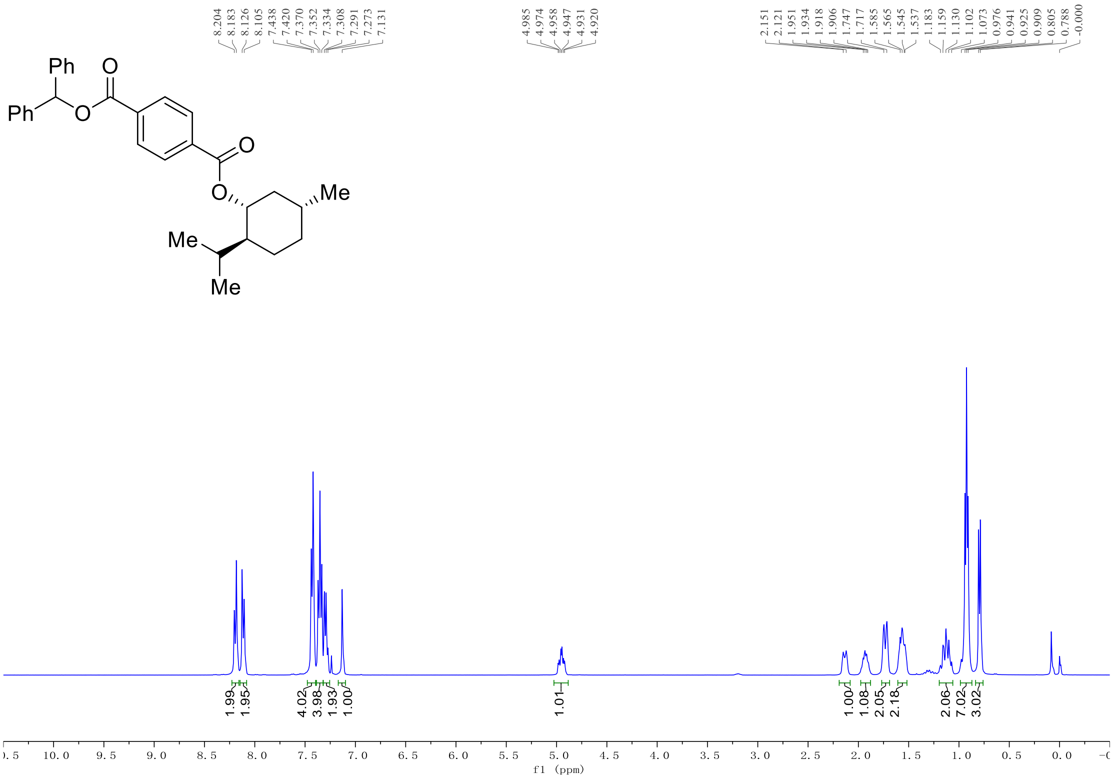

$^{13}$C NMR (100 MHz, $CDCl_3$)

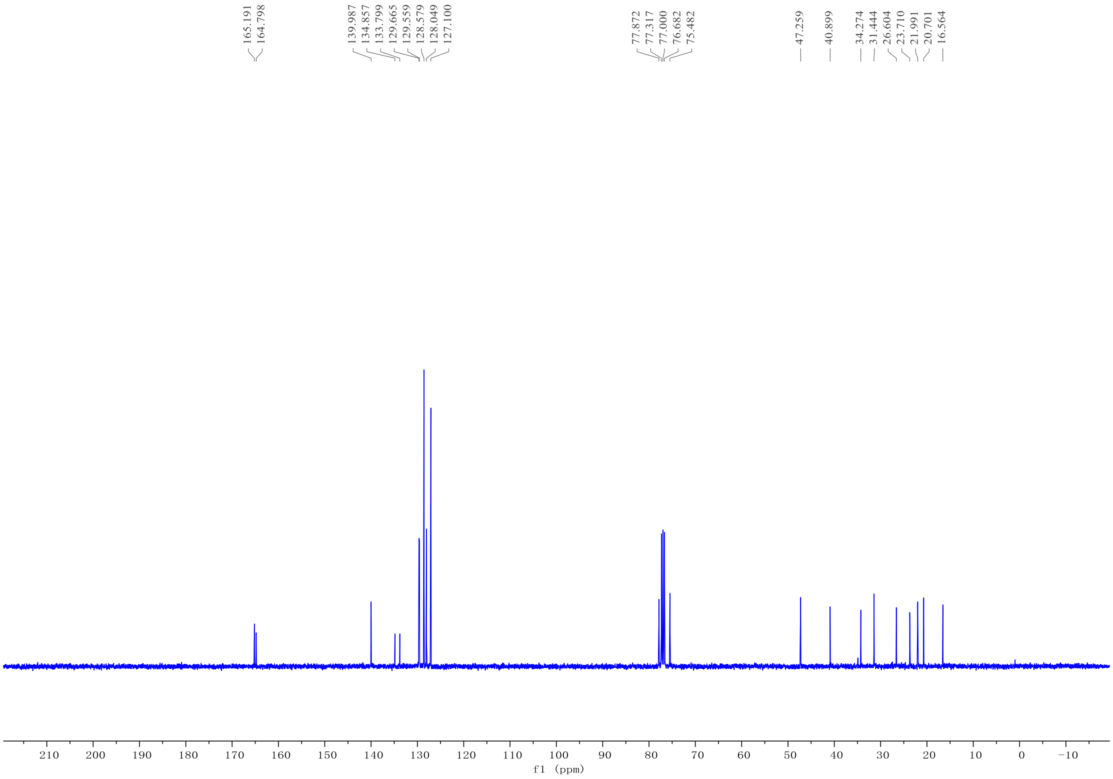

**$^{1}$H and $^{13}$C NMR Spectra for Compound 2aa**:

$^{1}$H NMR (400 MHz, $CDCl_3$)

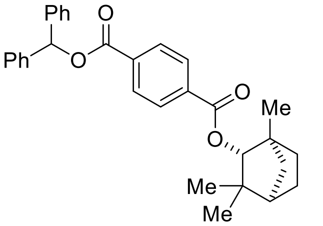


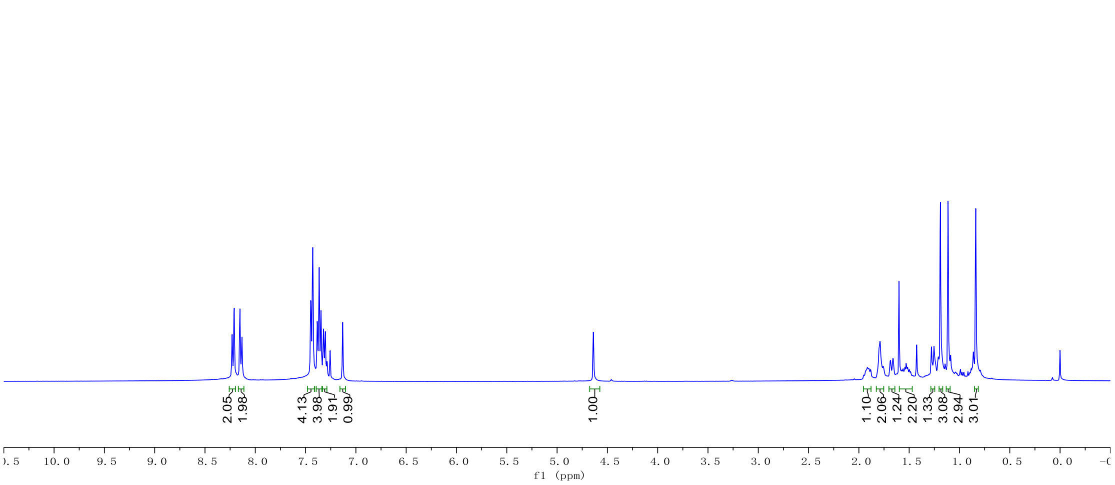


$^{13}$C NMR (100 MHz, $CDCl_3$)

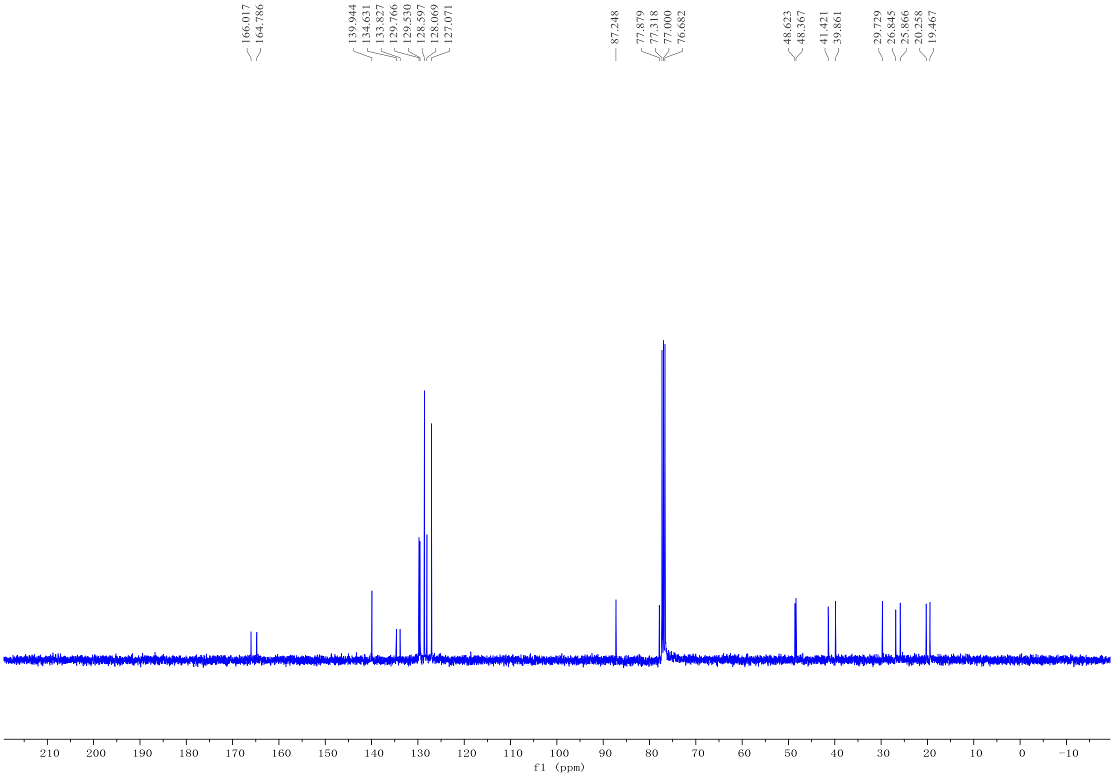

## $^{1}$H and $^{13}$C NMR Spectra for Compound 2ab:

$^{1}$H NMR (400 MHz, $CDCl_3$)

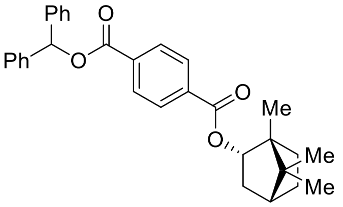


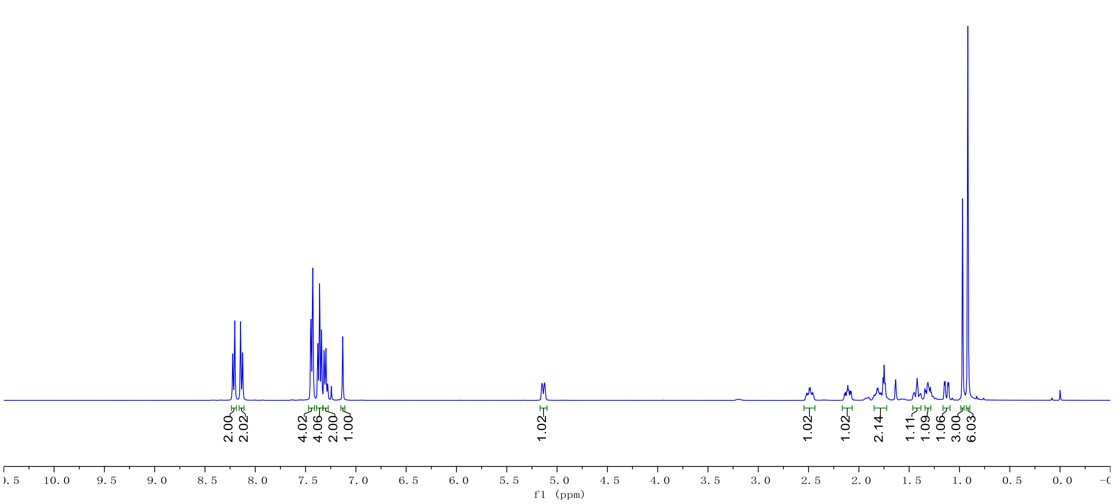


$^{13}$C NMR (100 MHz, $CDCl_3$)

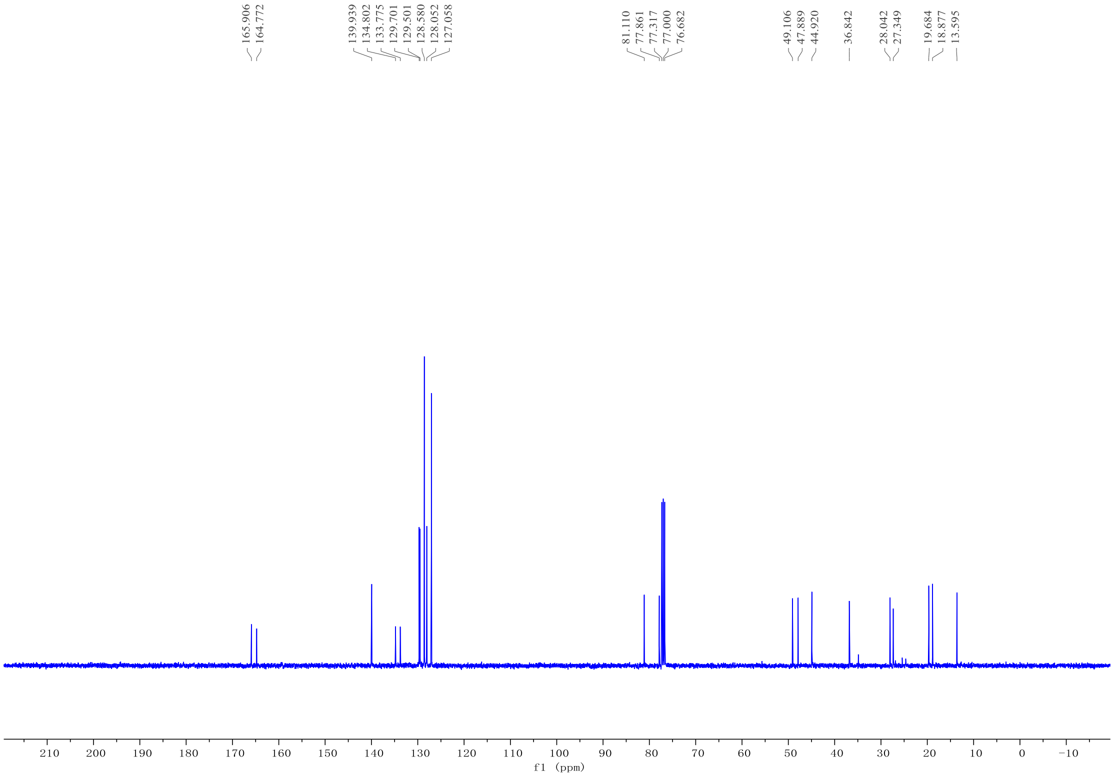

## $^{1}$H and $^{13}$C NMR Spectra for Compound (*Z*)-3aa:

$^{1}$H NMR (400 MHz, $CDCl_3$)

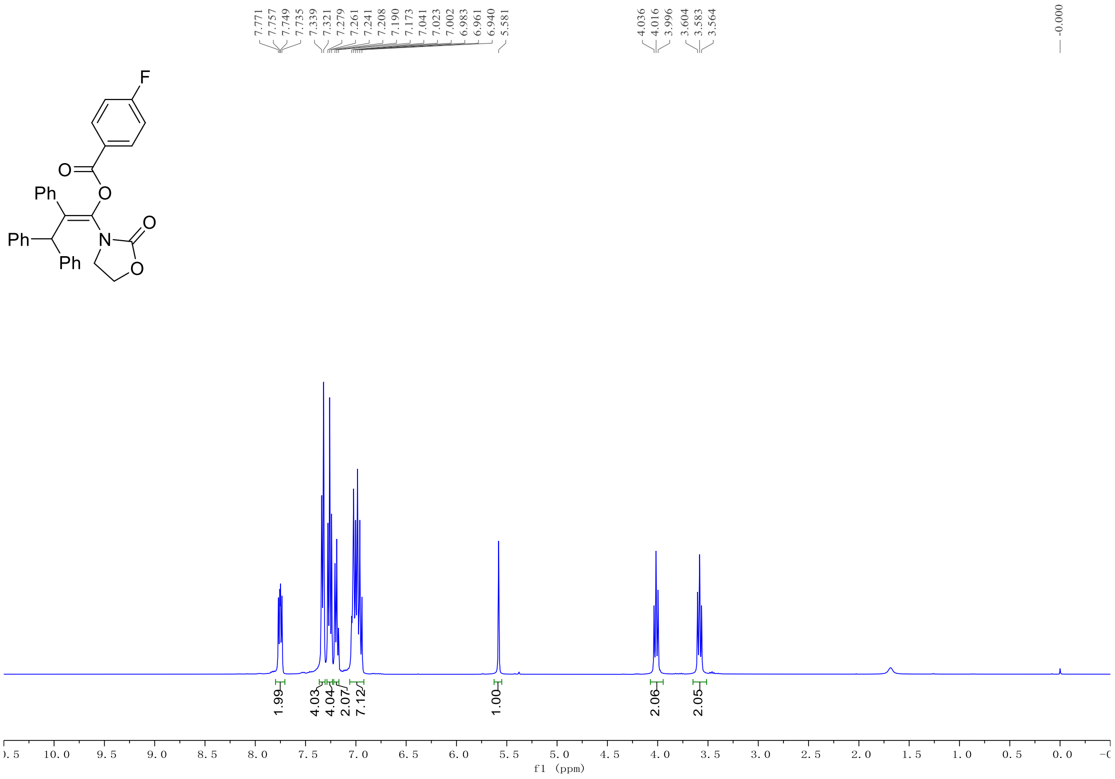


$^{13}$C NMR (100 MHz, $CDCl_3$)

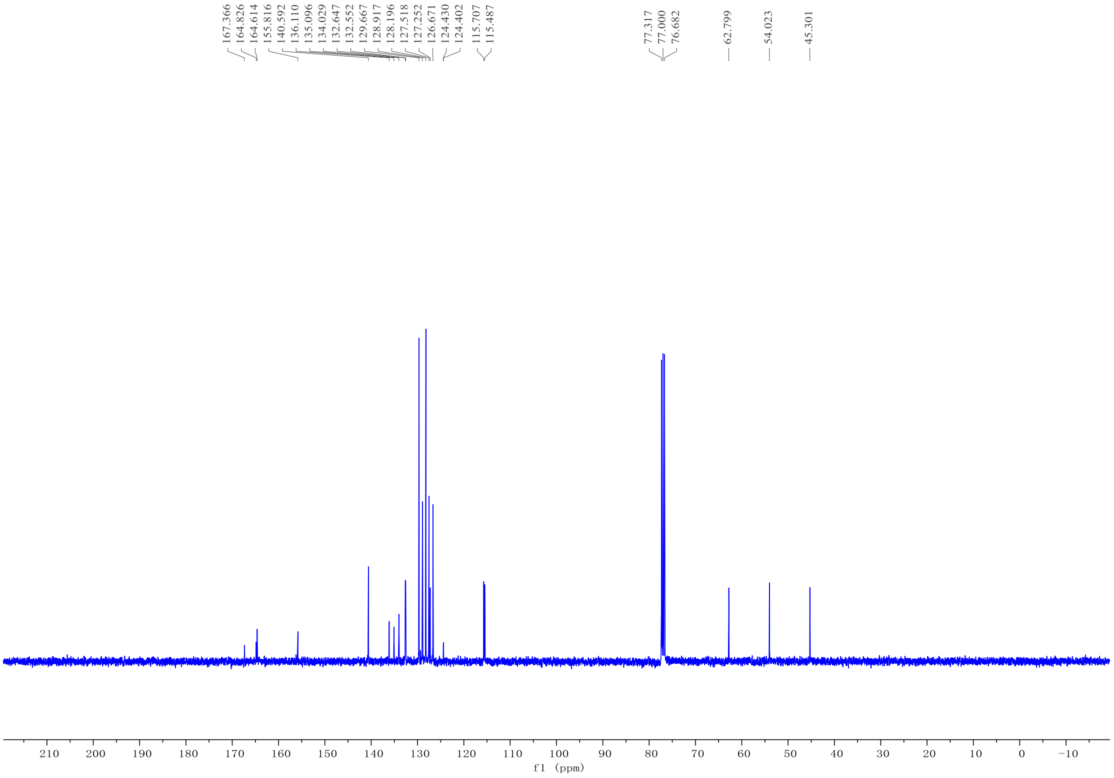

$^{19}$F NMR (400 MHz, $CDCl_3$)

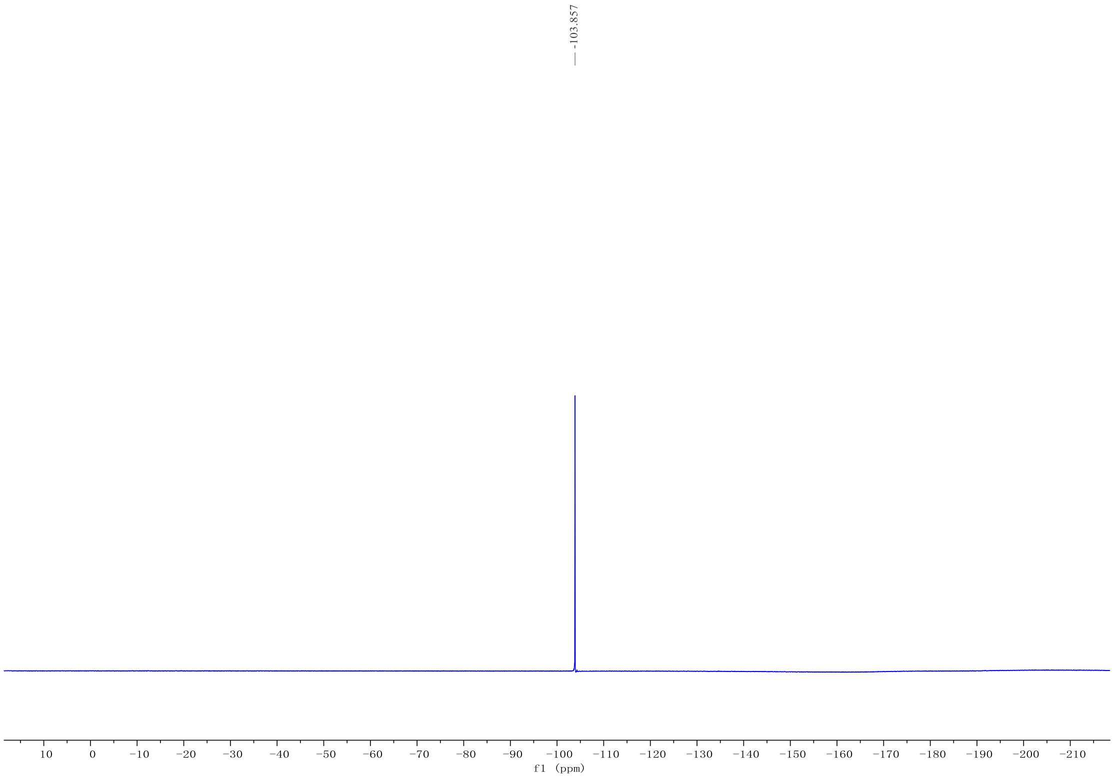


## $^{1}$H and $^{13}$C NMR Spectra for Compound (*Z*)-3ab:

$^{1}$H NMR (400 MHz, $CDCl_3$)

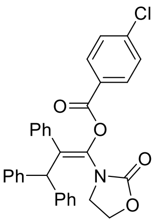


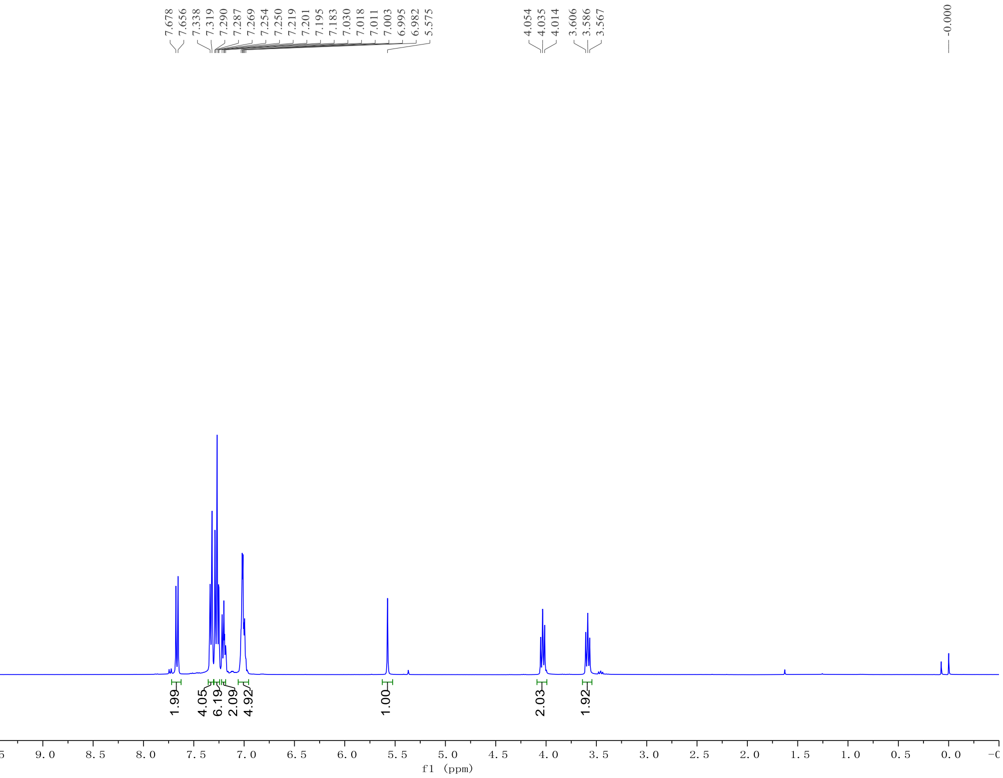

$^{13}$C NMR (100 MHz, $CDCl_3$)

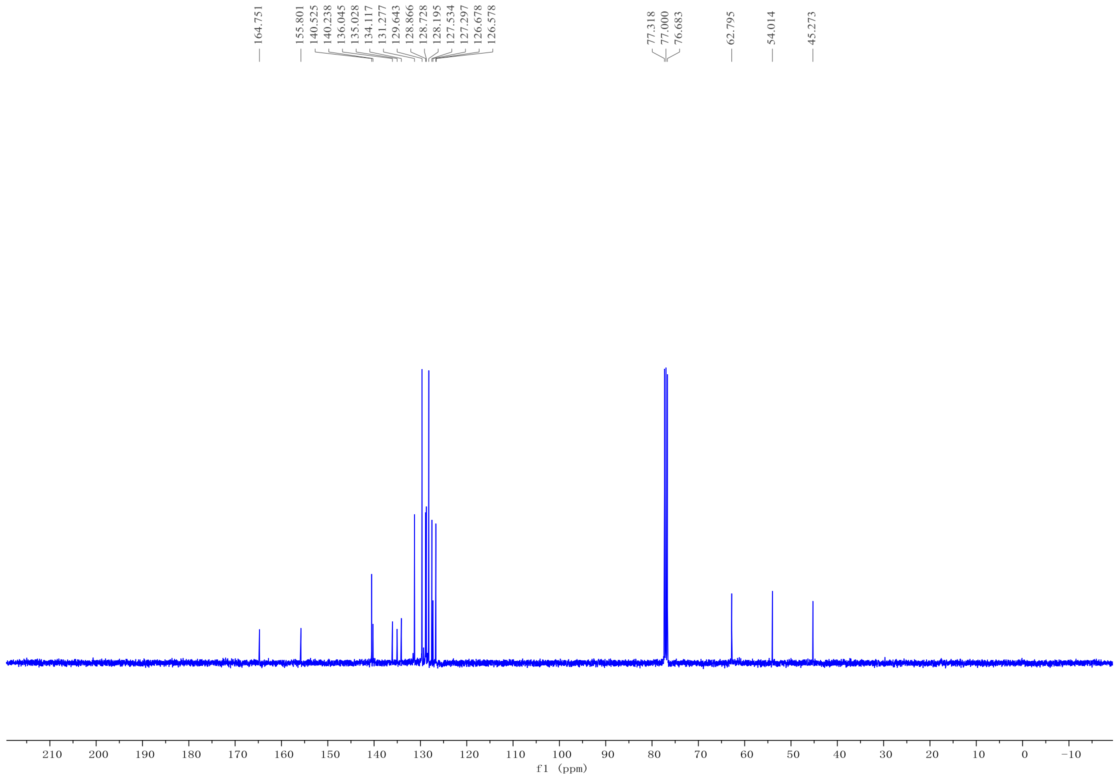


## $^{1}$H and $^{13}$C NMR Spectra for Compound (*Z*)-3ac:

$^{1}$H NMR (400 MHz, $CDCl_3$)

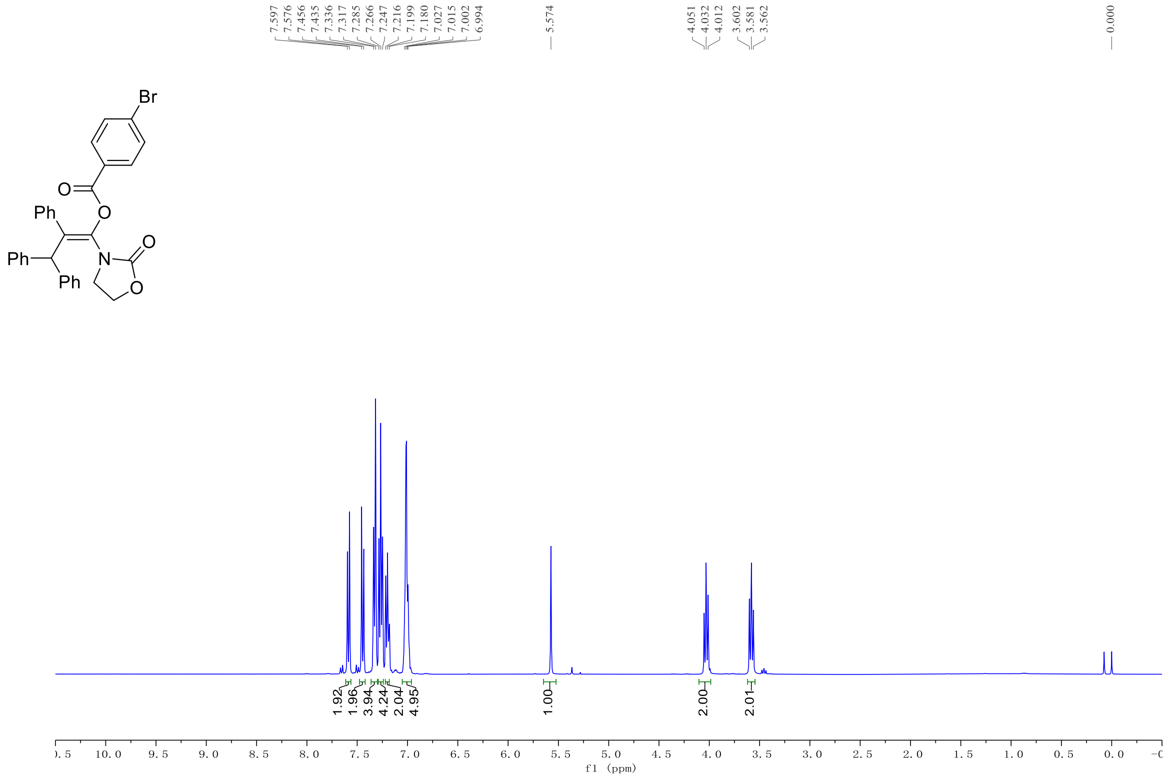

$^{13}$C NMR (100 MHz, CDCl$_3$)

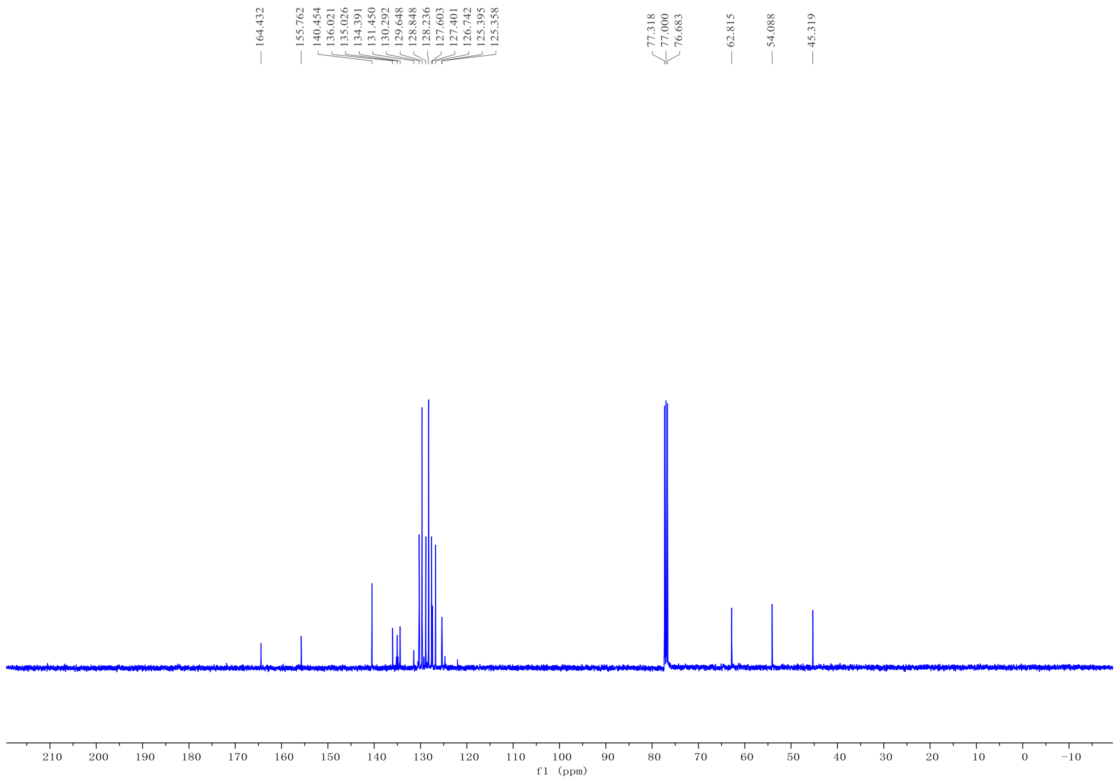


**$^{1}$H and $^{13}$C NMR Spectra for Compound (*Z*)-3ad**:

$^{1}$H NMR (400 MHz, CDCl$_3$)

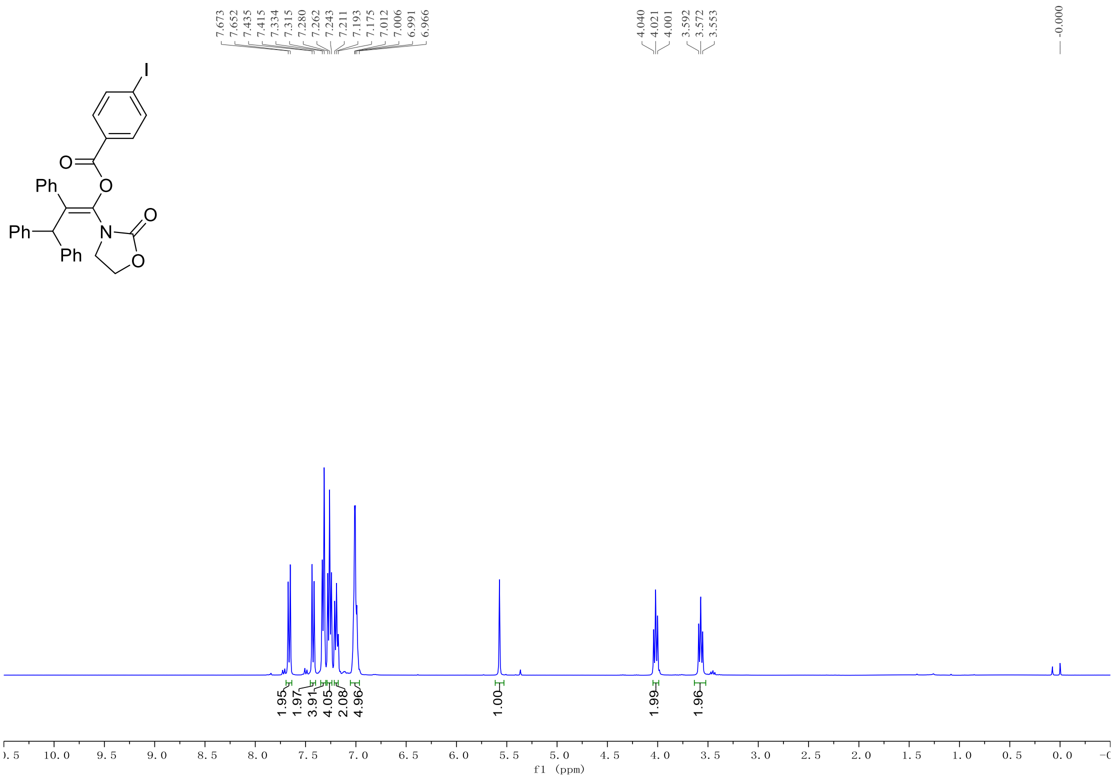

$^{13}C$ NMR (100 MHz, $CDCl_3$)

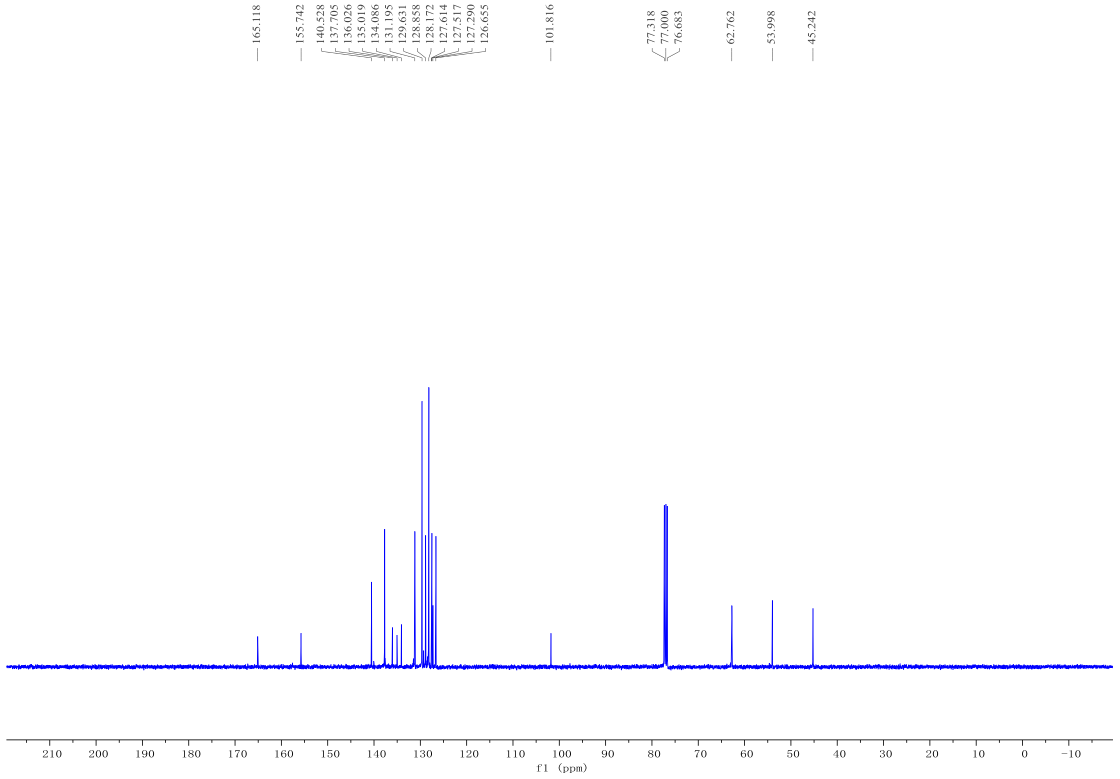


## $^1H$ and $^{13}C$ NMR Spectra for Compound (*Z*)-3ae:

$^1H$ NMR (400 MHz, $CDCl_3$)

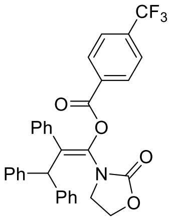


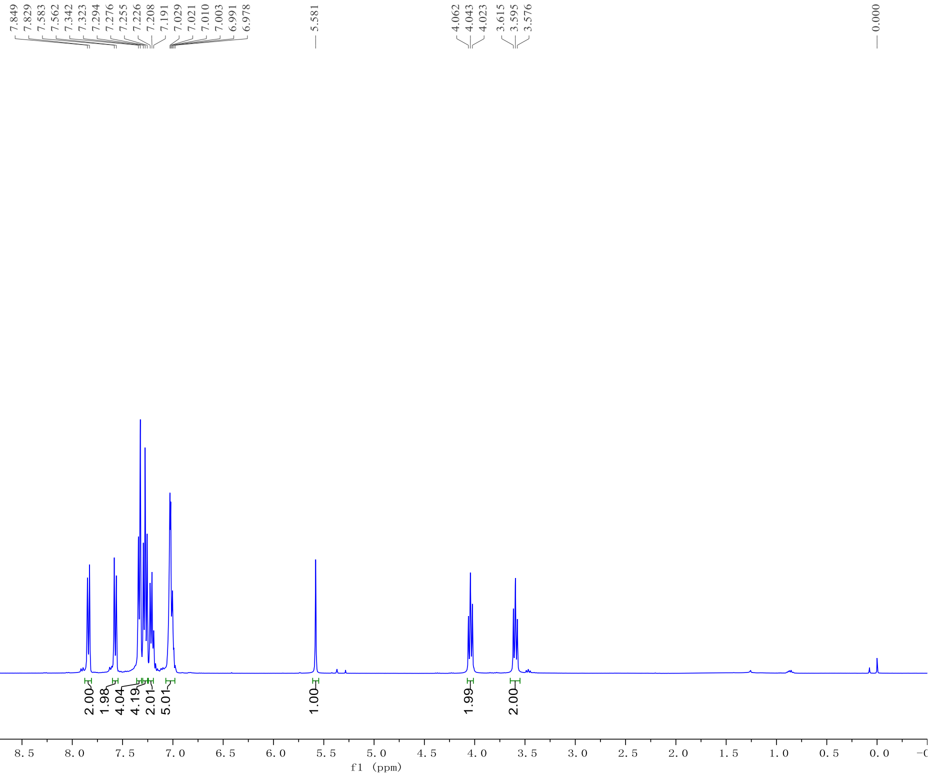

$^{13}$C NMR (100 MHz, $CDCl_3$)

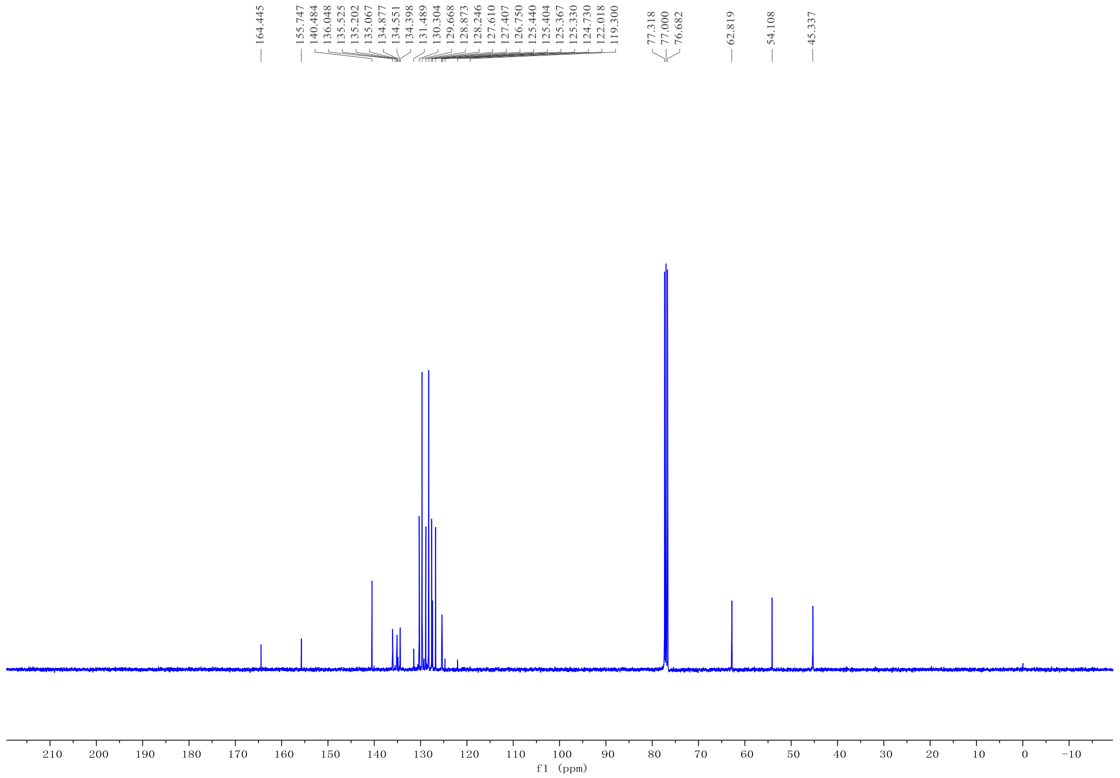


$^{19}$F NMR (376 MHz, $CDCl_3$)

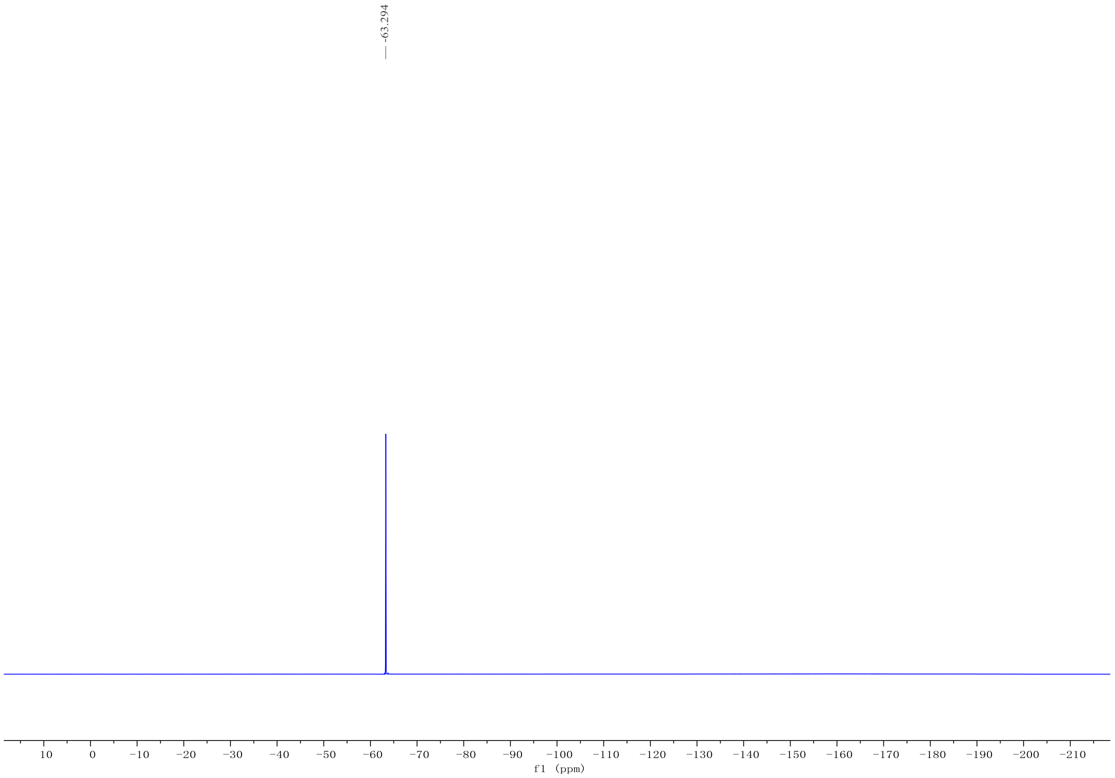

## $^1$H and $^{13}$C NMR Spectra for Compound (*Z*)-3af:

$^1$H NMR (400 MHz, $CDCl_3$)

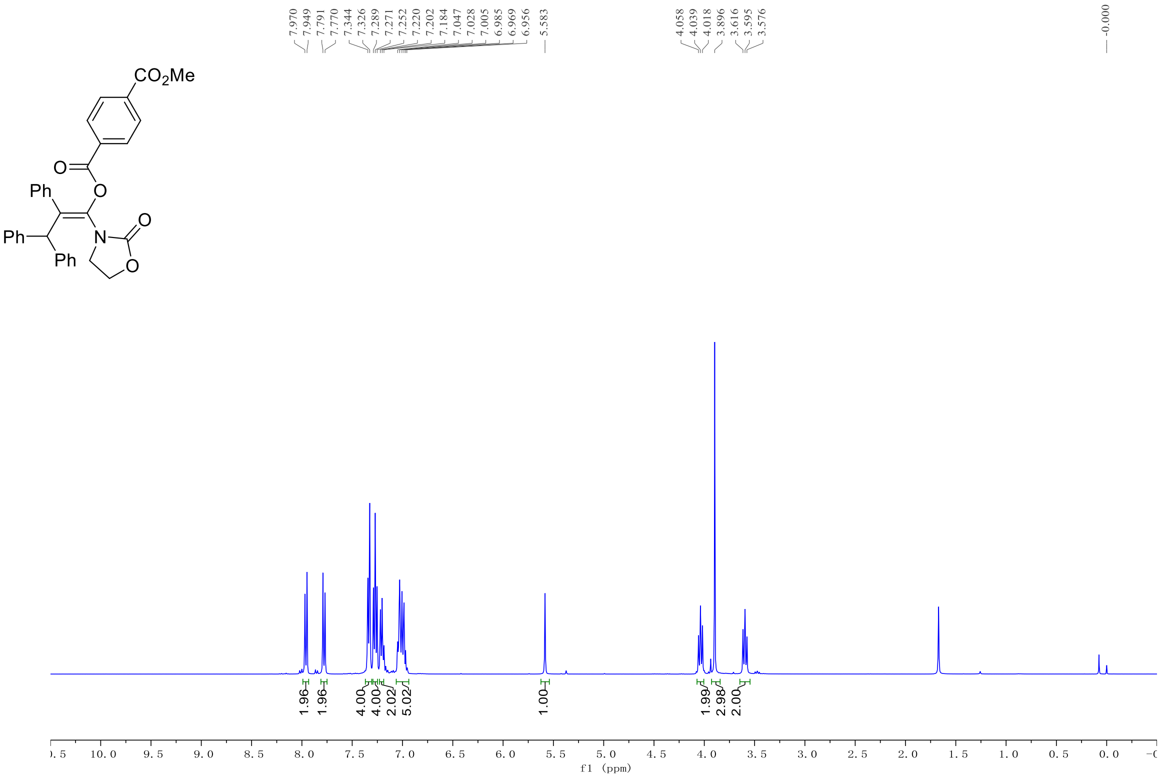


$^{13}$C NMR (100 MHz, $CDCl_3$)

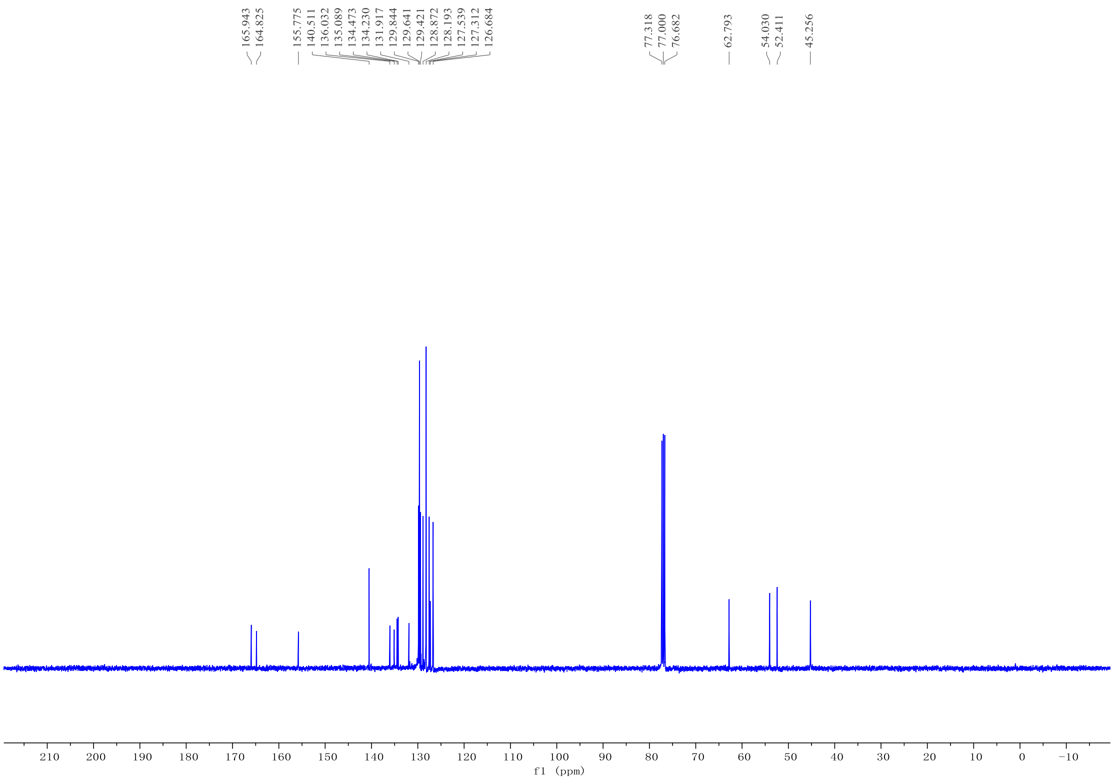

## $^{1}$H and $^{13}$C NMR Spectra for Compound (*Z*)-3ag:

$^{1}$H NMR (400 MHz, $CDCl_3$)

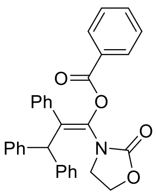

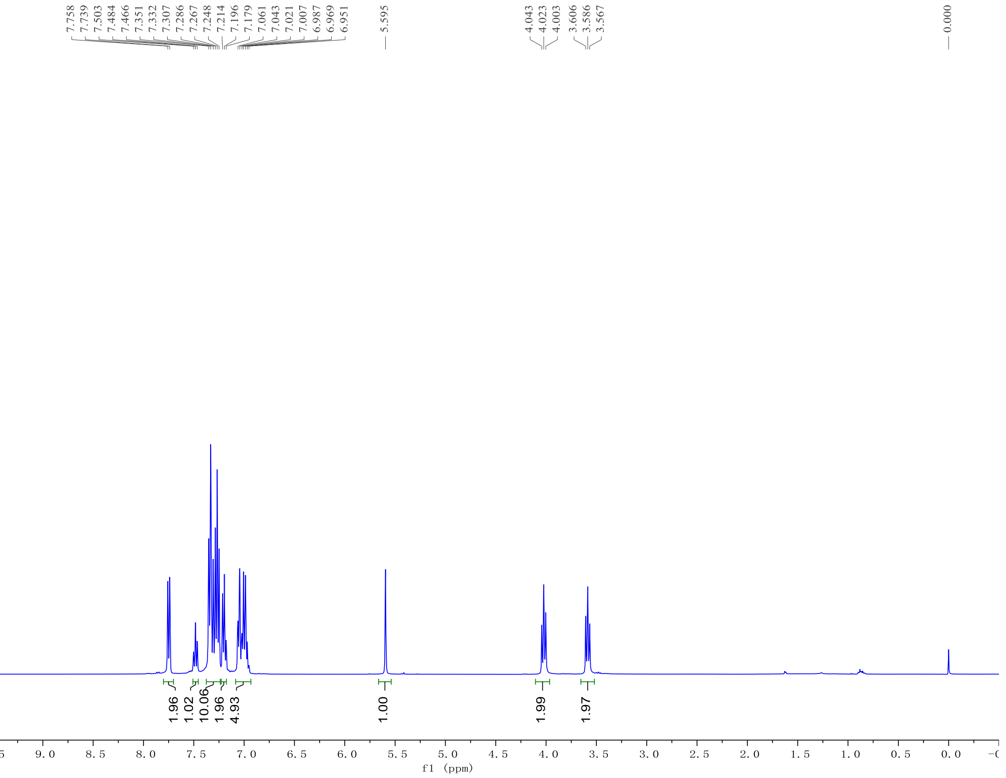

$^{13}$C NMR (100 MHz, $CDCl_3$)

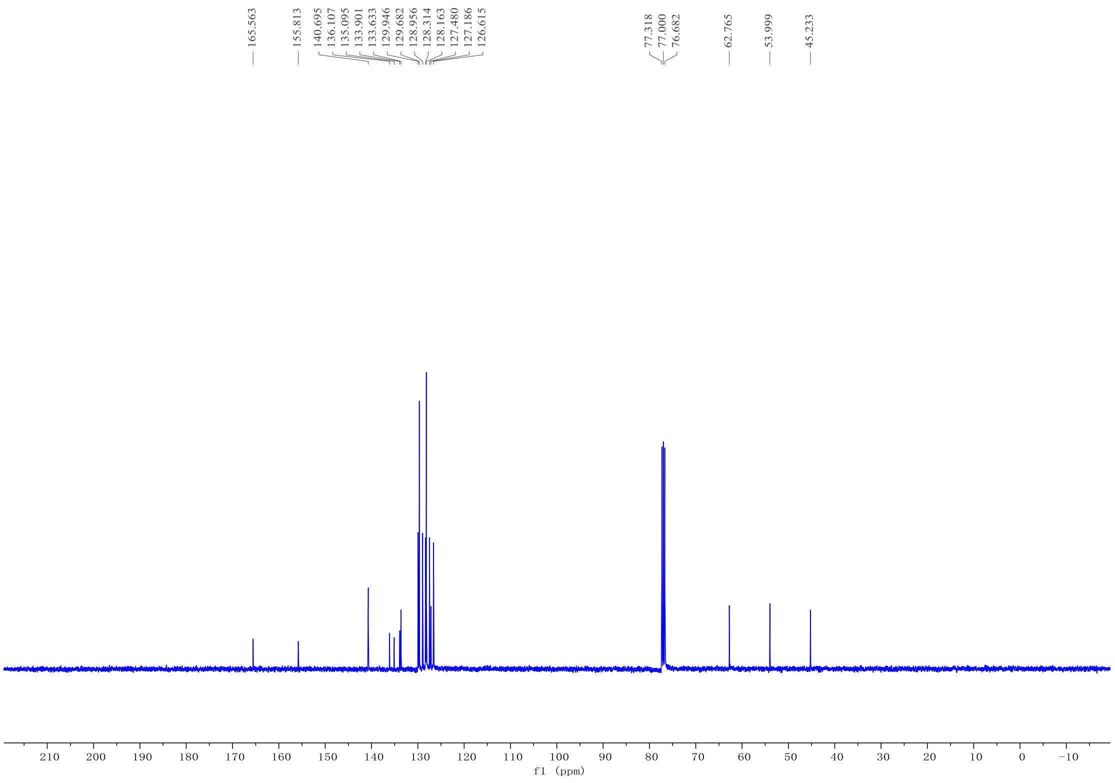

**$^{1}$H and $^{13}$C NMR Spectra for Compound (*Z*/*E*)-3ah**:

$^{1}$H NMR (400 MHz, CDCl$_3$)

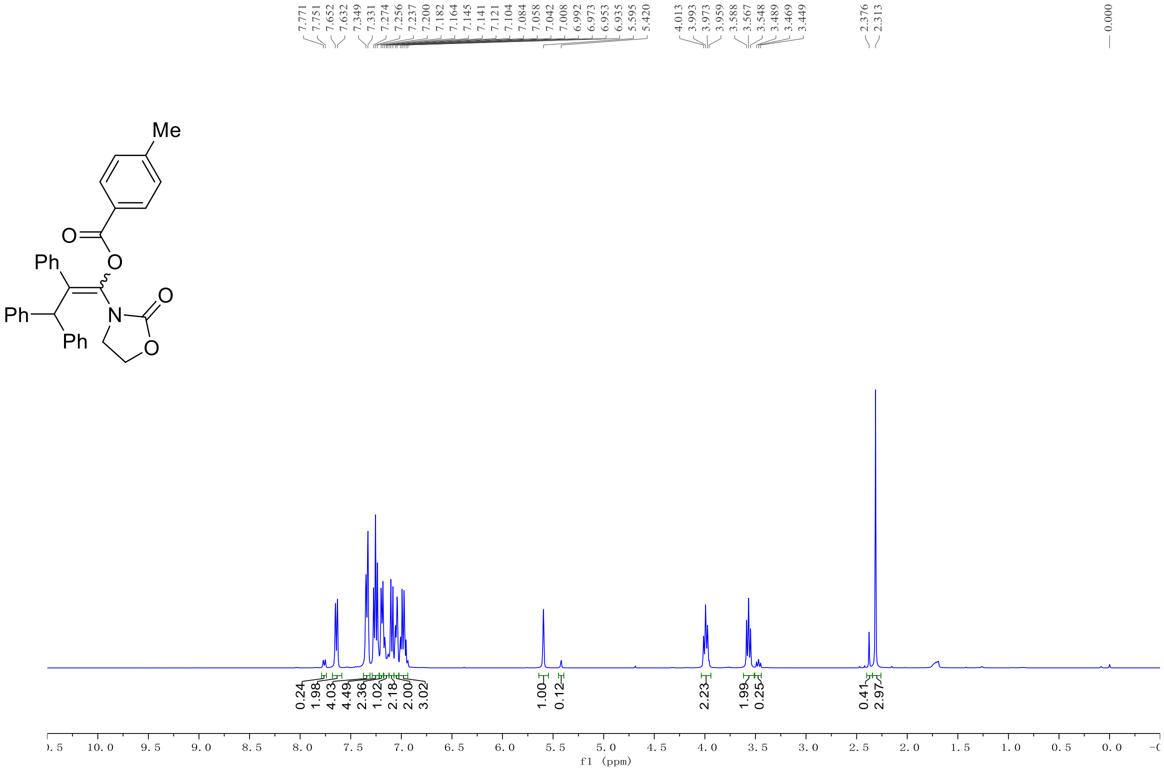


$^{13}$C NMR (100 MHz, CDCl$_3$)

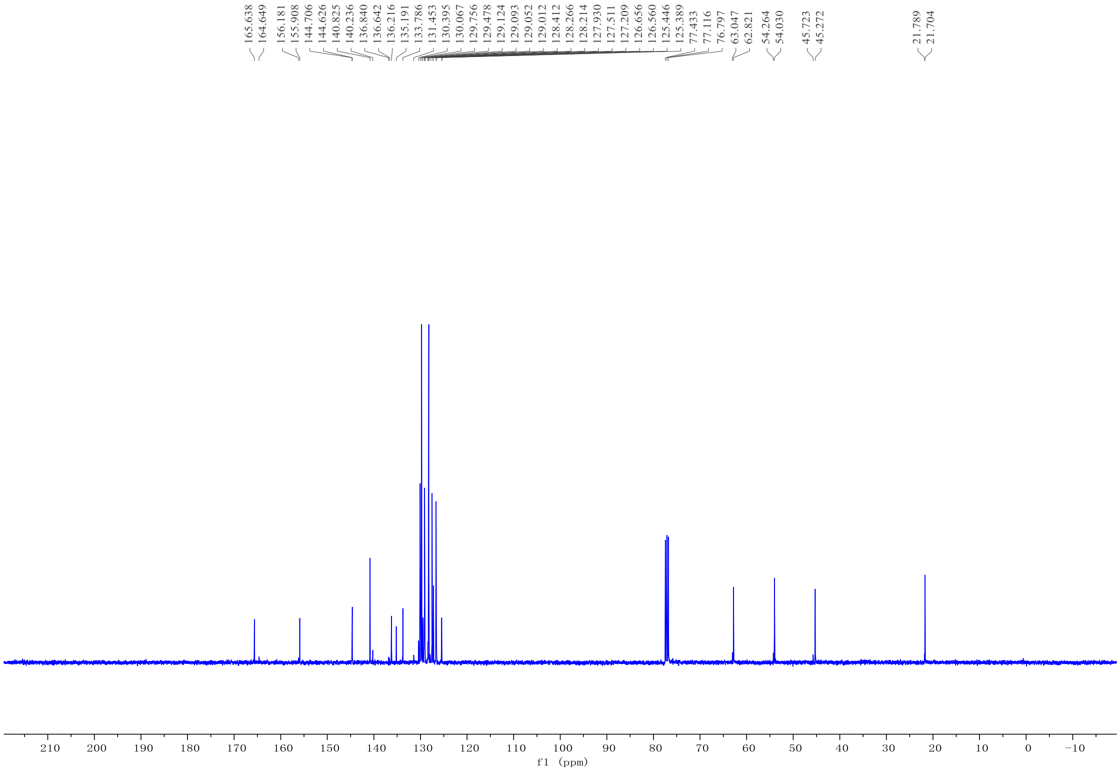

# $^{1}H$ and $^{13}C$ NMR Spectra for Compound (*Z*/*E*)-3ai:

## $^{1}H$ NMR (400 MHz, $CDCl_3$)

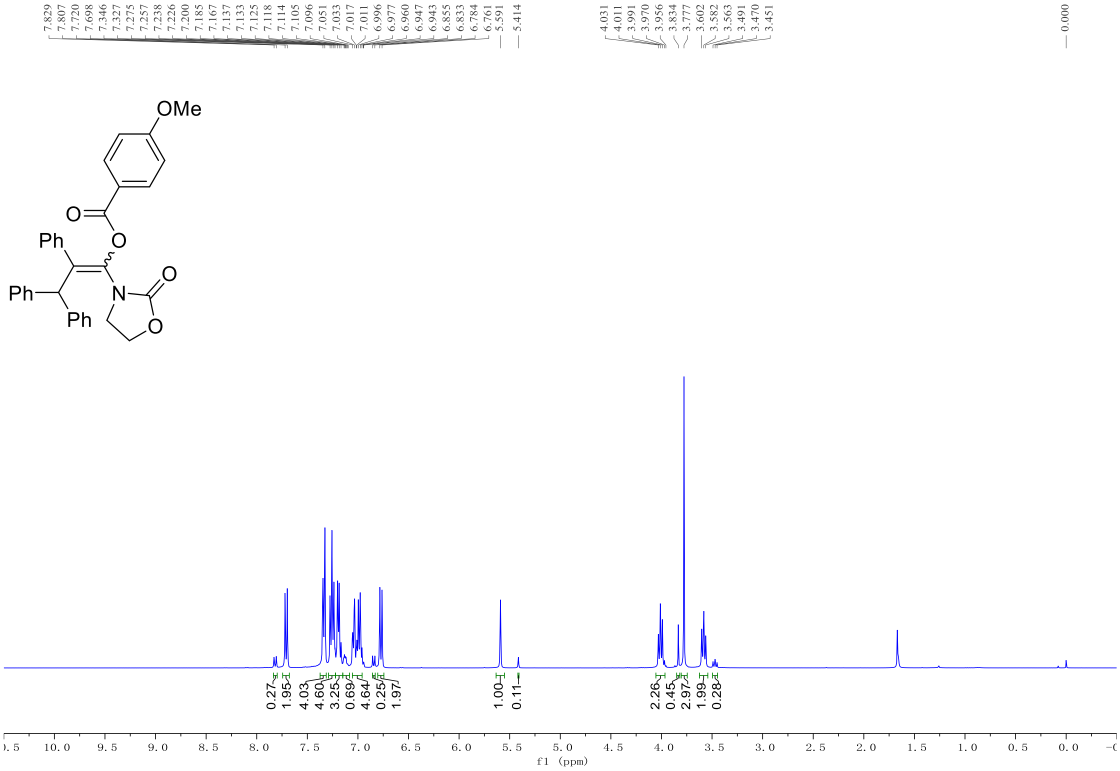


## $^{13}C$ NMR (100 MHz, $CDCl_3$)

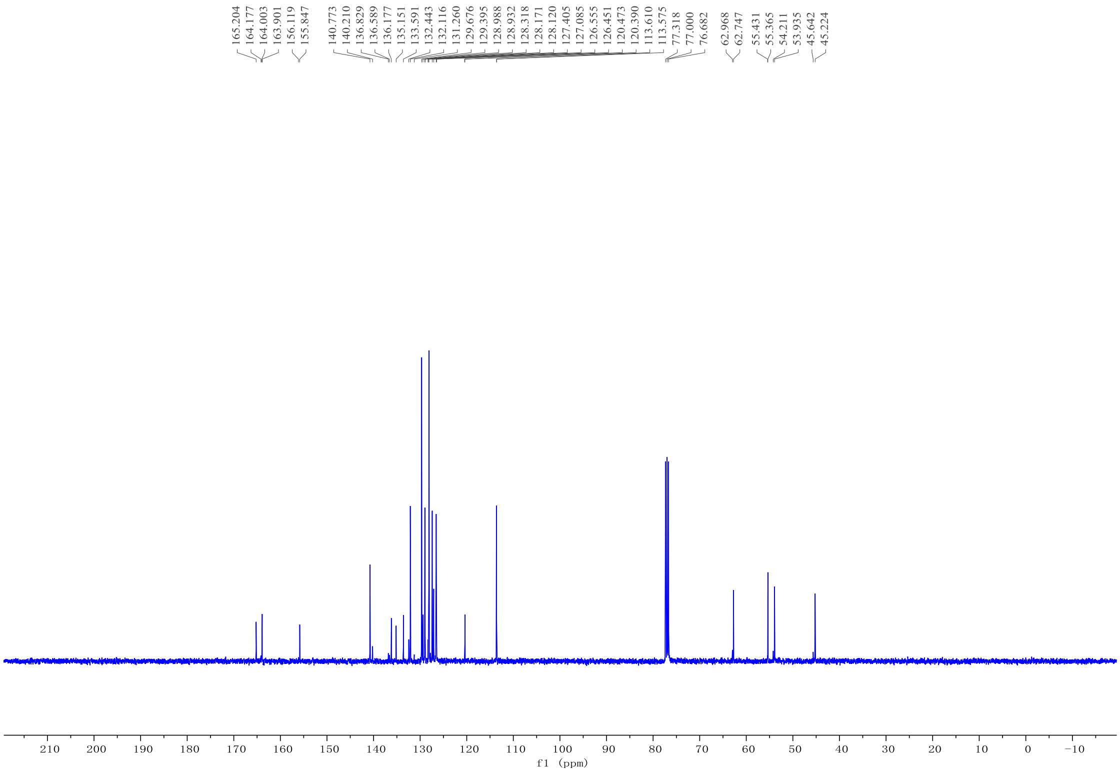

# $^{1}$H and $^{13}$C NMR Spectra for Compound (*Z*/*E*)-3aj:

$^{1}$H NMR (400 MHz, $CDCl_3$)

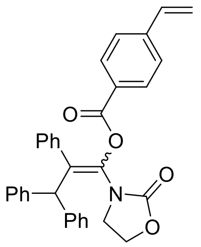


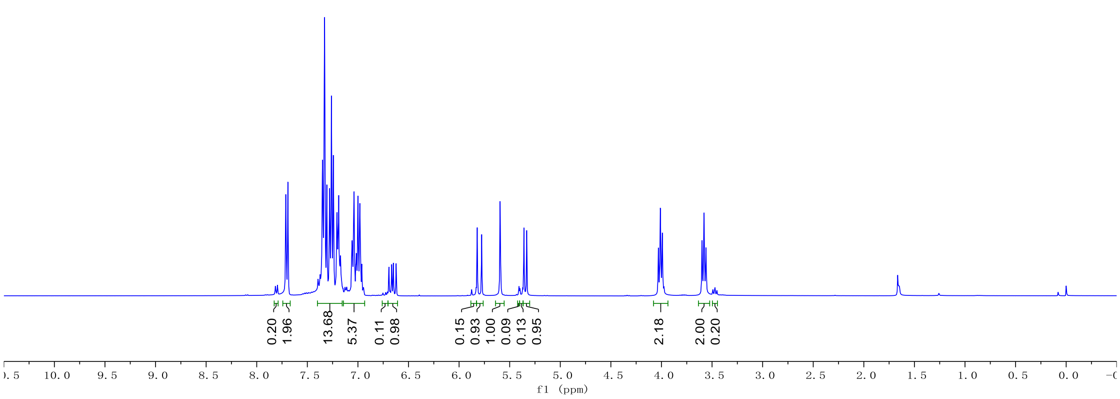

$^{13}$C NMR (100 MHz, $CDCl_3$)

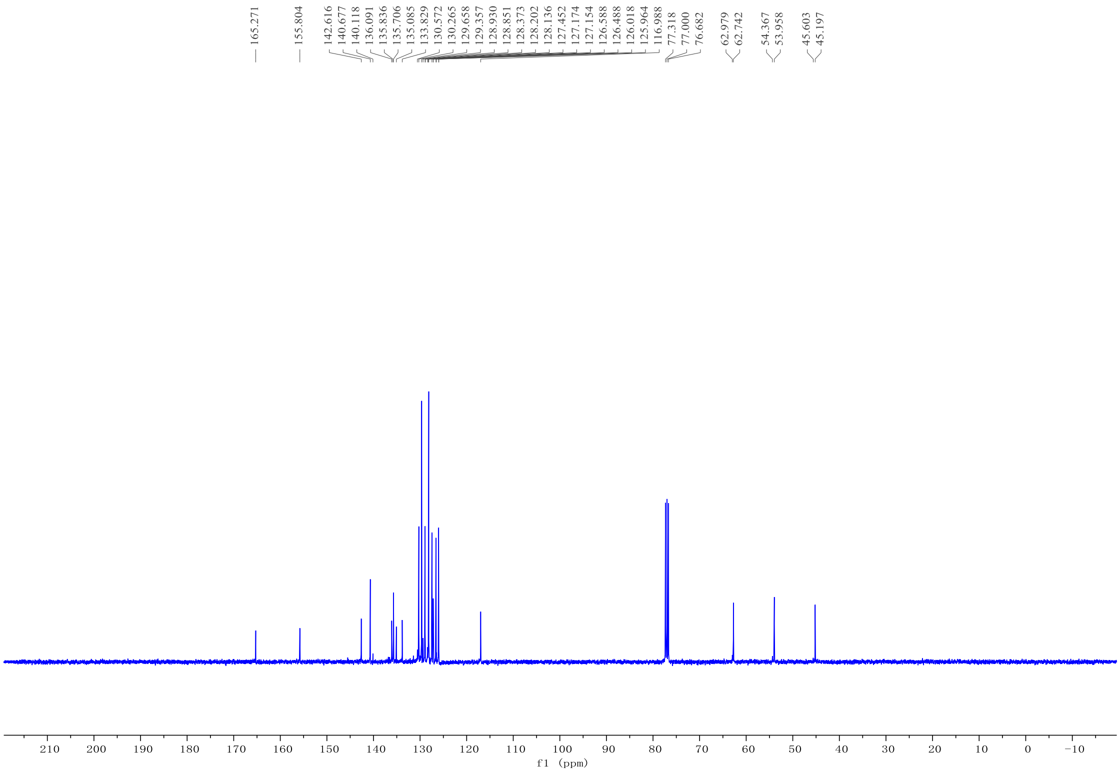

## $^{1}$H and $^{13}$C NMR Spectra for Compound (*Z*)-3ak:

### $^{1}$H NMR (400 MHz, $CDCl_3$)

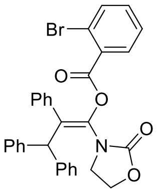


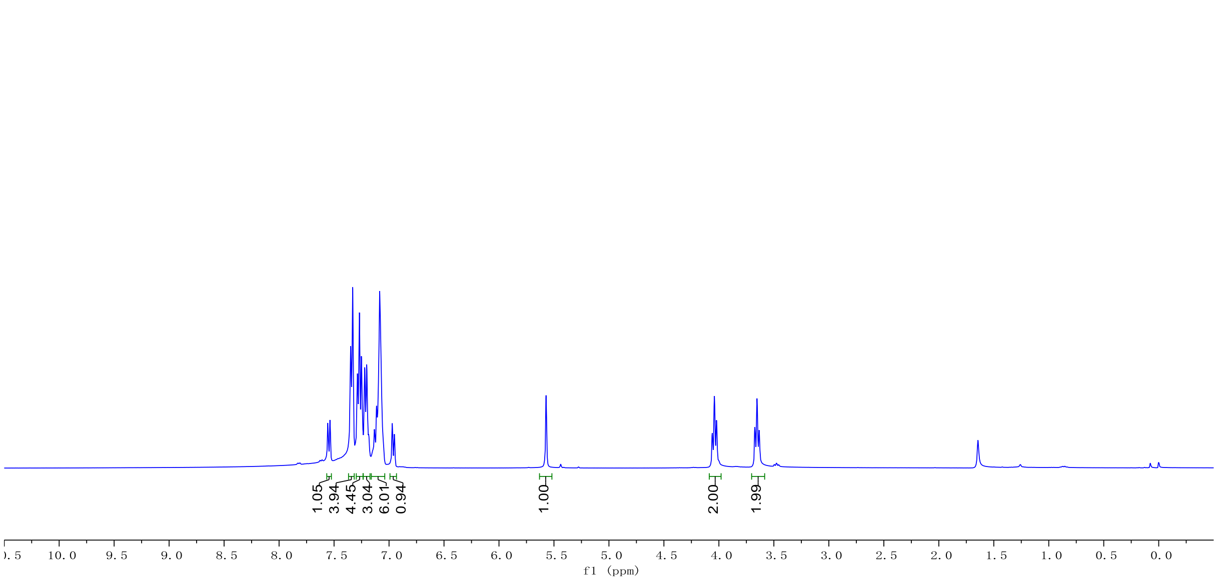


### $^{13}$C NMR (100 MHz, $CDCl_3$)

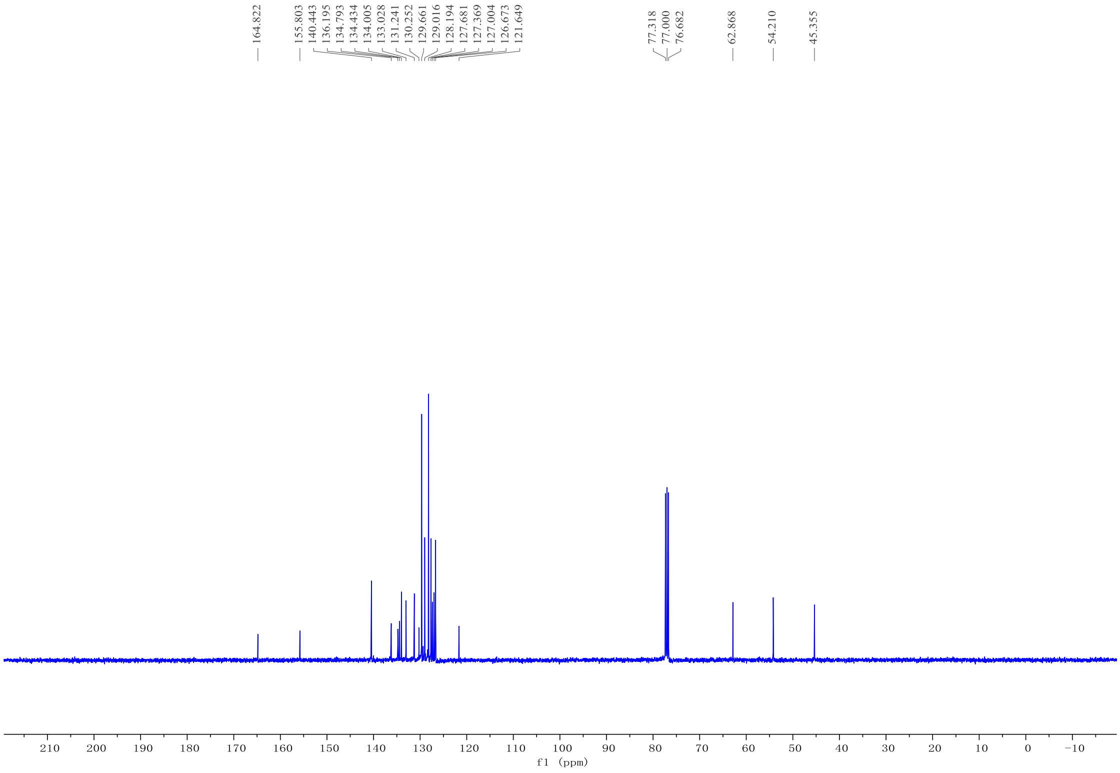

**$^{1}$H and $^{13}$C NMR Spectra for Compound (*Z*)-3al**:

$^{1}$H NMR (400 MHz, $CDCl_3$)

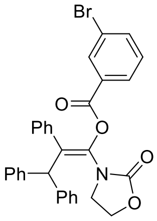

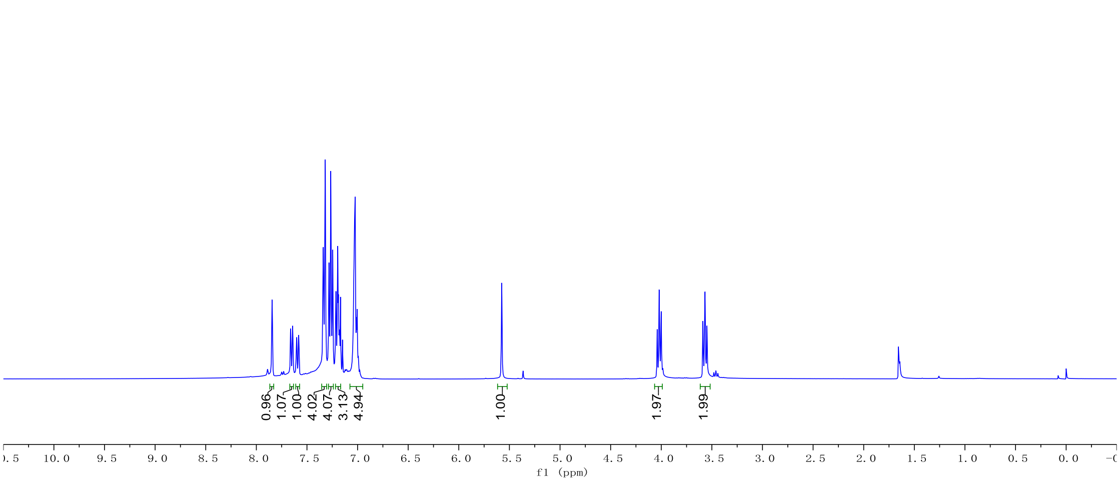

$^{13}$C NMR (100 MHz, $CDCl_3$)

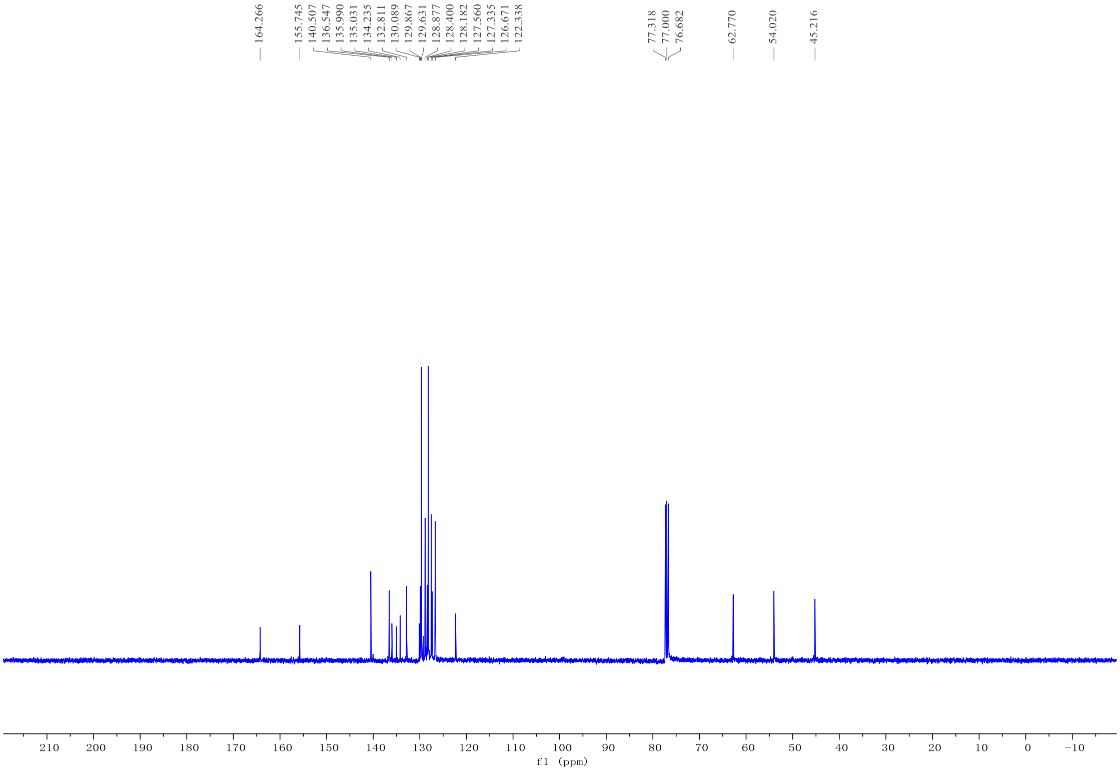

## **$^{1}$H and $^{13}$C NMR Spectra for Compound (*Z*/*E*)-3am**:

### $^{1}$H NMR (400 MHz, $CDCl_3$)

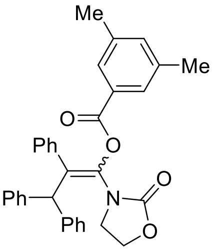


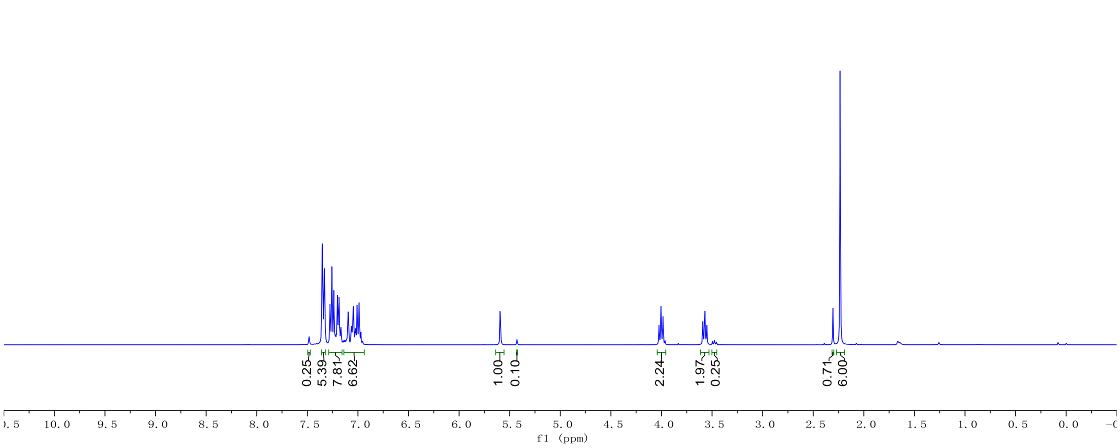


### $^{13}$C NMR (100 MHz, $CDCl_3$)

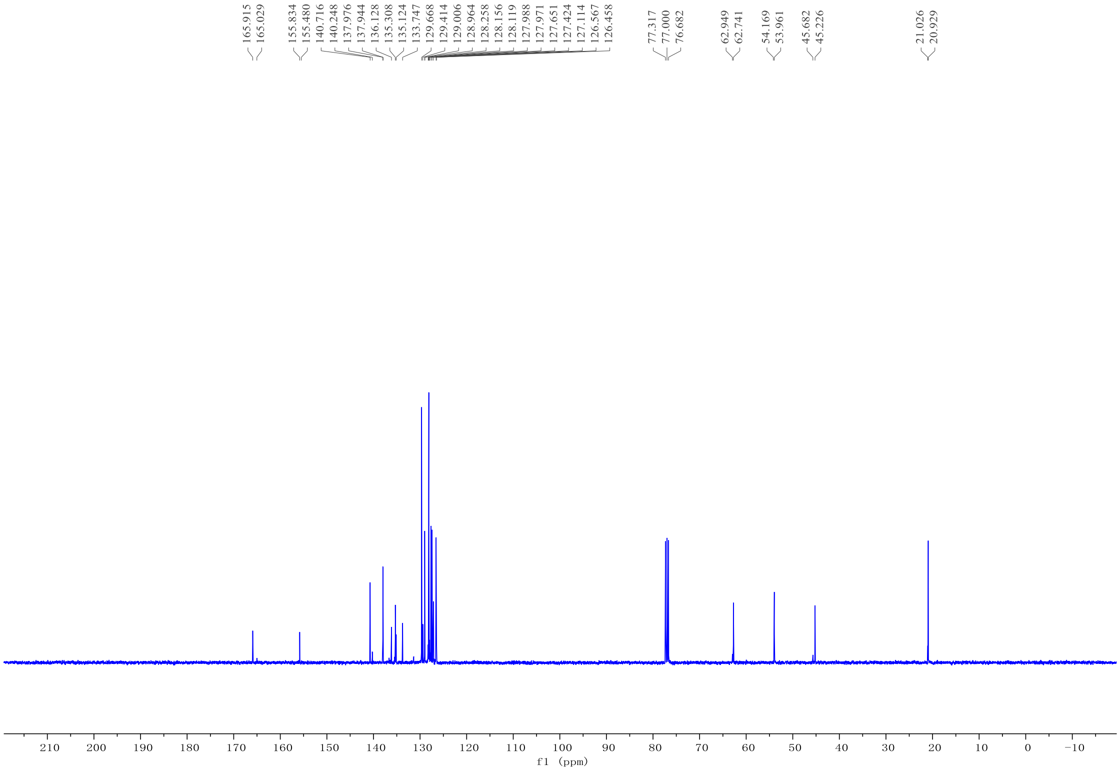

## $^{1}$H and $^{13}$C NMR Spectra for Compound (*Z*)-3an:

$^{1}$H NMR (400 MHz, $CDCl_3$)

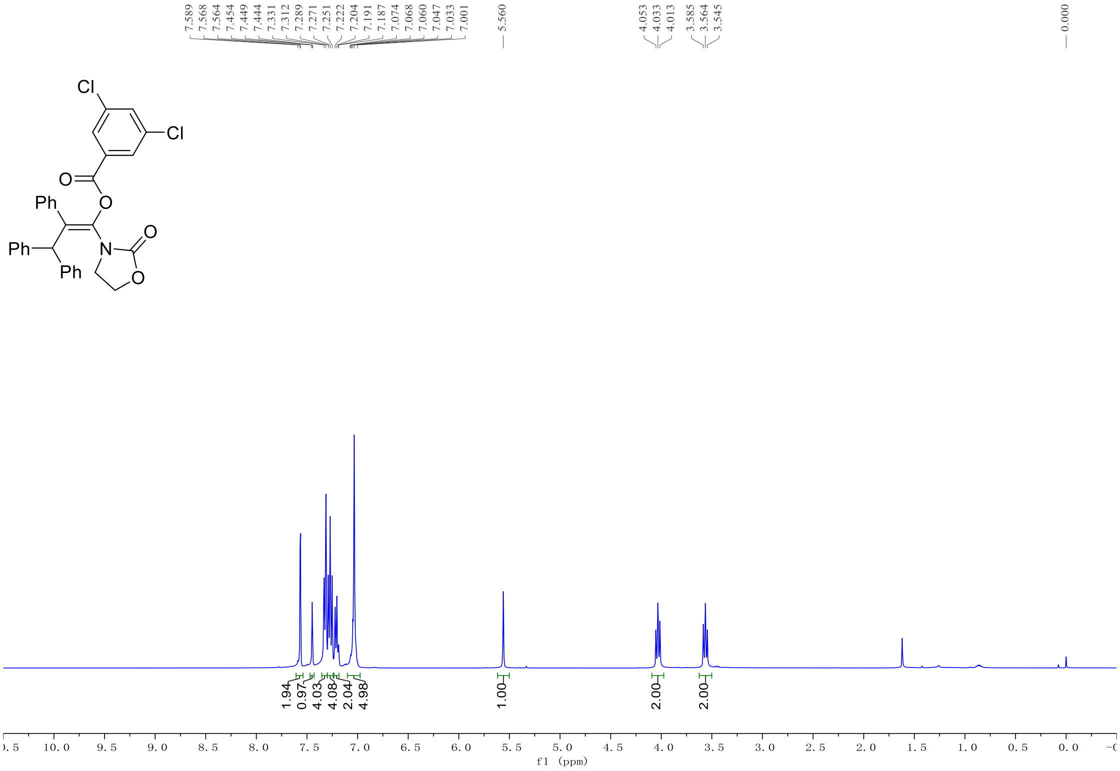

$^{13}$C NMR (100 MHz, $CDCl_3$)

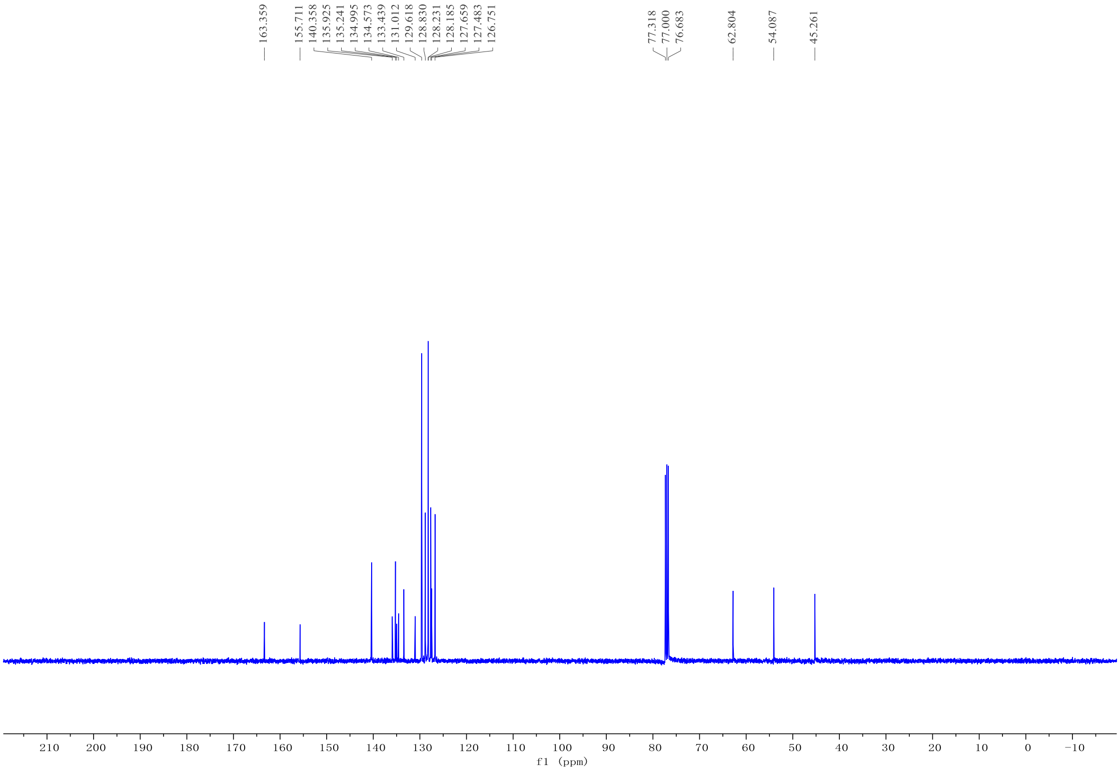

## $^{1}$H, $^{13}$C NMR and $^{19}$F NMR Spectra for Compound (*Z*)-3ao:

### $^{1}$H NMR (400 MHz, $CDCl_3$)

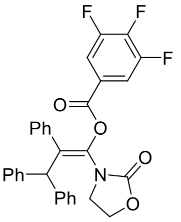


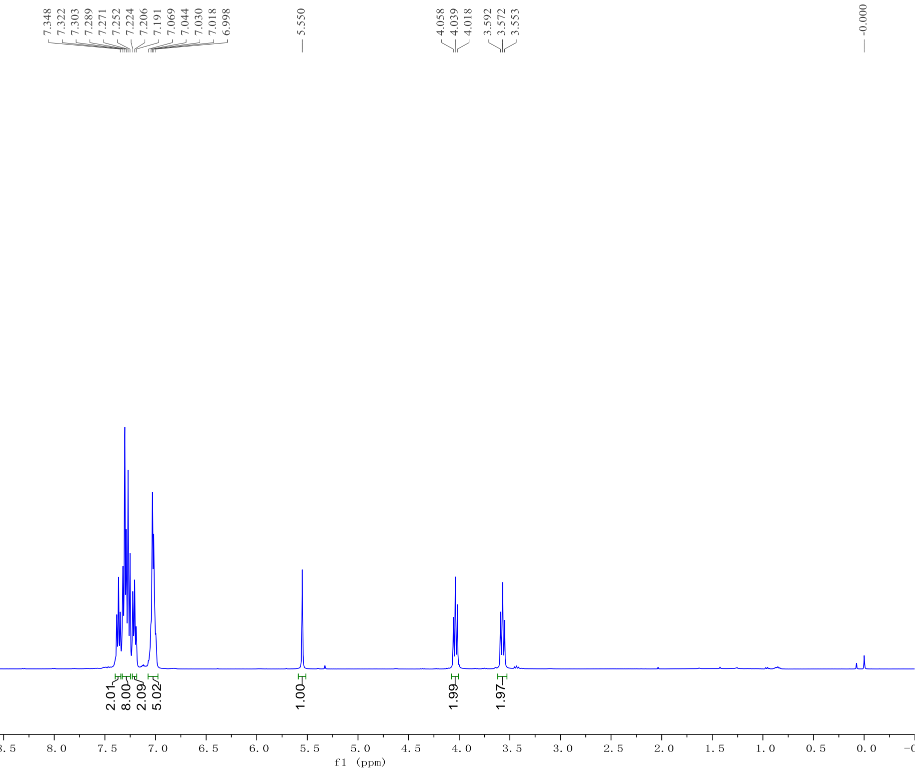


### $^{13}$C NMR (100 MHz, $CDCl_3$)

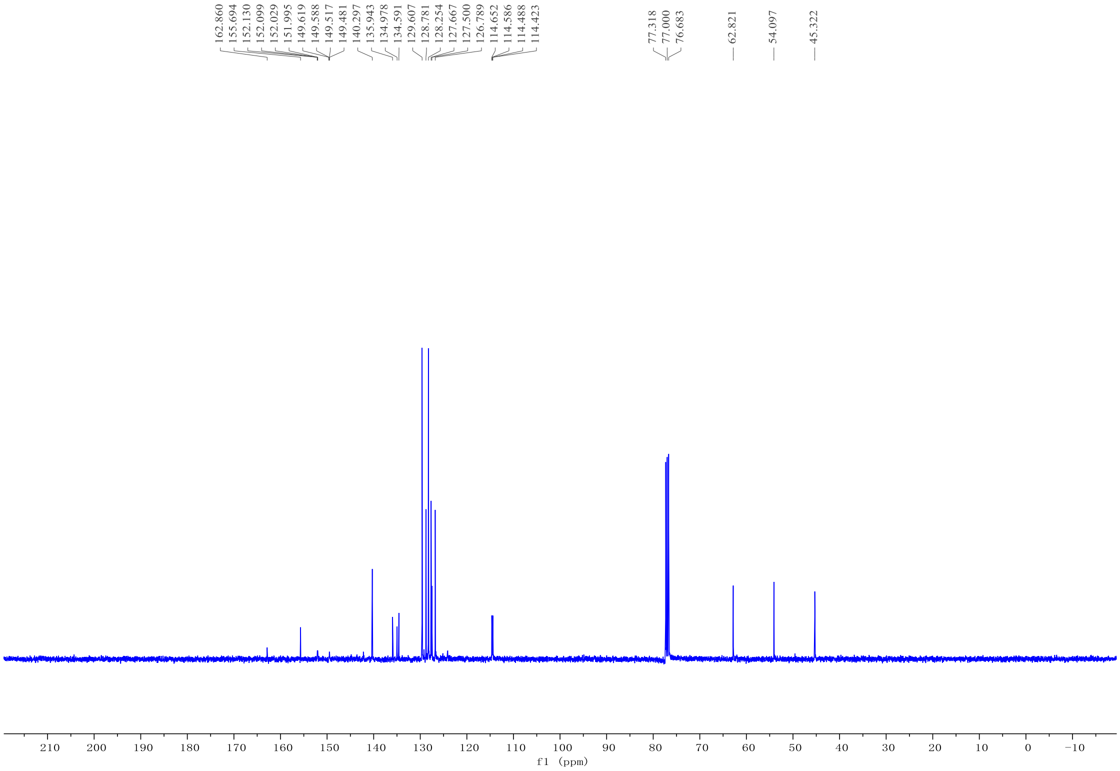

$^{19}$F NMR (376 MHz, $CDCl_3$)

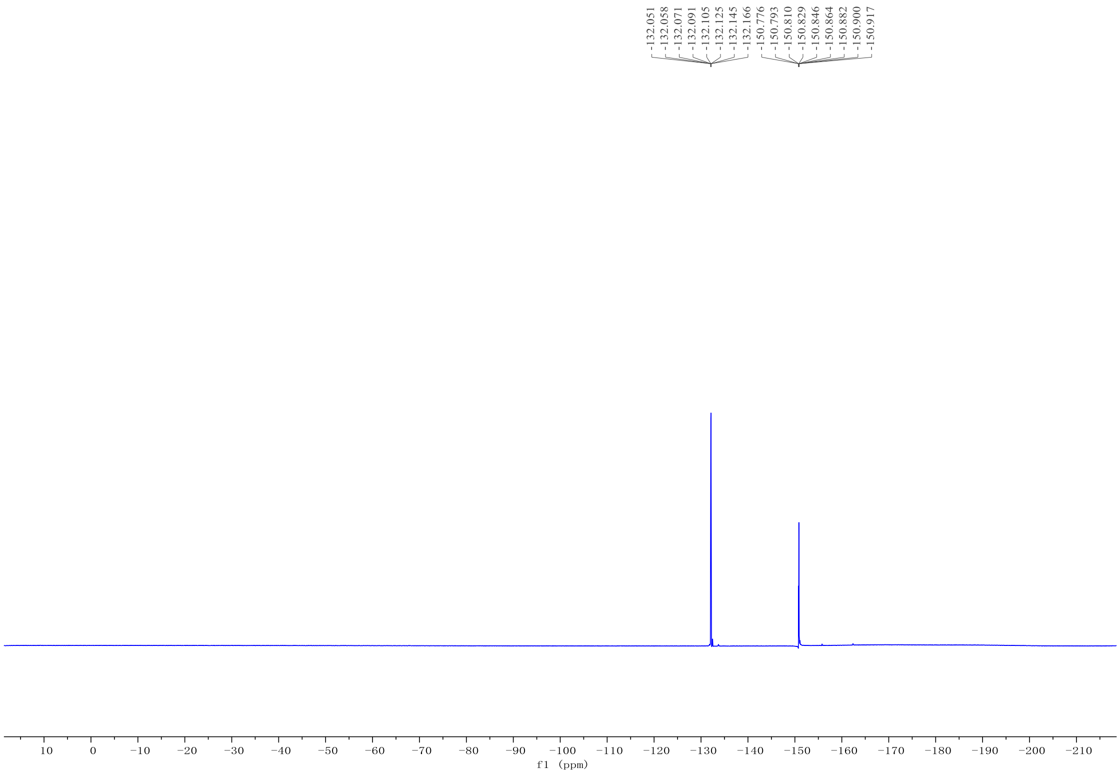


**$^{1}$H, $^{13}$C NMR and $^{19}$F NMR Spectra for Compound (*Z*)-3ap**:

$^{1}$H NMR (400 MHz, $CDCl_3$)

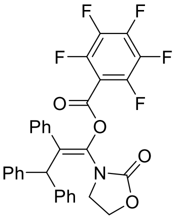


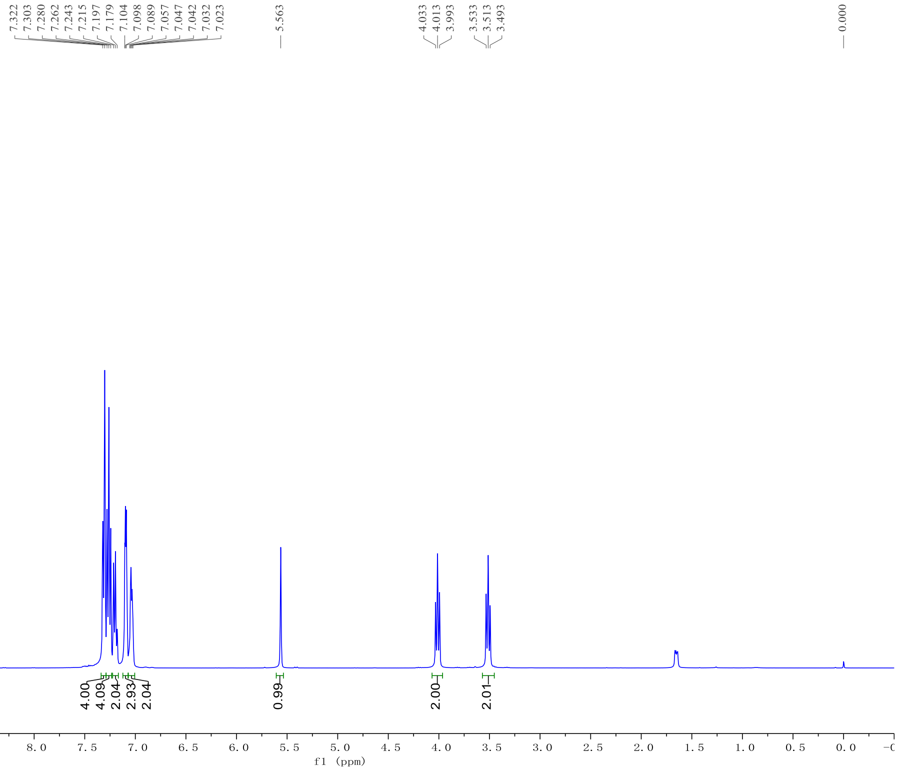

$^{13}C$ NMR (100 MHz, $CDCl_3$)

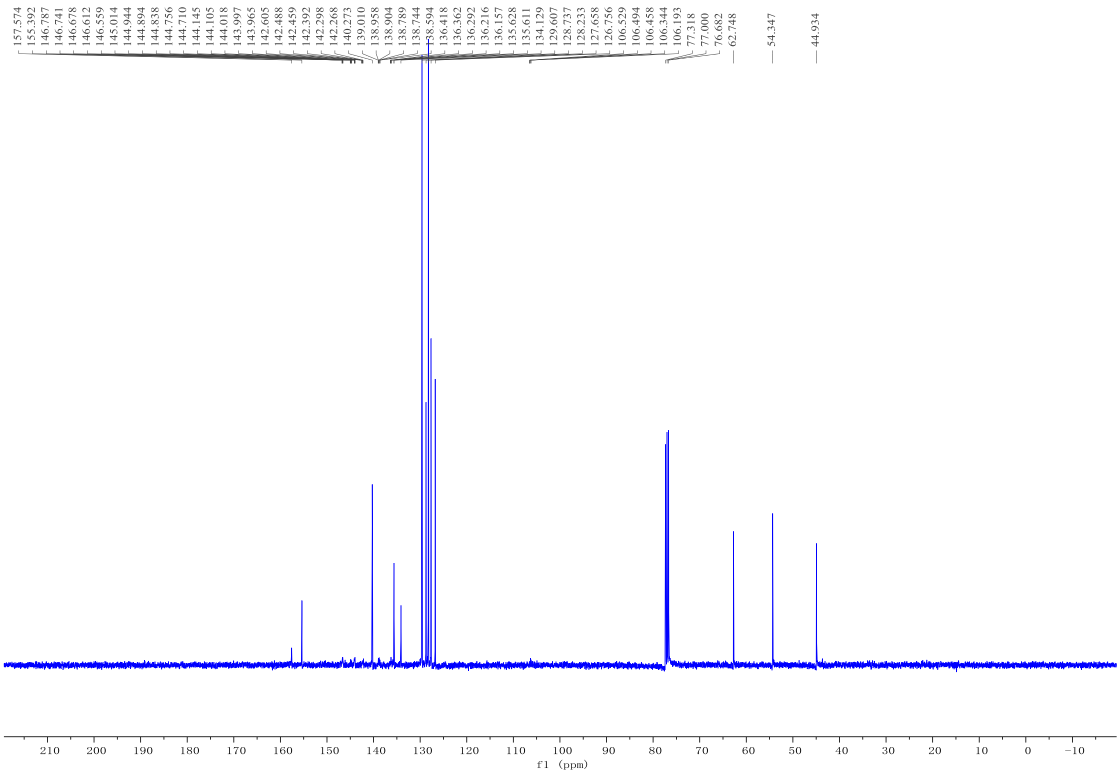


$^{19}F$ NMR (376 MHz, $CDCl_3$)

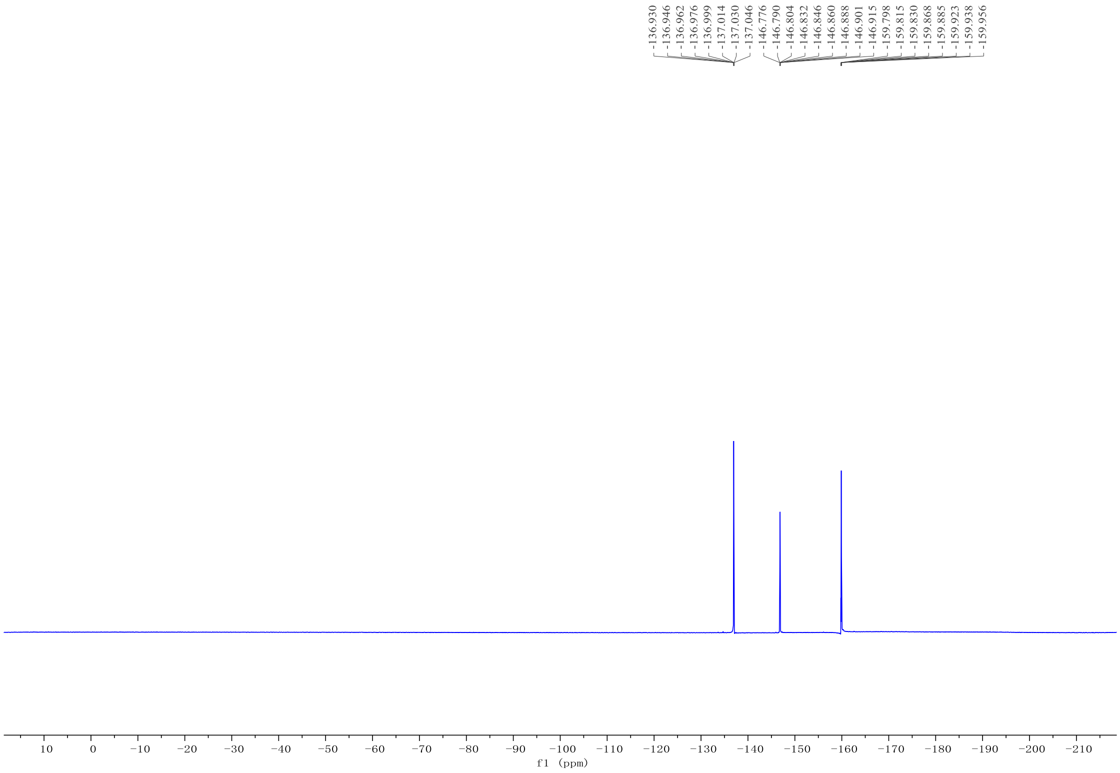

# $^{1}$H and $^{13}$C NMR Spectra for Compound (*Z*/*E*)-3aq:

## $^{1}$H NMR (400 MHz, $CDCl_3$)

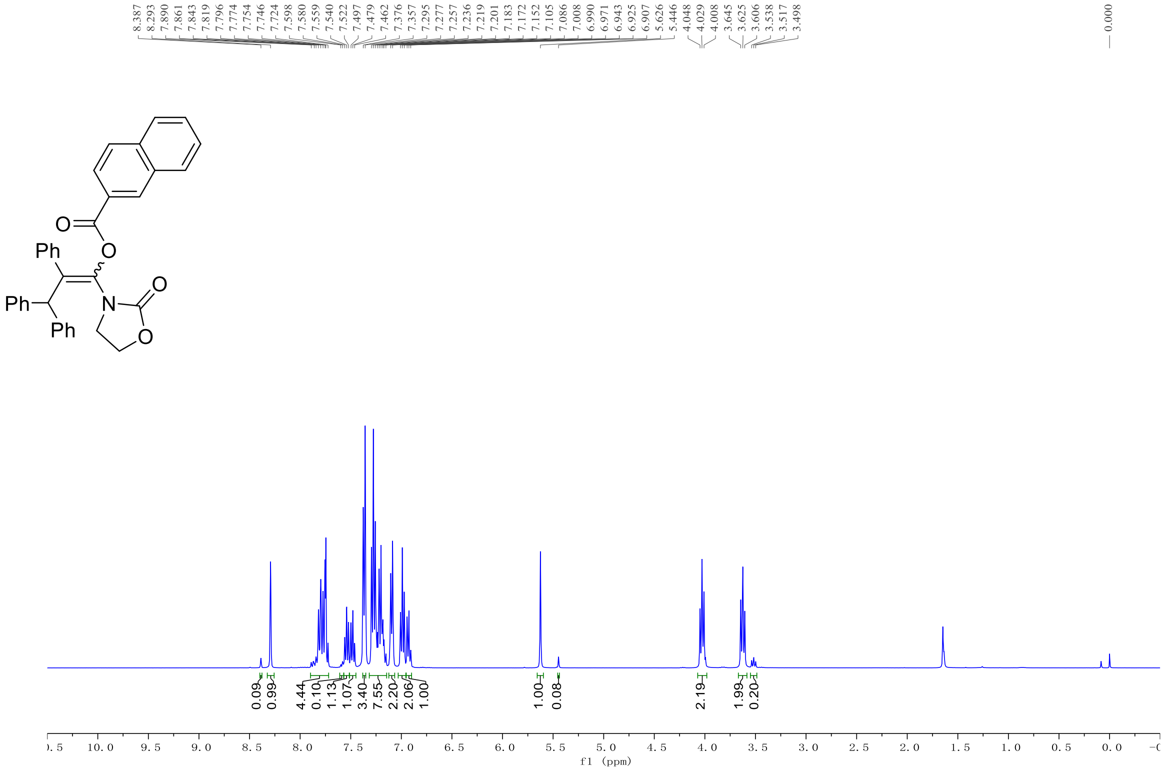


## $^{13}$C NMR (100 MHz, $CDCl_3$)

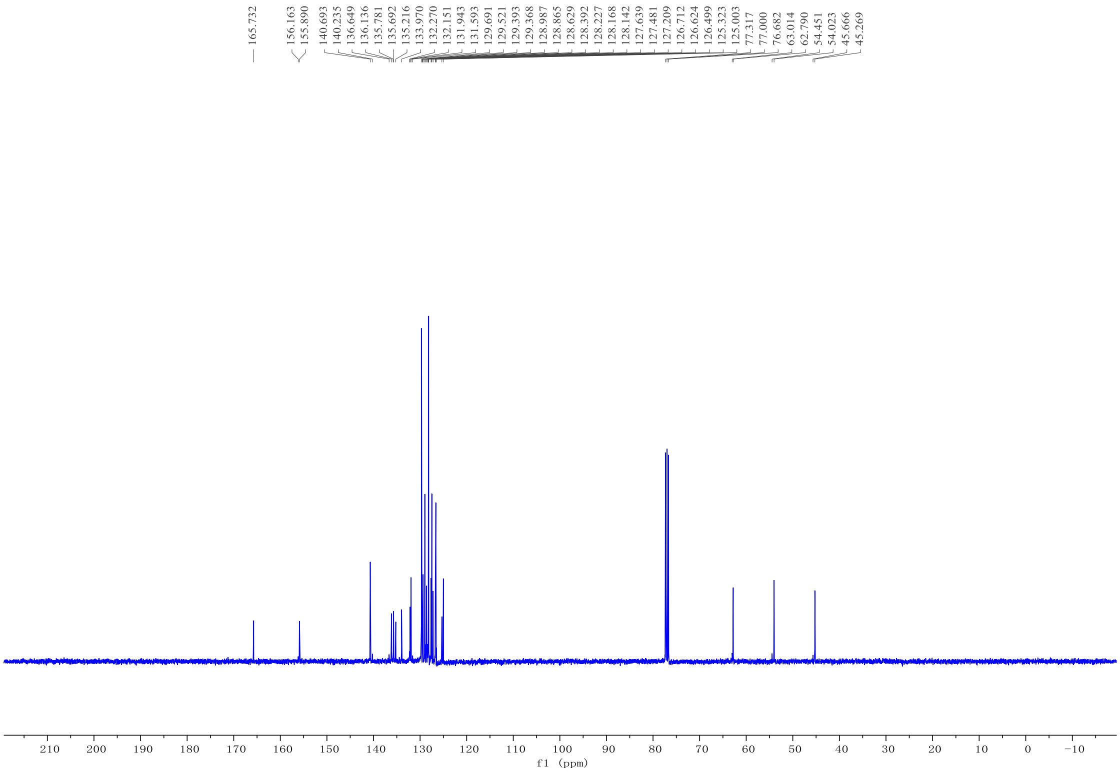

## ¹H and ¹³C NMR Spectra for Compound (*Z*/*E*)-3ar:

¹H NMR (400 MHz, $CDCl_3$)

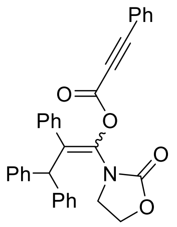


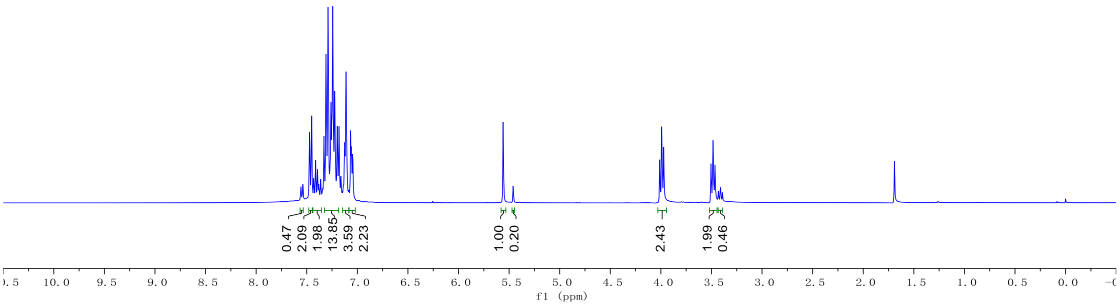


¹³C NMR (100 MHz, $CDCl_3$)

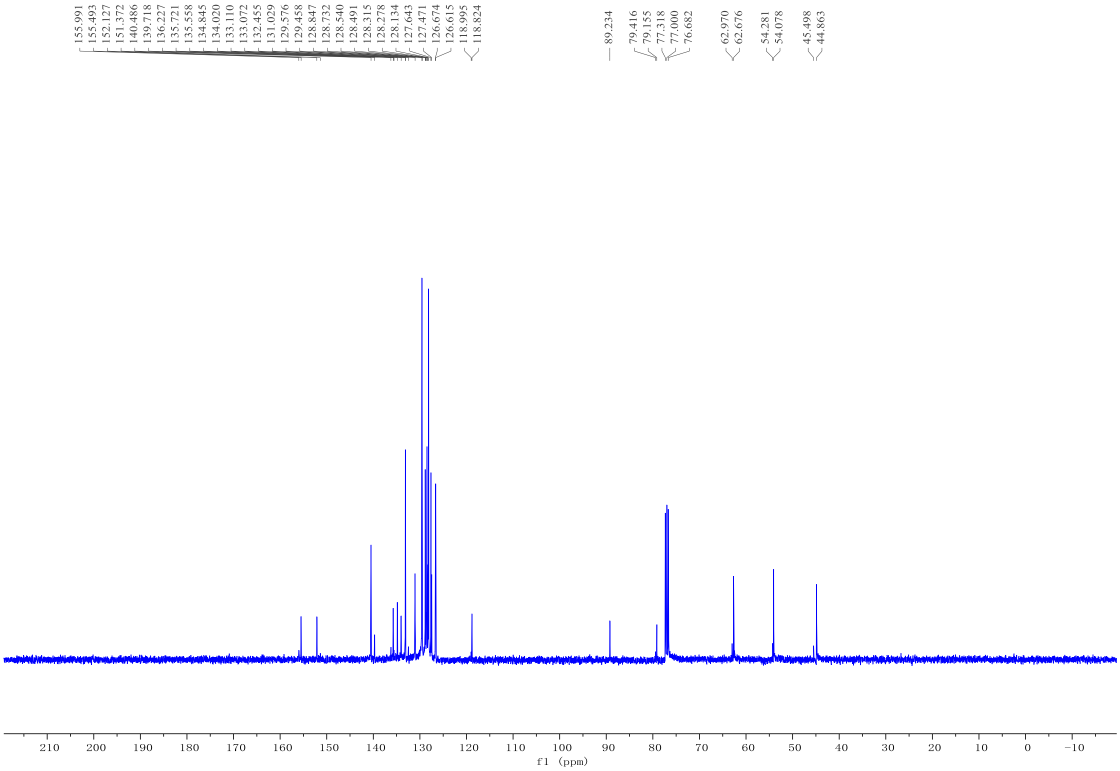

# $^{1}$H and $^{13}$C NMR Spectra for Compound (*Z*)-3as:

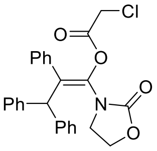


## $^{1}$H NMR (400 MHz, $CDCl_3$)

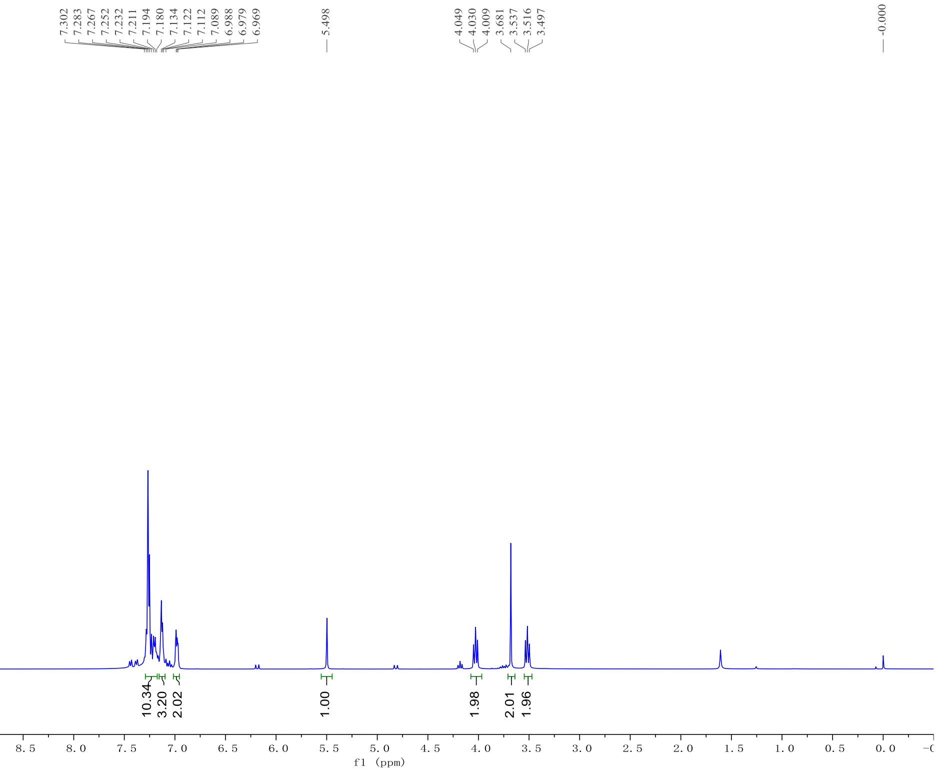


## $^{13}$C NMR (100 MHz, $CDCl_3$)

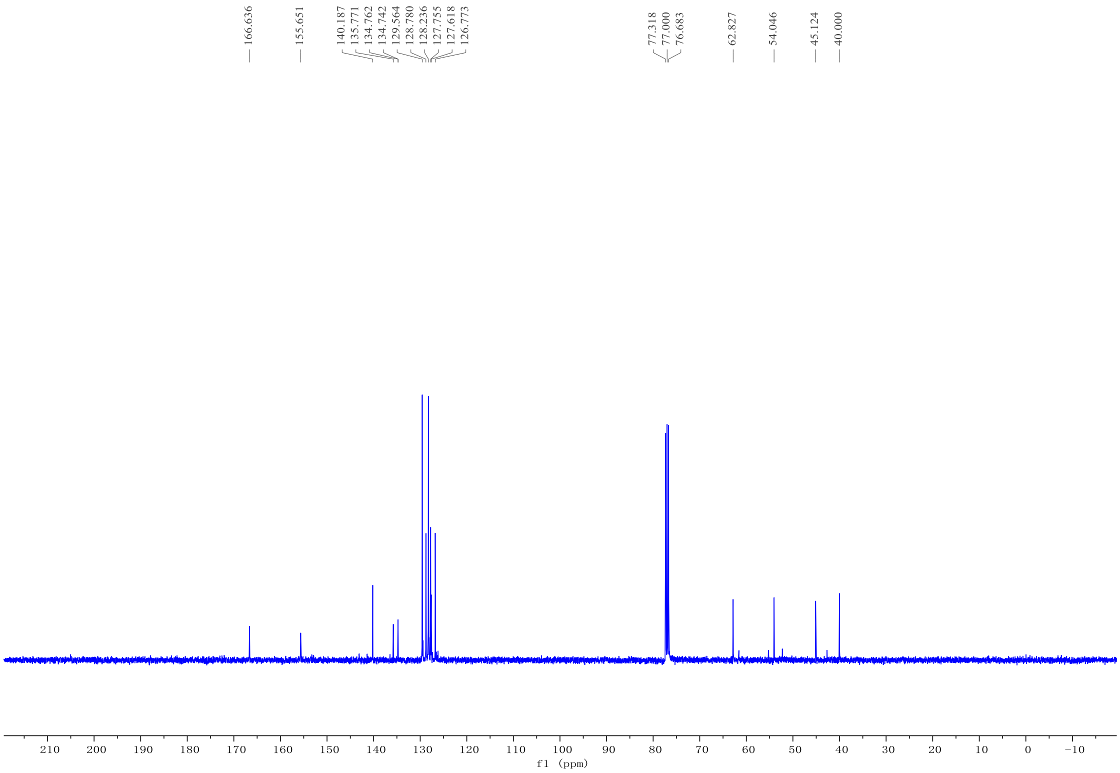

## $^{1}$H and $^{13}$C NMR Spectra for Compound (*Z*)-3at:

$^{1}$H NMR (400 MHz, $CDCl_3$)

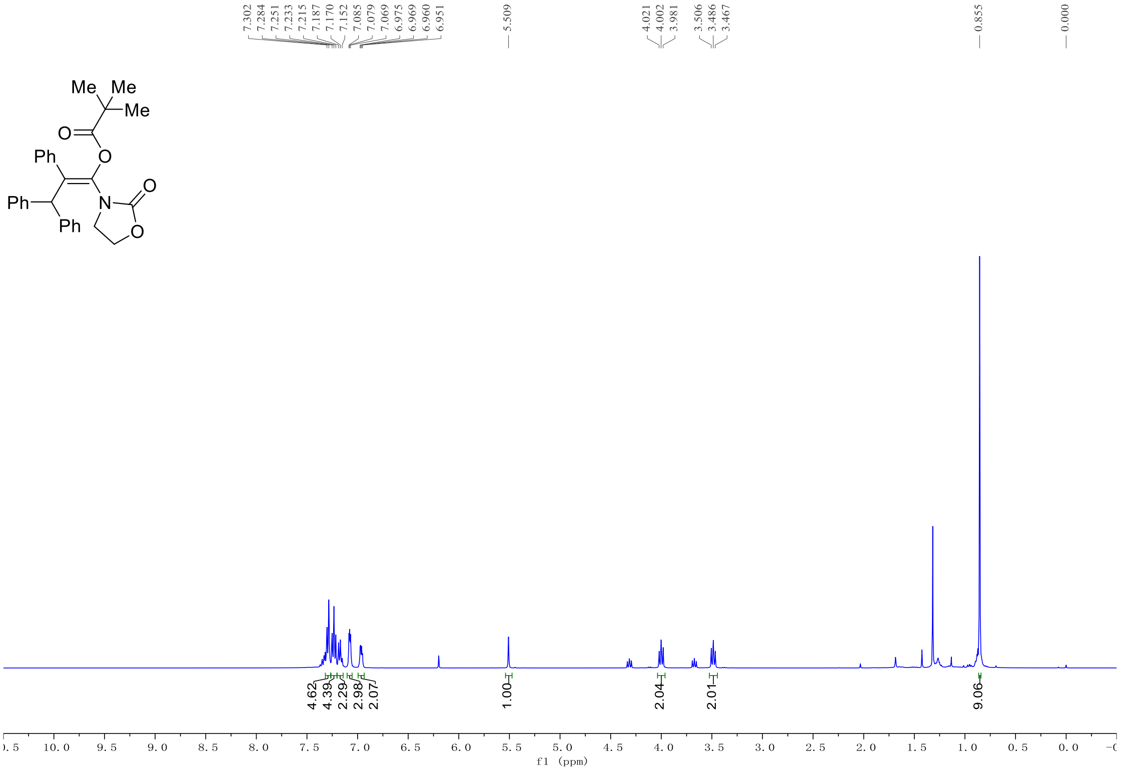


$^{13}$C NMR (100 MHz, $CDCl_3$)

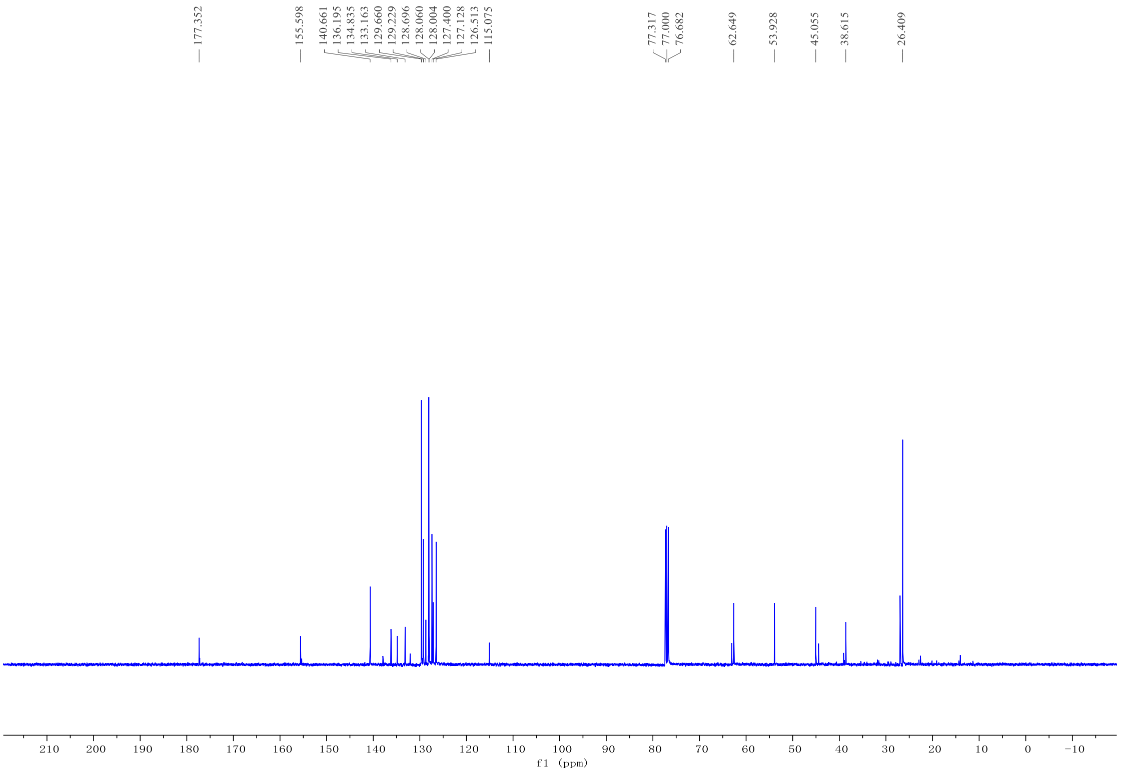

## $^{1}$H and $^{13}$C NMR Spectra for Compound (*Z*)-3au:

$^{1}$H NMR (400 MHz, $CDCl_3$)

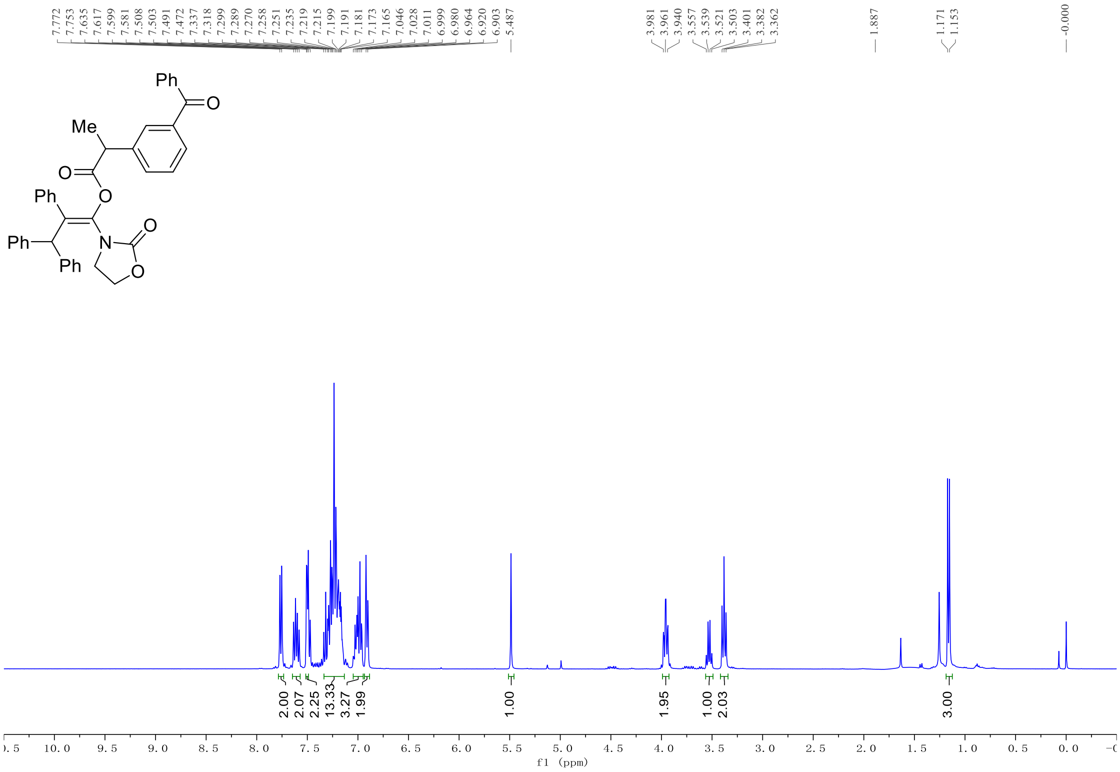


$^{13}$C NMR (100 MHz, $CDCl_3$)

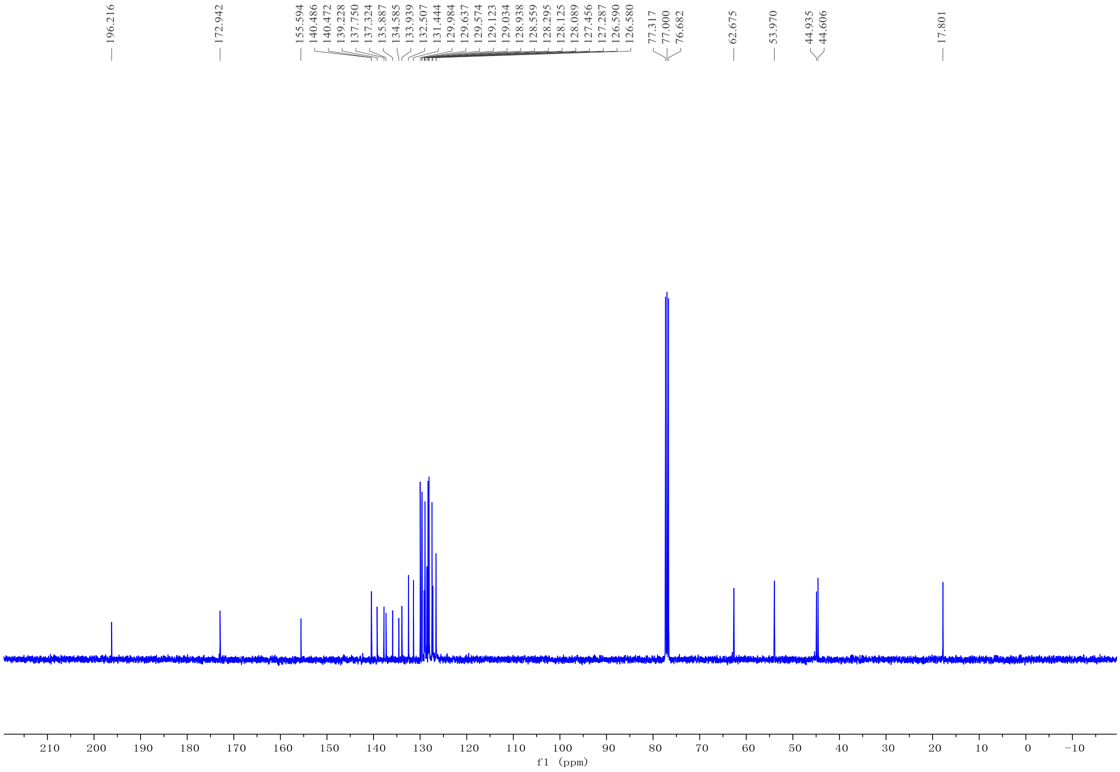

## $^{1}$H and $^{13}$C NMR Spectra for Compound (*Z*)-3av:

$^{1}$H NMR (400 MHz, $CDCl_3$)

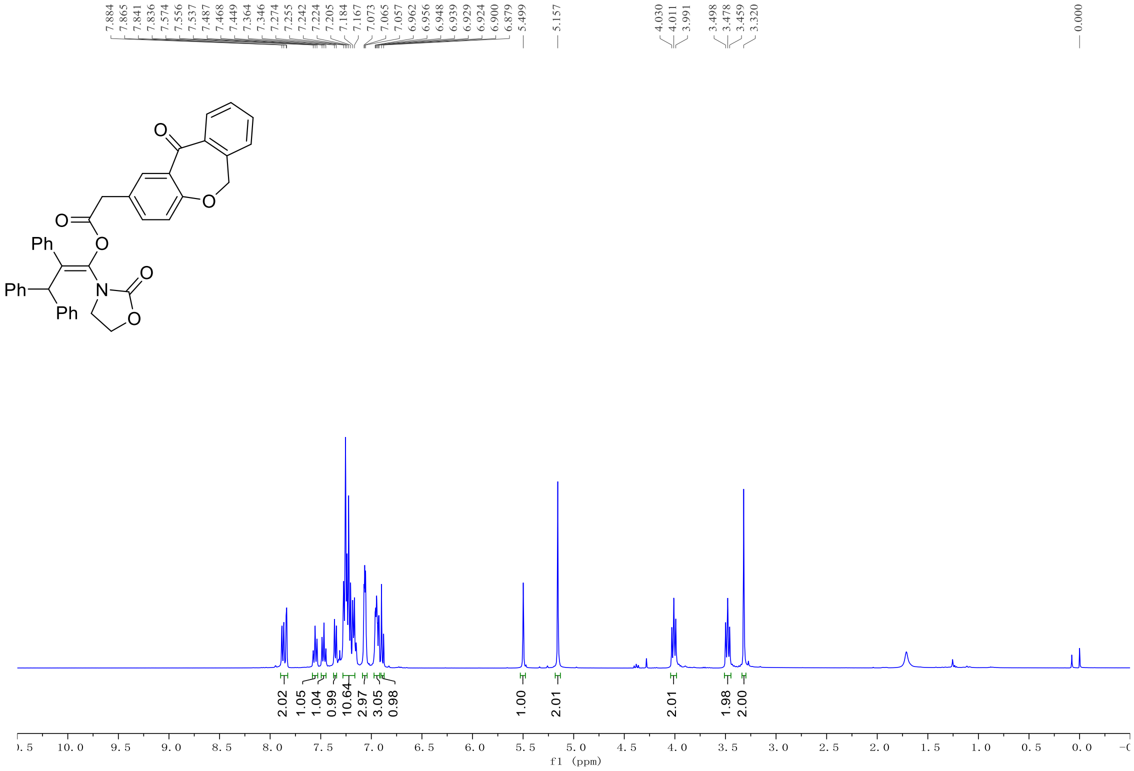


$^{13}$C NMR (100 MHz, $CDCl_3$)

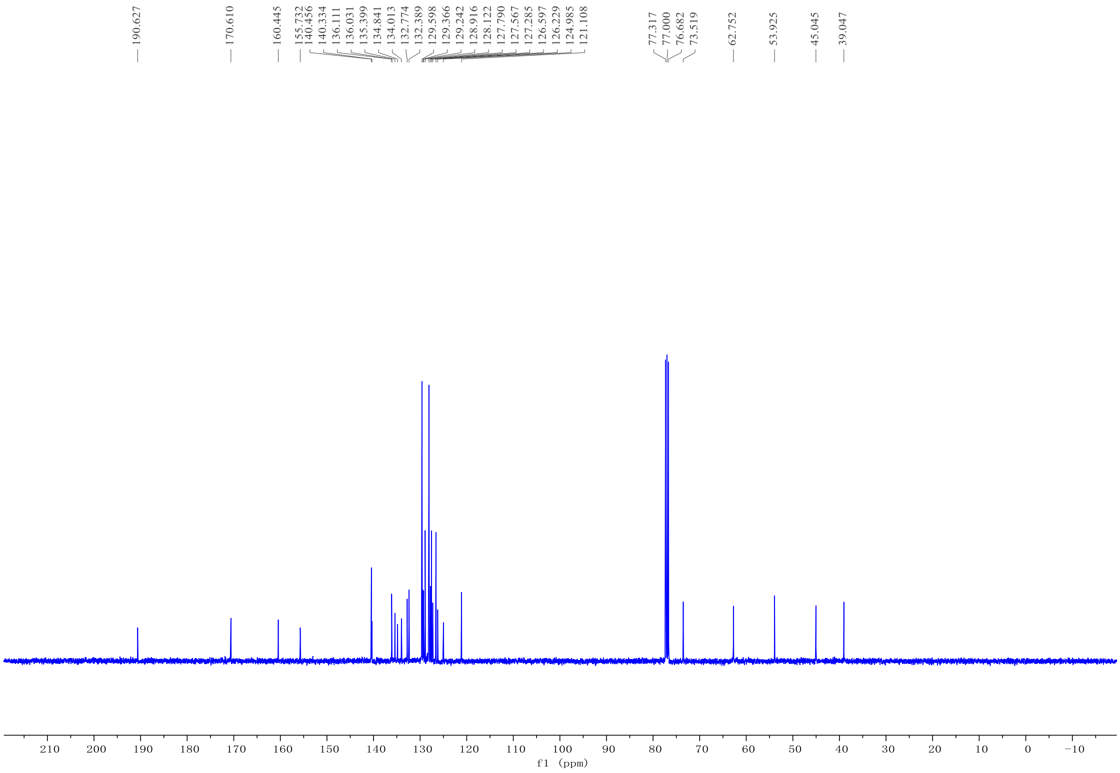

**$^{1}$H and $^{13}$C NMR Spectra for Compound (*Z*)-3aw**:

$^{1}$H NMR (400 MHz, $CDCl_3$)

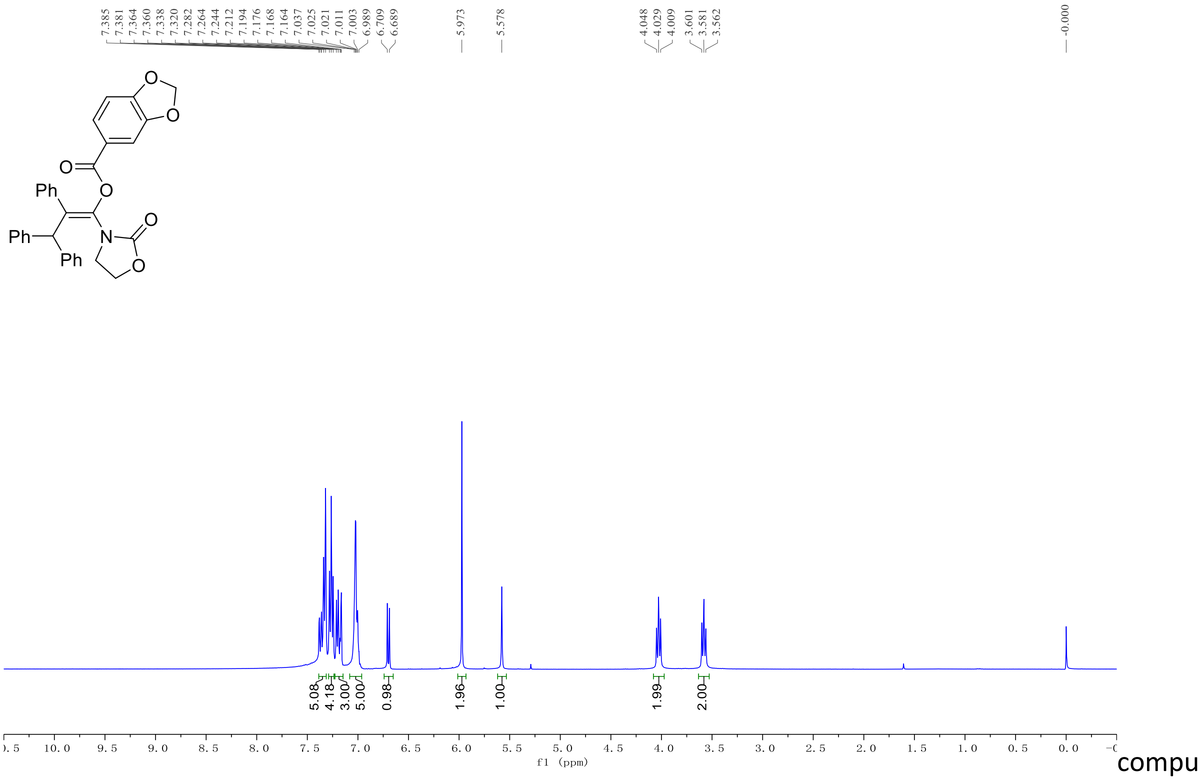


$^{13}$C NMR (100 MHz, $CDCl_3$)

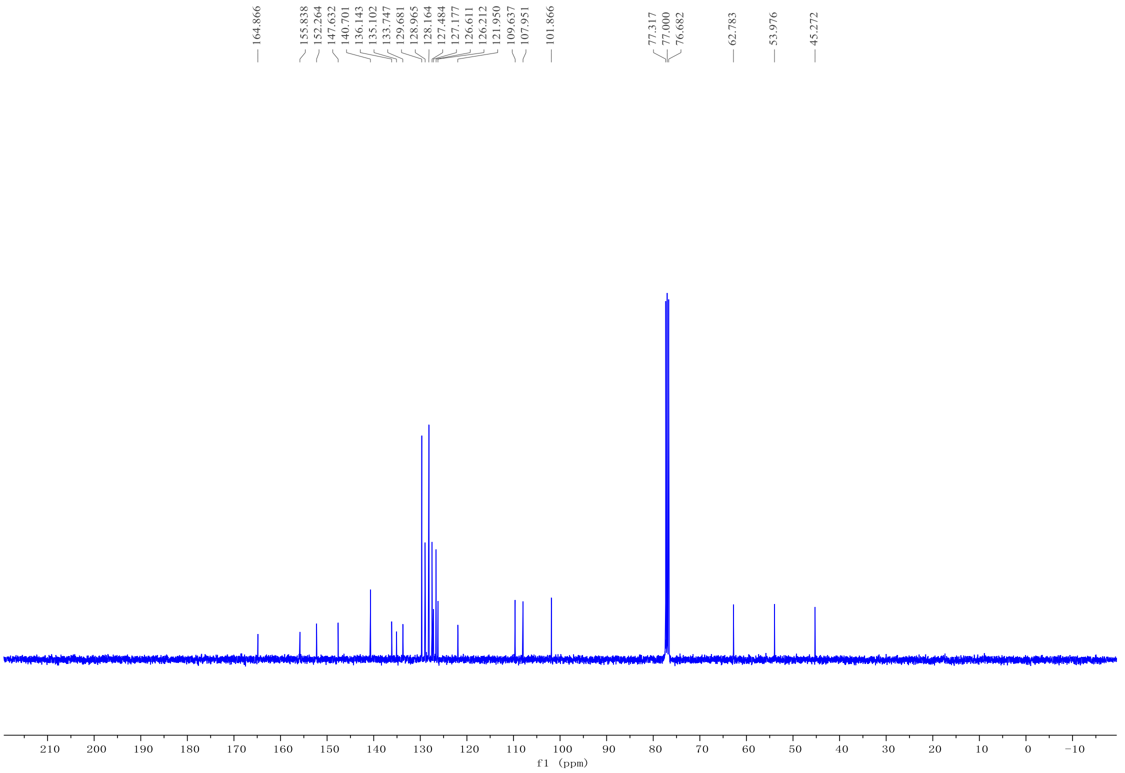

## $^{1}$H and $^{13}$C NMR Spectra for Compound (*Z*)-3ax:

$^{1}$H NMR (400 MHz, $CDCl_3$)

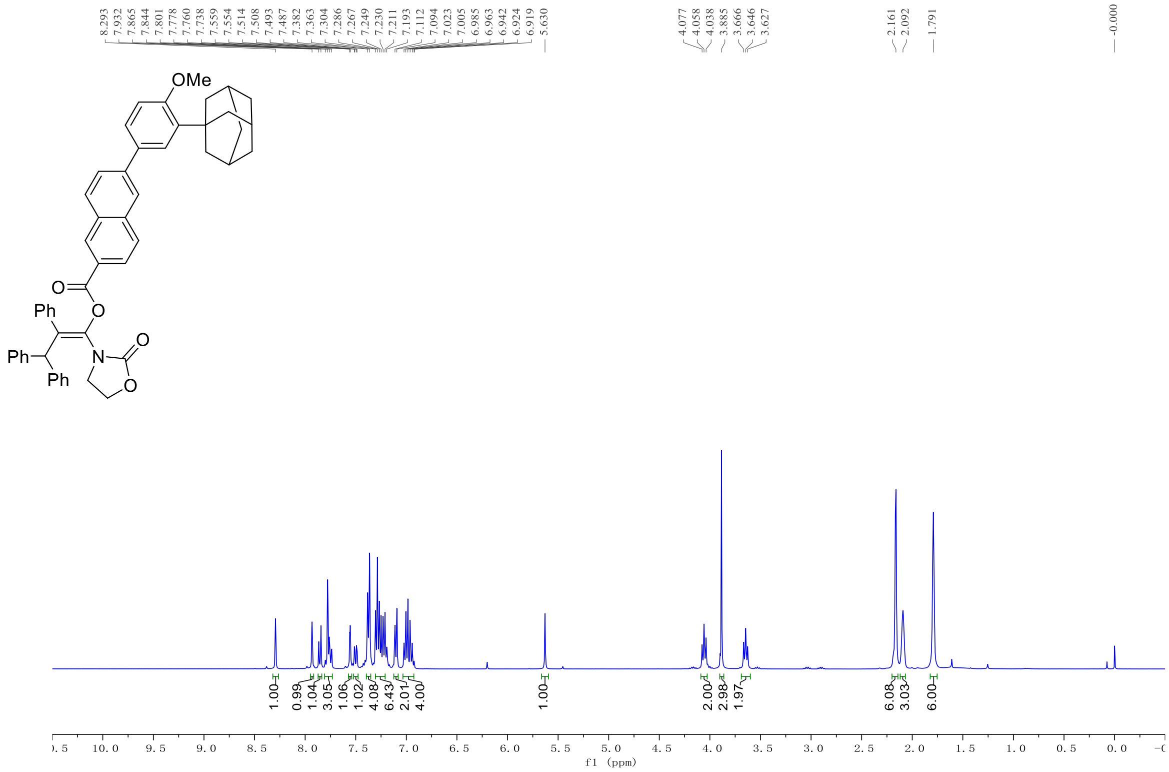

$^{13}$C NMR (100 MHz, $CDCl_3$)

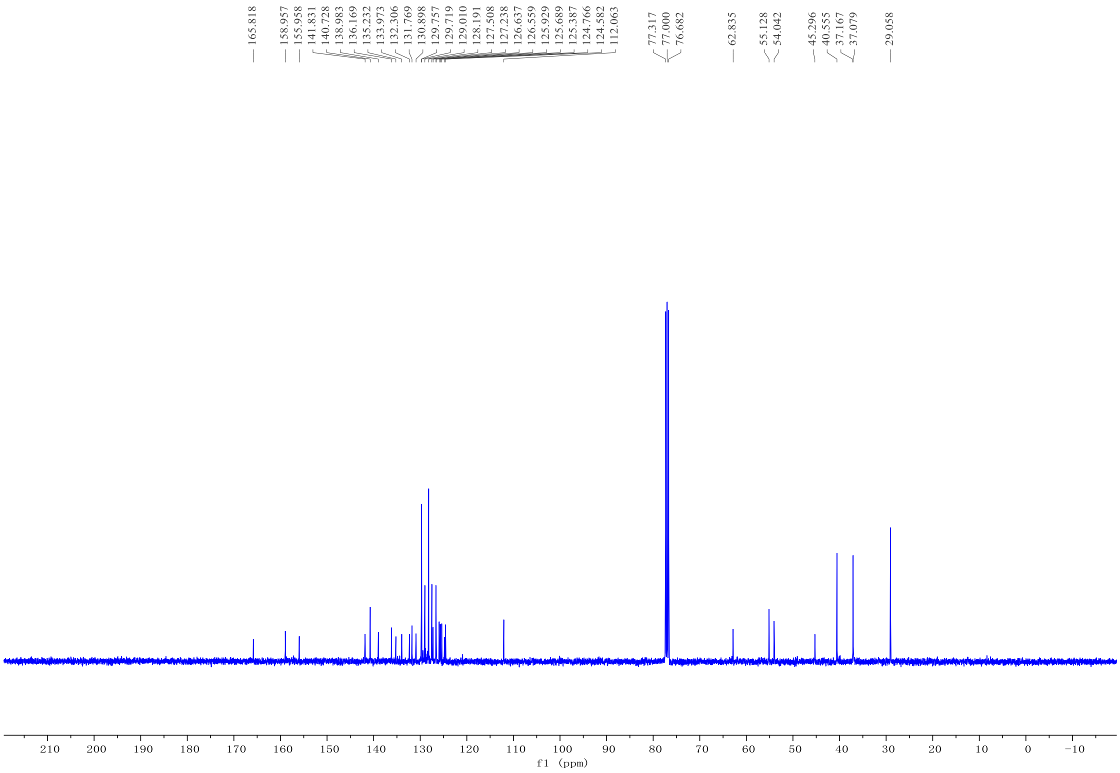

## $^{1}H$ and $^{13}C$ NMR Spectra for Compound (*Z*)-3ay:

### $^{1}H$ NMR (400 MHz, $CDCl_3$)

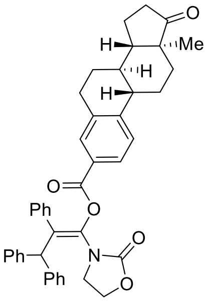


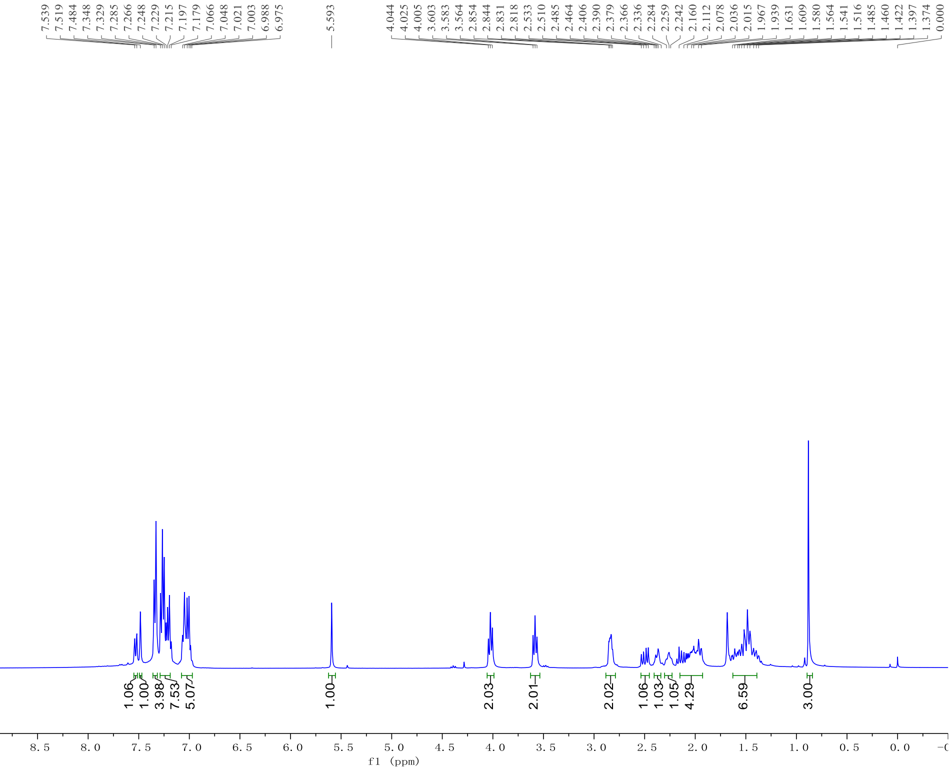


### $^{13}C$ NMR (100 MHz, $CDCl_3$)

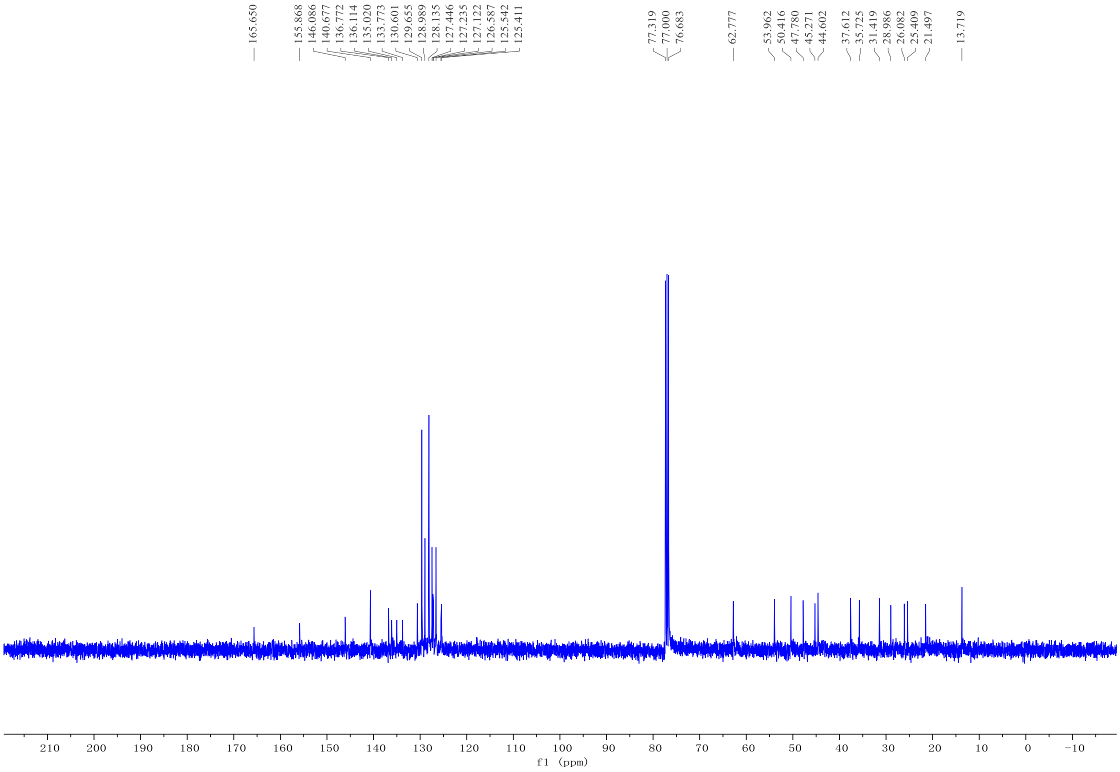

## $^{1}$H and $^{13}$C NMR Spectra for Compound (*Z*)-3az:

$^{1}$H NMR (400 MHz, $CDCl_3$)

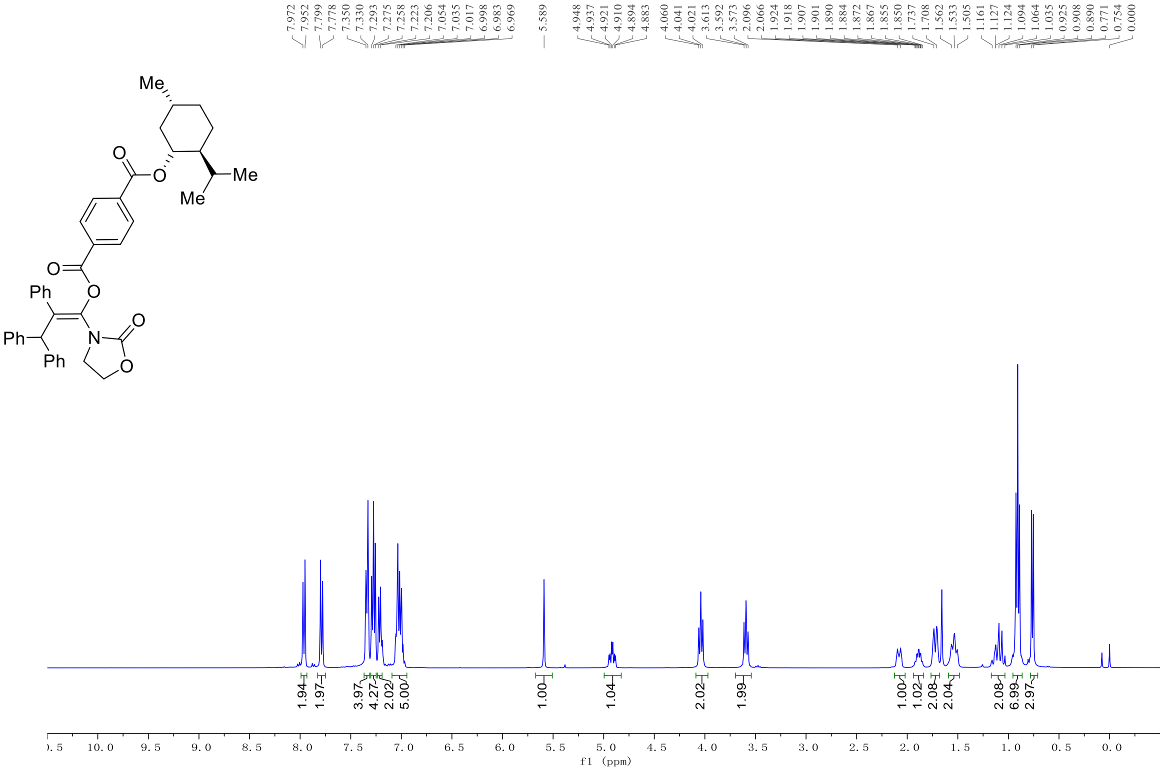

$^{13}$C NMR (100 MHz, $CDCl_3$)

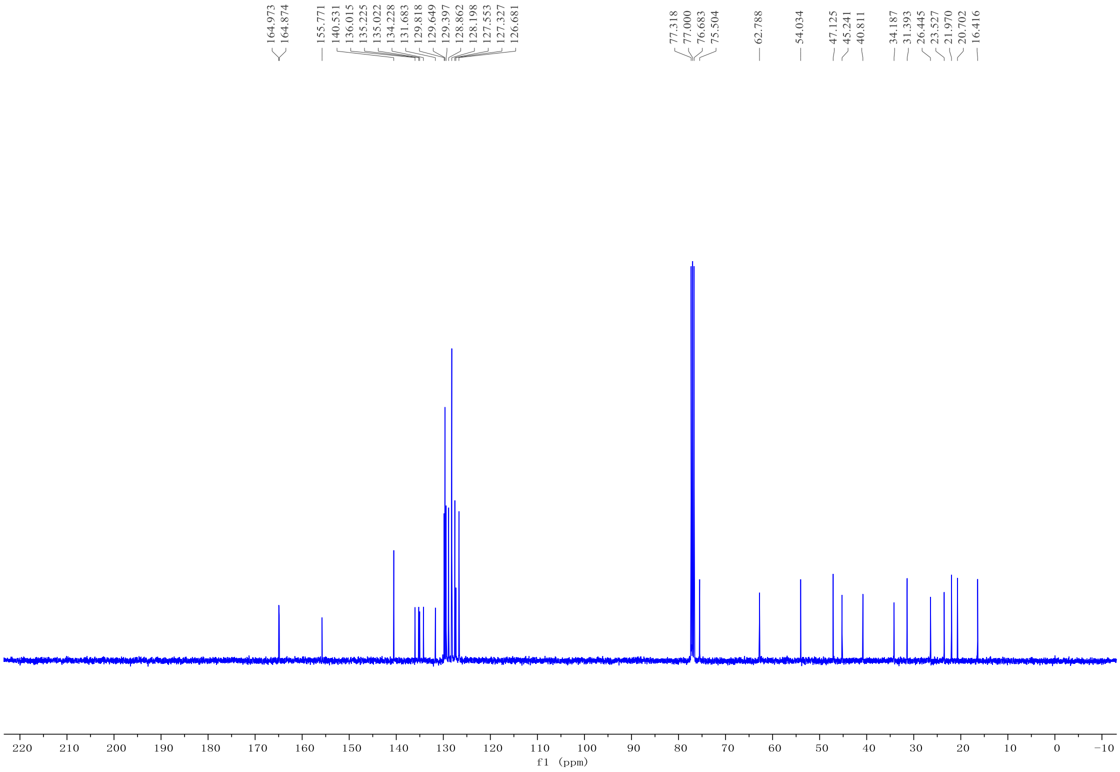

## $^{1}$H and $^{13}$C NMR Spectra for Compound (*Z*)-3aaa:

$^{1}$H NMR (400 MHz, $CDCl_3$)

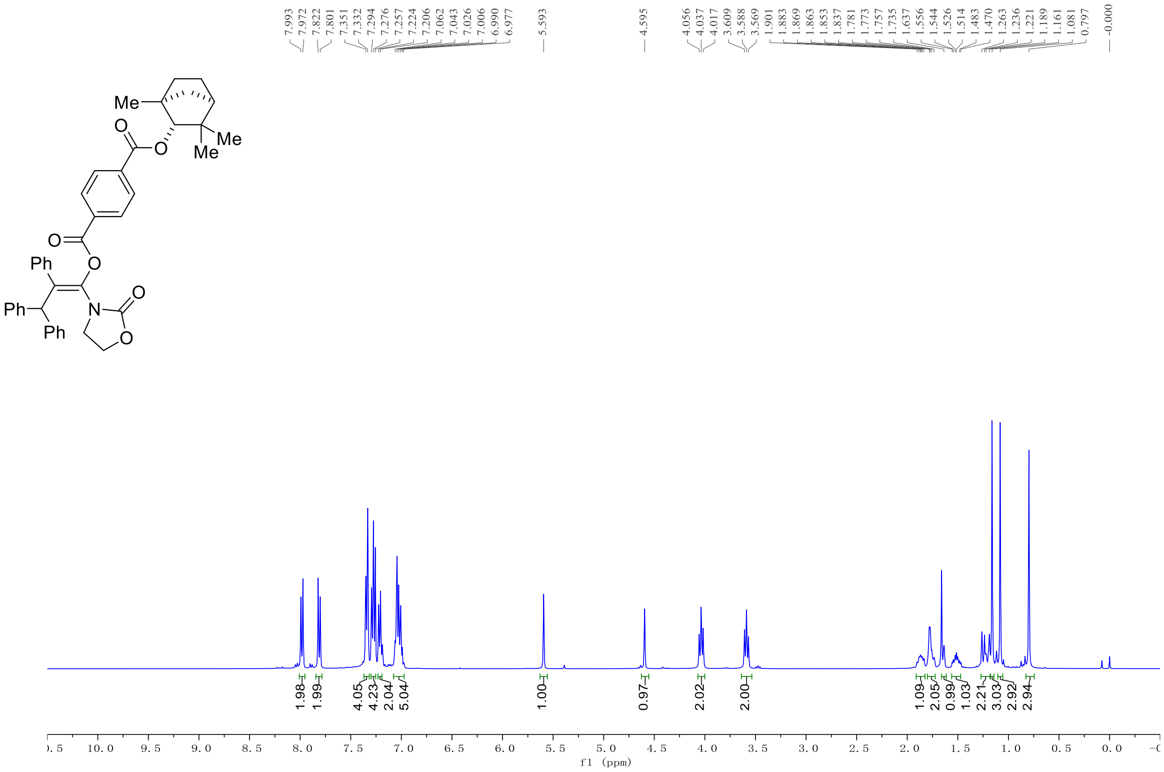


$^{13}$C NMR (100 MHz, $CDCl_3$)

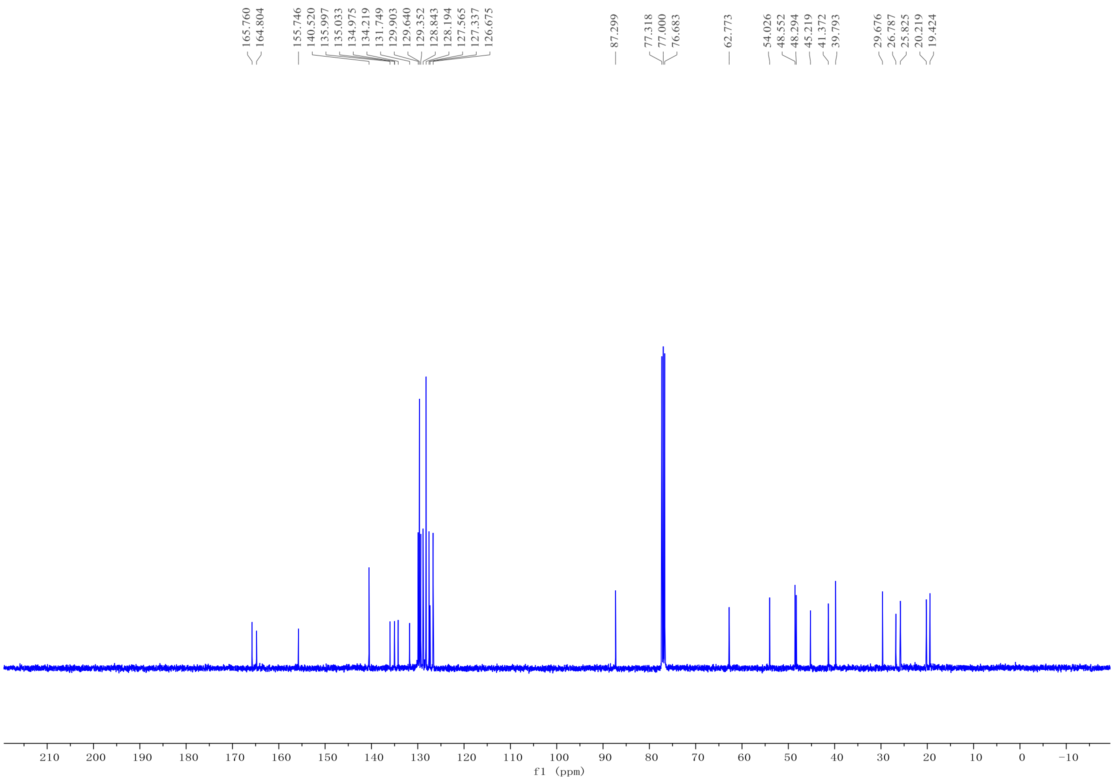

## $^{1}$H and $^{13}$C NMR Spectra for Compound (*Z*)-3aab:

$^{1}$H NMR (400 MHz, $CDCl_3$)

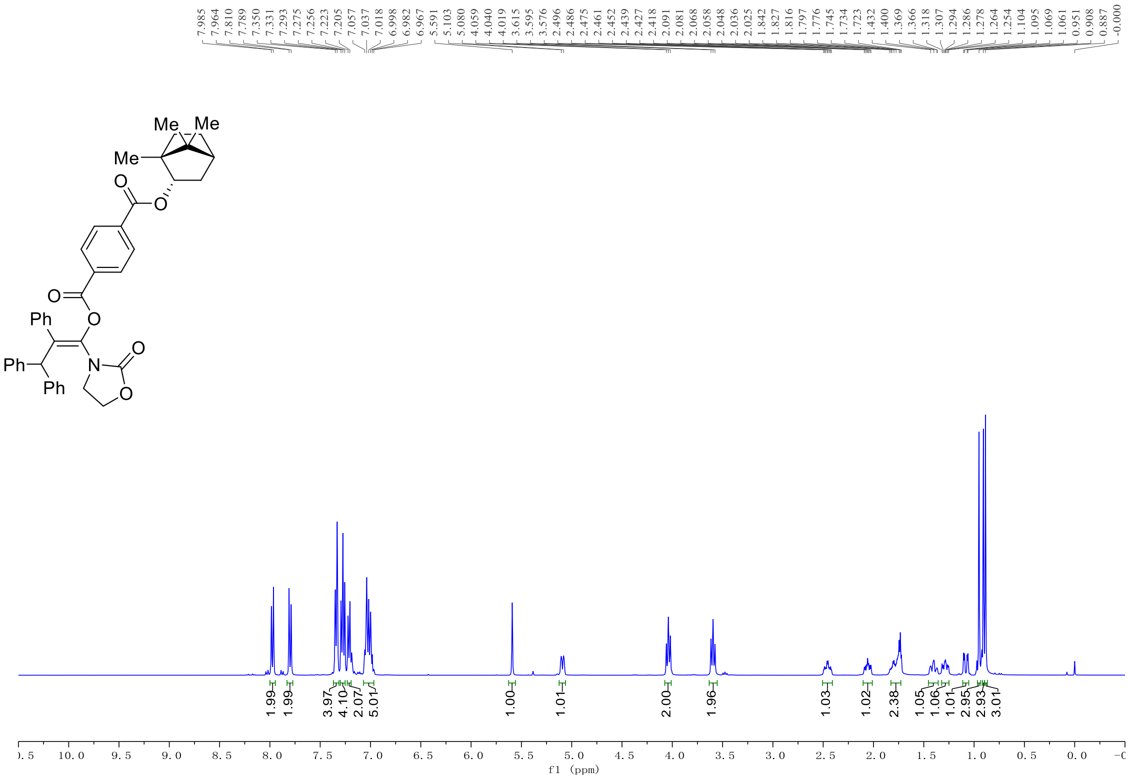


$^{13}$C NMR (100 MHz, $CDCl_3$)

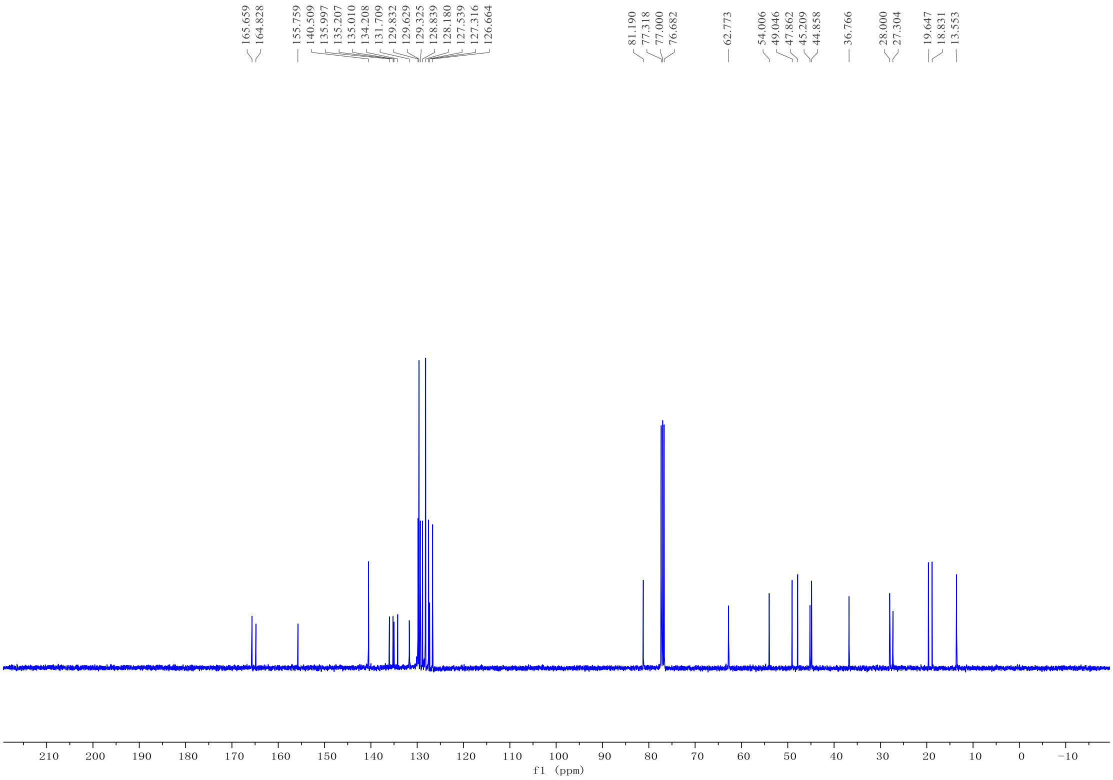

## $^{1}$H, $^{13}$C NMR and $^{19}$F NMR Spectra for Compound (*Z*)-3aac:

$^{1}$H NMR (400 MHz, $CDCl_3$)

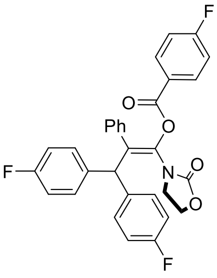


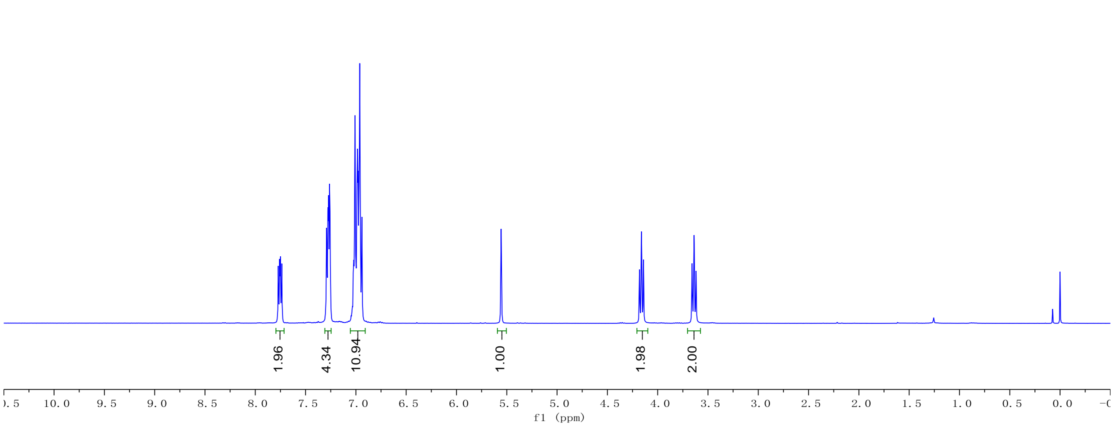


$^{13}$C NMR (100 MHz, $CDCl_3$)

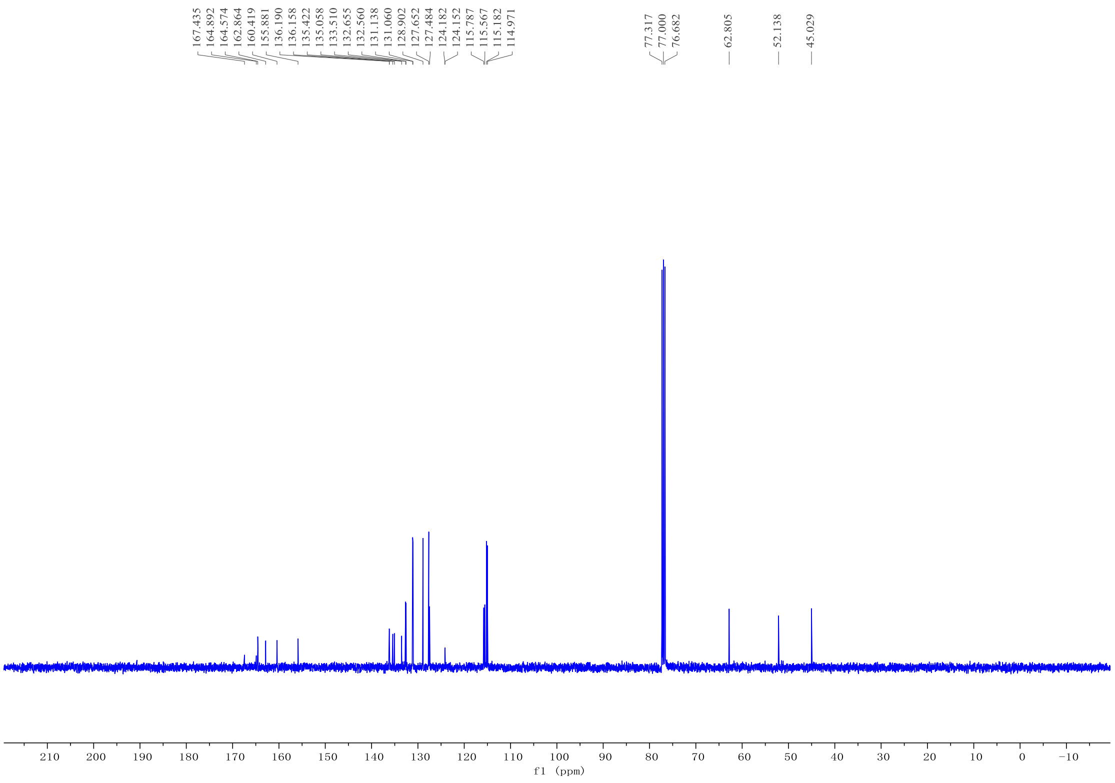

$^{19}$F NMR (376 MHz, $CDCl_3$)

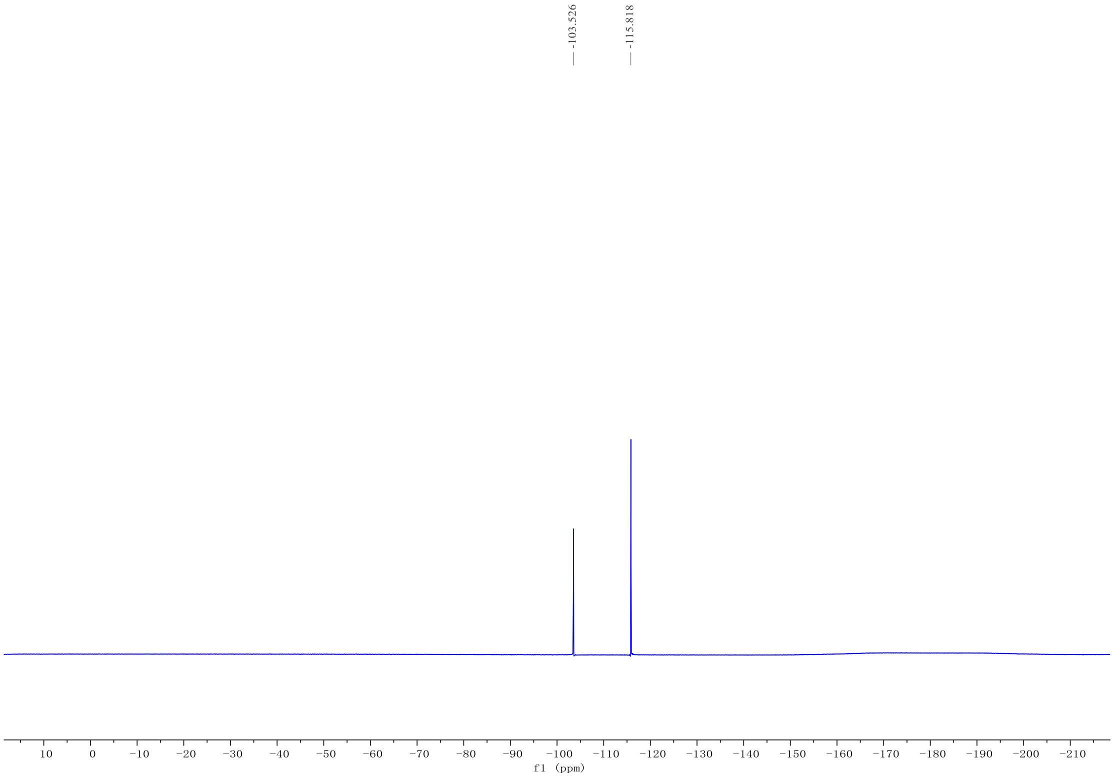


## $^{1}$H, $^{13}$C NMR and $^{19}$F NMR Spectra for Compound (*Z*)-3aad:

$^{1}$H NMR (400 MHz, $CDCl_3$)

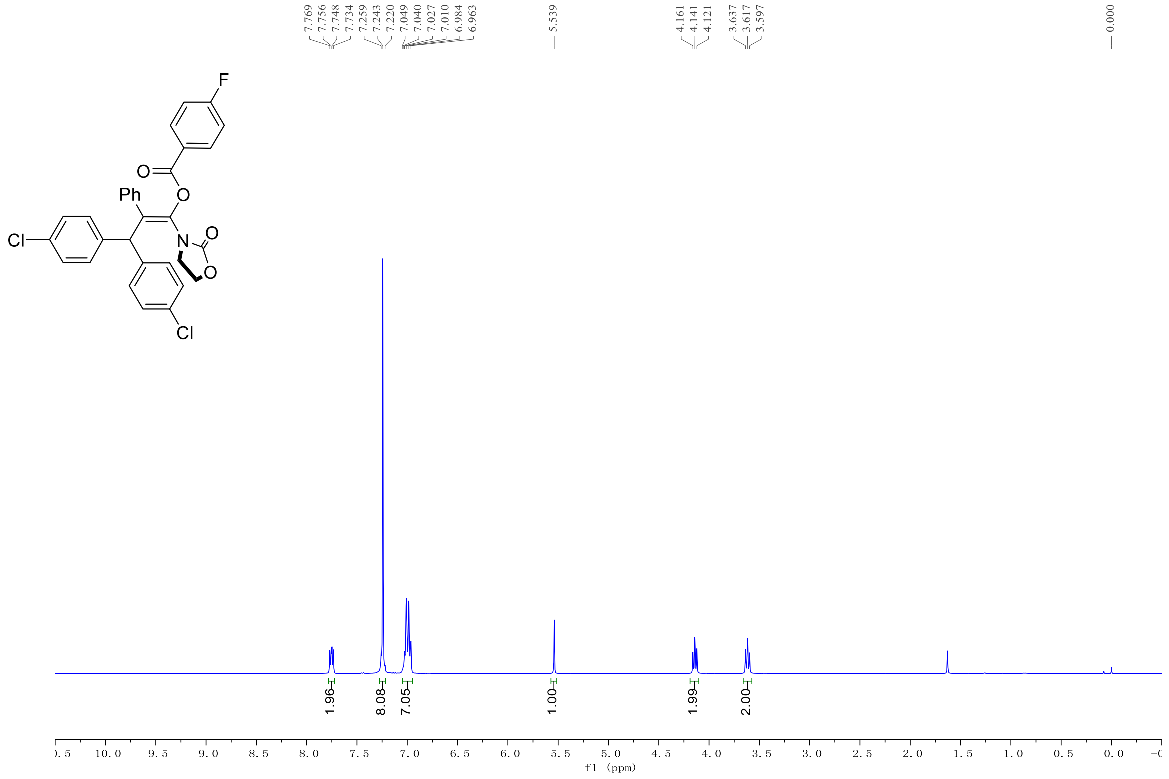

$^{13}C$ NMR (100 MHz, $CDCl_3$)

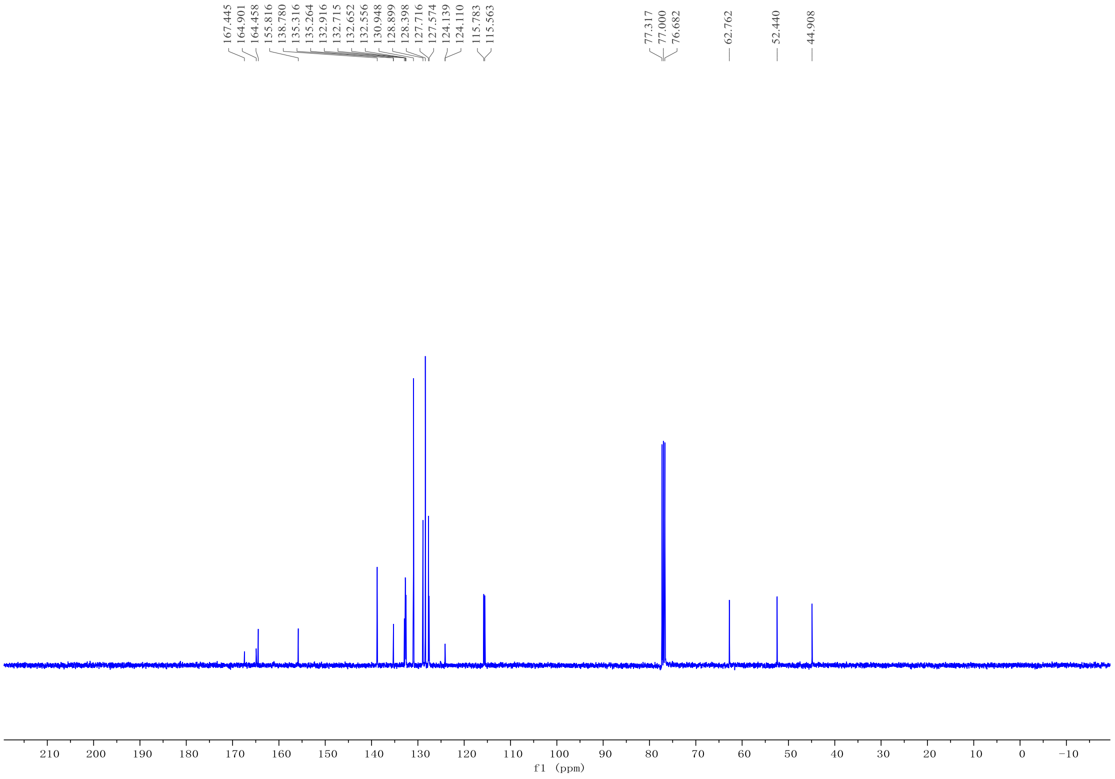


$^{19}F$ NMR (376 MHz, $CDCl_3$)

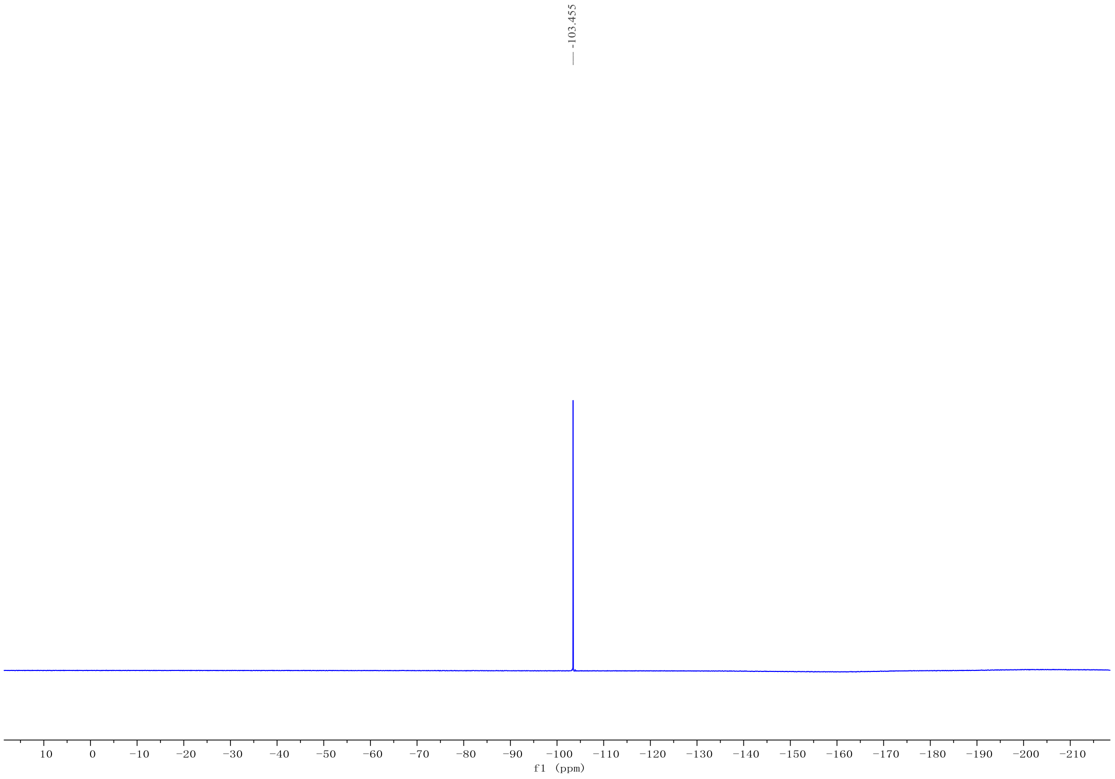

## $^{1}H$, $^{13}C$ NMR and $^{19}F$ NMR Spectra for Compound (*Z*)-3aae:

$^{1}H$ NMR (400 MHz, $CDCl_3$)

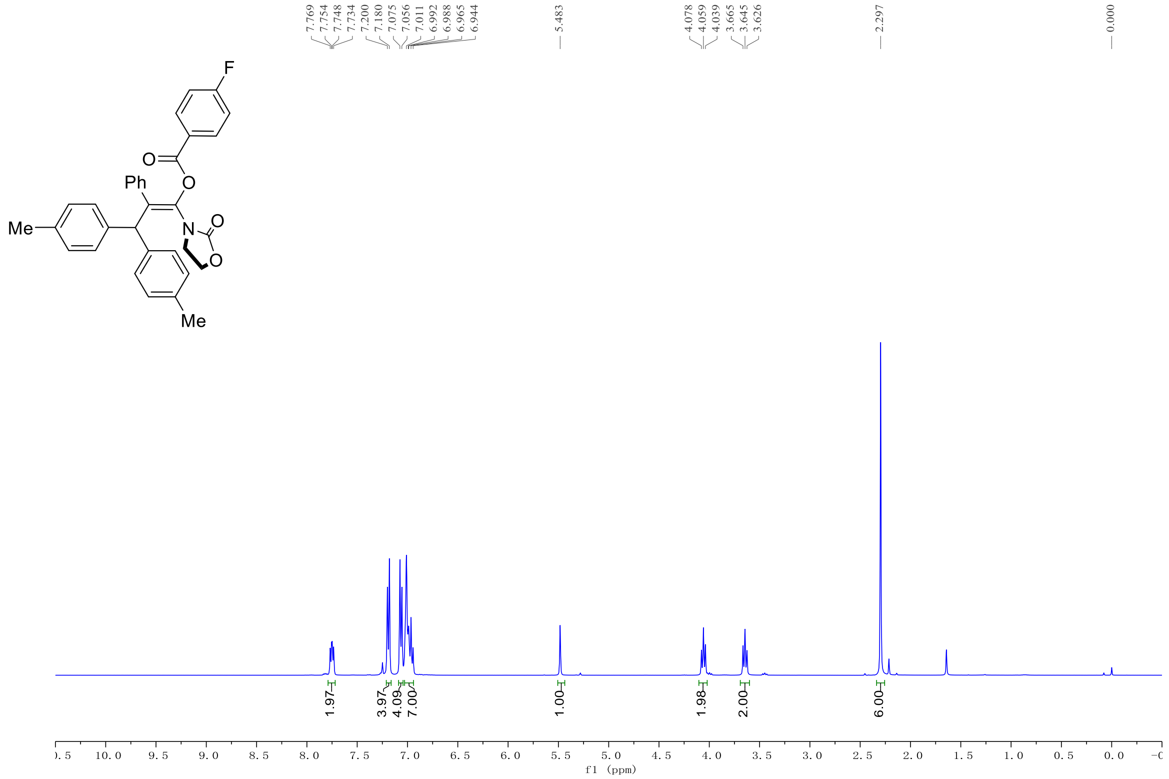


$^{13}C$ NMR (100 MHz, $CDCl_3$)

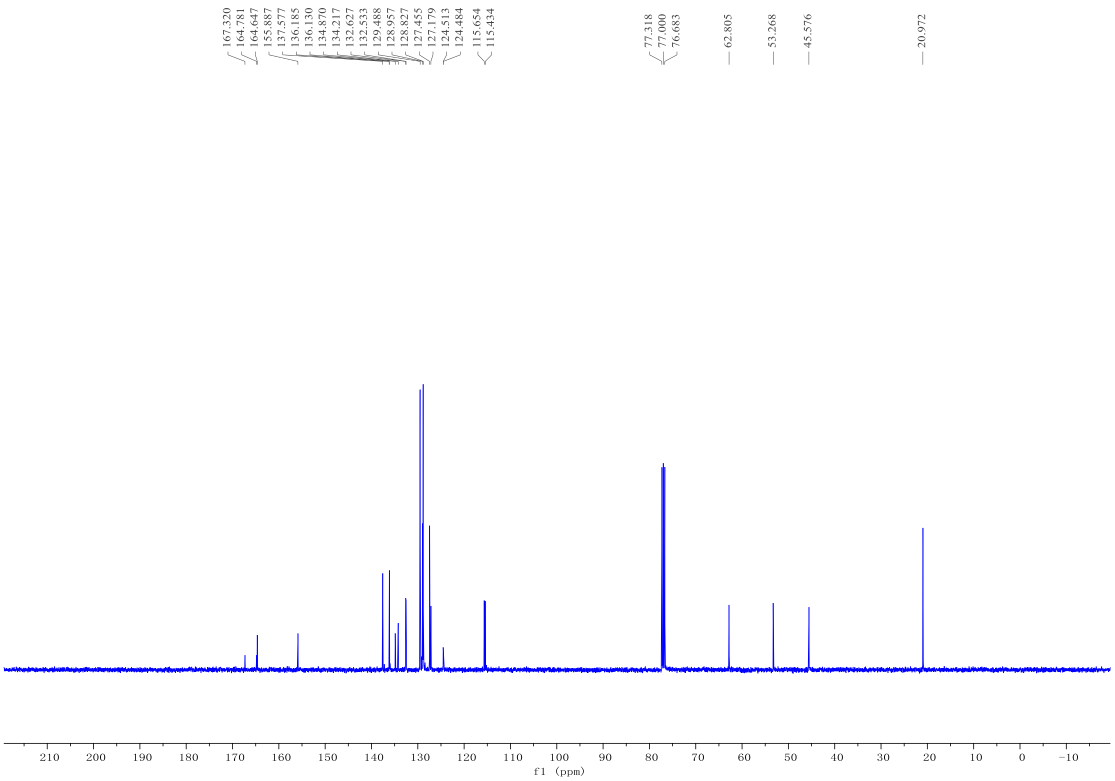

$^{19}F$ NMR (376 MHz, $CDCl_3$)

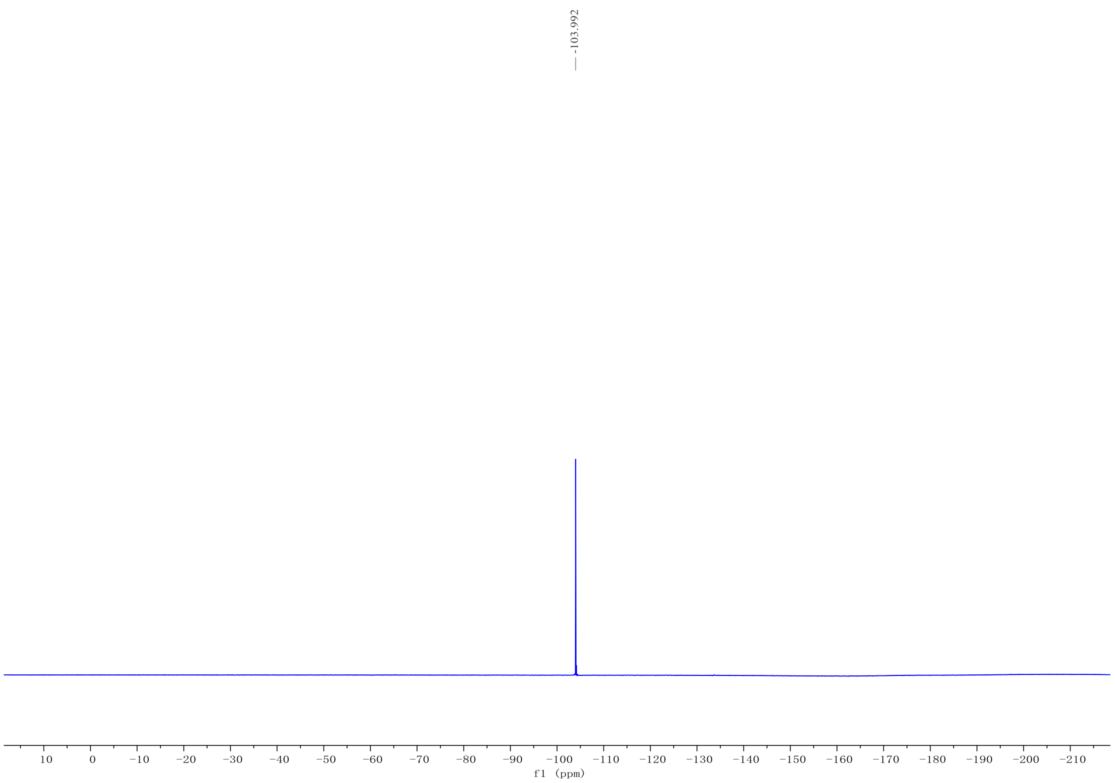

## $^{1}H$, $^{13}C$ NMR and $^{19}F$ NMR Spectra for Compound (*Z*)-3aaf:

$^{1}H$ NMR (400 MHz, $CDCl_3$)

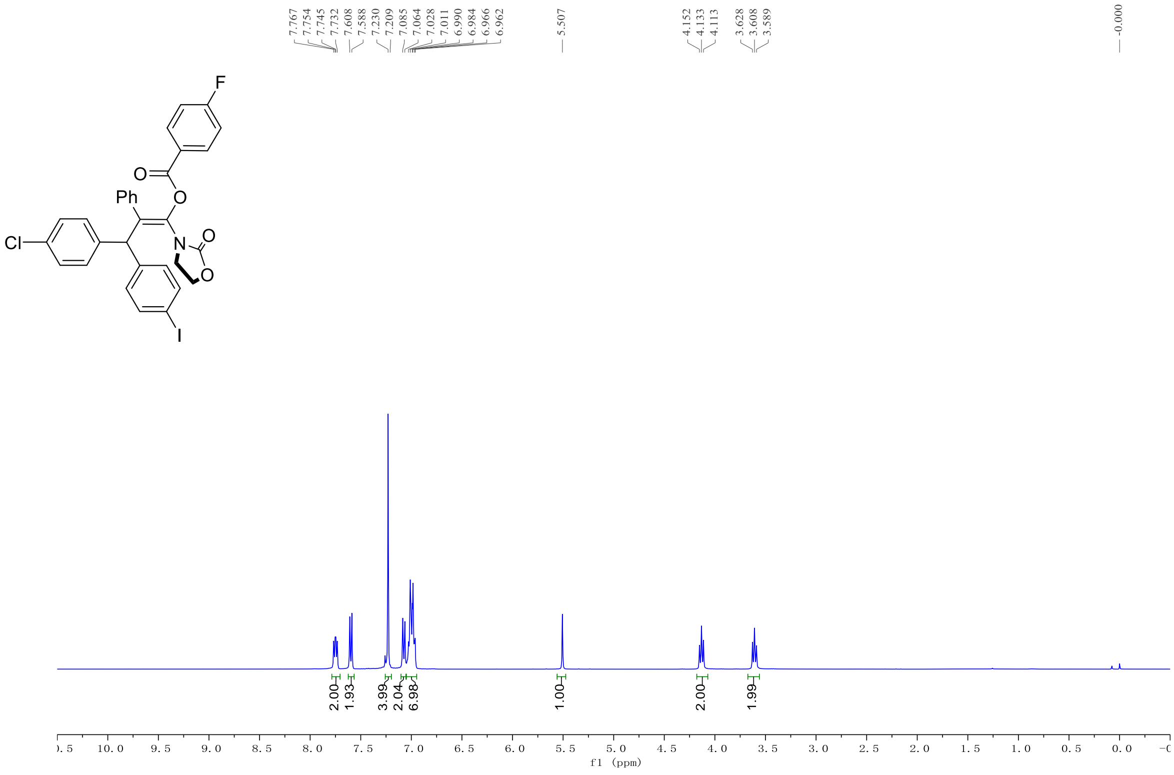

$^{13}C$ NMR (100 MHz, $CDCl_3$)

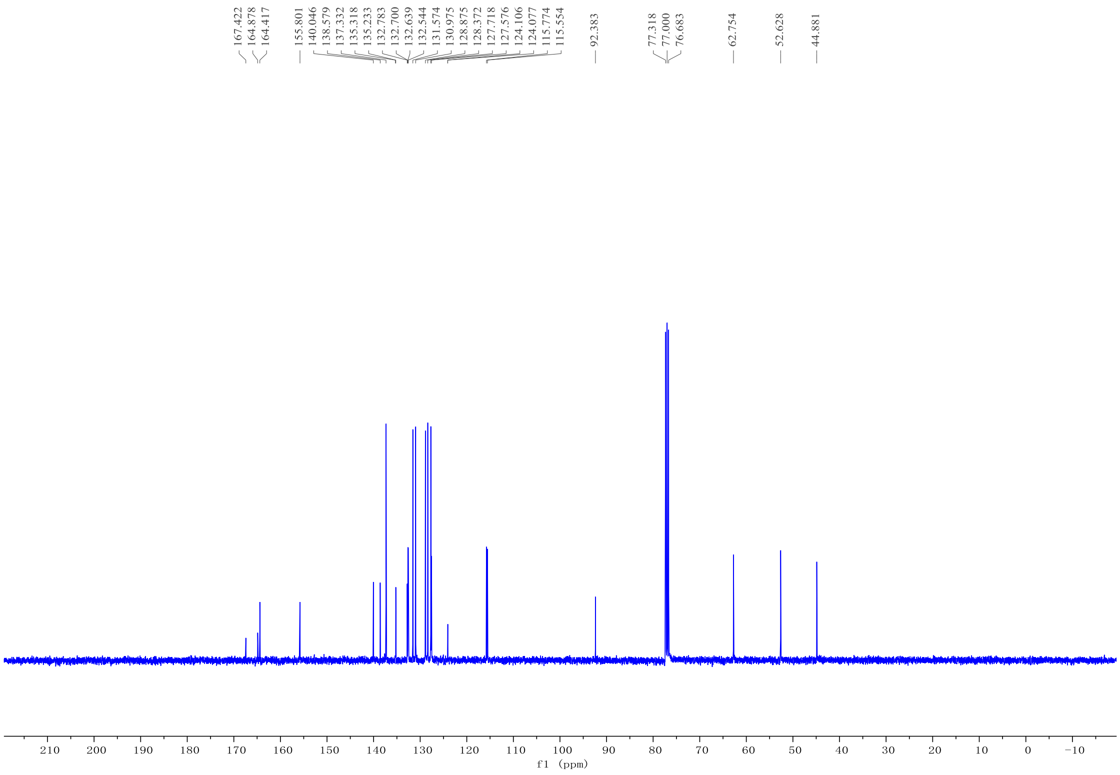


$^{19}F$ NMR (376 MHz, $CDCl_3$)

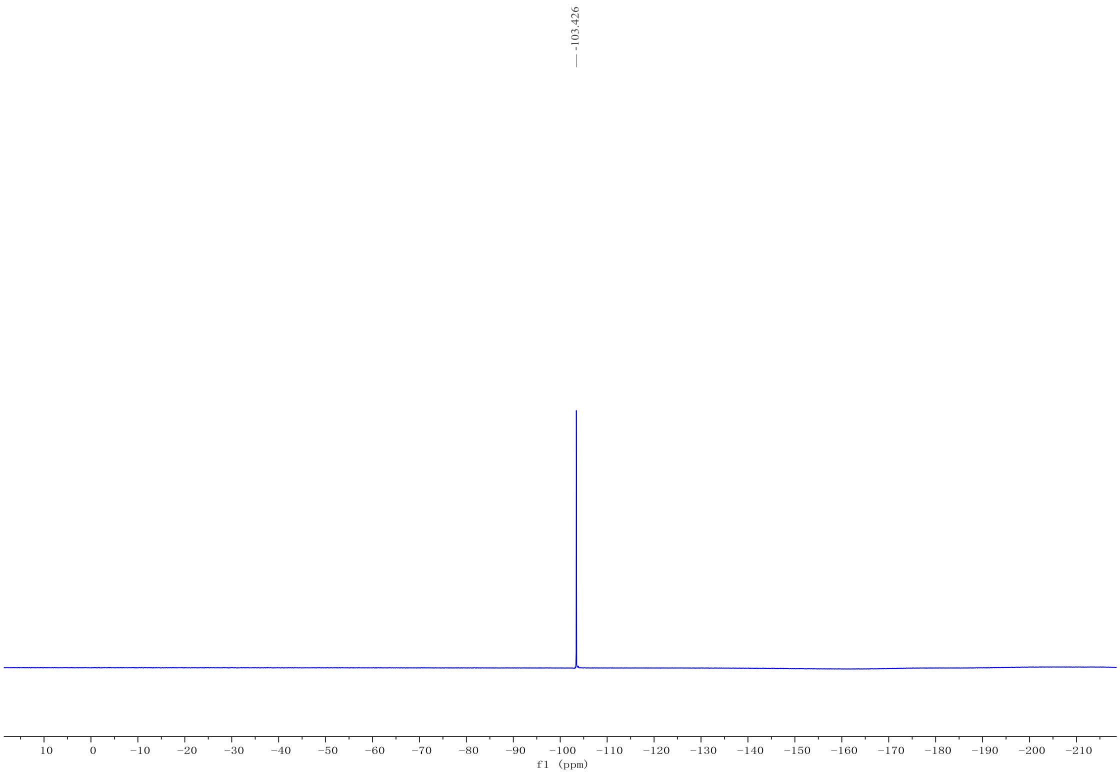

**$^{1}$H, $^{13}$C NMR and $^{19}$F NMR Spectra for Compound (*Z*)-3aag**:

$^{1}$H NMR (400 MHz, $CDCl_3$)

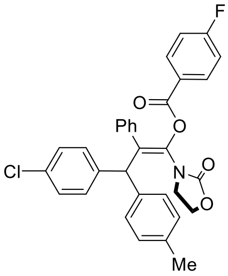


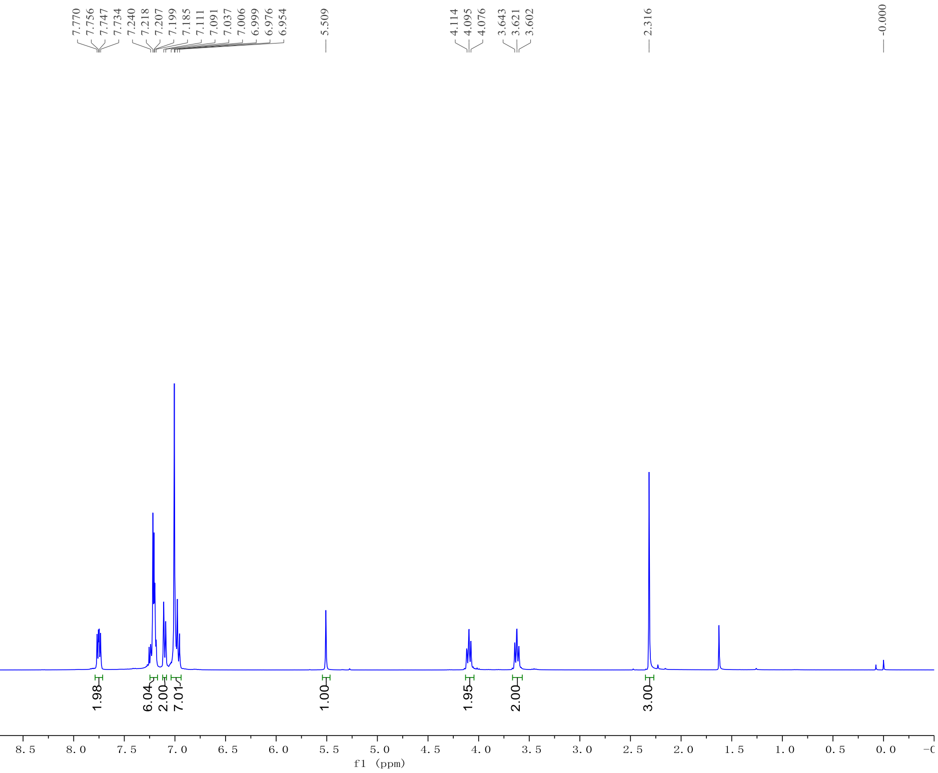

$^{13}$C NMR (100 MHz, $CDCl_3$)

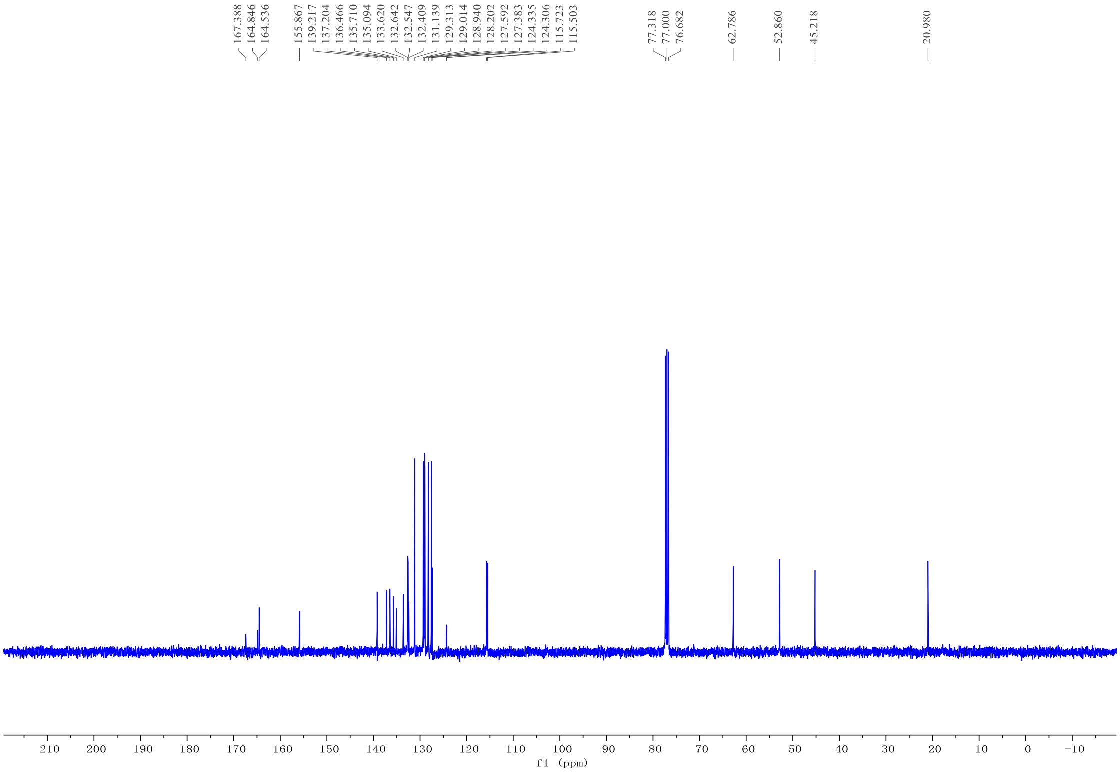

$^{19}$F NMR (376 MHz, CDCl$_3$)

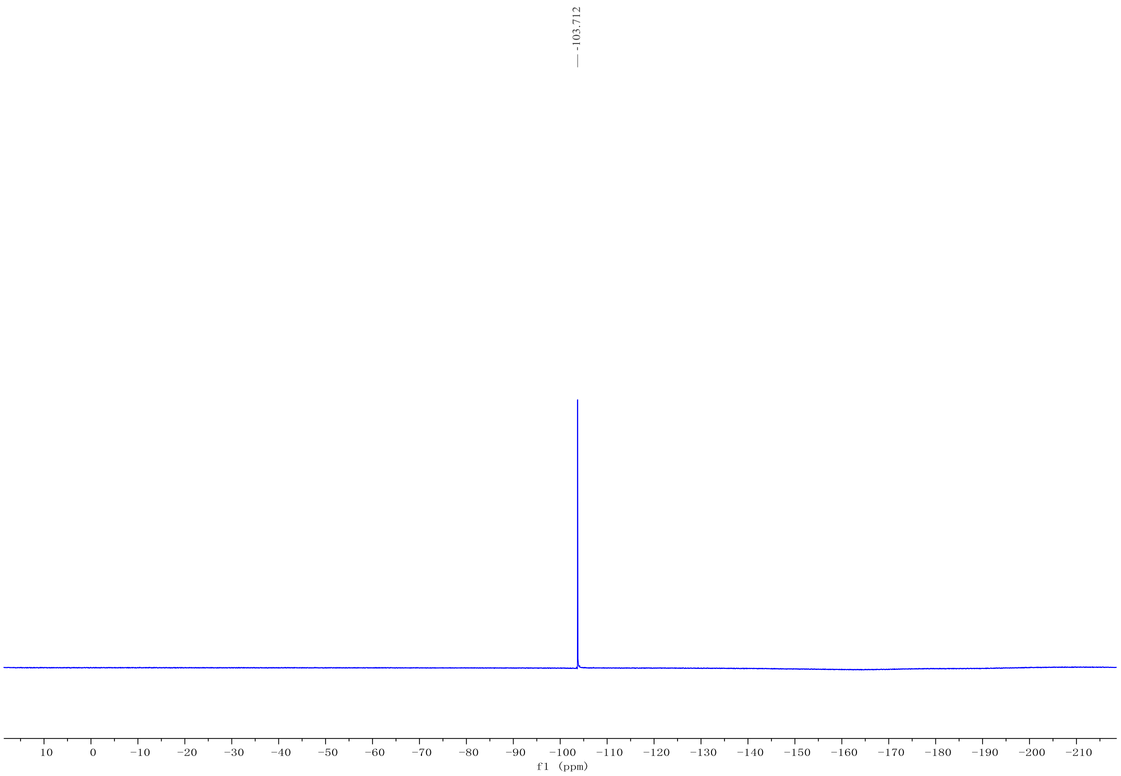


## $^{1}$H, $^{13}$C NMR and $^{19}$F NMR Spectra for Compound (*Z*)-3aah:

$^{1}$H NMR (400 MHz, CDCl$_3$)

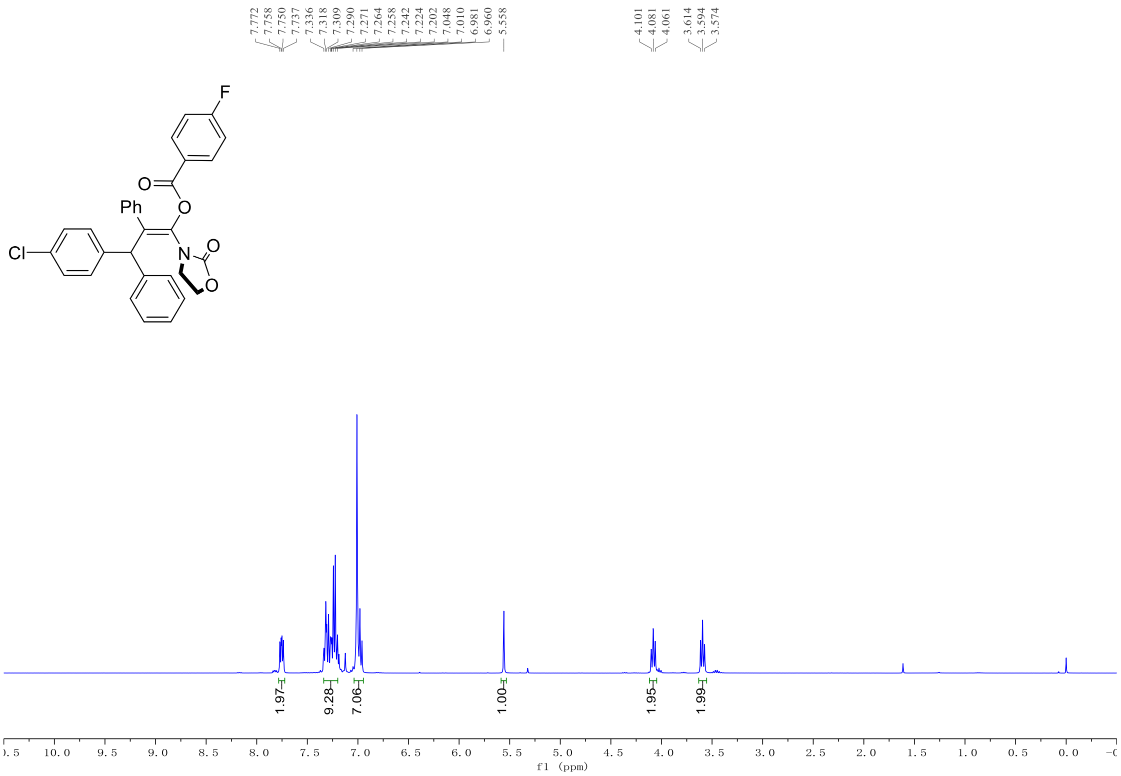

$^{13}C$ NMR (100 MHz, $CDCl_3$)

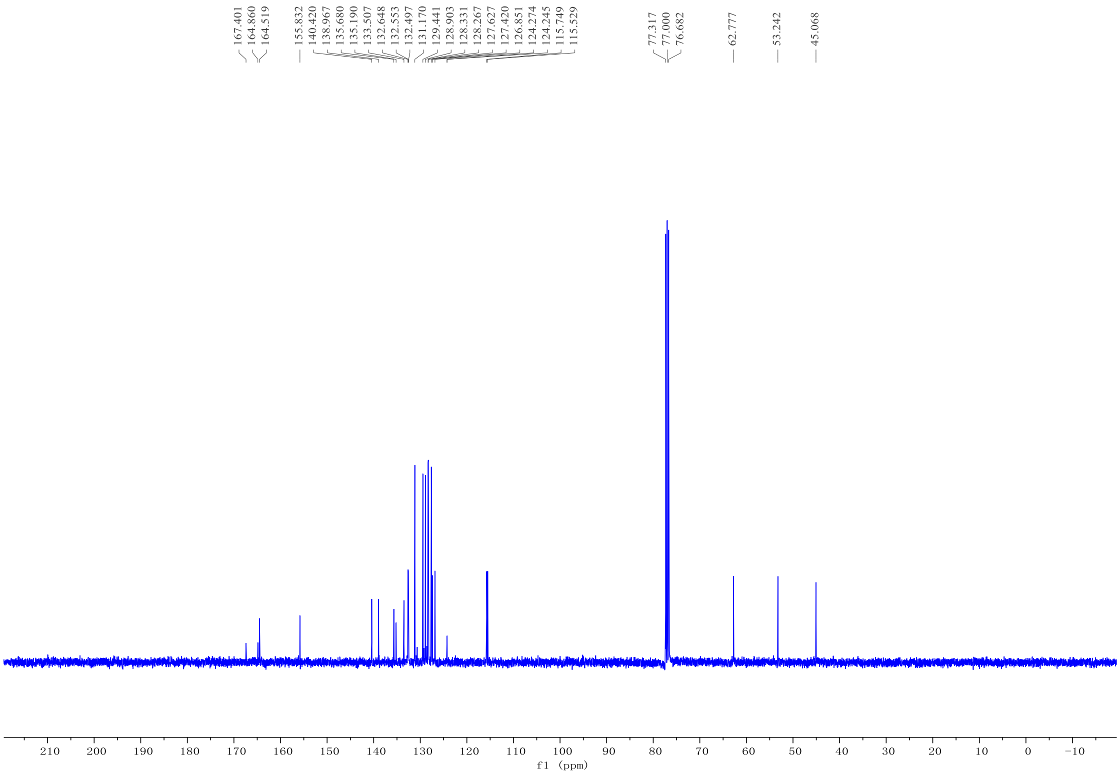


$^{19}F$ NMR (376 MHz, $CDCl_3$)

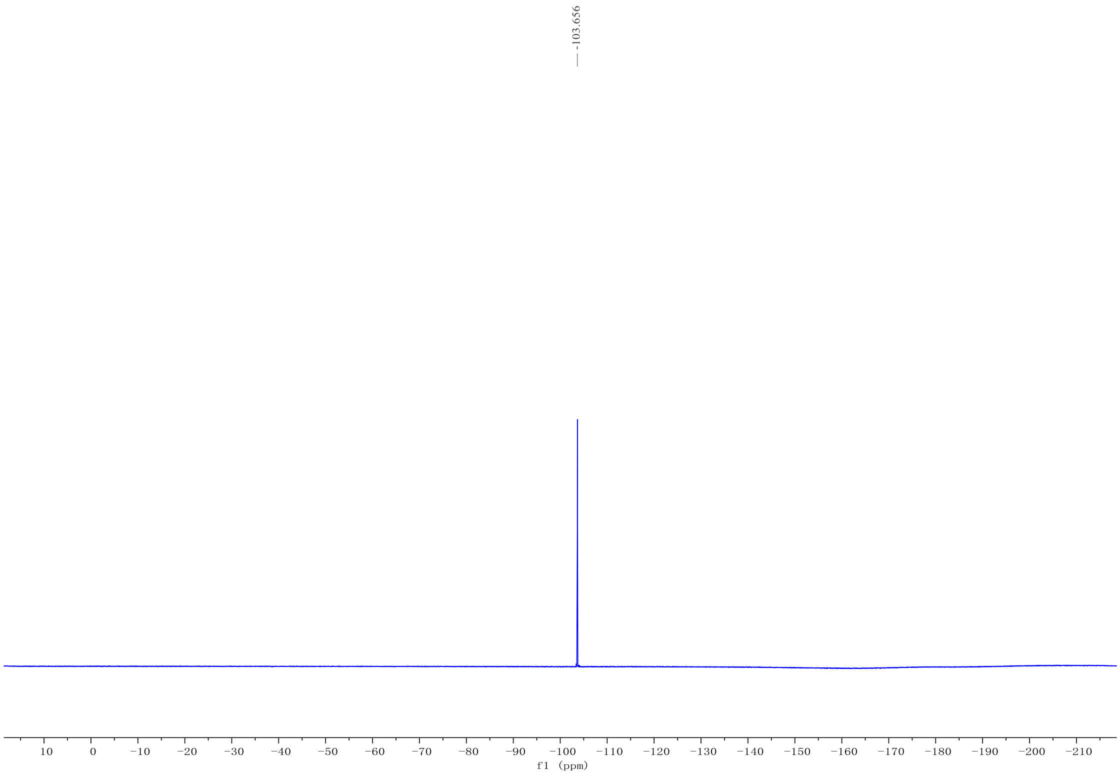

**$^{1}$H, $^{13}$C NMR and $^{19}$F NMR Spectra for Compound (*Z*)-3aai**:

$^{1}$H NMR (400 MHz, $CDCl_3$)

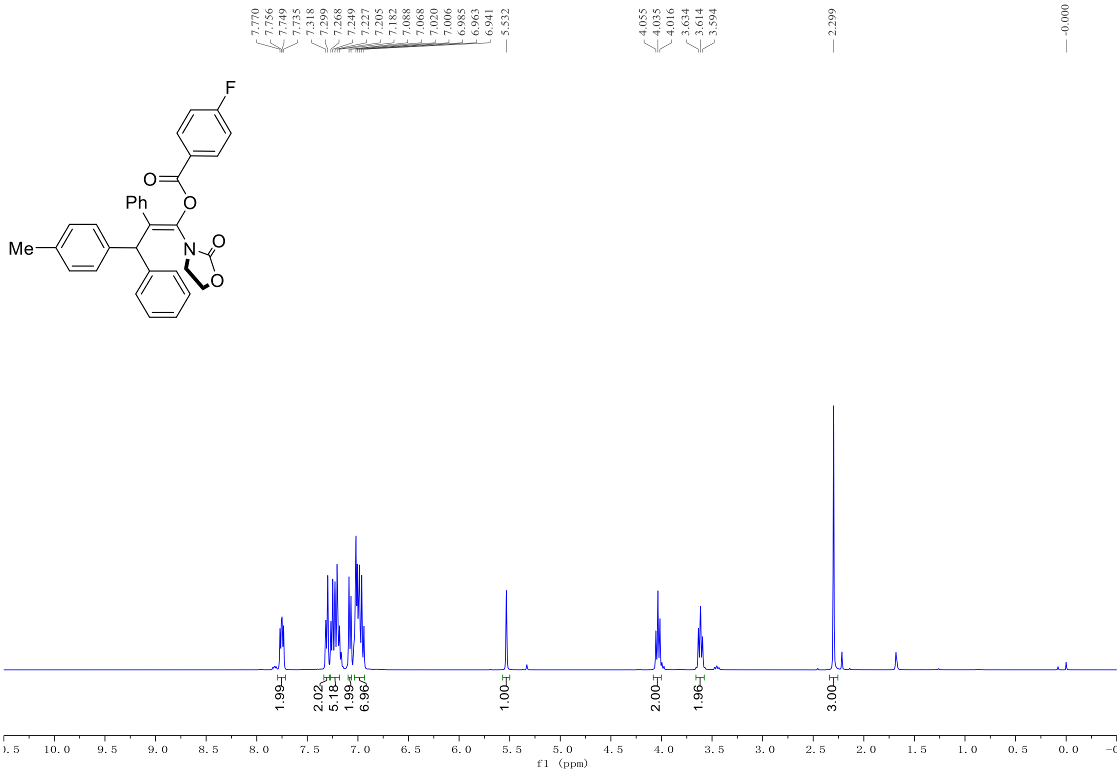


$^{13}$C NMR (100 MHz, $CDCl_3$)

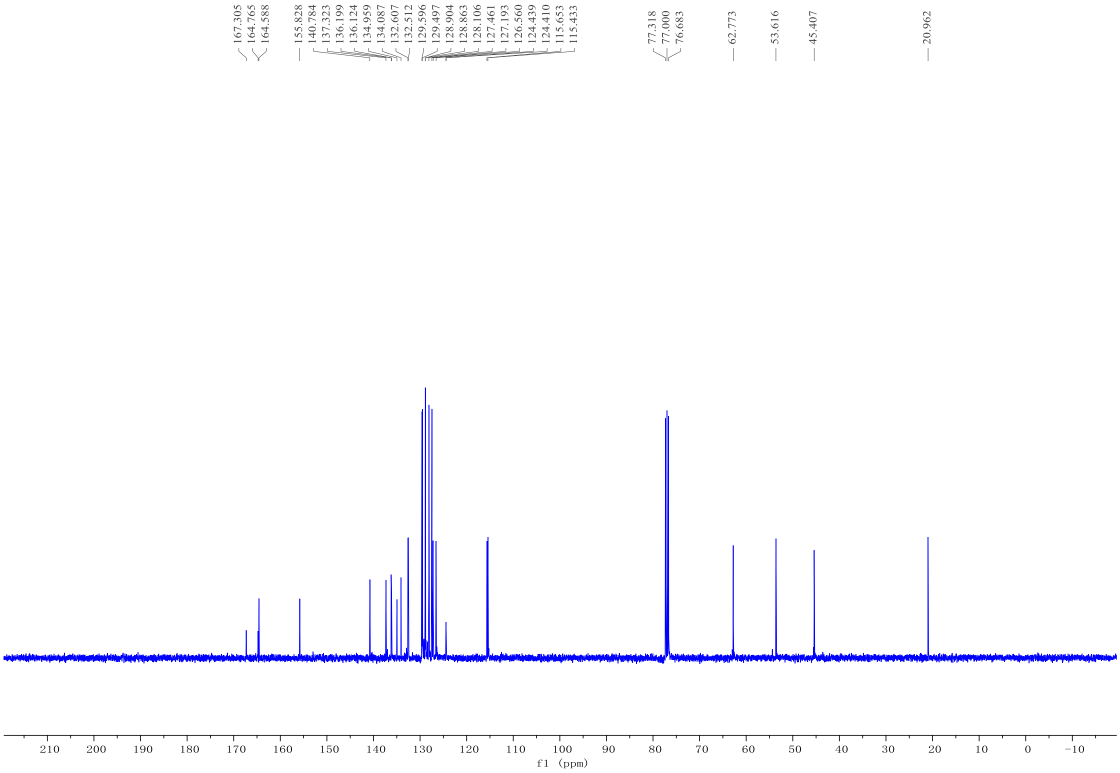

$^{19}F$ NMR (376 MHz, $CDCl_3$)

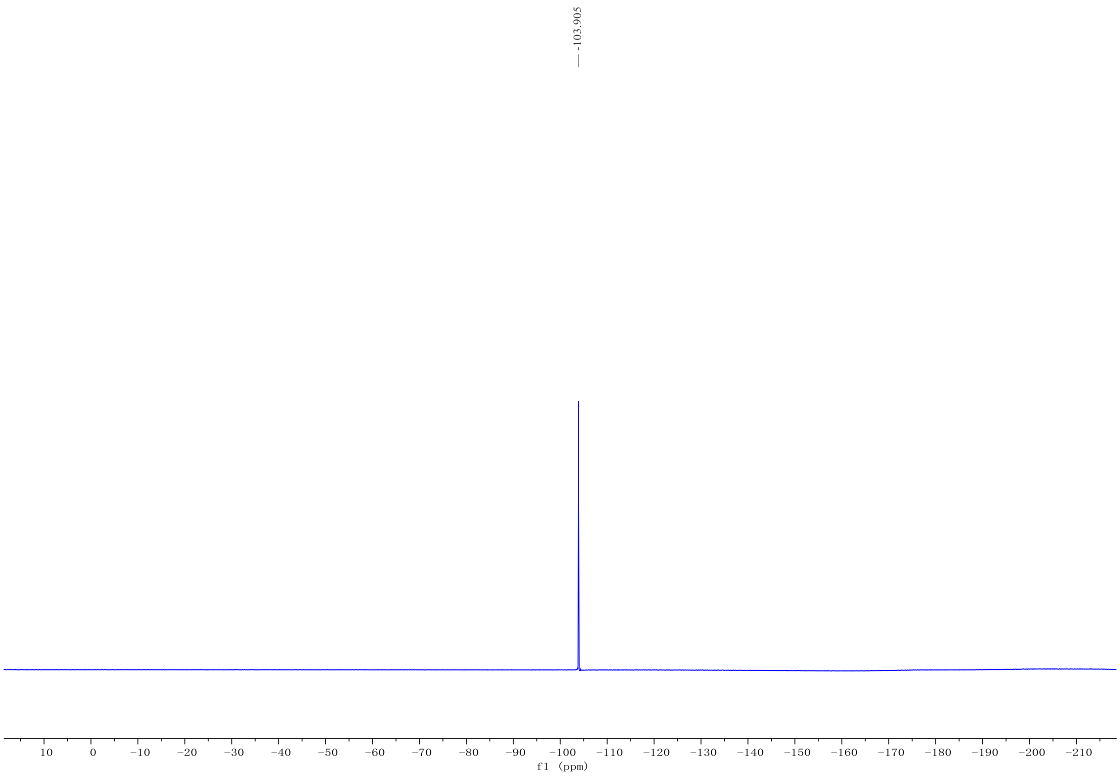


**$^{1}H$, $^{13}C$ NMR and $^{19}F$ NMR Spectra for Compound (*Z*)-3aaj**:

$^{1}H$ NMR (400 MHz, $CDCl_3$)

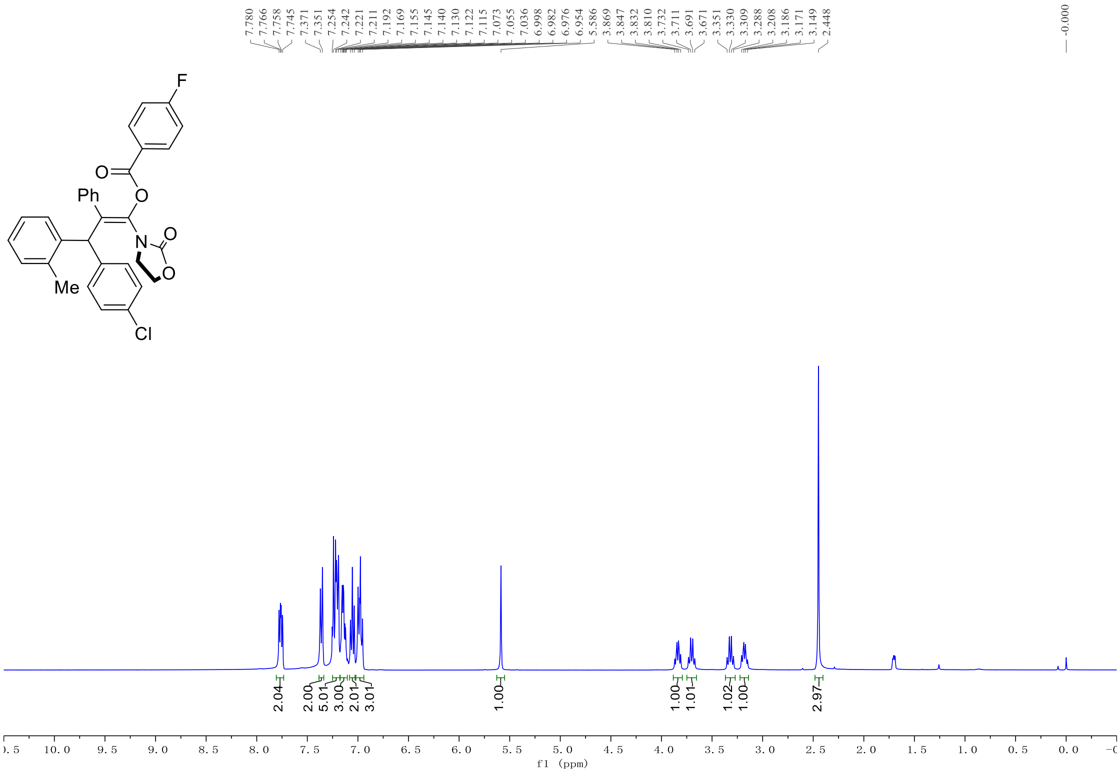

$^{13}C$ NMR (100 MHz, $CDCl_3$)

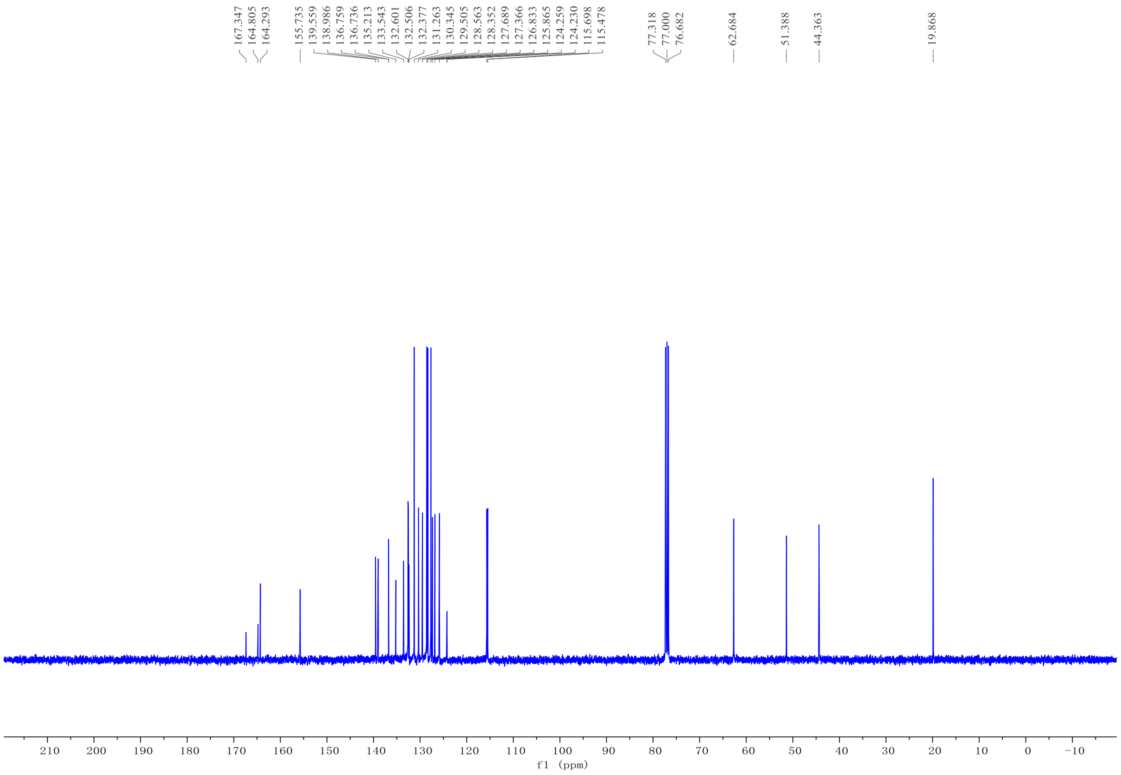


$^{19}F$ NMR (376 MHz, $CDCl_3$)

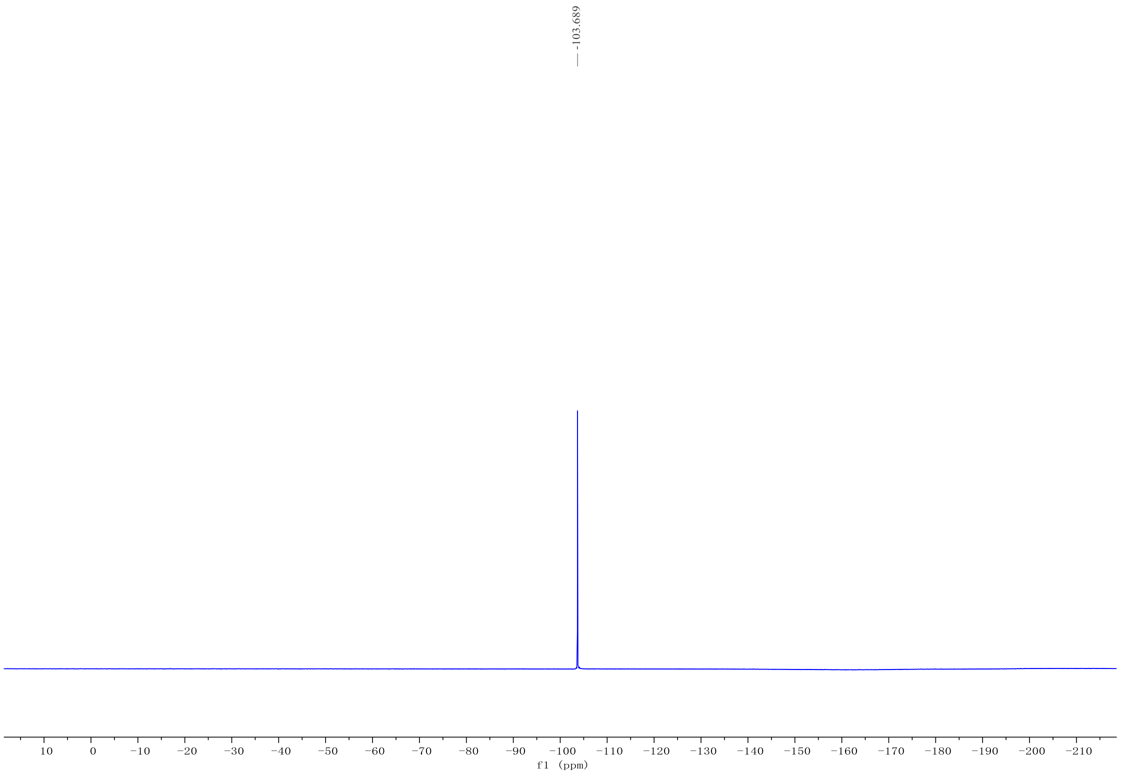

**$^{1}$H, $^{13}$C NMR and $^{19}$F NMR Spectra for Compound (*Z*)-3aak**:

$^{1}$H NMR (400 MHz, $CDCl_3$)

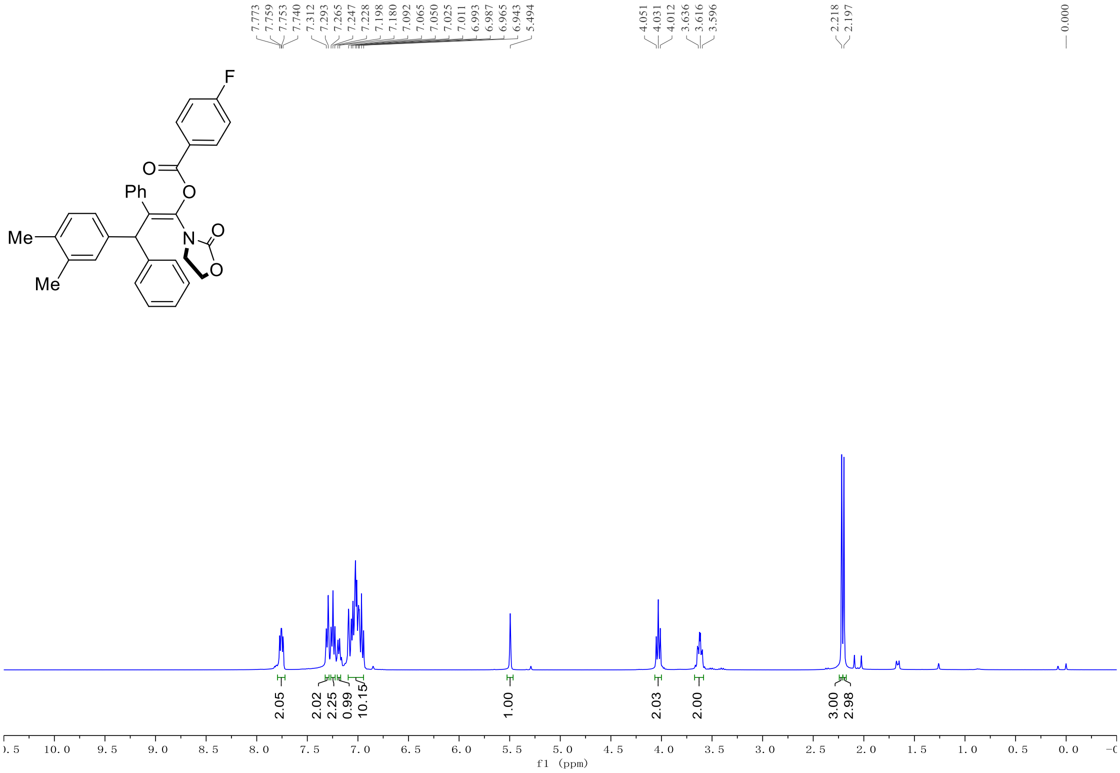


$^{13}$C NMR (100 MHz, $CDCl_3$)

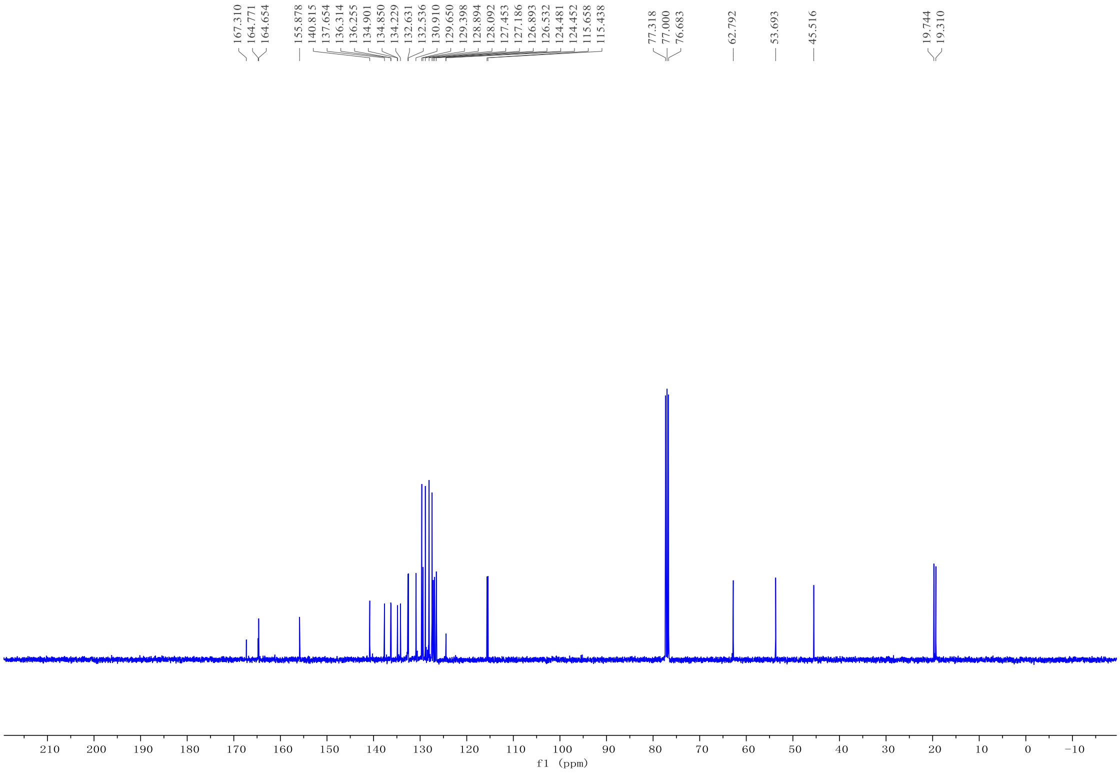

$^{19}F$ NMR (376 MHz, $CDCl_3$)

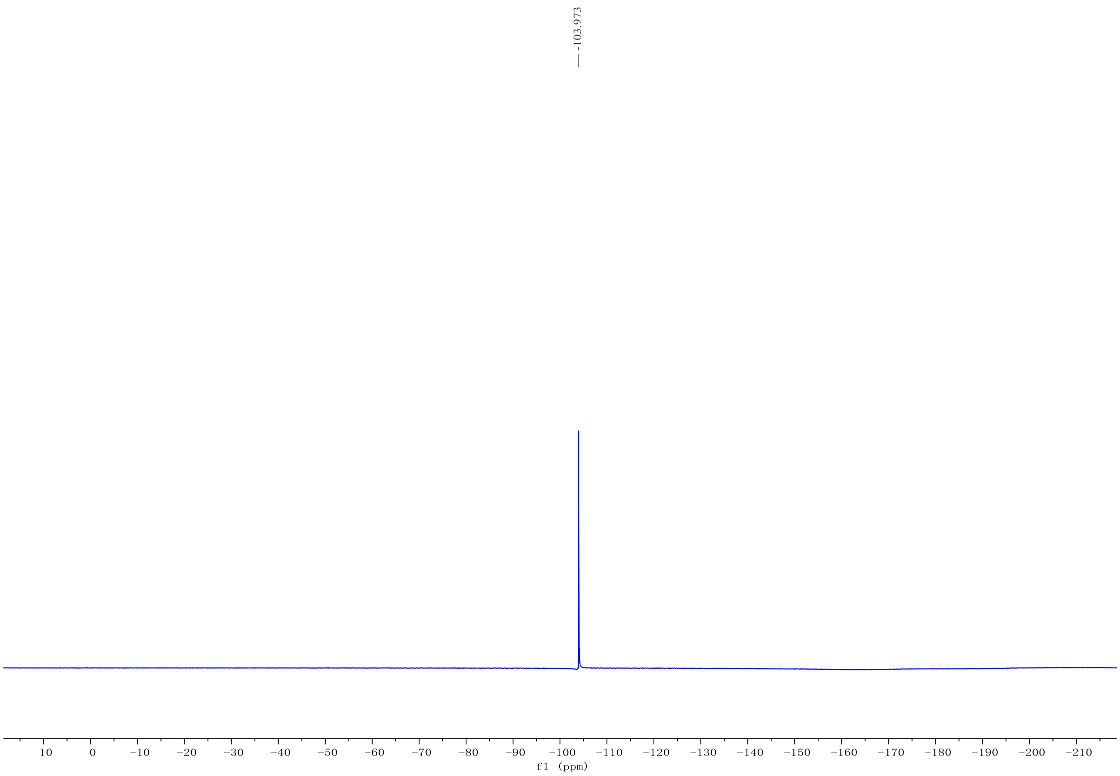


## $^{1}H$, $^{13}C$ NMR and $^{19}F$ NMR Spectra for Compound (*Z*)-3aal:

$^{1}H$ NMR (400 MHz, $CDCl_3$)

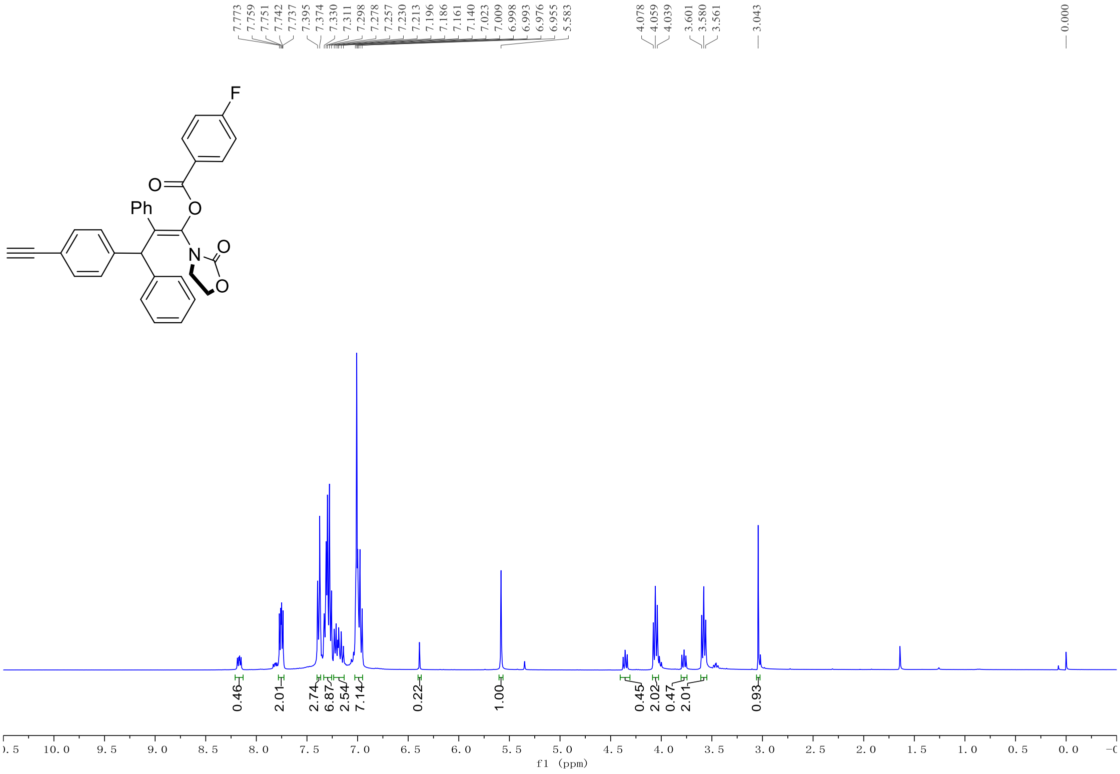

$^{13}C$ NMR (100 MHz, $CDCl_3$)

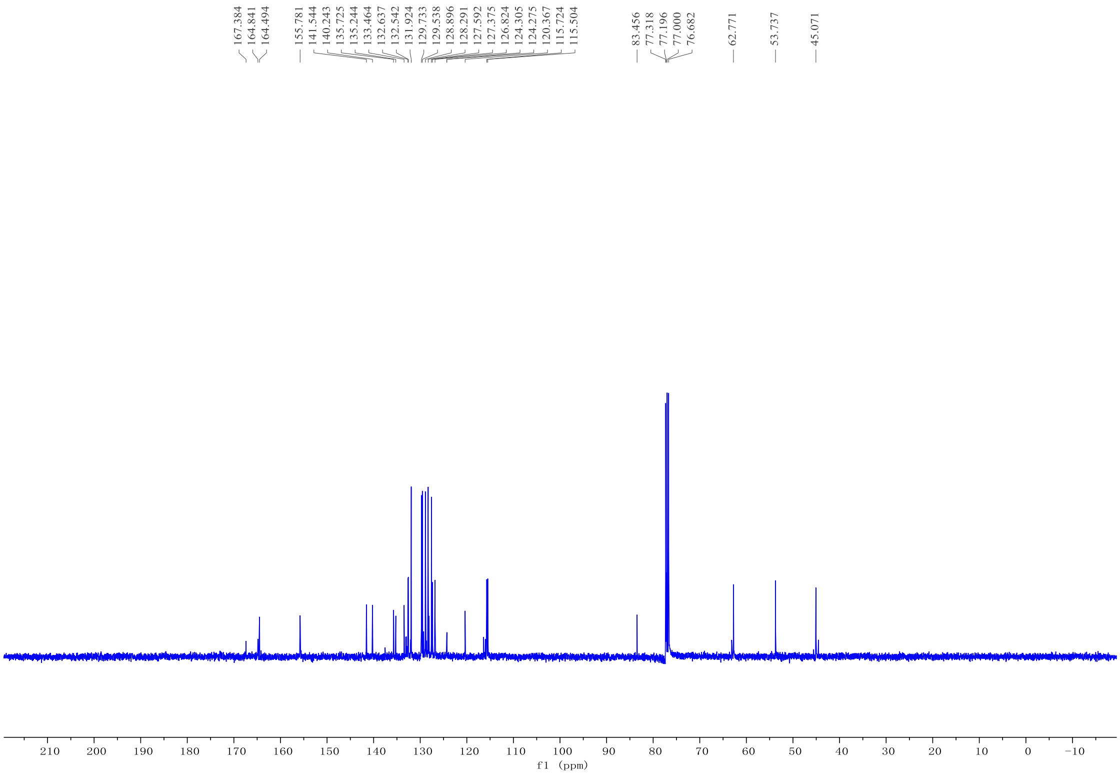


$^{19}F$ NMR (376 MHz, $CDCl_3$)

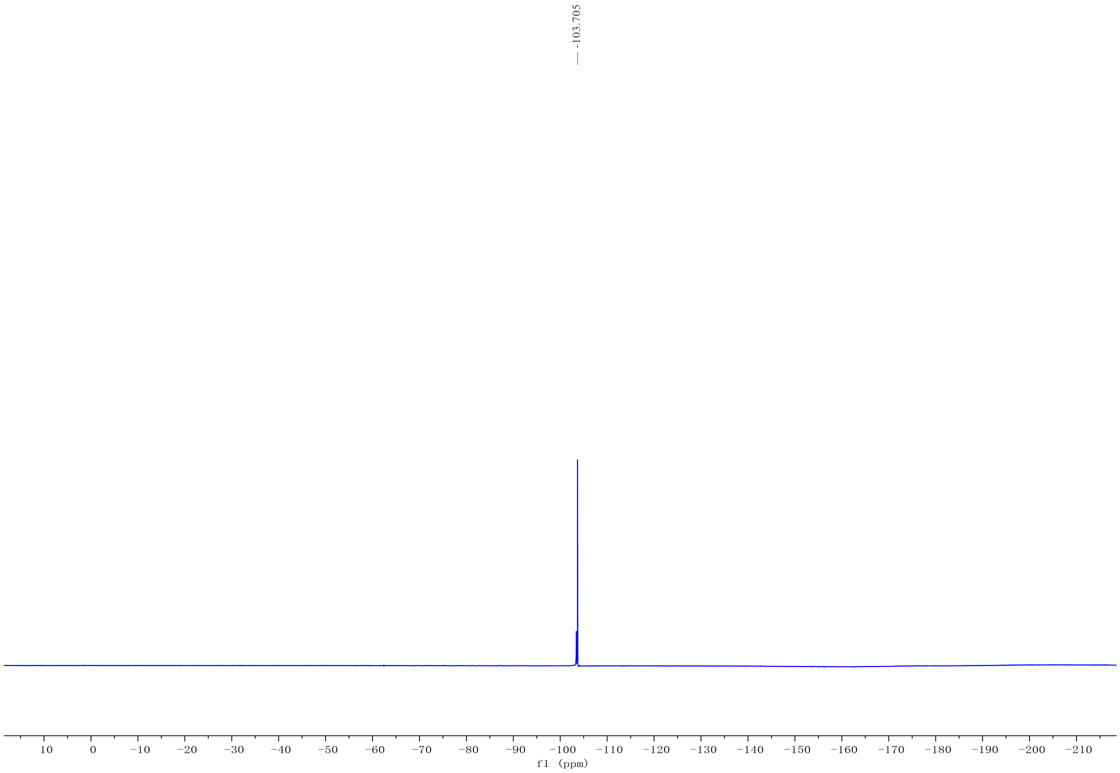

**$^{1}$H, $^{13}$C NMR and $^{19}$F NMR Spectra for Compound (*Z*)-3aam**:

$^{1}$H NMR (400 MHz, $CDCl_3$)

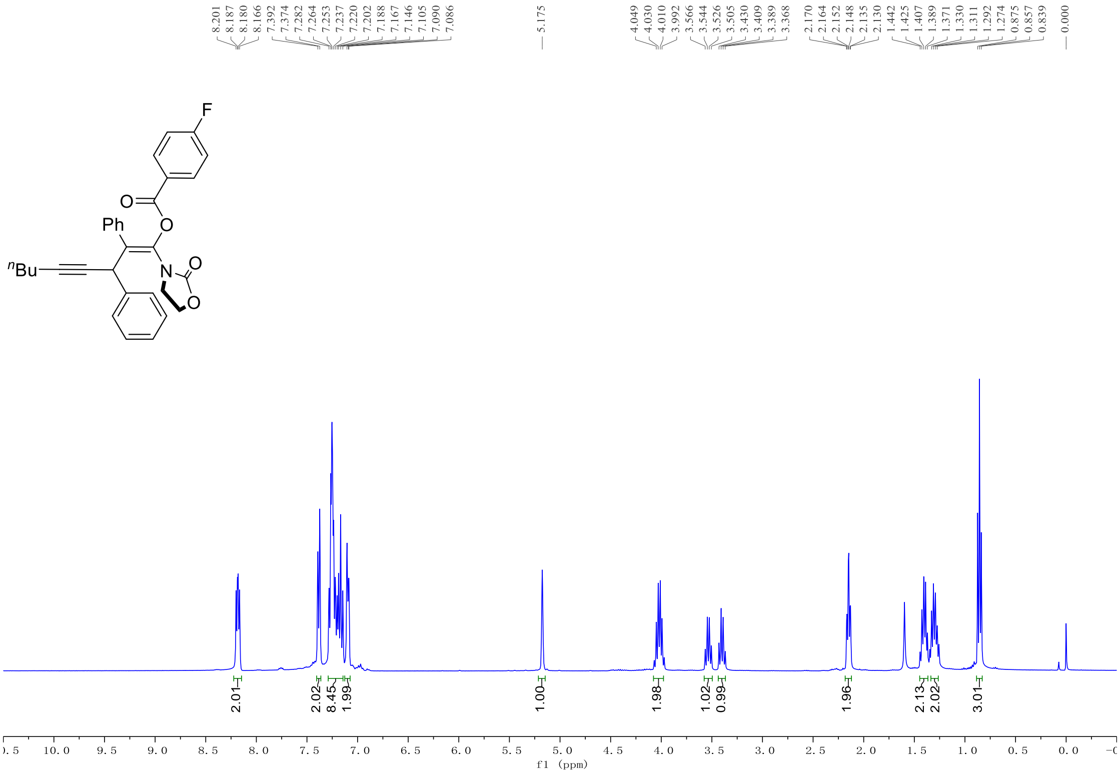

$^{13}$C NMR (100 MHz, $CDCl_3$)

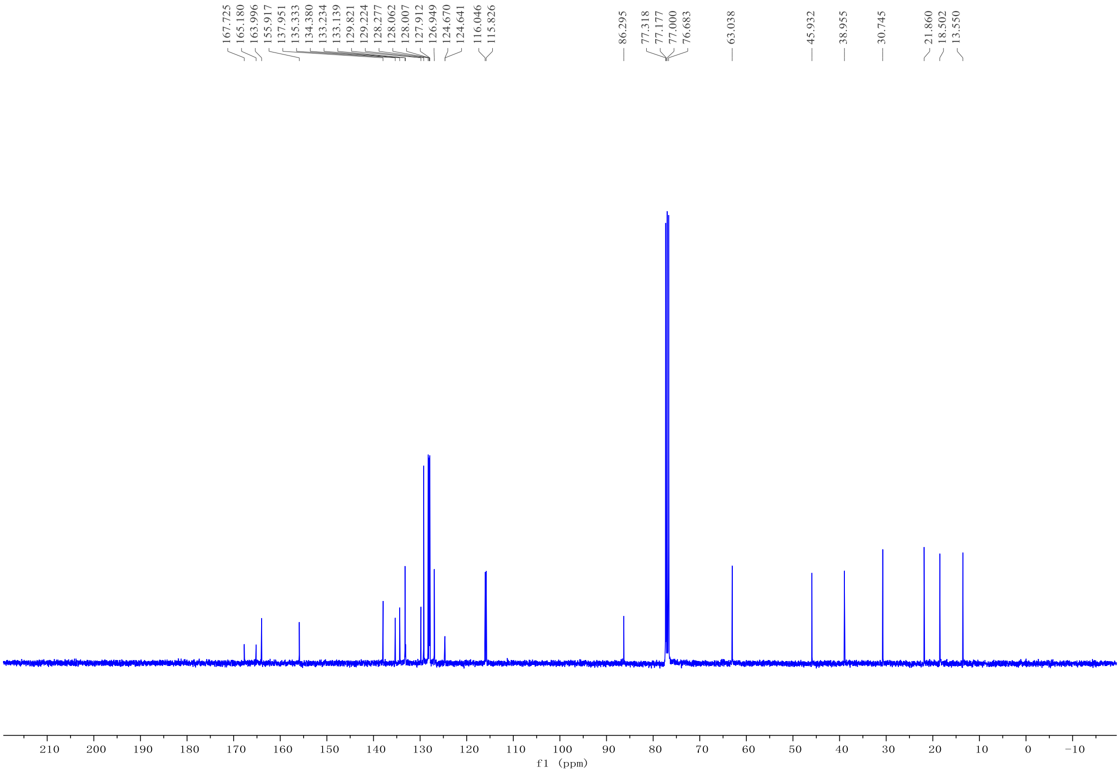

$^{19}$F NMR (376 MHz, $CDCl_3$)

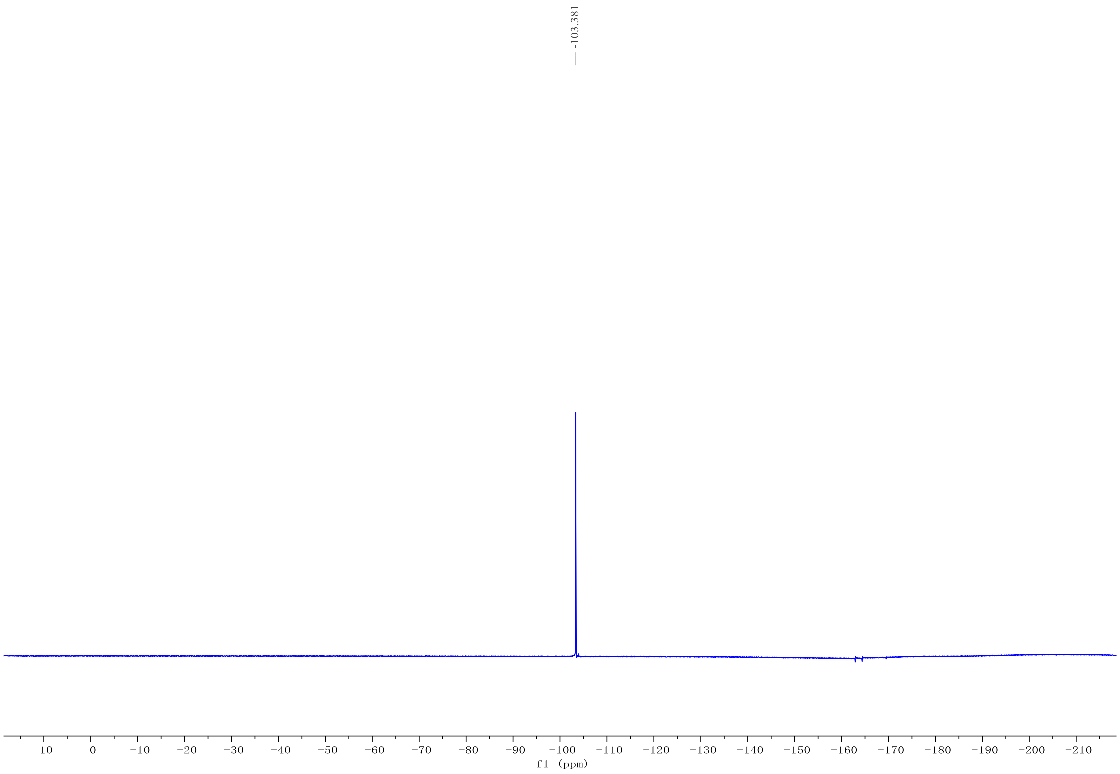

**$^{1}$H, $^{13}$C NMR and $^{19}$F NMR Spectra for Compound (*Z*)-3aan**:

$^{1}$H NMR (400 MHz, $CDCl_3$)

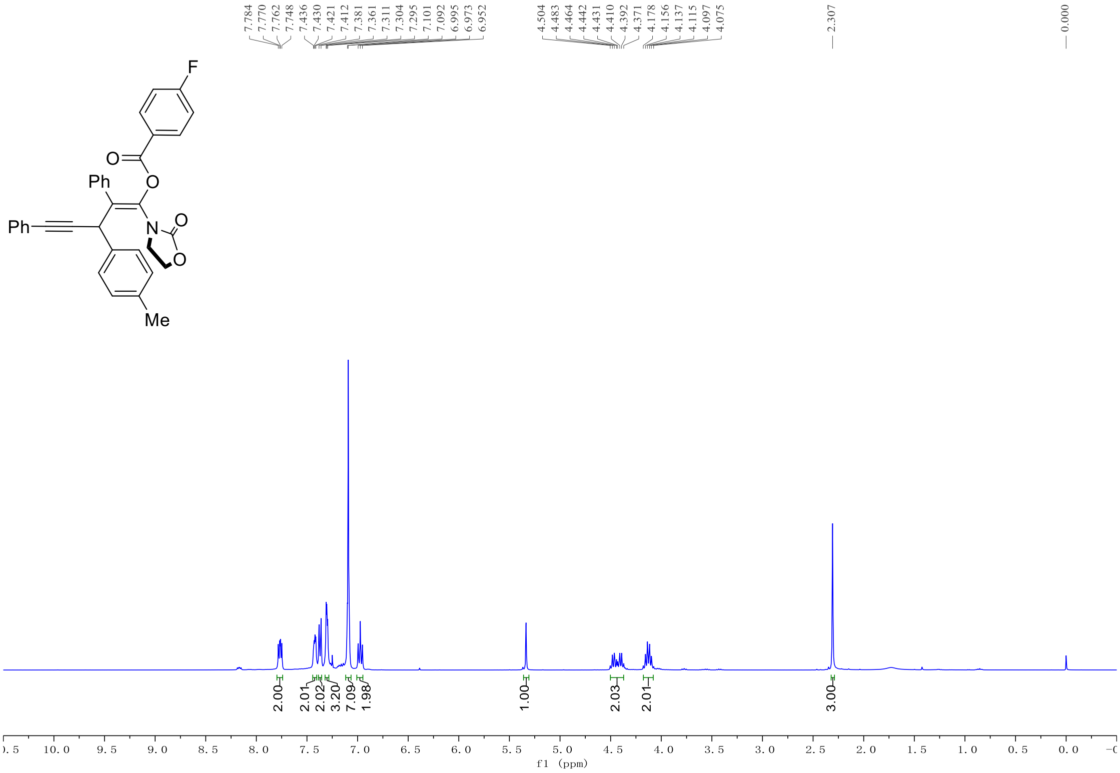

$^{13}C$ NMR (100 MHz, $CDCl_3$)

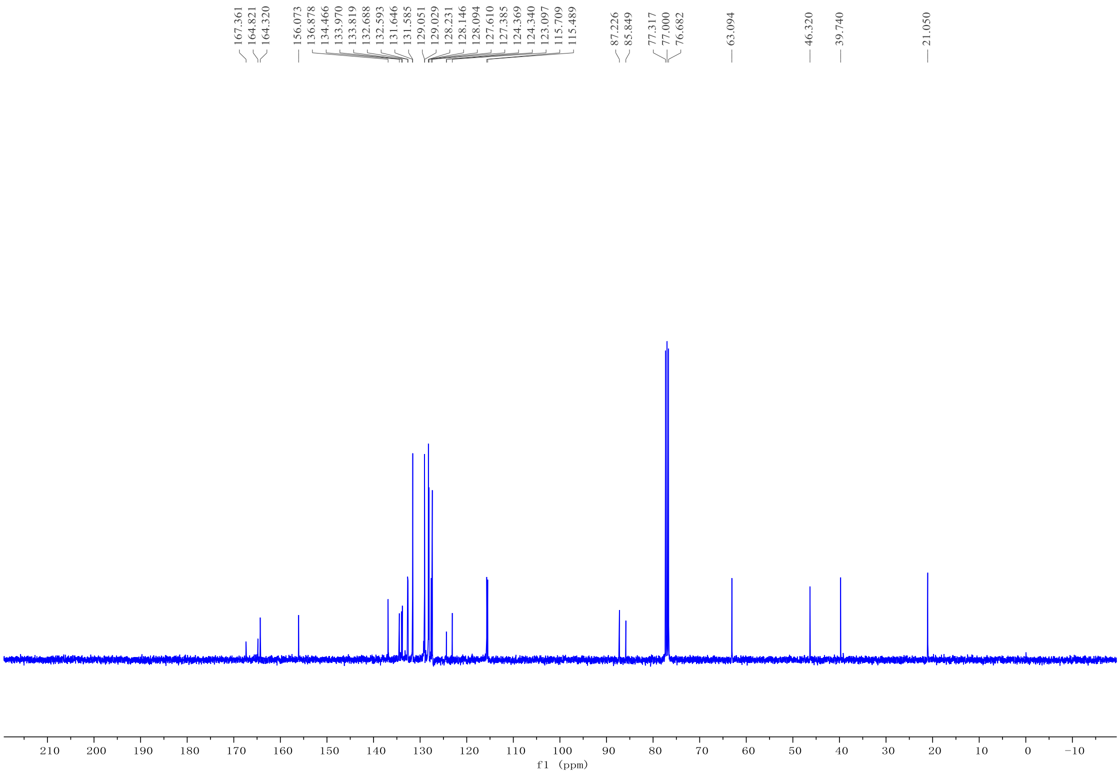


$^{19}F$ NMR (376 MHz, $CDCl_3$)

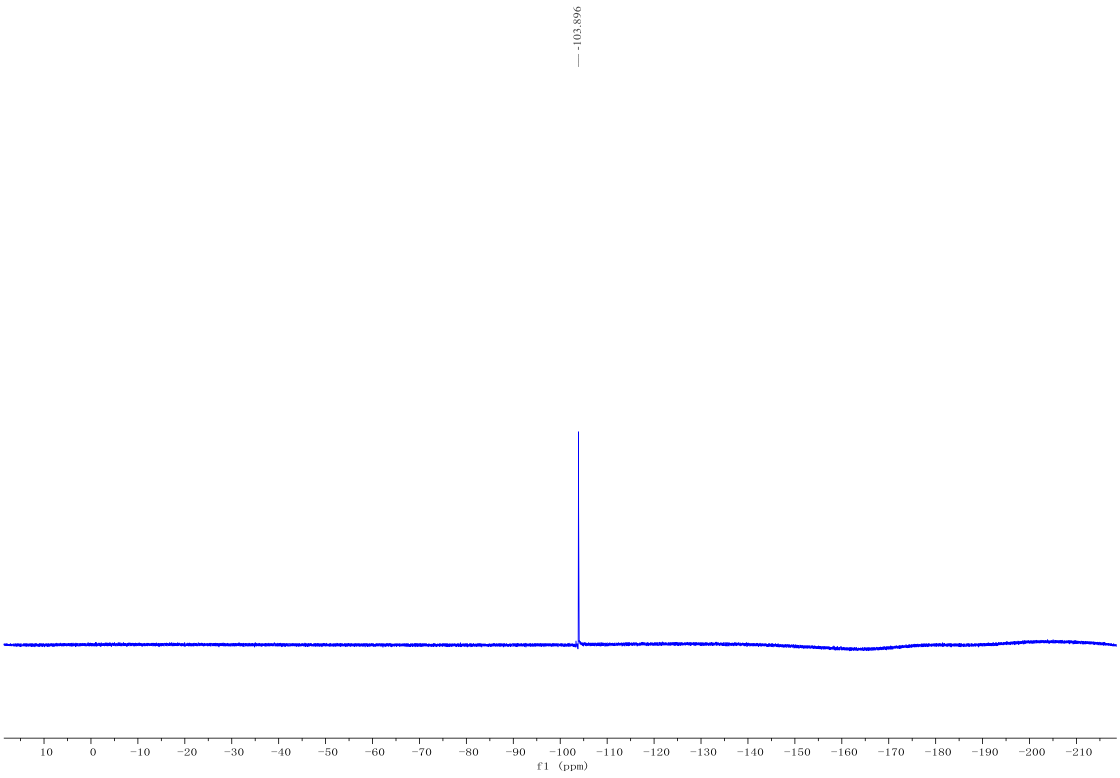

## $^{1}H$, $^{13}C$ NMR and $^{19}F$ NMR Spectra for Compound (*Z*)-3aao:

$^{1}H$ NMR (400 MHz, $CDCl_3$)

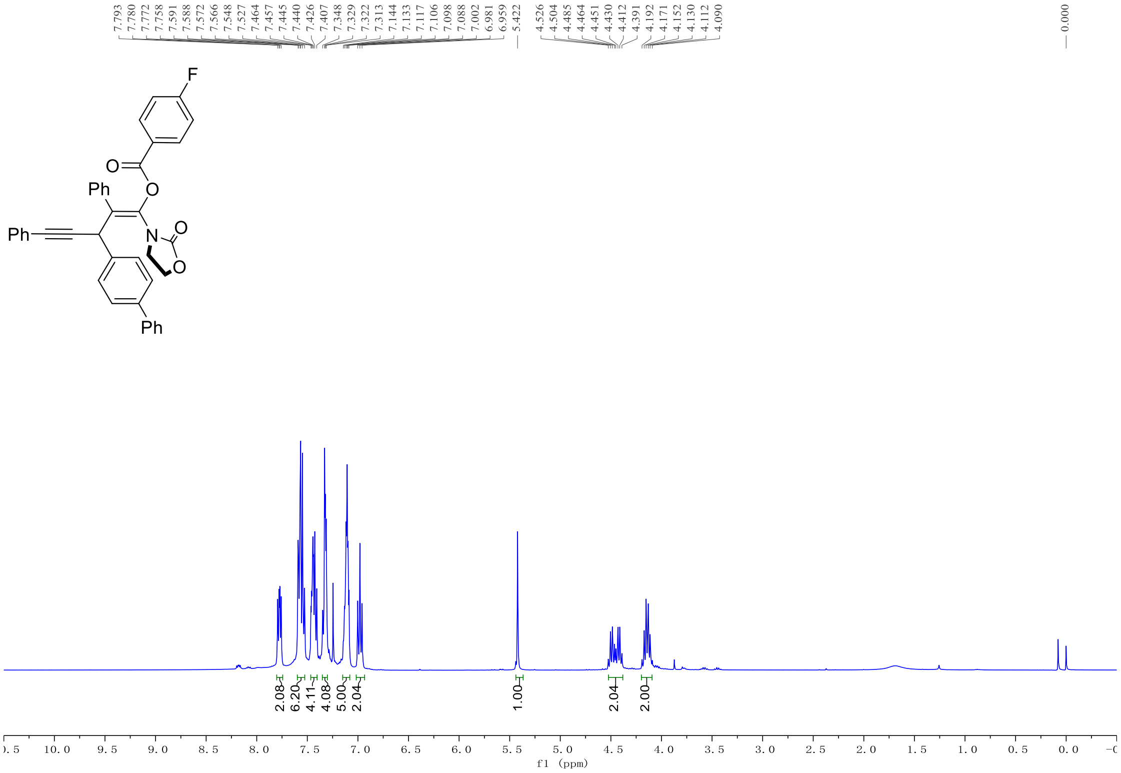


$^{13}C$ NMR (100 MHz, $CDCl_3$)

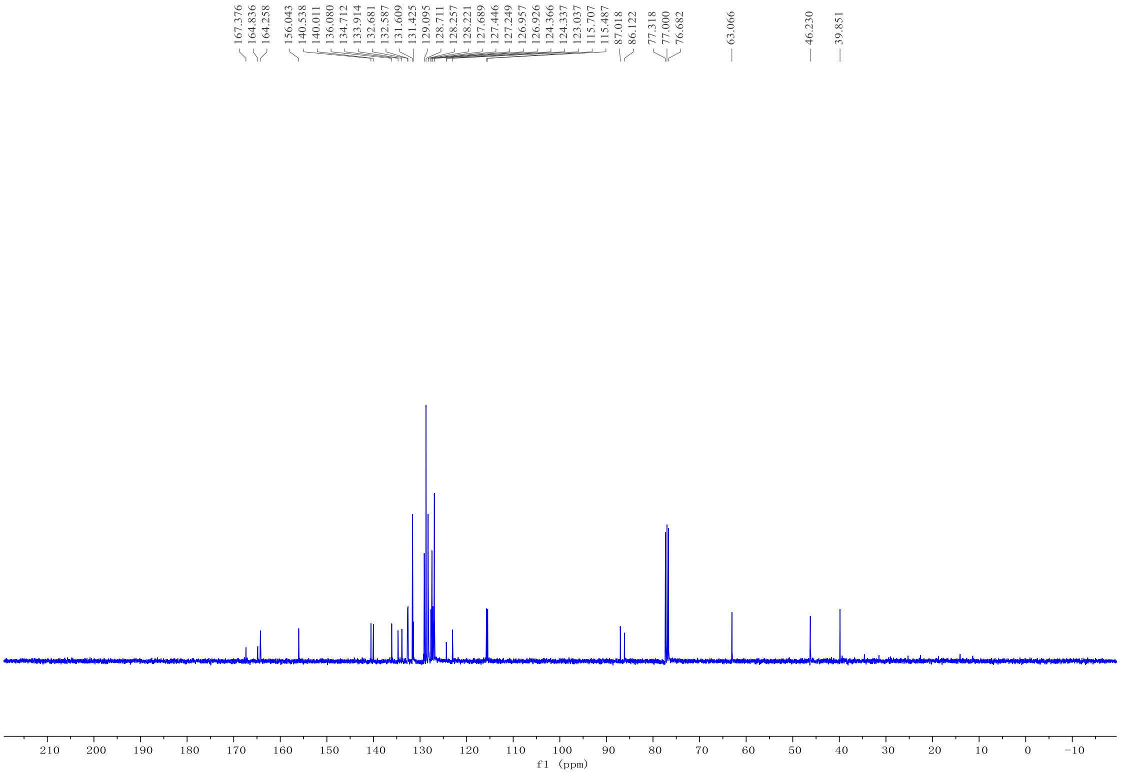

$^{19}F$ NMR (376 MHz, $CDCl_3$)

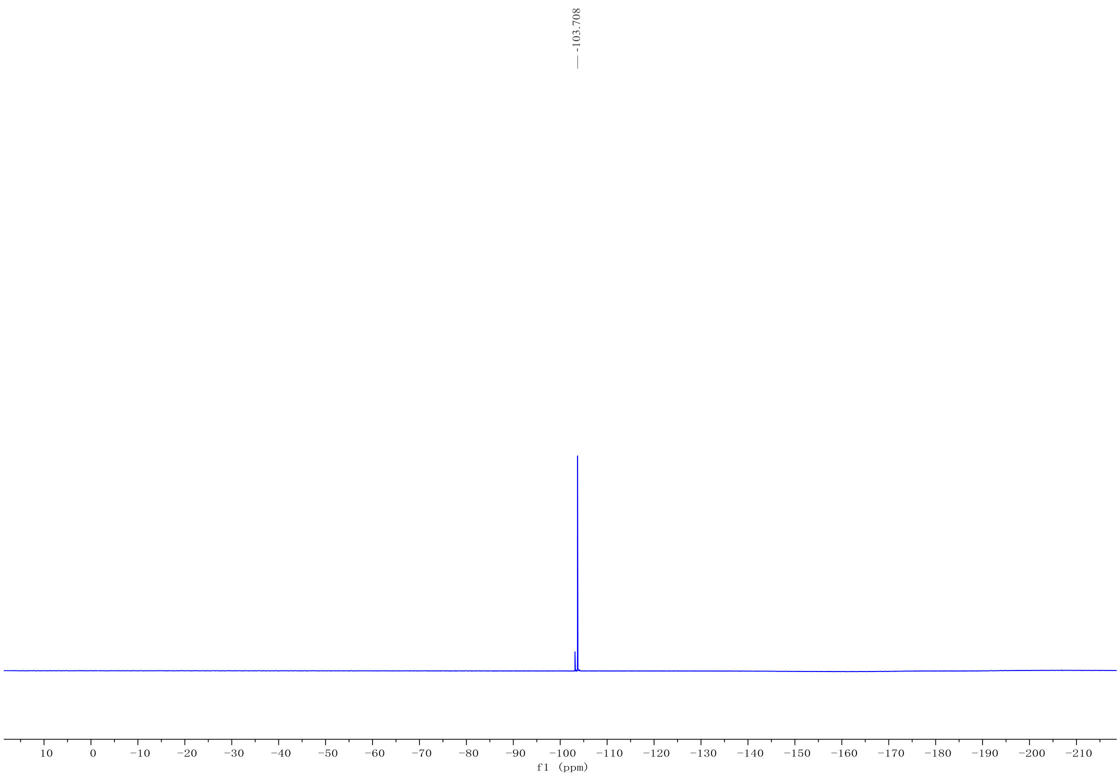

**$^{1}H$, $^{13}C$ NMR and $^{19}F$ NMR Spectra for Compound (*Z*)-3aap**:

$^{1}H$ NMR (400 MHz, $CDCl_3$)

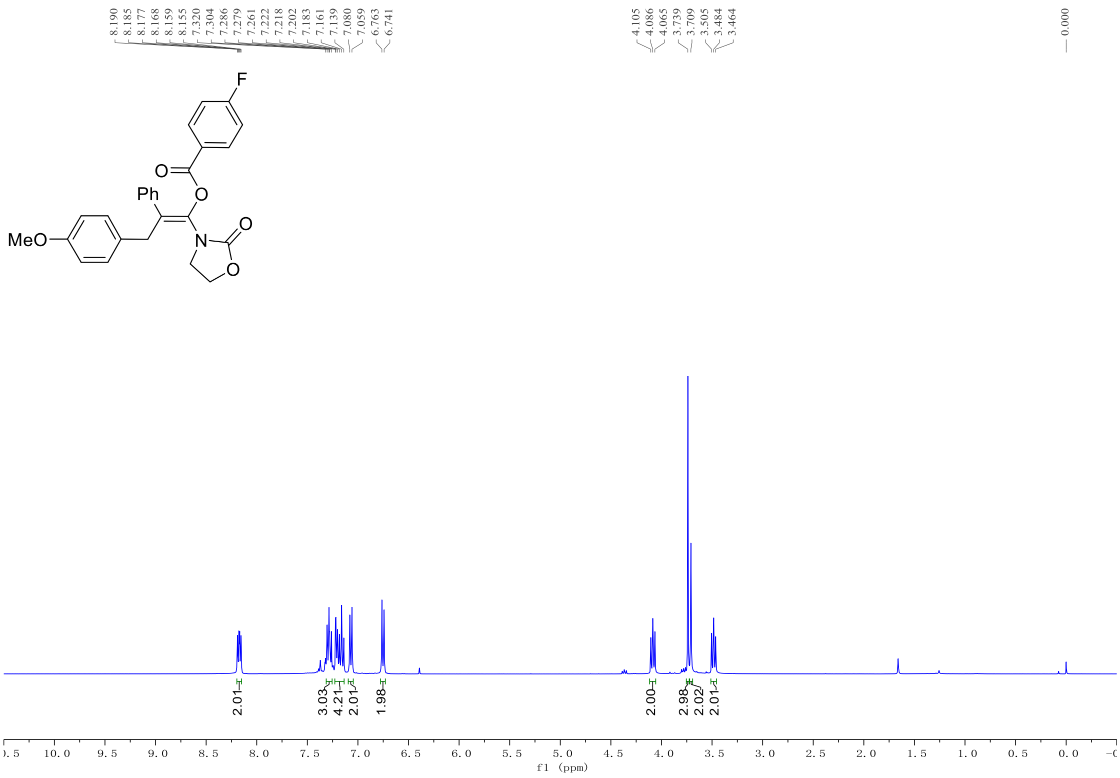

$^{13}C$ NMR (100 MHz, $CDCl_3$)

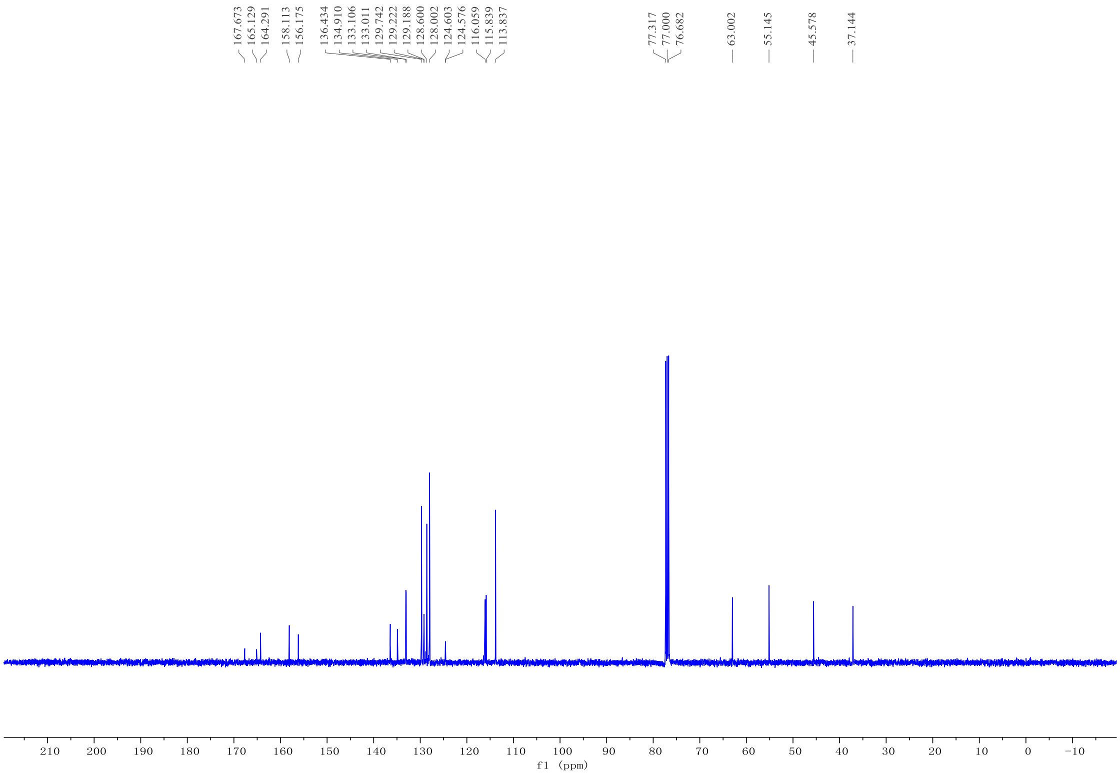


$^{19}F$ NMR (376 MHz, $CDCl_3$)

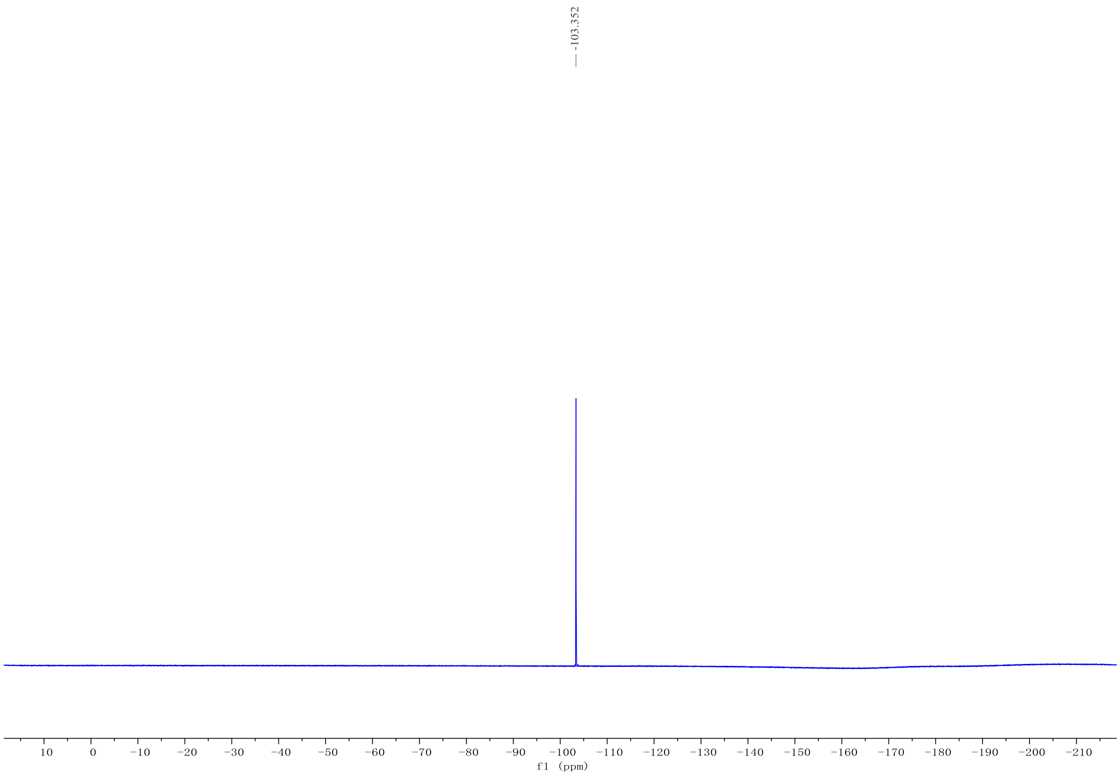

**$^{1}$H, $^{13}$C NMR and $^{19}$F NMR Spectra for Compound (*Z*)-3aaq and its regioisomer**:

$^{1}$H NMR (400 MHz, $CDCl_3$)

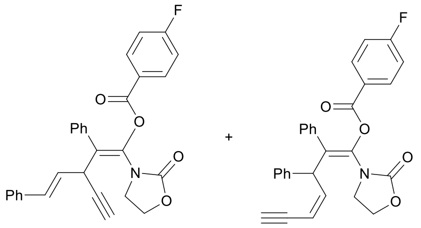


(*Z*)-**3aaq** (*Z*)-**3aaq'**

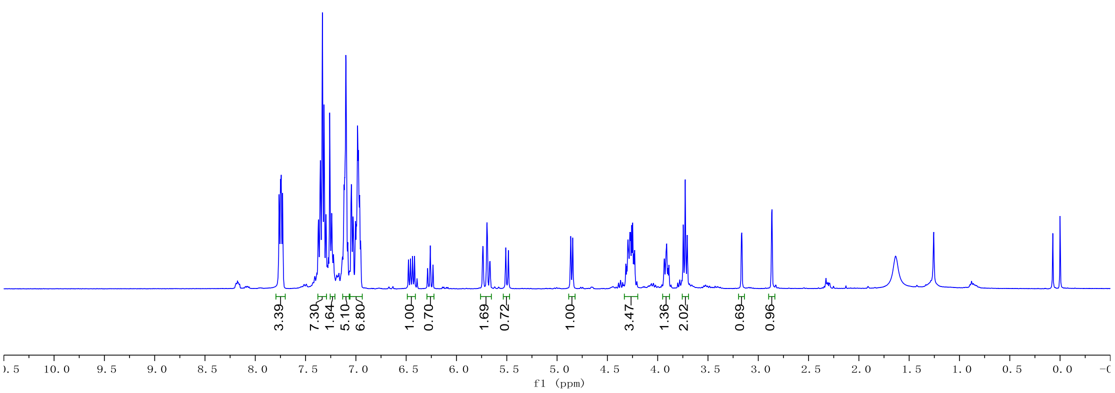


$^{13}$C NMR (100 MHz, $CDCl_3$)

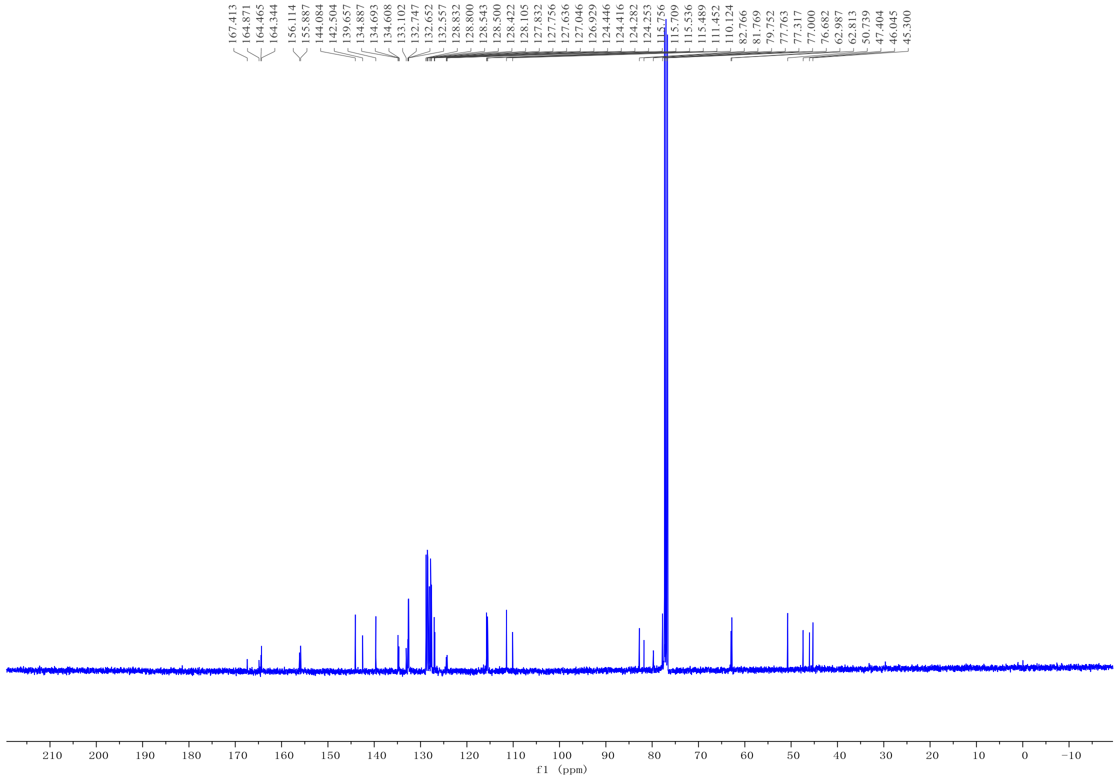

$^{19}$F NMR (400 MHz, CDCl$_3$)

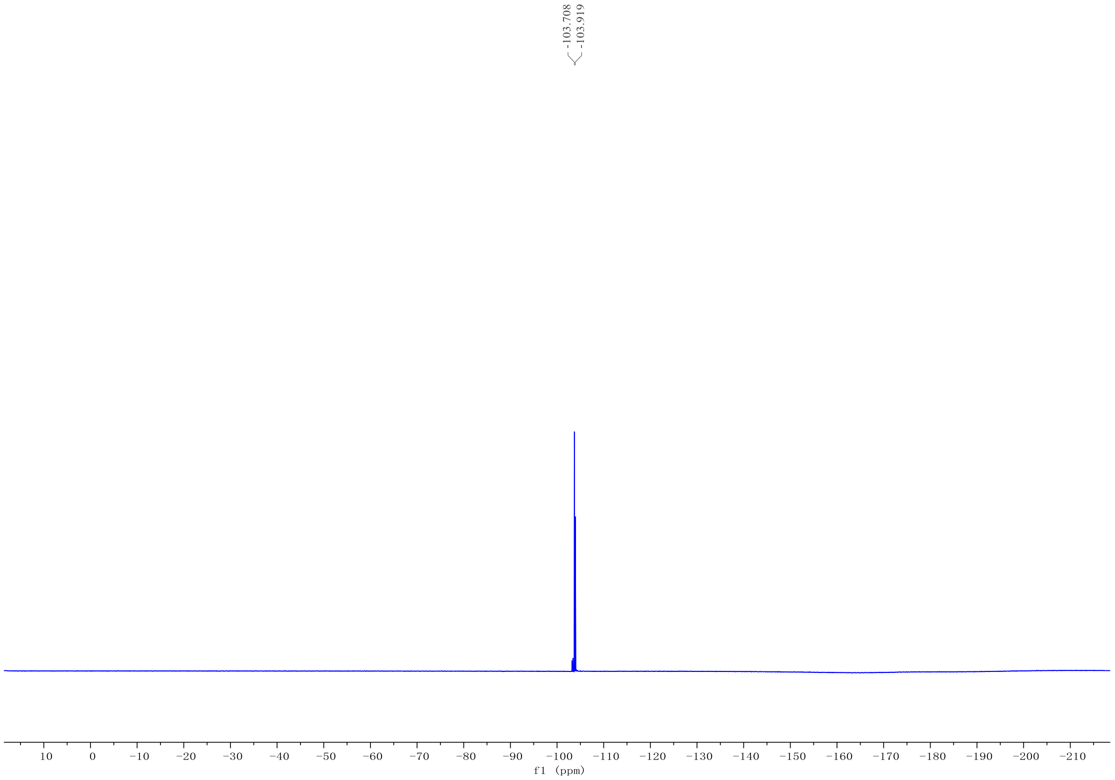

**$^{1}$H, $^{13}$C NMR and $^{19}$F NMR Spectra for Compound (*Z*)-3aar**:

$^{1}$H NMR (400 MHz, CDCl$_3$)

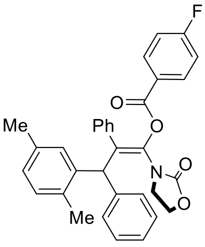


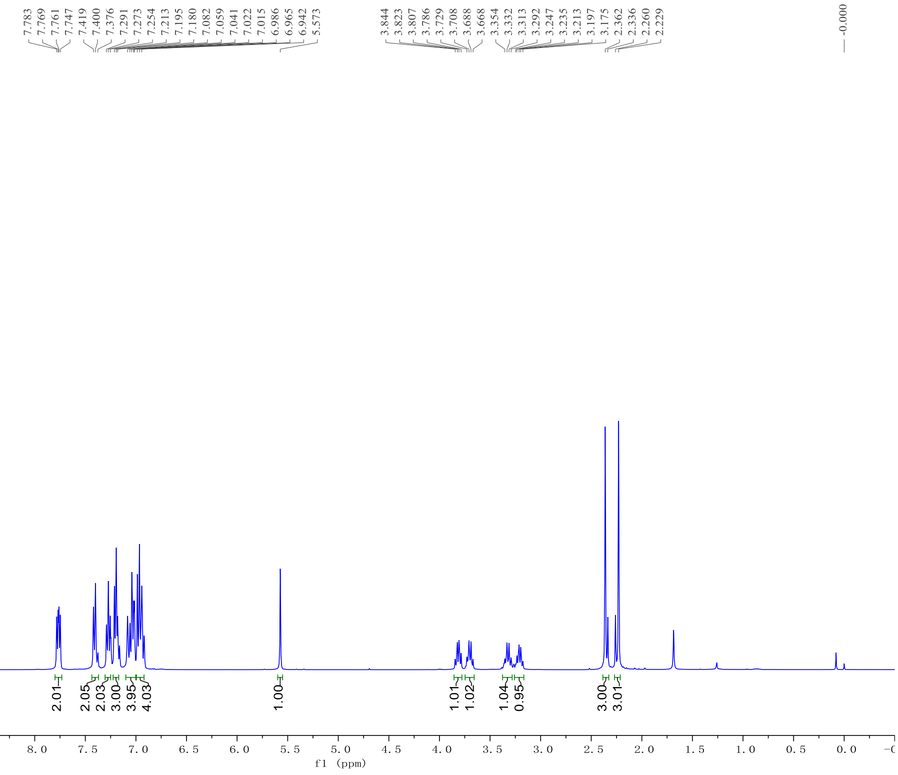

$^{13}C$ NMR (100 MHz, $CDCl_3$)

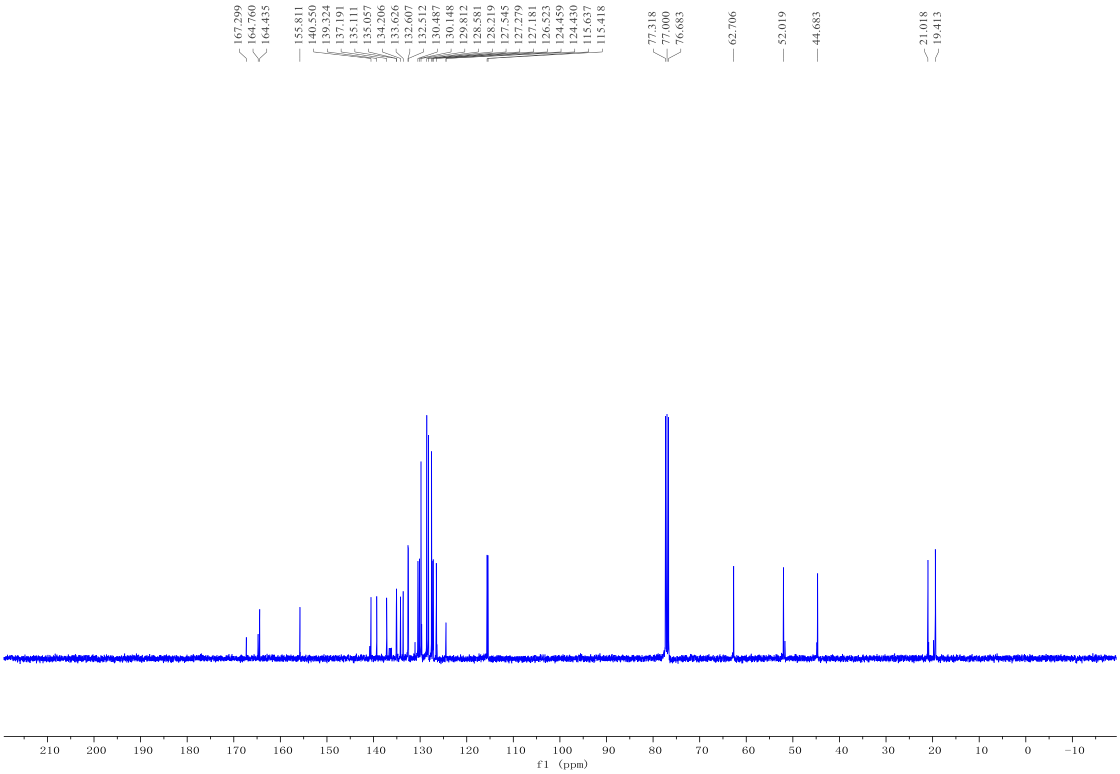


$^{19}F$ NMR (376 MHz, $CDCl_3$)

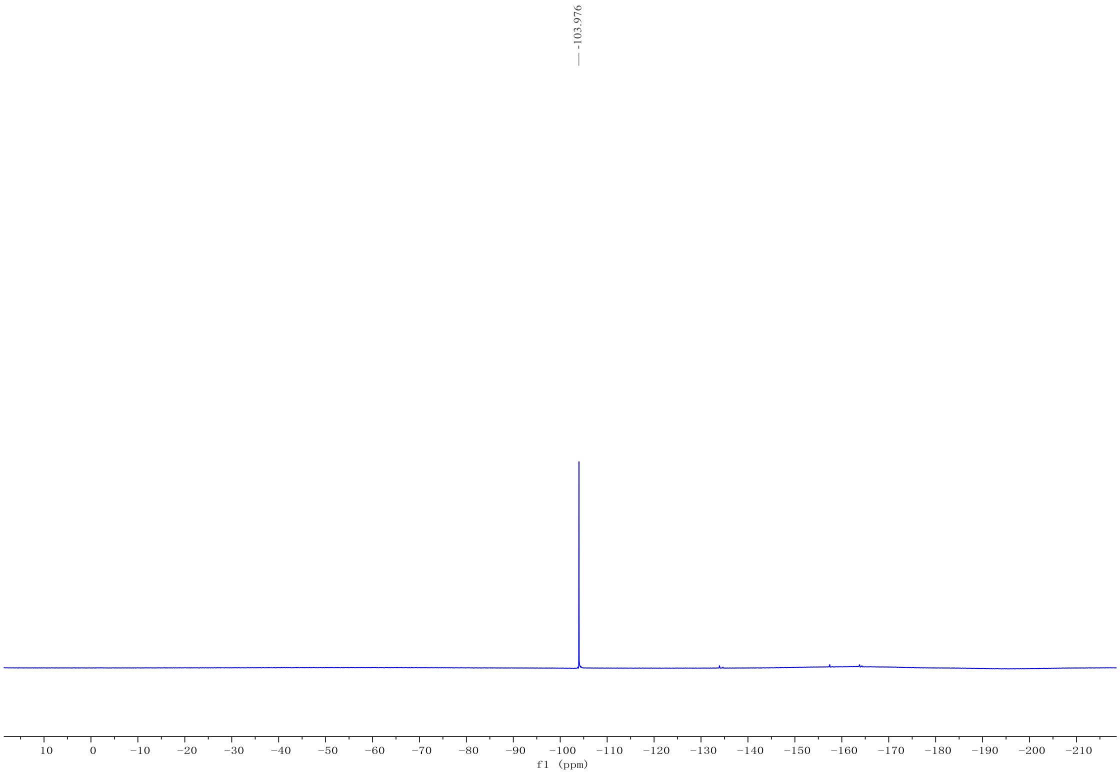

## **$^{1}$H, $^{13}$C NMR and $^{19}$F NMR Spectra for Compound (*Z*)-3ba**:

$^{1}$H NMR (400 MHz, $CDCl_3$)

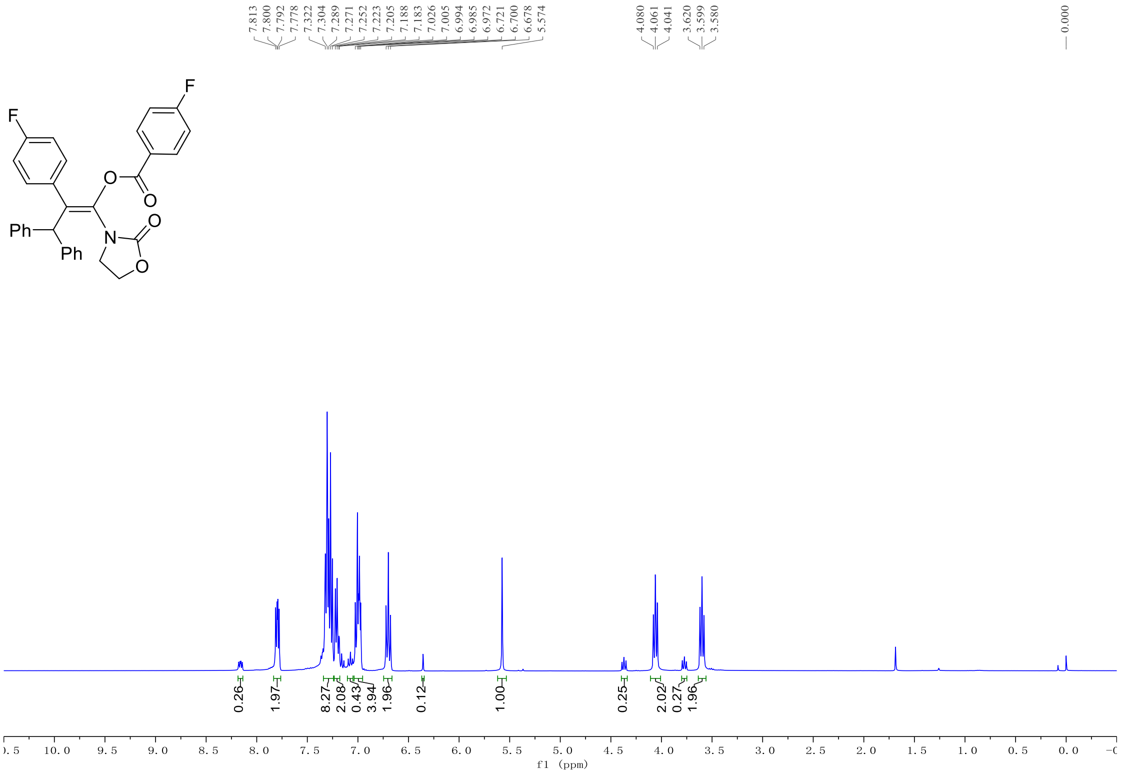


$^{13}$C NMR (100 MHz, $CDCl_3$)

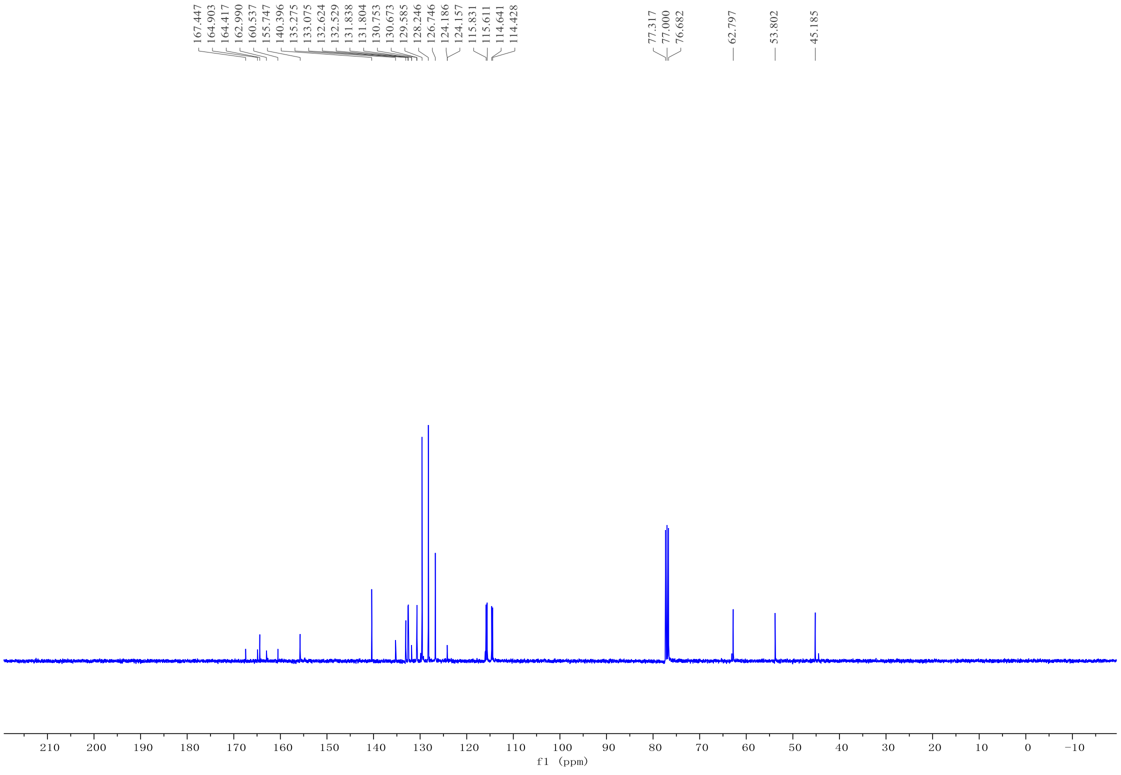

$^{19}F$ NMR (376 MHz, $CDCl_3$)

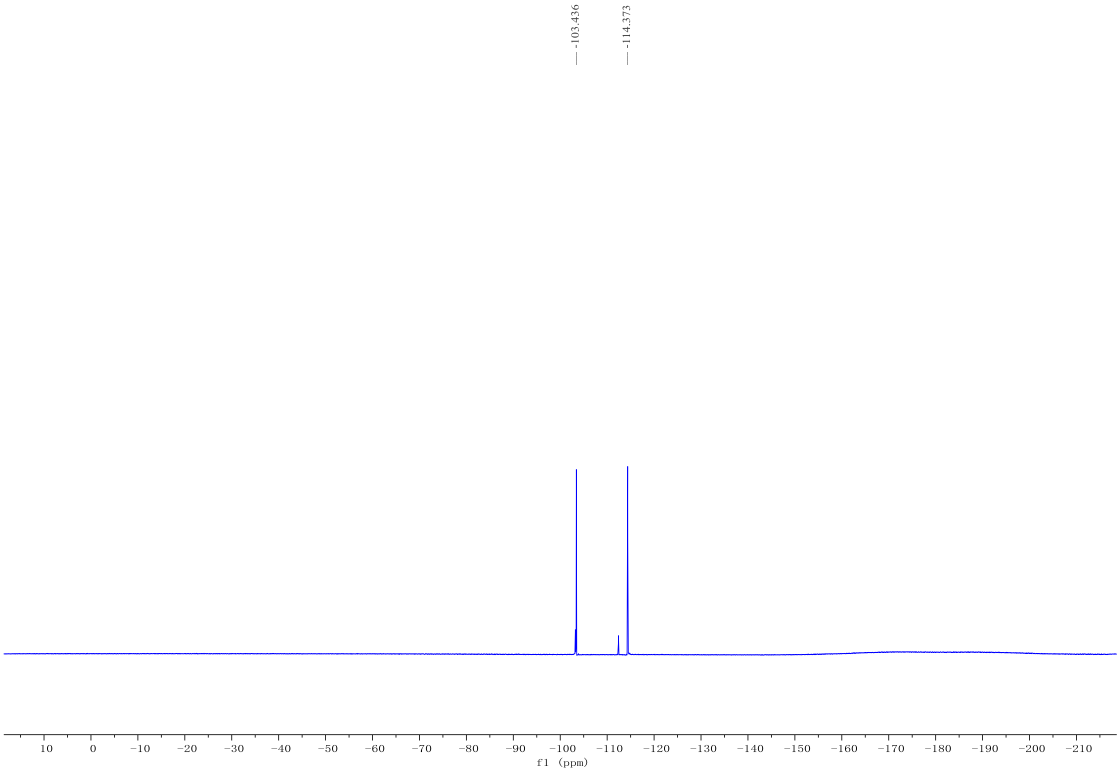

**$^{1}H$, $^{13}C$ NMR and $^{19}F$ NMR Spectra for Compound (*Z*)-3ca:**

$^{1}H$ NMR (400 MHz, $CDCl_3$)

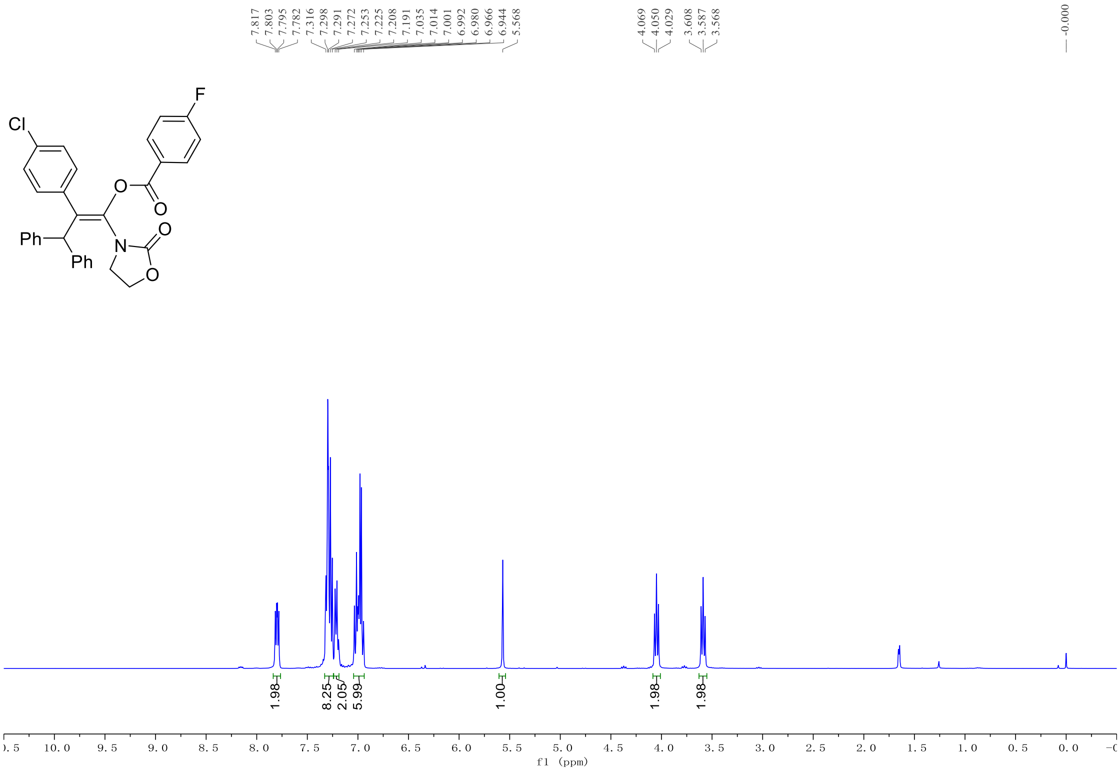

$^{13}C$ NMR (100 MHz, $CDCl_3$)

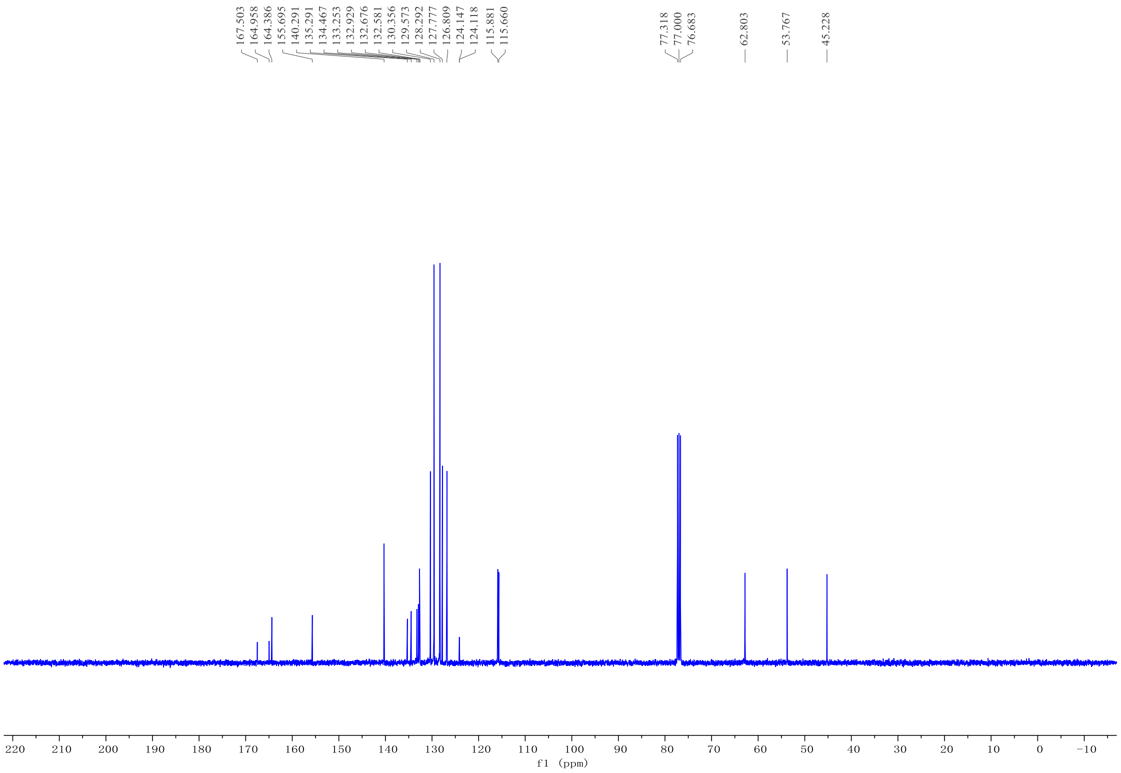


$^{19}F$ NMR (376 MHz, $CDCl_3$)

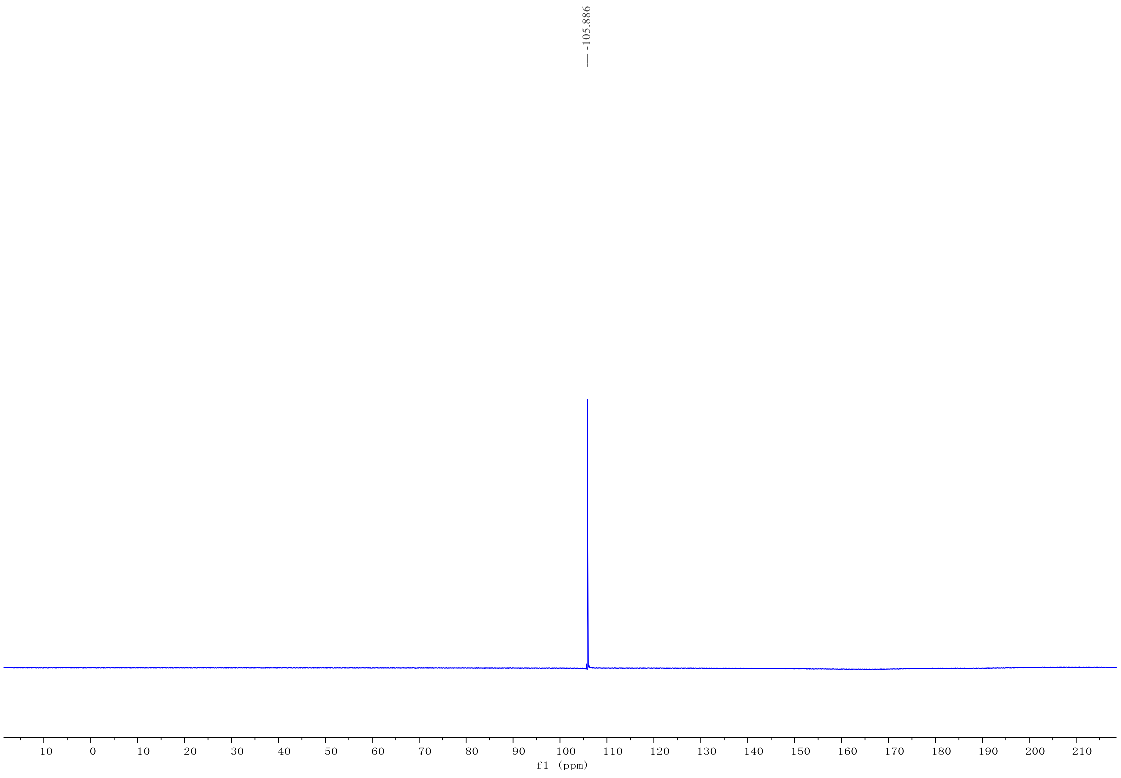

## $^{1}$H, $^{13}$C NMR and $^{19}$F NMR Spectra for Compound (*Z*)-3da:

$^{1}$H NMR (400 MHz, $CDCl_3$)

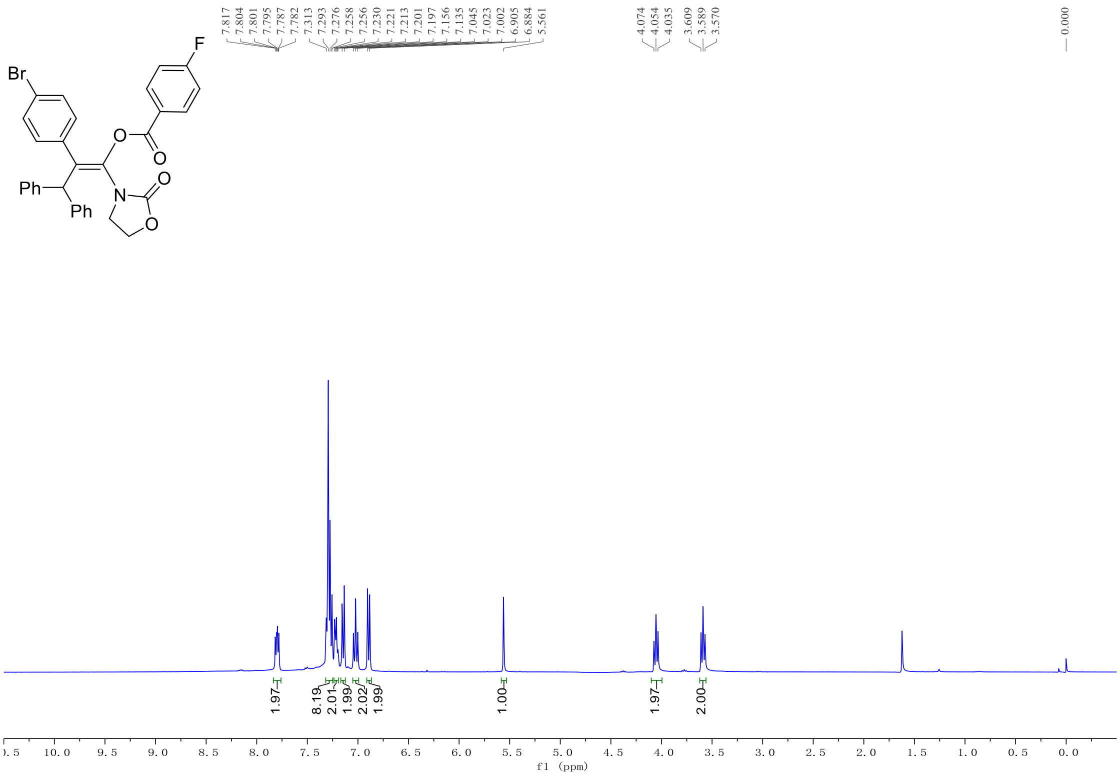


$^{13}$C NMR (100 MHz, $CDCl_3$)

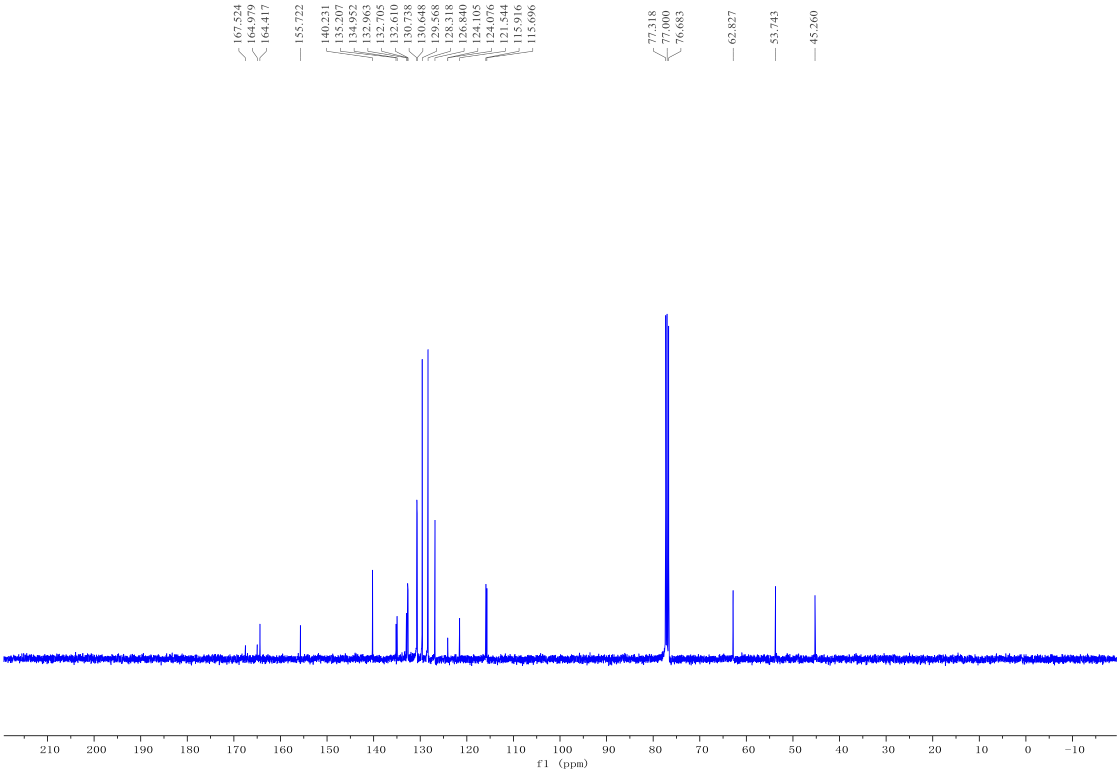

$^{19}F$ NMR (376 MHz, $CDCl_3$)

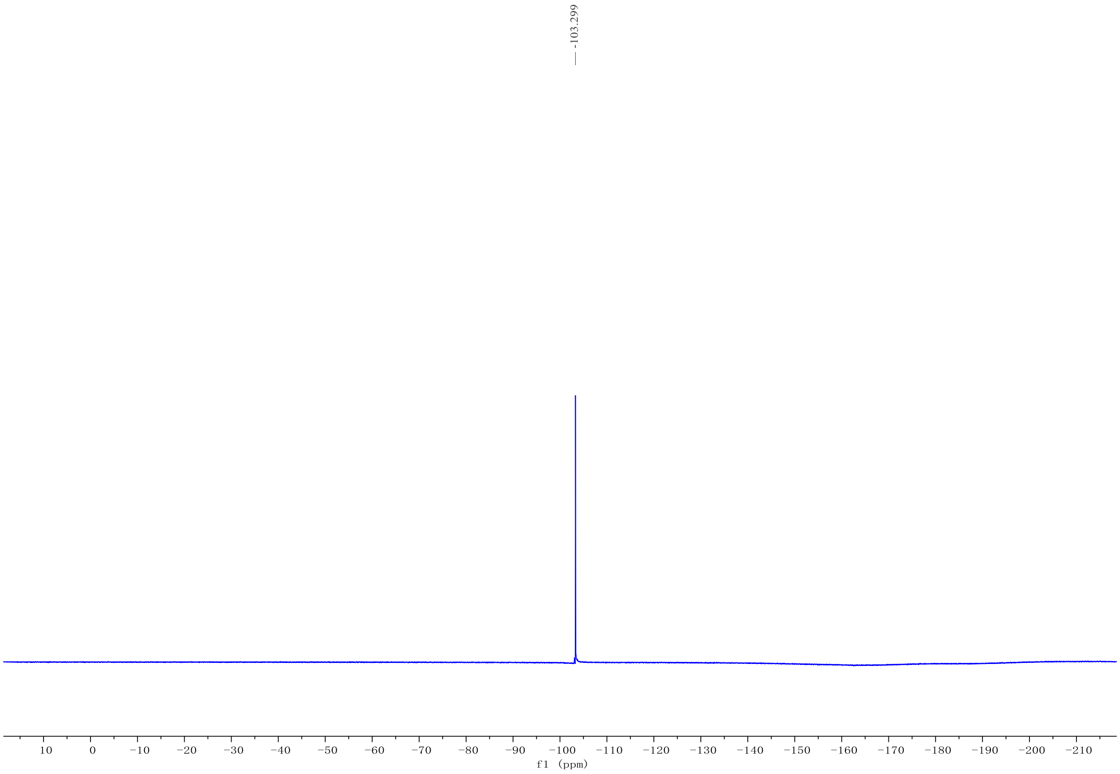

**$^{1}H$, $^{13}C$ NMR and $^{19}F$ NMR Spectra for Compound (*Z*)-3ea:**

$^{1}H$ NMR (400 MHz, $CDCl_3$)

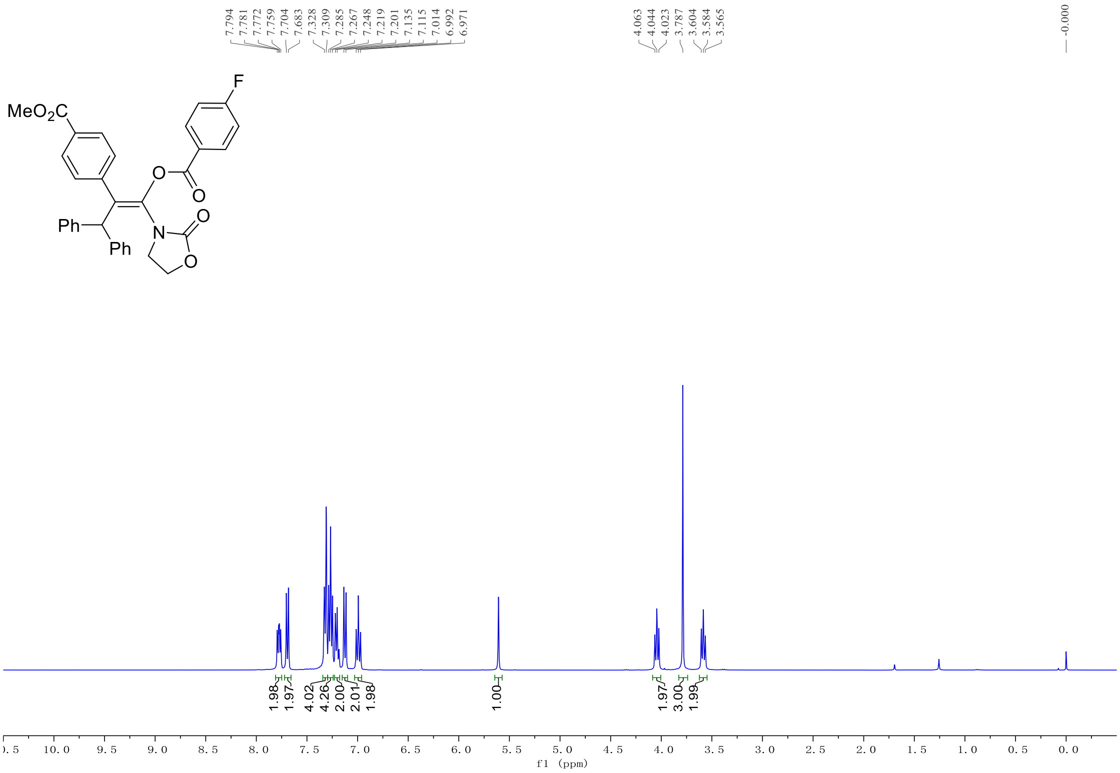

$^{13}C$ NMR (100 MHz, $CDCl_3$)

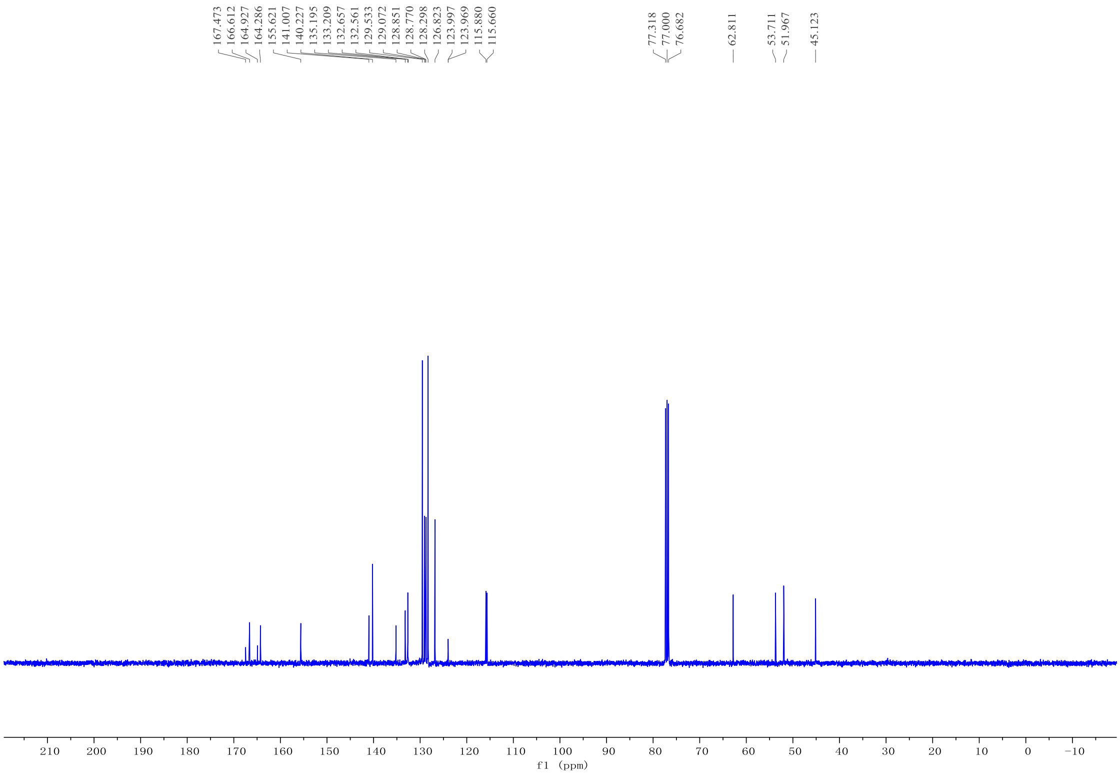


$^{19}F$ NMR (376 MHz, $CDCl_3$)

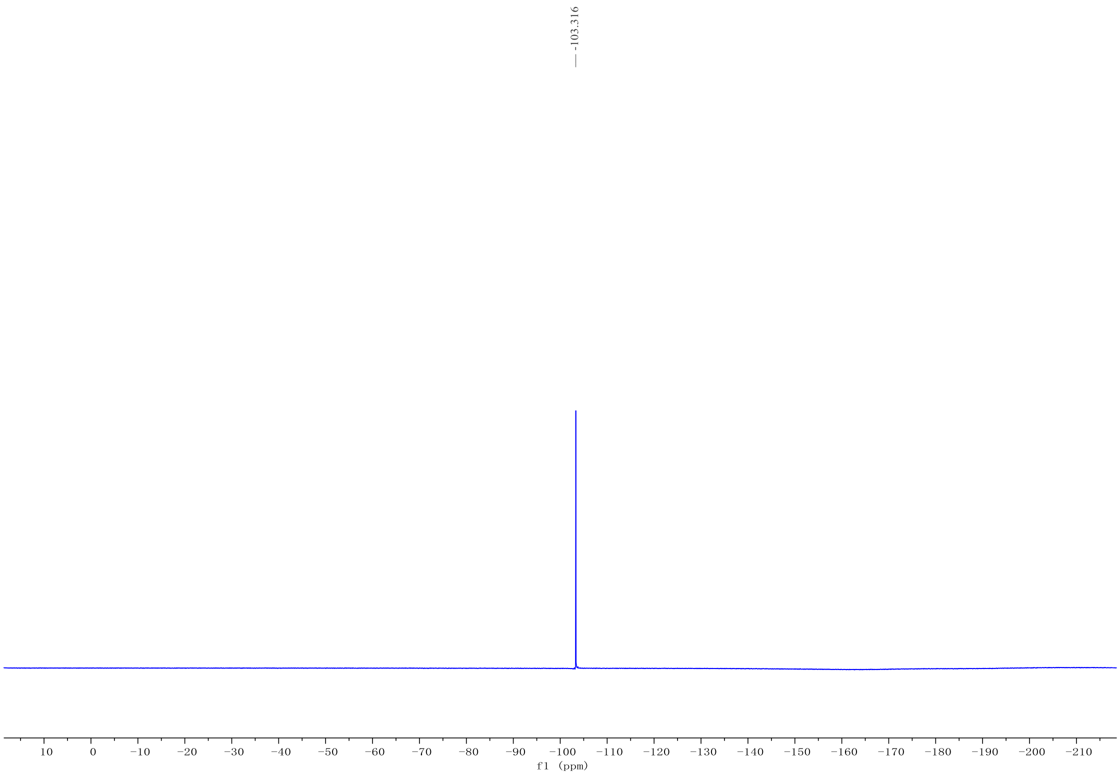

## $^{1}$H, $^{13}$C NMR and $^{19}$F NMR Spectra for Compound (*Z*)-3fa:

$^{1}$H NMR (400 MHz, $CDCl_3$)

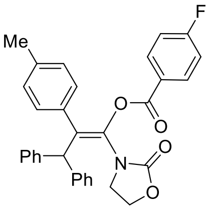

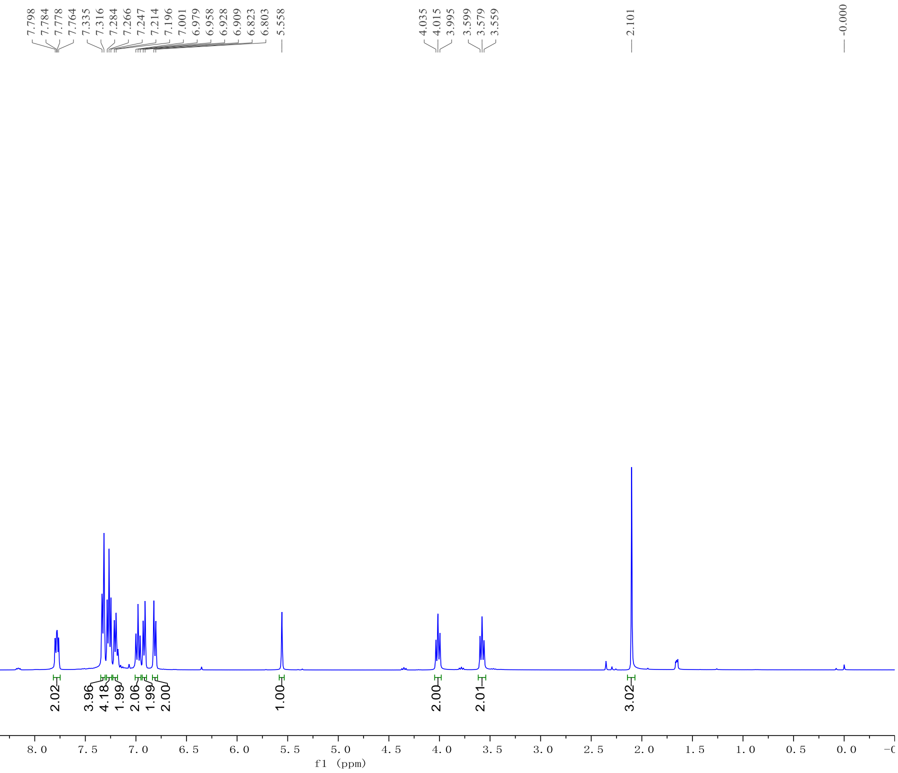

$^{13}$C NMR (100 MHz, $CDCl_3$)

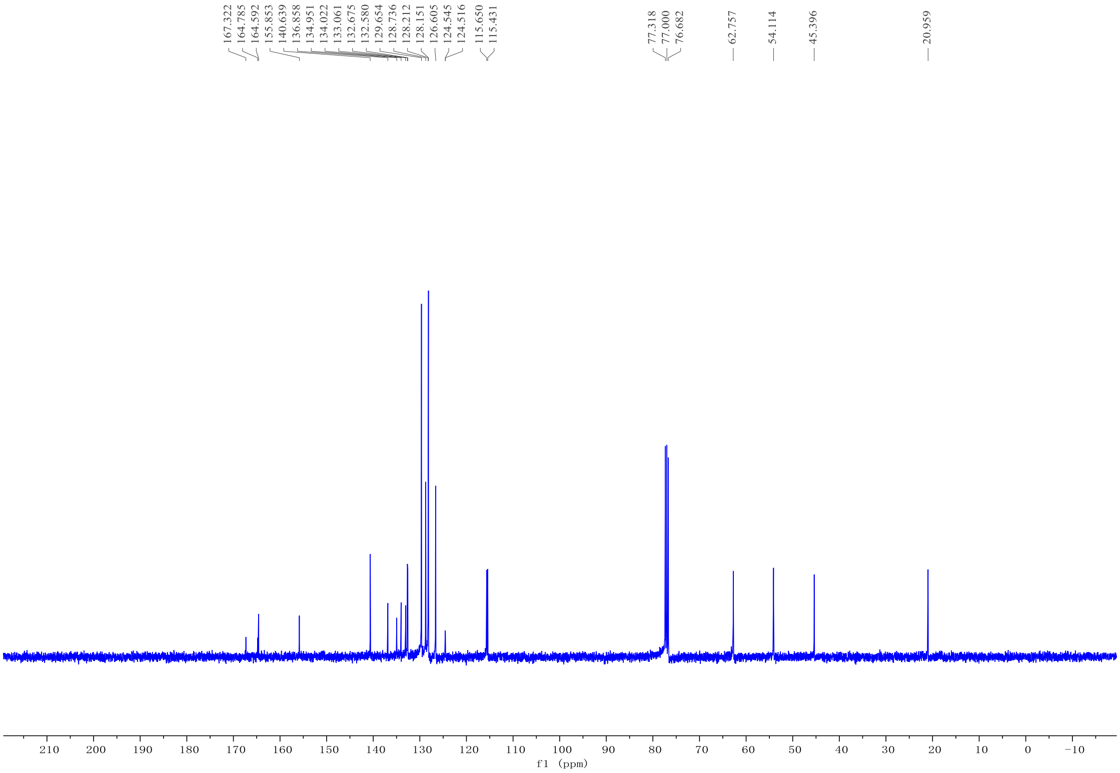

$^{19}F$ NMR (376 MHz, $CDCl_3$)

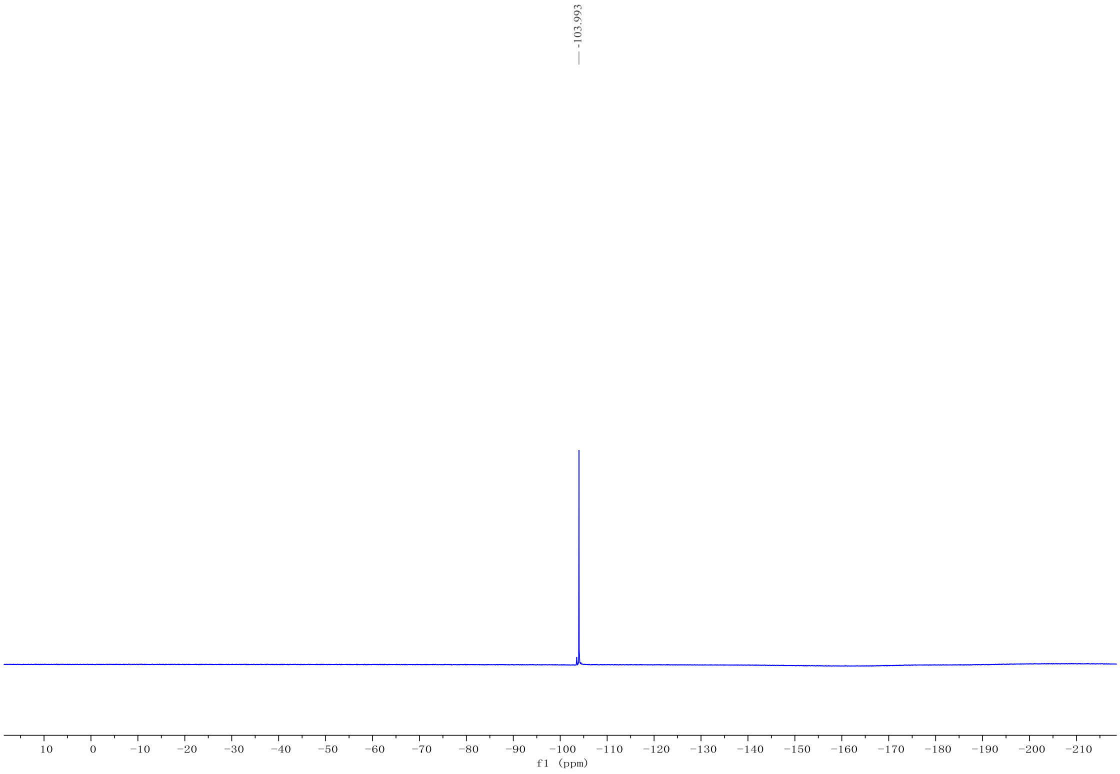


**$^{1}H$, $^{13}C$ NMR and $^{19}F$ NMR Spectra for Compound (*Z*)-3ga:**

$^{1}H$ NMR (400 MHz, $CDCl_3$)

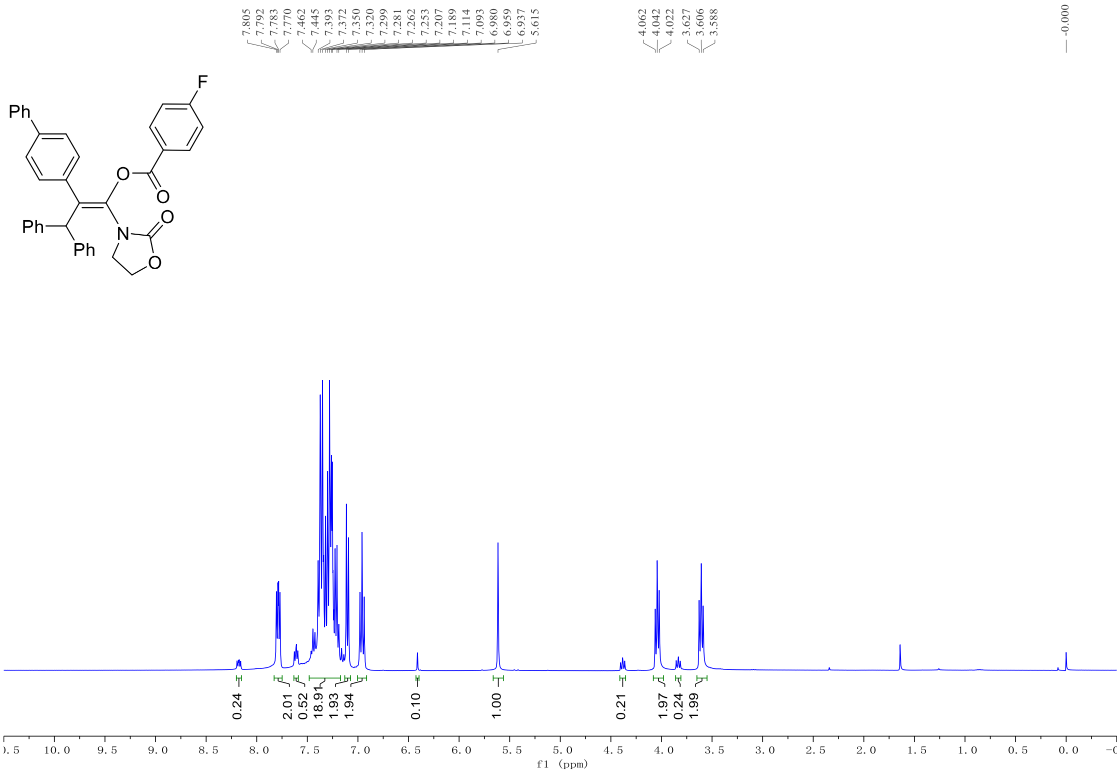

$^{13}C$ NMR (100 MHz, $CDCl_3$)

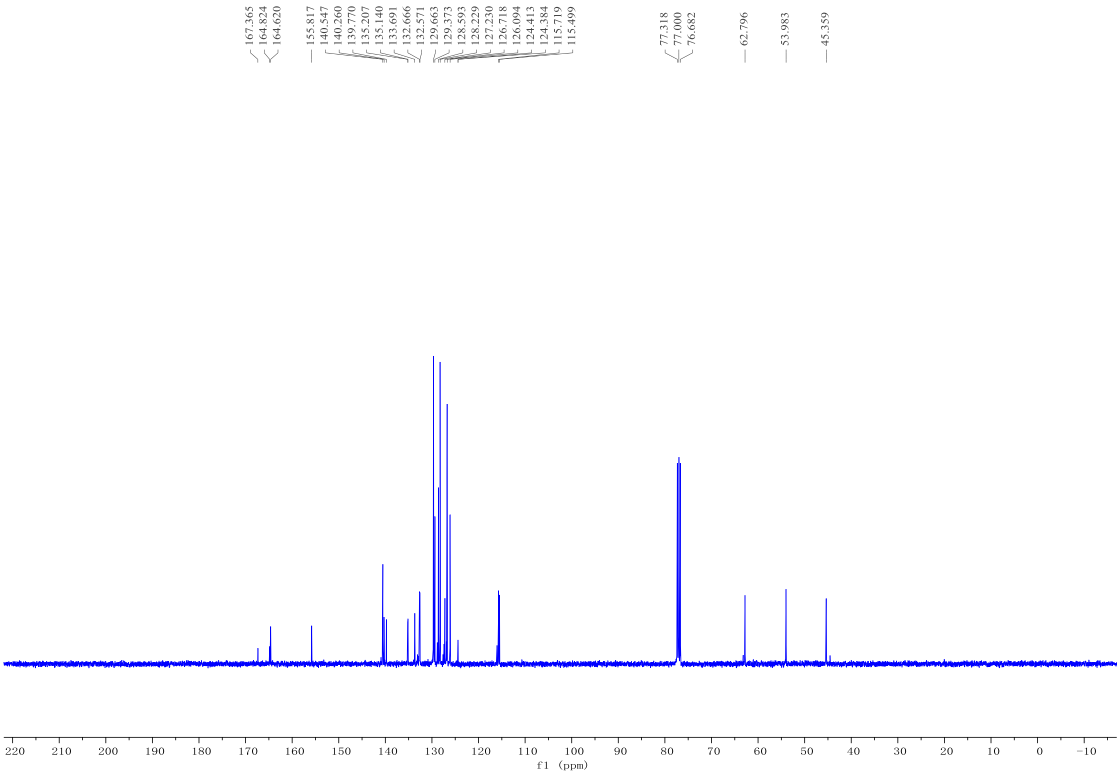


$^{19}F$ NMR (376 MHz, $CDCl_3$)

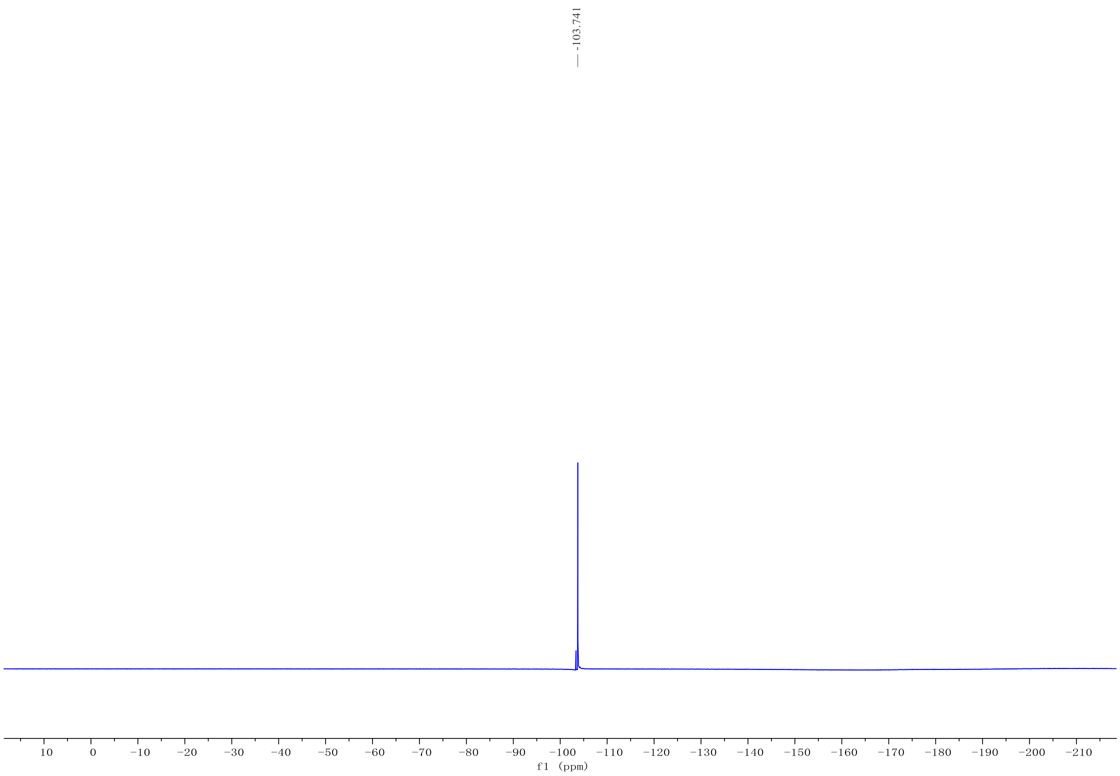

# $^{1}$H, $^{13}$C NMR and $^{19}$F NMR Spectra for Compound (*Z*)-3ha:

$^1$H NMR (400 MHz, $CDCl_3$)

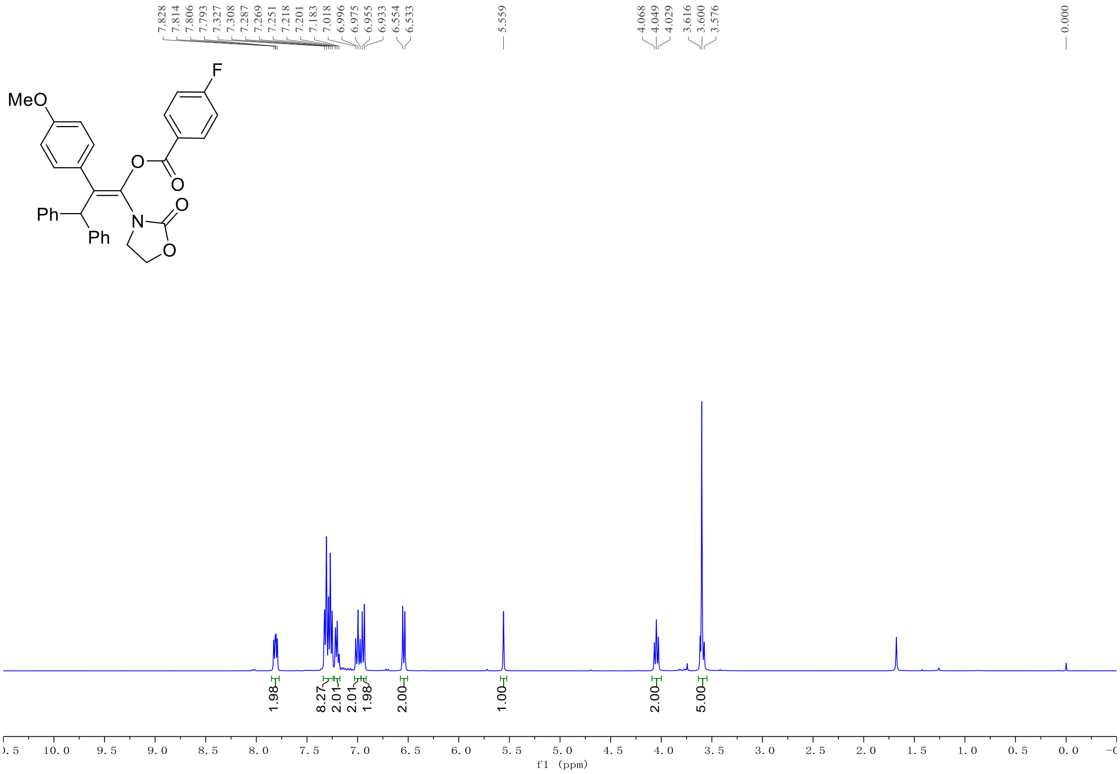

$^{13}$C NMR (100 MHz, $CDCl_3$)

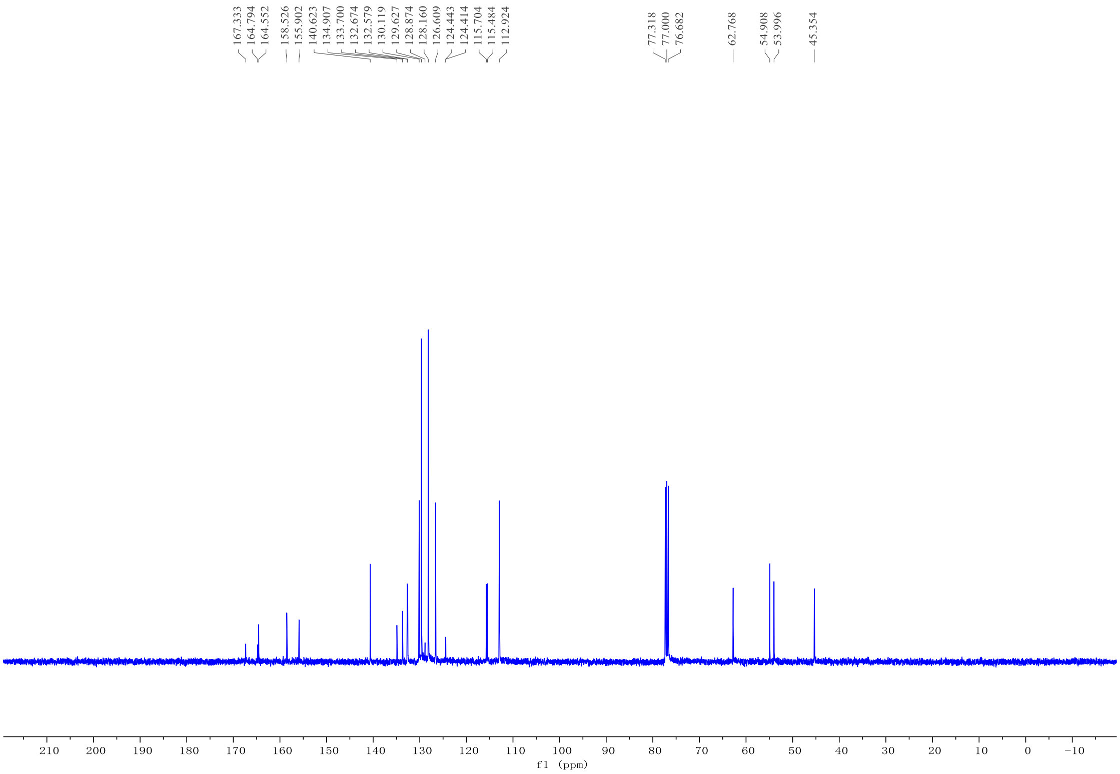

$^{19}$F NMR (376 MHz, $CDCl_3$)

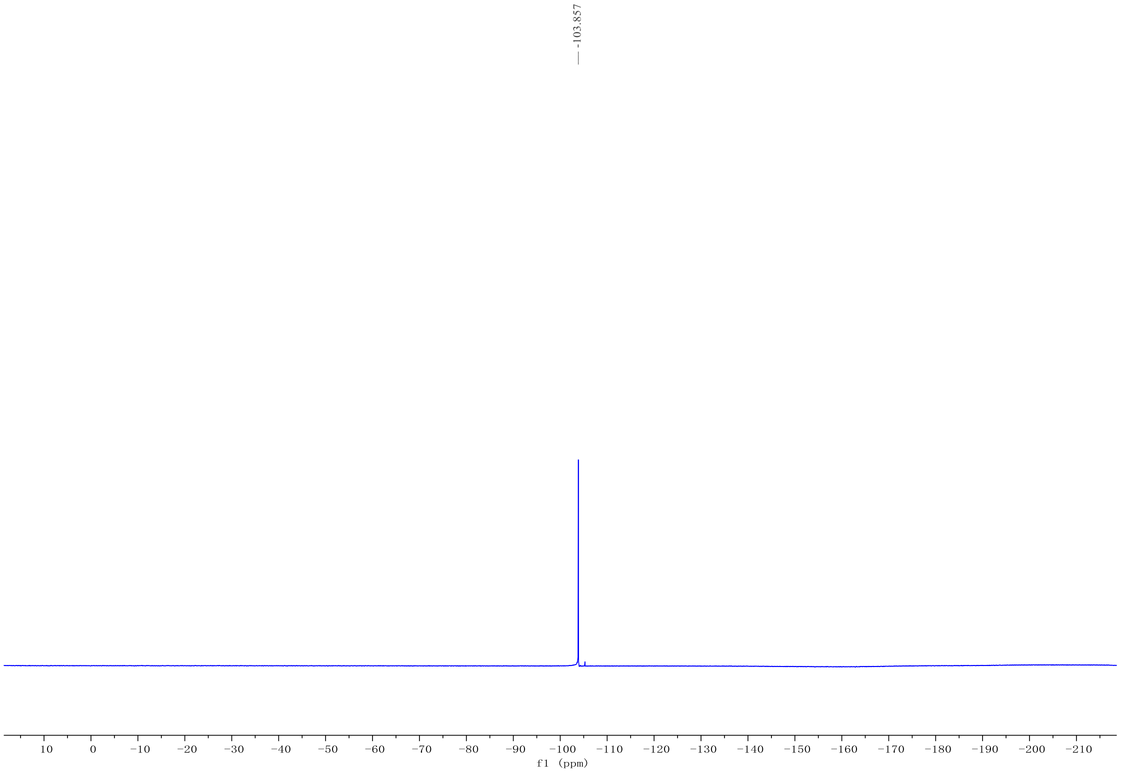


**$^{1}$H, $^{13}$C NMR and $^{19}$F NMR Spectra for Compound (*Z*)-3ia**:

$^{1}$H NMR (400 MHz, $CDCl_3$)

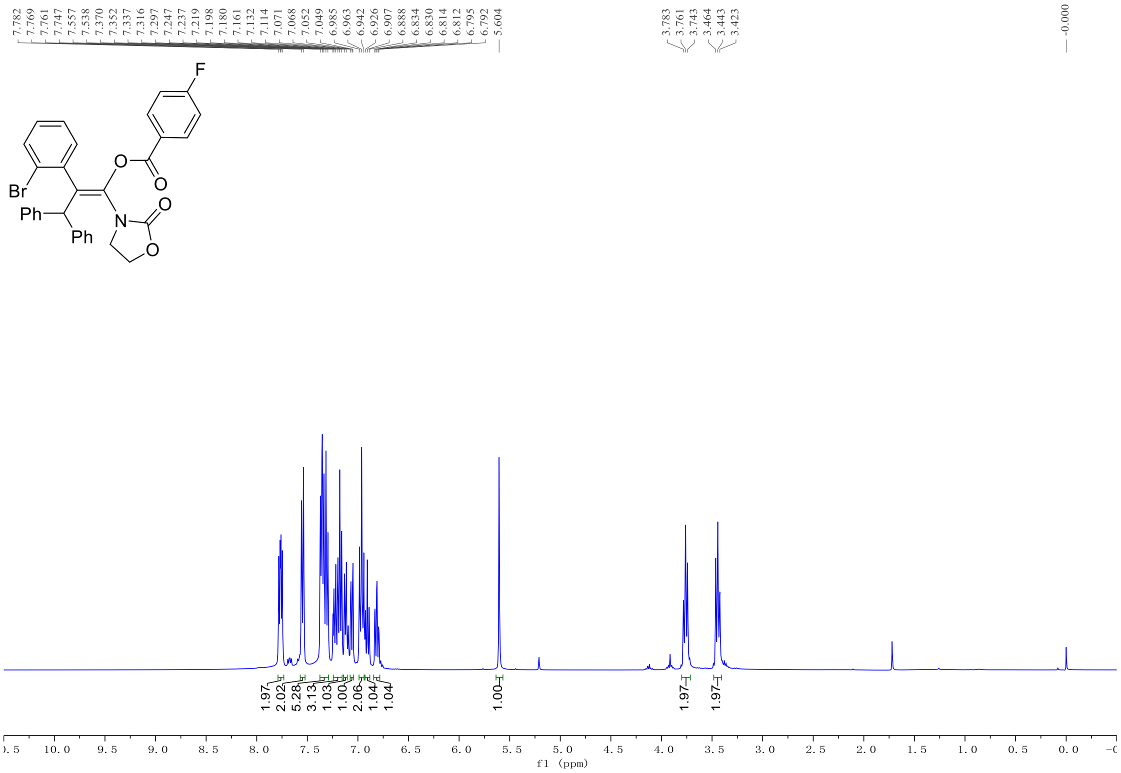

$^{13}C$ NMR (100 MHz, $CDCl_3$)

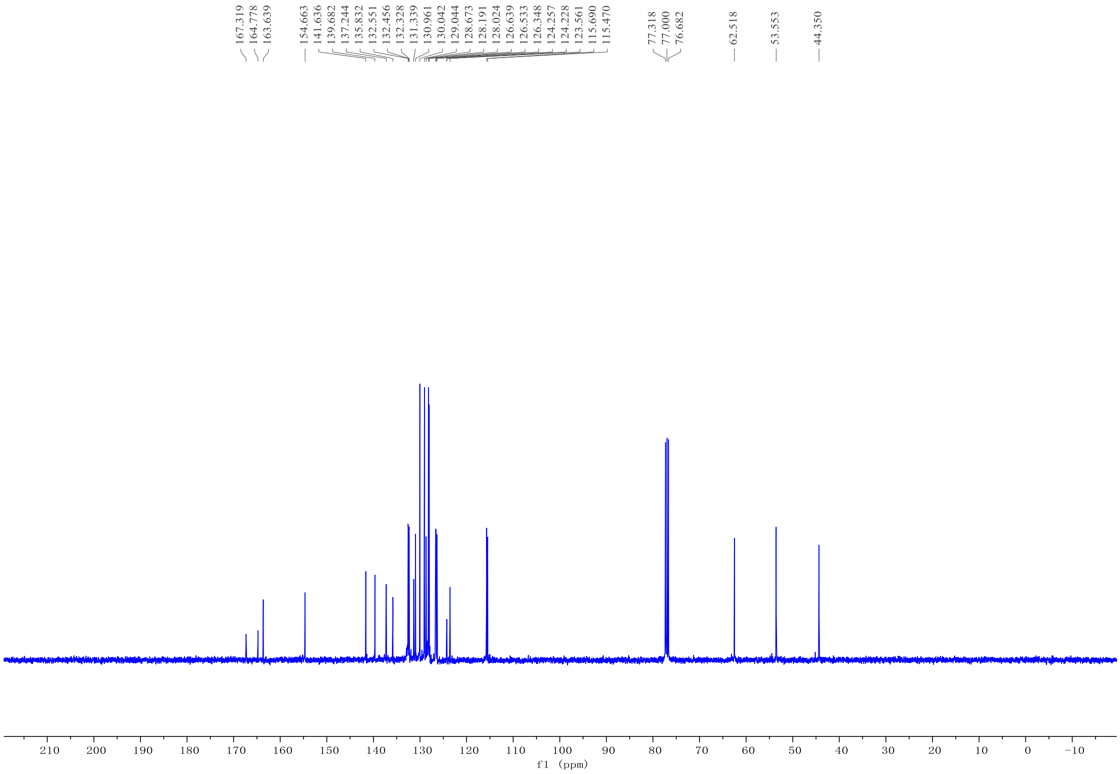


$^{19}F$ NMR (376 MHz, $CDCl_3$)

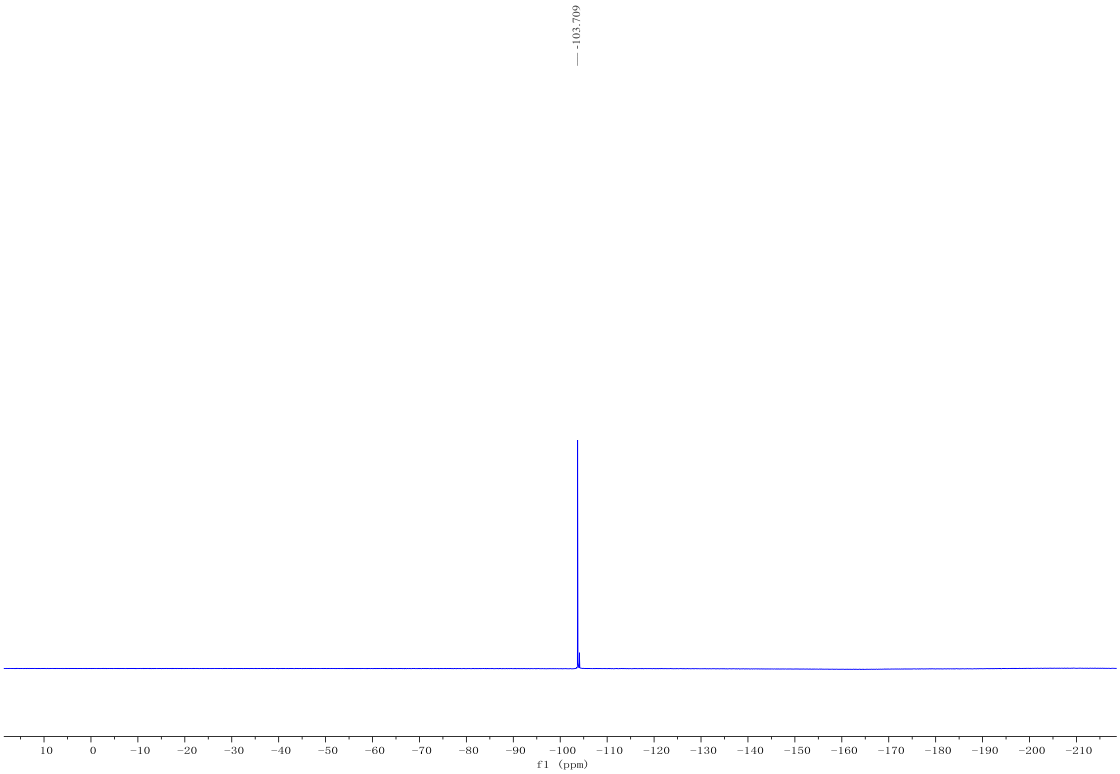

## $^{1}$H, $^{13}$C NMR and $^{19}$F NMR Spectra for Compound (*Z*)-3ja:

$^{1}$H NMR (400 MHz, $CDCl_3$)

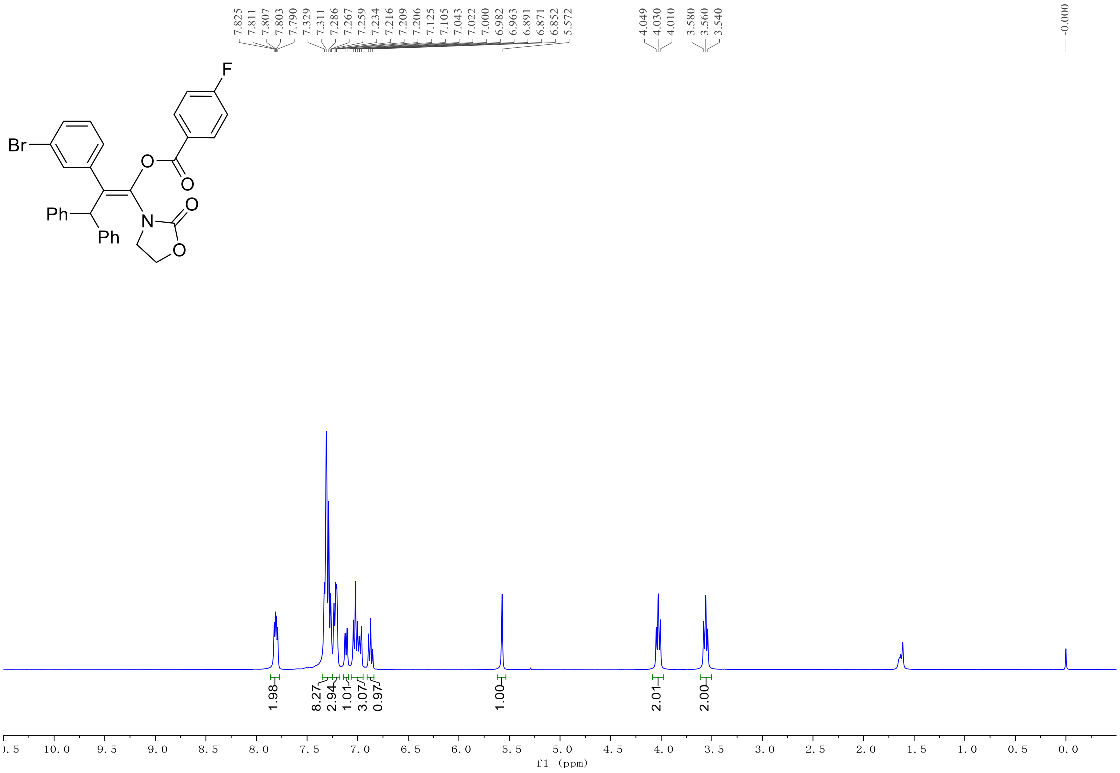

$^{13}$C NMR (100 MHz, $CDCl_3$)

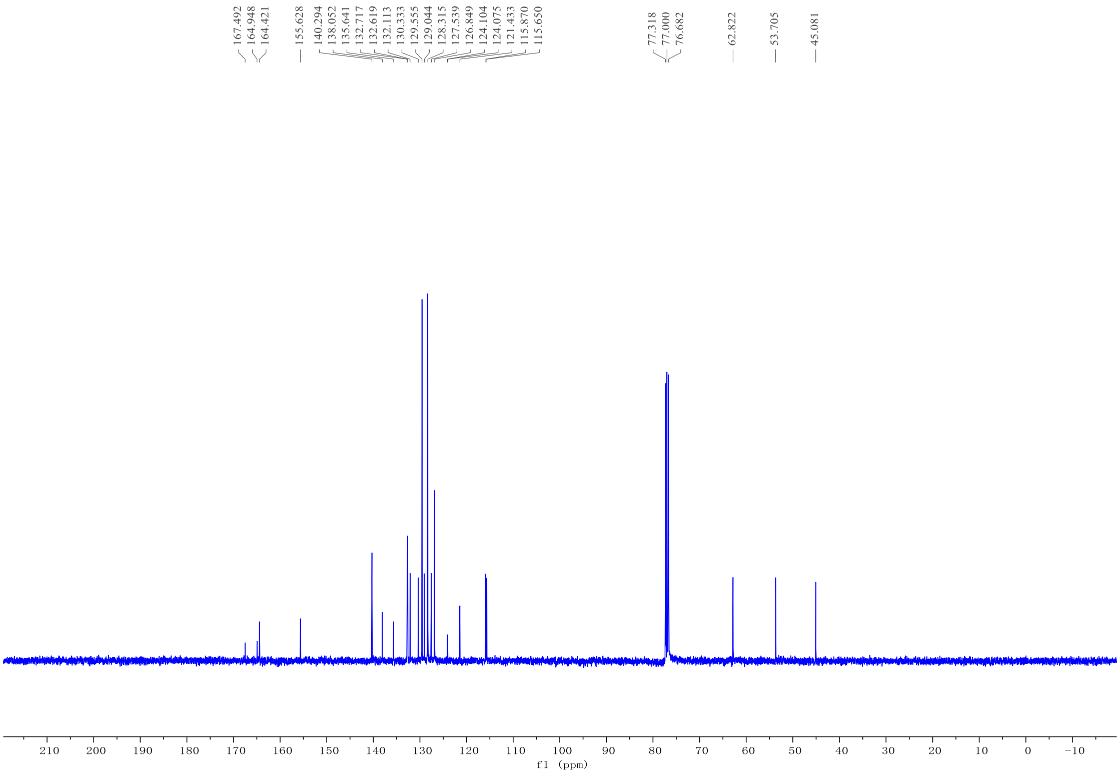

$^{19}F$ NMR (376 MHz, $CDCl_3$)

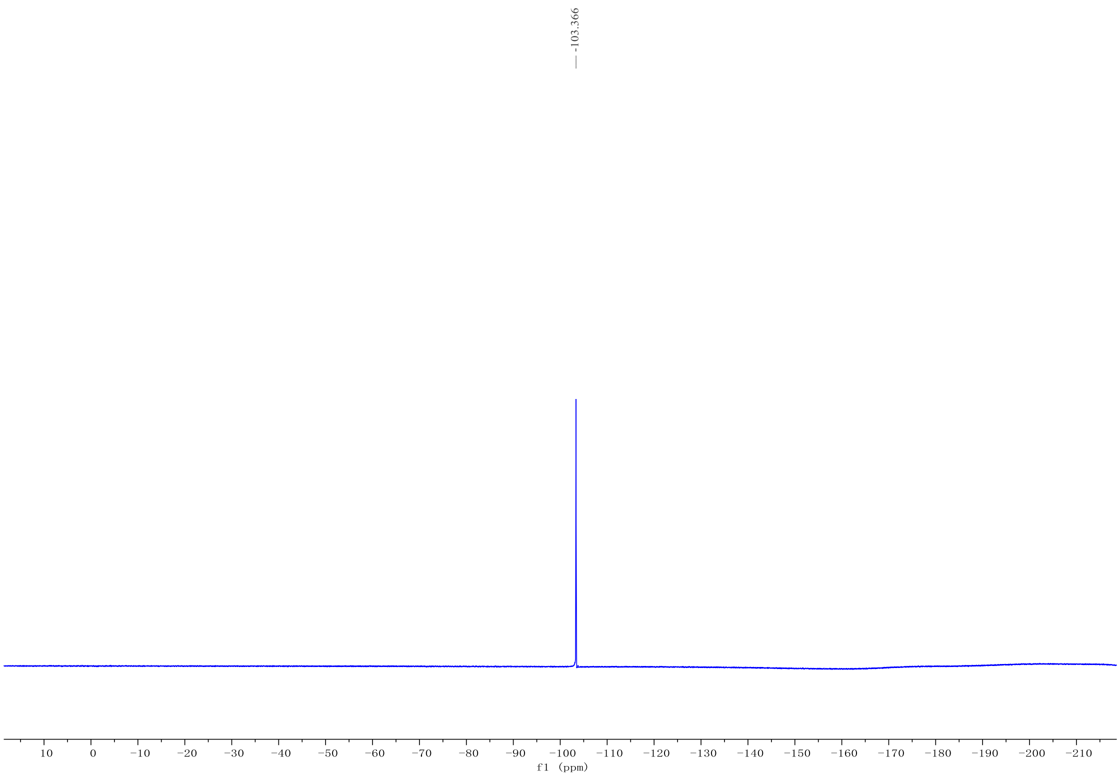

**$^{1}H$, $^{13}C$ NMR and $^{19}F$ NMR Spectra for Compound (*Z*)-3ka**:

$^{1}H$ NMR (400 MHz, $CDCl_3$)

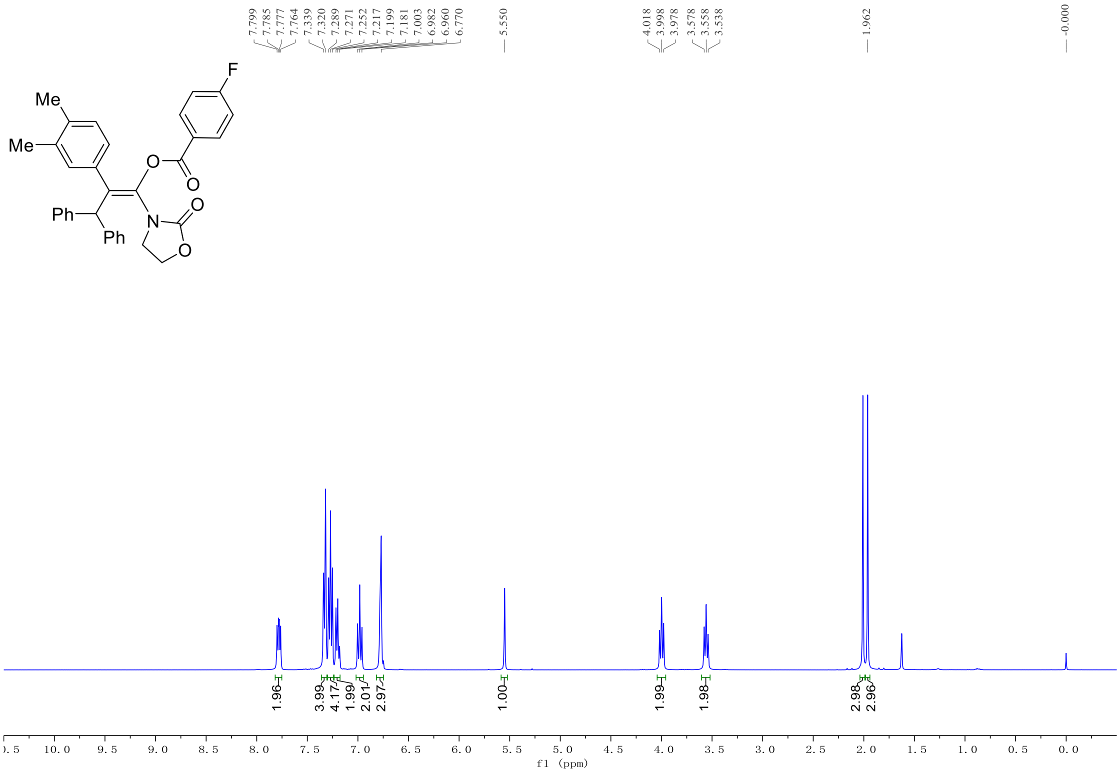

$^{13}C$ NMR (100 MHz, $CDCl_3$)

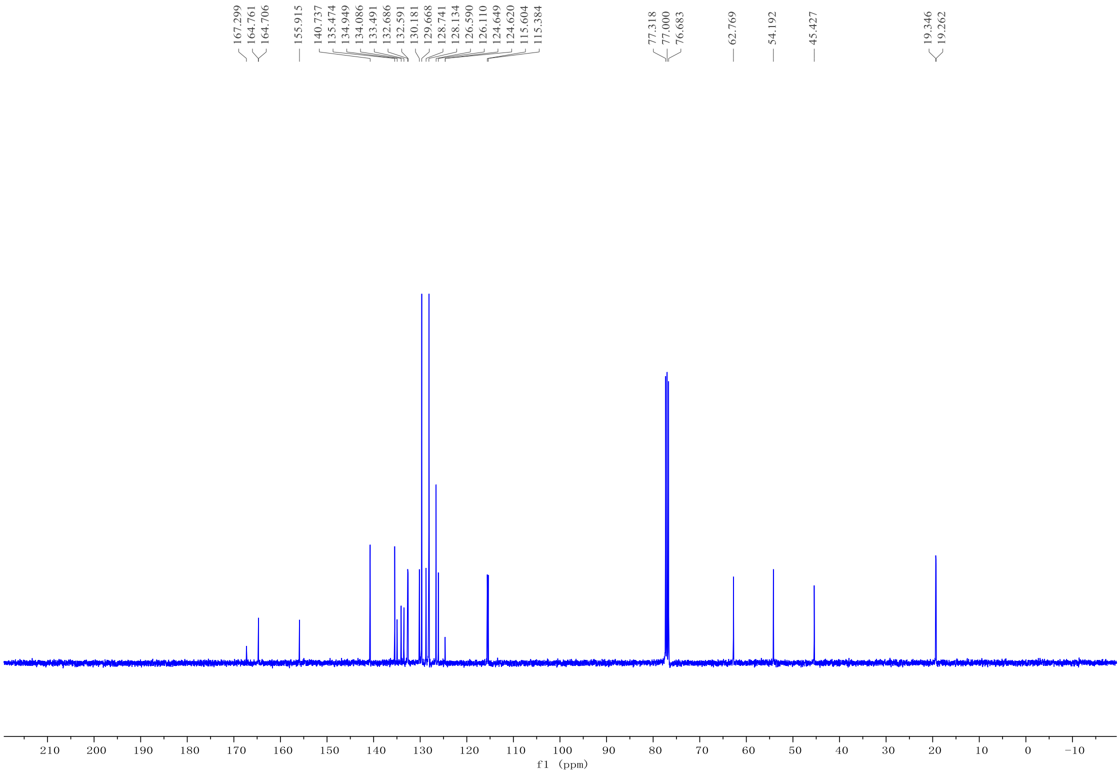


$^{19}F$ NMR (376 MHz, $CDCl_3$)

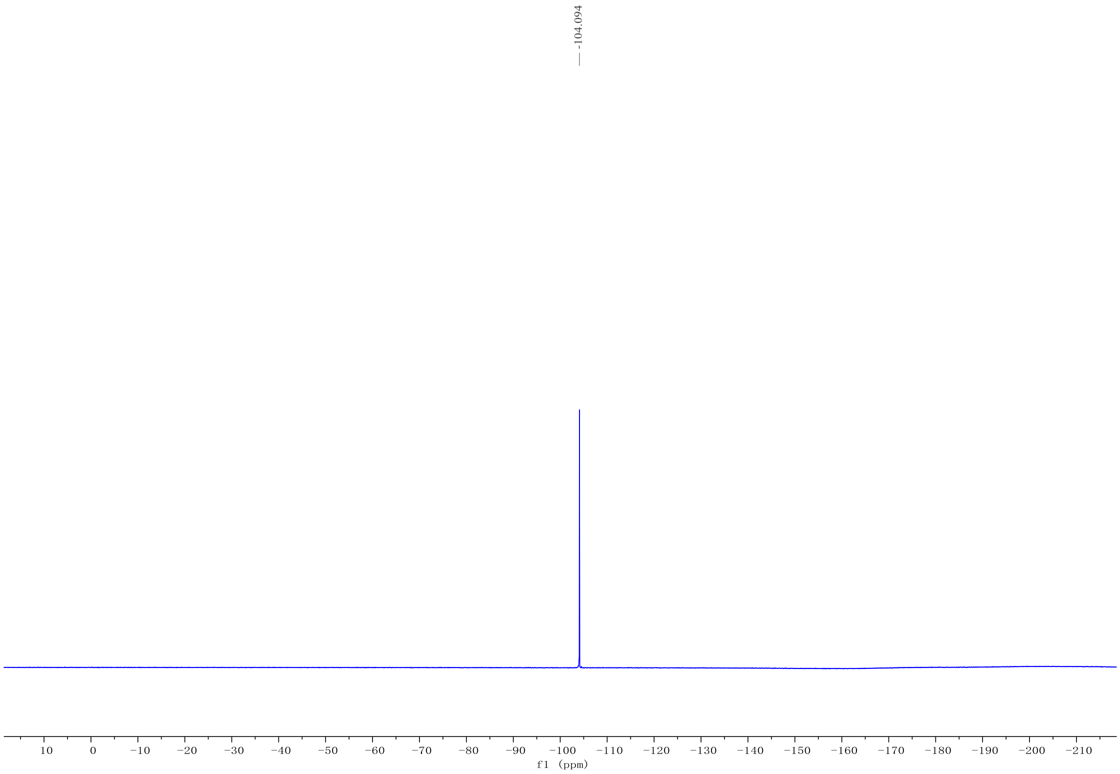

## **$^{1}$H, $^{13}$C NMR and $^{19}$F NMR Spectra for Compound (*Z*/*E*)-3la**:

$^{1}$H NMR (400 MHz, $CDCl_3$)

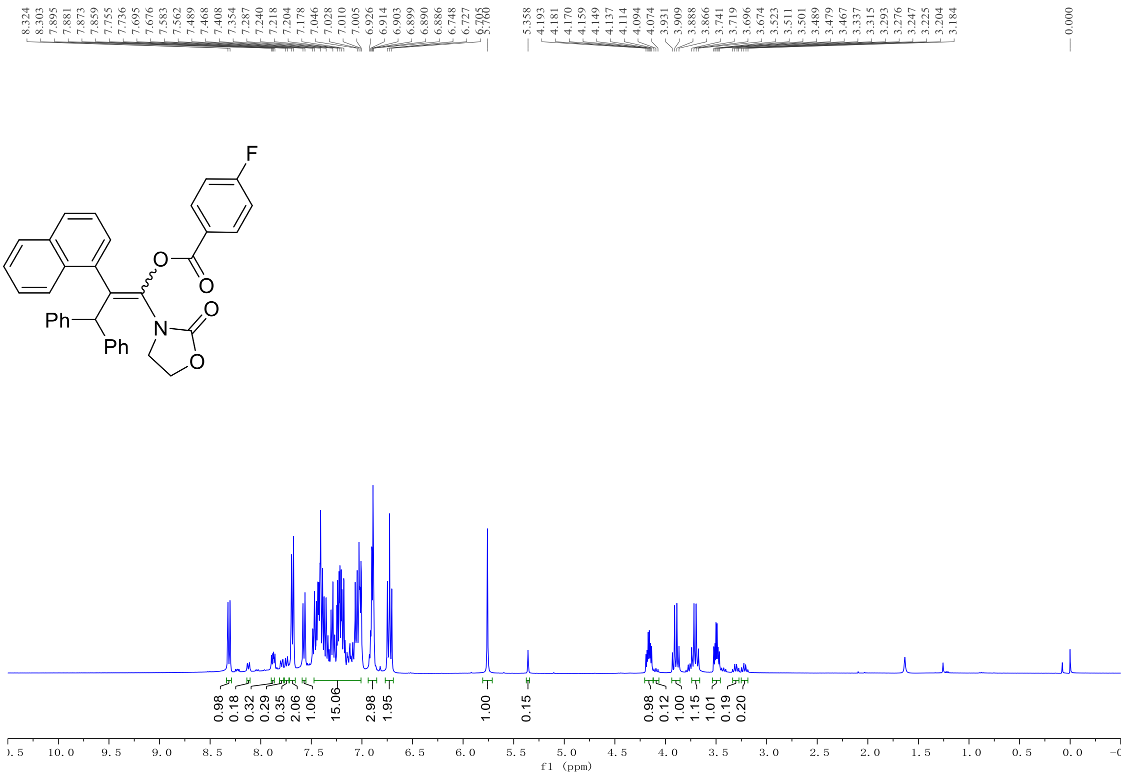


$^{13}$C NMR (100 MHz, $CDCl_3$)

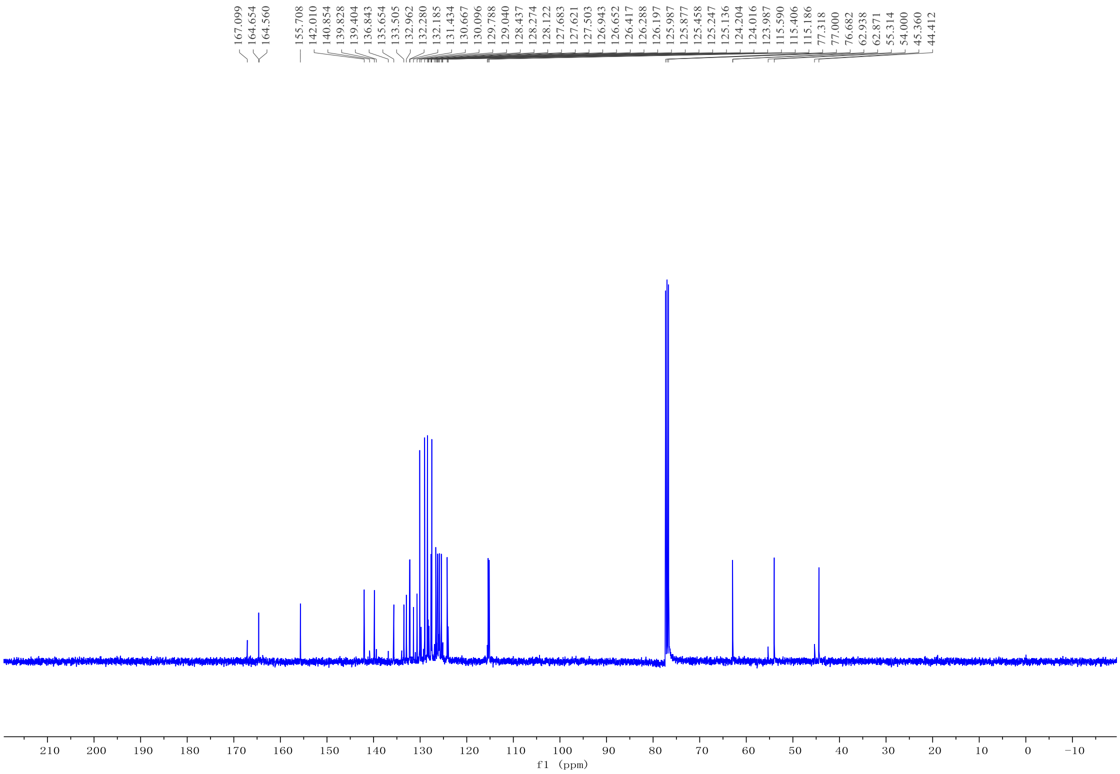

$^{19}$F NMR (376 MHz, $CDCl_3$)

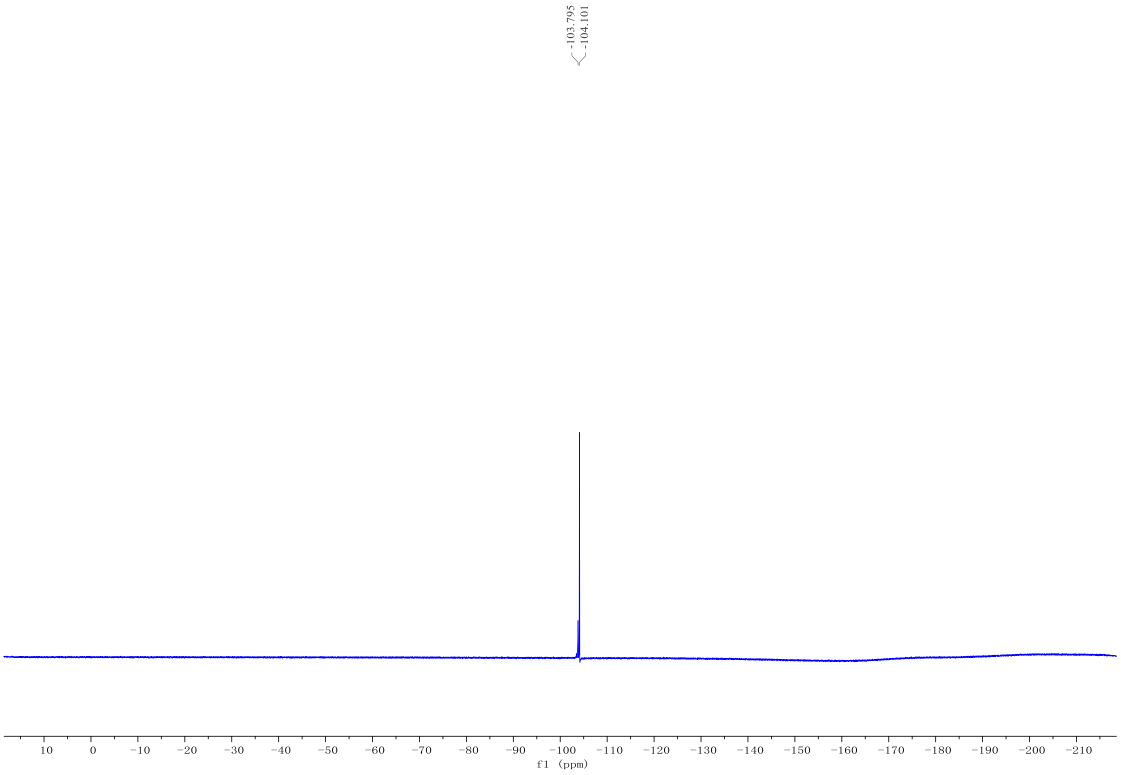


## $^{1}$H, $^{13}$C NMR and $^{19}$F NMR Spectra for Compound (*Z*)-3ma:

$^{1}$H NMR (400 MHz, $CDCl_3$)

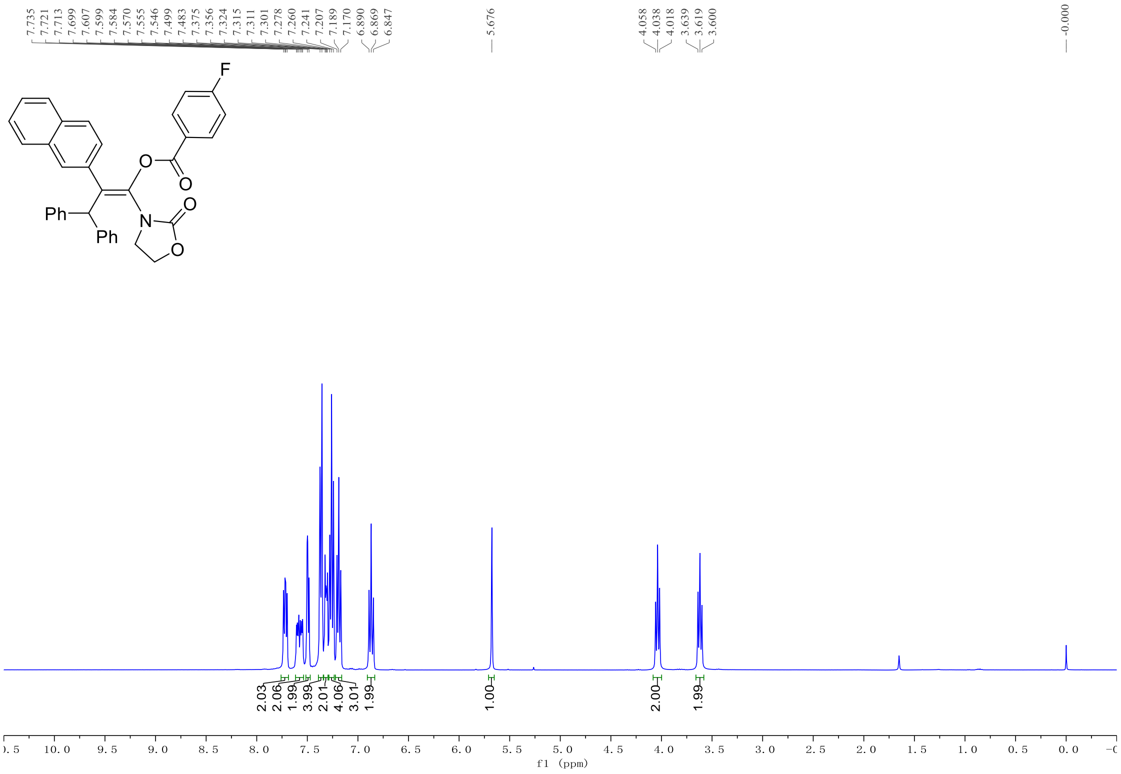

$^{13}C$ NMR (100 MHz, $CDCl_3$)

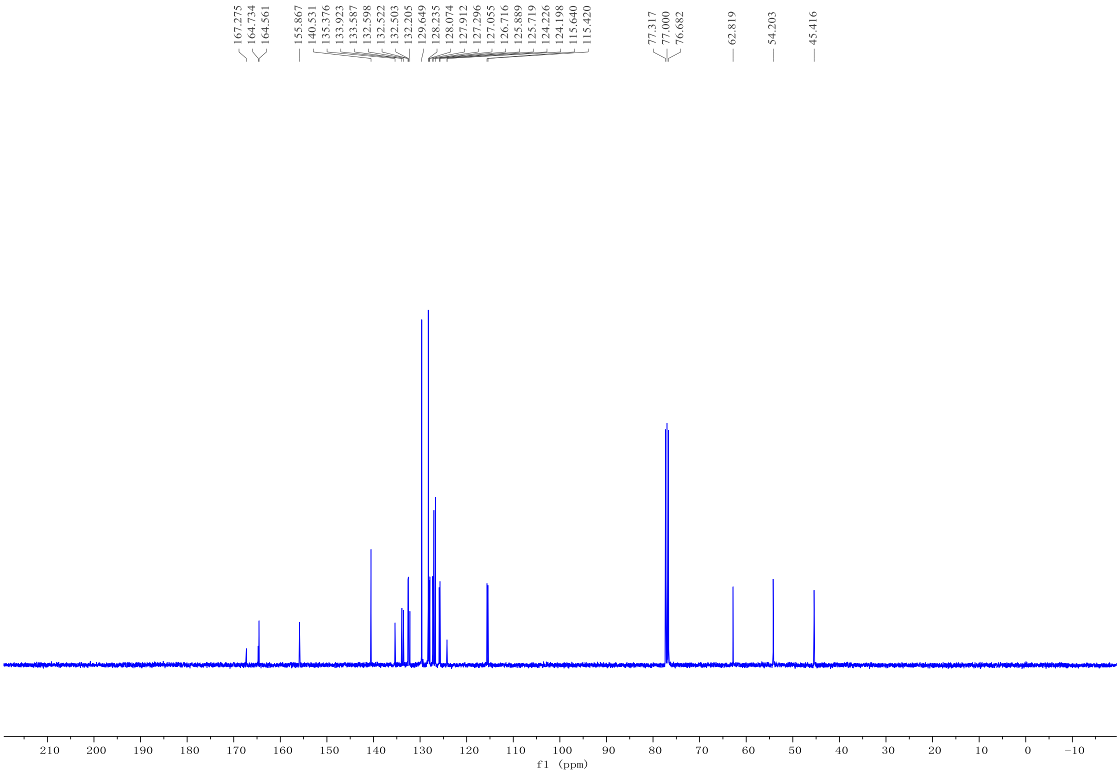


$^{19}F$ NMR (376 MHz, $CDCl_3$)

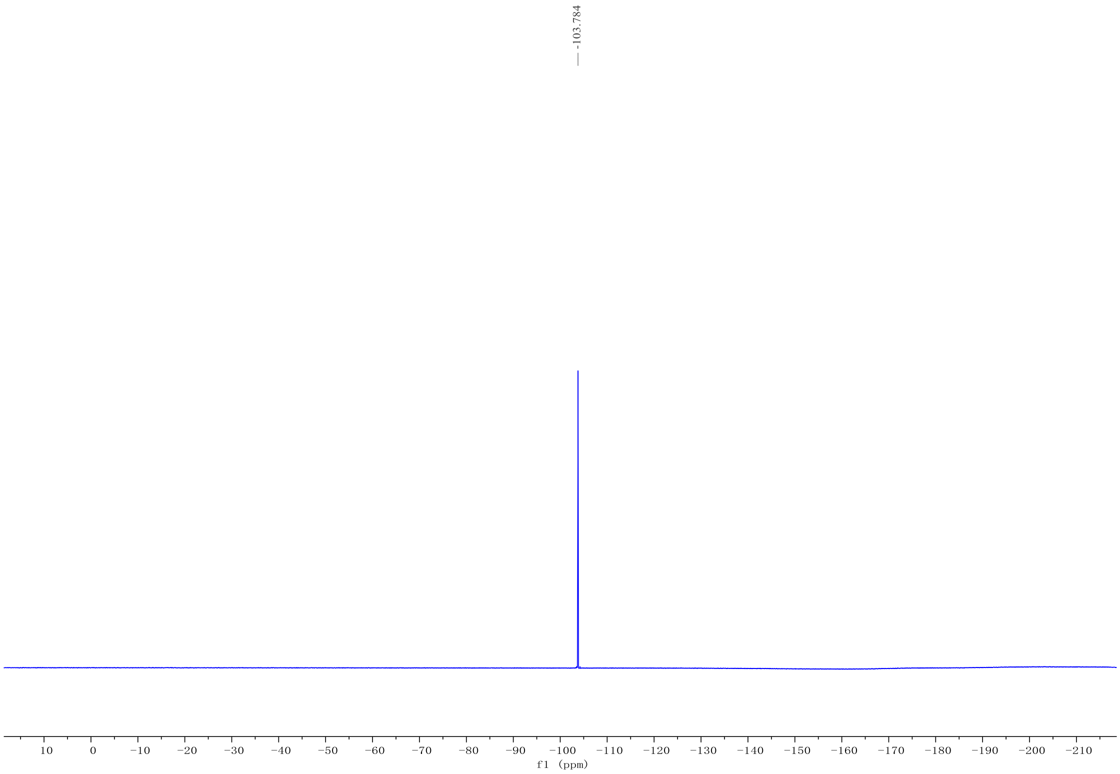

**$^{1}$H, $^{13}$C NMR and $^{19}$F NMR Spectra for Compound (*E*)-3na**:

$^{1}$H NMR (400 MHz, $CDCl_3$)

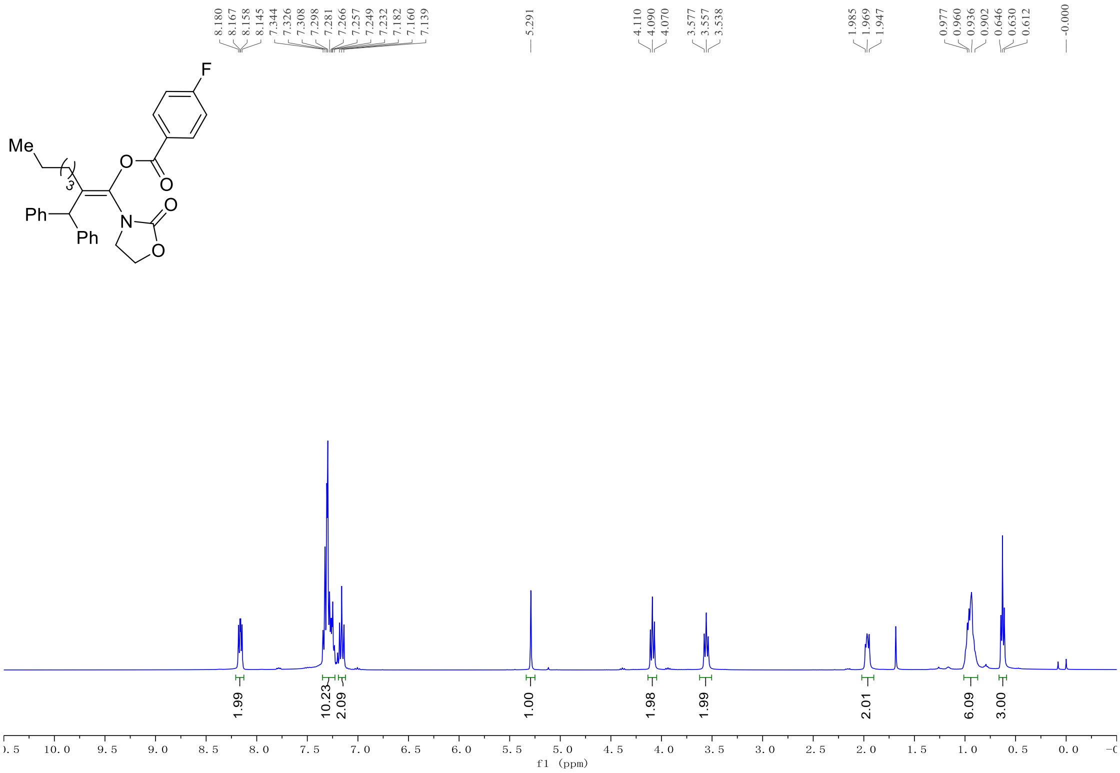

$^{13}$C NMR (100 MHz, $CDCl_3$)

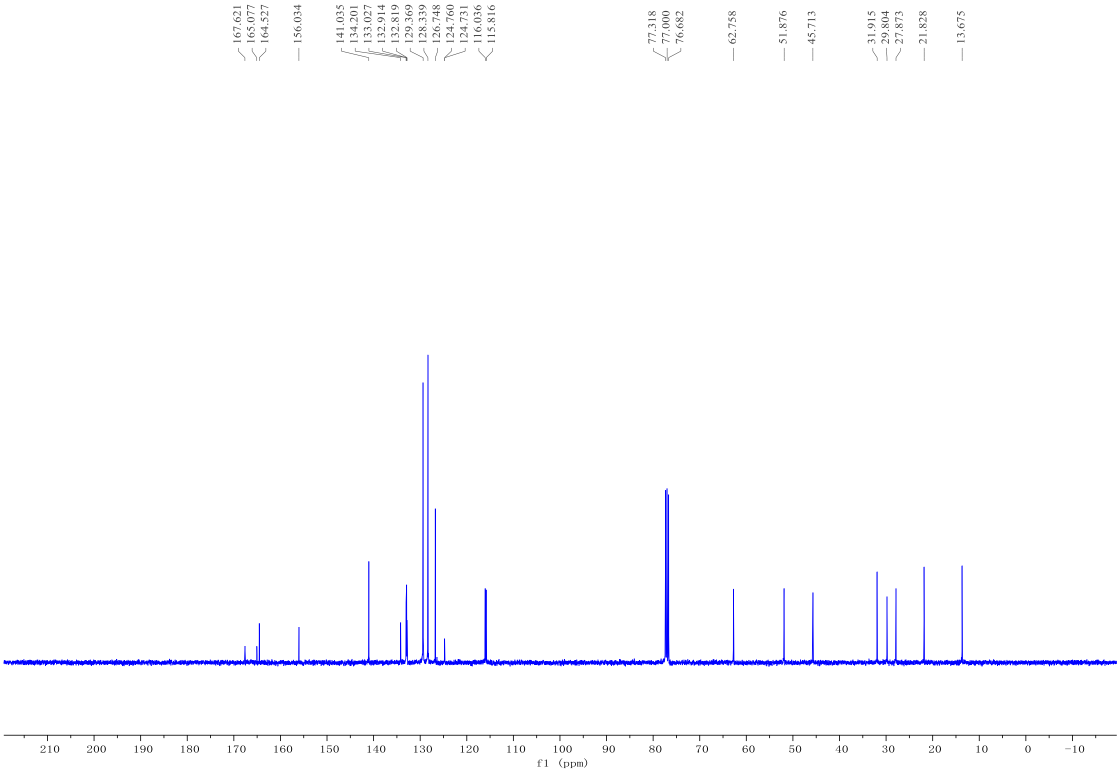

$^{19}$F NMR (376 MHz, $CDCl_3$)

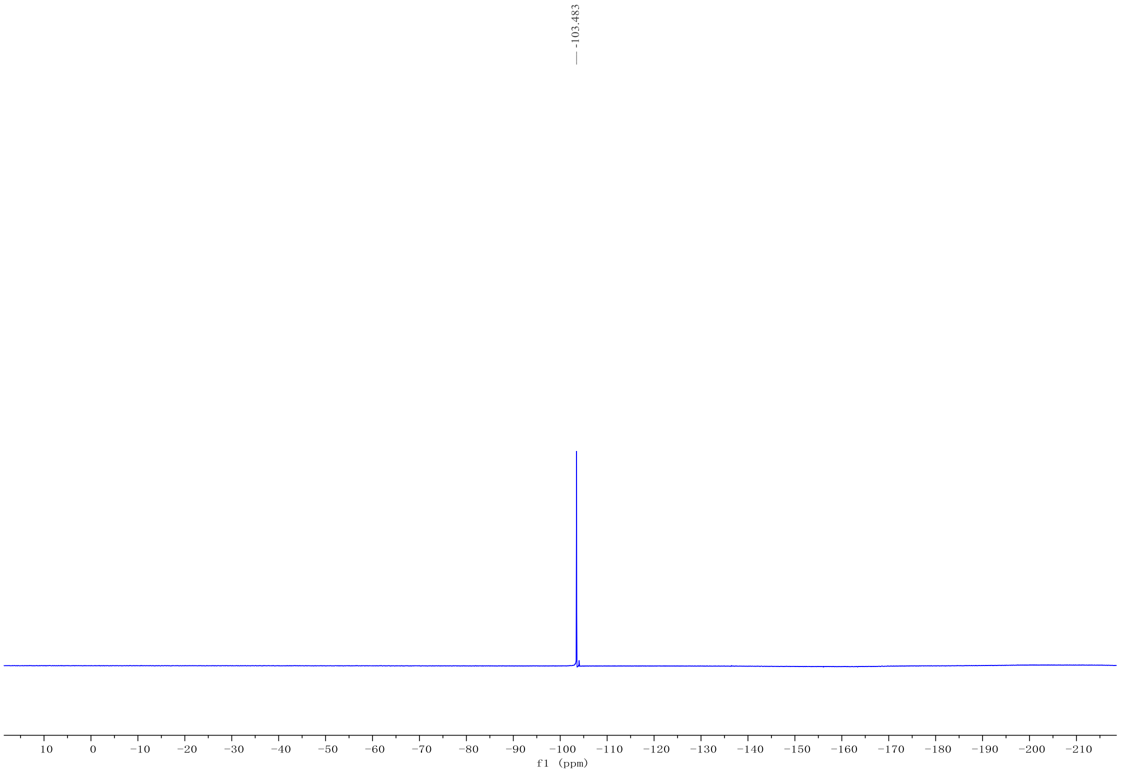


## $^{1}$H, $^{13}$C NMR and $^{19}$F NMR Spectra for Compound (*E*)-3oa:

$^{1}$H NMR (400 MHz, $CDCl_3$)

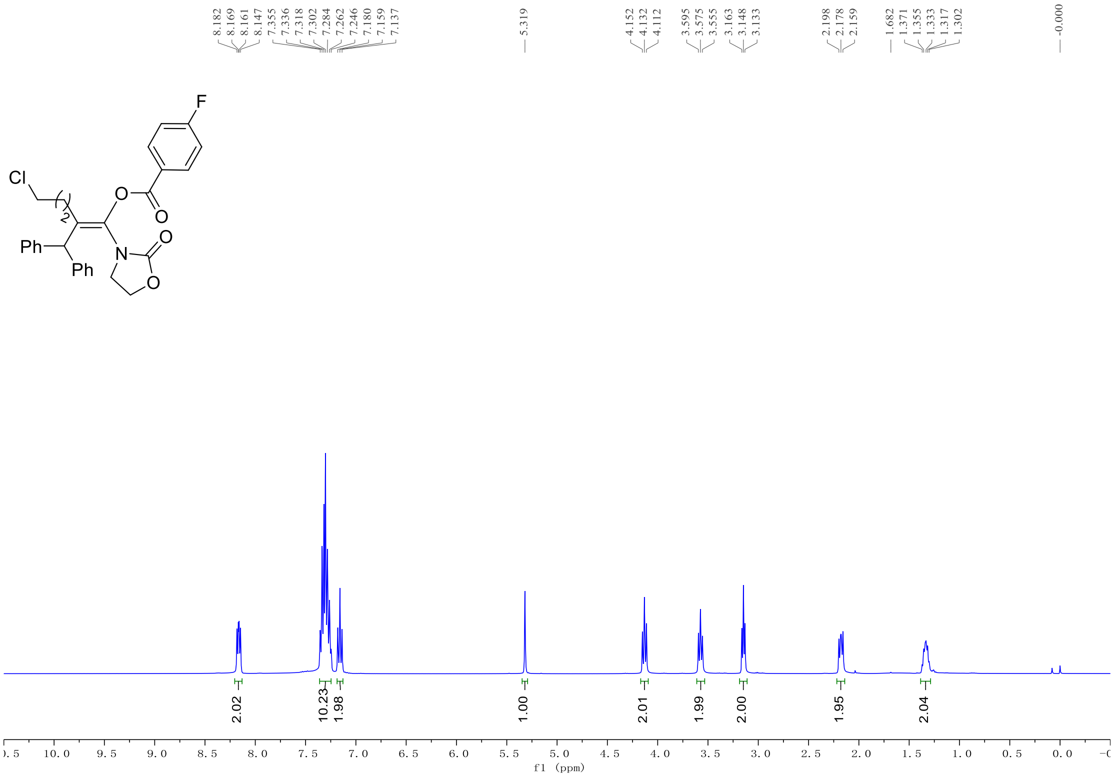

$^{13}$C NMR (100 MHz, $CDCl_3$)

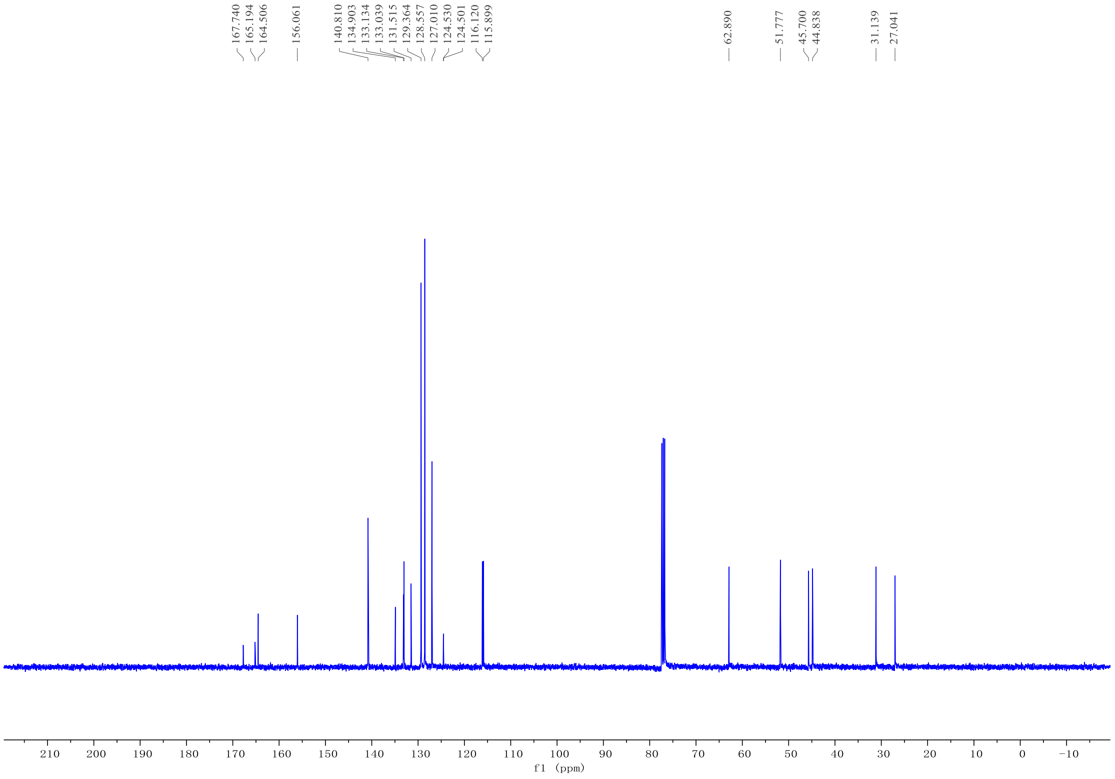


$^{19}$F NMR (376 MHz, $CDCl_3$)

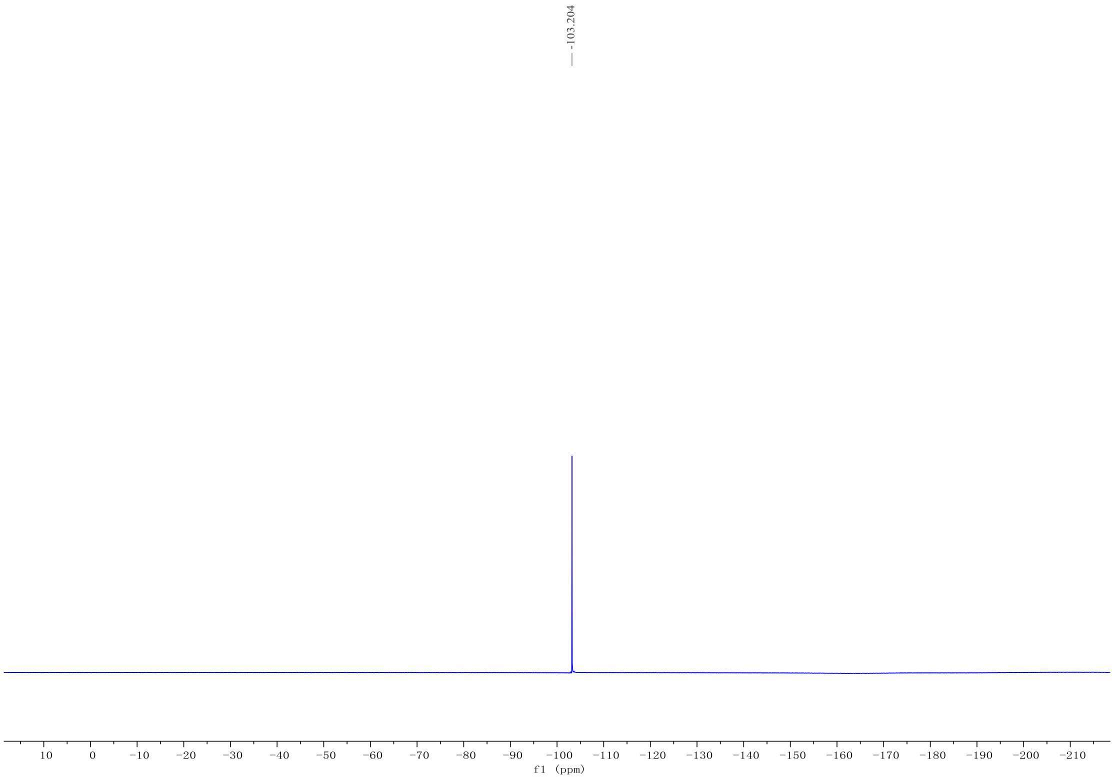

# $^{1}$H, $^{13}$C NMR and $^{19}$F NMR Spectra for Compound (*E*)-3pa:

## $^{1}$H NMR (400 MHz, $CDCl_3$)

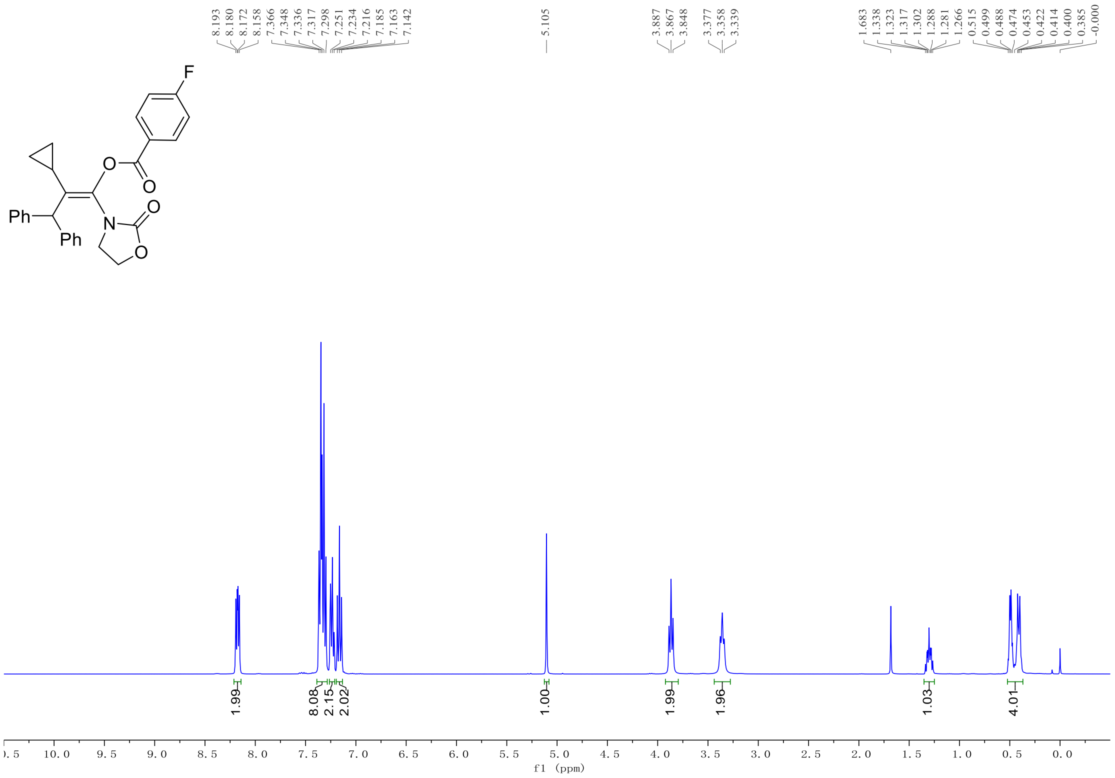


## $^{13}$C NMR (100 MHz, $CDCl_3$)

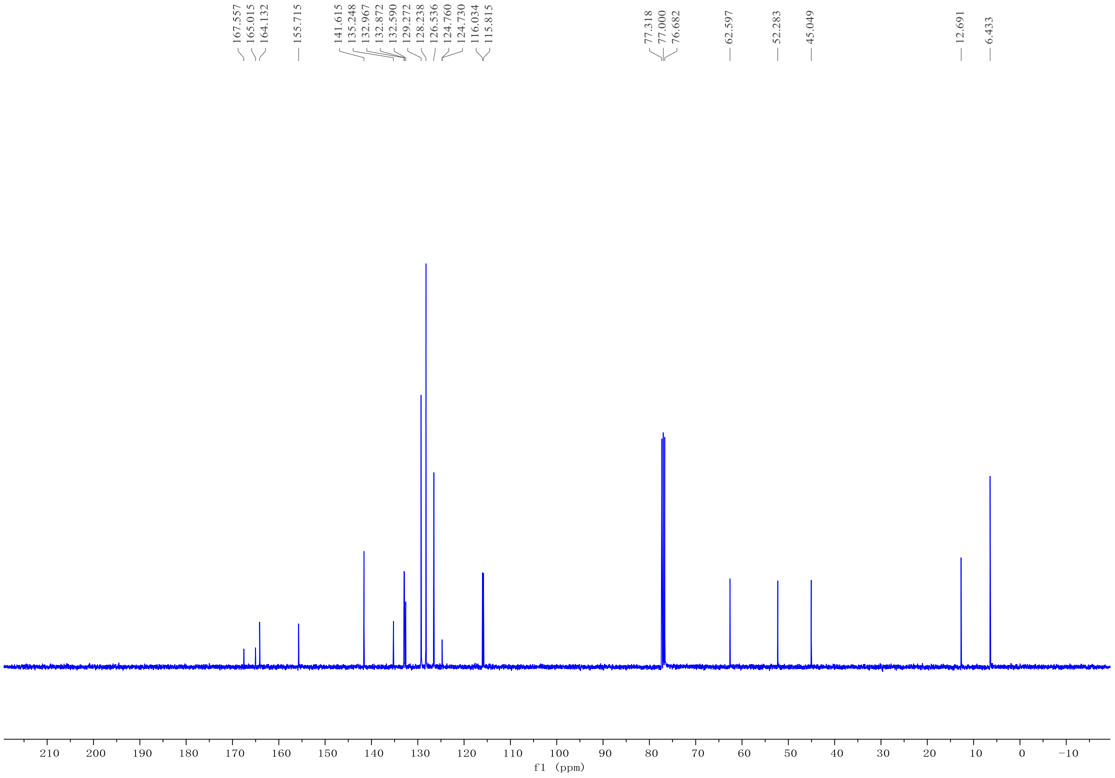

$^{19}F$ NMR (376 MHz, $CDCl_3$)

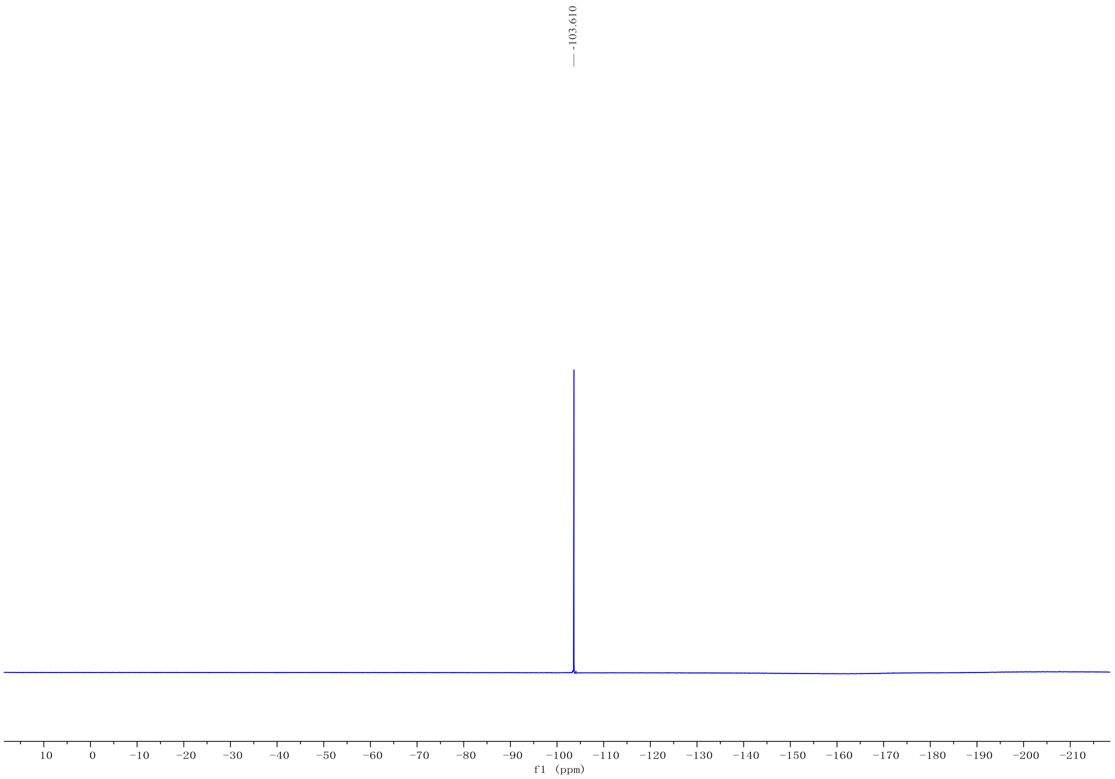


**$^{1}H$, $^{13}C$ NMR and $^{19}F$ NMR Spectra for Compound (*Z*/*E*)-3qa**:

$^{1}H$ NMR (400 MHz, $CDCl_3$)

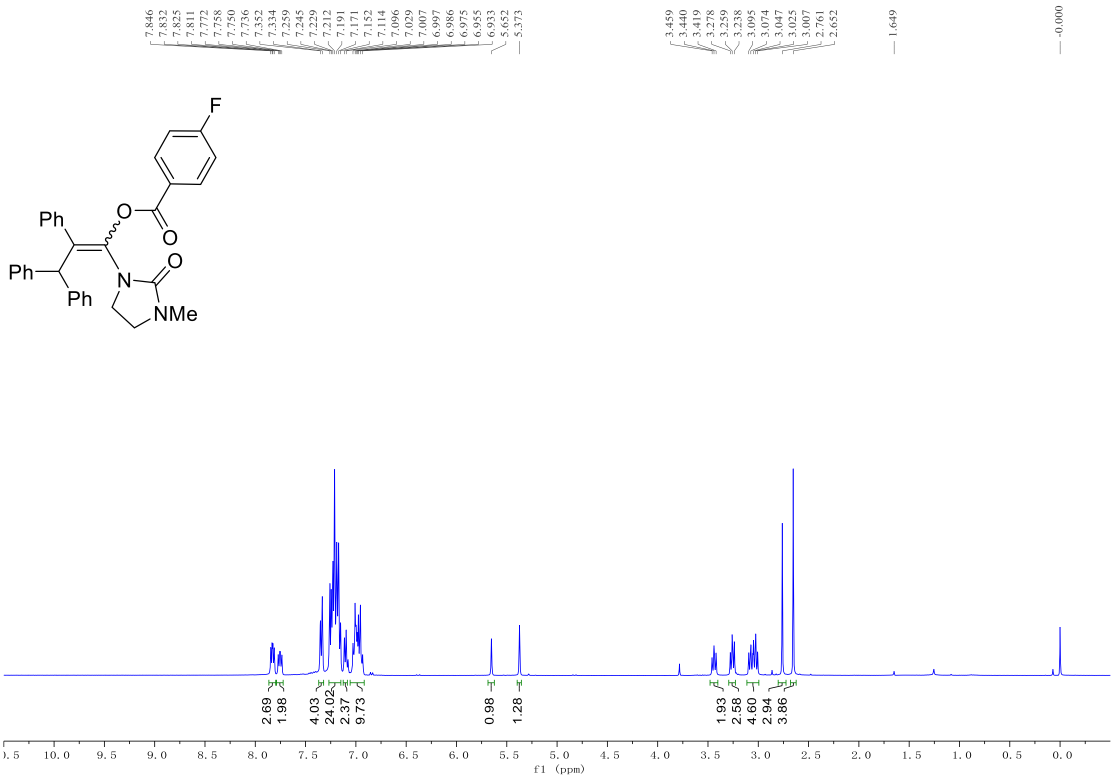

$^{13}$C NMR (100 MHz, CDCl$_3$)

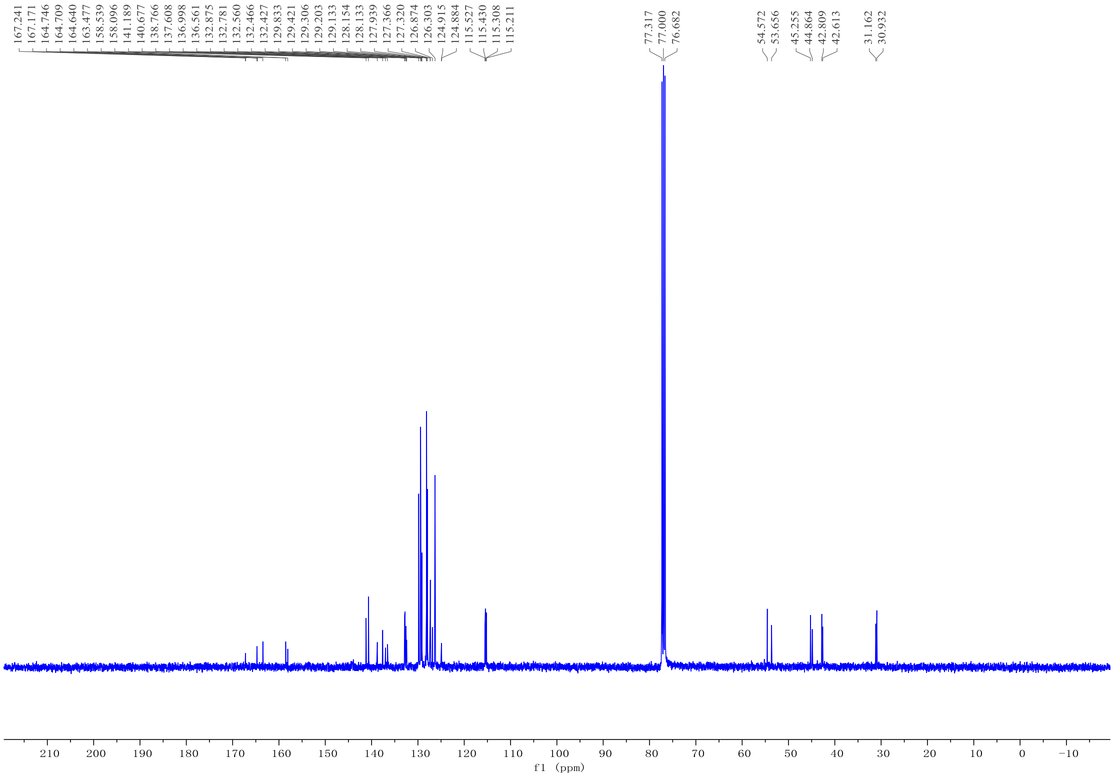


$^{19}$F NMR (376 MHz, CDCl$_3$)

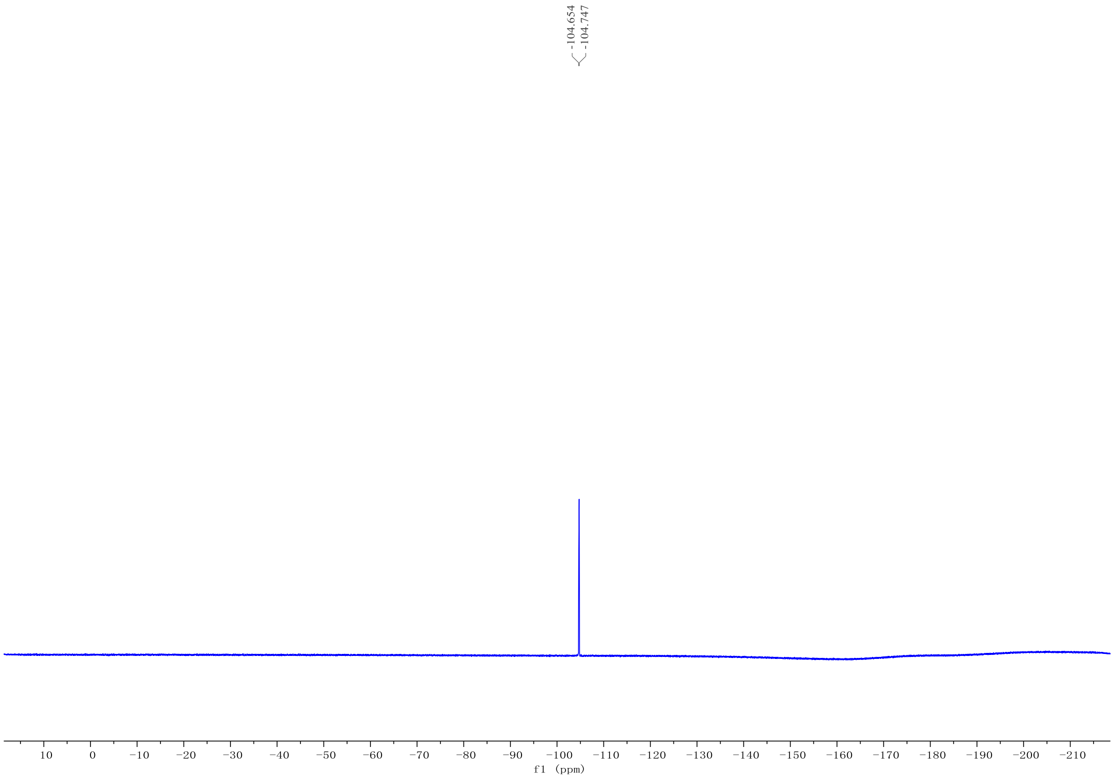

## $^{1}$H, $^{13}$C NMR and $^{19}$F NMR Spectra for Compound (*Z*)-3ra:

$^{1}$H NMR (400 MHz, $CDCl_3$)

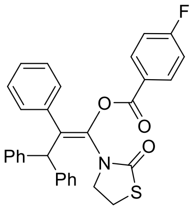


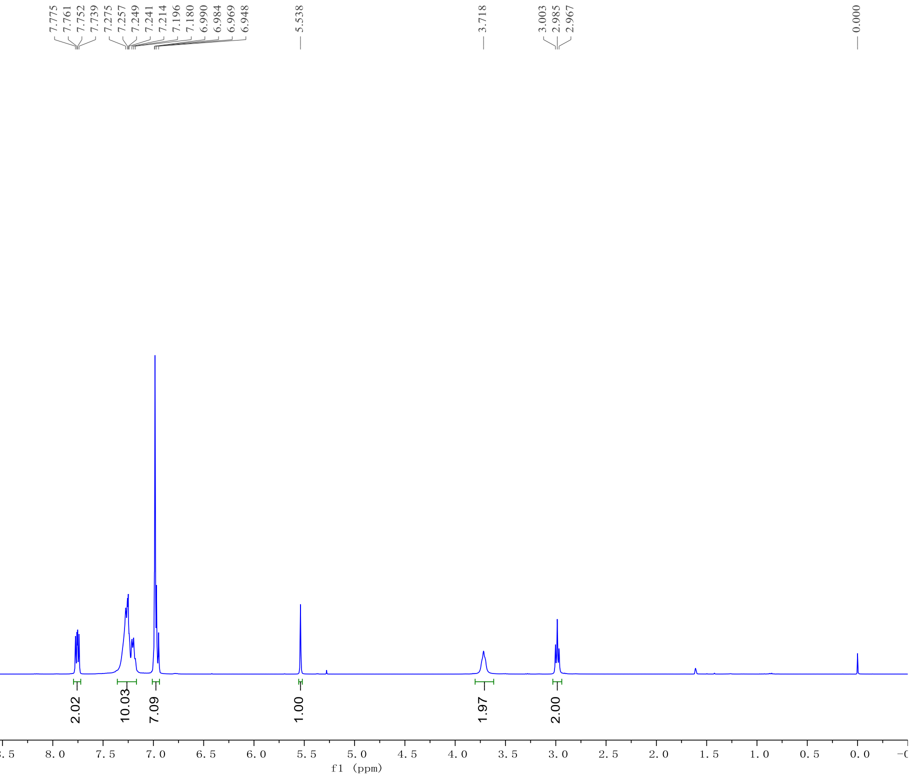


$^{13}$C NMR (100 MHz, $CDCl_3$)

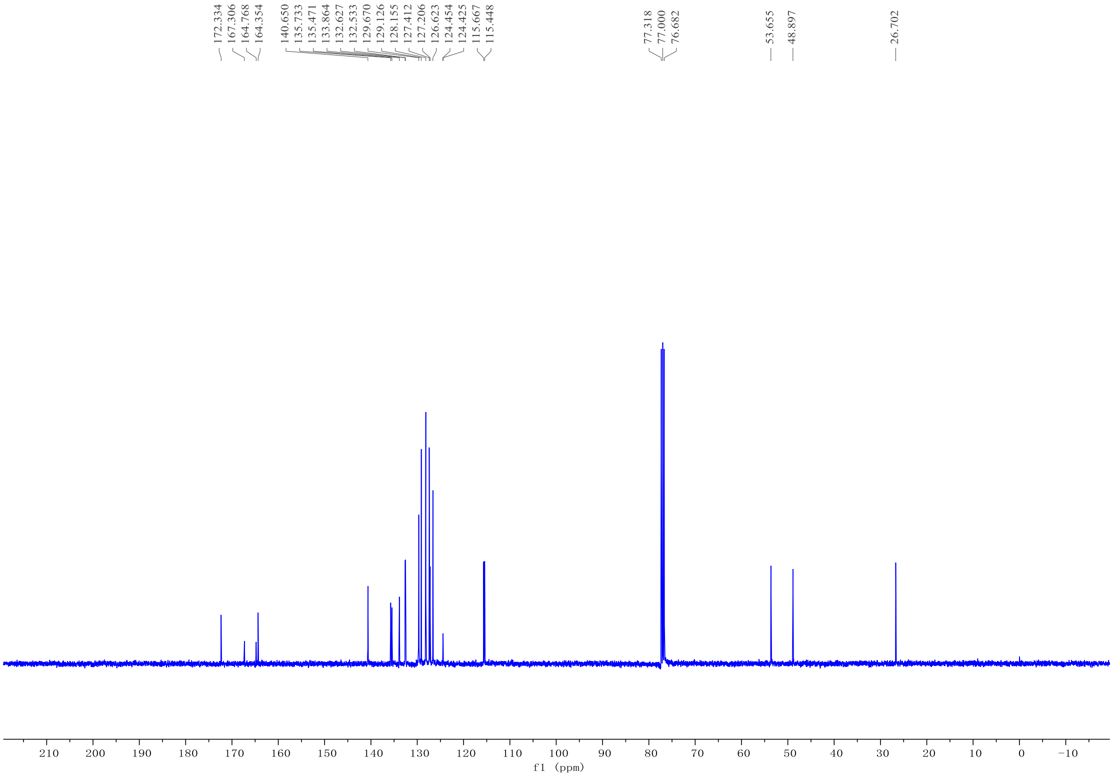

$^{19}$F NMR (376 MHz, $CDCl_3$)

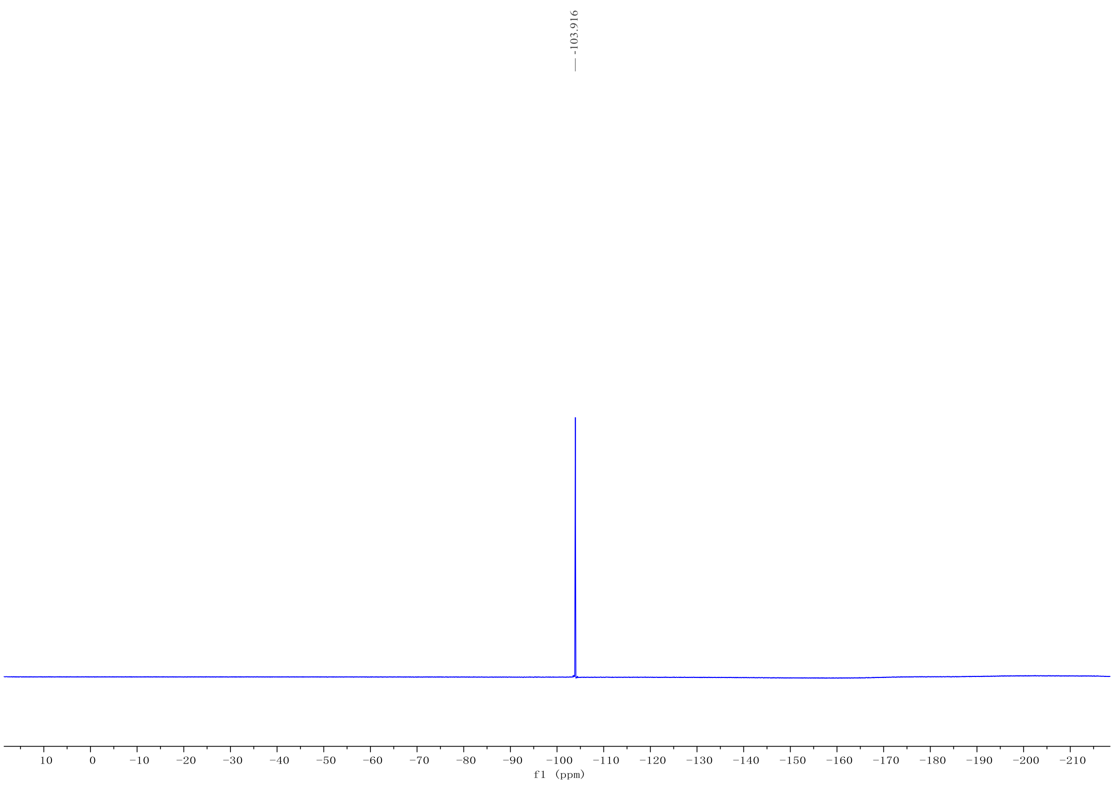


## $^{1}$H, $^{13}$C NMR and $^{19}$F NMR Spectra for Compound 4aa:

$^{1}$H NMR (400 MHz, $CDCl_3$)

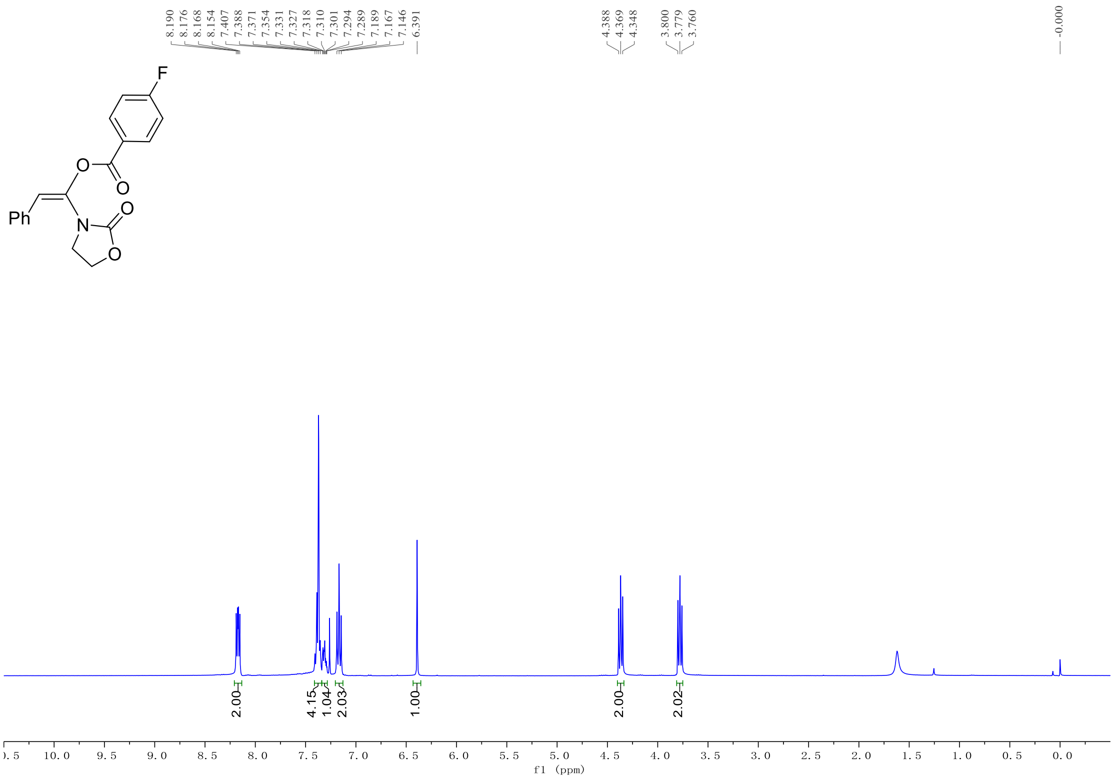

$^{13}C$ NMR (100 MHz, $CDCl_3$)

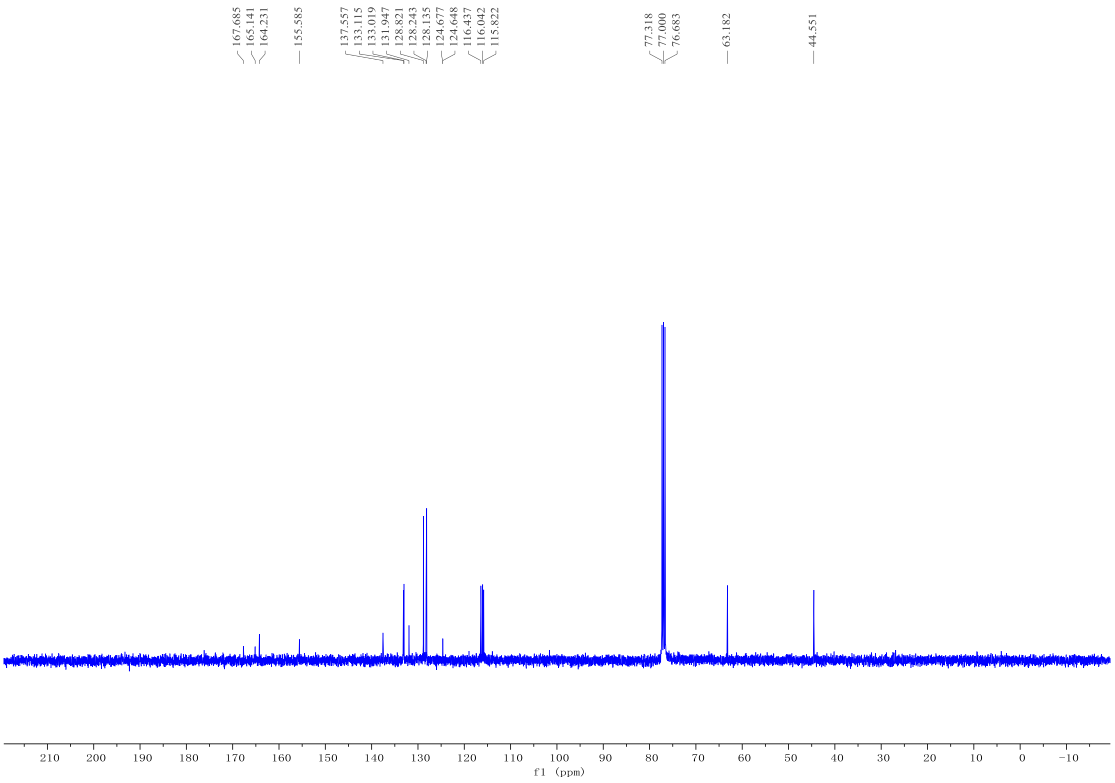

$^{19}F$ NMR (376 MHz, $CDCl_3$)

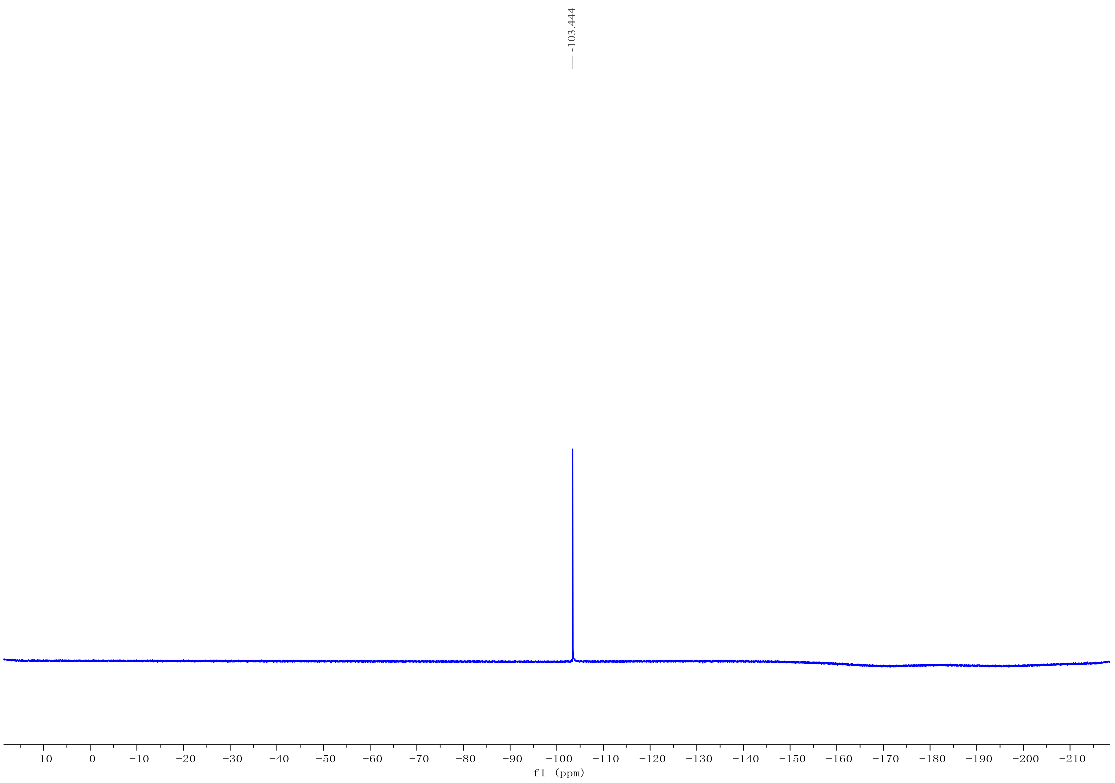

**$^{1}$H and $^{13}$C NMR Spectra for Compound (*E*)-6oa**:

$^{1}$H NMR (400 MHz, $CDCl_3$)

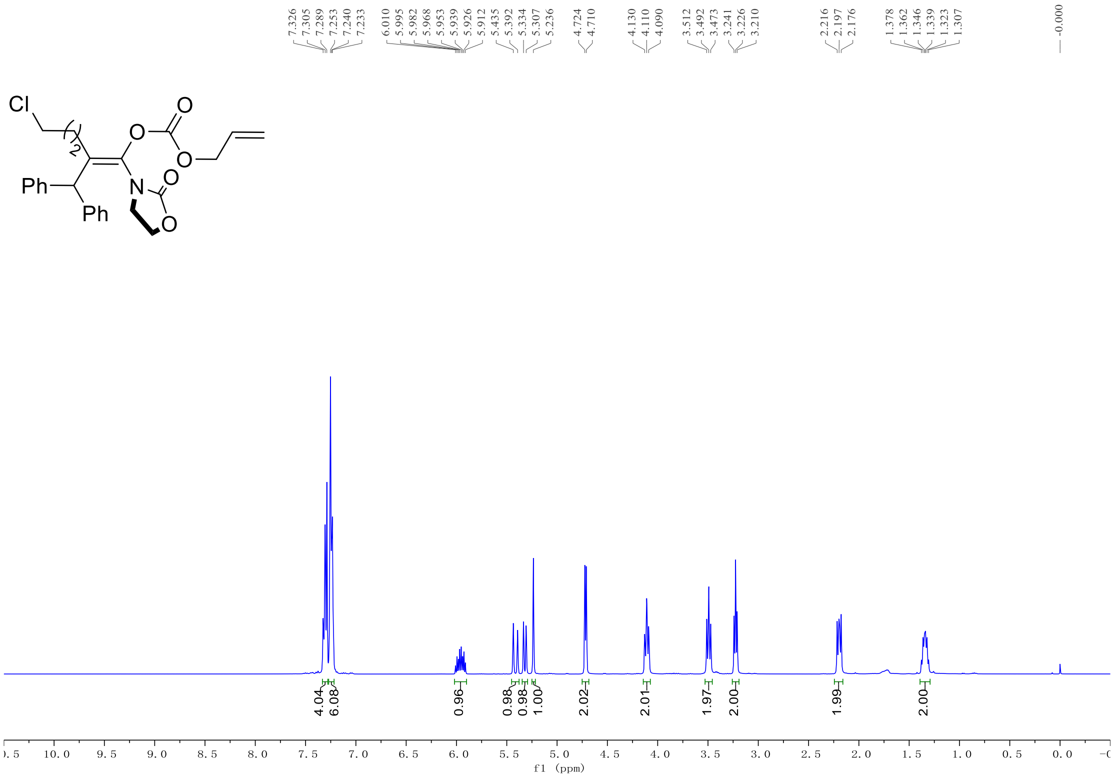


$^{13}$C NMR (100 MHz, $CDCl_3$)

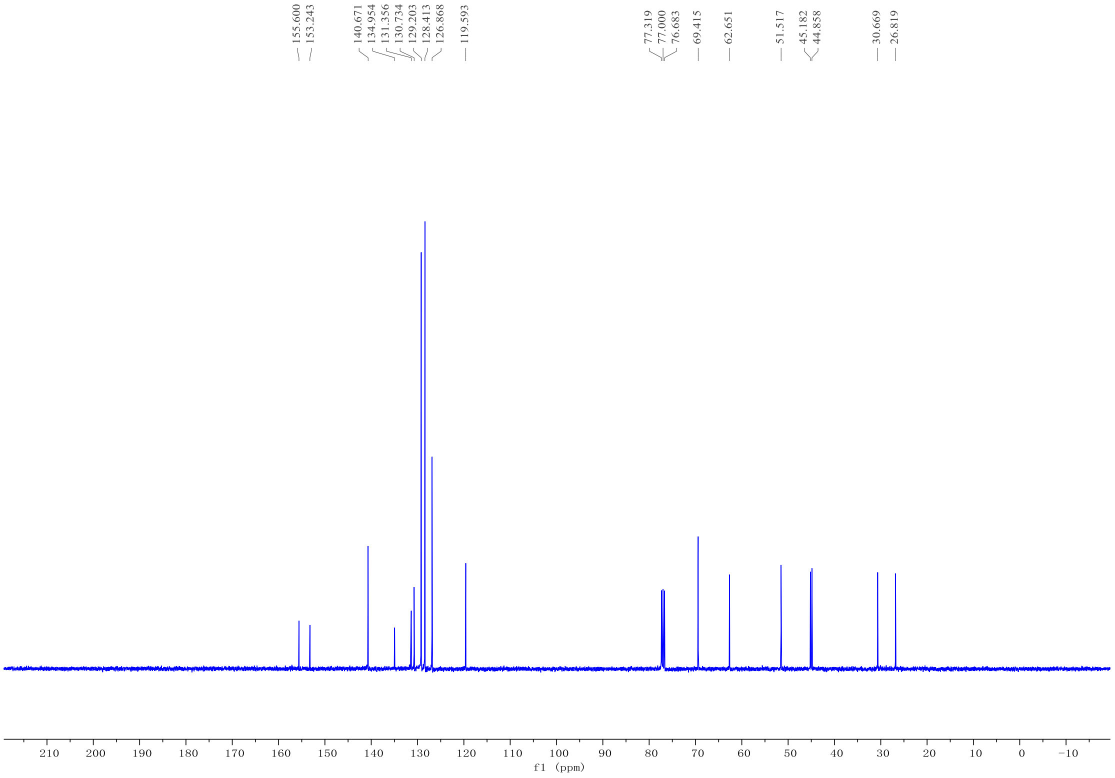

**$^{1}$H and $^{13}$C NMR Spectra for Compound (*E*)-6ob**:

$^{1}$H NMR (400 MHz, $CDCl_3$)

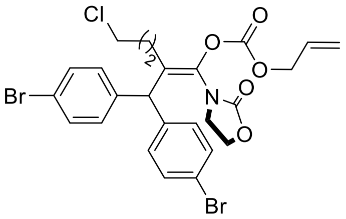

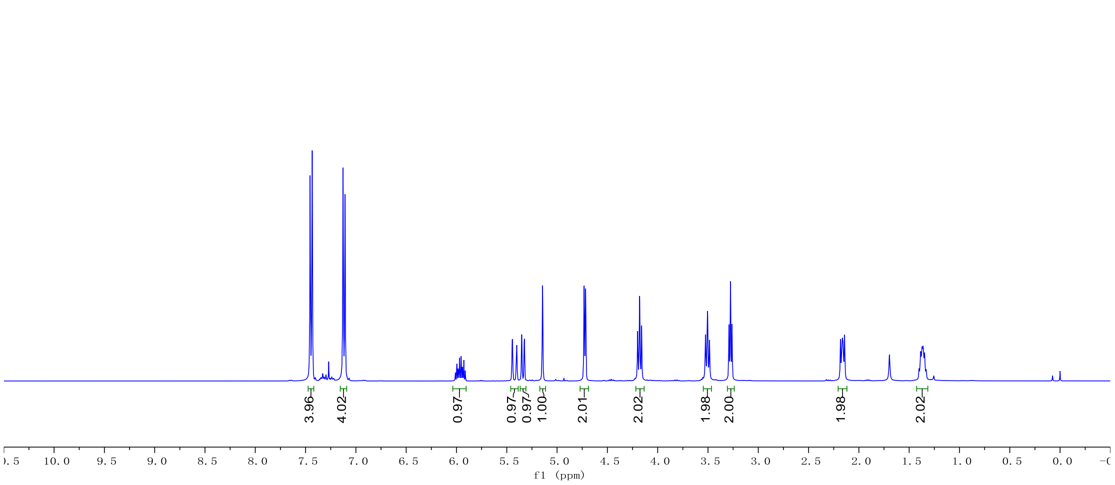

$^{13}$C NMR (100 MHz, $CDCl_3$)

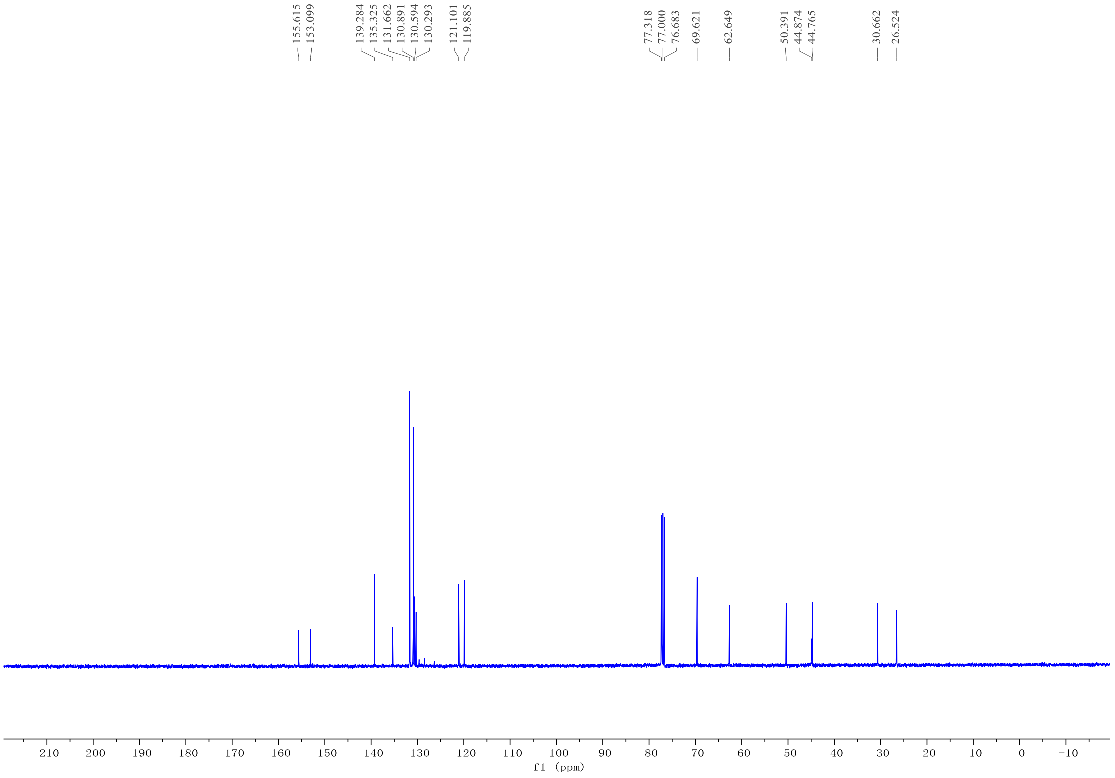

## $^{1}$H and $^{13}$C NMR Spectra for Compound (*E*)-6oc:

### $^{1}$H NMR (400 MHz, $CDCl_3$)

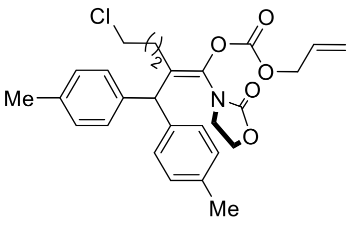


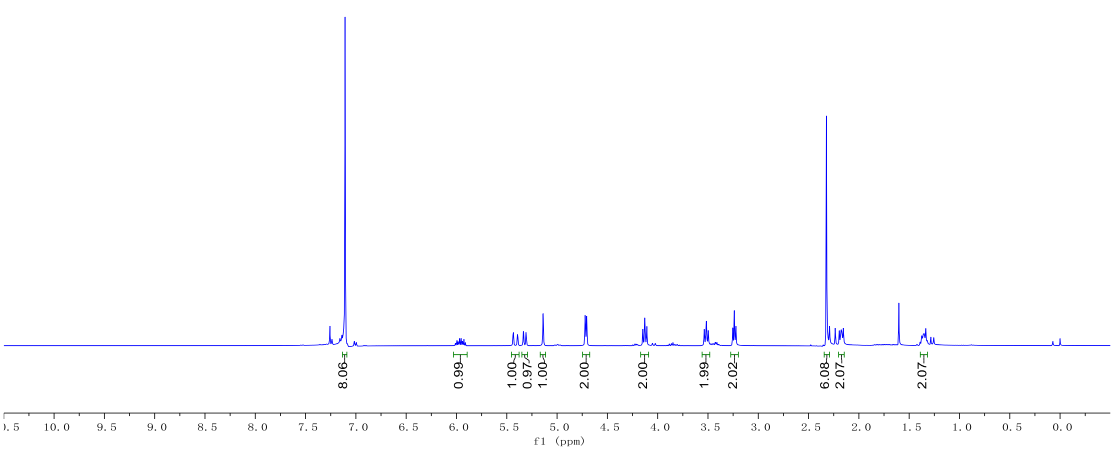


### $^{13}$C NMR (100 MHz, $CDCl_3$)

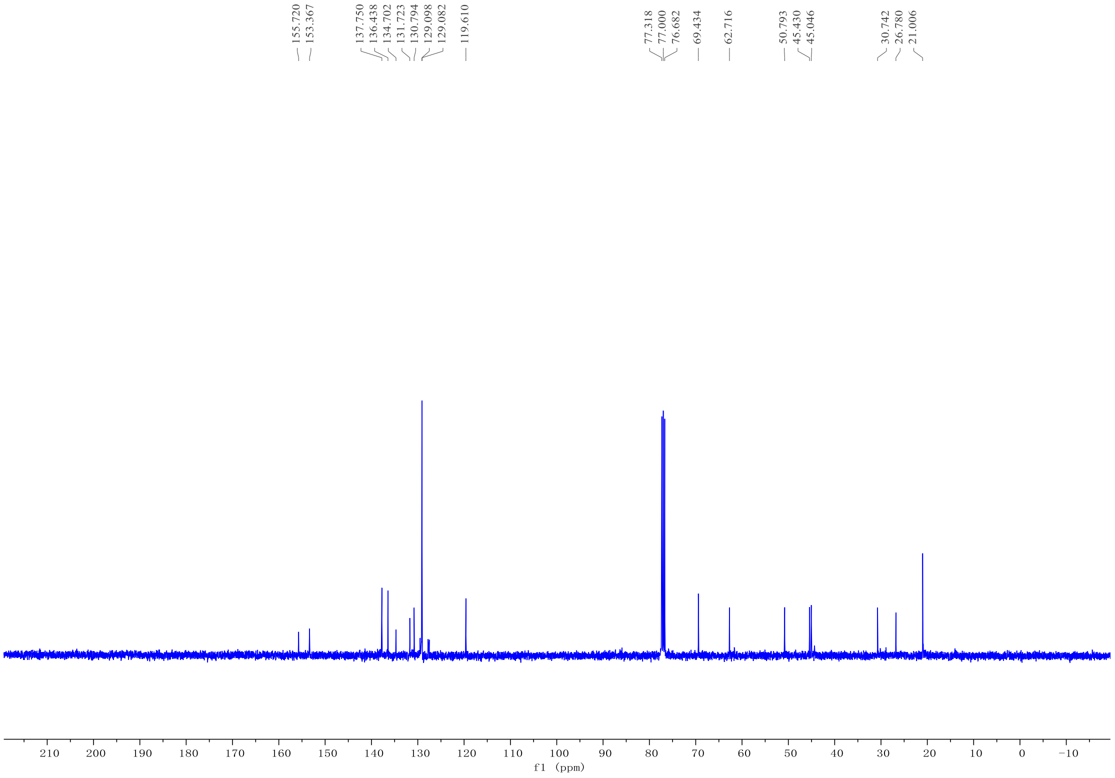

### $^{1}$H and $^{13}$C NMR Spectra for Compound (*E*)-6od:

$^{1}$H NMR (400 MHz, $CDCl_3$)

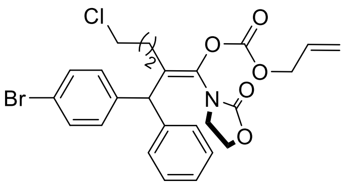


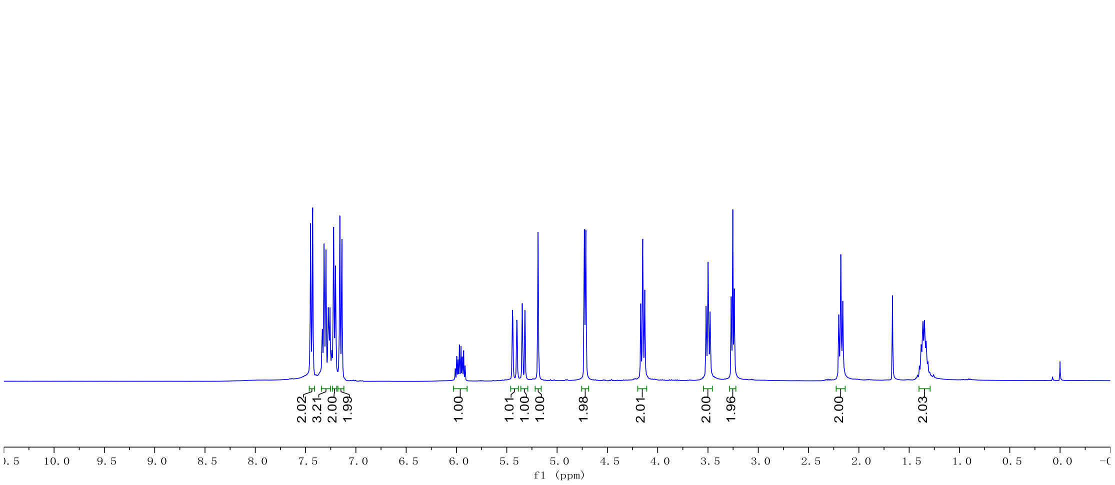


$^{13}$C NMR (100 MHz, $CDCl_3$)

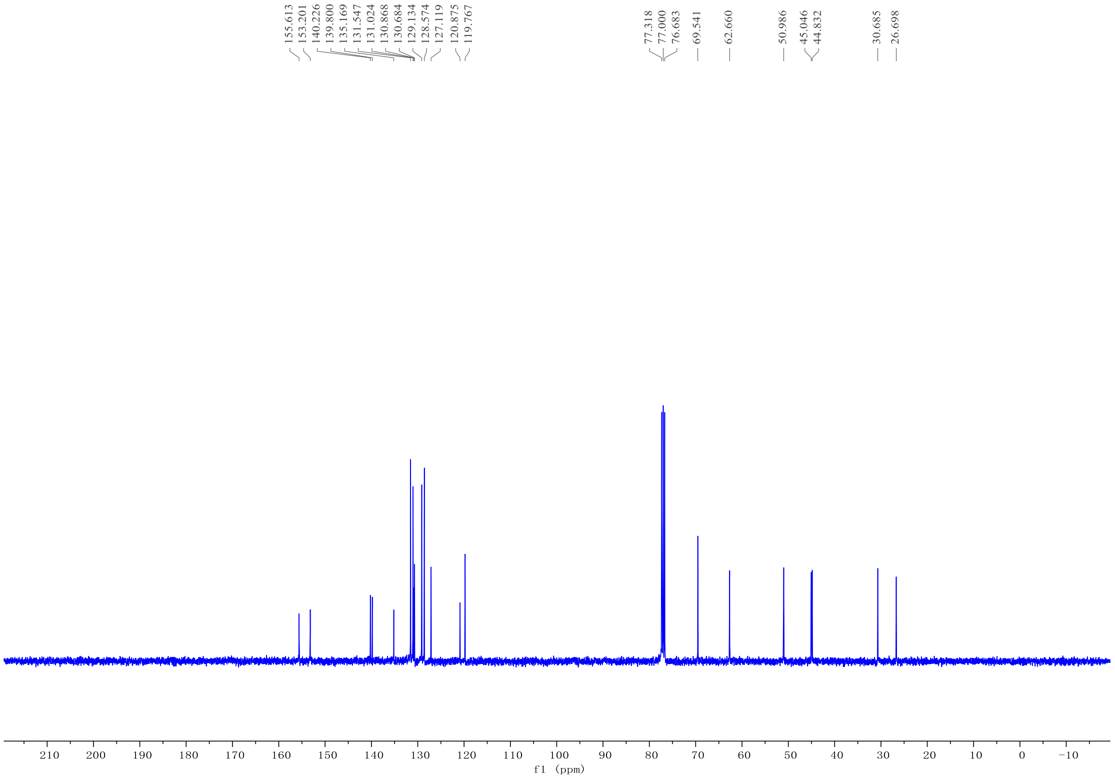

## $^1$H and $^{13}$C NMR Spectra for Compound (*E*)-6oe:

$^1$H NMR (400 MHz, $CDCl_3$)

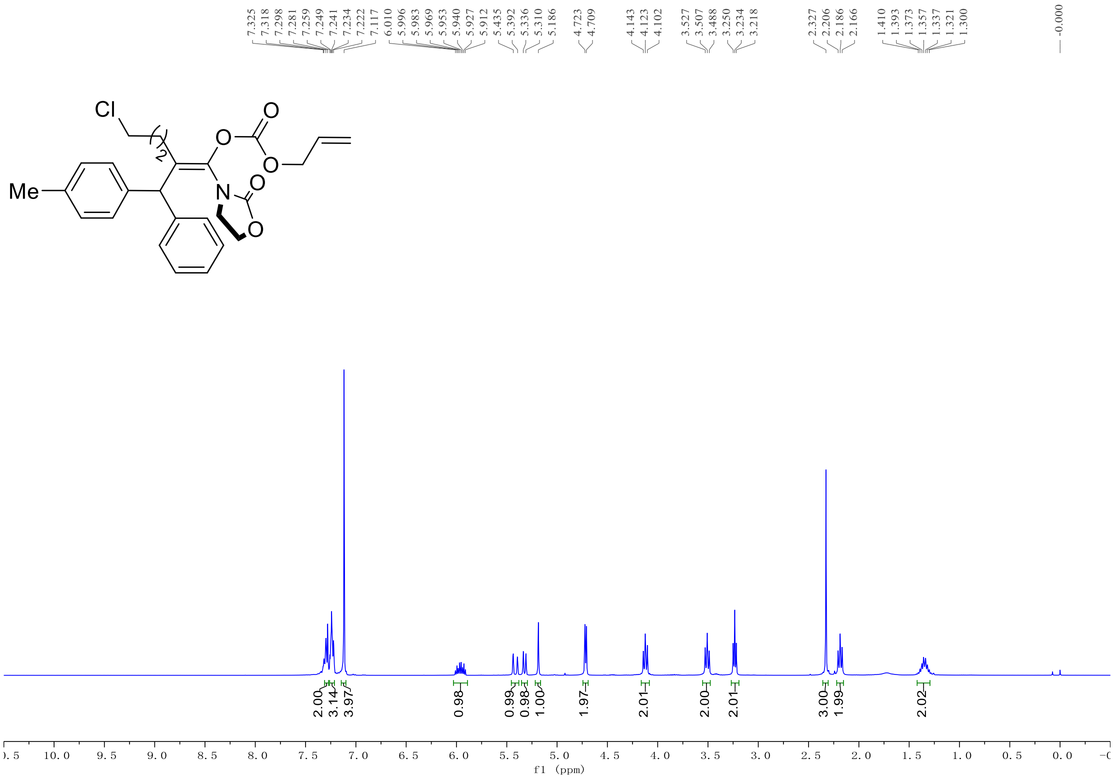


$^{13}$C NMR (100 MHz, $CDCl_3$)

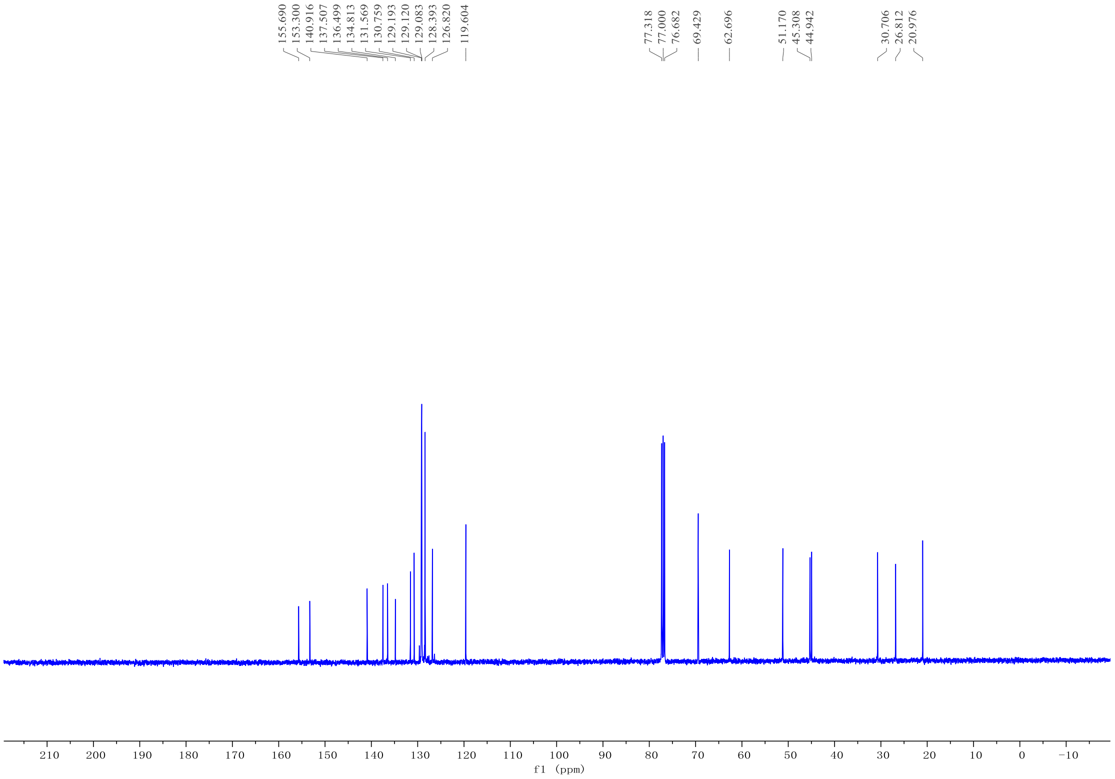

**$^{1}$H and $^{13}$C NMR Spectra for Compound (*E*)-6of**:

$^{1}$H NMR (400 MHz, $CDCl_3$)

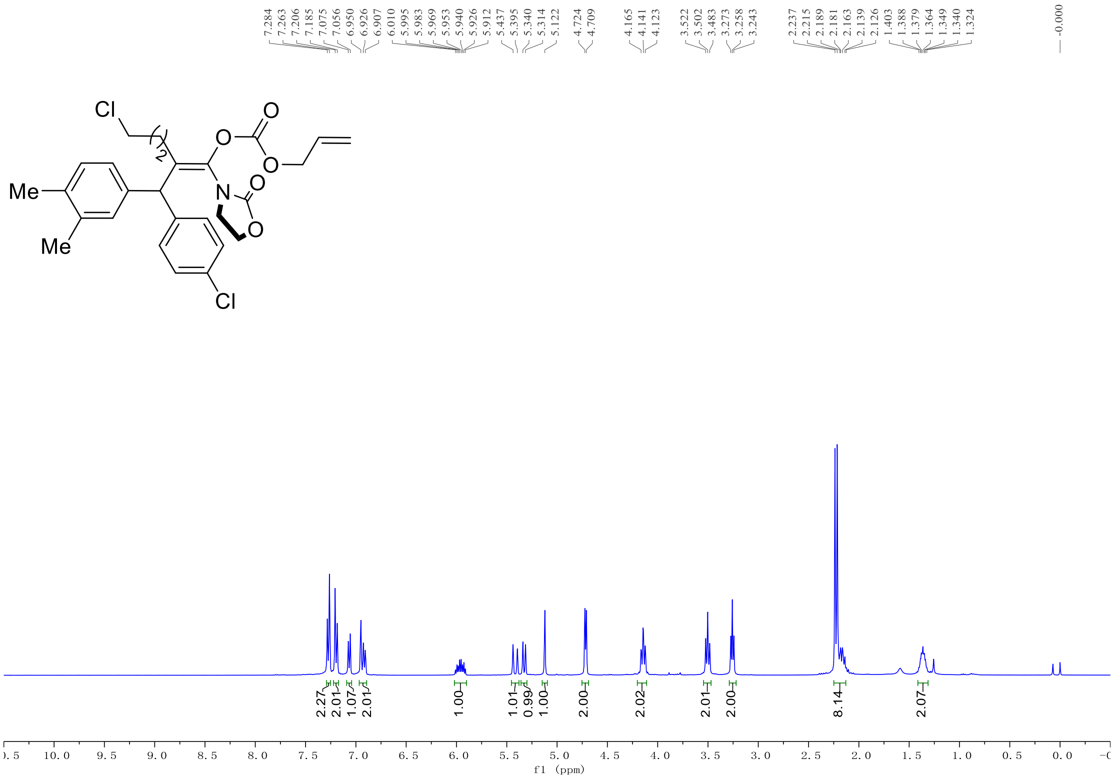


$^{13}$C NMR (100 MHz, $CDCl_3$)

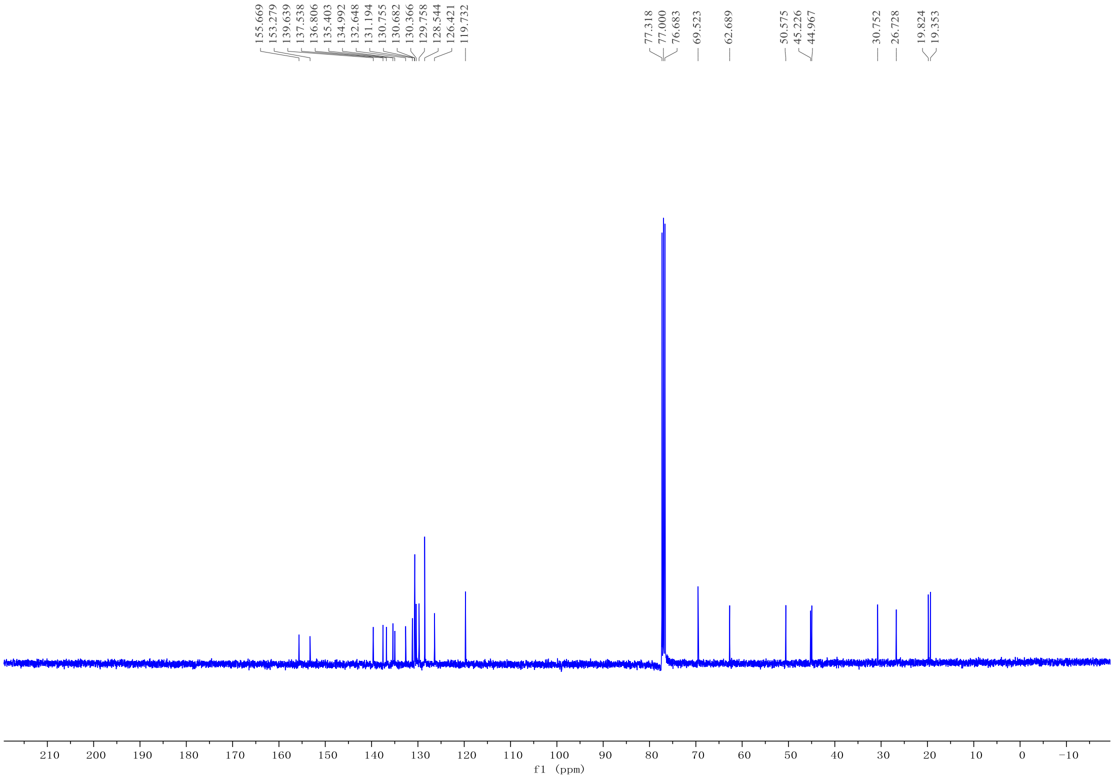

## $^{1}H$ and $^{13}C$ NMR Spectra for Compound (*E*)-6oh:

$^{1}H$ NMR (400 MHz, $CDCl_3$)

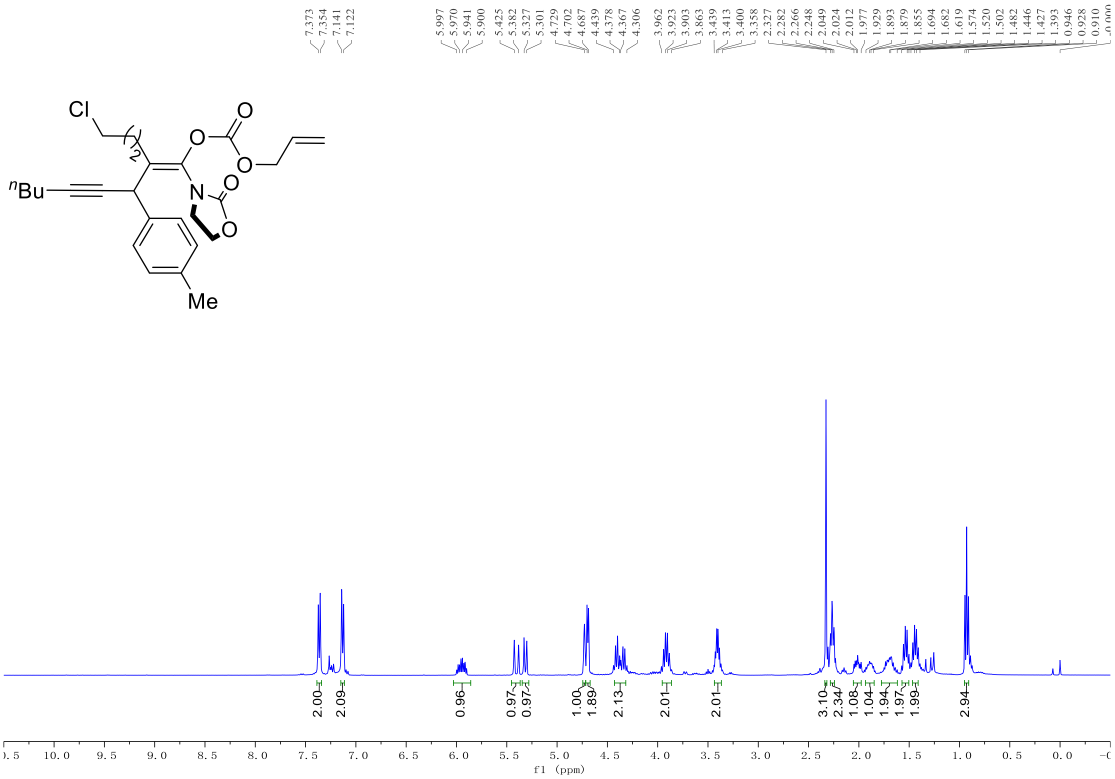


$^{13}C$ NMR (100 MHz, $CDCl_3$)

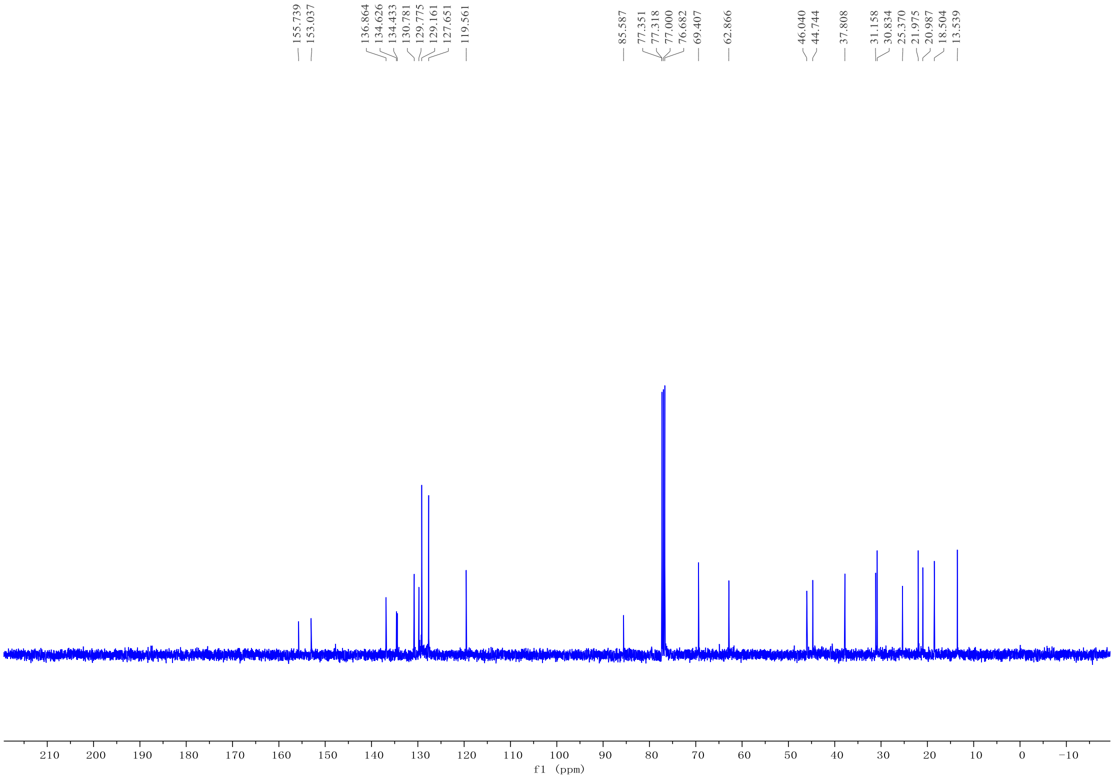

## $^{1}$H and $^{13}$C NMR Spectra for Compound (*E*)-6oi:

$^{1}$H NMR (400 MHz, $CDCl_3$)

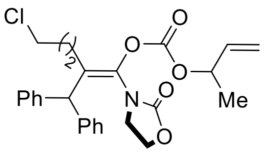


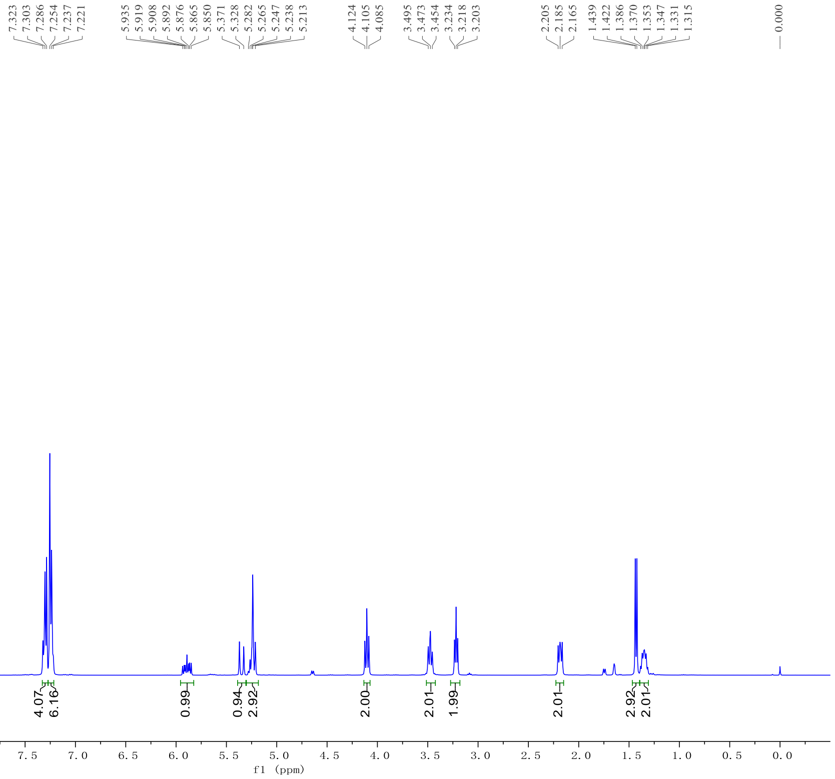


$^{13}$C NMR (100 MHz, $CDCl_3$)

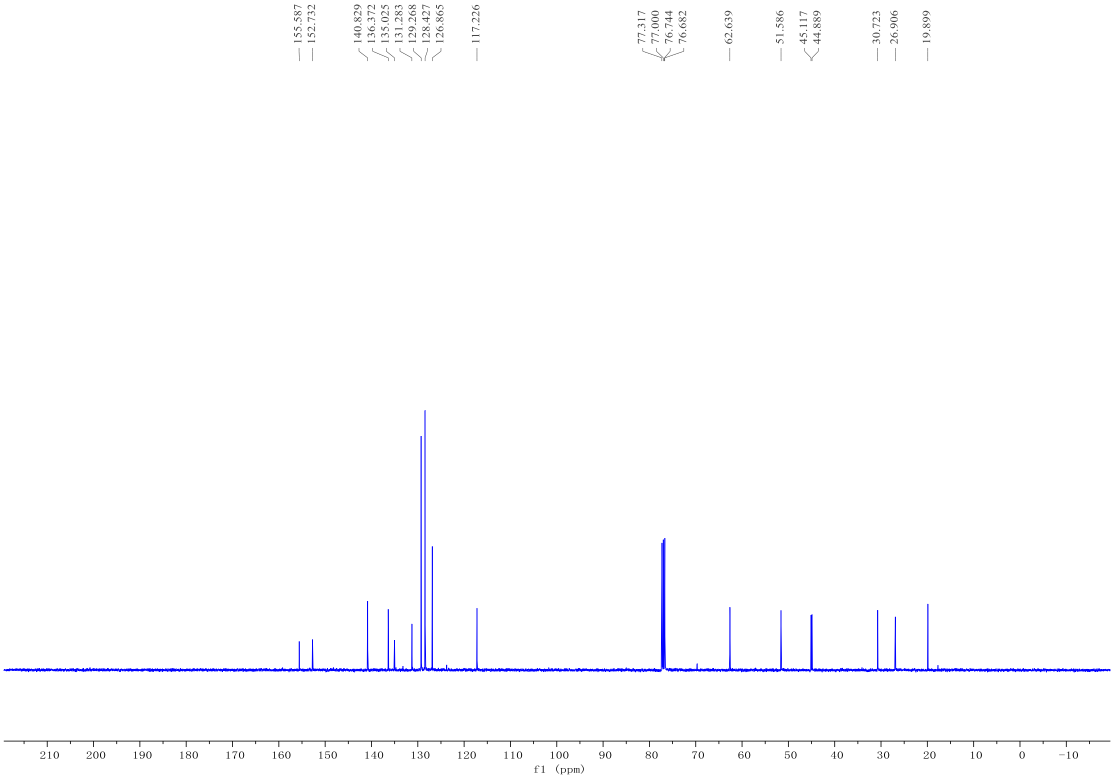

## $^{1}$H and $^{13}$C NMR Spectra for Compound (*E*)-6oj:

$^{1}$H NMR (400 MHz, $CDCl_3$)

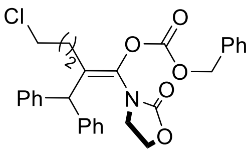

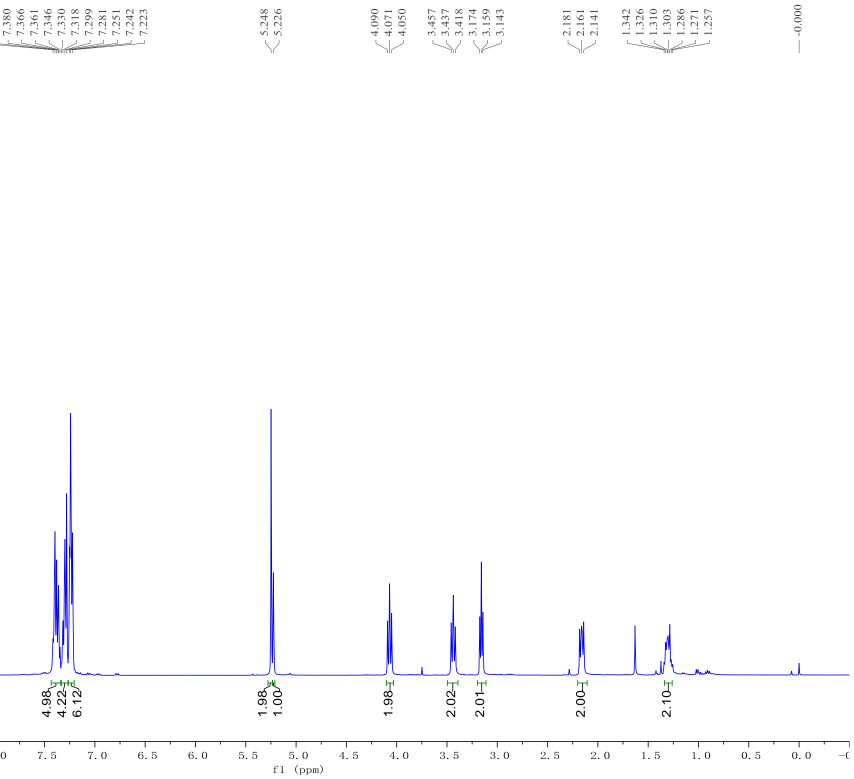

$^{13}$C NMR (100 MHz, $CDCl_3$)

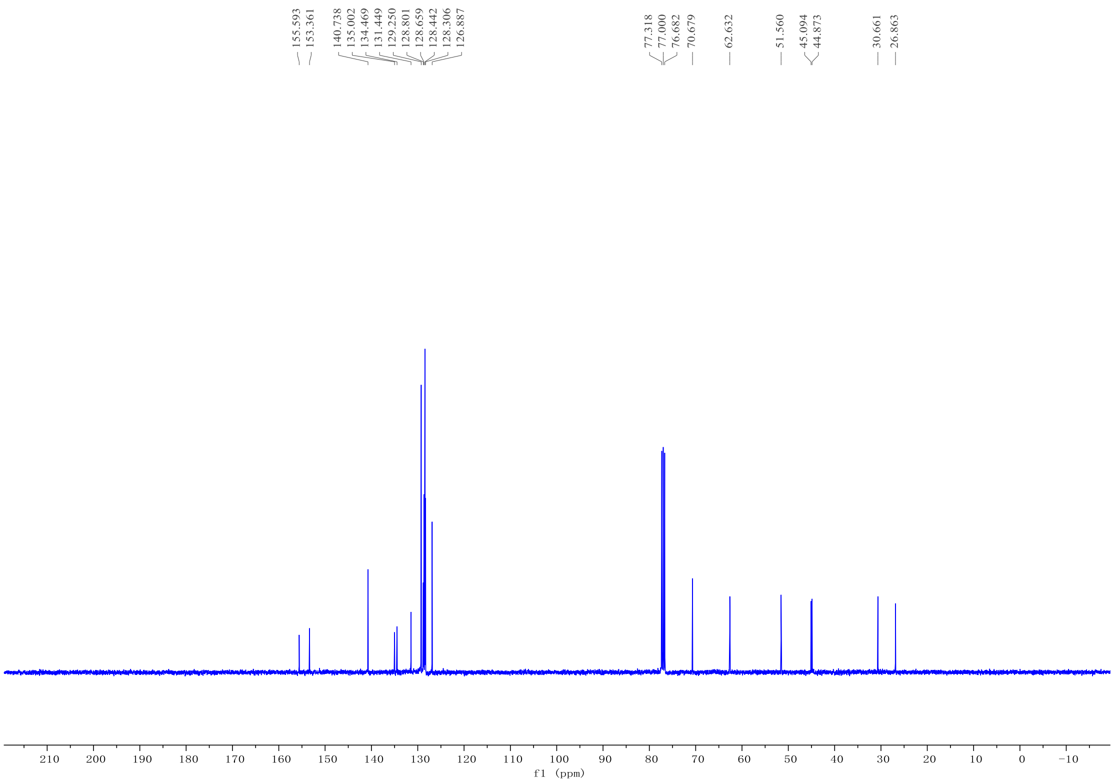

## $^{1}$H and $^{13}$C NMR Spectra for Compound (*E*)-6ok:

$^{1}$H NMR (400 MHz, $CDCl_3$)

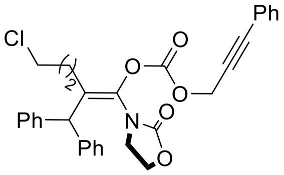


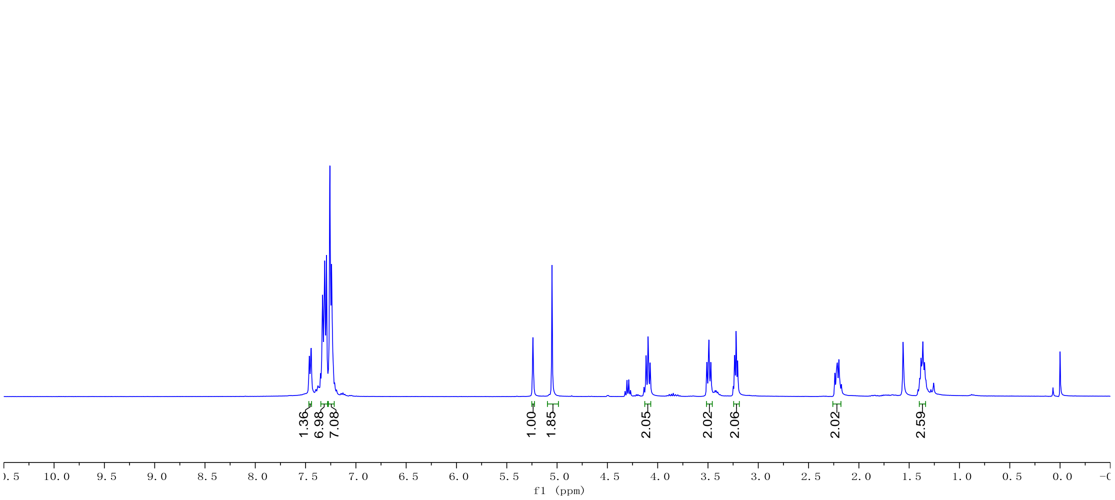


$^{13}$C NMR (100 MHz, $CDCl_3$)

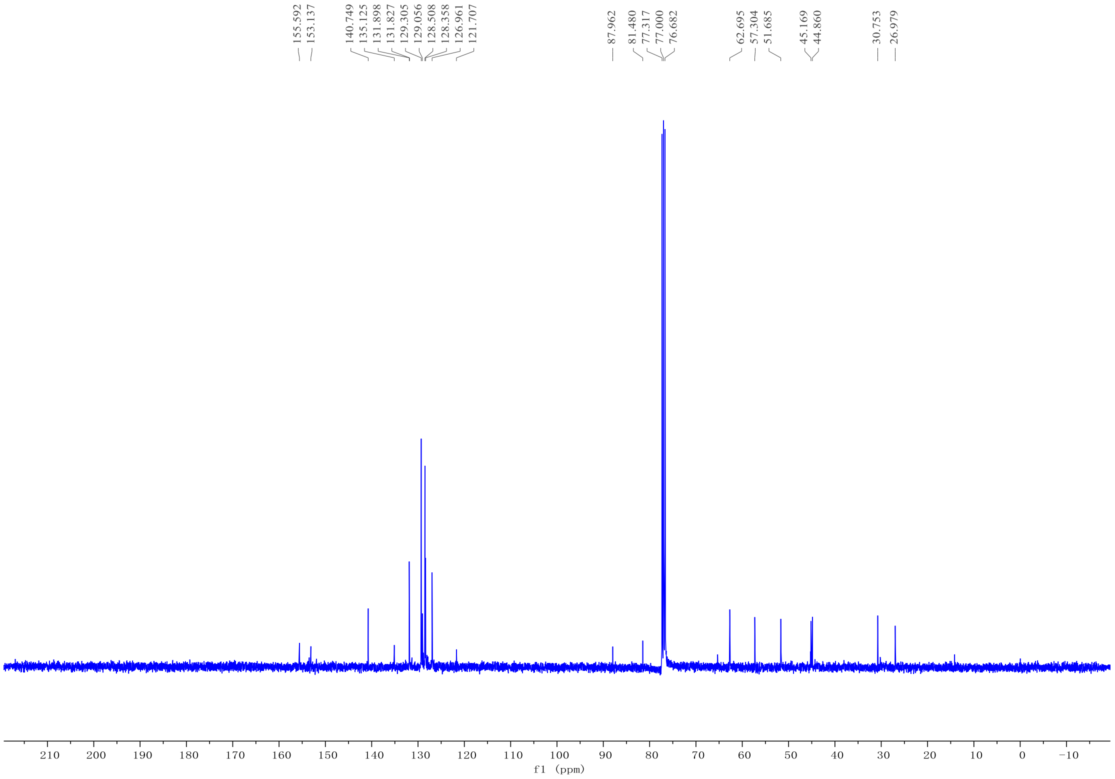

## $^{1}$H and $^{13}$C NMR Spectra for Compound (*E*)-6ol:

$^{1}$H NMR (400 MHz, $CDCl_3$)

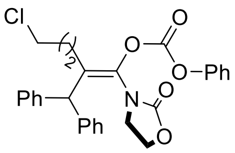


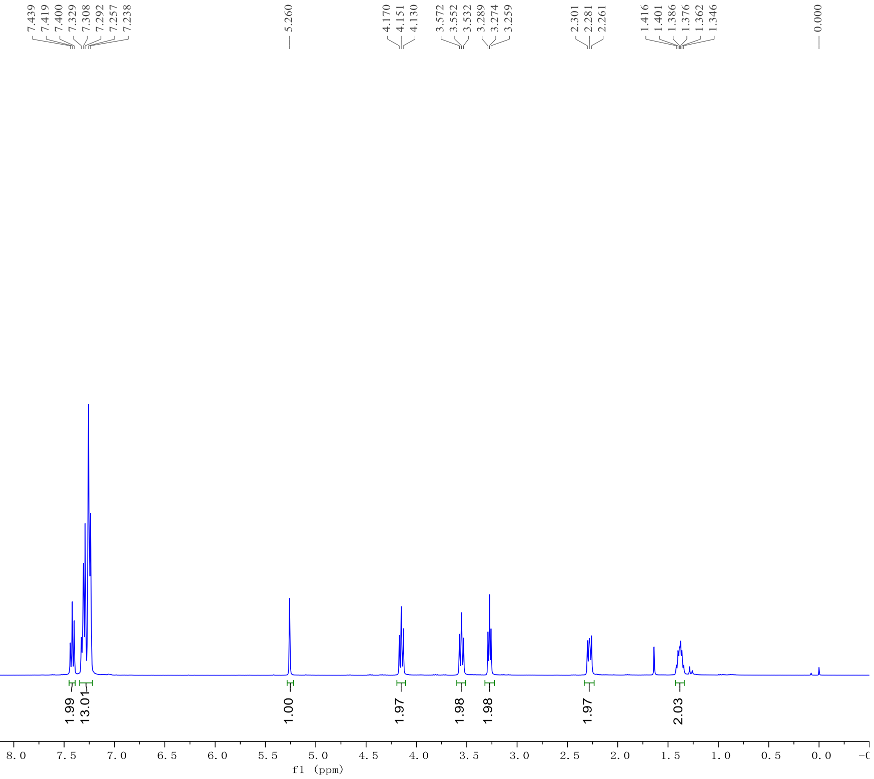


$^{13}$C NMR (100 MHz, $CDCl_3$)

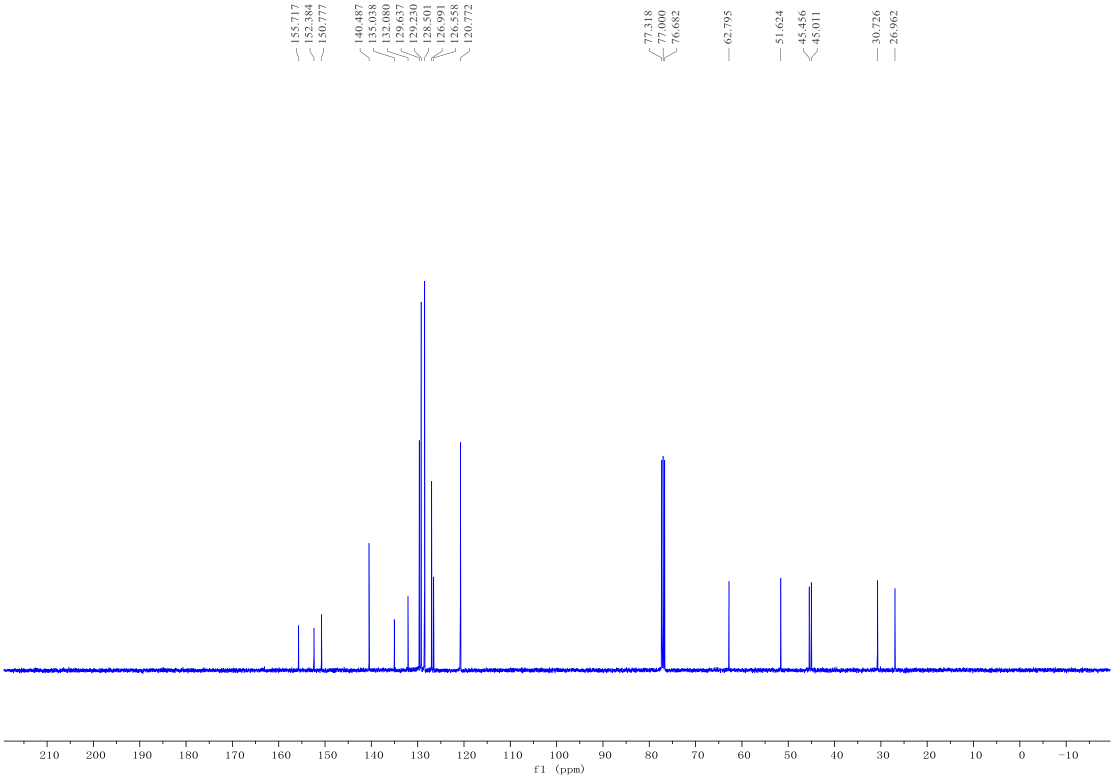

## $^{1}$H and $^{13}$C NMR Spectra for Compound (*E*)-6sa:

$^{1}$H NMR (400 MHz, $CDCl_3$)

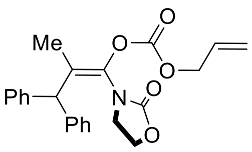


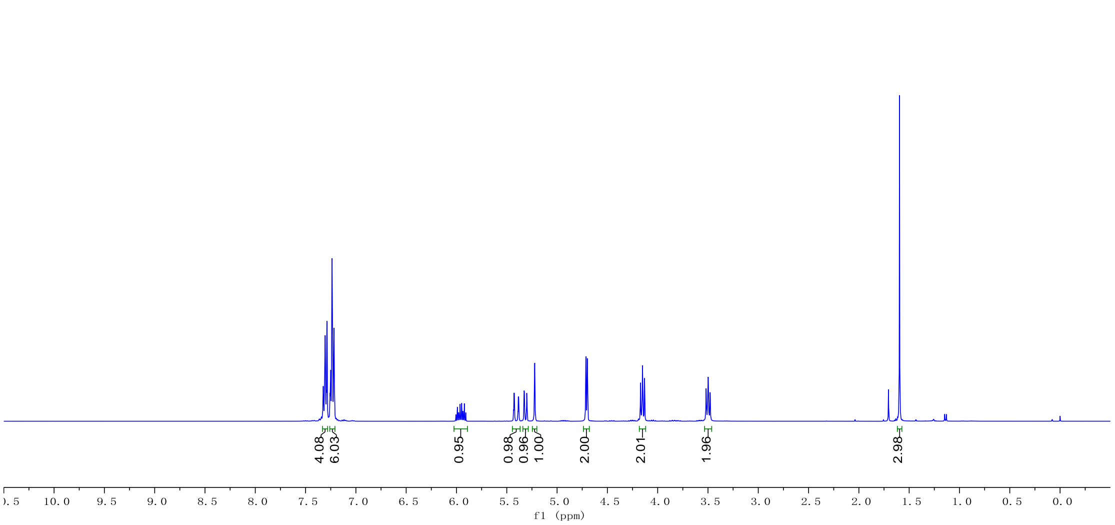


$^{13}$C NMR (100 MHz, $CDCl_3$)

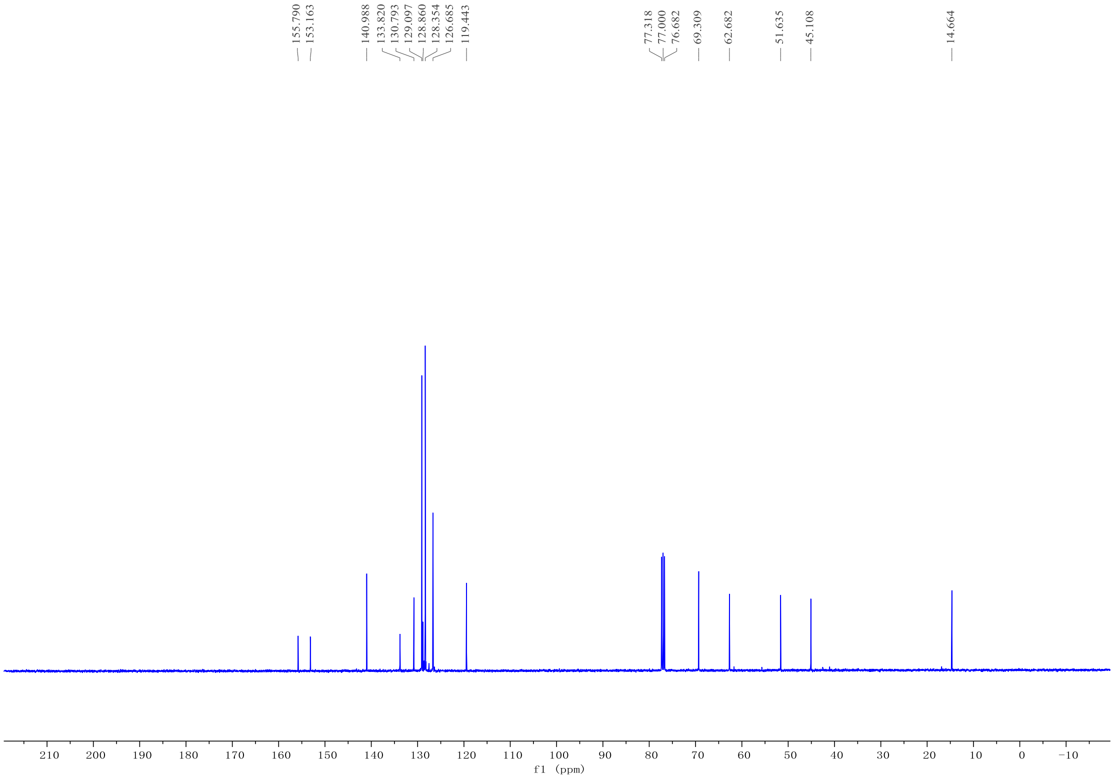

# $^{1}$H and $^{13}$C NMR Spectra for Compound (*E*)-6ta:

$^{1}$H NMR (400 MHz, CDCl$_3$)

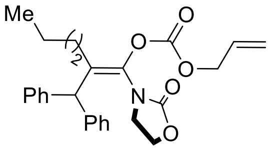

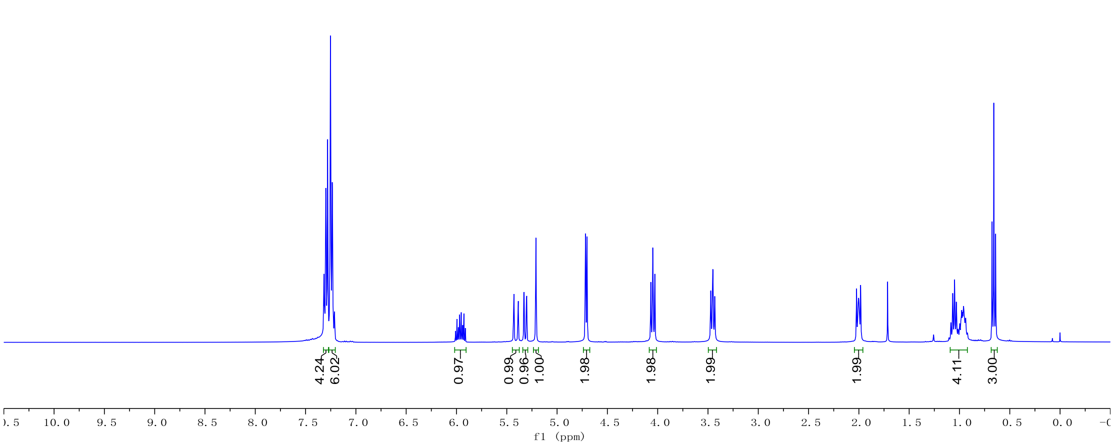

$^{13}$C NMR (100 MHz, CDCl$_3$)

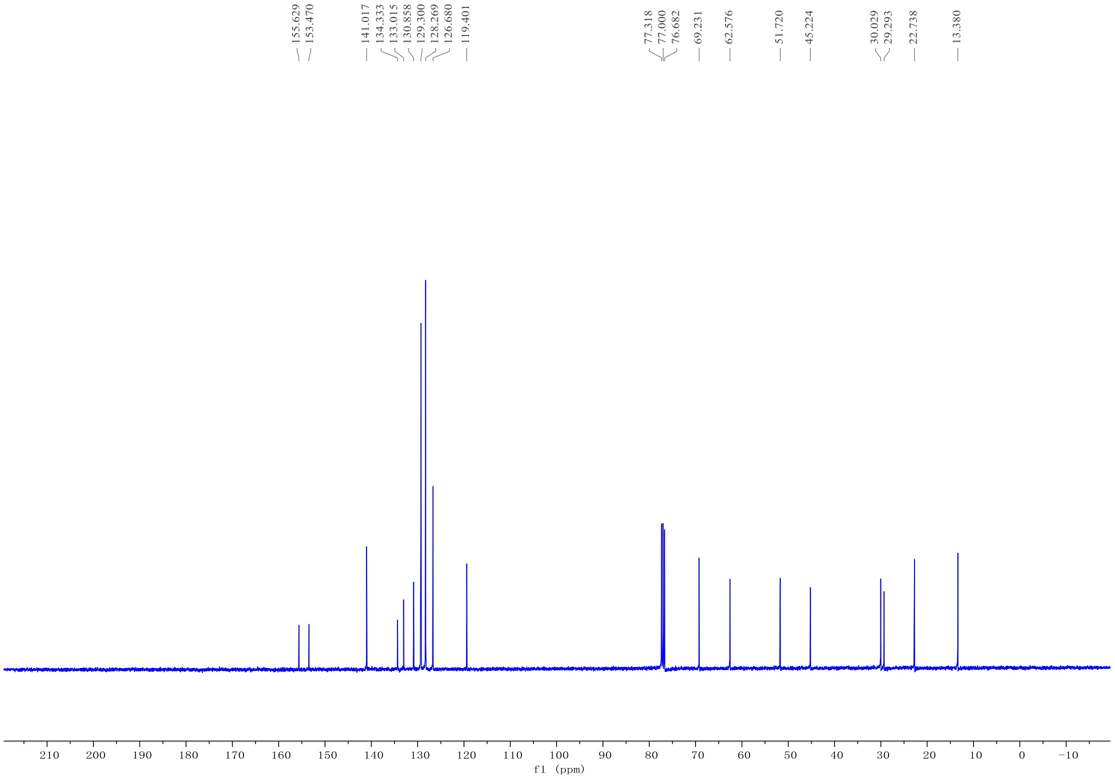

**$^{1}$H and $^{13}$C NMR Spectra for Compound (*E*)-6na**:

$^{1}$H NMR (400 MHz, $CDCl_3$)

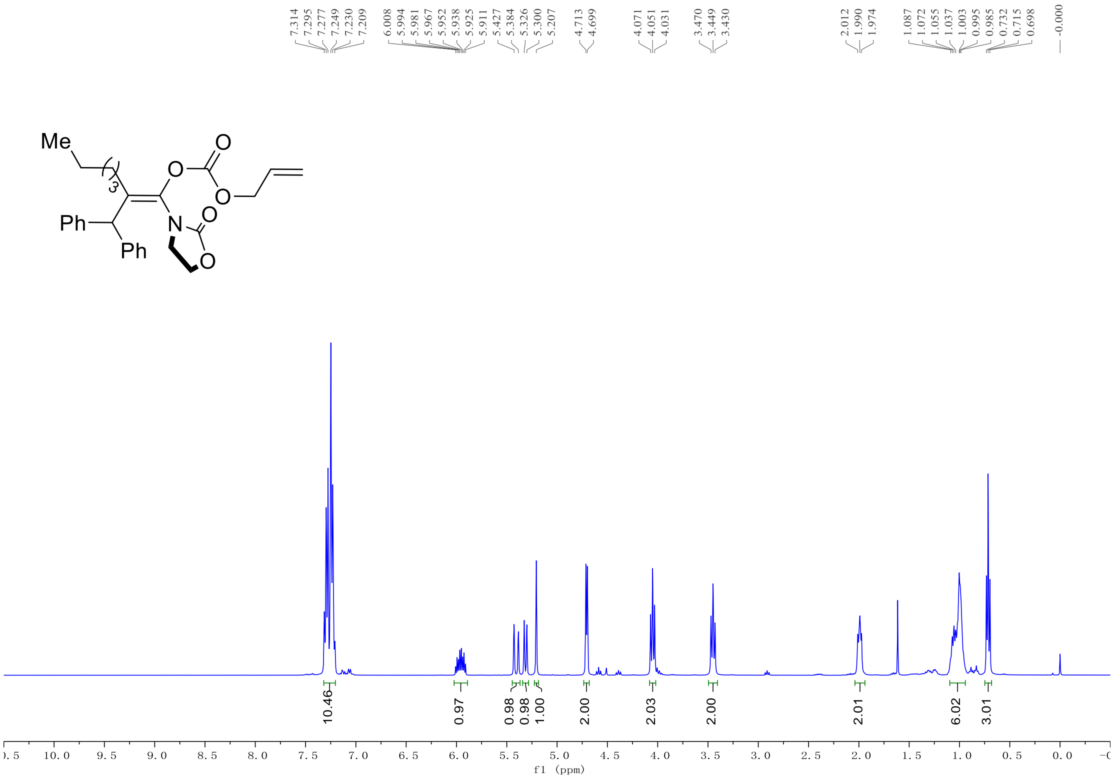

$^{13}$C NMR (100 MHz, $CDCl_3$)

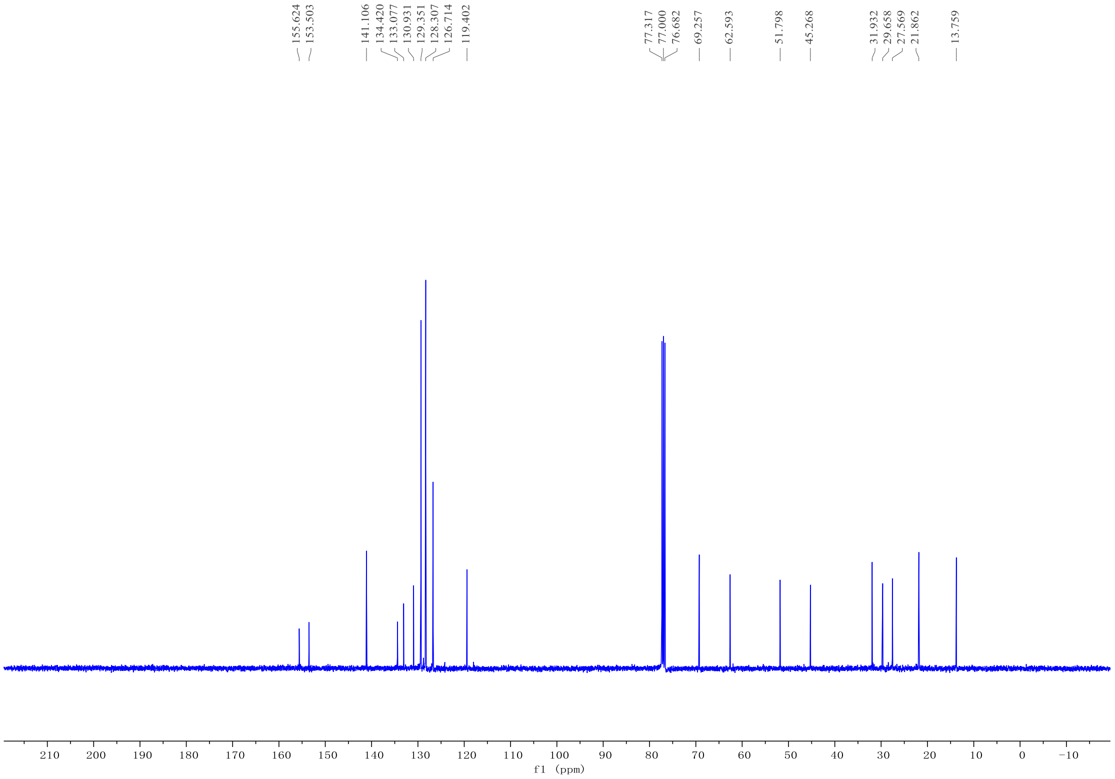

**$^{1}$H and $^{13}$C NMR Spectra for Compound (*Z*)-6aa**:

$^{1}$H NMR (400 MHz, $CDCl_3$)

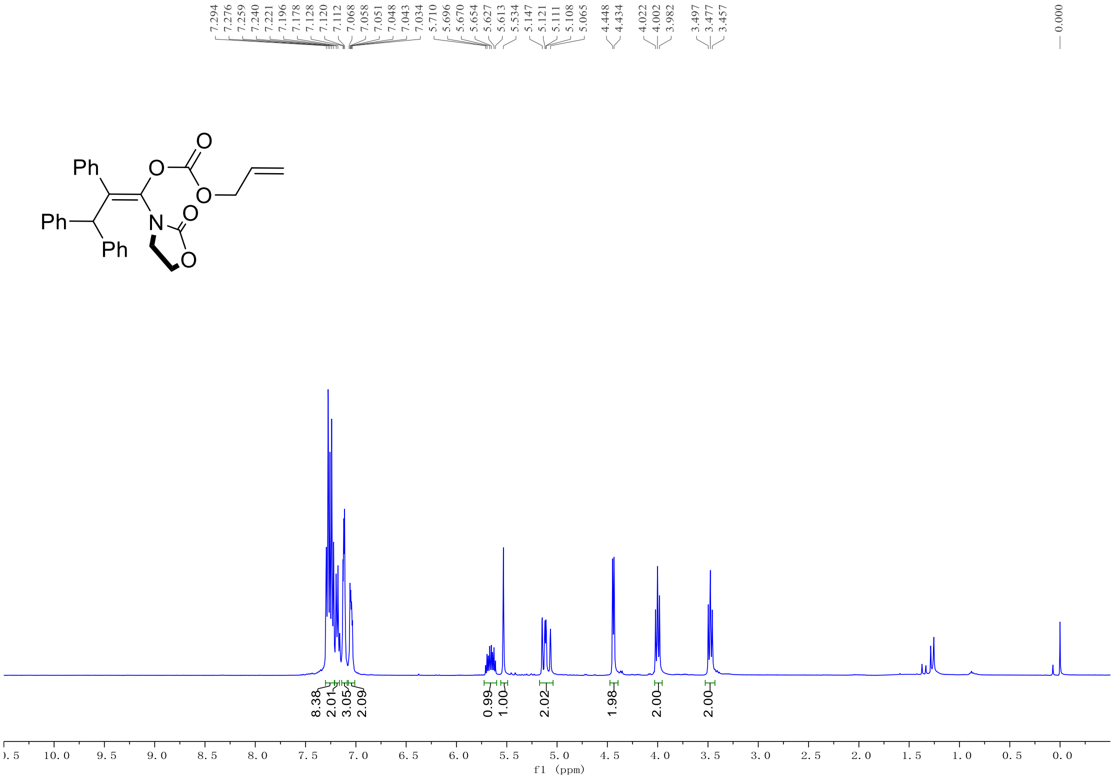


$^{13}$C NMR (100 MHz, $CDCl_3$)

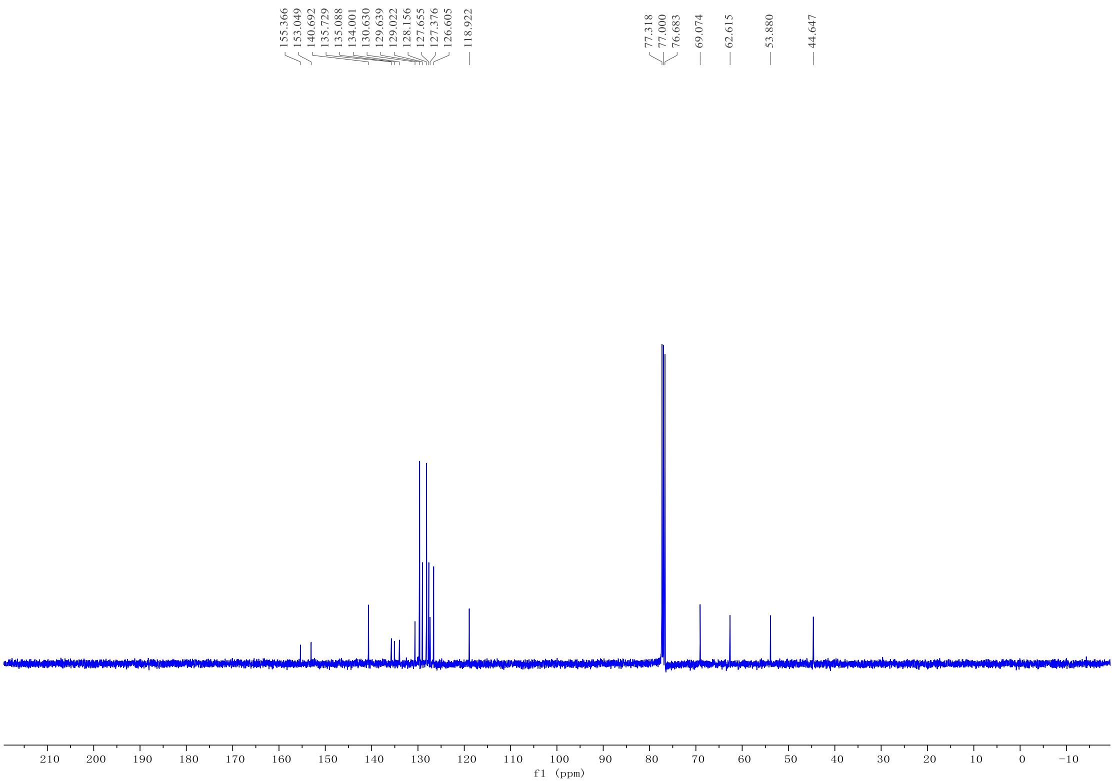

## $^{1}$H and $^{13}$C NMR Spectra for Compound (*E*)-6ua:

$^{1}$H NMR (400 MHz, $CDCl_3$)

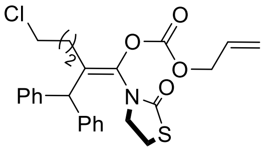


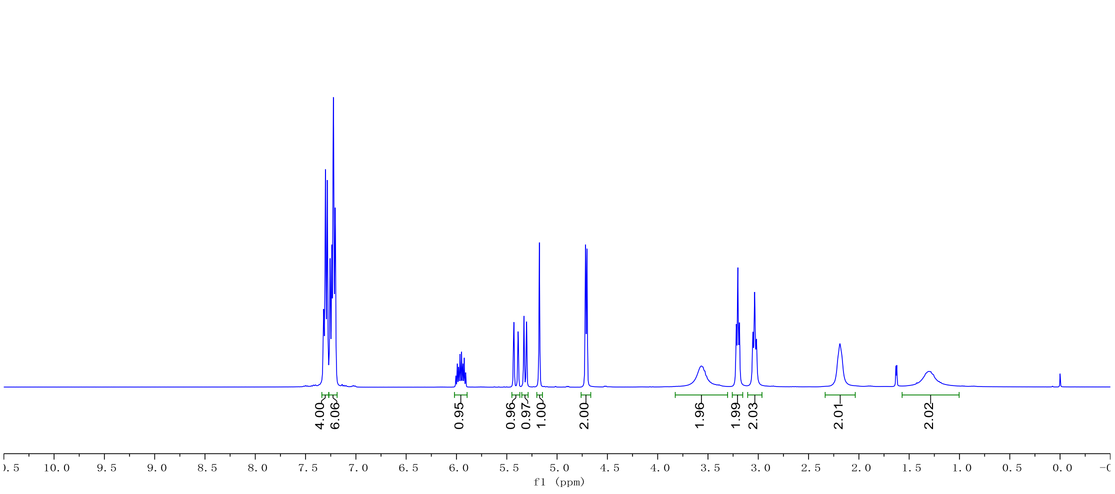


$^{13}$C NMR (100 MHz, $CDCl_3$)

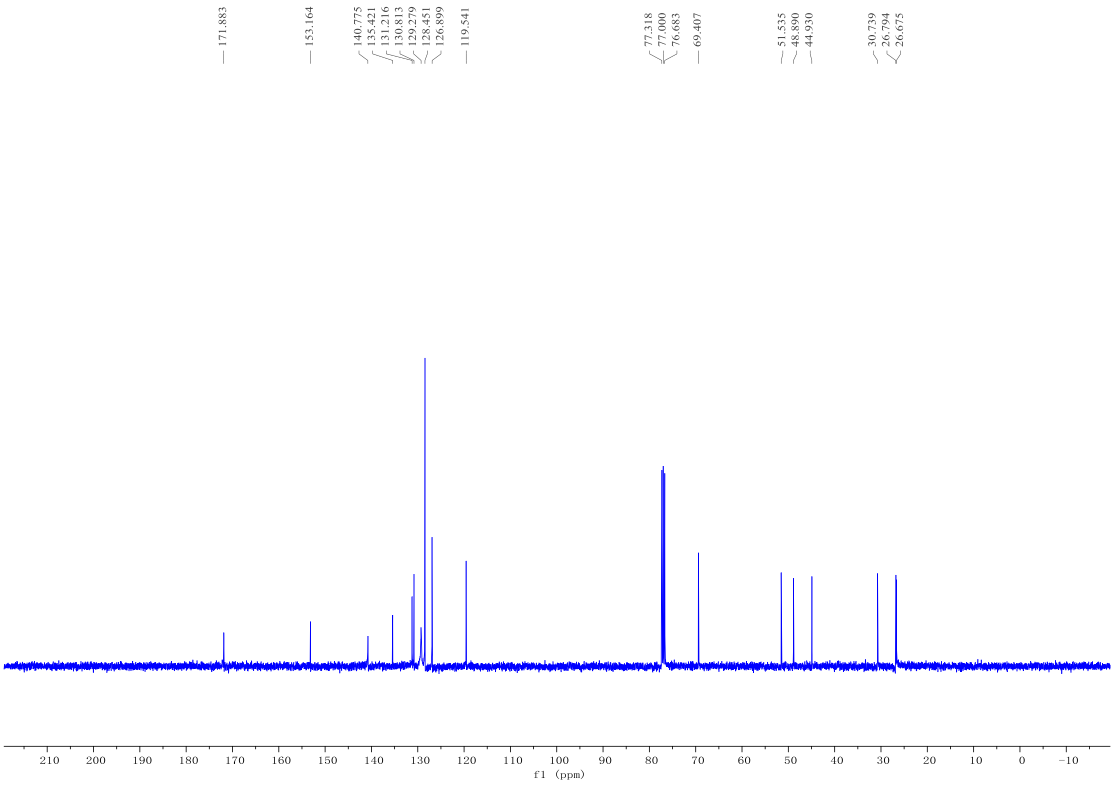

## $^{1}$H and $^{13}$C NMR Spectra for Compound (*E*)-6va:

$^{1}$H NMR (400 MHz, $CDCl_3$)

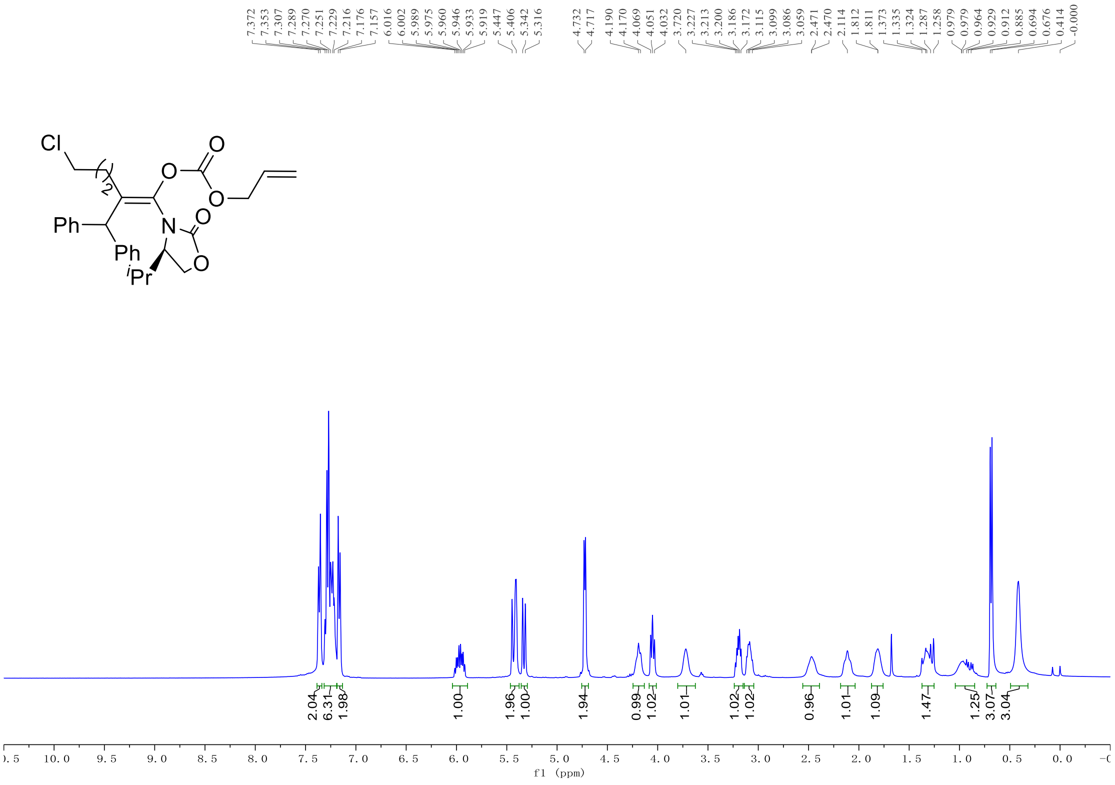


$^{13}$C NMR (100 MHz, $CDCl_3$)

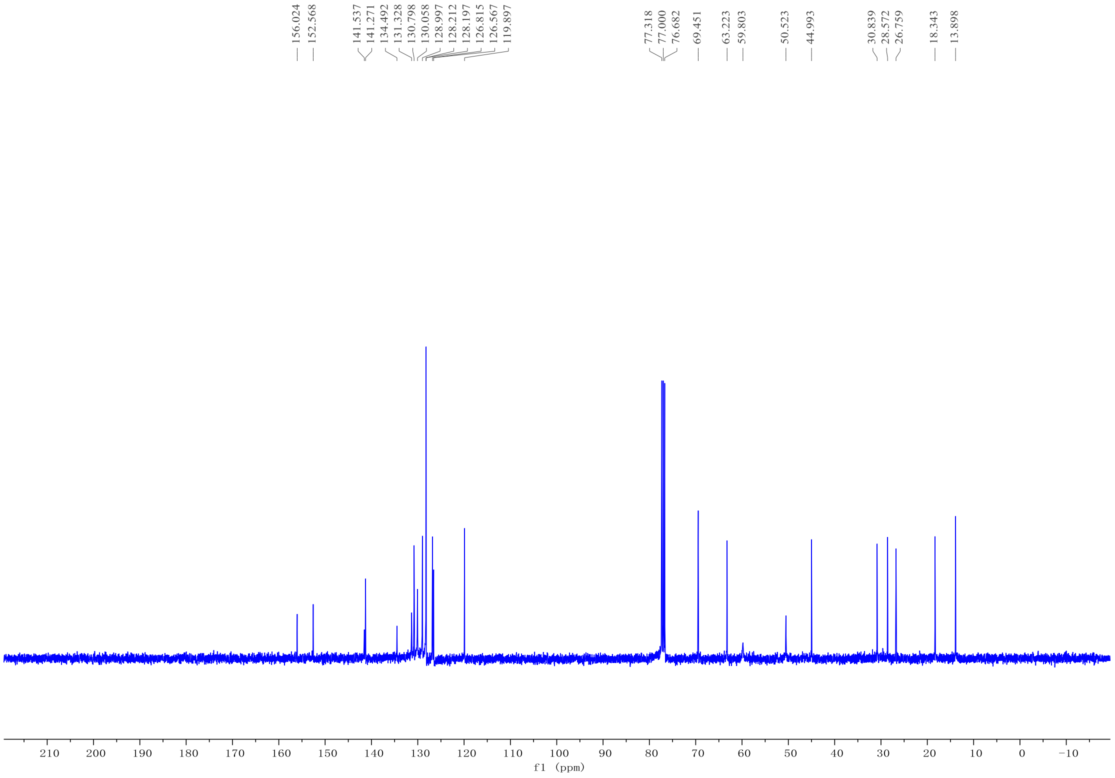

## $^{1}$H and $^{13}$C NMR Spectra for Compound (8):

$^{1}$H NMR (400 MHz, $CDCl_3$)

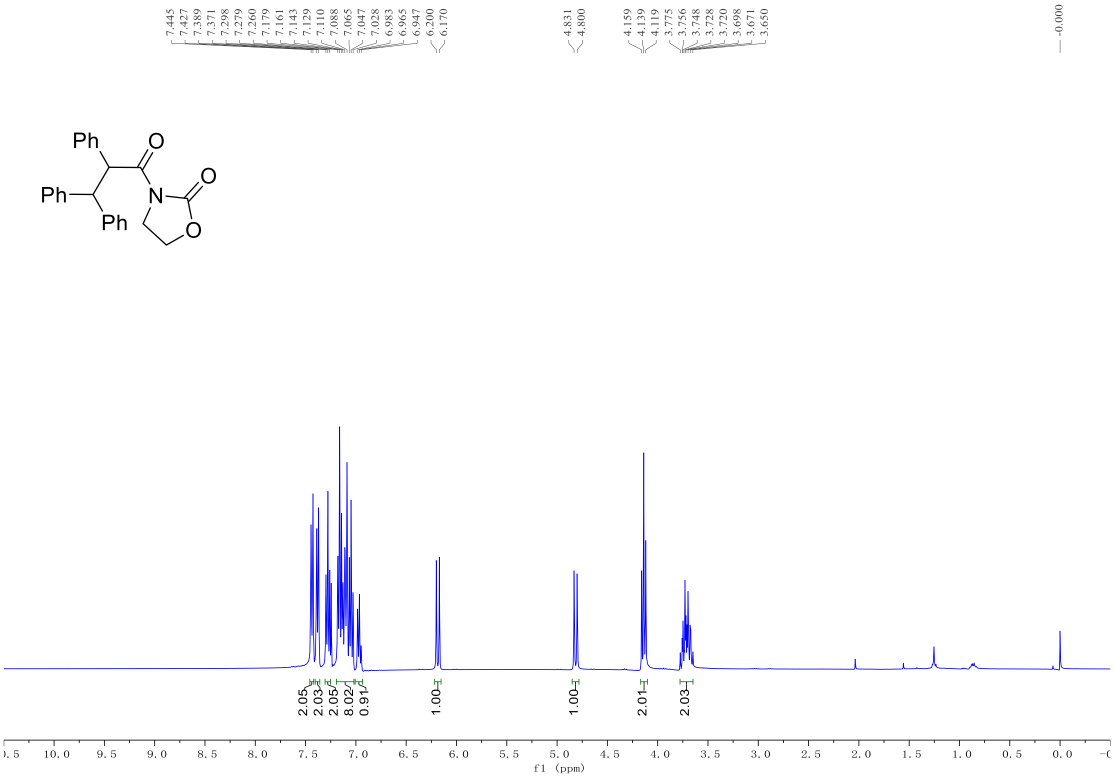


$^{13}$C NMR (100 MHz, $CDCl_3$)

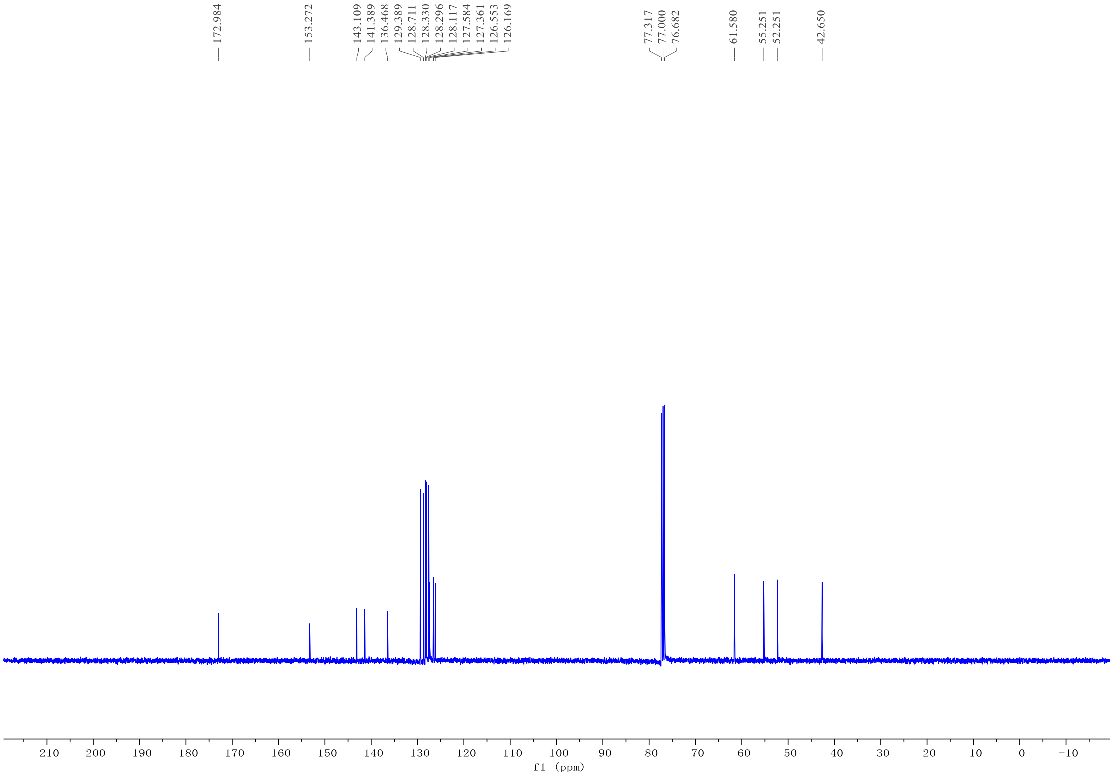

**$^{1}$H and $^{13}$C NMR Spectra for Compound (9)**:

$^{1}$H NMR (400 MHz, $CDCl_3$)

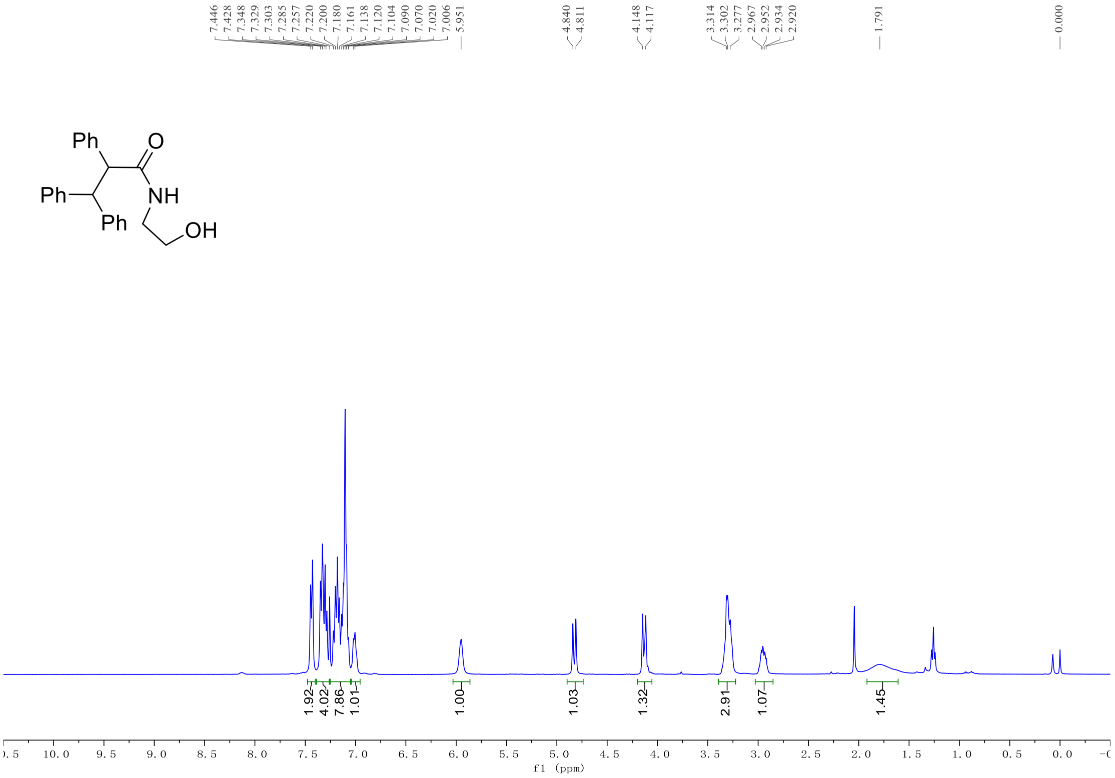

$^{13}$C NMR (100 MHz, $CDCl_3$)

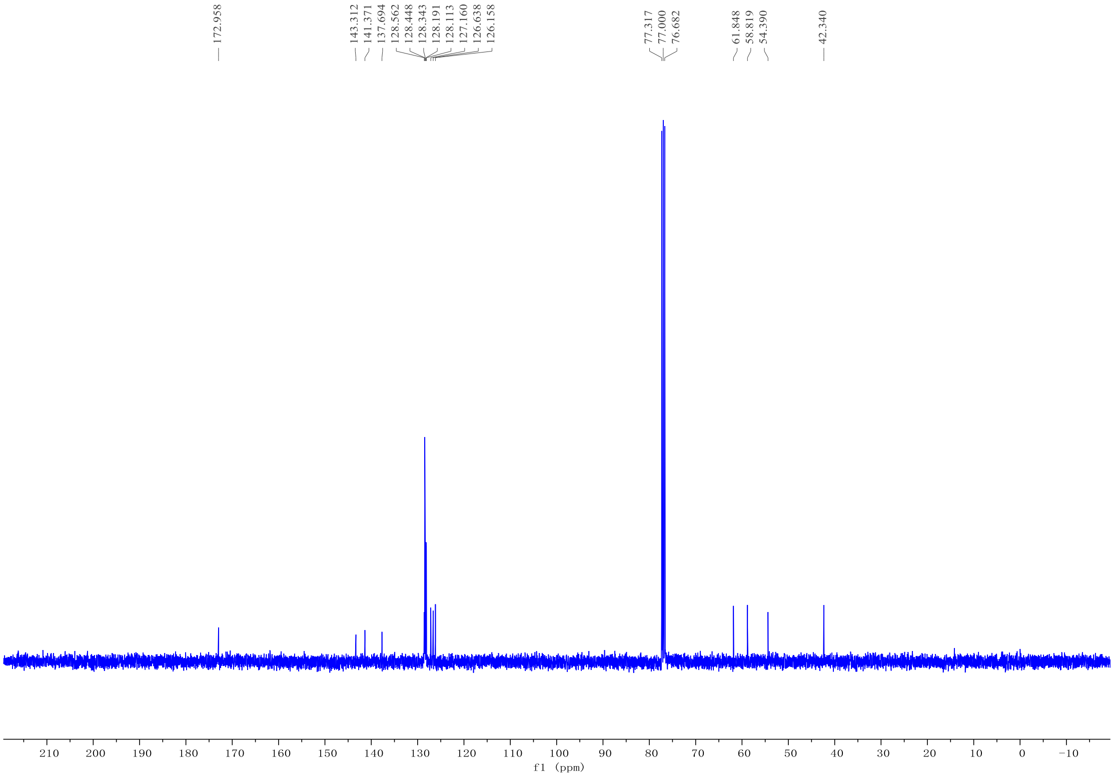

## $^{1}H$ and $^{13}C$ NMR Spectra for Compound (10):

$^{1}H$ NMR (400 MHz, $CDCl_3$)

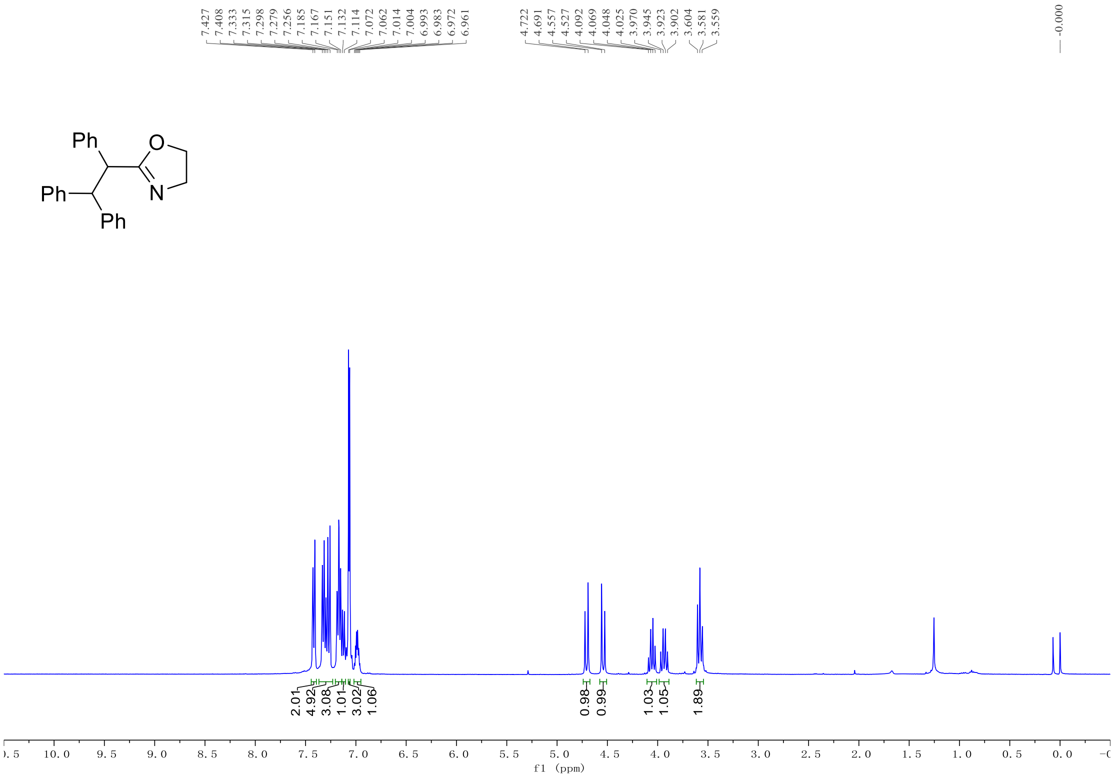

$^{13}C$ NMR (100 MHz, $CDCl_3$)

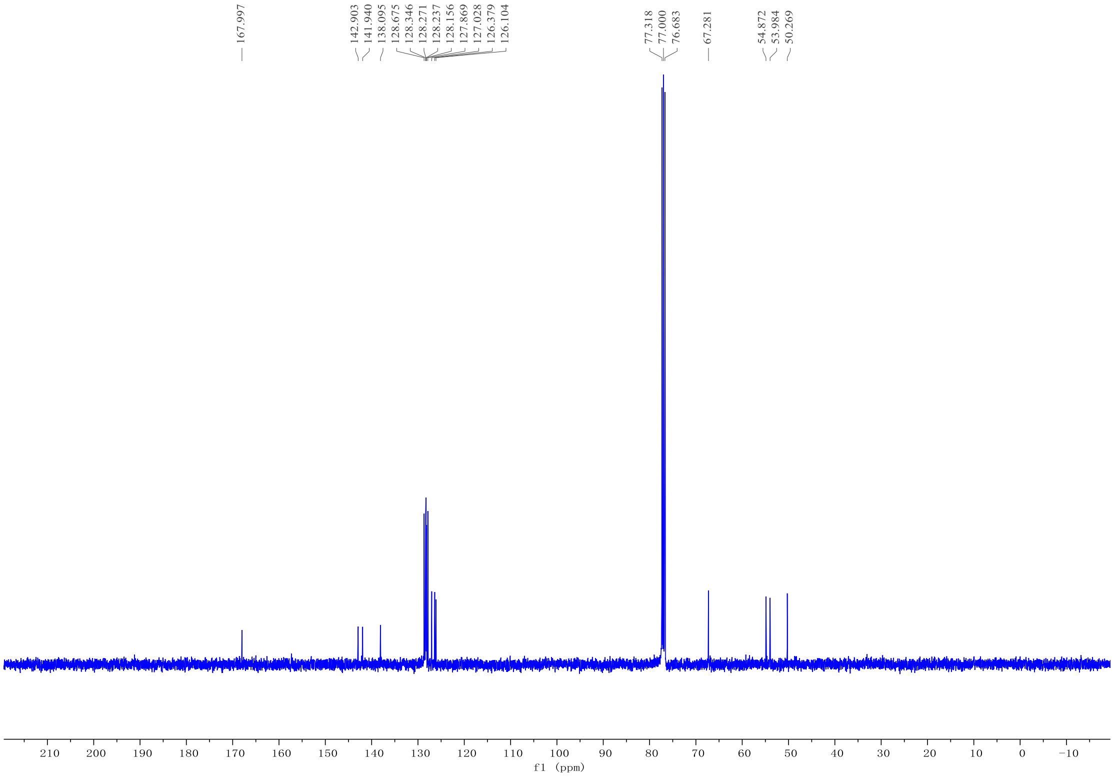

## $^{1}$H, $^{13}$C NMR and $^{19}$F NMR Spectra for Compound (11):

$^{1}$H NMR (400 MHz, $CDCl_3$)

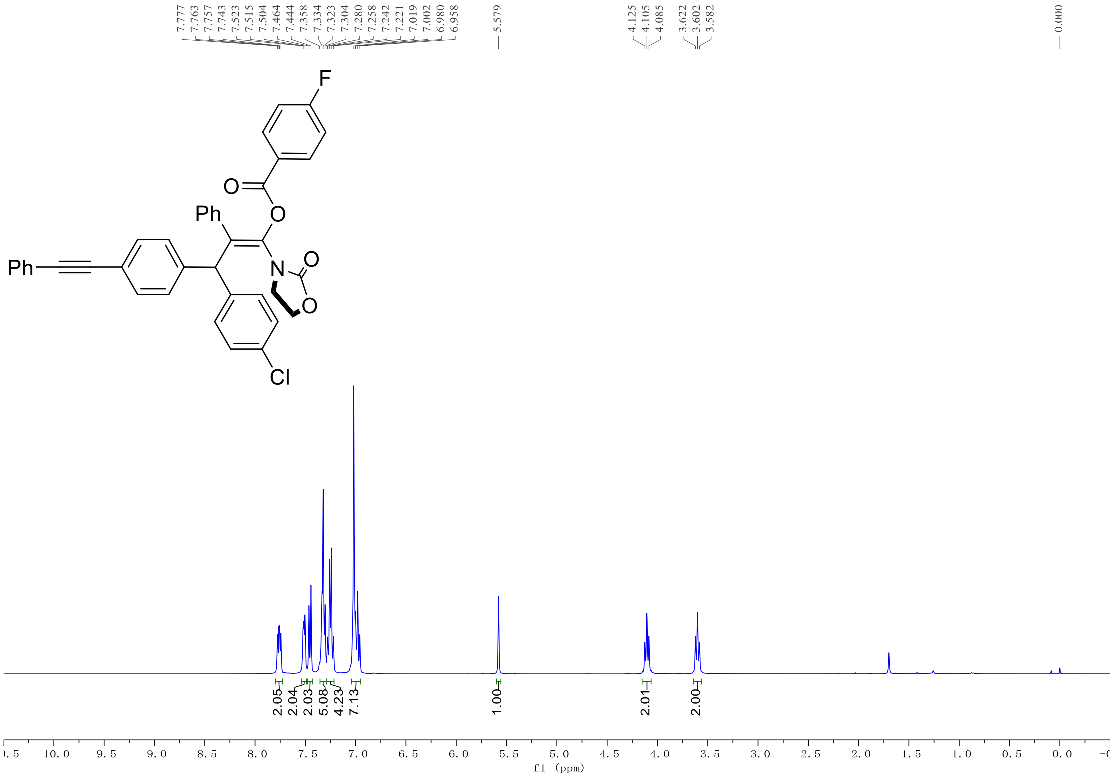


$^{13}$C NMR (100 MHz, $CDCl_3$)

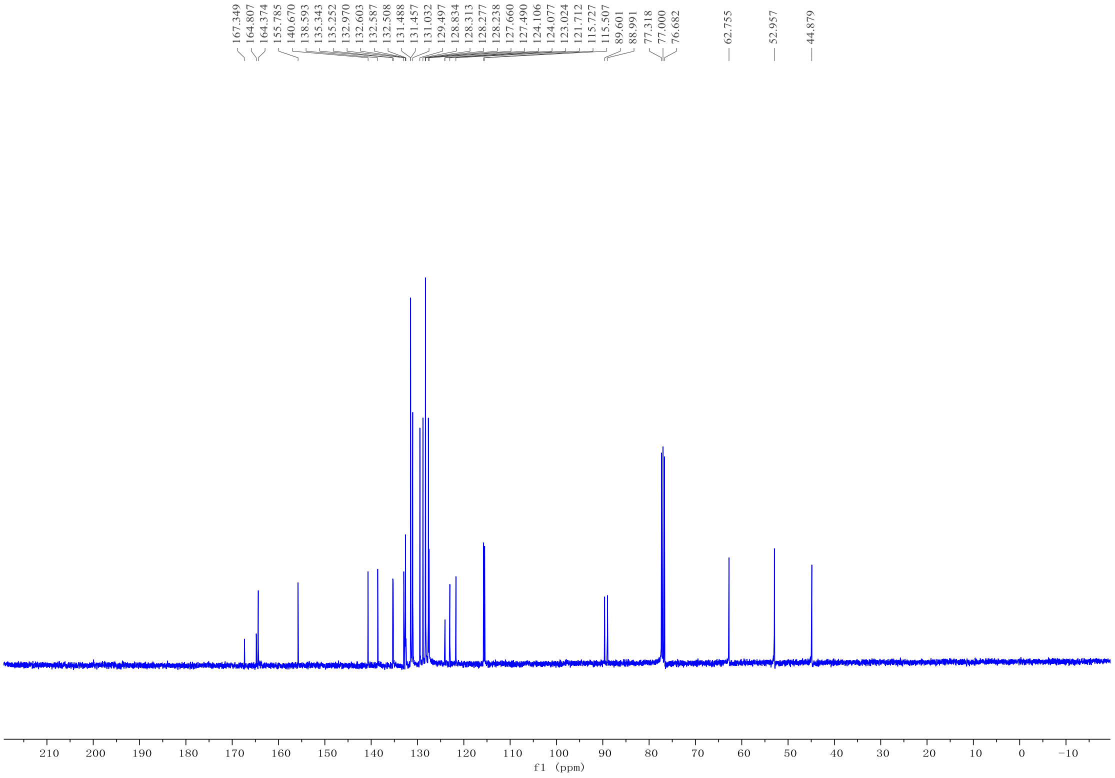

$^{19}$F NMR (376 MHz, CDCl$_3$)

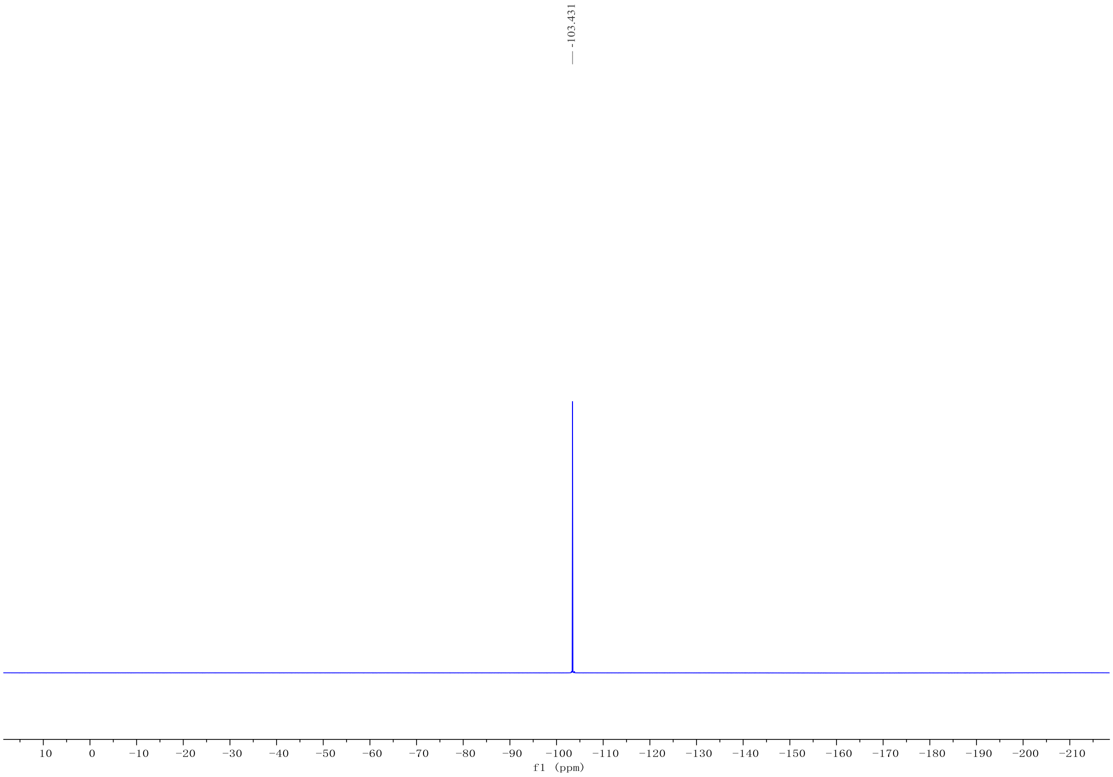


**$^1$H, $^{13}$C NMR and $^{19}$F NMR Spectra for Compound ((*Z*)-12)**:

$^1$H NMR (400 MHz, CDCl$_3$)

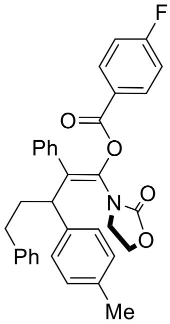


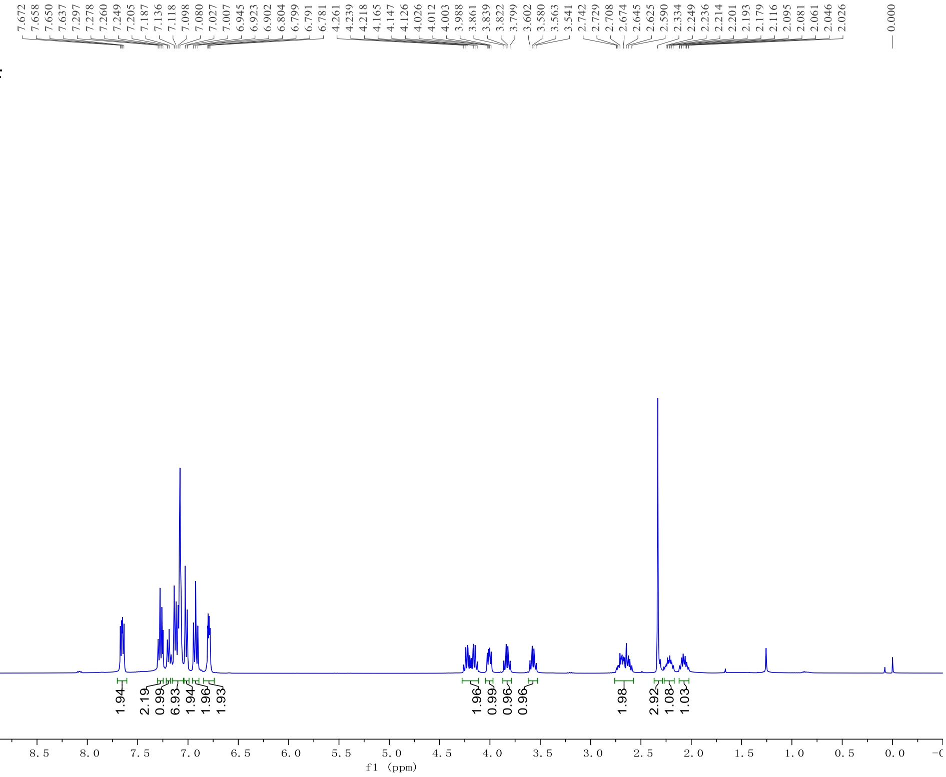

$^{13}C$ NMR (100 MHz, $CDCl_3$)

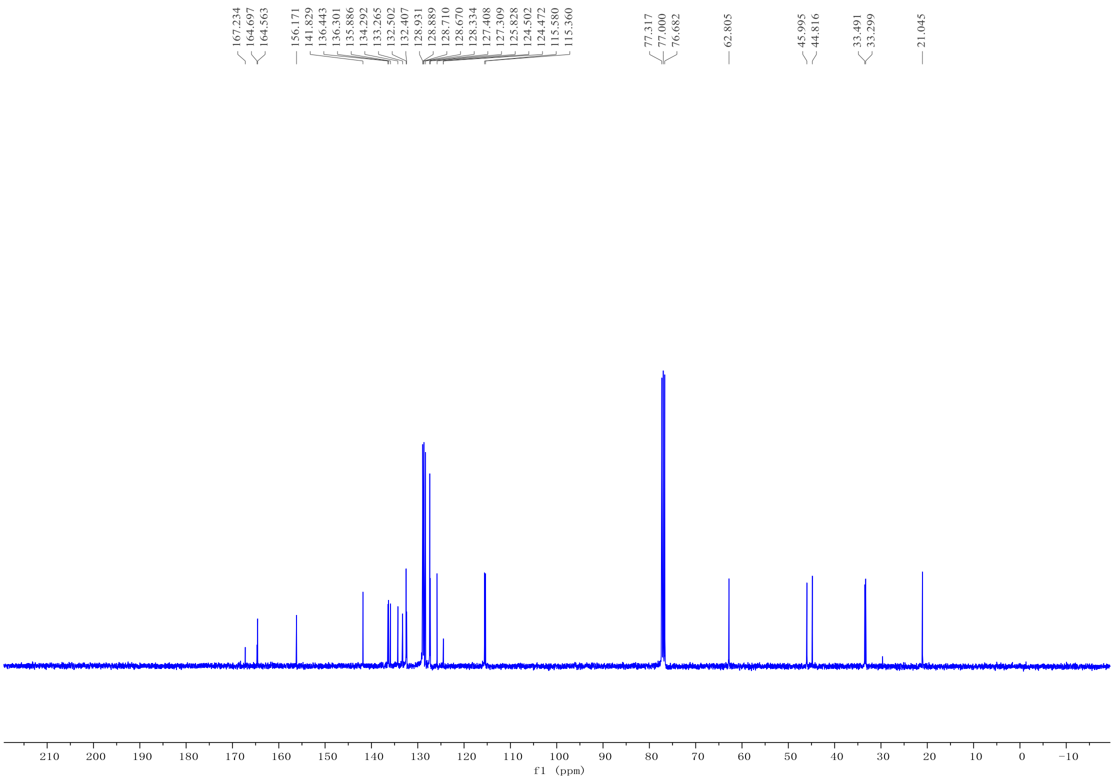


$^{19}F$ NMR (376 MHz, $CDCl_3$)

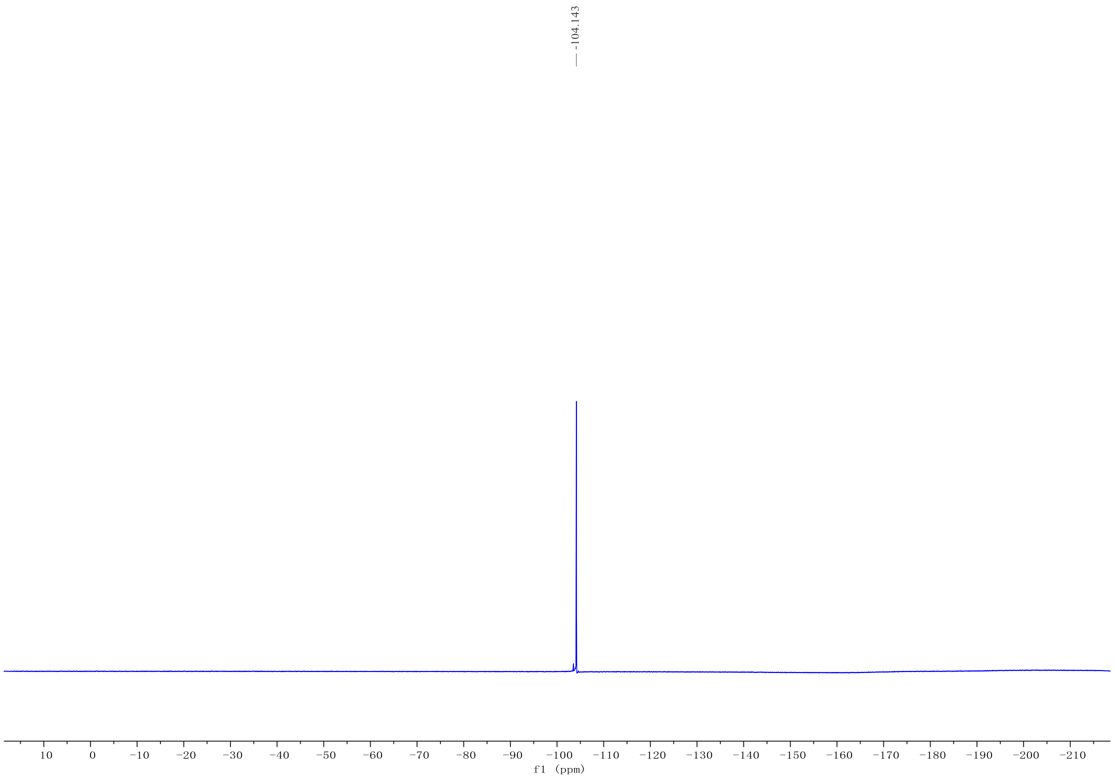

## $^{1}$H, $^{13}$C NMR and $^{19}$F NMR Spectra for Compound ((*Z*)-13):

$^{1}$H NMR (400 MHz, $CDCl_3$)

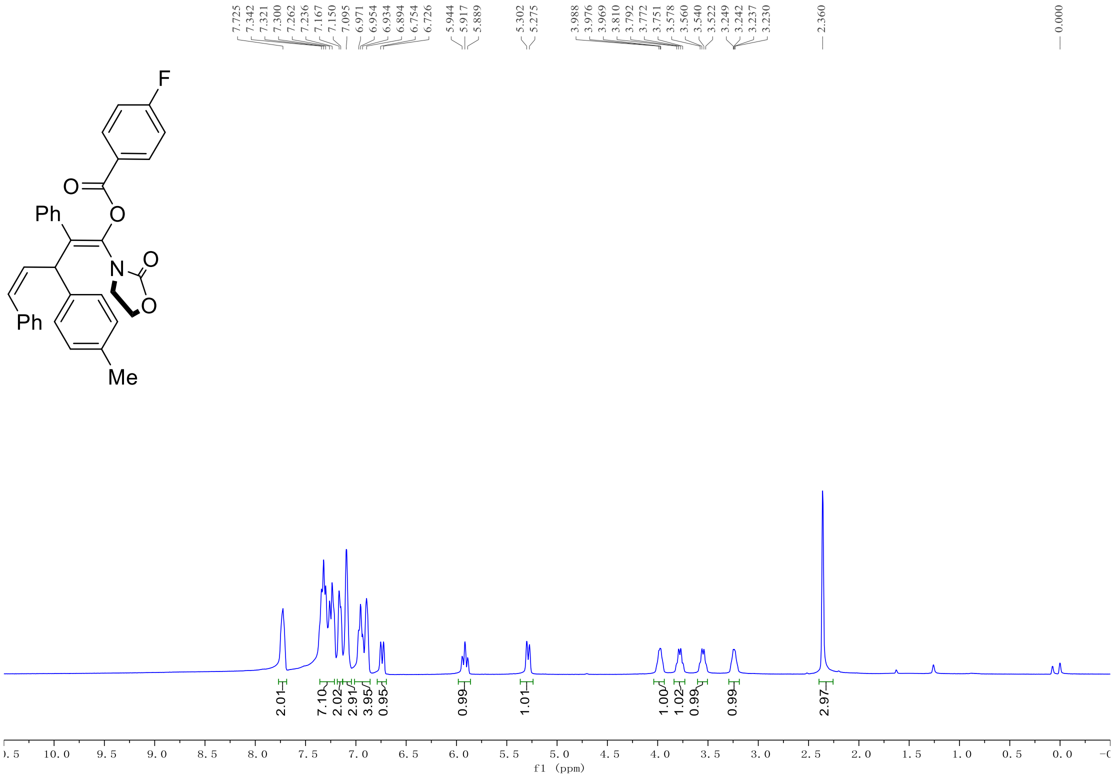


$^{13}$C NMR (100 MHz, $CDCl_3$)

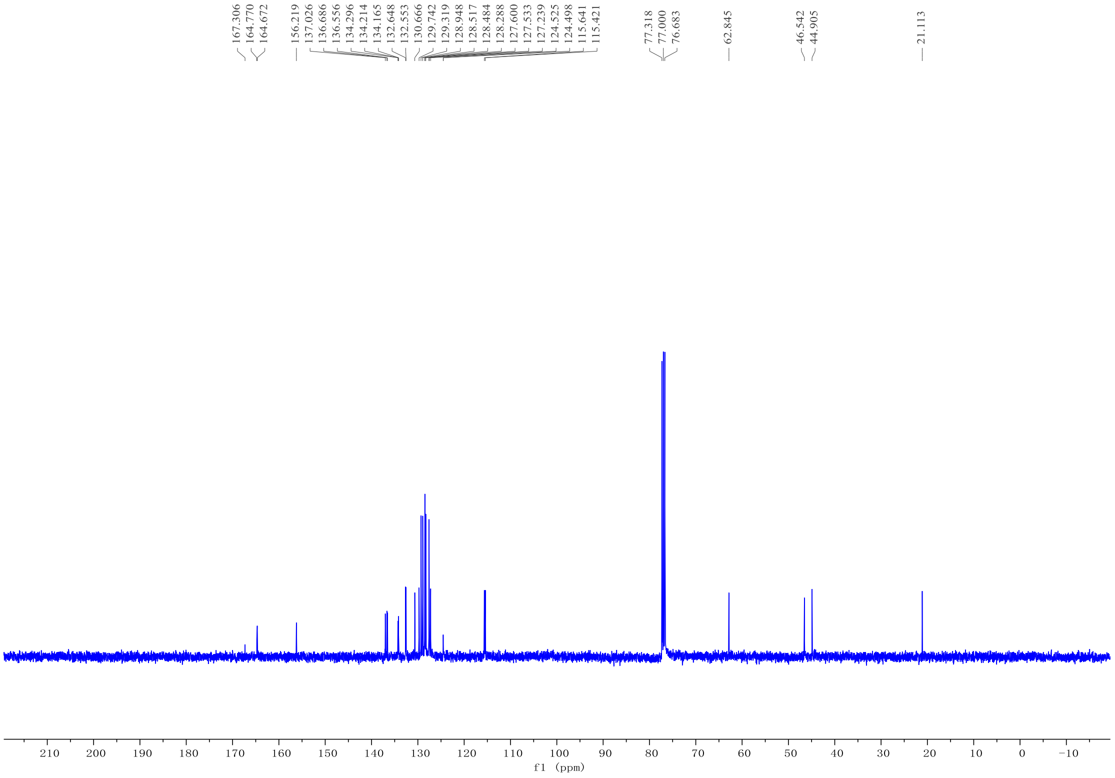

$^{19}F$ NMR (376 MHz, $CDCl_3$)

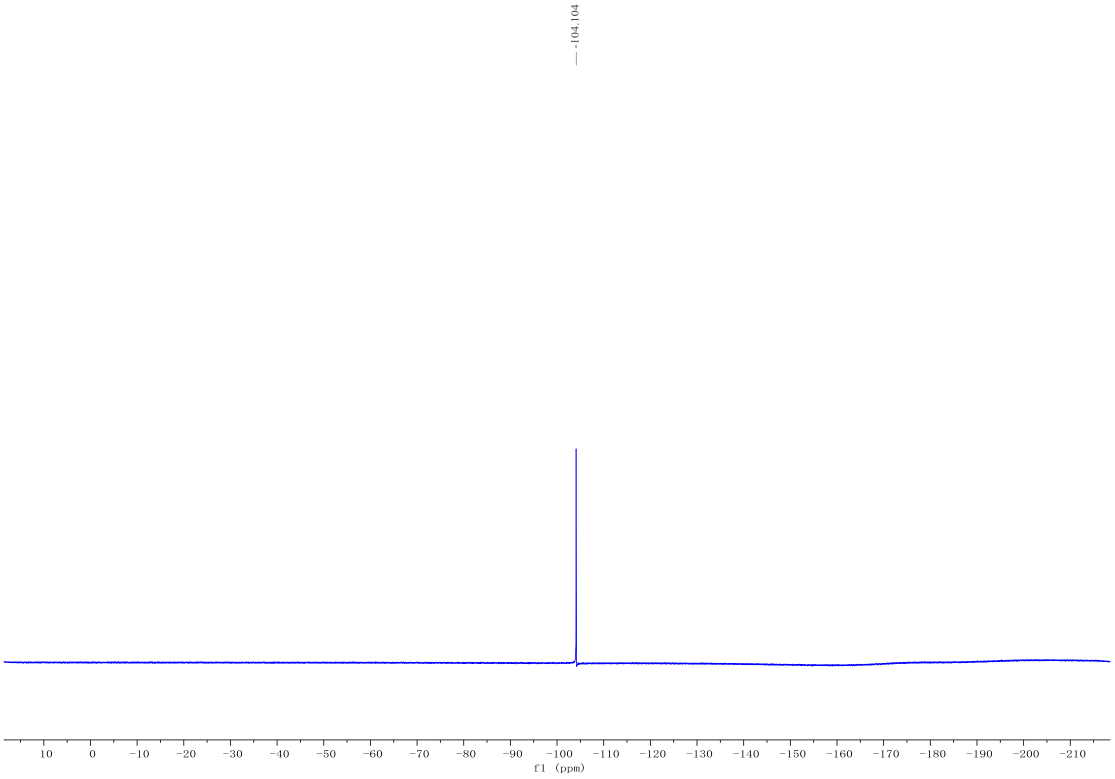

## $^{1}H$ and $^{13}C$ NMR Spectra for Compound (14):

$^{1}H$ NMR (400 MHz, $CDCl_3$)

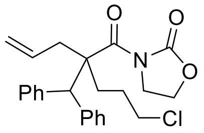

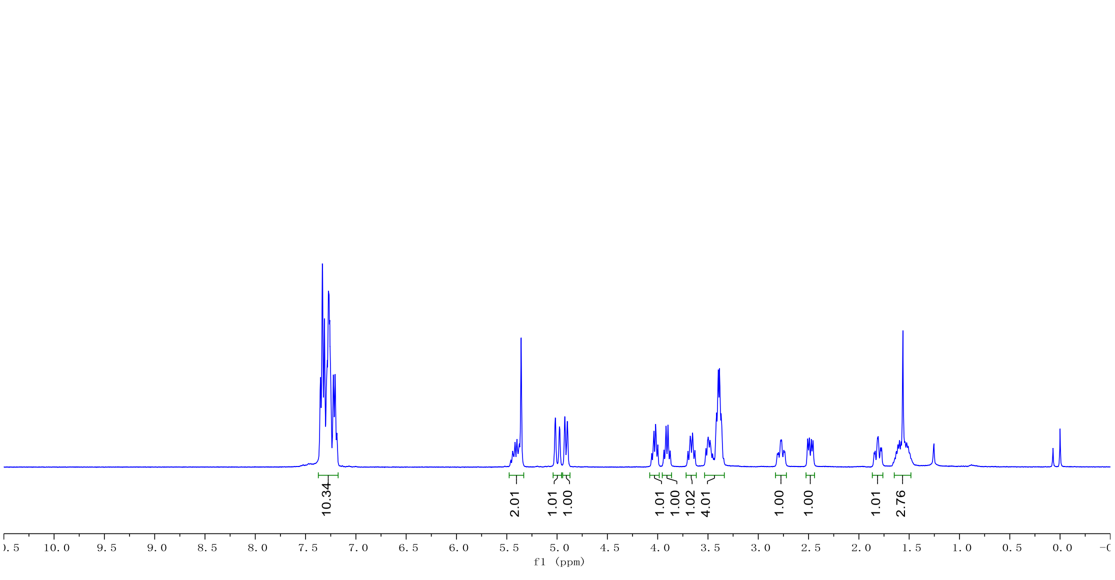

$^{13}C$ NMR (100 MHz, $CDCl_3$)

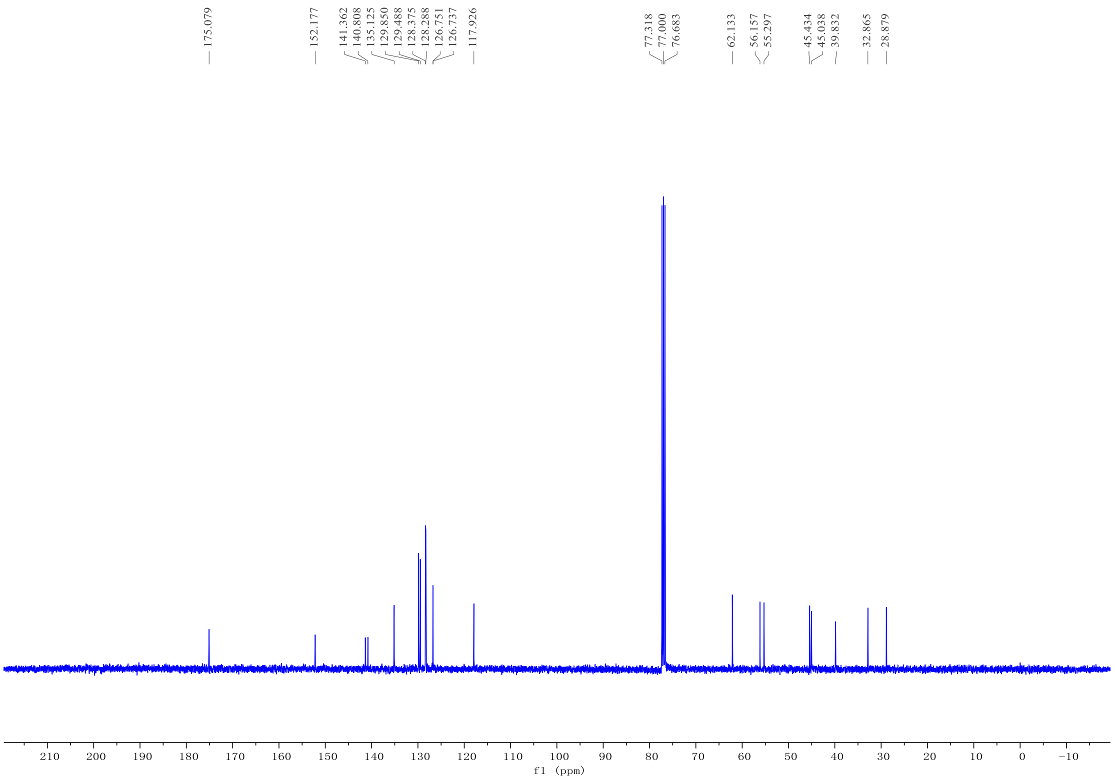


## $^1$H and $^{13}$C NMR Spectra for Compound (16):

$^1H$ NMR (400 MHz, $CDCl_3$)

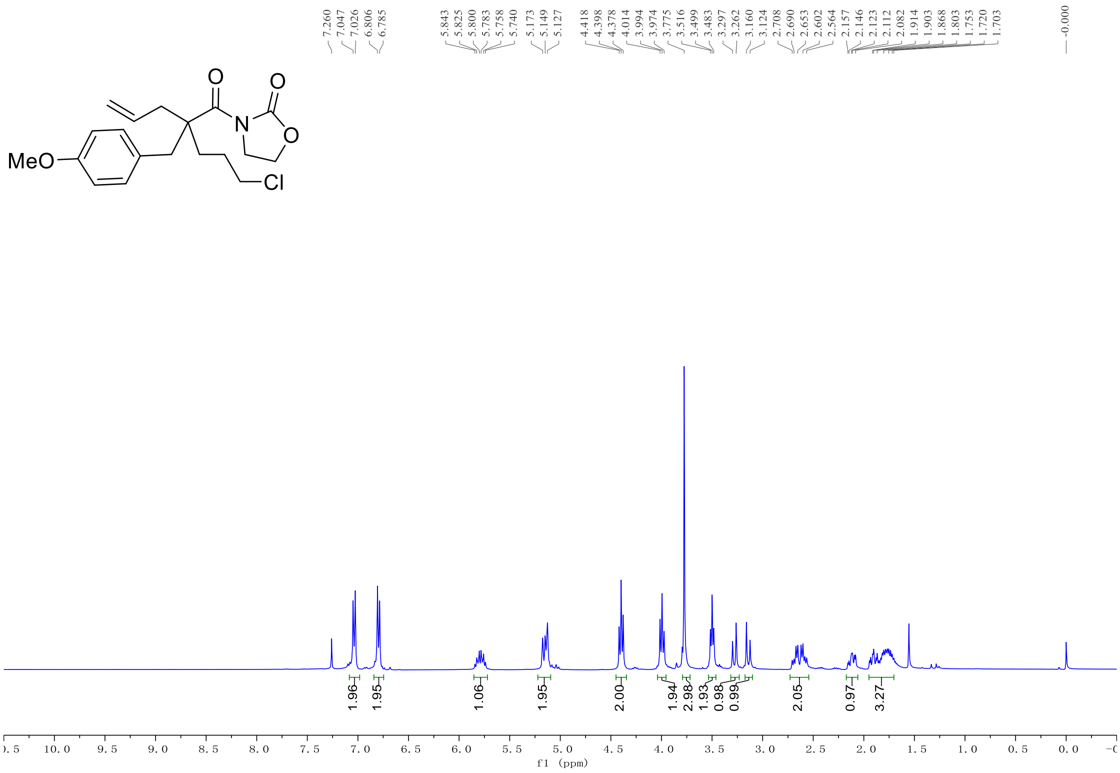

$^{13}$C NMR (100 MHz, CDCl$_3$)

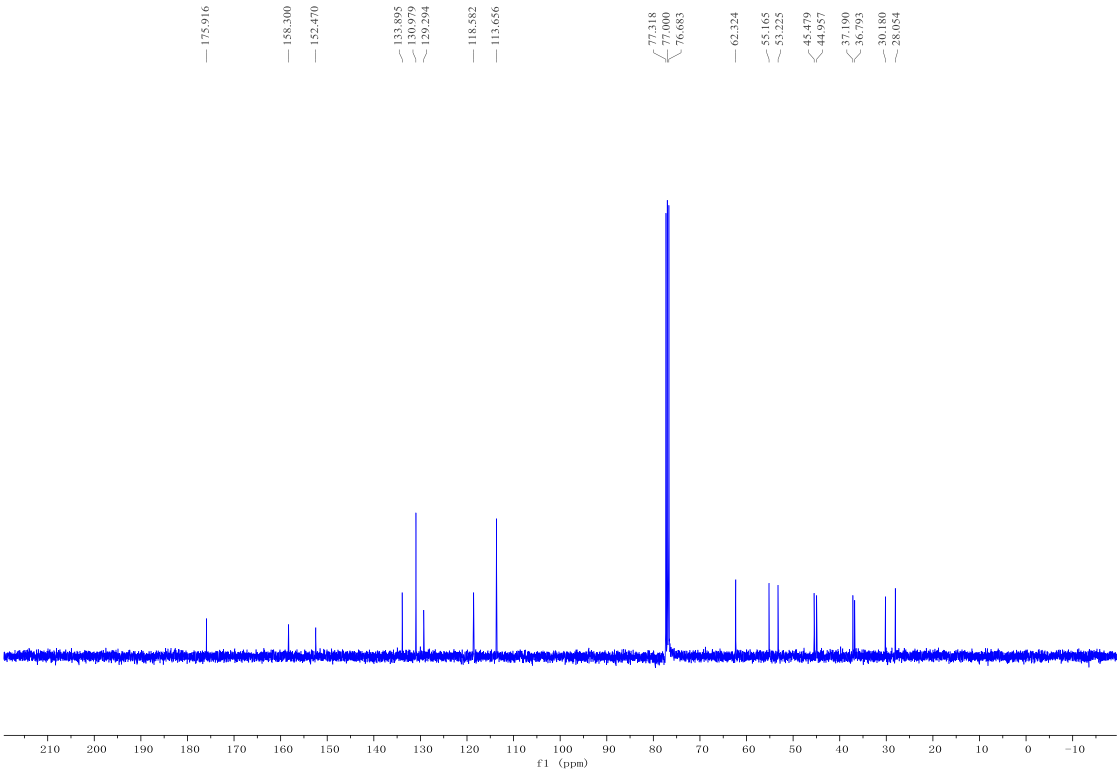

**$^{1}$H and $^{13}$C NMR Spectra for Compound (15)**:

$^{1}$H NMR (400 MHz, CDCl$_3$)

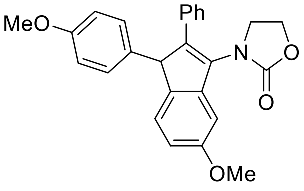


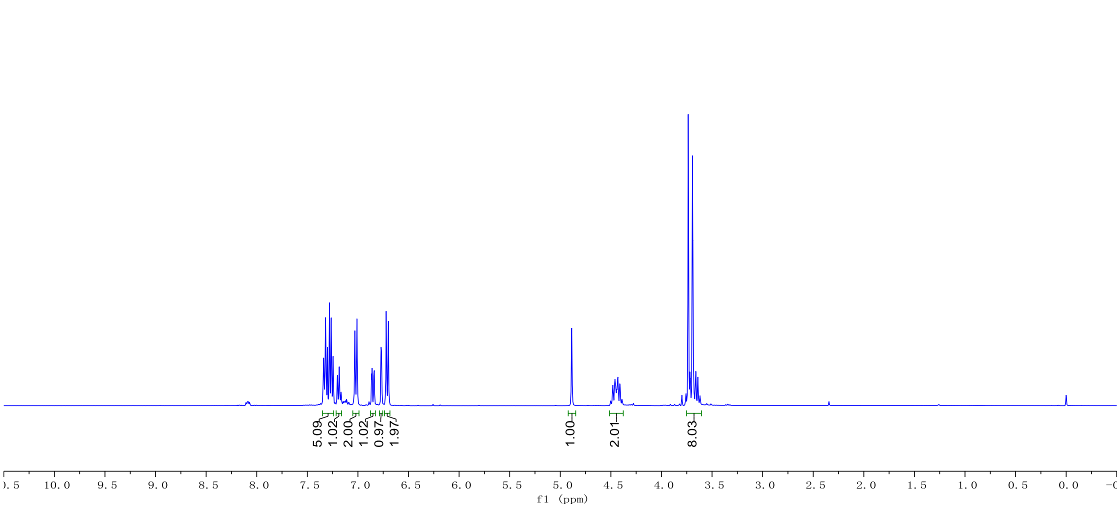

$^{13}$C NMR (100 MHz, $CDCl_3$)

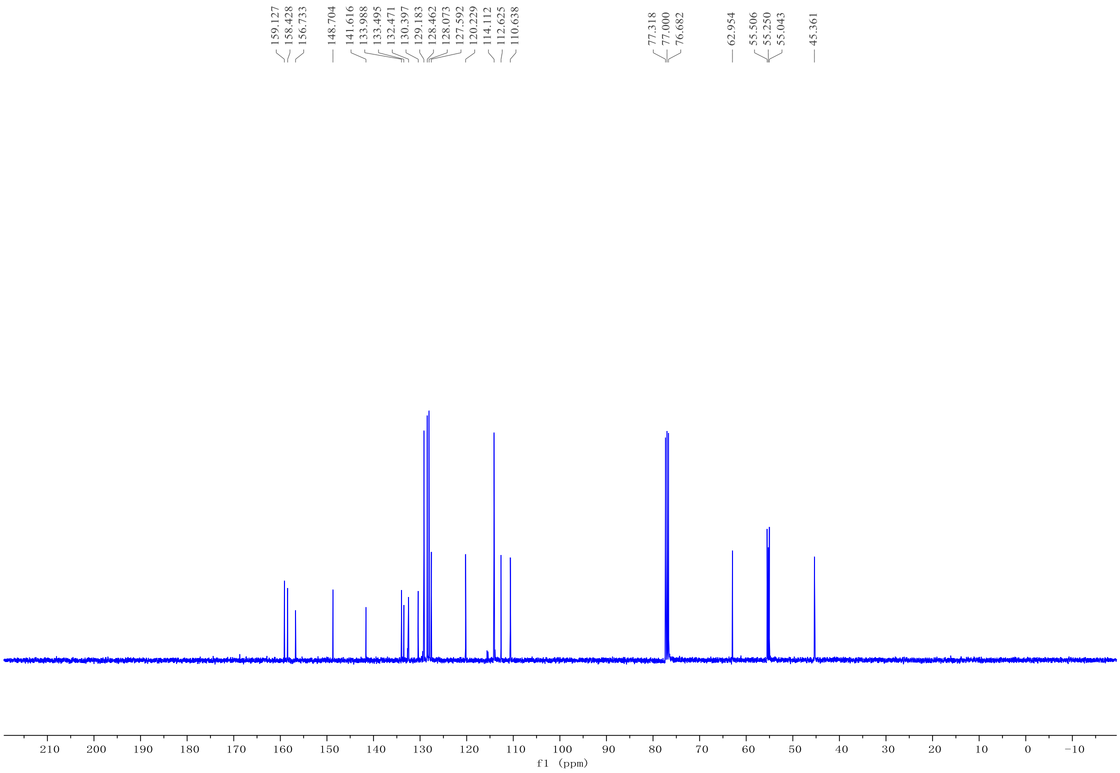


## **$^{1}$H and $^{13}$C NMR Spectra for Compound (*E*)-6vh**:

$^{1}$H NMR (400 MHz, $CDCl_3$)

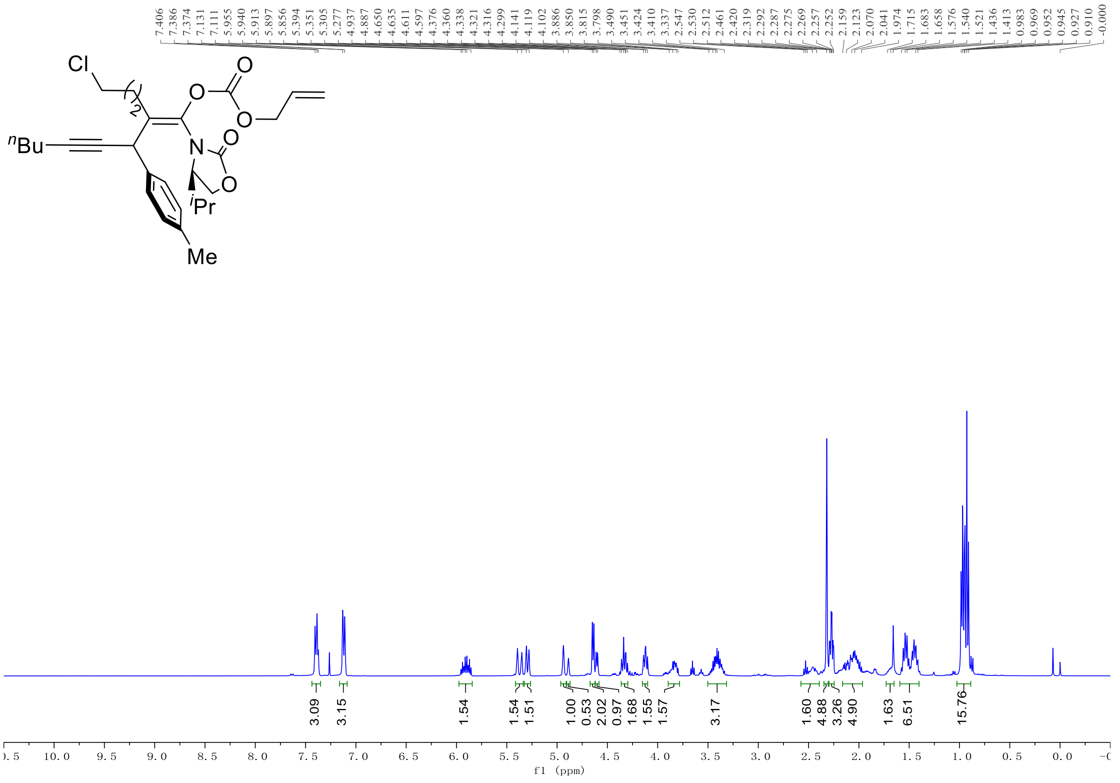

$^{13}$C NMR (100 MHz, $CDCl_3$)

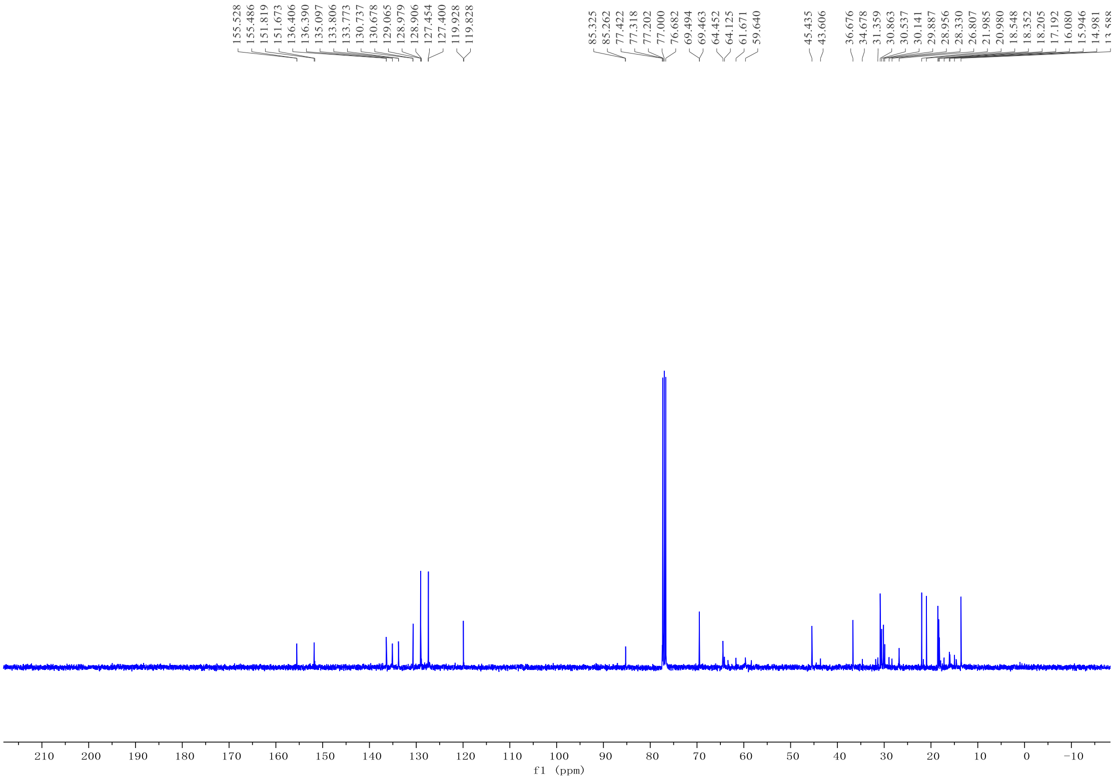


## **$^{1}$H, $^{13}$C NMR and $^{19}$F NMR Spectra for Compound 16(the major isomer)**:

$^{1}$H NMR (400 MHz, $CDCl_3$)

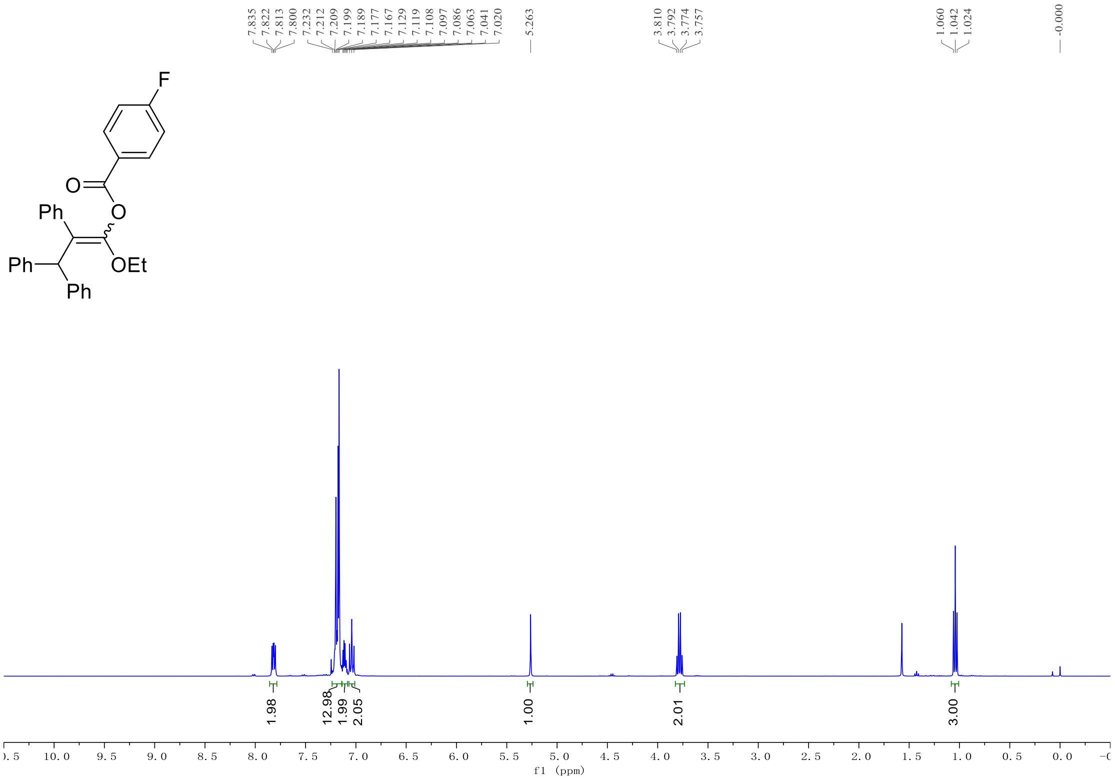

$^{13}C$ NMR (100 MHz, $CDCl_3$)

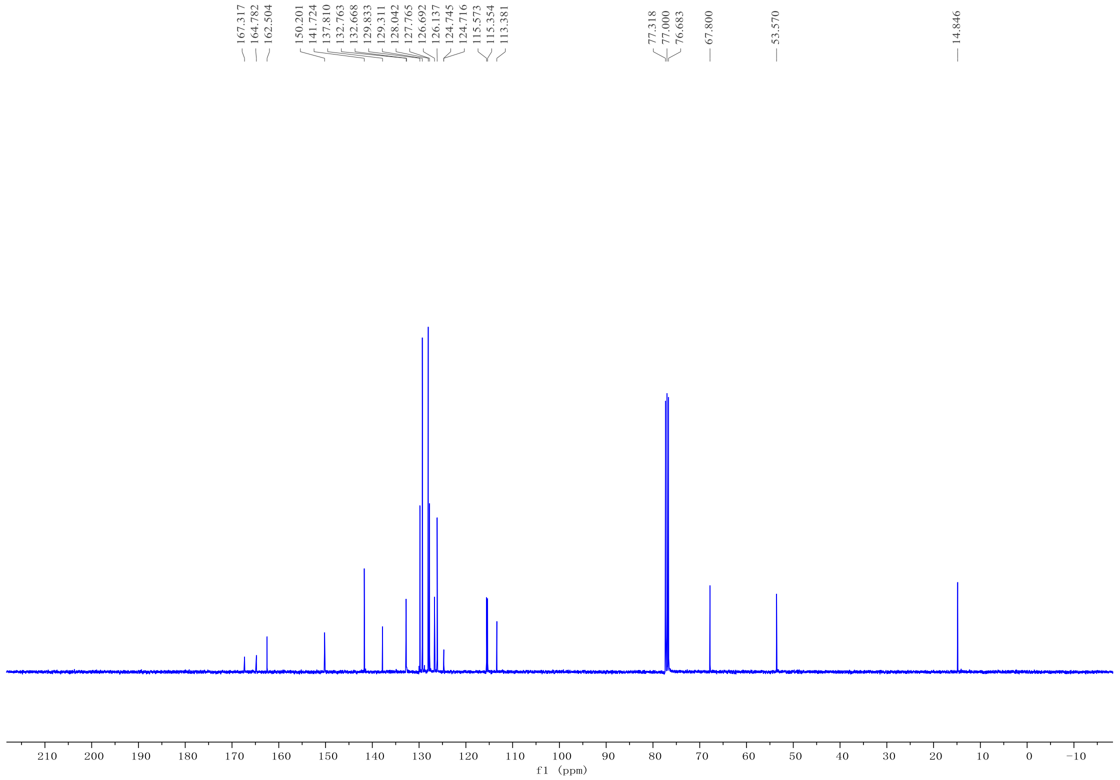


$^{19}F$ NMR (400 MHz, $CDCl_3$)

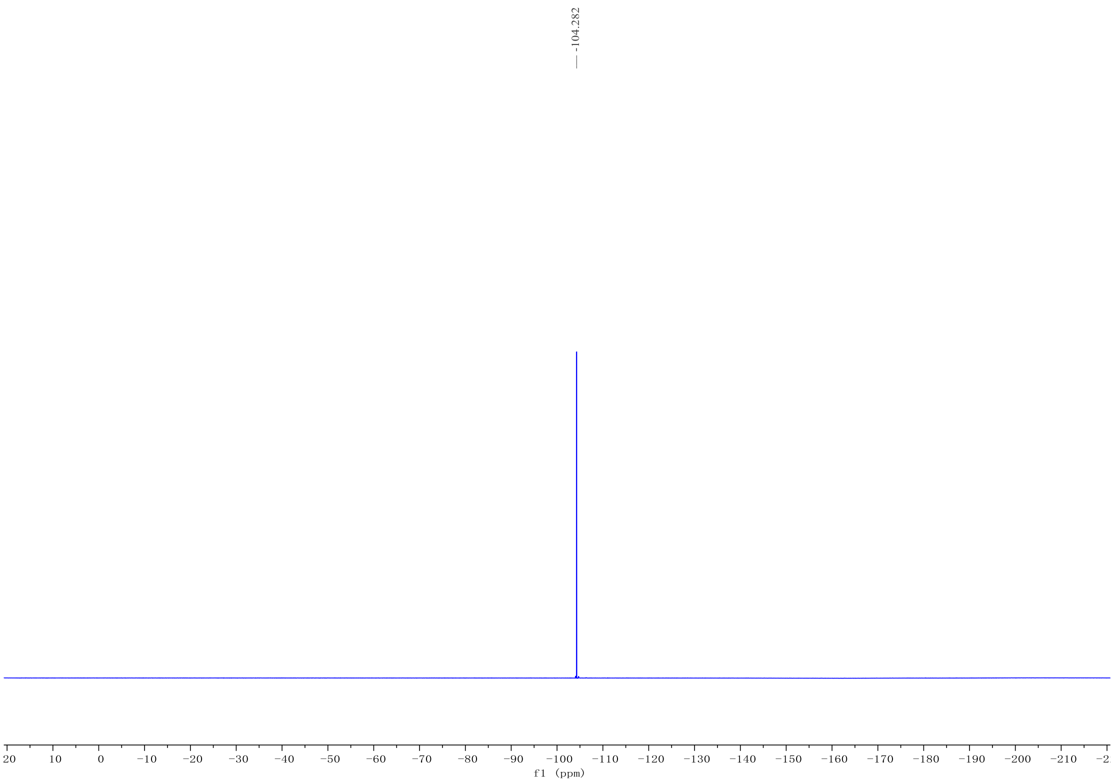

# 16 Computational Data

All density functional theory (DFT) calculations were performed with the Gaussian 16 package.[4] The 3D structures of the optimized species were generated using CYLview.[5] Geometry optimization and vibrational frequency calculations of all stationary points were carried out at M06-2X[6]/6-31G(d,p) level of theory. To get more accurate energies, single-point energies were computed at M06-2X/cc-PVTZ level for all the species along the reaction pathway. The solvent effect of toluene was treated with the polarizable continuum model (PCM)[7] in both geometry optimizations and single-point calculations. Activation free energy barriers here are defined as the free energy difference between the transition state and the lowest-energy stationary point before it along the reaction pathway. Natural population analyses (NPA)[8] were performed at M06-2X/cc-PVTZ level of theory.

## 16.1 Results of the DFT Calculations

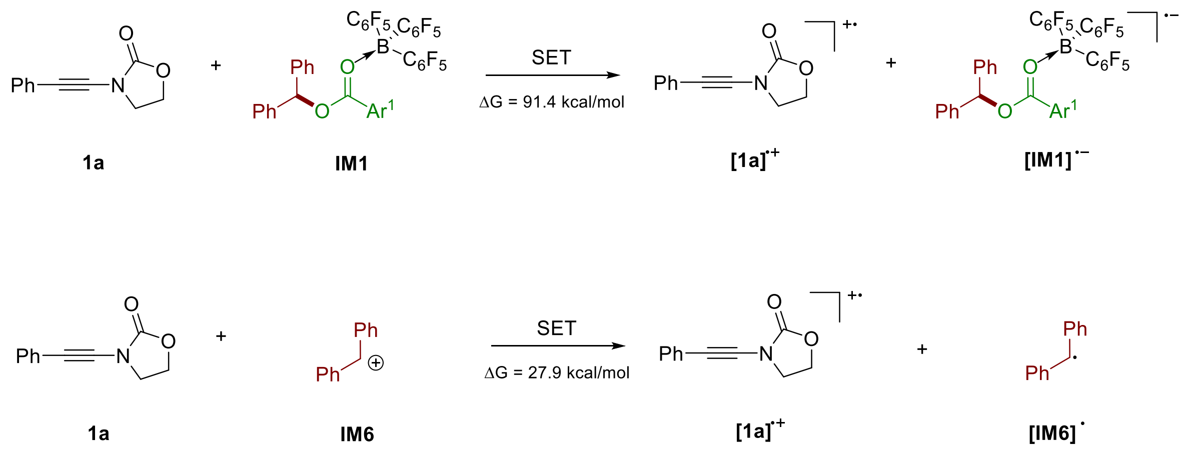


**Figure S1.** Computed reaction energies for SET process: ynamide **1a** and **2a**-B($C_6F_5$)$_3$ adduct **IM1** (or diphenyl methylene cation **IM7**) were defined as electron donor and acceptor, respectively. ($Ar^1$ = *p*-$FC_6H_4$, energies are in kcal/mol).

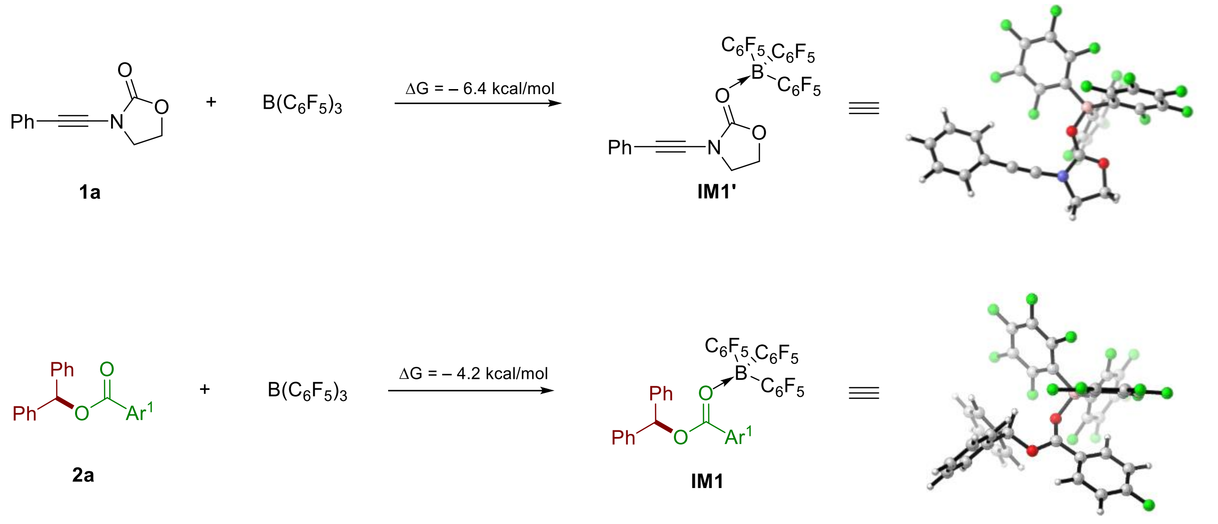


**Figure S2.** Calculated free energy changes and optimized geometries for the complexation of $B(C_6F_5)_3$ with ynamide **1a** or diaryl ester **2a** ($Ar^1$ = *p*-$FC_6H_4$, energies are in kcal/mol).

**Table S1.** The charge distributions on the selected atoms in the intermediate **IM4** and the $B(C_6F_5)_3$-ynamide complex **IM1'** via B-O complexation.

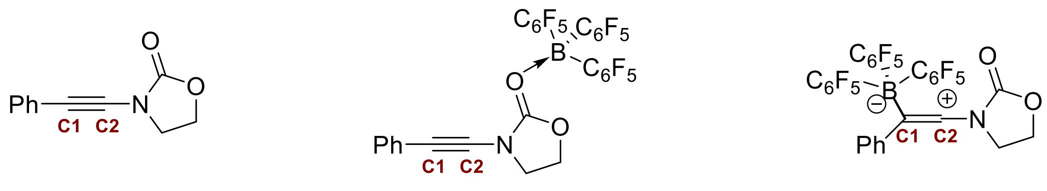


**2a** **IM1'** **IM4**

| Species | C1 | C2 |
|---|---|---|
| **1a** | -0.025 *e* | 0.172 *e* |
| **IM1'** | 0.048 *e* | 0.123 *e* |
| **IM4** | -0.143 *e* | 0.468 *e* |

Table S1 illustrates the charge distributions on the selected atoms in the intermediate **IM4** and the $B(C_6F_5)_3$-ynamide complex **IM1'** (via B-O complexation). The net positive charge on the C2 atom increases from 0.172 e in **1a** to 0.468 e in **IM4**, whereas there is only a slight change in the C1 atom, making it more electrophilic and hence more favorable for the electrophilic attack on the p bond of the alkyne moiety. However, the coordination of $B(C_6F_5)_3$ with the carbonyl oxygen of ynamide **1a** slightly alters the charge on the C2 atom of ynamide, and no nucleophilic addition intermediate via intermediate **IM1'** could be located according to our DFT calculation. These results indicate that the activation of the alkyne pathway through the formation of **IM1'** can be ruled out.

**Table S2.** M06-2X/6-31G(d,p) computed imaginary frequency number (ImF), free energies in toluene (G, in a.u.), M06-2X/cc-pVTZ calculated single point energies in toluene ($E_{sol}$, in a.u.), and corrected free energies ($G_c$, in a.u.) for all stationary points involved.

| Species | ImF | G | $E_{sol}$ | $G_c$ |
|---|---|---|---|---|
| **1a** | 0 | -629.351898 | -629.7182175 | -629.5781142 |
| **2a** | 0 | -1020.825625 | -1021.433702 | -1021.184074 |
| $B(C_6F_5)_3$ | 0 | -2207.409275 | -2208.419006 | -2208.320029 |
| **IM1** | 0 | -3228.24731 | -3229.885516 | -3229.510814 |
| **TS1** | 1 (88.8*i* $cm^{-1}$) | -3228.209218 | -3229.849904 | -3229.473837 |
| **IM2** | 0 | -3228.21282 | -3229.852531 | -3229.477777 |
| **TS2** | 1 (232.2*i* $cm^{-1}$) | -3857.568121 | -3859.593539 | -3859.052447 |
| **IM3** | 0 | -3857.594472 | -3859.62124 | -3859.07773 |
| *E*-**TS3** | 1 (206.5*i* $cm^{-1}$) | -3857.591184 | -3859.616046 | -3859.07404 |
| *Z*-**TS3** | 1 (40.9*i* $cm^{-1}$) | -3857.596777 | -3859.622441 | -3859.07671 |
| (*E*)-**3aa** | 0 | -1650.217037 | -1651.210139 | -1650.792397 |
| (*Z*)-**3aa** | 0 | -1650.218407 | -1651.213853 | -1650.79359 |
| **TS4** | 1 (82.2*i* $cm^{-1}$) | -2836.751429 | -2838.146443 | -2837.879738 |
| **IM4** | 0 | -2836.757482 | -2838.151372 | -2837.885897 |
| **TS5** | 1 (198.9*i* $cm^{-1}$) | -3857.566235 | -3859.587688 | -3859.045276 |
| **IM5** | 0 | -3857.576966 | -3859.602116 | -3859.053776 |
| **TS1‘** | 1 (237.9*i* $cm^{-1}$) | -3857.557337 | -3859.579897 | -3859.039031 |

## 16.2 Cartesian Coordinates of the Optimized Stationary Points

**1a**

C -3.29774600 0.71283400 1.45148400

C -1.92318500 0.76417800 1.25751800

C -1.38730900 0.53150200 -0.01218500

C -2.22908600 0.24692700 -1.09027600

C -3.60383100 0.19310900 -0.90940300

C -4.10979500 0.42826700 0.36226100

H -3.74746200 0.88663800 2.42187700

H -1.26128400 0.98090900 2.08802700

H -1.78745700 0.07074300 -2.06497200

H -4.28414800 0.02431400 -1.72426100

C 0.07687000 0.58049700 -0.27136600

O 0.77798200 0.87694800 0.84041400

O 0.58726500 0.38217300 -1.34877600

C 2.19767200 1.02179800 0.67513100

H 2.54150900 0.25270100 -0.02335300

C 2.82032700 0.79619600 2.03514800

C 3.69231500 -0.26928100 2.24327700

C 2.52413800 1.66088400 3.09180400

C 4.26811800 -0.47257400 3.49692000

H 3.92027800 -0.94438800 1.42280900

C 3.08872300 1.45327700 4.34420000

H 1.85401000 2.49953800 2.92186400

C 3.96471300 0.38617600 4.54800900

H 4.94889000 -1.30352300 3.65042500

H 2.85311800 2.12625900 5.16228700

H 4.40983000 0.22828900 5.52502800

C 2.53347000 2.38908100 0.10647300

C 3.83782900 2.62674800 -0.33172300

C 1.59327800 3.41505500 0.04539200

C 4.19635200 3.87310000 -0.83070800

H 4.57377200 1.82818200 -0.27730300

C 1.95357500 4.66578500 -0.45486700

H 0.57744200 3.23784200 0.38543600

C 3.25263500 4.89792900 -0.89384900

H 5.21153600 4.04562000 -1.17324200

H 1.21342500 5.45813200 -0.50208200

H 3.53066200 5.87099200 -1.28524700

F -5.43547600 0.37860500 0.54544300

**2a**

C 9.96670200 4.27797700 5.19245600

C 11.34942300 4.21865100 5.31863900

C 12.11729500 5.37485100 5.19621200

C 11.49408100 6.59621300 4.94715000

C 10.11207700 6.66691400 4.82161300

C 9.33551100 5.50513200 4.94310300

H 9.36562400 3.37971500 5.28541900

H 11.82917800 3.26480100 5.51240100

H 13.19659400 5.32440300 5.29444000
H 12.08702200 7.49988000 4.85092500
H 9.62086400 7.61460600 4.62928200
C 7.91114200 5.57729500 4.81245900
C 6.71021400 5.66139900 4.70244800
C 4.46968500 4.62848800 4.94272000
C 3.13989500 5.22185500 4.46337000
H 4.49525700 4.46273300 6.02411400
H 4.72957700 3.70530200 4.42401700
H 2.30742200 5.01232100 5.13304600
H 2.89475100 4.89487300 3.44982200
N 5.37128100 5.71439400 4.56935700
C 4.67940800 6.91171200 4.39817000
O 5.14619800 8.00359100 4.24150400
O 3.35679000 6.63840800 4.44317100

$B(C_6F_5)_3$

B -1.30112200 -0.01742000 0.15376900
C -2.08497800 1.34035100 0.15956500
C -3.34797400 1.47655300 -0.42528800
C -1.56800200 2.49817900 0.74935200
C -4.05089200 2.67076300 -0.43813600
C -2.24914900 3.70481500 0.77166600
C -3.49724700 3.79010000 0.16914900
C 0.26520000 -0.01764800 0.08401100
C 0.98231600 0.96553200 -0.60525600
C 1.04079800 -1.00054600 0.70708500
C 2.36582000 0.97714300 -0.68506200
C 2.42590600 -1.01207300 0.66326800
C 3.09043700 -0.01745800 -0.04189600
C -2.08359400 -1.37451500 0.21787600
C -1.62115500 -2.53395400 -0.41279300
C -3.29155200 -1.50851700 0.90991600
C -2.30291300 -3.73982200 -0.37398100
C -3.99221500 -2.70186500 0.98521200
C -3.49448700 -3.82275600 0.33418700
F -4.15860500 4.93705700 0.17364100
F -5.24356700 2.75335100 -1.01977500
F -3.93014300 0.43663800 -1.02529000
F -1.72108400 4.77479600 1.35801400
F -0.37432100 2.47726700 1.34516200
F 0.34150700 1.94585400 -1.24448300
F 3.00115900 1.92691400 -1.36468500
F 4.41308000 -0.01767700 -0.10096700
F 3.11949400 -1.96177200 1.28339700
F 0.45942400 -1.98023000 1.40159000
F -0.48383500 -2.51589300 -1.11015500
F -1.82880000 -4.81115800 -1.00241700
F -4.15525400 -4.96881200 0.38800500
F -5.13019200 -2.78171800 1.66798300
F -3.81868600 -0.46744000 1.55683400

**IM1**

B -0.82571200 -0.40230000 0.35541200

| | | | |
|---|---|---|---|
| C | -0.03923400 | -1.48653500 | 1.28357700 |
| C | -0.03641600 | -1.53933300 | 2.67216500 |
| C | 0.69345200 | -2.48883500 | 0.64584100 |
| C | 0.66786200 | -2.49804400 | 3.39251100 |
| C | 1.41103800 | -3.46139100 | 1.32791300 |
| C | 1.39924500 | -3.46124900 | 2.71592900 |
| C | -1.37520000 | 0.90971900 | 1.14331900 |
| C | -2.67104300 | 1.40932300 | 1.11621800 |
| C | -0.46746800 | 1.68474100 | 1.86154000 |
| C | -3.05105300 | 2.58000500 | 1.76567500 |
| C | -0.79503300 | 2.85787200 | 2.51917100 |
| C | -2.10913700 | 3.30632900 | 2.47528100 |
| C | -1.88769800 | -1.21942500 | -0.55816600 |
| C | -1.78475300 | -1.47376900 | -1.91947800 |
| C | -2.93738500 | -1.86026400 | 0.09254200 |
| C | -2.67581800 | -2.29411400 | -2.60401400 |
| C | -3.84777500 | -2.68409200 | -0.54804900 |
| C | -3.70989400 | -2.90499100 | -1.91320900 |
| F | 0.82734100 | 1.30730600 | 1.91525600 |
| F | 0.12664200 | 3.55376900 | 3.18322500 |
| F | -2.45330200 | 4.43059500 | 3.09448400 |
| F | -4.30708500 | 3.01639100 | 1.69100500 |
| F | -3.62843100 | 0.81058700 | 0.39359300 |
| F | 0.71408400 | -2.54939200 | -0.69198100 |
| F | 2.09610500 | -4.39961100 | 0.67286000 |
| F | 2.07865100 | -4.38497300 | 3.39105300 |
| F | 0.64506200 | -2.49801100 | 4.72558900 |
| F | -0.72372400 | -0.64865000 | 3.39908600 |
| F | -3.09035700 | -1.66950100 | 1.41095300 |
| F | -4.84116800 | -3.26633800 | 0.12200300 |
| F | -4.57066000 | -3.69194800 | -2.55307800 |
| F | -2.54014900 | -2.49763600 | -3.91471700 |
| F | -0.81495600 | -0.90712200 | -2.65750100 |
| C | -0.50267200 | 4.54448700 | -2.30234900 |
| C | 0.32173200 | 3.52797300 | -1.85060300 |
| C | -0.21188400 | 2.25827800 | -1.57927400 |
| C | -1.57704700 | 2.00914800 | -1.77589400 |
| C | -2.40829200 | 3.02107000 | -2.22747700 |
| C | -1.85378700 | 4.26968600 | -2.48181700 |
| H | -0.12562200 | 5.53776600 | -2.51293000 |
| H | 1.37853700 | 3.71056500 | -1.69465200 |
| H | -1.99787800 | 1.03228700 | -1.57315100 |
| H | -3.46799100 | 2.86039700 | -2.38458800 |
| C | 0.67800000 | 1.20501800 | -1.08814000 |
| O | 1.95183000 | 1.44378900 | -1.24178300 |
| O | 0.34529500 | 0.12566500 | -0.55501600 |
| C | 2.90294000 | 0.55266300 | -0.56754700 |
| H | 2.46648500 | 0.33058400 | 0.41157800 |
| C | 4.15330900 | 1.37988100 | -0.38538400 |
| C | 4.19824200 | 2.28579600 | 0.67690900 |
| C | 5.23001500 | 1.28761900 | -1.26560700 |
| C | 5.31449500 | 3.09484200 | 0.85879400 |

H 3.35365400 2.34963600 1.35926300

C 6.34774000 2.09778600 -1.08201700

H 5.19485900 0.57695100 -2.08572100

C 6.39095600 2.99990700 -0.02194700

H 5.34827500 3.79287500 1.68861400

H 7.18662600 2.02182100 -1.76582600

H 7.26527900 3.62633400 0.12180700

C 3.06795400 -0.73704100 -1.33535000

C 3.70480700 -1.79892700 -0.68957700

C 2.60291200 -0.89828700 -2.63729200

C 3.86340500 -3.01731800 -1.33885000

H 4.06942300 -1.67010700 0.32697600

C 2.76070100 -2.12201700 -3.28513900

H 2.10674000 -0.07843100 -3.14715700

C 3.38748200 -3.18167600 -2.63861100

H 4.34538100 -3.84212200 -0.82555100

H 2.38598400 -2.24503400 -4.29591500

H 3.50245800 -4.13551400 -3.14254400

F -2.65014400 5.24551700 -2.91432500

**TS1**

B -1.27090300 0.53294900 0.37212600

C -1.96344200 -0.21701300 1.66680900

C -3.10138800 0.16631600 2.36661800

C -1.34347200 -1.37352400 2.14485400

C -3.56678600 -0.50885500 3.49181000

C -1.80154400 -2.10019900 3.23672100

C -2.91398700 -1.64795600 3.93330000

C -1.94155400 1.97841400 -0.01182000

C -2.30732200 2.40399200 -1.28381000

C -1.99178400 2.97229000 0.96164800

C -2.69400300 3.70901000 -1.57410600

C -2.38267200 4.28009300 0.72601400

C -2.74010400 4.65284800 -0.56184400

C -1.31117000 -0.58911300 -0.81720800

C -0.24267300 -1.35828600 -1.25702900

C -2.54902200 -0.95622900 -1.33686700

C -0.37349400 -2.38927100 -2.18226900

C -2.73025000 -1.97533500 -2.25778100

C -1.62609900 -2.70273000 -2.68295000

F -1.58892900 2.69064100 2.21504200

F -2.39875300 5.18149100 1.71143500

F -3.10340100 5.90687000 -0.82311400

F -2.99761200 4.06167300 -2.82399900

F -2.24310600 1.58118500 -2.33935100

F -0.24171000 -1.84176800 1.54311100

F -1.19014400 -3.22075400 3.62934500

F -3.35653100 -2.31062800 4.99993000

F -4.62022600 -0.04616100 4.17171300

F -3.81729400 1.23887700 2.00893900

F -3.64481700 -0.29134300 -0.93759800

F -3.94279000 -2.27290200 -2.72816600

F -1.77132300 -3.68997500 -3.56582300

| | | | |
|---|---|---|---|
| F | 0.69493300 | -3.08550900 | -2.57965800 |
| F | 1.00771000 | -1.12305000 | -0.81974300 |
| C | 0.81342500 | 4.72185900 | -1.75053200 |
| C | 0.90253800 | 3.98657300 | -0.57570800 |
| C | 0.85722000 | 2.59054000 | -0.61478800 |
| C | 0.77530000 | 1.92593200 | -1.83898300 |
| C | 0.71001900 | 2.64966900 | -3.02372700 |
| C | 0.71389400 | 4.03489300 | -2.95385000 |
| H | 0.81759200 | 5.80564800 | -1.75250800 |
| H | 0.98373200 | 4.48759800 | 0.38342800 |
| H | 0.77582400 | 0.84384000 | -1.86286800 |
| H | 0.64481600 | 2.16159400 | -3.98884900 |
| C | 0.90021400 | 1.84019600 | 0.67961400 |
| O | 1.68036600 | 2.22020800 | 1.55666400 |
| O | 0.15064200 | 0.79483600 | 0.86573100 |
| C | 1.70357000 | 1.23260700 | 3.64787900 |
| H | 2.58765300 | 1.84220600 | 3.50161600 |
| C | 0.61225700 | 1.87154200 | 4.30354800 |
| C | 0.80774100 | 3.19615200 | 4.75718900 |
| C | -0.65465600 | 1.26591100 | 4.46088900 |
| C | -0.21563400 | 3.87507000 | 5.39117900 |
| H | 1.77207600 | 3.67063000 | 4.60199600 |
| C | -1.67632700 | 1.95279300 | 5.09442700 |
| H | -0.83702100 | 0.28377000 | 4.04415100 |
| C | -1.45497100 | 3.25004700 | 5.56017000 |
| H | -0.06469600 | 4.88879300 | 5.74355900 |
| H | -2.65671600 | 1.49864200 | 5.19449800 |
| H | -2.26335200 | 3.78899300 | 6.04368100 |
| C | 1.85381800 | -0.17550300 | 3.38099500 |
| C | 2.68084800 | -0.57980800 | 2.31353300 |
| C | 1.29815200 | -1.15049500 | 4.23311800 |
| C | 2.89842500 | -1.92370100 | 2.07285200 |
| H | 3.09864100 | 0.17923500 | 1.66075200 |
| C | 1.56636000 | -2.49476400 | 4.01408800 |
| H | 0.72674000 | -0.84901600 | 5.10417100 |
| C | 2.34694100 | -2.88138800 | 2.92748800 |
| H | 3.50099700 | -2.23138700 | 1.22584600 |
| H | 1.15975500 | -3.23992000 | 4.68705400 |
| H | 2.53566700 | -3.93488800 | 2.74901400 |
| F | 0.63053700 | 4.73920600 | -4.09012000 |

**IM2**

| | | | |
|---|---|---|---|
| B | -1.06844200 | 0.68411900 | 0.43127800 |
| C | -1.74423900 | -0.08874200 | 1.73128700 |
| C | -2.92358700 | 0.24649100 | 2.39072300 |
| C | -1.17271700 | -1.28963800 | 2.15529500 |
| C | -3.47629400 | -0.52081100 | 3.41316300 |
| C | -1.70734600 | -2.10354300 | 3.14707200 |
| C | -2.86971800 | -1.70675000 | 3.79408900 |
| C | -1.78378500 | 2.11452900 | 0.07528200 |
| C | -2.23026600 | 2.53869400 | -1.17081900 |
| C | -1.79675700 | 3.09913100 | 1.05746900 |
| C | -2.63369400 | 3.84430800 | -1.43409500 |

C -2.20192800 4.40705800 0.84927500

C -2.62237400 4.78533400 -0.41805900

C -1.16271800 -0.44724300 -0.75115300

C -0.10985000 -1.21640400 -1.22682700

C -2.41978800 -0.82973700 -1.21009200

C -0.27289400 -2.25963700 -2.13264800

C -2.63368400 -1.86150400 -2.11002100

C -1.54417600 -2.58676000 -2.57472200

F -1.36960600 2.79640500 2.29712800

F -2.17958100 5.30034900 1.84328100

F -2.99745700 6.04117800 -0.65581600

F -3.00150800 4.20257100 -2.66546700

F -2.24276100 1.71302300 -2.22617700

F -0.05098900 -1.74004800 1.57397400

F -1.12740300 -3.26076900 3.47704300

F -3.39814400 -2.45989100 4.75685000

F -4.58017100 -0.11197000 4.04459000

F -3.61141500 1.35209500 2.08386100

F -3.50347100 -0.17370900 -0.76529400

F -3.86372700 -2.17384000 -2.52197000

F -1.72097400 -3.58590500 -3.43830300

F 0.78203000 -2.95370000 -2.56809500

F 1.15791600 -0.96425000 -0.85010600

C 0.64415000 4.74795800 -1.98598700

C 0.91797200 4.00539100 -0.84524000

C 0.93170600 2.60799600 -0.89549000

C 0.73878500 1.95576500 -2.11215000

C 0.48827000 2.68899700 -3.26807800

C 0.42381500 4.06992300 -3.17809900

H 0.59702400 5.83081200 -1.96931500

H 1.10313200 4.50524700 0.10009400

H 0.79567300 0.87664200 -2.16328900

H 0.33002200 2.20662900 -4.22534400

C 1.20372500 1.87578800 0.38861400

O 2.21289500 2.16174500 1.02127500

O 0.36777700 0.96668500 0.84046300

C 1.40899300 1.00913500 3.61820600

H 2.05191000 1.64337600 3.00957800

C 0.38237000 1.69122000 4.31872500

C 0.51971000 3.09494500 4.45002500

C -0.80556100 1.06873700 4.76930200

C -0.46495400 3.83870500 5.07223000

H 1.41040900 3.57504000 4.05581100

C -1.80436500 1.82514800 5.35256000

H -0.96316500 0.01369200 4.58305900

C -1.62758100 3.20282300 5.51545500

H -0.35430900 4.91096300 5.18332200

H -2.73635800 1.35627100 5.65029100

H -2.41904700 3.79164200 5.96782000

C 1.70867600 -0.37249300 3.54794700

C 2.53095800 -0.77579600 2.46590100

C 1.25796900 -1.33796200 4.48256600

C 2.84487400 -2.11191000 2.29338800

H 2.85755000 -0.01992100 1.75760300

C 1.59070000 -2.66587400 4.30679400

H 0.70616300 -1.02666800 5.36267100

C 2.36901100 -3.05166600 3.20751700

H 3.44861100 -2.42638000 1.45016800

H 1.25636500 -3.40933300 5.02067400

H 2.61674600 -4.10015400 3.07574900

F 0.15761700 4.78061200 -4.28333700

**TS2**

B -0.99343300 0.38989200 0.12257200

C -1.64399100 -0.34135600 1.45327500

C -2.69096400 0.10024600 2.25565700

C -1.09118200 -1.56130600 1.84154200

C -3.11547400 -0.57853000 3.39414700

C -1.50298700 -2.28557600 2.95152100

C -2.50678900 -1.76901400 3.75574900

C -1.55286900 1.90659600 -0.16344400

C -1.92536300 2.43746600 -1.39309800

C -1.46818000 2.84531300 0.85730300

C -2.19479400 3.78860300 -1.59151000

C -1.73662100 4.19635800 0.71966400

C -2.10743200 4.67428500 -0.52888900

C -1.25217400 -0.65878500 -1.10469900

C -0.31526400 -1.50411600 -1.68215300

C -2.56101200 -0.85554800 -1.53619300

C -0.63616400 -2.45275000 -2.64912700

C -2.92971700 -1.78706500 -2.49286700

C -1.95224700 -2.59880100 -3.05361500

F -1.03835500 2.44831800 2.07010400

F -1.62372200 5.02875300 1.75780600

F -2.36096900 5.96906300 -0.70821400

F -2.51938400 4.24183300 -2.80342300

F -1.98629600 1.67343400 -2.49210700

F -0.07768600 -2.09194600 1.13885300

F -0.93069000 -3.45123100 3.26349800

F -2.88304200 -2.40458000 4.86675600

F -4.09223200 -0.08209300 4.16112500

F -3.35635200 1.22939000 1.98057200

F -3.53927800 -0.09850300 -1.01346200

F -4.20220000 -1.92041400 -2.87306600

F -2.27844000 -3.50534800 -3.97463500

F 0.31187900 -3.23032900 -3.17944800

F 0.98477900 -1.43263400 -1.34791400

C 1.41753600 4.52059800 -1.91915800

C 1.43934500 3.75542300 -0.75936100

C 1.27669000 2.36920400 -0.83180100

C 1.12335200 1.74289600 -2.07051200

C 1.12009100 2.49519000 -3.23848300

C 1.25547800 3.87256200 -3.13673000

H 1.51473500 5.59981200 -1.89660900

H 1.55082400 4.23153100 0.20965800

| | | | |
|---|---|---|---|
| H | 1.01846600 | 0.66521400 | -2.11141000 |
| H | 1.00323400 | 2.03868700 | -4.21420000 |
| C | 1.23357000 | 1.57726100 | 0.43582800 |
| O | 1.91192300 | 1.91081200 | 1.40499800 |
| O | 0.48836900 | 0.50132600 | 0.47713400 |
| F | 1.23543200 | 4.60685100 | -4.25757600 |
| C | 0.25630700 | 1.37091700 | 4.35673700 |
| H | 0.65272700 | 1.78260300 | 3.42769500 |
| C | -0.89325500 | 2.08014500 | 4.89628000 |
| C | -0.99652400 | 3.46339800 | 4.63442300 |
| C | -1.93056300 | 1.44121600 | 5.59828800 |
| C | -2.08059700 | 4.18671400 | 5.10026700 |
| H | -0.21225700 | 3.95918800 | 4.06928100 |
| C | -3.02289800 | 2.17177900 | 6.05048800 |
| H | -1.92356800 | 0.36655700 | 5.73485900 |
| C | -3.09223600 | 3.54254400 | 5.81844600 |
| H | -2.14865300 | 5.24840800 | 4.89053600 |
| H | -3.82949100 | 1.66218600 | 6.56533300 |
| H | -3.94734900 | 4.10832300 | 6.17387400 |
| C | 0.61099600 | -0.01206500 | 4.58820200 |
| C | 1.30778400 | -0.66928300 | 3.55290200 |
| C | 0.33282300 | -0.70702500 | 5.78346300 |
| C | 1.67647300 | -1.99834300 | 3.69301800 |
| H | 1.50808700 | -0.12874100 | 2.62980800 |
| C | 0.71089000 | -2.03584800 | 5.91512300 |
| H | -0.14062000 | -0.19918900 | 6.61614900 |
| C | 1.37659200 | -2.68101900 | 4.87240500 |
| H | 2.17532700 | -2.50900600 | 2.87712400 |
| H | 0.49454900 | -2.56767100 | 6.83508900 |
| H | 1.65798300 | -3.72375900 | 4.97801100 |
| C | 0.41202800 | 2.63414100 | 7.71796500 |
| C | 0.08133300 | 2.23270700 | 9.00778600 |
| C | 0.80873200 | 1.22766600 | 9.64209500 |
| C | 1.87945500 | 0.62079500 | 8.98701800 |
| C | 2.21859300 | 1.00876700 | 7.69714300 |
| C | 1.48472100 | 2.01970300 | 7.05696100 |
| H | -0.15228600 | 3.41652200 | 7.22118800 |
| H | -0.74902500 | 2.70903400 | 9.51784700 |
| H | 0.54397800 | 0.91957500 | 10.64799600 |
| H | 2.44862600 | -0.16049700 | 9.47918600 |
| H | 3.03779100 | 0.53048400 | 7.17061100 |
| C | 1.81339300 | 2.34946900 | 5.69744200 |
| C | 2.43513400 | 2.78253600 | 4.72751500 |
| C | 4.17830500 | 2.46778500 | 3.00609400 |
| C | 4.39654200 | 3.33380800 | 1.74742500 |
| H | 3.88942900 | 1.44874500 | 2.75094600 |
| H | 5.03484200 | 2.47472200 | 3.68101400 |
| H | 4.20977200 | 2.76925300 | 0.83551600 |
| H | 5.37980700 | 3.80230600 | 1.72377400 |
| N | 3.04544600 | 3.16017600 | 3.62835500 |
| C | 2.57379100 | 4.22358400 | 2.82376000 |
| O | 1.59352600 | 4.87814300 | 3.03488000 |

O 3.41784700 4.38344000 1.81068700

**IM3**

B -0.81701600 0.48043400 0.21334200

C -1.46556700 -0.20706400 1.56000900

C -2.52713300 0.26418900 2.32460000

C -0.98331600 -1.45883800 1.94022200

C -3.07017800 -0.44180900 3.39373900

C -1.46488500 -2.17760800 3.02451700

C -2.53587400 -1.66985600 3.74489200

C -1.27718700 2.03565800 -0.02409200

C -1.71102000 2.59903400 -1.21810600

C -1.04916500 2.95258700 0.99691600

C -1.91052000 3.96555600 -1.38864200

C -1.24884400 4.31873700 0.88410500

C -1.68465400 4.83058000 -0.33060100

C -1.22416400 -0.54765000 -0.99175000

C -0.39488300 -1.48375200 -1.59282800

C -2.56691400 -0.64323800 -1.34833400

C -0.84374900 -2.41960500 -2.52034400

C -3.06179800 -1.55782500 -2.26324600

C -2.18667400 -2.46000600 -2.85473900

F -0.55495600 2.51462400 2.17305900

F -1.01719700 5.13852500 1.91017500

F -1.86971400 6.14120100 -0.47999100

F -2.29450000 4.45130900 -2.57020100

F -1.90468800 1.85010800 -2.31233600

F -0.01451200 -2.04669900 1.21772900

F -0.92856400 -3.35148800 3.36667700

F -3.02338600 -2.34587000 4.78581100

F -4.09887600 0.05663500 4.08371800

F -3.10472000 1.44464800 2.05958400

F -3.45150500 0.19572700 -0.78574100

F -4.35950300 -1.59141300 -2.57289000

F -2.63594600 -3.35223800 -3.73698000

F 0.00705500 -3.28523700 -3.07829000

F 0.92266700 -1.51787400 -1.32437200

C 1.52208000 4.34252800 -2.19808700

C 1.62869800 3.62453100 -1.01364900

C 1.45245600 2.23777800 -1.01057700

C 1.20320700 1.56308300 -2.20683100

C 1.11552900 2.26700000 -3.40207200

C 1.26231900 3.64595100 -3.37120600

H 1.62165300 5.42144100 -2.22702600

H 1.80653900 4.14682600 -0.07863100

H 1.09236400 0.48599100 -2.19644800

H 0.92263000 1.77012900 -4.34548100

C 1.50112400 1.50285700 0.29367500

O 2.30743600 1.83488800 1.16007000

O 0.69177000 0.49405700 0.48261400

F 1.15849000 4.33294300 -4.51640300

C 2.16453200 1.53491500 4.85072900

C 2.73246200 2.34617600 4.01718500

| | | | |
|---|---|---|---|
| C | 4.56422900 | 2.82995700 | 2.46667300 |
| C | 4.44508400 | 3.80757600 | 1.28792600 |
| H | 4.57026600 | 1.79041300 | 2.14638100 |
| H | 5.40145000 | 3.05547500 | 3.12801300 |
| H | 4.12671500 | 3.29760600 | 0.37977000 |
| H | 5.34762700 | 4.39298100 | 1.12732200 |
| N | 3.31100400 | 3.10287400 | 3.19619200 |
| C | 2.64513400 | 4.30030500 | 2.64600600 |
| O | 1.63382400 | 4.74585100 | 3.06974200 |
| O | 3.39773000 | 4.73149000 | 1.65292200 |
| C | 1.17737100 | 0.48776700 | 4.28079300 |
| H | 0.96108200 | 0.78370300 | 3.24649600 |
| C | 1.89596000 | -0.84722800 | 4.23598800 |
| C | 2.15624600 | -1.40742800 | 2.98423100 |
| C | 2.34318400 | -1.50570500 | 5.38882200 |
| C | 2.81353100 | -2.63150200 | 2.88448700 |
| H | 1.82717600 | -0.88681600 | 2.08880400 |
| C | 3.01206500 | -2.72131800 | 5.28437000 |
| H | 2.16168300 | -1.07528500 | 6.36963700 |
| C | 3.24317400 | -3.28982100 | 4.03266900 |
| H | 2.98551700 | -3.06715700 | 1.90563300 |
| H | 3.34804000 | -3.22782600 | 6.18335400 |
| H | 3.75526500 | -4.24352800 | 3.95577200 |
| C | -0.12233200 | 0.57167800 | 5.07259000 |
| C | -0.84824000 | 1.76849900 | 5.00641700 |
| C | -0.60565300 | -0.47063600 | 5.86139700 |
| C | -2.02924200 | 1.91988900 | 5.72251100 |
| H | -0.48067800 | 2.58680900 | 4.39449800 |
| C | -1.78794700 | -0.31393400 | 6.58685200 |
| H | -0.08447400 | -1.42109400 | 5.89196100 |
| C | -2.49898600 | 0.87815100 | 6.52320700 |
| H | -2.58580700 | 2.84849000 | 5.65153600 |
| H | -2.15812900 | -1.13875900 | 7.18619300 |
| H | -3.42399500 | 0.99243500 | 7.07841800 |
| C | 2.59781700 | 1.58598500 | 6.27969500 |
| C | 3.94746800 | 1.34803200 | 6.56380600 |
| C | 1.69237500 | 1.82700300 | 7.31866400 |
| C | 4.39035500 | 1.34047300 | 7.88187300 |
| H | 4.63646900 | 1.13473800 | 5.75169100 |
| C | 2.14806900 | 1.81930100 | 8.63339700 |
| H | 0.64930200 | 2.02704700 | 7.10373600 |
| C | 3.49004100 | 1.57503400 | 8.91771200 |
| H | 5.43518800 | 1.14506700 | 8.09737700 |
| H | 1.44728300 | 2.00858100 | 9.43922000 |
| H | 3.83333700 | 1.56811100 | 9.94675700 |

*Z*-**TS3**

| | | | |
|---|---|---|---|
| B | -0.07313100 | 0.95202500 | -0.18807900 |
| C | -0.02996600 | -0.11944800 | 1.06297700 |
| C | -0.44327500 | 0.08563200 | 2.37730600 |
| C | 0.40807500 | -1.41423800 | 0.80200300 |
| C | -0.35882300 | -0.88754600 | 3.36626300 |

| | | | |
|---|---|---|---|
| C | 0.51985800 | -2.41443500 | 1.75587800 |
| C | 0.13397200 | -2.14547100 | 3.05825100 |
| C | -0.49133100 | 2.46451700 | 0.28115700 |
| C | -1.48203200 | 3.26254800 | -0.27965300 |
| C | 0.26754500 | 3.08808200 | 1.26926100 |
| C | -1.70727700 | 4.58349100 | 0.09617400 |
| C | 0.03663400 | 4.37849800 | 1.71752700 |
| C | -0.94275800 | 5.14573600 | 1.10334800 |
| C | -1.08532800 | 0.24572700 | -1.26367400 |
| C | -0.70948100 | -0.42043300 | -2.42113200 |
| C | -2.42737000 | 0.10632400 | -0.92179000 |
| C | -1.59807400 | -1.12732800 | -3.22383400 |
| C | -3.35178900 | -0.58783700 | -1.68624800 |
| C | -2.92973600 | -1.21339300 | -2.85207100 |
| F | 1.28203700 | 2.41633100 | 1.85125900 |
| F | 0.73906800 | 4.90540700 | 2.72953500 |
| F | -1.14220800 | 6.40512900 | 1.48946300 |
| F | -2.63848200 | 5.31308100 | -0.51928200 |
| F | -2.26241400 | 2.81290800 | -1.27121000 |
| F | 0.78634000 | -1.75534200 | -0.44613100 |
| F | 1.00292900 | -3.61485000 | 1.43004900 |
| F | 0.23003200 | -3.08249900 | 4.00015100 |
| F | -0.75515400 | -0.62103800 | 4.61273900 |
| F | -0.95858500 | 1.25648300 | 2.77749200 |
| F | -2.87291800 | 0.66762400 | 0.21264600 |
| F | -4.62933600 | -0.67415400 | -1.31222100 |
| F | -3.79709900 | -1.88898000 | -3.60455100 |
| F | -1.17667200 | -1.73116400 | -4.33811700 |
| F | 0.56839400 | -0.39025700 | -2.85235400 |
| C | 0.66680200 | 5.41137700 | -2.29011100 |
| C | 1.39984300 | 4.37802800 | -1.72616900 |
| C | 1.12728700 | 3.04846600 | -2.06228700 |
| C | 0.16460500 | 2.75764300 | -3.02689200 |
| C | -0.55667600 | 3.78790800 | -3.62348900 |
| C | -0.30912500 | 5.09198200 | -3.22636400 |
| H | 0.83787300 | 6.44729700 | -2.01887500 |
| H | 2.16880200 | 4.59104100 | -0.99349800 |
| H | -0.01147800 | 1.73461400 | -3.32931500 |
| H | -1.31260000 | 3.59204300 | -4.37468000 |
| C | 1.94145600 | 2.00585800 | -1.35611100 |
| O | 3.16636900 | 2.12573300 | -1.37411700 |
| O | 1.37428000 | 1.01401700 | -0.70958300 |
| F | -1.02140900 | 6.08398600 | -3.77922100 |
| C | 4.77322300 | 2.79666900 | 1.08761800 |
| C | 4.35043900 | 1.64552700 | 0.66856300 |
| C | 4.68668300 | -0.36213200 | -0.67935000 |
| C | 3.63373700 | -1.46804700 | -0.71264700 |
| H | 5.68879400 | -0.72513700 | -0.43765400 |
| H | 4.67926500 | 0.27334200 | -1.56246700 |
| H | 4.02503800 | -2.43610800 | -1.01472900 |
| H | 2.76087100 | -1.18321300 | -1.30129100 |
| N | 4.18922400 | 0.41014200 | 0.47059400 |

| | | | |
|---|---|---|---|
| C | 3.34276300 | -0.44125200 | 1.31130700 |
| O | 2.93811200 | -0.13164700 | 2.38366000 |
| O | 3.21136700 | -1.58556300 | 0.66733700 |
| C | 5.92504700 | 2.73110700 | 2.12391900 |
| C | 6.60825000 | 1.37070000 | 2.11950000 |
| C | 7.57757900 | 1.11622100 | 1.14405800 |
| C | 6.22270300 | 0.34066500 | 2.97921400 |
| C | 8.15400100 | -0.14535000 | 1.02729500 |
| H | 7.88007800 | 1.91432400 | 0.46993200 |
| C | 6.78847900 | -0.92723900 | 2.85281000 |
| H | 5.47293500 | 0.52149600 | 3.74275000 |
| C | 7.75329500 | -1.17397900 | 1.87897500 |
| H | 8.91402700 | -0.32443600 | 0.27366400 |
| H | 6.47642900 | -1.72085000 | 3.52359400 |
| H | 8.19764300 | -2.15974300 | 1.78961300 |
| C | 4.27457500 | 4.13418700 | 0.66230500 |
| C | 4.85081800 | 4.73840700 | -0.45779700 |
| C | 3.31459200 | 4.80585400 | 1.41601100 |
| C | 4.46659200 | 6.02630200 | -0.81589500 |
| H | 5.58296300 | 4.19547100 | -1.04699100 |
| C | 2.92586400 | 6.09212200 | 1.04254600 |
| H | 2.87069500 | 4.32609700 | 2.27891100 |
| C | 3.50741100 | 6.70540800 | -0.06408000 |
| H | 4.90921600 | 6.49564100 | -1.68779900 |
| H | 2.16114100 | 6.60162900 | 1.61933800 |
| H | 3.20784500 | 7.70889500 | -0.34845700 |
| C | 5.40344700 | 3.19322700 | 3.47387000 |
| C | 6.16840100 | 4.08648200 | 4.22481900 |
| C | 4.18638000 | 2.73315800 | 3.98663000 |
| C | 5.73165100 | 4.50838900 | 5.47729000 |
| H | 7.11089200 | 4.45217900 | 3.82655900 |
| C | 3.74934800 | 3.15933100 | 5.23828200 |
| H | 3.56775400 | 2.05124600 | 3.40726500 |
| C | 4.52058700 | 4.04592700 | 5.98583900 |
| H | 6.33487100 | 5.20365400 | 6.05165000 |
| H | 2.80083600 | 2.80056500 | 5.62362000 |
| H | 4.17574400 | 4.37989900 | 6.95875700 |
| H | 6.64337900 | 3.47705200 | 1.76225600 |

*E*-**TS3**

| | | | |
|---|---|---|---|
| B | -1.26539300 | 0.87617400 | 0.57117500 |
| C | -2.21552500 | 0.27453600 | 1.75929600 |
| C | -3.47445000 | 0.73503000 | 2.12778100 |
| C | -1.78134800 | -0.85148100 | 2.45469000 |
| C | -4.22812200 | 0.16007900 | 3.14534000 |
| C | -2.50426800 | -1.46603800 | 3.46511900 |
| C | -3.73688700 | -0.94312300 | 3.82291100 |
| C | -1.72904100 | 2.34060800 | 0.02264900 |
| C | -1.87921900 | 2.71330200 | -1.30746000 |
| C | -1.86369600 | 3.37991100 | 0.93941200 |
| C | -2.12263400 | 4.02248800 | -1.71008000 |
| C | -2.11870100 | 4.69564900 | 0.58672000 |
| C | -2.24498000 | 5.02001000 | -0.75742500 |

| | | | | | | | |
|---|---|---|---|---|---|---|---|
| C | -1.19467500 | -0.31293300 | -0.54765700 | C | 1.20327200 | 3.97651000 | -2.73787800 |
| C | -0.12583400 | -1.16329900 | -0.78584900 | H | 1.19938800 | 5.68858400 | -1.44583000 |
| C | -2.36701300 | -0.64656000 | -1.22123500 | H | 1.18157700 | 4.28908700 | 0.62200000 |
| C | -0.18608600 | -2.25031900 | -1.65196400 | H | 1.21876300 | 0.73455200 | -1.80332000 |
| C | -2.47838200 | -1.71720700 | -2.09303200 | H | 1.22262400 | 2.15436700 | -3.85797000 |
| C | -1.37286100 | -2.53140300 | -2.30758000 | C | 1.16647000 | 1.64045700 | 0.81816300 |
| F | -1.72769700 | 3.12641100 | 2.25343300 | O | 2.22246900 | 1.64210600 | 1.47830800 |
| F | -2.23403700 | 5.64756800 | 1.51409800 | O | 0.11070600 | 1.05126800 | 1.24984300 |
| F | -2.46686800 | 6.28035800 | -1.12582300 | F | 1.19612200 | 4.73283400 | -3.84269100 |
| F | -2.20135400 | 4.32790200 | -3.00650100 | C | 2.42438200 | 1.05348000 | 4.28326900 |
| F | -1.74124100 | 1.82234000 | -2.30002500 | C | 2.85672100 | 1.95925300 | 3.45430200 |
| F | -0.58509500 | -1.39925000 | 2.16673900 | C | 4.80454400 | 2.91384300 | 2.32114800 |
| F | -2.02300400 | -2.53396900 | 4.10386700 | C | 4.53814700 | 3.96078900 | 1.23669600 |
| F | -4.42925400 | -1.48435800 | 4.82395200 | H | 4.94464000 | 1.91241500 | 1.91838100 |
| F | -5.41686900 | 0.66802300 | 3.48019000 | H | 5.62690200 | 3.18732400 | 2.98453300 |
| F | -4.03650500 | 1.78800800 | 1.51803000 | H | 4.09242600 | 3.51253600 | 0.34596900 |
| F | -3.46085200 | 0.10500300 | -1.02969100 | H | 5.41019800 | 4.55843600 | 0.98264600 |
| F | -3.62624800 | -1.98082300 | -2.71761000 | N | 3.54073100 | 2.98386100 | 3.07586200 |
| F | -1.45327500 | -3.56727700 | -3.14049300 | C | 2.84219500 | 4.21084700 | 2.74652800 |
| F | 0.88623100 | -3.01988300 | -1.85210400 | O | 1.80550400 | 4.54984400 | 3.22034800 |
| F | 1.07704000 | -0.95118700 | -0.20526900 | O | 3.55534000 | 4.84128900 | 1.82066900 |
| C | 1.21020100 | 4.60596600 | -1.49907600 | C | 1.46026500 | -0.08331100 | 3.96504800 |
| C | 1.21074500 | 3.81713800 | -0.35653400 | H | 1.18473800 | 0.03441500 | 2.91697900 |
| C | 1.18322100 | 2.42123300 | -0.46515300 | C | 2.16604500 | -1.41954400 | 4.10513100 |
| C | 1.20617800 | 1.81374300 | -1.71860000 | C | 2.32828400 | -2.19784800 | 2.95590000 |
| C | 1.21703800 | 2.59755300 | -2.86924900 | C | 2.67912000 | -1.89007900 | 5.32011300 |

| | | | |
|---|---|---|---|
| C | 2.98494900 | -3.42398000 | 3.01685600 |
| H | 1.92928500 | -1.83893000 | 2.01117600 |
| C | 3.33619400 | -3.11595800 | 5.37930100 |
| H | 2.56022400 | -1.30322600 | 6.22613100 |
| C | 3.49174000 | -3.88533400 | 4.22858800 |
| H | 3.09751200 | -4.01873700 | 2.11623000 |
| H | 3.72498700 | -3.47052000 | 6.32844700 |
| H | 4.00241200 | -4.84160400 | 4.27796400 |
| C | 0.18801800 | 0.13152700 | 4.78036400 |
| C | -0.54982400 | 1.29268900 | 4.51068200 |
| C | -0.27976500 | -0.76099000 | 5.74141800 |
| C | -1.72685800 | 1.56046600 | 5.19716800 |
| H | -0.20630000 | 1.98159800 | 3.74276600 |
| C | -1.46073200 | -0.48908100 | 6.43550000 |
| H | 0.25212100 | -1.68542400 | 5.93444600 |
| C | -2.18155500 | 0.67010900 | 6.17240100 |
| H | -2.29416000 | 2.45442200 | 4.95788200 |
| H | -1.82163900 | -1.19850900 | 7.17318000 |
| H | -3.10552300 | 0.87070500 | 6.70565000 |
| C | 3.00302000 | 1.23618100 | 5.65866900 |
| C | 4.35545700 | 0.94962600 | 5.86091600 |
| C | 2.19815200 | 1.64273000 | 6.72681200 |
| C | 4.90164100 | 1.04978900 | 7.13635500 |
| H | 4.96285800 | 0.61747700 | 5.02381300 |
| C | 2.75497400 | 1.74026600 | 7.99874900 |
| H | 1.15307700 | 1.88141800 | 6.56228700 |
| C | 4.10044200 | 1.44227600 | 8.20580400 |
| H | 5.94915200 | 0.81659300 | 7.29411600 |
| H | 2.13333500 | 2.05545100 | 8.82982100 |
| H | 4.52488400 | 1.51946400 | 9.20124200 |

(*Z*)-**3aa**

| | | | |
|---|---|---|---|
| C | 10.89620300 | 7.73127700 | -9.58866000 |
| C | 11.60754100 | 6.85149000 | -8.78340100 |
| C | 10.93191400 | 6.05249500 | -7.85662600 |
| C | 9.54237800 | 6.13668900 | -7.73165100 |
| C | 8.82102200 | 7.00981400 | -8.53262500 |
| C | 9.51640100 | 7.79052400 | -9.44698300 |
| H | 11.38612800 | 8.36858400 | -10.31511900 |
| H | 12.68613700 | 6.78641900 | -8.86426100 |
| H | 9.04392300 | 5.50954300 | -7.00058400 |
| H | 7.74342400 | 7.09852500 | -8.46323300 |
| C | 11.64569500 | 5.10412700 | -6.96684300 |
| O | 12.97853100 | 5.06178200 | -7.23426500 |
| O | 11.13121300 | 4.44509000 | -6.09809800 |
| F | 8.82790900 | 8.63632000 | -10.22262900 |
| C | 14.70596600 | 4.61711600 | -5.68948700 |
| C | 13.74543800 | 4.15339600 | -6.49786500 |
| C | 13.31241500 | 2.35652500 | -8.17612600 |
| C | 13.10396900 | 0.86137100 | -7.91662200 |
| H | 14.21656800 | 2.55291000 | -8.75714200 |
| H | 12.45123100 | 2.82446100 | -8.66757800 |
| H | 14.05117800 | 0.31714600 | -7.96561200 |

| | | | |
|---|---|---|---|
| H | 12.36765100 | 0.40453800 | -8.57656300 |
| N | 13.46175300 | 2.82601000 | -6.80799200 |
| C | 12.92789200 | 1.92408200 | -5.90416600 |
| O | 12.76625200 | 2.06750800 | -4.72311600 |
| O | 12.61231300 | 0.79089600 | -6.57330100 |
| C | 15.81595900 | 3.78630700 | -5.06060500 |
| H | 16.72578000 | 4.36610800 | -5.27314200 |
| C | 15.72084400 | 3.73879800 | -3.54074900 |
| C | 16.89992400 | 3.59797000 | -2.80358500 |
| C | 14.50324200 | 3.81026400 | -2.86490100 |
| C | 16.86709700 | 3.53013200 | -1.41524300 |
| H | 17.85100000 | 3.53773500 | -3.32793900 |
| C | 14.46944500 | 3.74394000 | -1.47280200 |
| H | 13.58177500 | 3.88963700 | -3.43215000 |
| C | 15.64730700 | 3.60565500 | -0.74485000 |
| H | 17.79169100 | 3.42334700 | -0.85673000 |
| H | 13.51539800 | 3.79860800 | -0.95823400 |
| H | 15.61708400 | 3.55651300 | 0.33897000 |
| C | 16.05964400 | 2.42560900 | -5.70286500 |
| C | 16.61643500 | 2.38186200 | -6.98551300 |
| C | 15.76043000 | 1.22668500 | -5.05664300 |
| C | 16.85496100 | 1.16708700 | -7.61910900 |
| H | 16.85824700 | 3.31515000 | -7.49069800 |
| C | 15.99109200 | 0.00705600 | -5.69249100 |
| H | 15.32280800 | 1.24949700 | -4.06393700 |
| C | 16.54102600 | -0.02871700 | -6.97115500 |
| H | 17.29353300 | 1.15100300 | -8.61225800 |
| H | 15.74204100 | -0.91758100 | -5.18181100 |
| H | 16.72960600 | -0.97996700 | -7.45882200 |
| C | 14.72725000 | 6.07138500 | -5.36066600 |
| C | 15.86942600 | 6.84396300 | -5.58845700 |
| C | 13.60045400 | 6.68111300 | -4.79677700 |
| C | 15.88252900 | 8.19887600 | -5.26874600 |
| H | 16.74979600 | 6.39124200 | -6.03623100 |
| C | 13.61404700 | 8.03387200 | -4.47617900 |
| H | 12.71622000 | 6.07903700 | -4.60272800 |
| C | 14.75601000 | 8.79709400 | -4.71103500 |
| H | 16.77438300 | 8.78751900 | -5.45798100 |
| H | 12.73401400 | 8.49095000 | -4.03544100 |
| H | 14.76861500 | 9.85205200 | -4.45729900 |

(*E*)-**3aa**

| | | | |
|---|---|---|---|
| C | 7.07447600 | -0.03659000 | 0.07583400 |
| C | 5.97229400 | 0.31445700 | 0.84431600 |
| C | 5.91236800 | 1.57531900 | 1.44461700 |
| C | 6.95862900 | 2.48673600 | 1.27653900 |
| C | 8.06462100 | 2.14764500 | 0.51113400 |
| C | 8.09839700 | 0.88886900 | -0.07476400 |
| H | 7.15547600 | -1.00480400 | -0.40371600 |
| H | 5.15774200 | -0.38724600 | 0.98132500 |
| H | 6.88813300 | 3.45721800 | 1.75539800 |
| H | 8.89273000 | 2.83014600 | 0.36160000 |
| C | 4.76066800 | 2.00137500 | 2.27750400 |

| | | | |
|---|---|---|---|
| O | 3.81901800 | 1.01882600 | 2.38759400 |
| O | 4.64903500 | 3.07500700 | 2.81031600 |
| F | 9.16144100 | 0.55358200 | -0.81504400 |
| C | 2.37267800 | 0.80544000 | 4.26740000 |
| C | 2.65766300 | 1.35322300 | 3.08505700 |
| C | 1.14186000 | 2.00936600 | 1.17637400 |
| C | 0.65340500 | 3.42473700 | 0.84210400 |
| H | 0.31679000 | 1.30820200 | 1.34793600 |
| H | 1.80833300 | 1.60606500 | 0.41111600 |
| H | -0.36126400 | 3.44878400 | 0.44723000 |
| H | 1.33230300 | 3.93180600 | 0.15117200 |
| N | 1.85016400 | 2.27587600 | 2.41724600 |
| C | 1.48559200 | 3.48879600 | 2.97023400 |
| O | 1.80233000 | 3.94342900 | 4.03536700 |
| O | 0.67023900 | 4.12389800 | 2.09101100 |
| C | 3.39349000 | -0.04553700 | 5.01044400 |
| H | 4.22534500 | -0.21371300 | 4.31777300 |
| C | 3.98019200 | 0.69929500 | 6.21408400 |
| C | 4.15656200 | 2.08711100 | 6.16157200 |
| C | 4.44476300 | 0.00593400 | 7.33464300 |
| C | 4.76341100 | 2.76278100 | 7.21724300 |
| H | 3.80639100 | 2.64833600 | 5.29776100 |
| C | 5.05516800 | 0.68345000 | 8.38767400 |
| H | 4.32973900 | -1.07203400 | 7.38658900 |
| C | 5.21301000 | 2.06520300 | 8.33553200 |
| H | 4.88299900 | 3.84009400 | 7.16122900 |

| | | | |
|---|---|---|---|
| H | 5.40636300 | 0.12653600 | 9.25078400 |
| H | 5.68360300 | 2.59380800 | 9.15833200 |
| C | 2.79557100 | -1.41044400 | 5.31560500 |
| C | 1.89960300 | -1.60862600 | 6.37202300 |
| C | 3.09289400 | -2.48653800 | 4.47861100 |
| C | 1.32316200 | -2.85636100 | 6.58493100 |
| H | 1.65446300 | -0.77774500 | 7.02788900 |
| C | 2.51684000 | -3.73783200 | 4.68882700 |
| H | 3.78297700 | -2.33971700 | 3.65106200 |
| C | 1.62988200 | -3.92537600 | 5.74427700 |
| H | 0.63025000 | -2.99392100 | 7.40886600 |
| H | 2.76393600 | -4.56406300 | 4.02971700 |
| H | 1.18022900 | -4.89854300 | 5.91336600 |
| C | 1.00878700 | 0.98185900 | 4.84274100 |
| C | -0.04909200 | 0.26373500 | 4.27457200 |
| C | 0.76264000 | 1.79182300 | 5.95556200 |
| C | -1.33200100 | 0.34993300 | 4.80702400 |
| H | 0.15087300 | -0.38698400 | 3.42715700 |
| C | -0.52113900 | 1.87337000 | 6.48827600 |
| H | 1.57116400 | 2.36983600 | 6.38774000 |
| C | -1.56920500 | 1.15394600 | 5.91881200 |
| H | -2.14187300 | -0.21736000 | 4.35964300 |
| H | -0.70341900 | 2.50955900 | 7.34831800 |
| H | -2.56755800 | 1.22150400 | 6.33903600 |

**TS1’**

| | | | |
|---|---|---|---|
| B | -2.08648700 | -0.16200000 | 0.64384600 |
| C | -3.07341000 | -1.22284700 | 1.43668400 |
| C | -4.45776700 | -1.12610500 | 1.52128200 |
| C | -2.53725800 | -2.36736700 | 2.02639000 |
| C | -5.25608200 | -2.04728000 | 2.19320400 |
| C | -3.29444900 | -3.29953600 | 2.72693000 |
| C | -4.67030200 | -3.13854100 | 2.81536700 |
| C | -2.76597000 | 1.33011900 | 0.55608300 |
| C | -2.86629800 | 2.13588100 | -0.57114100 |
| C | -3.07798500 | 1.97382700 | 1.74720400 |
| C | -3.20258900 | 3.48527000 | -0.51663900 |
| C | -3.40934500 | 3.31315200 | 1.85938500 |
| C | -3.47303200 | 4.08153000 | 0.70540600 |
| C | -1.78926600 | -0.91714700 | -0.77832200 |
| C | -0.60694200 | -1.56587200 | -1.11266100 |
| C | -2.83686400 | -1.11575900 | -1.67357700 |
| C | -0.45209400 | -2.32200700 | -2.27136300 |
| C | -2.73125500 | -1.85963800 | -2.83758200 |
| C | -1.52226100 | -2.47410300 | -3.13694800 |
| F | -2.99387200 | 1.29224300 | 2.92478700 |
| F | -3.62079300 | 3.86554500 | 3.05711900 |
| F | -3.76888300 | 5.37702500 | 0.77414500 |
| F | -3.22822500 | 4.21986200 | -1.62778200 |
| F | -2.56160300 | 1.66293700 | -1.78542100 |
| F | -1.22888500 | -2.62793800 | 1.93408400 |
| F | -2.70796500 | -4.34694900 | 3.31527400 |
| F | -5.41231100 | -4.01131700 | 3.49324900 |
| F | -6.57248300 | -1.84297500 | 2.30472000 |
| F | -5.11683200 | -0.07653800 | 1.00169600 |
| F | -4.03366600 | -0.56680900 | -1.41010200 |
| F | -3.77175400 | -2.00432400 | -3.66002600 |
| F | -1.39354700 | -3.19794600 | -4.24777800 |
| F | 0.71558900 | -2.90526600 | -2.55050500 |
| F | 0.48669900 | -1.45347800 | -0.34129100 |
| C | 0.09367600 | 4.40895100 | 0.06401700 |
| C | 0.01943800 | 3.39378000 | 1.00901200 |
| C | 0.07083000 | 2.05285200 | 0.61547500 |
| C | 0.26591400 | 1.73207500 | -0.72750400 |
| C | 0.36560600 | 2.73873500 | -1.68225700 |
| C | 0.25528400 | 4.05758800 | -1.26989100 |
| H | 0.02858300 | 5.45604400 | 0.33703700 |
| H | -0.09023400 | 3.62937000 | 2.06374000 |
| H | 0.35472300 | 0.69615700 | -1.02555900 |
| H | 0.51428100 | 2.51667600 | -2.73236200 |
| C | -0.08204300 | 1.01745800 | 1.69395300 |
| O | 0.50803000 | 1.17753000 | 2.74978600 |
| O | -0.86105900 | -0.02941500 | 1.50855600 |
| C | -0.53576200 | 0.63057300 | 5.10524900 |
| H | -1.37746500 | 0.62829200 | 4.41613300 |
| C | -0.09887000 | 1.95968500 | 5.49263400 |
| C | -0.76329900 | 3.03771400 | 4.87927200 |
| C | 0.88092000 | 2.22215400 | 6.46546700 |

| | | | |
|---|---|---|---|
| C | -0.42611700 | 4.34391200 | 5.19048400 |
| H | -1.55265200 | 2.83385400 | 4.16107400 |
| C | 1.21238200 | 3.53526500 | 6.77748900 |
| H | 1.35191200 | 1.40944100 | 7.00614600 |
| C | 0.57004800 | 4.59312100 | 6.13659900 |
| H | -0.94722900 | 5.16434200 | 4.70911700 |
| H | 1.96679300 | 3.73327200 | 7.53103600 |
| H | 0.83402000 | 5.61550100 | 6.38671700 |
| C | 0.21340900 | -0.61582000 | 5.09488300 |
| C | -0.48140700 | -1.77436700 | 4.70606300 |
| C | 1.58004200 | -0.71185900 | 5.39671200 |
| C | 0.14993600 | -3.00603900 | 4.67001300 |
| H | -1.53420800 | -1.69492500 | 4.44489300 |
| C | 2.21378600 | -1.94818100 | 5.35900900 |
| H | 2.15823400 | 0.17992800 | 5.60074600 |
| C | 1.50104600 | -3.09432500 | 5.01251600 |
| H | -0.40254300 | -3.88405100 | 4.35552300 |
| H | 3.27253000 | -2.01433200 | 5.58384200 |
| H | 2.00387200 | -4.05534800 | 4.98255300 |
| F | 0.32568800 | 5.03186500 | -2.18849600 |
| C | -0.08752200 | -1.18845900 | 8.19637000 |
| C | 0.81209700 | -1.33114400 | 9.24724900 |
| C | 0.97745900 | -0.30582100 | 10.17599900 |
| C | 0.23549700 | 0.86944300 | 10.06147900 |
| C | -0.66855200 | 1.02507200 | 9.01863600 |
| C | -0.83231900 | -0.00701100 | 8.08159400 |
| H | -0.21541800 | -1.98263800 | 7.46753600 |
| H | 1.38716900 | -2.24644900 | 9.33717700 |
| H | 1.68322500 | -0.42301400 | 10.99157900 |
| H | 0.36220100 | 1.66756200 | 10.78498300 |
| H | -1.24358700 | 1.93845500 | 8.90785200 |
| C | -1.72779900 | 0.20512300 | 6.97694500 |
| C | -2.77794300 | 0.43242100 | 6.37615200 |
| C | -4.50146400 | -0.28838400 | 4.76791400 |
| C | -5.60928400 | 0.59298600 | 4.15530500 |
| H | -4.89993000 | -1.10270300 | 5.37444200 |
| H | -3.81155900 | -0.67973500 | 4.01703200 |
| H | -6.59007100 | 0.36707400 | 4.57515400 |
| H | -5.64812200 | 0.53657900 | 3.06942900 |
| N | -3.80723100 | 0.69115900 | 5.60425400 |
| C | -4.26746800 | 2.01887200 | 5.36733400 |
| O | -3.81895100 | 3.00549400 | 5.86561100 |
| O | -5.27947100 | 1.94710500 | 4.50701700 |

**TS4**

| | | | |
|---|---|---|---|
| C | 11.95789300 | 4.15781600 | -4.29907000 |
| C | 13.03296500 | 4.74315400 | -4.40231800 |
| C | 14.89066600 | 6.20639200 | -3.59609000 |
| C | 16.35727100 | 6.04879100 | -4.02844200 |
| H | 14.74784100 | 5.92674200 | -2.55285900 |
| H | 14.49116000 | 7.20550100 | -3.78069800 |
| H | 16.90314800 | 5.37599900 | -3.36384500 |
| H | 16.88095500 | 6.99801500 | -4.12122600 |

N 14.25670000 5.23074700 -4.48876400
C 15.12769400 4.84545700 -5.53716600
O 14.85180800 4.12852200 -6.45199500
O 16.30826100 5.43865300 -5.32934000
C 10.54923200 3.91726800 -4.52602400
C 9.61203600 4.86281600 -4.09173300
C 10.12075300 2.73372000 -5.13863900
C 8.25553500 4.61488200 -4.25904500
H 9.95742300 5.76407600 -3.59748200
C 8.76086100 2.50241500 -5.31312300
H 10.85306500 2.00655100 -5.46679200
C 7.82741600 3.43535000 -4.86659200
H 7.53052800 5.34255900 -3.91060900
H 8.43083900 1.58610400 -5.79090900
H 6.76675600 3.24469400 -4.99186500
B 12.99328000 2.92308900 -2.63601700
C 11.74612100 1.95734700 -2.40256900
C 11.76186400 0.57288400 -2.57760400
C 10.54197500 2.47491600 -1.91517300
C 10.64286900 -0.22633100 -2.37838700
C 9.40061100 1.71490100 -1.71902700
C 9.45327400 0.34881300 -1.95727200
C 14.26732600 2.41906200 -3.45464300
C 15.57897700 2.80199200 -3.17109400
C 14.13241100 1.65473500 -4.61612200
C 16.67277900 2.43879800 -3.94697700
C 15.19617900 1.24829500 -5.40476800
C 16.48245600 1.64757100 -5.06895700
C 13.23308400 3.87400200 -1.35459300
C 12.78827000 5.16246700 -1.08498700
C 13.88252400 3.24240400 -0.29438700
C 13.00080100 5.79615100 0.13571200
C 14.11047200 3.83235100 0.93753700
C 13.66154300 5.12840700 1.15390800
F 12.91383000 1.31005300 -5.04596200
F 14.99230400 0.51953100 -6.49667100
F 17.51349700 1.29020400 -5.82074900
F 17.89136900 2.87420800 -3.62712400
F 15.86238500 3.60725400 -2.13171400
F 10.43717800 3.78204800 -1.64603600
F 8.27094900 2.27591900 -1.29478300
F 8.37772900 -0.40519600 -1.76637800
F 10.71056000 -1.54244200 -2.56803900
F 12.88125300 -0.06810100 -2.92328000
F 14.33475300 1.99232700 -0.47120000
F 14.74771100 3.17646600 1.90430300
F 13.86699300 5.72317900 2.32394600
F 12.56314200 7.03934100 0.33115600
F 12.13453400 5.88715300 -2.00571100

**IM4**

C 12.06217900 4.00402100 -3.99938500
C 13.02296500 4.76442200 -4.33058400

| | | | |
|---|---|---|---|
| C | 14.79641400 | 6.37264600 | -3.70060900 |
| C | 16.26523500 | 6.16558100 | -4.09854900 |
| H | 14.61374100 | 6.17745900 | -2.64435500 |
| H | 14.41159400 | 7.35154800 | -3.98847900 |
| H | 16.78853400 | 5.50873800 | -3.40324900 |
| H | 16.80693600 | 7.09894900 | -4.23249500 |
| N | 14.15507100 | 5.33001200 | -4.52246900 |
| C | 15.06032300 | 4.88921800 | -5.57769500 |
| O | 14.76605400 | 4.13997000 | -6.45040300 |
| O | 16.21948900 | 5.49969900 | -5.37777000 |
| C | 10.64900200 | 4.01169600 | -4.42600200 |
| C | 9.90387700 | 5.19255000 | -4.35850500 |
| C | 10.06446600 | 2.83615000 | -4.90249700 |
| C | 8.57500900 | 5.19242900 | -4.76435400 |
| H | 10.36343800 | 6.09315800 | -3.96594400 |
| C | 8.73650000 | 2.85018300 | -5.31922700 |
| H | 10.65319400 | 1.92720900 | -4.95602400 |
| C | 7.98970700 | 4.02279200 | -5.24600500 |
| H | 7.99480600 | 6.10695600 | -4.70208600 |
| H | 8.28577400 | 1.93938600 | -5.69871400 |
| H | 6.95204300 | 4.02611600 | -5.56255800 |
| B | 12.82370500 | 2.93121900 | -2.77381700 |
| C | 11.72432500 | 1.80083400 | -2.37722200 |
| C | 11.92751100 | 0.42282400 | -2.37451600 |
| C | 10.49487400 | 2.21096700 | -1.85823200 |
| C | 10.95840700 | -0.48630900 | -1.96284000 |
| C | 9.50020300 | 1.33902400 | -1.44398200 |
| C | 9.73524000 | -0.02723000 | -1.50141100 |
| C | 14.17195200 | 2.39747300 | -3.52061300 |
| C | 15.46137300 | 2.85569700 | -3.27293300 |
| C | 14.06967900 | 1.55898900 | -4.62941300 |
| C | 16.57192700 | 2.49756600 | -4.03079700 |
| C | 15.14730200 | 1.15418100 | -5.40112500 |
| C | 16.41664800 | 1.63113100 | -5.09970700 |
| C | 13.09742000 | 3.77358800 | -1.40040900 |
| C | 12.59263700 | 5.01614600 | -1.03720700 |
| C | 13.81434400 | 3.12092000 | -0.39760100 |
| C | 12.81174800 | 5.59492800 | 0.21066500 |
| C | 14.05561200 | 3.65574200 | 0.85679700 |
| C | 13.54601900 | 4.91115500 | 1.16429900 |
| F | 12.86079700 | 1.12505700 | -5.01512100 |
| F | 14.97535700 | 0.34284100 | -6.44095600 |
| F | 17.46181300 | 1.27510100 | -5.83742000 |
| F | 17.76868800 | 3.01731600 | -3.74788200 |
| F | 15.70657500 | 3.75552300 | -2.29576300 |
| F | 10.23017200 | 3.52050700 | -1.73769600 |
| F | 8.33907000 | 1.79681700 | -0.97726300 |
| F | 8.80143900 | -0.88628900 | -1.10348000 |
| F | 11.20567300 | -1.79569100 | -1.99208800 |
| F | 13.09420700 | -0.11134100 | -2.75245400 |
| F | 14.32485500 | 1.90693800 | -0.65026700 |
| F | 14.76378700 | 2.98571100 | 1.76465000 |

F 13.76351100 5.44975200 2.36097900

F 12.30946600 6.79785900 0.49308400

F 11.85312100 5.75018600 -1.88859000

**TS5**

C 9.84539700 7.81212000 -8.46854900

C 10.29391700 6.78630200 -7.64471700

C 11.65393400 6.68228100 -7.34703300

C 12.56719600 7.61064200 -7.84823800

C 12.13126700 8.62383500 -8.69087600

C 10.77706500 8.70088000 -8.98644800

H 8.79674900 7.93296800 -8.71302600

H 9.58664800 6.08049300 -7.21766200

H 13.62021200 7.51569700 -7.60506500

H 12.81495500 9.34735200 -9.11846800

C 12.16103100 5.58084800 -6.48168700

O 12.73834400 5.83651900 -5.42850900

O 11.99236800 4.32089800 -6.82632000

F 10.35124500 9.67651800 -9.79698000

C 12.34697500 3.66460500 -3.67962300

C 13.33728800 4.38096200 -4.10046700

C 15.25246000 5.70723200 -3.23437900

C 16.72323900 5.40056700 -3.54802800

H 15.00582600 5.55006700 -2.18443700

H 14.94246300 6.70233300 -3.56136900

H 17.16020100 4.71278500 -2.82285600

H 17.33743000 6.29227500 -3.65194300

N 14.60347900 4.68930700 -4.06824900

C 15.49371100 4.22635100 -5.07902700

O 15.19427000 3.52491300 -5.99994600

O 16.69894100 4.73202100 -4.82725100

C 10.95775100 3.75605500 -4.18224400

C 10.24171300 4.95434500 -4.11094300

C 10.39021000 2.64051300 -4.80176800

C 8.97899200 5.04055400 -4.68779900

H 10.68123900 5.80814300 -3.60756500

C 9.13612200 2.74055700 -5.39880000

H 10.95040200 1.71339100 -4.85409000

C 8.42925800 3.93924800 -5.34622800

H 8.42267200 5.97068500 -4.62348900

H 8.71791800 1.87825100 -5.90809300

H 7.44672400 4.01310700 -5.80164600

B 12.90051000 2.59169800 -2.43301900

C 11.70333400 1.54002700 -2.06304400

C 11.80057300 0.15176000 -2.01169400

C 10.47974400 2.04869200 -1.62333400

C 10.74599400 -0.67387800 -1.63473500

C 9.40147800 1.26367300 -1.24494000

C 9.53611600 -0.11683300 -1.25548300

C 14.26220500 1.90778400 -3.03808100

C 15.56465300 2.24207600 -2.68401800

C 14.18264800 1.04918000 -4.13076300

C 16.69675200 1.77312200 -3.34294000

C 15.27765600 0.53911700 -4.80968500
C 16.55576400 0.90697500 -4.41421200
C 13.15362600 3.42475700 -1.04282500
C 12.74991600 4.71846500 -0.73872100
C 13.72193500 2.72925600 0.02412000
C 12.92706000 5.30213200 0.51353600
C 13.91724100 3.26644300 1.28556200
C 13.51265100 4.57212400 1.53310200
F 12.97558000 0.69668300 -4.60429500
F 15.11554200 -0.27635900 -5.85324600
F 17.62243800 0.44651800 -5.05926800
F 17.90943500 2.18668500 -2.96444800
F 15.81526500 3.13146000 -1.70051200
F 10.30526000 3.37698100 -1.53570400
F 8.25402500 1.81867200 -0.85255500
F 8.52001100 -0.89547600 -0.89049100
F 10.89845600 -1.99913200 -1.61934300
F 12.94260000 -0.48207000 -2.30753600
F 14.13023800 1.46559000 -0.16618600
F 14.48274000 2.55095700 2.25808100
F 13.68828000 5.11405500 2.73638800
F 12.52634500 6.55547300 0.73721200
F 12.15656900 5.50253900 -1.65722600
C 11.54411000 3.93941000 -8.15385300
H 10.53126600 4.32357500 -8.30431600
C 11.49880900 2.42891400 -8.17496800
C 12.36810800 1.65541300 -7.40637400
C 10.58373500 1.81010900 -9.02700200
C 12.31249500 0.26543600 -7.49274700
H 13.06824800 2.13737300 -6.72794700
C 10.53510500 0.42293500 -9.11519300
H 9.90613000 2.41747000 -9.62237900
C 11.40099000 -0.35108500 -8.34597300
H 12.98144800 -0.33070800 -6.88364700
H 9.81850700 -0.05188800 -9.77741500
H 11.36206100 -1.43376300 -8.40729500
C 12.46978100 4.51785000 -9.20453200
C 11.94176900 5.18188600 -10.30946300
C 13.85348400 4.40583100 -9.05201700
C 12.79146400 5.74019800 -11.26105900
H 10.86416800 5.28566200 -10.41180000
C 14.69981900 4.97220200 -10.00013300
H 14.26430900 3.88855800 -8.18799900
C 14.17135200 5.64028900 -11.10424900
H 12.37550500 6.26339300 -12.11587900
H 15.77488200 4.88870500 -9.87820000
H 14.83500800 6.08186300 -11.84049400

**IM5**

C 10.11821200 7.78985100 -8.40456100
C 10.49351400 6.74913100 -7.56508200
C 11.85032600 6.52668100 -7.32239200
C 12.83786500 7.33570400 -7.88748100

| | | | | | | | |
|---|---|---|---|---|---|---|---|
| C | 12.46587400 | 8.35907000 | -8.74672900 | H | 10.67818800 | 5.99952900 | -3.51775800 |
| C | 11.11318900 | 8.56167400 | -8.98969900 | C | 9.05186200 | 3.01980800 | -5.37345000 |
| H | 9.07785400 | 8.00941000 | -8.61212100 | H | 10.89944000 | 1.98111800 | -4.99806400 |
| H | 9.74069700 | 6.12912100 | -7.08504700 | C | 8.33998100 | 4.20890300 | -5.22094100 |
| H | 13.88532900 | 7.13363700 | -7.68844100 | H | 8.37271600 | 6.20297000 | -4.40411900 |
| H | 13.19877600 | 8.99501000 | -9.22819500 | H | 8.60707000 | 2.17754500 | -5.89442000 |
| C | 12.26697200 | 5.38371900 | -6.48682400 | H | 7.33087300 | 4.29600800 | -5.61109500 |
| O | 12.80254000 | 5.67610000 | -5.36658600 | B | 12.89035600 | 2.83063000 | -2.57063500 |
| O | 12.11433200 | 4.16190700 | -6.81350700 | C | 11.72316900 | 1.70558100 | -2.27438800 |
| F | 10.75342000 | 9.54627200 | -9.81612300 | C | 11.83433200 | 0.32361700 | -2.39976400 |
| C | 12.36807100 | 3.88195600 | -3.76714900 | C | 10.49003600 | 2.13647800 | -1.78199800 |
| C | 13.23568700 | 4.68046700 | -4.38648100 | C | 10.79473700 | -0.55859600 | -2.12074300 |
| C | 15.23378400 | 5.57326700 | -3.07903100 | C | 9.42581900 | 1.29498700 | -1.49417600 |
| C | 16.71177100 | 5.38523600 | -3.46284100 | C | 9.58002500 | -0.07187800 | -1.66855000 |
| H | 15.00627700 | 5.10601500 | -2.12109300 | C | 14.29610400 | 2.18357800 | -3.13549900 |
| H | 14.92459900 | 6.62277800 | -3.06385900 | C | 15.57983700 | 2.35513400 | -2.63521700 |
| H | 17.17611400 | 4.56890300 | -2.90572200 | C | 14.25833500 | 1.53716700 | -4.36830500 |
| H | 17.30315500 | 6.29272800 | -3.35125300 | C | 16.73269300 | 1.95942200 | -3.30928900 |
| N | 14.59657300 | 4.86025100 | -4.18246600 | C | 15.37301800 | 1.12429600 | -5.07758100 |
| C | 15.46427500 | 4.66696700 | -5.23126500 | C | 16.63579800 | 1.34535300 | -4.54557200 |
| O | 15.16939100 | 4.25914600 | -6.33224000 | C | 13.08342200 | 3.59123800 | -1.12212700 |
| O | 16.70235200 | 5.02938500 | -4.85883500 | C | 12.76848100 | 4.90619400 | -0.80725100 |
| C | 10.94452700 | 3.98119200 | -4.20356800 | C | 13.50159300 | 2.82343700 | -0.03606900 |
| C | 10.22111300 | 5.16891500 | -4.04620100 | C | 12.87100300 | 5.43814600 | 0.47466700 |
| C | 10.34050100 | 2.90085400 | -4.85962600 | C | 13.61774300 | 3.30566300 | 1.25776800 |
| C | 8.92544200 | 5.27930600 | -4.54564300 | C | 13.29341000 | 4.63086500 | 1.51684200 |

| | | | |
|---|---|---|---|
| F | 13.06692200 | 1.30333100 | -4.94405500 |
| F | 15.25013200 | 0.54100700 | -6.27499800 |
| F | 17.72663300 | 0.97004100 | -5.20795200 |
| F | 17.93431900 | 2.22176200 | -2.78456200 |
| F | 15.81162200 | 3.00780200 | -1.47834600 |
| F | 10.27790500 | 3.44524600 | -1.56281300 |
| F | 8.26845000 | 1.78301400 | -1.04237600 |
| F | 8.57673500 | -0.90533100 | -1.39601500 |
| F | 10.96504500 | -1.87406400 | -2.27320500 |
| F | 12.97661100 | -0.25667100 | -2.79434600 |
| F | 13.84192700 | 1.54141700 | -0.23416600 |
| F | 14.03474900 | 2.51859400 | 2.25086200 |
| F | 13.39725500 | 5.12088000 | 2.75115100 |
| F | 12.55999000 | 6.71646100 | 0.70522700 |
| F | 12.35797400 | 5.77148200 | -1.75594900 |
| C | 11.53035700 | 3.77069500 | -8.12481200 |
| H | 10.51099400 | 4.16440400 | -8.13315600 |
| C | 11.48206000 | 2.26658000 | -8.11456800 |
| C | 12.57958300 | 1.51248000 | -7.70288000 |
| C | 10.32182000 | 1.63148100 | -8.55670600 |
| C | 12.50658600 | 0.12335000 | -7.72153400 |
| H | 13.47231400 | 2.00315900 | -7.32673300 |
| C | 10.25954700 | 0.24182700 | -8.59455300 |
| H | 9.46507500 | 2.22466700 | -8.86644900 |
| C | 11.35225300 | -0.51217600 | -8.17355200 |
| H | 13.35173000 | -0.45621400 | -7.36859700 |
| H | 9.35577700 | -0.24959300 | -8.93896100 |
| H | 11.30072800 | -1.59584900 | -8.18749600 |
| C | 12.33565600 | 4.37416900 | -9.25207000 |
| C | 11.66198100 | 5.00169700 | -10.29878000 |
| C | 13.73144500 | 4.32905400 | -9.24437300 |
| C | 12.37847500 | 5.58848000 | -11.33799500 |
| H | 10.57587400 | 5.04927100 | -10.29064900 |
| C | 14.44304200 | 4.92516600 | -10.28109500 |
| H | 14.26440600 | 3.87192600 | -8.41606900 |
| C | 13.77053000 | 5.55439400 | -11.32738100 |
| H | 11.84951000 | 6.08148600 | -12.14687900 |
| H | 15.52757900 | 4.89947100 | -10.26761400 |
| H | 14.33104500 | 6.01982400 | -12.13139300 |

## 17 References


1 (a) P. Hansjacob, F. R. Leroux, V. Gandon and M. Donnard, *Angew. Chem. Int. Ed.*, 2022, **61**, e202200204; (b) C. Theunissen, P. Thilmany, M. Lahboubi, N. Blanchard and G. Evano, *Org. Synth.*, 2019, **96**, 195-213; (c) T. Hamada, X. Ye and S. S. Stahl, *J. Am. Chem. Soc.*, 2008, **130**, 833-835.

2 (a) A. Dasgupta, R. Babaahmadi, S. Pahar, K. Stefkova, L. Gierlichs, B. F. Yates, A. Ariafard and R. L. Melen, *Angew. Chem. Int. Ed.*, 2021, **60**, 24395-24399 (b) M. T. La and H.-K. Kim, *Tetrahedron Letters*., 2018, **59**, 1855-1859.

3 (a) J. Masson-Makdissi, Y. J. Jang, L. Prieto, M. S. Taylor and M. Lautens, *ACS Catal.*, 2019, **9**, 11808-11812; (b) B. M. Trost, J. Xu and T. Schmidt, *J. Am. Chem. Soc.*, 2009, **131**, 18343-18357.

4 Gaussian 16, Revision A.03, M. J. Frisch, G. W. Trucks, H. B. Schlegel, G. E. Scuseria, M. A. Robb, J. R. Cheeseman, G. Scalmani, V. Barone, G. A. Petersson, H. Nakatsuji, X. Li, M. Caricato, A. V. Marenich, J. Bloino, B. G. Janesko, R. Gomperts, B. Mennucci, H. P. Hratchian, J. V. Ortiz, A. F. Izmaylov, J. L. Sonnenberg, D. Williams-Young, F. Ding, F. Lipparini, F. Egidi, J. Goings, B. Peng, A. Petrone, T. Henderson, D. Ranasinghe, V. G. Zakrzewski, J. Gao, N. Rega, G. Zheng, W. Liang, M. Hada, M. Ehara, K. Toyota, R. Fukuda, J. Hasegawa, M. Ishida, T. Nakajima, Y. Honda, O. Kitao, H. Nakai, T. Vreven, K. Throssell, J. A. Montgomery, Jr., J. E. Peralta, F. Ogliaro, M. J. Bearpark, J. J. Heyd, E. N. Brothers, K. N. Kudin, V. N. Staroverov, T. A. Keith, R. Kobayashi, J. Normand, K. Raghavachari, A. P. Rendell, J. C. Burant, S. S. Iyengar, J. Tomasi, M. Cossi, J. M. Millam, M. Klene, C. Adamo, R. Cammi, J. W. Ochterski, R. L. Martin, K. Morokuma, O. Farkas, J. B. Foresman, and D. J. Fox, Gaussian, Inc., Wallingford CT, 2016.

5 C. Y. Legault, CYLview, version 1.0b; Universitéde Sherbrooke: Quebec, Canada, 2009; http://www.cylview.org.

6 (a) Y. Zhao and D. G. Truhlar, *Theor. Chem. Acc.*, 2008, **120**, 215–241; (b) Y. Zhao and D. G. Truhlar, *Acc. Chem. Res.*, 2008, **41**, 157–167.

7 J. Tomasi, and M. Persico, *Chem. Rev.* 1994, **94**, 2027–2094.

8 (a) A. E. Reed and F. Weinhold, *J. Chem. Phys.,* 1983, **78**, 4066–4073; (b) J. P. Foster and F. Weinhold, *J. Am. Chem. Soc.,* 1980, **102**, 7211–7218.
